# Sensory gene identification in the transcriptome of the ectoparasitoid *Quadrastichus mendeli*

**DOI:** 10.1038/s41598-021-89253-w

**Published:** 2021-05-06

**Authors:** Zong-You Huang, Xiao-Yun Wang, Wen Lu, Xia-Lin Zheng

**Affiliations:** Guangxi Key Laboratory of Agric-Environment and Agric-Products Safety, College of Agriculture, Guangxi University, Nanning, 530004 China

**Keywords:** High-throughput screening, Herpetology

## Abstract

Sensory genes play a key role in the host location of parasitoids. To date, the sensory genes that regulate parasitoids to locate gall-inducing insects have not been uncovered. An obligate ectoparasitoid, *Quadrastichus mendeli* Kim & La Salle (Hymenoptera: Eulophidae: Tetrastichinae), is one of the most important parasitoids of *Leptocybe invasa*, which is a global gall-making pest in eucalyptus plantations. Interestingly, *Q. mendeli* can precisely locate the larva of *L. invasa*, which induces tumor-like growth on the eucalyptus leaves and stems. Therefore, *Q. mendeli*–*L. invasa* provides an ideal system to study the way that parasitoids use sensory genes in gall-making pests. In this study, we present the transcriptome of *Q. mendeli* using high-throughput sequencing. In total, 31,820 transcripts were obtained and assembled into 26,925 unigenes in *Q. mendeli*. Then, the major sensory genes were identified, and phylogenetic analyses were performed with these genes from *Q. mendeli* and other model insect species. Three chemosensory proteins (CSPs), 10 gustatory receptors (GRs), 21 ionotropic receptors (IRs), 58 odorant binding proteins (OBPs), 30 odorant receptors (ORs) and 2 sensory neuron membrane proteins (SNMPs) were identified in *Q. mendeli* by bioinformatics analysis. Our report is the first to obtain abundant biological information on the transcriptome of *Q. mendeli* that provided valuable information regarding the molecular basis of *Q. mendeli* perception, and it may help to understand the host location of parasitoids of gall-making pests.

## Introduction

Sensory genes play a key role in the life of parasitoids, such as foraging, oviposition site selection, and mating partners^1^. There are two major chemosensory mechanisms through olfaction and taste in which chemical signals are detected by one of the large multigene families that encode chemosensory proteins (CSPs), gustatory receptors (GRs), ionotropic receptors (IRs), odorant-binding proteins (OBPs), sensory receptors (ORs) and sensory neuron membrane proteins (SNMPs)^2^. The function of CSPs and OBPs is the first step in the recognition of chemical stimuli from the outside environment^3^. Chemoreceptors (such as GRs, IRs and ORs) are involved in the recognition and identification of various chemical signals and environmental odors to modulate chemical perception^4^. SNMPs are involved in cell signal transduction^4^.

Some sensory genes of parasitoids in Hymenoptera have been identified, including Bethylidae, e.g., *Sclerodermus* sp.^2^; Braconidae, e.g., *Cotesia vestalis* Haliday^5^, *Cot. chilonis* Matsumura^6^, *Microplitis demolitor* Wilkinson^7^, *M. mediator* Haliday^8^, *Microcentrus cingulum* Brischke^9^, *Aphidius gifuensis* Ashmead^10^, *Ap. ervi* Haliday^11^ and *Meteorus pulchricornis* Wesmael^12^; Encyrtidae, e.g., *Anastatus japonicus* Ashmead^13^ and *Aenasius bambawalei* Hayat^14^; Eupelmidae, e.g., *Copidosoma floridanum* Ashmead^15^; Ichneumonidae, e.g., *Campoletis chlorideae* Uchida^1^; Trichogrammatidae, e.g., *Trichogramma dendrolimi* Matsumura^16^ and *Tric. japonicun* Ashmead^3^; Eulophidae, e.g., *Asecodes hispinarum* Boucek and *Chouioia cunea* Yang^4,17^. Previous studies have revealed that the sensory genes of parasitoids are involved in searching and locating wood-boring pests and leaf-mining pests^18,19^. For example, *Scleroderma sichuanensis* Xiao can accurately find the location of their hidden host *Monochamus alternatue* Hope and then parasitize them. SsicOBP1 and SsicOBP2 are the basis for the behavior of the odor, which have shown a strong reaction with (−)-α-pinene, (+)-β-pinene, camphene, and (+)-3-carene^20^. However, the sensory genes of parasitoids used to locate gall-making pests have not yet been solved, which has aroused great interest. Understanding this information can provide potential molecular targets for research based on reverse chemical ecology.

*Quadrastichus mendeli* Kim & La Salle (Hymenoptera: Eulophidae) is one of the most important larval ectoparasitoids of *Leptocybe invasa* (Hymenoptera: Eulophidae), which is a global gall-making pest in eucalyptus plantations^21,22^. *Q. mendeli* is a uniparental parasitoid, and no males have been found^23,24^. Female *Q. mendeli* prefers to parasitize young and mature larvae of *L. invasa* with a percentage of parasitism of 84.20 ± 11.40^23^. *Q. mendeli* has now been successfully established and effectively controls *L. invasa* populations in fields in Australia, China, Cambodia, India, Israel, Italy, Kenya, Laos, South Africa, Thailand and Vietnam^25–29^. Previous studies revealed that sensory genes of parasitoids play important roles in locating hosts^8^. Therefore, *Q. mendeli*–*L. invasa* is an ideal system to study the way that parasitoids use sensory genes in gall-making pests.

High-throughput sequencing is massively parallel, high throughput DNA sequencing, which is rapidly changing methodologies of molecular genetic studies. Through high-throughput sequencing, the view for the impact of gene translation can be expand efficiency, and more unknown proteins of parasitoids were found, such as *Sclerodermus* sp.^2^ and *A. bambawalei*^14^. In this study, we performed high-throughput sequencing of the transcriptome and identified members of the major sensory genes that are crucial for *Q. mendeli* to locate *L. invasa.* Comparative analysis of the sensory genes in *Q. mendeli* with those in other species was also examined, and it provided valuable information regarding the molecular basis of *Q. mendeli* perception.

## Materials and methods

### Insects

Branches of saplings damaged by *L. invasa* were collected from Guangxi University (108°29′ E, 22°85′ N), Nanning City, Guangxi Zhuang Autonomous Region, in October 2018. Specimens were placed in a glass container filled with water to retain freshness and transferred to a sealed net cage (length × width × height = 40 cm × 40 cm × 80 cm) with 70–80% relative humidity and a natural light photoperiod maintained at 27 ± 1 °C. The water in the glass container was replaced daily until the end of the emergence of *Q. mendeli*. The emerged *Q. mendeli* were collected daily using 50-mL plastic tubes. One day later, the tubes were immediately placed into liquid nitrogen and stored at − 80 °C. Six groups of female *Q. mendeli* adults (a group of twenty) were prepared for RNA extraction.

### RNA sequencing

A NanoPhotometer spectrophotometer (Thermo Fisher Scientific, Massachusetts, USA) and the Nano6000 Assay Kit for the Agilent Bioanalyzer 2000 system (Agilent Technologies, California, USA) were applied to check the purity and integrity of the total RNA, respectively. After total RNA extraction, magnetic beads with Oligo dT (Thermo Fisher Scientific, Hampton, USA) were used to enrich mRNA, and then, a fragmentation buffer was added to make it a short fragment. The fragments were sequenced on an Illumina HiSeq4000 (Illumina, California, USA).

### Transcriptome data analysis

Reads obtained from the sequencing machines included dirty reads containing adapters or low-quality bases, which affected the subsequent assembly and analysis. De novo transcriptome assembly was carried out with the short read assembly program Trinity v3.0^30^. Basic annotation of unigenes includes protein functional annotation, pathway annotation, COG/KOG functional annotation and Gene Ontology (GO) annotation. To annotate the unigenes, we used the BLASTx program (http://www.ncbi.nlm.nih.gov/BLAST/) with an E-value threshold of 1e−5 for the NCBI nonredundant protein (Nr) database (http://www.ncbi.nlm.nih.gov), the Swiss-Prot protein database (http://www.expasy.ch/sprot), the Kyoto Encyclopedia of Genes and Genomes (KEGG) database (http://www.genome.jp/kegg)^31,32^, and the COG/KOG database (http://www.ncbi.nlm.nih.gov/COG). Protein functional annotations could then be obtained according to the best alignment results^33^.

### Sequence alignment and phylogenetic analysis

TransMembrane prediction using Hidden Markov Models 2.0 (http://www.cbs.dtu.dk/services/TMHMM)^34^. The signal peptides were predicted using SignalP 4.1 (http://www.cbs.dtu.dk/services/SignalP/)^35^. Amino acid sequence alignment was performed using the ClustalW method implemented in Mega v7.0^36^. The *Q. mendeli* CSP, GR, IR, OR, OBP and SNMP nucleotide sequences were used as queries (BLASTx) in the GenBank database, and sequences from different insect species (i.e., *Apis mellifera* Ligustica, *Bombyx mori* Silk, *C. floridanum*, *D. melanogaster*, *M. mediator*, *N. vitripennis, Solenopsis invicta* Buren, *Tribolium castaneum* Herbst and *Tric. Pretiosum* Riley) and their amino acids were retrieved in the GenBank database and used to construct a phylogenetic tree. *A. mellifera*, *B. mori*, *D. melanogaster* and *Trib. castaneum* are model insects; *S. invicta* is a kind of social insect; *C. floridanum*, *M. mediator* and *Tric. pretiosum* were parasitoid from Hymenoptera. Amino acid sequences were aligned using the Muscle method implemented in Mega v7.0^37^. The resulting alignment was manually curated to remove gap-rich regions. Maximum-likelihood trees (for CSP, GR, IR, OR, OBP and SNMP) were constructed using IQ-TREE with the best-fitting substitution model^38^. Subsequently, trees were viewed and graphically edited in FigTree v1.4.3^39^ and Adobe Illustrator CS6. Branch support was assessed using the bootstrap method based on 1000 replicates.

## Results

### Transcriptome assembly and annotation

To obtain high-quality clean reads, raw reads with adapters, low quality, and an N content greater than 10% were removed. The number of clean reads in female adults of *Q. mendeli* ranged from 18,733,252 to 25,259,462, and the sample GC content ranged from 45.06 to 53.01% (Table 1). At the same time, Q20 and Q30 ranged from 92.75 to 94.36% and 88.45 to 90.08% respectively (Table 1). In total, 31,820 transcripts were obtained and assembled into 26,925 unigenes (Additional file 1). A total of 42.10% of unigenes had a length greater than 2000 bp and an average length of 1369 bp (Table 2). The NR database (15,543, 57.73%) had the largest match. In general, the sequences had E-values between 0 and 1E−150 (Table 3). The e-value is an indication of the degree of similarity between the initial sequence used for searches and the sequence retrieved, and the higher the score, the greater the degree of similarity between them. The transcripts of *Q. mendeli* were most similar to the sequences of *Nasonia vitripennis* Walker (26.82%), followed by the sequences of *Ceratosolen solmsi marchali* Mayr (7.49%), *Cop. floridanum* (3.77%), *Tric. pretiosum* (2.92%), and other species (44.27%) (Additional file 2). SwissProt (11,644, 43.25%) and KOG (10,924, 40.57%) shared similar quantities; KO (7524, 27.94%) showed the least match (Table 3).

**Table 1:** Sequencing summary of the *Quadrastichus mendeli* transcriptome.

Sample name	Raw reads	Clean reads	Clean data (Gb)	Q20 (%)	Q30 (%)	GC content
*Q. mendeli* 1	24,740,386	21,110,044	3.71	94.03	89.45	45.79
*Q. mendeli* 2	22,581,498	19,278,188	3.39	94.13	89.65	45.22
*Q. mendeli* 3	29,819,798	25,259,462	4.47	93.85	89.11	45.06
*Q. mendeli* 4	25,051,518	18,733,252	3.76	92.75	88.45	53.01
*Q. mendeli* 5	26,482,834	22,495,242	3.97	93.94	89.31	45.26
*Q. mendeli* 6	26,557,838	22,736,816	3.98	94.36	90.08	45.18

**Table 2:** Number and length of transcripts and unigenes.

Length range/bp	Contig	Transcript	Unigene
0–300	442,916 (95.09%)	7976 (25.07%)	5859 (21.76%)
301–500	5879 (1.26%)	6191 (19.46%)	4816 (17.89%)
501–1000	5696 (1.22%)	5739 (18.04%)	4915 (18.25%)
1001–2000	5660 (1.22%)	5694 (17.89%)	5315 (19.74%)
> 2001	11,297 (2.43%)	11,914 (37.44%)	11,335 (42.10%)
Total number	465,788	31,820	26,925
Total length (bp)	66,378,092	39,430,660	36,871,436
N50 length (bp)	1617	2356	2504
Mean length (bp)	143	1239	1369

**Table 3:** Unigenes annotated in different databases.

	1E − 20 < evalue < = 1E − 5	1E − 50 < evalue < = 1E − 20	1E − 100 < evalue < = 1E − 50	1E − 150 < evalue < = 1E − 100	0 < = evalue < = 1E − 150	Total no	PCT (%)
KO	809	1006	1140	835	3734	7524	27.94
KOG	2027	2342	2545	1540	2470	10,924	40.57
NR	2495	2828	2334	1683	6203	15,543	57.73
Swissprot	2407	2847	2690	1477	2223	11,644	43.25

*NR* NCBI non-redundant protein sequences, *Swissprot* A manually annotated and reviewed protein sequence database, *KO* KEGG Orthology, *KOG* Clusters of Orthologous Groups of proteins, *Total no.* total number of annotated unigenes, *PCT* (*%*) percentage (%).

**Figure 1:**
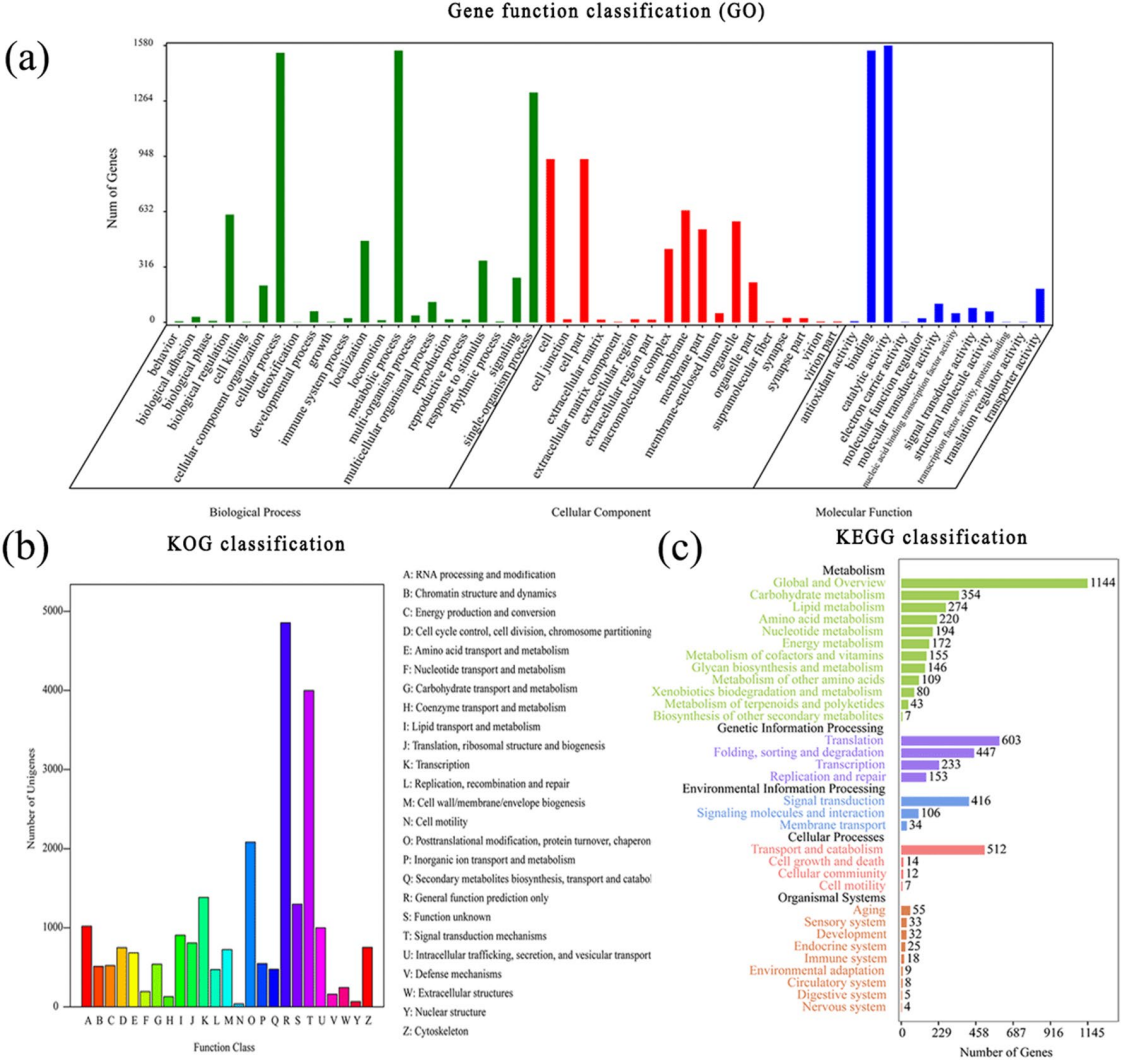
Distribution of transcriptome contigs from *Quadrastichus mendeli* adults. (**a**) Gene Ontology (GO) analysis. (**b**) Eukaryotic Orthologous Groups of protein (KOG) classification. (**c**) Kyoto Encyclopedia of Genes and Genomes (KEGG) classification. (n = 15,623).

In total, 14,735 were annotated into 52 subcategories belonging to three main GO categories: a ‘biological process’, ‘cellular component’ and ‘molecular function’ (Fig. 1a). There were 22 subcategories in the ‘biological process’, 18 subcategories in the ‘cellular component’, and 12 subcategories in the ‘molecular function’. The top ten subcategories were ‘catalytic activity’ (1580), ‘metabolic process’ (1552), ‘binding’ (1552), ‘cellular process’ (1539), ‘single-organism process’ (1312), ‘cell’ (932), ‘cell part’ (932), ‘membrane’ (639), ‘biological regulation’ (615) and ‘organelle’ (576) (Additional file 3). By KOG classifications, 4689 unigenes were classified functionally into 25 categories (Fig. 1b). The cluster of ‘general fractional prediction only’ was the largest group, which had 4855 unigenes. The ‘signal transduction mechanisms’ group was second with 3998 unigenes. The top 2 categories had 36.64% unigenes annotated to the KOG database (Additional file 4). In total, 5160 unigenes were functionally classified into 5 KEGG categories (Fig. 1c). They were ‘cellular processes’ (587 unigenes, 7.66% of the unigenes annotated to the KEGG database), ‘environmental information processing’ (739, 9.64%), ‘genetic information processing’ (1612, 21.03%), ‘metabolism’ (4504, 58.77%) and ‘organismal systems’ (222, 2.90%) (Additional file 4). Among the 31 subcategories, ‘Global and Overview’ (2123, 27.70%), ‘translation’ (664, 8.66%) and ‘Signal transduction’ (599, 7.82%) were the top 3 (Additional file 5).

### Identification of candidate chemosensory genes

**Figure 2:**
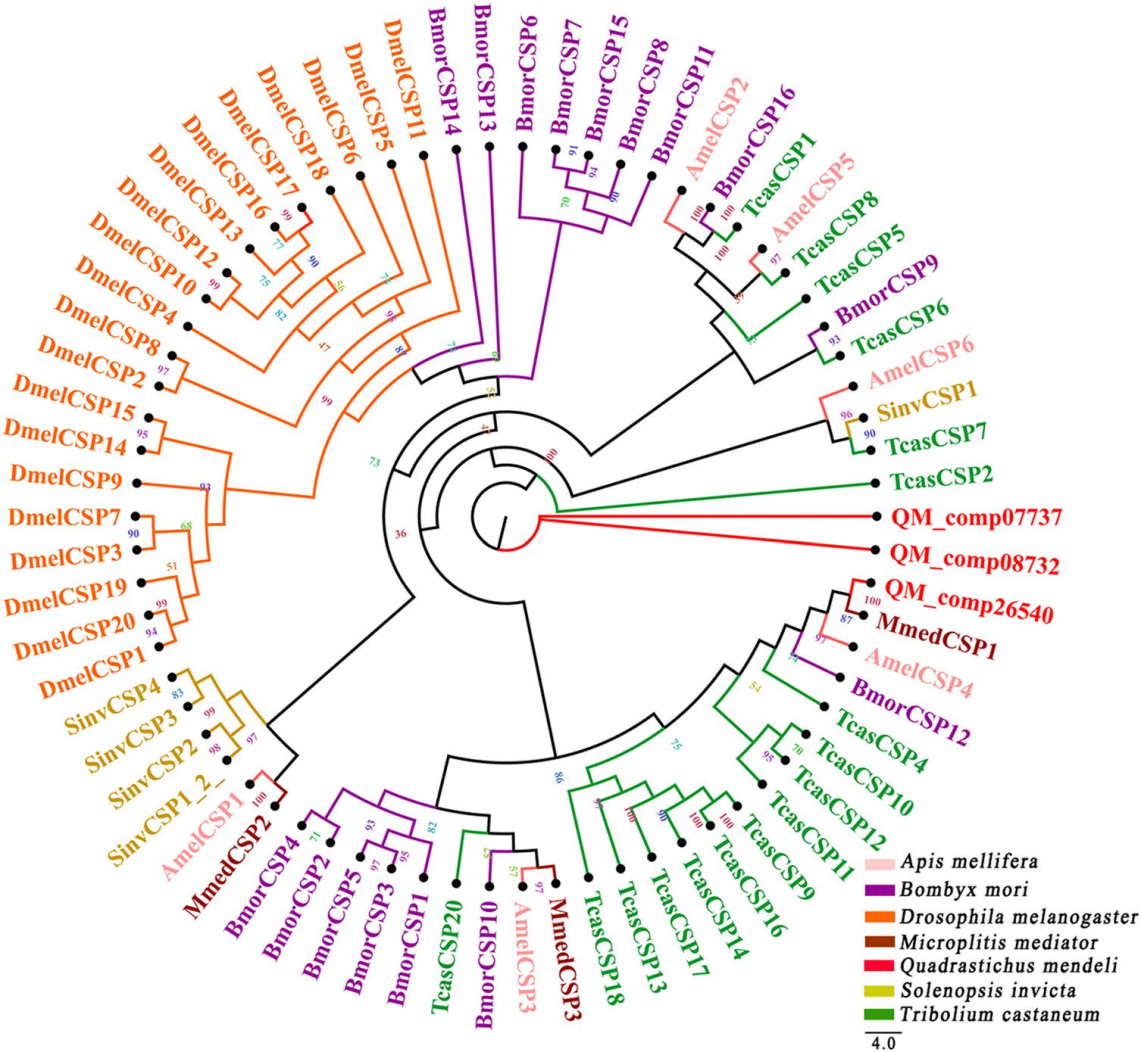
Phylogenetic tree of chemosensory proteins (CSPs) from *Quadrastichus mendeli* and other insects based on the maximum likelihood method. Included are CSPs from *Apis mellifera* (Amel), *Bombyx mori* (Bmor), *Drosophila melanogaster* (Dmel), *Microplitis mediator* (Mmed), *Quadrastichus mendeli* (Qmen), and *Tribolium castaneum* (Tcas). The specific clades are marked. Node support was assessed with 1000 bootstrap replicates.

In this study, 3 putative unigenes encoding CSPs were identified, named QM_comp07737, QM_comp08732 and QM_comp26540 (Additional file 6). The lengths of these unigenes were 509 bp, 608 bp and 280 bp, respectively (Additional file 7). Among these unigenes, QM_comp07737 and QM_comp26540 were incomplete due to a lack of a 5′ or 3′ terminus (Additional file 7). QM_comp08732 sequences were full-length putative CSP genes because they had complete ORFs and 4 cysteines, which are characteristic of typical insect CSPs. QM_comp08732 with a molecular weight of 17 kDa had a signal peptide sequence of approximately 22 amino acids at the N-terminus (Additional file 8). Through a homology search with known proteins, the results showed that 73% of QM_comp26540 was orthologs of the proteins in *Tenebrio molitor* L., and the orthologs of other CSP sequences were also above 60% (Additional file 8). A phylogenetic tree based on the maximum likelihood method was constructed used the 3 CSP sequences of *Q. mendeli* along with 67 CSP sequences from 6 other species (i.e., *A. mellifera*, *B. mori*, *D. melanogaster*, *M. mediator*, *S. invicta* and *Trib. castaneum*) (Fig. 2 and Additional file 9). The phylogenetic tree showed that QM_comp26540 shares a high homology and is closely clustered with MmedCSP1, which has been functionally characterized, and QM_comp07737 and QM_comp08732 did not branch clusters with any other insects; they may be specific CSPs of *Q. mendeli* (Fig. 2).

### Identification of candidate gustatory receptors

**Figure 3:**
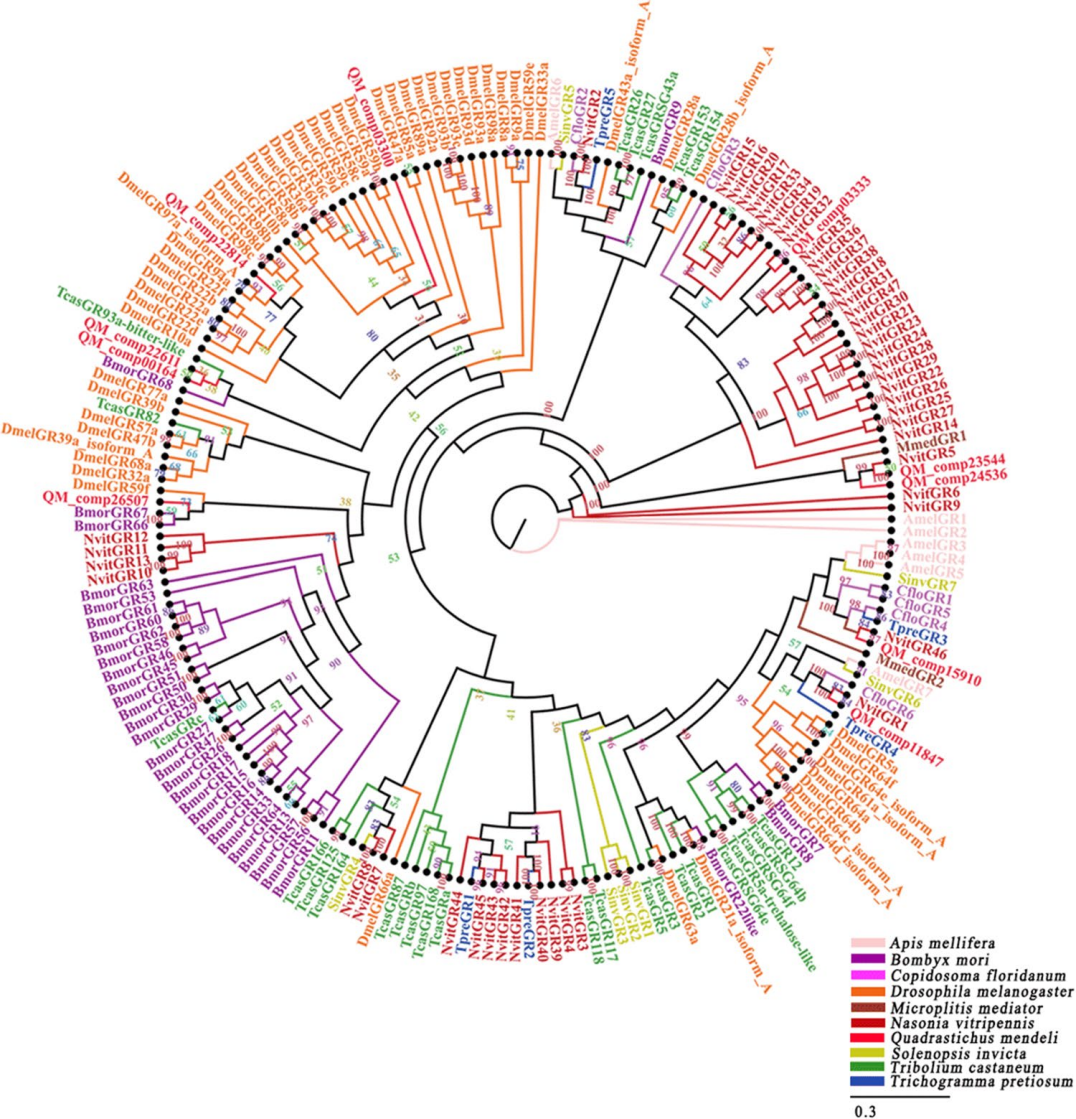
Phylogenetic tree of gustatory receptors (GRs) from *Quadrastichus mendeli* and other insects based on the maximum likelihood method. Included are OBPs from *Apis mellifera* (Amel), *Bombyx mori* (Bmor), *Copidosoma floridanum* (Cflo), *Drosophila melanogaster* (Dmel), *Microplitis mediator* (Mmed), *Nasonia vitripennis* (Nvit), *Quadrastichus mendeli* (Qmen), *Tribolium castaneum* (Tcas), and *Trichogramma pretiosum* (Tpre). The specific clades are marked. Node support was assessed with 1000 bootstrap replicates.

Ten candidate GR proteins were identified from the data sets (Additional file 6). Among these unigenes, QM_comp03300, QM_comp23544, QM_comp24536 and QM_comp26507 were incomplete due to the lack of a 5′ or 3′ terminus (Additional file 7). QM_comp00164, QM_comp03333, QM_comp11847, QM_comp15910, QM_comp22611 and QM_comp22814 sequences were full-length putative GR genes because they had complete ORFs. These unigenes had molecular weights that ranged between 4 and 56 kDa and had a signal peptide sequence that ranged between 15 and 41 amino acids at the N-terminus (Additional file 8). Through a homology search with known proteins, the results showed that 79% of QM_comp15910 were orthologs of the proteins in *Trichomalopsis sarcophagae* Gahan. A phylogenetic tree based on the maximum likelihood method was constructed used the 10 GR sequences of *Q. mendeli* along with 191 GR sequences from 9 other species (i.e., *A. mellifera*, *B. mori*, *C. floridanum*, *D. melanogaster*, *M. mediator*, *N. vitripennis*, *S. invict*, *Trib. castaneum* and *Tric. pretiosum*) (Fig. 3 and Additional file 9). The phylogenetic tree showed that most GRs of *Q. mendeli* shared high homology and closely clustered with the proteins in *B. mori* (Fig. 3).

### Identification of candidate ionotropic receptors

**Figure 4:**
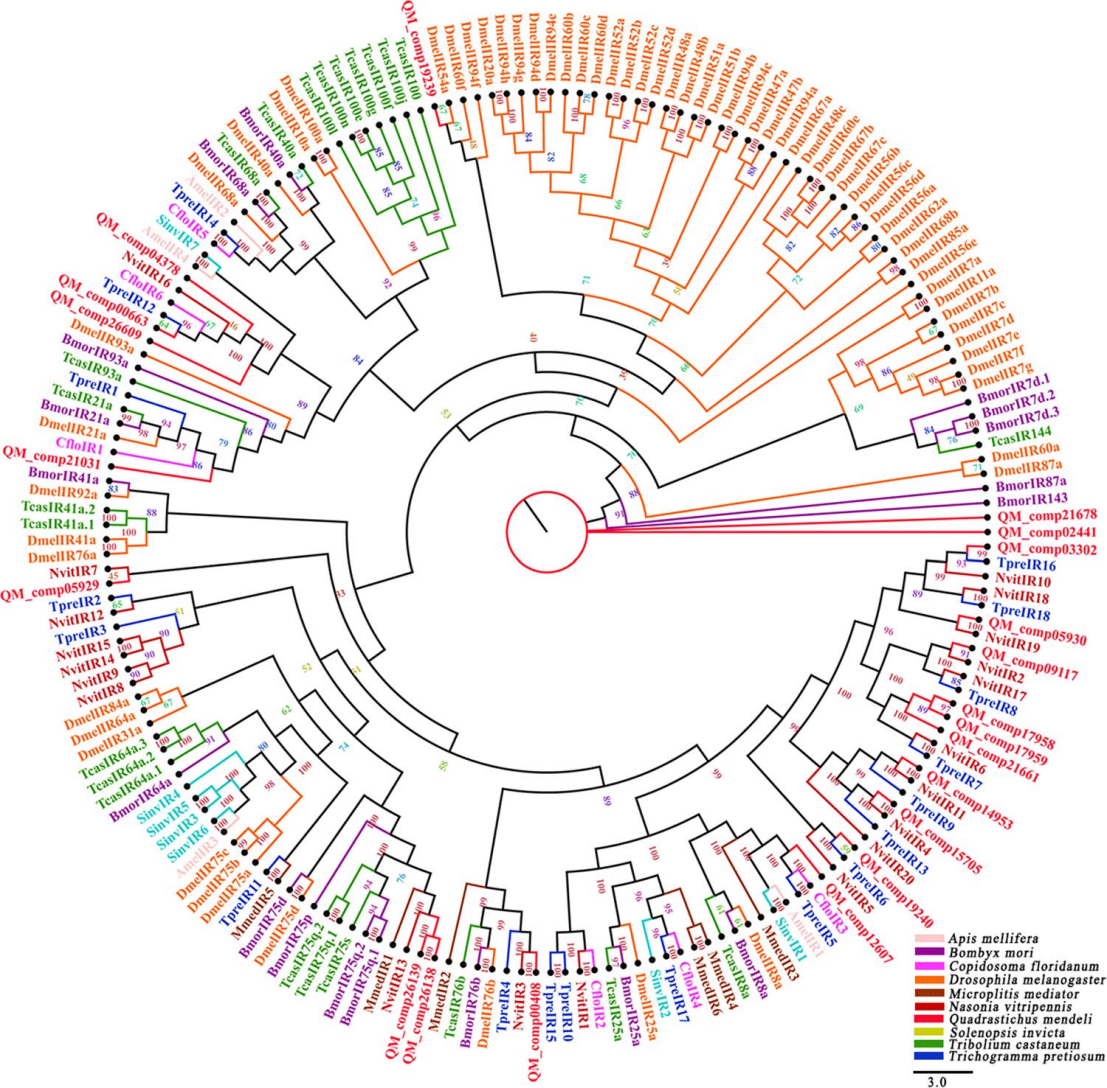
Phylogenetic tree of ionotropic receptors (IRs) from *Quadrastichus mendeli* and other insects based on the maximum likelihood method. Included are IRs from *Apis mellifera* (Amel), *Bombyx mori* (Bmor), *Copidosoma floridanum* (Cflo), *Drosophila melanogaster* (Dmel), *Microplitis mediator* (Mmed), *Nasonia vitripennis* (Nvit), *Quadrastichus mendeli* (Qmen), *Tribolium castaneum* (Tcas), and *Trichogramma pretiosum* (Tpre). The specific clades are marked. Node support was assessed with 1000 bootstrap replicates.

Twenty-one candidate IR proteins were identified from the data sets (Additional file 6). Among these unigenes, 13 unigenes were incomplete due to the lack of a 5′ or 3′ terminus (Additional file 7). Eight unigenes were full-length putative IR genes because they had complete ORFs. These unigenes had molecular weights that ranged between 5 and 12 kDa (Additional file 8). Through a homology search with known proteins, the results showed that 100% of the IRs in *Q. mendeli* were orthologs of the proteins in *Asbolus verrucosus* LeConte, and the orthologs of other IR sequences were also above 37% (Additional file 8). A phylogenetic tree based on the maximum likelihood method was constructed used the 21 IR sequences of *Q. mendeli* along with 131 IR sequences from 9 other species (i.e., *A. mellifera*, *B. mori*, *C. floridanum*, *D. melanogaster*, *M. mediator*, *N. vitripennis*, *S. invict*, *Trib. castaneum* and *Tric. pretiosum*) (Fig. 4 and Additional file 9). The 21 IRs of *Q. mendeli* along with 131 IRs from 9 other species (i.e., *A. mellifera*, *B. mori*, *C. floridanum*, *D. melanogaster*, *M. mediator*, *N. vitripennis*, *S. invict*, *Trib. castaneum* and *Tric. pretiosum*) were chosen to construct a phylogenetic tree based on the amino acid sequences (Additional file 9). The phylogenetic tree showed that all candidate IR proteins were clustered with at least one Hymenoptera ortholog (Fig. 4).

### Identification of candidate odorant binding proteins

**Figure 5:**
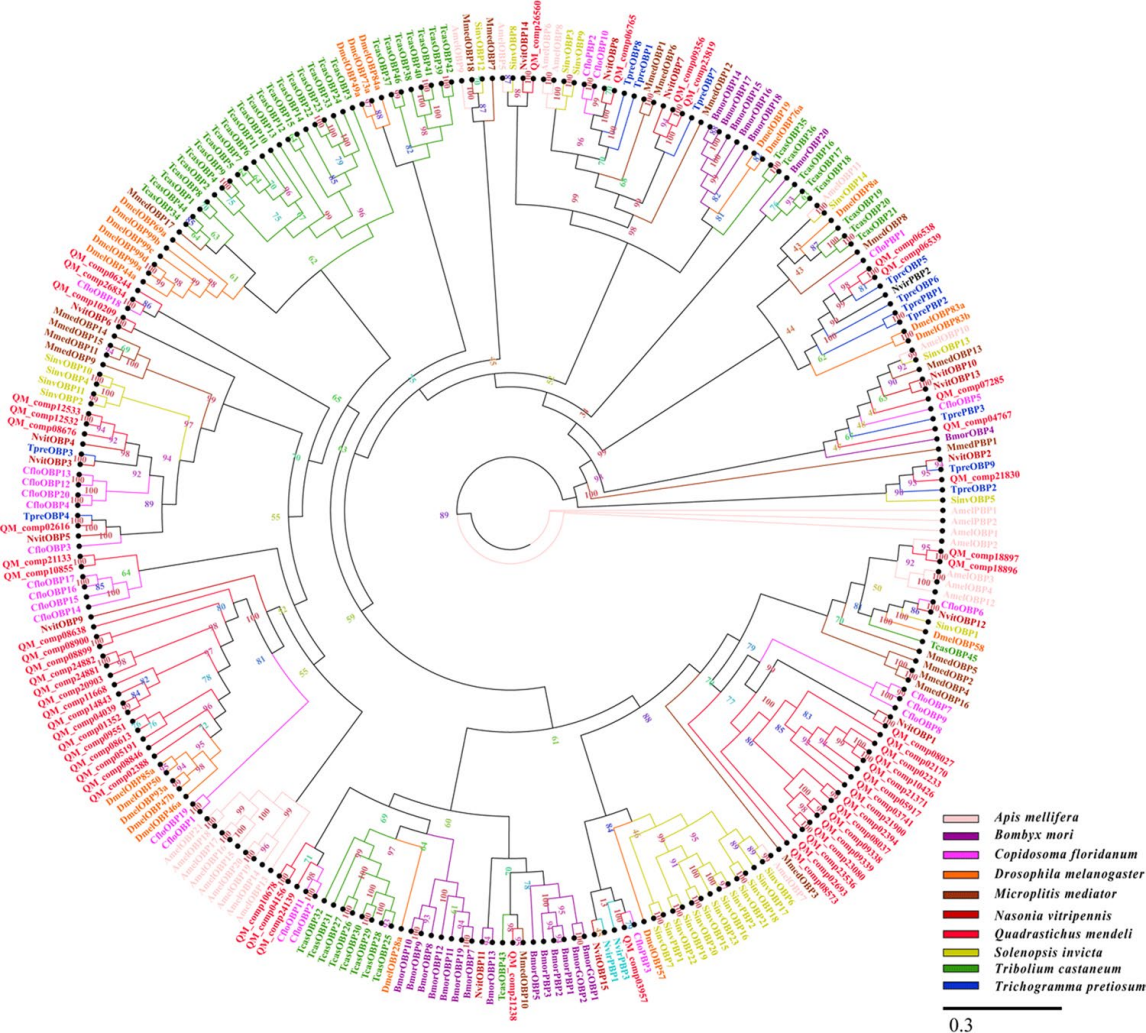
Phylogenetic tree of odorant binding proteins (OBPs) from *Quadrastichus mendeli* and other insects based on the maximum likelihood method. Included are OBPs from *Apis mellifera* (Amel), *Bombyx mori* (Bmor), *Copidosoma floridanum* (Cflo), *Drosophila melanogaster* (Dmel), *Microplitis mediator* (Mmed), *Nasonia vitripennis* (Nvit), *Quadrastichus mendeli* (Qmen), *Tribolium castaneum* (Tcas), and *Trichogramma pretiosum* (Tpre). The specific clades are marked. Node support was assessed with 1000 bootstrap replicates.

Fifty-six candidate OBP proteins were identified from the data sets (Table 4; Additional file 6). Among these unigenes, 10 unigenes were incomplete due to the lack of a 5′ or 3′ terminus (Additional file 7). Forty-six unigenes were full-length putative OBP genes because they had complete ORFs. These unigenes had molecular weights ranging between 10 and 17 kDa and had a signal peptide sequence ranging between 16 and 23 amino acids at the N-terminus (Additional file 8). Insect OBPs can be classified into classical OBPs (six-cysteine conserved signature) and Minus-C (missing C2 and C5) and Plus-C (carries an additional conserved cysteine located between C1 and C2 and after C6). QM_comp02388, QM_comp07285, QM_comp08846, QM_comp10855, QM_comp21133, QM_comp21238 and QM_comp24139 had four conserved cysteines, which were minus-COBPs. Other OBPs had six conserved cysteines, which are classic OBPs. Plus-COBPs were not found in hymenopteran species. Through a homology search with known proteins, the results showed that 88% of QM_comp19239 were orthologs of the proteins in *N. vitripennis*, and the orthologs of other OBP sequences were also above 42% (Additional file 8). A phylogenetic tree based on the maximum likelihood method was constructed used the 56 OBP sequences of *Q. mendeli* along with 209 OBP sequences from 9 other species (i.e., *A. mellifera*, *B. mori*, *C. floridanum*, *D. melanogaster*, *M. mediator*, *N. vitripennis*, *S. invict*, *Trib. castaneum* and *Tric. pretiosum*) (Fig. 5 and Additional file 9). The phylogenetic tree showed that all candidate OBP proteins were clustered with at least one Hymenoptera ortholog (Fig. 5).

**Table 4:** Detailed information on the OBP ungenes of *Quadrastichus mendeli*.

Unigene ID	Unigene length (bp)	ORF length (aa)	Complete ORF	5′ or 3′ terminus lost	Signal peptide	Signal peptide (aa)	Cysteine number	FPKM (Mean)	Homology search with known proteins			
									Identity (%)	E value	Species	Protein ID
QM_comp01352	728	132	Yes	–	Yes	18	8	14.67	37	2e−20	*C. floridanum*	XP_014204137.1
QM_comp02170	549	129	Yes	–	Yes	19	7	234.80	53	2e−21	*N. vitripennis*	XP_001600573.1
QM_comp02233	411	129	No	3′	Yes	21	8	0.32	34	2e−07	*C. floridanum*	XP_014212211.1
QM_comp02388	1415	125	Yes	–	No	–	5	1.67	58	1e−150	*Habropoda laboriosa*	KOC59862.1
QM_comp02394	438	130	Yes	–	Yes	19	8	17.91	43	5e−27	*N. vitripennis*	XP_001600573.1
QM_comp02616	679	157	Yes	–	No	–	9	203.66	68	2e−46	*N. vitripennis*	XP_001601182.1
QM_comp02693	407	118	No	3′	Yes	19	7	5.73	52	7e−26	*N. vitripennis*	XP_001600573.1
QM_comp03741	518	129	Yes	–	Yes	19	7	1.12	40	6e−18	*N. vitripennis*	XP_001600573.1
QM_comp04039	793	133	Yes	–	Yes	18	9	5.00	34	1e−08	*C. floridanum*	XP_014204137.1
QM_comp04767	321	41	No	3′	No	–	0	0.58	48	2e−09	*C. floridanum*	XP_014204137.1
QM_comp05191	915	113	Yes	–	Yes	20	10	10.24	41	7e−12	*C. floridanum*	XP_014204137.1
QM_comp05917	727	133	Yes	–	Yes	19	6	35.75	41	5e−14	*N. vitripennis*	XP_001600573.1
QM_comp06244	433	127	No	3′	Yes	17	8	8.89	63	5e−45	*T. dendrolimi*	ANG08504.1
QM_comp06765	917	150	Yes	–	Yes	23	7	3.90	52	2e−41	*Ceratosolen solmsi marchali*	XP_011505749.1
QM_comp07285	729	92	Yes	–	No	–	4	1.40	86	1e−09	*C. floridanum*	XP_014206764.1
QM_comp08027	538	140	Yes	–	Yes	19	8	113.56	49	2e−39	*N. vitripennis*	XP_001600573.1
QM_comp08037	736	134	Yes	–	Yes	19	9	192.11	37	9e−24	*N. vitripennis*	XP_001600573.1
QM_comp08573	1211	133	Yes	–	Yes	21	6	4.55	36	2e−06	*C. floridanum*	XP_014212211.1
QM_comp08613	542	113	Yes	–	Yes	20	9	21.22	44	2e−12	*C. floridanum*	XP_014204137.1
QM_comp08638	476	130	Yes	–	Yes	20	7	311.84	51	2e−25	*C. floridanum*	XP_014204137.1
QM_comp08676	507	135	Yes	–	Yes	17	7	275.44	69	6e−63	*N. vitripennis*	XP_001601068.1
QM_comp08846	492	125	Yes	–	Yes	20	7	62.90	38	6e−08	*C. floridanum*	XP_014204137.1
QM_comp08899	580	128	Yes	–	Yes	18	7	43.75	35	1e−12	*N. vitripennis*	XP_001606346.1
QM_comp08900	484	129	Yes	–	Yes	18	7	125.85	36	2e−13	*N. vitripennis*	XP_001606346.1
QM_comp09338	510	131	Yes	–	Yes	19	11	59.68	42	4e−23	*N. vitripennis*	XP_001600573.1
QM_comp09339	507	131	Yes	–	Yes	19	9	176.04	51	3e−19	*N. vitripennis*	XP_001600573.1
QM_comp09356	647	145	Yes	–	Yes	22	6	4.84	53	5e−51	*N. vitripennis*	XP_001603472.2
QM_comp09551	688	108	Yes	−	Yes	20	9	9.27	43	1e−08	*C. floridanum*	XP_014204137.1
QM_comp10209	523	137	Yes	–	Yes	17	7	36.04	45	5e−30	*N. vitripennis*	XP_001601290.1
QM_comp10426	498	124	Yes	–	Yes	19	9	7.66	33	1e−08	*N. vitripennis*	XP_001600573.1
QM_comp10855	879	144	Yes	–	No	–	4	8.33	28	7e−07	*N. vitripennis*	XP_001601068.1
QM_comp11668	700	126	Yes	–	Yes	18	8	45.36	34	7e−12	*C. floridanum*	XP_014204137.1
QM_comp12532	510	135	Yes	–	Yes	16	7	3.52	74	6e−57	*N. vitripennis*	XP_001601068.1
QM_comp12533	669	135	Yes	–	Yes	16	7	21.25	74	2e−56	*N. vitripennis*	XP_001601068.1
QM_comp14843	1465	125	Yes	–	Yes	19	8	144.20	44	4e−14	*C. floridanum*	XP_014204137.1
QM_comp18897	568	132	Yes	–	Yes	20	9	2.74	26	4e−06	*N. vitripennis*	XP_001600573.1
QM_comp20903	596	130	Yes	–	Yes	20	11	13.86	27	1e−05	*C. floridanum*	XP_014206340.1
QM_comp21133	508	141	Yes	–	No	–	7	0.95	29	3e−05	*Ceratosolen solmsi marchali*	XP_011505723.1
QM_comp21238	482	104	Yes	–	No	–	2	0.86	88	1e−43	*N. vitripennis*	XP_016845336.1
QM_comp21371	321	100	No	3′	Yes	19	6	1.40	36	3e−09	*N. vitripennis*	XP_001600573.1
QM_comp21830	1294	144	Yes	–	Yes	23	9	73.07	79	9e−65	*N. vitripennis*	XP_001600769.1
QM_comp21900	238	41	No	3′	No	–	1	0.67	51	2e−16	*N. vitripennis*	XP_001600573.1
QM_comp23080	223	73	No	3′	Yes	19	5	0.59	32	1e−06	*N. vitripennis*	XP_001600573.1
QM_comp23536	487	145	Yes	–	Yes	19	5	0.99	28	6e−07	*T. pretiosum*	XP_014221963.1
QM_comp23819	378	120	No	3′	Yes	22	4	0.65	49	4e−29	*N. vitripennis*	XP_001603472.2
QM_comp24139	1063	142	Yes	–	Yes	18	4	197.63	26	3e−04	*C. floridanum*	XP_014206340.1
QM_comp24881	456	124	Yes	–	Yes	17	8	3.80	26	3e−04	*C. floridanum*	XP_015603383.1
QM_comp24882	483	133	Yes	–	Yes	17	8	1.79	28	2e−04	*T. pretiosum*	XP_014221963.1
QM_comp26560	572	145	Yes	–	Yes	22	6	40.28	69	5e−66	*N. vitripennis*	XP_016842824.1
QM_comp26834	545	126	Yes	–	Yes	17	8	249.66	73	8e−49	*C. floridanum*	AHE40949.1
QM_comp03957	562	132	Yes	–	Yes	20	7	12.82	46	6e−28	*C. floridanum*	XP_014208150.1
QM_comp04156	497	139	No	5	Yes	20	9	93.54	30	2e−10	*N. vitripennis*	XP_016845238.1
QM_comp06538	520	139	Yes	–	Yes	20	7	2.40	77	8e−67	*T. dendrolimi*	ANG08495.1
QM_comp06539	593	139	Yes	–	Yes	20	7	43.82	73	1e−52	*T. pretiosum*	XP_014224061.1
QM_comp10678	1344	136	Yes	–	Yes	20	6	21.55	51	4e−41	*T. sarcophagae*	OXU16757.1
QM_comp18896	1057	154	Yes	–	Yes	20	9	5.18	41	4e−25	*C. floridanum*	XP_014208127.1

### Identification of candidate odorant receptors

**Figure 6:**
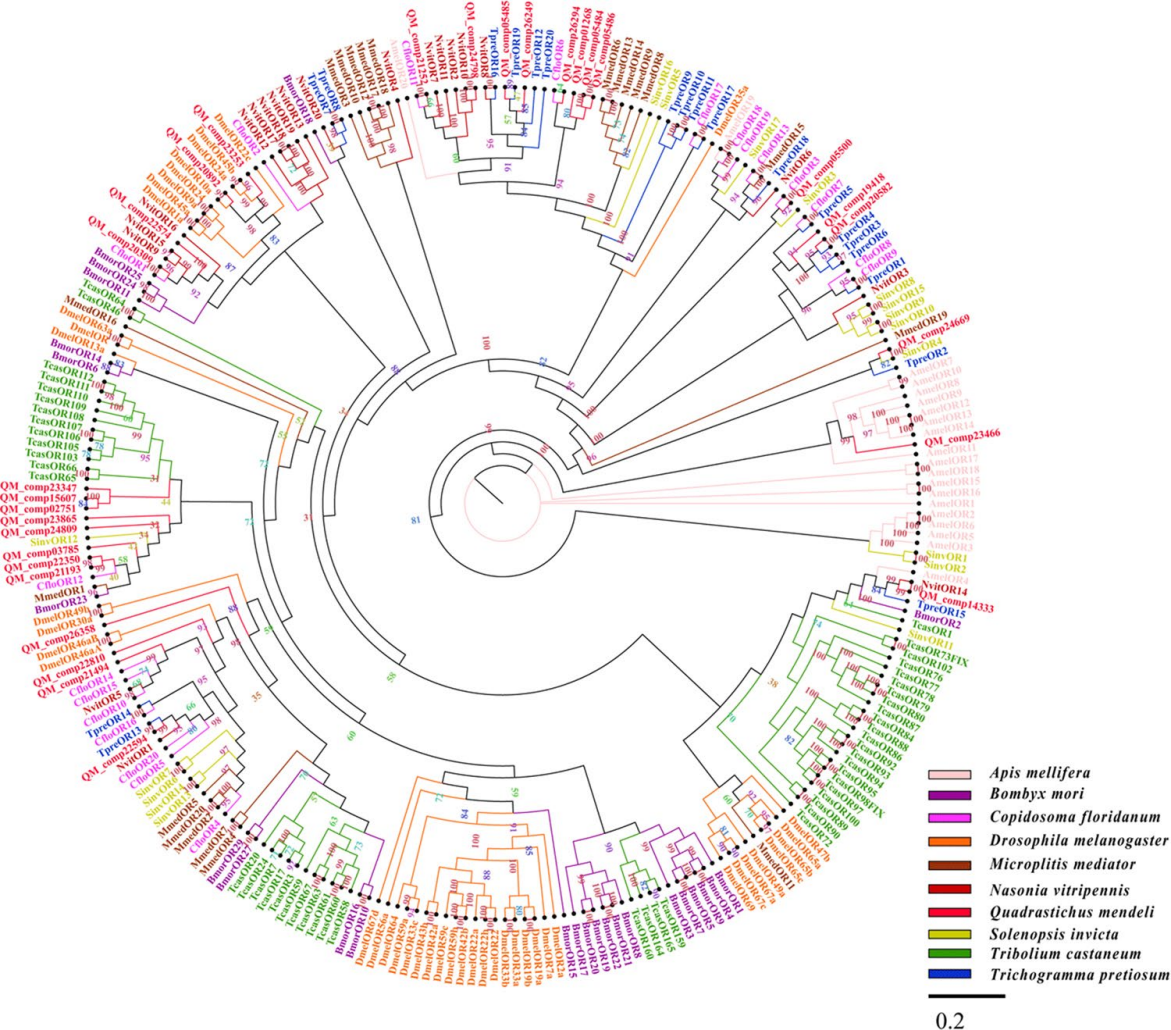
Phylogenetic tree of odorant receptors (ORs) from *Quadrastichus mendeli* and other insects based on the maximum likelihood method. Included are ORs from *Apis mellifera* (Amel), *Bombyx mori* (Bmor), *Copidosoma floridanum* (Cflo), *Drosophila melanogaster* (Dmel), *Microplitis mediator* (Mmed), *Nasonia vitripennis* (Nvit), *Quadrastichus mendeli* (Qmen), *Tribolium castaneum* (Tcas), and *Trichogramma pretiosum* (Tpre). The specific clades are marked. Node support was assessed with 1000 bootstrap replicates.

Thirty candidate OR proteins were identified from the data sets (Additional file 6). Among these unigenes, 21 unigenes were incomplete due to the lack of a 5′ or 3′ terminus (Additional file 7). Nine unigenes were full-length putative OR genes because they had complete ORFs. These unigenes had molecular weights ranging between 4 and 53 kDa and had a signal peptide sequence ranging between 16 and 23 amino acids at the N-terminus (Additional file 8). Through a homology search with known proteins, the results showed that 95% of QM_comp14333 were orthologs of the proteins in *C. cunea*, and the orthologs of other OR sequences were also above 32% (Additional file 8). A phylogenetic tree based on the maximum likelihood method was constructed used the 30 OR sequences of *Q. mendeli* along with 235 OR sequences from 9 other species (i.e., *A. mellifera*, *B. mori*, *C. floridanum*, *D. melanogaster*, *M. mediator*, *N. vitripennis*, *S. invict*, *Trib. castaneum* and *Tric. pretiosum*) (Fig. 6 and Additional file 9). The phylogenetic tree showed that all candidate OR proteins were clustered with at least one Hymenoptera ortholog (Fig. 6).

### Identification of candidate sensory neuron membrane receptors

**Figure 7:**
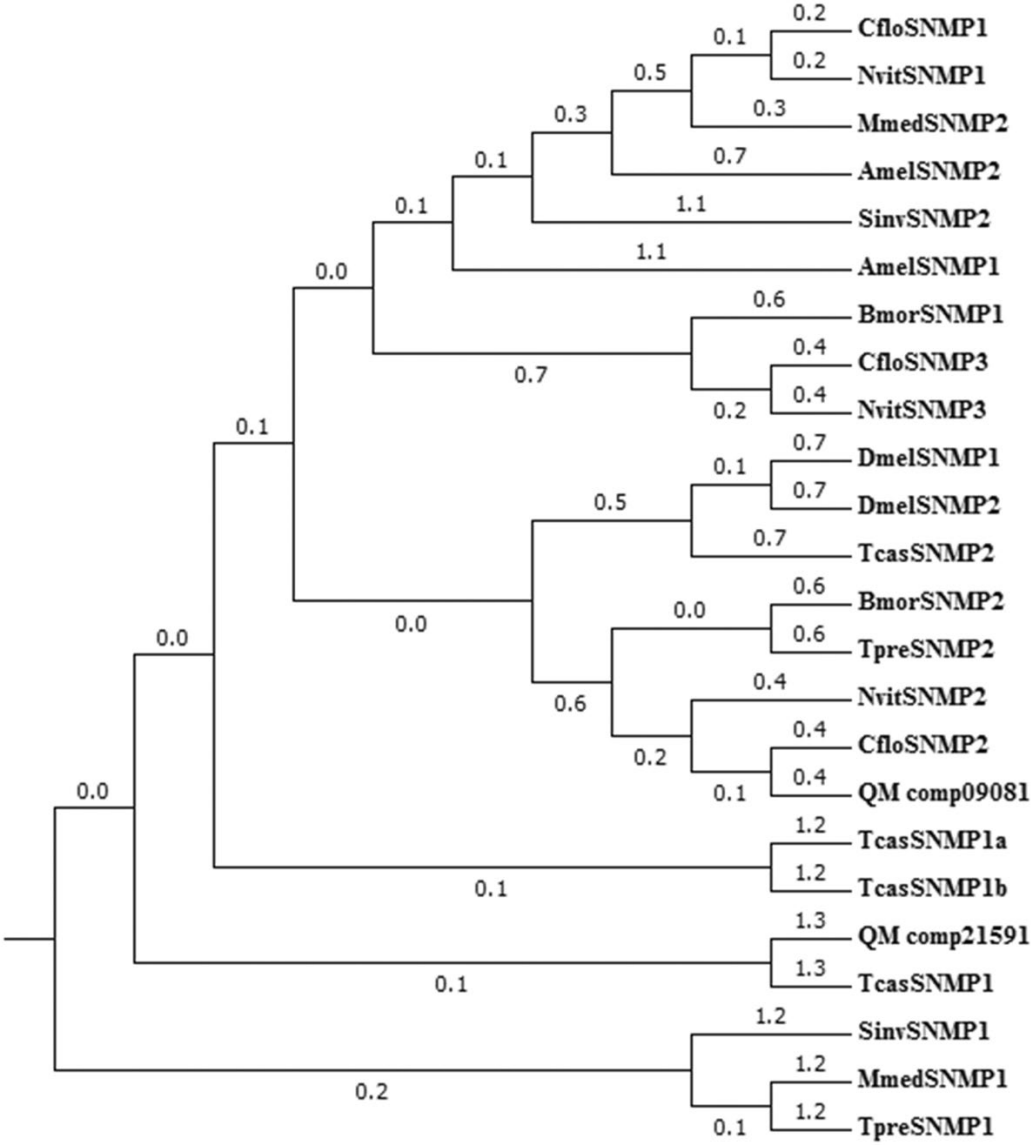
Phylogenetic tree of sensory neuron membrane proteins (SNMPs) from *Quadrastichus mendeli* and other insects based on the maximum likelihood method. Included are SNMPs from *Apis mellifera* (Amel), *Bombyx mori* (Bmor), *Copidosoma floridanum* (Cflo), *Drosophila melanogaster* (Dmel), *Microplitis mediator* (Mmed), *Nasonia vitripennis* (Nvit), *Quadrastichus mendeli* (Qmen), *Tribolium castaneum* (Tcas), and *Trichogramma pretiosum* (Tpre). The specific clades are marked. Node support was assessed with 1000 bootstrap replicates.

Two candidate SNMP proteins were identified from the data sets (Additional file 6). QM_comp21591 was incomplete due to the lack of a 3′ terminus (Additional file 7). QM_comp09081 was a full-length putative SNMP gene because it had complete ORFs. QM_comp09081 had a signal peptide sequence of 13 amino acids at the N-terminus, and the molecular weight of QM_comp09081 was 59 kDa (Additional file 8). According to sequence similarity, SNMP is divided into two SNMP subtypes, SNMP1 and SNMP2. Through a homology search with known proteins, the results showed that 67% of QM_comp09081 were orthologs of the proteins in *N. vitripennis* (Additional file 8). A phylogenetic tree based on the maximum likelihood method was constructed used the 2 SNMP sequences along with 25 SNMP sequences from 9 other species (i.e., *A. mellifera*, *B. mori*, *C. floridanum*, *D. melanogaster*, *M. mediator*, *N. vitripennis*, *S. invict*, *Trib. castaneum* and *Tric. pretiosum*) (Fig. 7 and Additional file 9). The phylogenetic tree showed that QM_comp21591 fell into the same clade as the insect SNMP1 group, and QM_comp09081 fell into the same clade as the insect SNMP2 group (Fig. 7).

## Discussion

In this study, the major sensory genes (i.e., CSPs, GRs, IRs, OBPs, ORs and SNMPs), which perhaps regulate *Q. mendeli* to locate its host *L. invasa,* are first reported, providing valuable information for exploring how parasitoids use sensory genes to locate gall-making pests.

CSPs are widespread in the antenna and other chemical sensory organs of insects^40^ and are involved in the chemical perception and related behavior of insects^41^. Compared to the total number of insects in the world, CSPs have been identified in only a few species of insects to different degrees, such as Coleoptera^42^, Hemiptera^43^, Hymenoptera^12^, Lepidoptera^44^ and Orthoptera^45^, whose numbers show interspecific diversity. For example, the number of CSPs varies from 4 CSPs in *Drosophila melanogaster* to 22 in *B. mori*^46^. In this study, 3 candidate CSPs were identified, which are less than those in the parasitoids *Ch. cunea* (11), *Sclerodermus* sp. (10), *Me. pulchricornis* (8) and *Tric. dendrolimi* (7)^2,4,12,16^ and are greater than those in the parasitoids *Cot. chilonis* (2) and *Ap. ervi* (2)^6,11^. Previous research confirmed that generalists seem to be specifically suited for the processing of odorant mixtures, and they respond in a similar manner to plant volatiles^47^. For example, *Ch. cunea* is a generalist and has multiple hosts (e.g., *Stilpnotia salicis* L., *Ivela ochropoda* Eversmann, *Clostera anachoreta* Fabricius, *Semiothisa cinerearia* Bremer & Gray and *Clania variegeta* Snellen), while *Cot. chilonis* mainly parasitizes larvae of the genus *Chilo* Zincken^48,49^. It can be deduced that the number of CSPs relates to their host range. For the specialist *Q. mendeli*, the use of CSPs is expected to cope with the variability in host availability^46^. Previous studies also revealed that CSPs could be involved in the solubilization of hydrocarbons in the stratum corneum to recognize its companion^50^. Therefore, CSPs in *Q. mendeli* should be associated with its obligate parasitic characteristics, indicating that QmenCSPs may function in the chemical sensing of *L. invasa* and its shelter host eucalyptus trees. A similar story has been confirmed for *M. mediator*, which can accurately find and then parasitize its hidden host *Agrotis segetum* Denis and Schiffermüller^51^. The phylogenetic tree showed that QM_comp07737 and QM_comp08732 did not branch clusters with any other insects and QM_comp26540 was in the same clade as MmedCSP1 of *M. mediator*. The result showed that QM_comp07737 and QM_comp08732 may be specific CSPs of *Q. mendeli*^4^, and their functions needed to be further explored. QM_comp26540 had the closest relationship with MmedCSP1, which has a strong reaction with methyl salicylate, pentane, ocimene, *β*-ionone, 3,4-dimethylbenzaldehyde, 2-hexanone and cis-3-hexe-1-ol^51^. Previous research suggested that MmedCSP1 can function in chemical sensing of the plant volatiles of *M. mediator*’s host *A. segetum*^51^. Encouragingly, significant GC-EAD responses of *Q. mendeli* antenna to eucalyptus volatile α-phellandrene and 1,8-cineole were observed^52^. Thus, QM_comp26540 may function as a chemosensor, which is involved in the process of recognizing plant volatiles from eucalyptus when *Q. mendeli* searches its host, *L. invasa*.

GRs are widespread in gustatory organs of insects that respond to various taste-related soluble compounds, and cuticular hydrocarbons and odorants, such as sugars, amino acids, salts, bitter compounds, CO_2_ and pheromones, can be recognized and combined by GRs^53,54^. To date, GRs in some model insects with genome reports have been identified, such as *A. gambiae* (76), *B. mori* (69), *D. melanogaster* (68) and *N. vitripennis* (58)^55,56^. In this study, 10 candidate GRs were identified, which was similar to other parasitoids, such as *An. japonicus* (8)^13^, *Sclerodermus* sp. (6)^2^ and *M. mediator* (6)^8^. This could be attributed to the sequencing depth and species-functional specificity of GRs^53^. DmelGR5 and DmelGR64 in *D. melanogaster* are receptor proteins for sweet taste and are used to detect glucose, sucrose, maltose, maltitol and cottonseed sugar^57^. For *Q. mendeli*, females that were fed a honey solution or honey solution + young eucalyptus leaves lived for a longer time than those who underwent other treatments, including flowers, gall leaves, water, galled leaves + honey solution, no food and young leaves^23^. Therefore, GRs in *Q. mendeli* should play a key role in recognizing sugar and fresh eucalyptus leaves via various soluble compounds^58^. The phylogenetic tree showed that QM_comp00164 and QM_comp22611 were the same clade as the proteins in *Trib. castaneum*, QM_comp03300, QM_comp22814, and QM_comp26507 were the same clade as the proteins in *D. melanogaster*, and other proteins in *Q. mendeli* were the same clade as the proteins in *N. vitripennis.* Thus, GRs of *Q. mendeli* may share high homology and closely cluster with the proteins in *D. melanogaster*, *N. vitripennis*, and *Trib. castaneum.* Interestingly, QM_comp11847 was in the same clade as the sugar receptor NvitGR1, which was used to recognize the only source of nutrients from host *Lucilia caesar* L. for the offspring of *N. vitripennis*^59,60^. Thus, QM_comp11847 may be involved in recognizing host organisms and sugars, which helps *Q. mendeli* to quickly access energy from these molecules.

IRs, which evolve from the ionotropic glutamate receptor (iGluR), are a new class of sensory proteins mainly in taste organs/sensilla that respond to food components, such as sugars, salts, water and bitter compounds, and detect small temperature differences^61–63^. In this study, 21 candidate IRs were identified, which was more than in the parasitoids *Me. pulchricornis* (19)^12^, *Ch. cunea* (10)^4^, *M. mediator* (6)^8^, *Sclerodermus* sp. (3)^2^, *Mi. cingulum* (3)^9^ and *An. japonicus* (3)^13^. Physiological recordings from taste sensilla in *D. Melanogaster* and other insects have revealed responses of taste neurons to salts, sugars, water, bitter compounds and a large diversity of other tastants^61,62^. Taste sensilla are widely distributed on the antennae of *Q. mendeli*^28^, suggesting that QmenIRs may function as taste receptors. The phylogenetic tree showed that QmenIRs were spread across the tree branches and clustered with homologous IRs from other species, which suggested that QmenIRs may be functionally conserved. QM_comp21031 was located in the same clade as BmorIR21a of *B. mori*, DmelIR21a of *D. melanoga*ster and TcasIR21a of *Trib. castaneum*, indicating that QM_comp21031 has the closest relationship with insect IR21a, which can mediate cool sensing in *Drosophila*^64^. Thus, QM_comp21031 may perceive changes in temperature since insect IR21a can achieve both heat avoidance and heating^65,66^. Taking the oviposition features into consideration, we deduced that female *Q. mendeli* may be capable of sensing surface heat on galls related to *L. invasa* damage, which requires further exploration.

OBPs are crucial in insect olfactory perception and are the first step in the recognition of chemical stimuli from the outside environment^3^. In some model insects, OBPs have been identified to different degrees, such as *B. mori* (57)^67^, *D. melanoga*ster (51)^68^, *Trib. castaneum* (46)^69^ and *A. gambiae* (44)^70^. In this study, 56 candidate OBPs were identified, which was more than in the parasitoids *Ae. bambawalei* (54)^14^, *Ch. cunea* (25)^4^, *Tric. dendrolimi* (24)^16^, *M. mediator* (20)^8^, *Me. pulchricornis* (16)^12^, *T. japonicum* (15)^3^, *Ap. ervi* (15)^11^, *Sclerodermus* sp. (10)^2^, *Cop. floridanum* (8)^15^, *Cot. chilonis* (8)^6^ and *As. hispinarum* (8)^17^. The number of *Q. mendeli* OBPs identified was less than that in the parasitoid *Cot. vestalis* (74)^5^. Previous studies revealed that OBPs in parasitoids play a key role in binding and transporting hydrophobic odorants from the environment to sensory receptors^71^. In *Q. mendeli*, a significant behavioral response to the gall volatiles d-limonene and decanal was observed (unpublished data). Relevant QmenOBPs function in chemical sensing of these volatiles characterizing *L. invasa* and its shelter host eucalyptus trees, which bioinformatics analysis could help to target. The phylogenetic tree showed that 15 QmenOBPs were the same clade as 5 DmelOBPs of *D. melanogaster*, and 15 QmenOBPs were the same clade as NvitOBP1 of *N. vitripennis*, and 3 CfloOBPs of *C. floridanum*, which suggested that most OBPs of *Q. mendeli* may share high homology and closely cluster with the proteins in *C. floridanum*, *D. melanogaster*, and *N. vitripennis*. The evolutionary relationship of *Q. mendeli* OBPs, as inferred in the phylogenetic tree, indicated that they are orthologous sequences due to the absence of monophyletic groups. Our results showed that QM_comp21238 is the same clade as MmedOBP10 of *M. mediator*, which is involved in the process of recognizing *β*-ionone and Nonanal when they find the location of their hidden host *A. segetu*^51,72^. Thus, QM_comp21238 may also be involved in the process of recognizing similar odorants or ligands when *Q. mendeli* locates its shelter host, *L. invasa*.

ORs are thought to play critical roles in the perception of chemosensory stimuli by insects^54^. The number of ORs in parasitoids vary greatly^1,4^. In this study, 30 candidate ORs were identified, which was more than in the parasitoid *Tric. dendrolimi* (9)^16^, *Mi. cingulum* (9)^9^ and *Sclerodermus* sp. (8)^2^. Previous studies revealed that the OR of *M. mediator* play an important role in recognizing plant volatiles, such as nonanal and farnesene, which provided a key start to manipulate and develop ORs in wasps to find hosts and use them as biological tools for pest control^73^. The phylogenetic tree showed that QmenORs were spread across the tree branches and clustered with homologous ORs from other species, which suggested that QmenORs may be functionally conserved. The RNAi investigation of the role of MmedOrco, the *M. mediator* ortholog of *Drosophila* Or83b, supported the assumption that this highly conserved gene plays a similar role in insects^73,74^. QmenORs may function as chemoreceptors to recognize plant volatiles from eucalyptus. Our results showed that QM_comp20892 is in the same clade as DmelOR10a of *D. melanoga*ster, which plays a role in responding to odorants such as methylsalicylate and acetophenone^75,76^. For *Q. mendeli*, QM_comp20892 may be involved in the process of recognizing similar odorants or ligands.

SNMPs are involved in cellular signal transduction and play a role in odor detection^5^. Two SNMPs are normally broadly identified in different insects, e.g., parasitoids *Ch. cunea* and *Sclerodermus* sp.^2,4^. It has been reported that SNMP1 and SNMP2 are both expressed in antennae sensilla and have different expression patterns^4,77^. In *Ch. cunea*, CcunSNMP1 is a morphine receptor of neurons, and CcunSNMP2 is mainly expressed in supporting cells and the lymph of antennal sensilla^4^, while the location and expression patterns of SNMPs in *Q. mendeli* should be further studied since this information should be associated with their functions. SNMP1 of *D. melanogaster* is involved in pheromone detection and enhances the Ca^2+^ responses served in signal transduction^78^. SNMP1 of *M. mediator* was determined to participate in both pheromone and general odor detection^79^. In contrast, the general functional mechanism of SNMP2 in parasitoids is still poorly understood. The phylogenetic tree showed that QM_comp21591 was the same clade as TcasSNMP1 of *Trib. castaneum* and QM_comp09081 was the same clade as CfloSNMP2 of *C. floridanum*, suggesting that QM_comp21591 and QM_comp09081 shall be SNMP1 and SNMP2 in *Q. mendeli* respectively. In addition, *Q. mendeli* is a uniparental parasitoid that is not required in the male search for mating^22^. Thus, the function of QM_comp21591 and QM_comp09081 may include an oviposition pheromone receptor rather than a sex receptor and a membrane protein with unknown functions, which needs to be further explored.

Chemical detection involves a series of complicated processes that require participation and interactions by multiple cascades of sensory proteins. Insect sensory proteins are capable of functional cooperation and division. Firstly, OBPs and CSPs are both chemically binding proteins to various odorants and can also respond to the same chemicals, e.g., MmedOBP10 and MmedCSP1 are involved in the process of recognizing *β*-ionone and nonanal when they find the location of their hidden host *A. segetu*^51,72^. Secondly, ORs and IRs are both chemoreceptors, while there are differences in the process of recognizing odor substances^80^. For example, IRs are better at detecting long-lasting odor pulses, and they are less sensitive, suggesting that they are better at close-range odor detection. In contrast, ORs are more sensitive and better at resolving brief (low molecular flux) pulsed stimuli^80,81^. Moreover, features of functional organization have emerged between behavioral response profiles of OBPs and electrophysiological response profiles of ORs^75^. Therefore, the sensory genes in *Q. mendeli* should systematically act on the process of locating their gall-making host, and the biological functions of these genes and their products are still poorly known. Overall the sensory genes of the wasp reported here provide valuable insight into the molecular mechanisms of olfaction, which help pave the way for the host location of *Q. mendeli* in gall-making pests.

## Supplementary Information

Additional File 1.

Additional File 2.

Additional File 3.

Additional File 4.

Additional File 5.

Additional File 6.

Additional File 7.

Additional File 8.

Additional File 9.

Additional File 10.

## References

[1] Sun Y (2018). An odorant receptor mediates the attractiveness of cis-jasmone to *Campoletis chlorideae*, the endoparasitoid of *Helicoverpa armigera*. Insect Mol. Biol..

[2] Zhou CX, Min SF, Yan LT, Wang MQ (2015). Analysis of antennal transcriptome and odorant binding protein expression profiles of the recently identified parasitoid wasp, *Sclerodermus* sp. Comp. Biochem. Phys. D..

[3] Wu JD, Shen ZC, Hua HQ, Zhang F, Li YX (2017). Identification and sex expression profiling of odorant-binding protein genes in *Trichogramma japonicum* (Hymenoptera: Trichogrammatidae) using RNA-Seq. Appl. Entomol. Zool..

[4] Zhao Y (2016). Transcriptome and expression patterns of chemosensory genes in antennae of the parasitoid wasp *Chouioia cunea*. PLoS ONE.

[5] Nichols Z, Vogt RG (2008). The SNMP/CD36 gene family in Diptera, Hymenoptera and Coleoptera: *Drosophila**melanogaster*, *D*. *pseudoobscura*, *Anopheles**gambiae*, *Aedes**aegypti*, *Apis**mellifera*, and *Tribolium**castaneum*. Insect Biochem. Mol. Biol..

[6] Qi Y (2014). Transcriptome analysis of an endoparasitoid wasp *Cotesia chilonis* (Hymenoptera: Braconidae) reveals genes involved in successful parasitism. Arch. Insect Biochem..

[7] Zhou X (2015). Chemoreceptor evolution in Hymenoptera and its implications for the evolution of eusociality. Genome Biol. Evol..

[8] Wang SN (2018). Characterization of antennal chemosensilla and associated odorant binding as well as chemosensory proteins in the parasitoid wasp *Microplitis mediator* (Hymenoptera: Braconidae). Sci. Rep..

[9] Ahmed T, Zhang T, Wang Z, He K, Bai S (2016). Gene set of chemosensory receptors in the polyembryonic endoparasitoid *Macrocentrus cingulum*. Sci. Rep..

[10] Kang ZW (2017). Identification and expression analysis of chemosensory receptor genes in an aphid endoparasitoid *Aphidius gifuensis*. Sci. Rep..

[11] Ballesteros GI (2017). Expression differences in *Aphidius ervi* (Hymenoptera: Braconidae) females reared on different aphid host species. PeerJ.

[12] Sheng S (2017). Candidate chemosensory genes identified in the endoparasitoid *Meteorus pulchricornis* (Hymenoptera: Braconidae) by antennal transcriptome analysis. Comp. Biochem. Phys. D..

[13] Li H (2018). cDNA cloning, sequence analysis and expression profile of a chemosensory protein from the *Clostera restitura* (Lepidoptera: Notodontidae). Sci. Silvae Sin..

[14] Nie XP (2018). Antennal transcriptome and odorant binding protein expression profiles of an invasive mealybug and its parasitoid. J. Appl. Entomol..

[15] Donnell DM (2014). Analysis of odorant-binding protein gene family members in the polyembryonic wasp, *Copidosoma floridanum*: Evidence for caste bias and host interaction. J. Insect Physiol..

[16] Zhang SF (2016). Sensory and immune genes identification and analysis in a widely used parasitoid wasp *Trichogramma dendrolimi* (Hymenoptera: Trichogrammatidae). Insect Sci..

[17] Li K (2015). Identification of putative odorant binding protein genes in *Asecodes hispinarum*, a parasitoid of coconut leaf beetle (*Brontispa longissima*) by antennal RNA-Seq analysis. Biochem. Biophys. Res. Commun..

[18] Sullivan BT, Pettersson EM, Seltmann KC, Berisford CW (2000). Attraction of the bark beetle parasitoid *Roptrocerus xylophagorum* (Hymenoptera: Pteromalidae) to host-associated olfactory cues. Environ. Entomol..

[19] Zou, D. Y. Studies on the role of sensilla and mines in host location in *Diglyphus isaea*. Master’ thesis (Chin Acad. Agri. Sci., 2009) **(in Chinese with English abstract)**.

[20] Zhu, X. Q. Binding characterization of OBP1 and OBP2 in the *Scleroderma sichuanensis* Xiao and behavior verification. Master’ thesis (Sichuan Agricultural University, 2017) **(in Chinese with English abstract)**.

[21] Mendel Z, Protasov A, Fisher N, La Salle J (2004). Taxonomy and biology of *Leptocybe invasa* gen. & sp. n. (Hymenoptera: Eulophidae), an invasive gall inducer on eucalyptus. Aust. J. Entomol..

[22] Huang ZY, Li J, Lu W, Zheng XL, Yang ZD (2018). Parasitoids of the eucalyptus gall wasp *Leptocybe invasa*: A global review. Environ. Sci. Pollut. Res..

[23] Kim IK, Mendel Z, Protasov A, Blumberget D, La Salle J (2008). Taxonomy, biology, and efficacy of two Australian parasitoids of the eucalyptus gall wasp, *Leptocybe invasa* Fisher & La Salle (Hymenoptera: Eulophidae: Tetrastichinae). Zootaxa.

[24] Zheng XL (2016). Parasitoids of the eucalyptus gall wasp *Leptocybe invasa* (Hymenoptera: Eulophidae) in China. Parasite.

[25] Dittrich-Schröder G (2014). Biology and host preference of *Selitrichodes neseri*: A potential biological control agent of the eucalyptus gall wasp, *Leptocybe invasa*. Biol. Control.

[26] Mendel Z, Protasov A, La Salle J (2017). Classical biological control of two eucalyptus gall wasps main outcome and conclusions. Biol. Control.

[27] Bush SJ (2018). First record of *Quadrastichus mendeli*, a parasitoid of *Leptocybe invasa* in South Africa. South Forests.

[28] Huang ZY (2018). Ultrastructure of female antennal sensilla of an endoparasitoid wasp, *Quadrastichus mendeli* Kim & La Salle (Hymenoptera: Eulophidae: Tetrastichinae). Microsc. Microanal..

[29] Lawson, S. *et al*. Biological control of galling pests in eucalypt plantations in the Mekong region. Preprint at http://aciar.gov.au/project/fst/2012/091 (2014).

[30] Grabherr MG (2011). Full-length transcriptome assembly from RNA-Seq data without a reference genome. Nat. Biotechnol..

[31] Kanehisa M, Sato Y (2019). KEGG Mapper for inferring cellular functions from protein sequences. Protein Sci..

[32] Kanehisa M, Furumichi M, Sato Y, Ishiguro-Watanabe M, Tanabe M (2021). KEGG: Integrating viruses and cellular organisms. Nucleic Acids Res..

[33] Conesa A (2005). Blast2GO: A universal tool for annotation, visualization and analysis in functional genomics research. Bioinformatics.

[34] Krogh A, Larsson B, von Heijne G, Sonnhammer EL (2001). Predicting transmembrane protein topology with a hidden Markov model: Application to complete genomes. J. Mol. Biol..

[35] Petersen TN, Brunak S, von Heijne G, Nielsen H (2011). SignalP 4.0: Discriminating signal peptides from transmembrane regions. Nat. Methods.

[36] Thompson JD, Gibson TJ, Higgins DG (2002). Multiple sequence alignment using ClustalW and ClustalX. Curr. Protoc. Bioinform..

[37] Kumar S, Stecher G, Tamura K (2016). MEGA7: Molecular evolutionary genetics analysis version 7.0 for bigger datasets. Mol. Biol. Evol..

[38] Trifinopoulos J, Nguyen LT, Von HA, Minh BQ (2016). W-IQ-TREE: A fast online phylogenetic tool for maximum likelihood analysis. Nucleic Acids Res..

[39] Wu Z (2016). Differential expression analysis of chemoreception genes in the striped flea beetle *Phyllotreta striolata* using a transcriptomic approach. PLoS ONE.

[40] Picimbon JF, Dietrich K, Breer H, Krieger J (2000). Chemosensory proteins of *Locusta migratoria* (Orthoptera: Acrididae). Insect Biochem. Mol..

[41] Sánchez-Gracia A, Vieira FG, Rozas J (2009). Molecular evolution of the major chemosensory gene families in insects. Heredity.

[42] Li XM (2009). Candidate chemosensory genes identified in *Colaphellus bowringi* by antennal transcriptome analysis. BMC Genomics.

[43] Song YQ (2009). Analysis of the antennal transcriptome and chemoreception-related genes of the bean bug, *Riptortus pedestris* (Hemiptera: Alydidae). Acta Entomol. Sin..

[44] Wang XY, Xiong M, Lei CL, Zhu F (2015). The developmental transcriptome of the synanthropic fly *Chrysomya megacephala* and insights into olfactory proteins. BMC Genomics.

[45] Zhou, Y. T. Identification and function analysis of olfactory proteins in *Oedaleus asiaticus* (Orthoptera: Acrididae). Master’ thesis (Inner Mongolia University, 2019) **(in Chinese with English abstract)**.

[46] Vieira FG, Rozas J (2011). Comparative genomics of the odorant-binding and chemosensory protein gene families across the Arthropoda: Origin and evolutionary history of the chemosensory system. Genome Biol. Evol..

[47] Steidle, J. L. M. & Van Loon, J. J. A. In *Chemoecology of Parasitoid and Predator Oviposition Behavior* (eds Hilker, M. & Meiners, T.) 291–317 (Blackwell, 2002).

[48] Zheng YN, Qi JY, Sun SH, Yang CC (2012). Advance in research of *Chouioia cunea* Yang (Hymenoptera: Eulophidade) and its biocontrol application in China. Chin. J. Biol. Control.

[49] Yuan X, Fan HL, Li DS (2017). Effects of insect-resistant transgenic rices on enemies in paddy fields: A review. Guangdong Agric. Sci..

[50] Ozaki M (2005). Ant nestmate and nonnestmate discrimination by a chemosensory sensillum. Science.

[51] Zhang S, Zhang YJ, Su HH, Gao XW, Guo YY (2009). Binding characterization of chemosensory protein MmedCSP1 in *Microplitis mediator* (Hymenoptera: Braconidae). Acta Entomol. Sin..

[52] Shivaraju, C. Bio-intensive management of invasive eucalyptus gall wasp, *Leptocybe invasa* Fisher & La Salle (Eulophidae: Hymenoptera). PhD dissertation, (University of Agricultural Sciences, Bengaluru, 2012). http://krishikosh.egranth.ac.in/handle/1/66048.

[53] Hill CA (2002). G protein-coupled receptors in *Anopheles gambiae*. Science.

[54] Jiao YC, Moon SJ, Montell C (2007). A *Drosophila* gustatory receptor required for the responses to sucrose, glucose, and maltose identified by mRNA tagging. Proc. Natl. Acad. Sci..

[55] Scott K (2001). A chemosensory gene family encoding candidate gustatory and olfactory receptors in *Drosophila*. Cell.

[56] Ebbs ML, Amrein H (2007). Taste and pheromone perception in the fruit fly *Drosophila melanogaster*. Pflugers Arch..

[57] Kent LB, Robertson HM (2009). Evolution of the sugar receptors in insects. BMC Evol. Biol..

[58] Feng, M. X. Study on biology and ecology of *Quadrastichus mendeli* Kim & La Salle (Hymenoptera: Eulophidae). Master’ thesis (Hainan University, 2016) **(in Chinese with English abstract)**.

[59] Robertson HM, Gadau J, Wanner KW (2010). The insect chemoreceptor superfamily of the parasitoid jewel wasp *Nasonia vitripennis*. Insect Mol. Biol..

[60] Blaul B, Ruther J (2011). How parasitoid females produce sexy sons: A causal link between oviposition preference, dietary lipids and mate choice in *Nasonia*. Proc. R. Soc. B..

[61] Koh TW (2014). The *Drosophila* IR20a clade of ionotropic receptors are candidate taste and pheromone receptors. Neuron.

[62] Liman ER, Zhang YV, Montell C (2014). Peripheral coding of taste. Neuron.

[63] Chen C (2015). *Drosophila* ionotropic receptor 25a mediates circadian clock resetting by temperature. Nature.

[64] Ni L (2016). The ionotropic receptors IR21a and IR25a mediate cool sensing in *Drosophila*. Elife.

[65] Li ZB (2020). Identification of leg chemosensory genes and sensilla in the *Apolygus lucorum*. Front. Physiol..

[66] Greppi C (2020). Mosquito heat seeking is driven by an ancestral cooling receptor. Science.

[67] Gong DP, Zhang HJ, Zhao P, Xia QY, Xiang ZH (2009). The odorant binding protein gene family from the genome of silkworm, *Bombyx mori*. BMC Genomics.

[68] Hekmat-Scafe DS (2002). Genome-wide analysis of the odorant-binding protein gene family in *Drosophila melanogaster*. Genome Res..

[69] Richards S (2008). The genome of the model beetle and pest *Tribolium castaneum*. Nature.

[70] Xu PX, Zwiebel LJ, Smith DP (2003). Identification of a distinct family of genes encoding atypical odorant-binding proteins in the malaria vector mosquito, *Anopheles gambiae*. Insect Mol. Biol..

[71] Zhou Y (2020). Expression and functional characterization of odorant binding protein genes in the endoparasitic wasp *Cotesia vestalis*. Insect Sci..

[72] Li K (2014). Odorant binding characteristics of three recombinant odorant binding proteins in *Microplitis mediator* (Hymenoptera: Braconidae). J. Chem. Ecol..

[73] Li KM, Ren LY, Zhang YJ, Wu KM, Guo YY (2012). Knockdown of *Microplitis mediator* odorant receptor involved in the sensitive detection of two chemicals. J. Chem. Ecol..

[74] Jones WD, Nguyen TA, Kloss B, Lee KJ, Vosshall LB (2005). Functional conservation of an insect odorant receptor gene across 250 million years of evolution. Curr. Biol..

[75] Swarup S, Williams TI, Anholt RRH (2011). Functional dissection of odorant binding protein genes in *Drosophila melanogaster*. Genes Brain Behav..

[76] Crava CM, Sassù F, Tait G, Becher PG, Anfora G (2019). Functional transcriptome analyses of *Drosophila suzukii* antennae reveal mating-dependent olfaction plasticity in females. Insect Biochem. Mol. Biol..

[77] Gu SH (2013). Molecular identification and differential expression of sensory neuron membrane proteins in the antennae of the black cutworm moth *Agrotis ipsilon*. Insect Physiol..

[78] Halty-deLeon L, Miazzi F, Kaltofen S, Hansson BS, Wicher D (2016). The mouse receptor transporting protein RTP1S and the fly SNMP1 support the functional expression of the *Drosophila* odorant coreceptor Orco in mammalian culture cells. J. Neurosci. Methods.

[79] Shan S (2019). Molecular characterization and expression of sensory neuron membrane proteins SNMPs in the parasitoid *Microplitis mediator* (Hymenoptera: Braconidae). Insect Sci..

[80] Rytz R, Croset V, Benton R (2013). Ionotropic receptors (IRs): Chemosensory ionotropic glutamate receptors in *Drosophila* and beyond. Insect Biochem. Mol..

[81] Getahun MN, Wicher D, Hansson BS, Olsson SB (2012). Temporal response dynamics of *Drosophila* olfactory sensory neurons depends on receptor type and response polarity. Front. Cell. Neurosci..

