# Supplementary figures and images for: Sensory gene identification in the transcriptome of the ectoparasitoid Quadrastichus mendeli
Source: Sci Rep. 2021 May 6;11:9726. doi: 10.1038/s41598-021-89253-w (PMC8102506; doi:10.1038/s41598-021-89253-w)

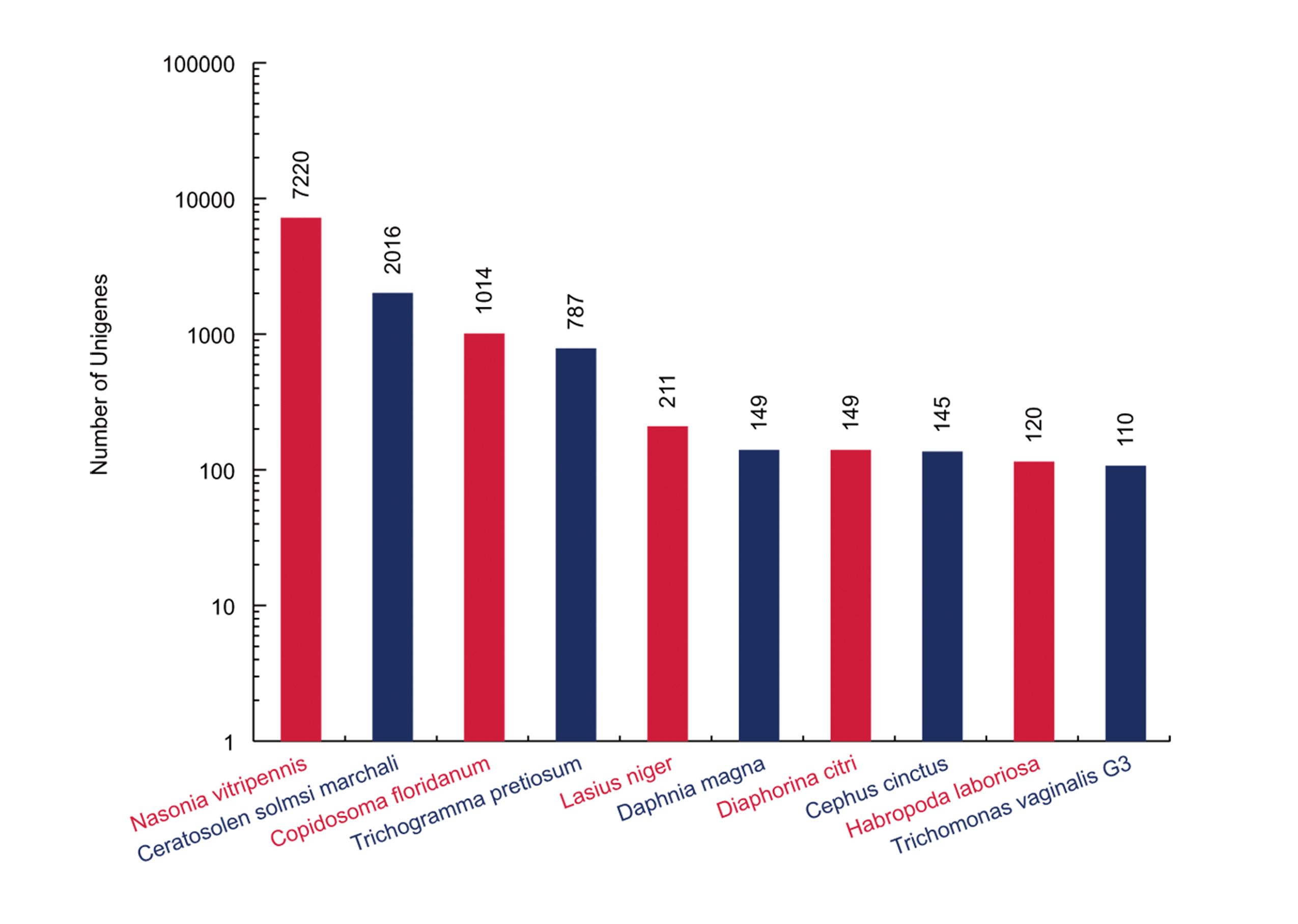

Supplement: Supplementary file 2 — Additional File 2. [file 41598_2021_89253_MOESM2_ESM.gif]
