# Supplementary material for: Sensory gene identification in the transcriptome of the ectoparasitoid Quadrastichus mendeli
Source: Sci Rep. 2021 May 6;11:9726. doi: 10.1038/s41598-021-89253-w (PMC8102506; doi:10.1038/s41598-021-89253-w)
Supplement: Supplementary file 7 — Additional File 7. [file 41598_2021_89253_MOESM7_ESM.doc]

**Additional file 6. The sensory protein fasta sequences of *Q. mendeli*.**

**1. CSP protein fasta sequences of *Q. mendeli***

Complete ORF:

>QM_comp08732

MSGHRAYLLALTVAVLFLAGLVVAQDATVQSAEVTTEVPSVESTTIGEFYPLSWVKVNYKPIVDNERLFRKYKQCLLAEKSVGCPSNVIQLKNLLPEVFETLCAKCLPAHIERVKEAVEYICKTRRDDYDEVRKAKDPDGSLQKKFEDKFGAVNC-

Incomplete ORF:

>QM_comp07737

MYRTLGFVFLSVLVIFSSKVSSSDDDSETSGKKSLKTLKISDLFDKSKMEHHYPEIWTEVDYKPIVDNNRLFKKYKECILAEKLTGCPRIVAEIQKLIPEIVESMCAKCLPIHIERFKEAVSYICHKRREDYDDFRRLRDPDGSIQARFEEKYGKLDC-

>QM_comp26540

MYKVFALAVCCVVIASCVAKPQTKKYTTKYDNIDLEEIIANERLLKNYYNCLMDEGKCTPDGSELKRLLPDALENECKSC

**2. GR oprotein fasta sequences of *Q. mendeli***

**Complete ORF:**

>QM_comp00164

MEGHSINNSTAFSLPTTNTLE-

>QM_comp03333

MTFNEVEFTVVVVFSLDYSLLISMVSSMITYLVIIFQFQSEN-

>QM_comp11847

MYYKDEVENAYKLQKVTVGDEVMTVDAKCKKNNNRNETKKHSPGFGSRMLSIFGSSTSGKGRIFKSPTLAKVNAVQPRPAMQNRRFSPNDDDPECFHRAISNVLLLAQFFGLLPINGIRARSVRRFSFKKFSPRVIYSYCILFAVGVMTSISVFHLFKTLDANSFSTKGGIADATVGAIFYGNSLLGNIMFLRLCPRWISIQYDWRAMERLLDNNAGIARCKRPALRWRLNLIAGIILSLALIEHILSEINNTPMEVFGRNHTFEDFLVVYTNKSHRFIVKHVNYNFTLGLFIFVVSKIATFTWNFTDLFIMMVSTGLAERYKCLNERVTKVSPAQLSATDWQELRESYAALSALVKKVDNEISSIILLSFGNNIYFICLQLLNGLAPNGTGNEIINSIYFFGSFIFLIGRTTSVTLLCARINDECKIILPALYNCPSMNYNLEAQRLQHQIATDDVALTGHRFFSITRNFMLAVAGAIVTYEVVLLQFNVALSREESLDA-

>QM_comp15910

MRSYRIEPTSKSSSSAQPTVWTITKRLSPKVNGEYRNAKTAVESPKRGRYETLKMDSIPVDVSRPSASNSGANLLESRRSFHSALRPIIILAQVFGVFPVSGVMSSSASGLKFTWKSLRIVYCILSAIGSINLTIFSIYRLATTSITSNKTSNLVFALTAGVTSLLFLKLARQWPTFAVSWEDMERELAARHNSQRQNSLNLALKFKILSIVVMVLAFVEHALSILSGYFSALECANLRGDDDVWATYFMLQFPGVHIDRHTNTNMFTHKNYAIWKGLIVQFINFLSTFSWNFMDLFLILVSVALTDQFRQLNHRLYSIKGKRCFMVKAMPDWWWAEARIDFNRLATMTRKVDSQISDIVLLSFSTNLYFICIQLLNSFKPMPNAIQTIYFCFSFGFLLSRTSAVSLYAAAVHDESHLPAPVLYSVCTESYSKEVRRFLTQVTTDNISLTGMKFFSITRSLILTIAGTIVTYELVLVQFNAVQSDHQQSSNITNVCEVK-

>QM_comp22611

MHLYFLLVLTTTVNNILDQDKKTRKIINNYLKNANIHQTALETVSRFSMYLLHSKLNITIYNVCSLETSLILKIIGTLTTYLMILLQLQPQDMMQHFDSNKTLPLPANLSTI-

>QM_comp22814

MSFRKFTMKLLTKRVKMRSEILKFN-

Incomplete ORF:

>QM_comp03300

MRNITRNLKLMESLHLKCLFYYFKVCGTAPVNFNIQAINSAKVNRWTFTRNWKGITYNLFLVPCIIVWSYFNMSADYYEPKHLGFDESVYAIVDTFAVFSTTSIILIFCIQNKRLLDLFSKV

>QM_comp23544

MRFDGVDRDKPILGVTENGKLILNYVTALAELGLTVSRREQFV

>QM_comp24536

MLRRTKLWVWLSLALSVAAWTSINQLGMHAFDESYLHNVGYMITYVGTYVATLKLIGLLFLLGQRFAYLNRLLASHSRVAKPGGHGEAFAKKIEKWYNNLLSTSEKLNEIYSCSLFLFLLNLFCHAVSNMYFFTIWTIVDPSYLKNIK

>QM_comp26507

MQSIIEQGTVADTFLQKIETNKDCQKMHKLQLRKVVAQVLIIFTAVSIINFAYIYQKRLTPRGIISMIIFNYPFVLLSIADATFINLVQYSRT

**3. IR protein fasta sequences of *Q. mendeli***

Complete ORF:

>QM_comp03302

MHLLSISKFIIYLINVEALPEIVRIGGLFHSSDSKQEIAFRYAVEKINGNRDILPKSRLSAQIEQINPQDSFHASKRVCHLLKTGIAAIFGPQNAHTASHIQSICDTMEIPHLETRWDFRLKREGCLVNLYPHPATLSKAYVDLVTSLGWKSFTIIYENNEGLVRLQELLKAHGPTEFPITIRQLGEESNIGHGYRPLLKQIKNSAESHIILDCSTDKIYTVLKQAQEIGMMTDYHSYFITSLDLHTVDLNEFKHGGTNITAFRIVNPDKTQDTVRDWIFGEQRYFRKLEMEHNEKNYTFIKTETALMYDAVYLFARALHVLDASQQIEIKTLSCDSADTWDHGYSLINYMKNVEMDGLTGAIKFDNQGFRSDFELDVIELNTKDGLKKIGNWNSTRGINFTRSYGEVYTQIVDSLHNKTFIVTTILSAPYCMWKESSKKLSGNSQFEGYSVDLIQEIARILKFNYTIRLVPDGRYGSYNKELKEWDGMIKELLDQKADLAIADLTITYDREQAVDFTMPFMNLGISILYRKPVKQPPNLFSFLSPLSLDVWIYMATAYLGVSVLLFILARFSPYEWENSHTINNQTSAIENEYTLLNSLWFTIGSLMQQGSDIAPRAISTRMVAGMWWFFTLIMISSYTANLAAFLTVERMDSPIESAEDLAKQTKIKYGALKGGSTAAFFRDSNFSTYQRMWHFMETAKPPNEVFAKSNVEGVERVVKGRGSYAFLMESTSIEYVIERNCELTQIGGLLDSKGYGIAMPPNSPYRTAISGAILKLQEEGKLHVLKTKWWKEKHGGGACRDETSKSSSTASELGLANVGGVFVVLMGGMGVACVIAVCEFLWKSRKIAVEERRQRSSEKPMCIGFTDLR-

>QM_comp09117

MGVVTIFGHVEKITSTYIQNLCDAKEIPFITVQWDHHQERGKSINLHPHTSSLSKVYYIILKEMGWKSFSIVYENADGLIRLSMLLKQWESNNFPIVFYRIKRNVDYRYTLQQVKQSGSRNIIIDCSSNILQDILYQAMQVGVISDQFSVFVTSLDFHTVNFEPLQYSDVNLTGIRIIQSDRLSEMEELLINEDYDFGLSNAQKLATEEALMFDALHLFLKGFQRLKNATDGDPRKILCDTTISWEYGISLNNFIKSVEMDGLTGFLTFDTSGFRSTFELEIFKVGSTGMTKIGKCKSSNDYVDWLLDSSIEMSQNTLSLRNTTFRVLIALTEPYGMLKETVFKMSGNDQYEGFAIDIIYEISKMLGFNYTFSVQEDNVYGSLSPKTGQWNGMLRKIIDNDADLAITDLTITAERETAVDFTMPFMNLGVSILYQKPKAAPPSLMSFLLPFSANVWLNLISAICIVSALFFIIGRMCPDEWNNIYPCIEEPENLENQFTMKNSLWFCLGAIMQQGSEIAPIGNSTRMLAGCWWFFCLIMVSSYTANLAAFLTVTTVERPIKNAEDLASQDFIKYGAKKGGSTLGFFRDSNSSTYQKMYQYMMDNSKDVLTSDNDEGKKKVLSGNYAFLMESSSIEYIQERECNLTQIGGLLDQKGYGIAMRKNASYRNELSGAVLKLQEMGILTSLKNKWWKEKGGGGRCQEDSSGGQAEELGMANVGGVFLVLVVGIVLSFFMTVMEFLYDLIATSQHEKIHLKRNLSYELKFILNFGTSTKPTKRCSS-

>QM_comp14953

MKGEQPGLLLAAPPLLLLLILQLTSNGTVHALPPLIKIGAIFTLDQANSSTELAFKYAVHKINKDRLILPDTTLVYDIQYVPKDDSFHASKKACQQVKFGVQAIFGPSDPILGQHIHSICDALDIPHLEARLDLDSEAKEFSINLHPAQSLLNNAYQDVMAYLNWTRVAIVYEEDYGLVKLRELVRSRRSQDMEIYLRQADLDSYRQVLSVIKAKEIRNLIVDTKPGNMHHFLRMILQLQMNDYNYHYLFTTFDIETFDLEDFKYNFVNITAFRLVDADDAEVRGVLRDMEKFQTHGGSAGLLNKSRVIQAEAALMYDSVQVFAVGLRTLEQSHALRPMNISCELEHPWDGGLSLINYINTVDRKGISGPIGFKEGRRIQFKLDLLKLRQQSLVKVGEWKPGTGVNITDKSAFFEPGATNVTLVVITILETPYVMLRSKGNFSGNDRYEGFCIDLLKEIAHMVGFAYRIELVPDGKYGVYDYETGEWNGIVRQLMDKKADLAVGSMTINYARESVIDFTKPFMNLGISILFKVPTSHPARLFSFMNPLAIEIWLYVLAAYILVSVTIFVVARFSPYEWNNPHPCHSQNSDIVENQFSLANSFWFTIGTLMQQGSDLNPKATSTRIVSGVWWFFTLIIISSYTANLAAFLTVERMITPIENAEDLASQTDISYGTLESGSTMTFFRDSMIETYKKMWRFMENKKPSVFVPTYEEGIQRVLQGNYAFLMESTMLDYIVQRDCNLTQIGGLLDTKGYGIATPMGSPWRDKISLAILELQEKGEIQILYDKWWKSPSDTCMRNDKDKGSKANALGVDNIGGIFVVLLCGLTFAVLIAIFEFCYNSKRSVPVERQSSPLPPATCSGSLQGLPESAMSSIGQQQESLCSEMARELCRAIRCHASSRRRKACEKCSSHIVGYPIDNTMPTPINGRNSLGLEMSPHIHRHHHSPVHHHDFDGNL-

>QM_comp15705

MALLFAVIVIMVTTAMSLPPVIRIGAIFTEDQKDSPSELAFKYAVYKINKEKVLLPNTTMVYDIQYVPKDDSFRTSKKACKQMSRSVQGIFGPADPLLGAHVQSICEALDVPHLEARVDFEPSFKEFSINLYPAQDHLNRAFRDLMSFLNWTKVAIIYEEDYGLFKLQDLVKAPPSPKTEMYIRQAGPGSYRQVLREVRHKEIYKLIIDTDPVYMQQFFRAILQLQMNDYRYHYMFTSFDIETFDLEDFKYNSVNMTAFRLVDLDEPTVADTLKHMERFQPIGHTILNRSGIIQAEPALVYDSVQVFAHGLAALDRSHVLRPANLSCEREEPWDDGLSLYNYINSASLQGLTGHIEFNEGKRTNFKLDLLKLKKEELIKVGEWKLGSGVNISDVAAFYESSATNITLVVMTREEKPYVMVKEDKNLTGNARFEGFCIDLLKWIAGQVGFQYAIRLVPDHMYGVYDPDTKEWNGIVRELMEKRADLAVASMTINYARESVIDFTKPFMNLGIGILFKVPSSQPTRLFSFMNPLAVEIWLYVLAAYMLVSFTLFVMARFSPYEWNNPHPCLGETNVVENQFTISNSFWFITGTFLRQGSGLNPKATSTRIVGGIWWFFTLIIISSYTANLAAFLTVERMITPIENAADLADQTDIPYGTLEGGSTMTFFRDSKIAIYQKMWRFMESKQPSVFVSSYEEGIKRVLEGNYAFLMESTMLDYAVQRDCNLTQIGGLLDSKGYGIATPKGSPWRDKISLAILELQEKGVIQILYDKWWKNTGDVCNRDEKSKESKANALGVENIGGVFVVLLCGLALAILVAILEFCWNSKKNAQSDRSLCAEMASELRFAIQCGSRQRKLRNAASSSSSALGGPGGASCNRCNTRSSMRRSIRYRDPSQRRETSRERRARELLQHQQQHHQQPLPPSSSSLHEETTYVPNIDYPWLNAGGWCGGDDGDEEIIELRASNTRRVQLSERSIGCSRREHHLASAADLPAASSSAGSSNGGGGGGGGGSGSGNGSEQQQQQQQQQQQQQQQQQQLQQRAHSIPASHALPTSSLALIPHSRSFAQTTPDSGDEAETSLFRHRLGSRR-

>QM_comp17958

MSMSYLVLGSIVLHLVLAYDGVIIPKAVIGGFFDRNASMRSAFSVAVRTINECNYDNEICEQMHSTENGINPRLYHQVEFSAMAVEIDIDDVAALYKKFGELSGTIRNSFDDKFSPTNEYGLSTVIGPHDRLSSSFTQGLCDYHDIPHIIIRRDLEFQHNPVQTINLYPDTNTLTTVYIQMIKKMNWGSFSILYEHSENLIAIRNVLKMYKFYTYNIYTFRLGNGPNYSEPLLKAKESQHKCLLLDCHSNVLLNVLRQAQELGMMSESYKYFVTSLDLQATDLWPFRYTGANISGLRLVDPENSFVSKFVGSHLDDFGITSVDKLLVNDALMFDAVALFAQGYKDFVYKNPIRFGARLTHLDDGSSHPWIHGLSLRNYILYNHLDGLTGPVIMDPDGVRRRFKLDVINLKQTGLTKVGSWDPEQGFDELELTNEISVEHFKVLITMNAPYAQKVKHSRSMEGNARYEGFVVDIIKTLAEDLHFNYTFYVQEDSDNGNCTKDETTEQCTCTGMMGKILRNEMDMAITDLTITENRAKCIQFSTAFWNLGMSILYKKPQKAEATWYSFFLPFDRTVWMNLGLSWIIVSFLFFIFGRLSPPEWNNPYPCIEEPDELVNQFTINNAFWFTIGALMQQGSEIAPIGGSTRILAGSWWFFCLIVTNTYIANLAAFLTIETPVTSVRGIEDLYNQTAIKYGAKKGGSTFTYFKSSSDPKHKELYNNMIDPEWQKKYMVDTNEKGIELARRTDMNYAFFMESSSIEYVQHRKCDLEQAGGLIDQKAYAIGYAKNFTYAKEVNKLISKLNENSVIKDLYKKWWTEKGAVCGDDNGNMPQPMNLSQLIGVFYVLTGGVFVSLMVTLYEFVRDVQAKAKIEKASTMKTLVEELINVAGSKSVKPVLRKQRSSDTNYSDNHSCNLTMTTQA-

>QM_comp17959

MSMSYLILASILHLVLAYDDVRAPKAVIGGFFDRNASMRNAFNLAIRTINECSYDHEICEQMHSTEDDVNPSVYHQVEFTTIAVEIDDDDLNALHQKFGKMSGAGGDFHHDKLSSSRNNDYGISTVIGPHDHLSSIFMQGVCEFYDIPHIIIRPDVDLQHKPVQTINLYPNTKTLANVYIEMIKRINWGSFSILYEHTDNLLAIRNVLKMYEFYTHNIYSFRLGNGPNYSEPLLRAKYSLHKSFLLDCHTDVLFNVLKQAQELGMMSESYRFLVTSLDLQTIDLWPFQYTGANISGLRLVDPSNEFVSKFVGSHLDDFGITSVDKLVANDALMFDAVALFAQGYKDFVYKNPIRFGAKLTHLDRGSSPPWSHGLSLKNYILYNHLEGLTGPVILDPDGARRRFKLDVLNLQQTGLAVVGNWDPDKGFNELKLSTQMSMEHFKVLITMNPPYGQKVNQSHRMTGNDRYEGFVVDIIKVLAKELHFNYTLYVQEDSDNGNCTKDKNTDYCTCTGMMGKILRNEMDIAITDLTITENRAQCIQFSTVFWNLGMSILYKKPQKAEPTWYSFFLPFNYRVWIYLGLVWMMMSVLFFIFGRLSSPEWTNPYPCIDEPDELECQFTIDNSFWFTIGTLLQQGTELAPIGGSTRILAGSWWFFCLIITNTYTANLAAFLTVETPLKTVKGIEDLYNQTAIKFGAKKGGSTFTYFQSSSNPKHRELYNTMIDPEWMKNYIVKDNDMGIEMARRTDINYAFFMESSSIEYVQHRKCDLEQAGGLIDQKAYAIGYATNFTYVKEVNKMITQLQENLVIKDLYKKWWTEKGAVTCETGSNAPQPMQLDQLIGIFYVLTGGIIISLIVTLYEFVRDVQAKAKIEKASLMKTLIEELINVAGSESVKPVLKKQRSSDRNSSTSHSCDLTMTTQT->QM_comp19239

MLCCLASLRRNYTEVPYINCQLVCRDSEFSSSSCKGCIIN-

>QM_comp19240

MSSRWLLLLVILSLGSHLEVCGDDAGFWTGGGGSNDKGGVQRTSSTIKIGKSDRSSGSQQSHGRGSSMIKIGEGSKLLNGSARVVRPALTTSMTSTSIPLSYASVQPDSQGSLNEYTNGTTKMLKVGLAVPYKSFGYREYTKAVSRVVTALQKSTKRPNLGLFQHYDIFVKVAMQELTPSPMNILNSLCKEFLSLNVSAILYLMNYEQYGRSTASTQYFLQLAGYLGIPVIAWNADNSGLERRTSQSSLHLQLAPSIEHQAAAMLSILERYKWHQFSVVTSQIAGHDDFVQAVRERISEMQERFKFTMLNAVTVTKSEDLKDLVSVESRVMLLYSSKEEANNIFRHANEFKITGENYVWVVTQSVIQNIQPGHQFPVGMIGVHFDTSSTSIVNEIATAIKVYAYGVEDFVNDRSNSGYSLNTDLSCEDQSSESRWSTGEHFFKYLKNVSVEAEYGKPPVEFTQDGVLKSAELKIMNLRPGPSMQLVWEEIGTWKSWEKDGLDIKDIVWPGNMHTPPPGVPEKFHVKITFLEEPPYINLAPPDPVTGKCLVERGVHCRVAKDPDPEAEIQATGGARNGTAAAFQCCSGFCIDLLQKFSEEMGFTYELLRVEDGKWGTLENGKWNGLMAELVNRKTDMVMTSLKINSEREAVVDFTVPFMESGSAIVVAKRTGIISPTAFLEPFDTASWMLVGFVAIHSATFMIFLFEWLSPSSFGIPDYTKPKPQQHRQHRHHHHHHHHHHHHRHYRHERERHQQNHHHHHHHRPRHHRFSLCRVYWLVWAVLFQAAVHVDSPRGFTARFMTNVWAMFAVVFLAIYTANLAAFMITREEFFDFTGVDDHRLARPMSHKPPIKFATLPFTHTDSVLSKYFKEMYAYMKNYNKNSVSEGIDAVINGDLDAFIYDGTVLDYLVSQDQDCRLLTVGSWYAMTGYGLAFPRNSRFLKMFNQKLLEYRDNGDLERLRRFWMTGTCRPDKEVQKSSDPLALEQFLSAFLMLMVGILIAAILLLLEHIYSKYIRRRLAKDSRAKRYLPTPLSGLSLQREMMNPEIDLET-

Incomplete ORF:

>QM_comp00408

MDKELVQAVECGLIKQIKSKNLTEAVICPLNLKSKERRLKVSDLWMTYKVVFSGMGVGGIVFLLELISKLIQMCLNSEWYRTCCKTRIRGSSPPDVNSAWITDKPETNVWARTPPPVYQFSEEWTHKTYSINGRDYYIIIEEGGDQRLVP

>QM_comp00663

MAPFTGETWACLSAALLLVGPFLYFMIKSSPVPISPYDTSGLKTTWQCSWYVYGALLQQGGMSLPKADSARLVVGTWWIVVMVVVATYSGNLIAFL

>QM_comp02441

MKEEVCCHLLYQAVPCSCPPDFRIFPHYFRRLLHLIVETLSELQSANQLQEPQTGQCQNRNASGTNSIESSGGASSNGNGSTSEFDRRLLAKLAKNDTFE

>QM_comp04378

MVHPGSQWLYVISDGASHDANVTMFLDLLSEGENVAFVYNSTSLGPECHMGLTCHIKEFVRTLAISLESSLKTELELYDTVTEEEFEVVRLSKA

>QM_comp05929

TTTAATCGCTACAAAAGAATTAAAAACTTATCGTATTTACAACTATAATTTCAAATAATGATTGAAAATGGTCGATAATCATATTTTCATGACGTTGTTTTGAAAGAATCTTGTTCTTGGTGATATTGATGGAAAAATTAATCACAGGAAGTTAACTAATTACAATTTCAGAGTTCTCCATCTTAACGAATGCACCATGGAACGTGTAATGATAAGGACATCAAGCCTACTGATTGTTTTGTTATTTATTCCGACCTGTCTAGCTGCTATTCAAAATATAAACATAGGTGCCATTTTTCATCCAGGCGATGGAAAACTAGAAGAGTCTTTTGATGCGGCAATAAAAGATTTAAATGATGAATTGAATAGCCATTTCAACTTCATAGCTGTTAAAAAAACGAATGTTGAACTGGATAGTTTCAAAACTGCCTCTGTTGTTTGTGAACTACTAGAAATTGGAGTGGCAGCGATTTTCGGACCAAAACAATATCCAACACGCGATGTAGTTGCCAGCATAACGAATCGCTTCAATATTCCGCATATAGAATATAGTTACCGTCAGGCAGATTATCAAGAAGATGGACCAGCTAGTATAAATGTCTATCCAAGCAGTAGAATCTACGAAAAAGTTATTGCTGAAACTGTAAAACGCATGAATTGGCATTCATATACTTTTGTATATCAGACGACCGATGCTTTATCACGTGTTCAAGAAGCGTTACAGAATCAAGGAGTATTAAATTGCCCAATCACGGTCCGAGAATTACAACCATTGCATCAAATCCGAATCTTAAAAACGAAAAAAGAGCAAGATAATGTTTATGAAAGCTTAGTAAAGGAAATACGATTGTCATCGCAGTTCAGTTTATTACTCGATATTGAAAATGATAATATATCTGAGTTATTCGAAATAATGAAGAAAATGGAATTAATGAGTGACTATTTCATGTGTTTTCTTATGAACTTGGATGTGCCCGTGCTG

>QM_comp05930

MNLDVPVLTMKKYISKNNVANITWLQFMTSDYSNETVEAALLYDAVFALSGAVNNVHKDGNSQNDYNKIIPKPLSCSGEAKYEGGLNITKAFYEKTRTGKKTGMMTFDENNQRQFYLKVMNIQAGRETVVGEWNGNQLVINRNEKEINNTLTEAAQNKIFRVTTRVGAPYIMLADGDAKGRQIGEKKYQGYCIDLISKIEEFLKIKCEFDIVPDGEYGALNSQTHQWNGLVKQLLELKADFAICDLTITSERQSAVDFTAPFMNLGISILFSKPSKVIPKLFAFMDPLSTEVWMYMATAYLIVSLMLFFQARIAPGEWVNPHPCNPDPEELENNFTLMNSMWLTMGSLMQQGSDILPRTPSIRMVAGIWWFFVLIMVSSYTANLAAFLTAVKMEDSINDVEDLAKQTKISYGAVKGGSTYSFFKNSNTSLYQRIFNSMTDTKPSVFTSNNDEGVDRVKNGKRKYAFFMESTTIEYQIERNCELQMVGTLLDNKGYGIAMPPNSPYRTMISTAILHLQEKGDLQQLKQKWWKEMGGGRCNDDSDEPANSNELGMPHVGGVFLVLMLGCIISTLIAIIEFLWNIRKVAIEEKTTLLEAFVKEIKFVINIWAISKPIKGEKSSKSPSSQNGSCSKSDSSNVSPITSLRRIDIRNETSVD

>QM_comp21031

MFVAPNKWLLISHECSSIDCVLLKLTDLQTSLLPDSEVLVWLSQLKRVISVYKVSMESGRDWQIEERAHILPNGTLIPEHNSLNVTSRRRSNLQKAHLKSSLVIRDFDSLNHLDEYVKSDPDPSSKCVYPWAKFLA

>QM_comp21678

MPKLPLLLPLLLLASGVSGQAPVTLLLVIEPPDAEILSNLNDVVAEAESQFGANLVKMDVKVVQVDREFVDENYDRVCAQLYK

>QM_comp26138

MTKPRLGDETLVVIGAISQQGFWYEAQATSTRLLVLFGLLGFLSLHAAYAANIVALLQSTSNSIKSLKDLLASPLDLGVHDTIYNRYYFKTLSILEPKVRGAIAEKSEWMD

>QM_comp26139

MDLDEGVKRLRKGHFALHCDVSYAYKLVSDIFEEDEKCDFEEIDFMNVLELHIGVTKRSPYKEFLRVKALRVRETGMHTRQTKKFYFRRPRCDAAASSRRFISVGFTECKGAFYALG

>QM_comp26609

MRLDLMDTGVCNFHVGTEDFLHENMGLLVAADSPYLEIINNAIIRMHESGLINKWSLDVLPLRDKCFVTNAAQEVTNHKVDMGDMQGIFFVLAIGFTLALILIGSEFFWHRRKILAERNL

>QM_comp12607

MDFDFDLVVPEDGSFGQKVNGVWDGLVGDLARGQTDIAVGALTMTSEREEVIDFVAPYFEQSGILIVMRKPVRKASLFKFMTVLRLEVWLSIVGALTLTGIMIWLLDKYSPYSARNNKHMYPYPCREFTLKESFWFALTSFTPQGGGEAPKALSSRTLVAAYWLFVVLMLATFTANLAAFLTVERMQSPVQSLEQLARQSRINYTVLANSSIHQYFLNMKMAEEKLYQVWKEITLNSTSDQVEYRVWDYPIKEQYGHILQAITQVGPVSSTEEGFQKVEDSENAEFAFIHDSSEIKYKVTQNCNLTEVGEVFAEQPYAIAVQQGSHLQEEISRKILDLQKDRHFETLSSKYWNQSLKGNCANADDNEGITLESLGGVFIATLFGLALAMLTLAGEVIYYRRRNARNPGQAGGQSSSSPLSGRDDQLNKGSVGKDNEQIIIQKLAAKLQLKPAPPVAFGEQKPTNPKPRVSHISVYPRPFPFKD

>QM_comp21661

MTVEQLKGIFYVLGVGLVLSCFQTAFEFVQNIRSIARKTKVSFCKAAKIELKIATELKPVK

**4. OBP protein fasta sequences of *Q. mendeli***

Complete ORF:

>QM_comp02170

MKGYLSIIAISLALVLVKCEPPPEMKEMLEECEKEVGVTKDLVHGPEHSDDPKVKCFHACMMKKKGEMVDGKINEEKAIEAMSKHGHGEPNKEVVTECATKANEEKDECEIAGAFHKCMMEKHPHKKE

>QM_comp02388

MLAIGSTLVCISRGKTIQSRCTYQHTTRVHFTVMTLAPTPSSTSLKKHAKVSFKNQSSPNEFTHPRVPILYCSALEVLMVCGLHQENISSFDFHVSTTRVVSVHHGQLGEKLKCYPTKVHFHRG

>QM_comp02394

MKAYLCILSLLSALVLVQCGVNPQMAAVINECKEEFGLTEESLSSINFEDPKIQCFAACVVEKEDRLTDGKLSIDKEVEAASRYMPDKIDETLRDKMTECVNEANEEKGECAVSGTLFKCIAEKFGIHP

>QM_comp02616

MLSISDTFIPLCAFLISTYNMKCFVIVLAVCVAGAYAATLNDEQKATLKGFKEGCITESGVSADTVNAIIKGGEIKREEKLDCFSACMLKKIGIMRADGTIDVEASRAKVKTTNVDVAKADKIIDKCKDLIGKDTCETGGNVFACFVQGKDFPVLD

>QM_comp03741

MKIYINVAVTLLALVLVKCEIPEKVLKILEECEREVETLNVTPNTHEQKAKCFHACTSKKHGFMVNEKMIIDKAIDVATTYESKEVGDIIHTAITECVNKANEKTEECEVAGVYYKCYMEKVTSHYHS

>QM_comp04039

MKCLLAFCITICVVAVKSAEQDVSKLGKGKEKPKQLNEECAEESGFNLKAMYHEIATNKASQFTPTQPIRCFVFCMYQKIGLMNEDTSLTQKFYDTEGAHKCTHIVGNDACDTAYKVMECVTKHEIWFKVDY

>QM_comp05191

MKLLLCFCAVLCSISAFCKAASPADIEDCAIEHDVDLRYLHESNTDKKFVPTREMMCFAACAFKKDGILSDDGKLTDKAKDSLVVKSCKITDGKDACETAGMMMDCVSNNPW

>QM_comp05917

MSISFYILGILLTLTLITAEPPAEIQRQNDECEKEMGITKEWLREHPNDHQAKCFNACRMKKDGSIANGQVITERAIEVASKYGPGGANDADKVEIRKCANKANEEKDECEVAAVFHKCFMENIYAHKFDSK

>QM_comp06765

MKAYVGTAILLIIIGMEFKSIECGKKLDIEGMKDFLKPMSKSCKTKTGVSSELIAGTAVGQWPKERPLMCYFKCLATMLKIVNKQGEVSLREVNKNLDTLVAEDLAPKMKDIINTCLDTVERIADICEYIFDVIHCGYQKDPSLYFLPN

>QM_comp07285

MPIVCLQTRPSFVSDKMIATAASVVNACQTQTGVTTGKSSATPHLHLQPFSPLCRSNYYIQAREPRVCAEFAAPPNFSLAQSPPNITWLFI

>QM_comp08027

MKSFGCIFVVLVVAVCIYGDSSEEHREMRREKFEKCKKEVGMSEDRPHGPPNFDDPKEKCFTACLMKEDEKLVDGKIVIDKVLSFHKKRMPDFNDDIQEKLTYCVETANEKSDECDVAATMMKCTFETLGPPPHRRHHQ

>QM_comp08037

MKVYLCILGILSAVVLVQCEIDPKLKAIVDECKKEFELSEEALKSPDFEDSKVHCFAACVLEKDGRLTEGKVSIDKEVEAASRYIPERIDETLKDKMTECVNKANEETGDCAVSGALFKCIVDTFGTLHPETS

>QM_comp08573

MKTSFVFLLTLAAVITESLQADDAVLQKKIQECFAESKIPDDLQKTAITAQLDNPKLKCFNACMMKKKGTMKNGKINPETQLAEAQPESYDIIKEVVRTCSDIANRQSDECEVSYSYVKCLINNAGAYQGLQ

>QM_comp08613

MKLLVSFCLVICAAGIFCNAVIDENTLKCAEEKKVDLKSMEALMKQDNFTPTQEIKCFAACILKKEGLLNGDGTLTQKAIDHPVVKNCTITDVQDACETAGKWMECIAAIPW

>QM_comp08638

MKAVLALFAVVCVVGVQSHAGMAHNLTPEQIARITAEVEECARVNHIGHEVFEDMKAGKAPTPSRDLDCFSACVLKKNGVMNADGSTNHKPNESEAGKACKDLKGTDDCDTAGKIMSCLHKNDLIPKIK

>QM_comp08676

MKSIVVVLAFCVVGALAGITDEQKAKLKEYRTACVTETGVSEDAIDKAKKGEVDIHDEKLNCFAACMQKKVGIMRADGTLDEQVARSKLPKDLPQDKVDAVINMCKAQVGKDACETGGKVMACLAKTEAINLLN

>QM_comp08846

MKLLLSFCVVICATNFFCNAMVPTEIKECAYKYNADLDVWRKIDILDASFTPTEQMKNFSTCVYKKYGIMKEDGTLSKEFSEGPLITICKTTAGKDESENTVKLMKCFAEHGDHLLKNVKLPSK

>QM_comp08899

MKLLLVIFAAICVIGVYSSESFLDKIHPQVEECLTELNVDRQKYLDMVKNPNFIRDRDTDCLSACLLRKRNLMDANGKFTADFETRGIDKMCKAEYGKDECETASTLITCLRKMAESIGNVNTEISS

>QM_comp08900

MKLPLVIFAAICVIGVYTNENFPEKLNPQVEECLIEKKVDRQKYIDMITNPNFVRDREMDCLSACVMKKRNLMDANGKFTADFETGGLGQICKAEYGKDECETASKLITCVLKKEENDKDNVNTEKSS

>QM_comp09338

MKTYCCVAAFLCAFVLVQCEIDPELKSKIDECLNEFELSEESLKTPNFEDDKFKCFAACTMKKNDCLIDGKASVEKEIALASKYMADKIDDTLEAKMTECVDKANEEEDDCAVAGALFKCVTETFGITYP

>QM_comp09339

MKTYCCVAVFLCAFVLVQSEIDPELKSKVDECLKEFELSEESLKTPDFEDAKVKCFAACTMKKDDRLTDGKASVDKEVALAPKYMADKIDDSLTDKMTECVDKANEEEDDCAVAGALYKCVTEKFGPITP

>QM_comp09356

MKVLSRVAVFALILLVFDDVNAKMTMDQIKNTLKPFKASCLKKTGIDPALVEGTKSGNFPEDRALMCFVRCVFQMMKIAKNGEISLVAMNQQVDLMMPEEYVDEMRAIIDNCGALANAKEDSCEGPFTFAKCFYDSNSELYFFP

>QM_comp09551

MTLLRSFCLIICALSVFCNAAKEADLQKCAEENNVDLKSLDEKNATPTREVKCMFACVFKKDGILNNDGTLSDKAKNGLSIKNCNVTDEKDACETAGKLMKTCPDVI

>QM_comp10209

MKVLFVTLLFCIASTLAELTEEQKEMIKPYKDACMAEMKIDQAAIDMARKTFKEEGKFEVGQMGCFSACMFKKIGFMNEEGKFDEEMVRALTDGQFPEAQMDAAIGACKDEAGKDDCETAGKLVLCFLKQGISVSP

>QM_comp10426

MRICLCIFTIFSVLVLMKCDDQMFEECRKETGITEEKLKVLSSSKEALCLSACMMKKKGVIVNGKAVMDKVIEDISKDMPENTIDAIKIFTPACVNKANKKKDECMVFMVLRDCLIKKRLISK

>QM_comp10855

MFFDWPIIFPSRALIALEGMQELQNYEKQCVRLTGVSADHLQMAWLDRKIYEDEAQEKFAVCMLQKFRIMNMDGSINTDSVSYFLLTDNPASFETARDCAALAGSNVRETARKIMTCLLQTNIIVMNPPLPAQEFWLSNISSI

>QM_comp11668

MKLSLALFLIICSVGVYCIDVSKIAENVESVNPNIKYCAAENKIDEKTFEEIQKNLAFPKTREMQCFMACVMEKDGMMNSDGTFTKKFTESKAAEVCKNLGGKNKCEIGFNTVDCLIKNNFPFNH

>QM_comp12532

MKFFVVFAFCVAAAAADGITEEQKAKLREYKVACIAETGVSEDVVGKLKAGEPVVFDEKLNCFSACMLKKTGIMKPDGSIDEQVARAKVPKDLPQDKVDQVINSCKVQVGKDACETGGKVLGCLAKTKAVNLLN

>QM_comp12533

MKFFVVFAFCVAAAAAQGITEEQKAKLREYKVACVAETGVSEDVVGKLKDGESVVFDEKLNCFSACMLKKTGIMKPDGSIDEQVARAKVPKDLPQDKVDAVINSCKVQVGKDACETGGKVLGCLAKTKAVNLLH

>QM_comp14843

MKFLILCTIICIFAIPSYGAEDDAKSEKDSTRLPTEIEECAREQGLTNTQDVEETIKNLDYNNLTREMQCFTACIFKKQGLIGADGTAHKNFQRPEIAKHCKDLSGKDECEVAFKIVTCMNRPEIFTDA

>QM_comp18897

MMKCAFALLCAFVVLGCAYSADLPFISQMKECGSEMGLSADQVMEMVAKNDPQLDCVRACVLERLGALNNGQLDKGALISILDQNKDTLPNYDQLRGNLDMCYGEATTAGLTDRCQLGGKFSSCMQSQMTG

>QM_comp20903

MKFCIGFCAVFCIAGVSCNGQDGYPKKPPQDLINRCALRHDINDAIMNEIILTKNFSKTHDTQCFAACLMDESGIVKSDGNICEYFQISAPGIQCNNWSGDDMCEISANVLSCLFQKNFMTLQGLLPDL

>QM_comp21133

MYCCVAACAQSEPNLQRKLHLREDQCVGDHDVLYTIERIDRLRSTRHLPEDGSLDEFAACMLKNYQIFKPDGTINTDVKSHTLLISEERQAEPLSKHCIARIGSDNKDTLRKI

FECYLRSGIYIIDFAPRTRTMKPRSLF

>QM_comp21238

MVAKIRIIIEGICEATDMDITIMLVVDMDMEIMDIRIRIQTDTTTTIIIIIMAMDTTIGIITTTTTTMTCTIMTESASKHASPSAFSMNSIWLIKEVFRKGRQ

>QM_comp21830

MKMDRHLCCALVICLQALVFVSAGPPDWISPEILEMVQSDKARCMGEHGTTEALIEEVNQGHLPNDRAITCYMYCLFEAFSLVDEDGELEVEMLVGFLPEHMQEVANELIEACAKESGSDVCEKMYAVAKCVQQRRPDLWFMI

>QM_comp23536

MKIYLCITYILSVFVLIQCAIEPALRAKFEECAKELGLTSEPKTPSSIGPKFKCFIACAMRKTDRLLEGELSI

>QM_comp24139

MKIYVALFLLIALPAAFASFSPDIEIFLLKLEEDCSKESGLGSAEVEEIRKSRVVPDNEIANDWAKCMMQKHNVIKSDGTVNMDVRSINIPADTDTVLRAFPKCSVLTGRDIADTARLMMNCYRSNSEGLIMGFSKRQTAA

>QM_comp24881

MKILLAFIAICIVVVNCDRPLMATNSDMETCAVRNKIDKVTVTELKRNRNFTATPDTQCFAGCVLREVGIMLDDGTVVREFIDSELASRCNNYFFAFGKDNCEIAAKMLGCLTQKRVVILPEE

>QM_comp24882

MKILLAFIAICIVVVNCVSNNNIEFEDRPLMATNSDMETCAVRNKIDKVTVTELKRNRNFTATPDTQCFAGCVLREVGIMLDDGTVVREFIDSELASRCNNYFFAFGKDNCEIAAKMLGCLTQKRVVILPEE

>QM_comp26560

MKNHAIIGILILSVHLINEAYAGATREQMEKMSEGFRKTCIGKTGAEFSIVAGIRDGNFIEDPTAKCYTKCIMALMKTLTKQGQIDADMMIKQINIMGSPDIAGPMTAAVRKCYAEVSADEPCELAWLFTKCIHAANPSVFFFP

>QM_comp26834

MKAFAVVLAVCLAVACADDPLKDVPKDLIKTCLTENGFDAAQYPNGLRNVKVTDNQEKNRDCYYACMMKKMNLMKPDGMLIEDNLKSKFNLNLETLQKALNTCKAQVKGNDSCKLAACLMANRGI

>QM_comp03957

MKLIILAFCLLFVAIQSIRGEDESMQSLKECLDENDIKDDFTKLKENDDPKLRCIMACVMEKEGVLKNGDIKLGQLKKDLLEDAEQLGEKKVSEIVDMCGNIAKDLTDVCDKANLIGMCIKEEFEKHNIKF

>QM_comp06538

MKTVAVCLLVVVLGVAKIHAANEVPKEIQEMIAGVREKCHRETGVDIEHVDRTVEGYFHPTETLGCYFSCVFGHFELLDHNGHLDFDKIIPKIPESFKDHGMEMINACRHLTGKNPCDMAFNVVQCFQKTNPEKFFVI

>QM_comp06539

MKTVAVCLLVVVLGVAKIRAANEIPKEIQEMVAGVREKCHRETGVDIEHIDRTVEGYFHPTETLGCYFSCIFGHFDLLDHDGHLDFDKLIPKIPDSFKDHGMEMINACRHLTGKNPCDAAFNIVQCFQKTNPEKFFVI

>QM_comp10678

MHSFTVILVFYALFARYAHADSYNVELTEEDREIFKKCIKEGGLTKEELNAAVRNFDKDADRKVKCFRGCLLRNHKVIKDDNTIDGRAAVRYYHVEDVEPLKKLILKCSASTSGTDYCDVAQTVESCFRASDIEN

>QM_comp18896

MMKCAFALLCAFVVLGCAYSADLPFISQMKECGSEMGLSADQVMEMVAKNDPQLDCVRACVLERLGALNNGQLDKGALISILDQNKDTLPNYDQLRGNLDMCYGEATTAGLTDRCQLGGKFSSCMQSQMTGVAADMLMKGASQIGNMANKFH

Incomplete ORF:

>QM_comp01352

MKVLIIFCLVFNVICVYSDNAEDKHNVEVDKEKINAQNEECISEHGIDLKTMFHEIQTNKDFEPTRPMLCFAACLLKKNGLMNPNGSLTRKYNDSAGGQECKNITGRDECEAAFKIMNCLKKHEFLLTGED

>QM_comp02233

MKMKIYFFAAAILCSLVLVKCDEMEDFEKYNNECQTERSEAVVAGLAKPAASYDDPEWWCPGACMARKFGTIVNGQVIAERMVANILKKKEGGAKDVIKADAEGCIDKANIEKDECAVFMTLQNCLSEK

>QM_comp02693

MRAYLGILGIFLAMVLVNCELPEELKKFFEECGKEIGETPKGPPNFDDPKMVCFAACLMKKGGSLVDGKIDAEKAFEFGTKHLMEKVPDNAKDSITECATKANEAKDECEVSGAFHKC

>QM_comp04767

MPSNLRIHAAKHFKLLFSVRVAFLFFVNSSNDFRSTSVSAA

>QM_comp06244

MKAFVMFFAVCCFGVYARNITLTNDELDENIKMCLAKTRLSQAFFKSGDETLKYLTEEQKSCFLACMFKKAGIIEHDGSVRSVTDDMDVATANAIDRCQSLADGNICKLAWCLHKTEKFGIPVISD

>QM_comp21371

MRIYLCIFIIFSVSLLVKCGNQMVEECRRETGMIEESLVSDSNDPKAWCVSACVLKKKGIMVNGKAIADKVIEDLTQDMPEIAKDAVKTGSPACVNKANQ

>QM_comp21900

MVDGKIVIEKAVEVASKNHGNSDSLKEDITECANKANEAQE

>QM_comp23080

MKIYLCITYILSVFVLIQCAIEPALRAKFEECAKELGLTSEPKTPSSIGPKFKCFIACAMRKTDRLLEGELSI

>QM_comp23819

MKVFSRIAVFALVLLFFEDVNARMTLDQLKNTLKPYKTSCQKKTGIDPALLEGTKSGNFPEDRNLMCFYRCLFQMMKIARDGEILIEHTAQIMSMLMPEEHVEEMIAIIKNCGVIGNAKE

>QM_comp04156

MNTLTVFLLLCAIVAINVQANIPKSKFNPELKNVWDKCTKEAKLSEAELDIVLENYSKIADQRVKCLKGCIFKSLHVINADNKIDEAGAQKYYEGVATKKDTINEAIEKCMPRGTGNDQCDIAHNFESCLCSEYIKCS

**5. OR protein fasta sequences of *Q. mendeli***

**Complete ORF:**

>QM_comp01268

RLKKGSALFDPHENSKNVPKDIEDKVRPIISEAILLHNEAFEMINLMEDTNNVGMVGILFQNMILFGACPFLLSVHLQRPIEFSRYVLMFILITLHFWVIFTHGQSIIDHSEAIFDTCYEFKWYYLSKRTSNVINIMMTRSVKPSEVTGGKIFVLSMETYGRMVKSGLSLFTIFKSLGVA-

>QM_comp03785

MRKAAEMASKSVLREWQREELVYKNMRAAILLHKEAIQFVGK

>QM_comp05484

MYRYSTAFMIIFIHMYYVYLPGQKIINYSLLISDDW-

>QM_comp05486

MYRYSTAFMIIFIHMYYVYLPGQKIINYSLLISDDCYSCVWYQMSARCKYLLKFMMLRSTRPLELTGGKMFVLCMATYAGMLKAGMSYFTMLANTK-

>QM_comp05500

MKNFGMTIVMMNFAIKLVAFRLSHRVVKYLVNGIIDDCTIVDKSGDNKVMARNIKTSHFVYKLIAVCFNAMIISSIVTSVASYNKTKSIEHTEYLIPVTYPYIAKNWVNYILVNVFSLLQFFACANLQAIIDGFLVLMVFHAATQVHNIRSALKRYSDACCTTTDRAKINIALRDFLQLHQKYLDFAANVEKAFTYVILSQLVAVTLIMVAVEYTVLTMIDDNAMTADIYFYLNFLIGDLFISATYCAAGEYLIVQSDYIHRDLCQCSWYKFHIKDMKTMLILLPKLQRPPFMTYGKLGKLSLFCFTSILNTSFAYLSVFRAIKTRQNYDMN-

>QM_comp14333

MMKMKQVGLVADLMPNIRITQAVGHWLFNYYSEGMRFPHKIYCMVTLFLMLFQYGTMAINLVKEADDVDQLTANTITVLFFMHPIVKVIYLAARAKIFYKCLGIWNNPNSHPLFAESNQRYHTLAITKMRRLLFSVCGAVAFSVICWTGITFVEESIRKVHDKETNETTIVPIPKLMIRSAYPWNAMSGAAHIFSLIYQFYYLVITMGICNMFDVLFCSFLLFACEQLQHLKAIMKPLMELSATLDTVVPNSGELFKAGSADHLRESSGIQPSSNGENVLDVDVRGIYSNRQDFTATFRPTAGTTFNGGVGPNGLTKKQEMLVRSAIKYWVERHKHVVRLVTTVGDAYGVALLLHMLTTTITLTLLAYQATKVNGVNVYAATTIGYLLYTLGQVFLFCIFGNRLIEESSSVMEAAYSCHWYDGSEEAKTFVQIVCQQCQKAMSISGAKFFTVSLDLFASVLGAVVTYFMVLVQLK-

>QM_comp15607

MIGAGYFRVLQHRLFRAHNEAMARHYDDESLDADEQLVACVRLHQRILNYCSRIDEITRSFFFFQLLASGYNTSLIAVKLAGTDPDRYKYAPLSIFSLLQLFICQWAPDNLIDESEAVSMAAYYISFPWMKRGQMGKLLVLMIARAQRPVQLTALGAVNLSLENFGSMLKGALSFFTVLNSFNE-

>QM_comp20309

MVGELRLLTSRFRNLKSGDNYKRNLKECIDRHVLLIESKR

>QM_comp23253

MYPAALFKIELNKFNENTTNLPAVIFNGLLIRTRGMISNLACFAGNLYQLQSKMA-

Incomplete ORF:

>QM_comp02751

MENCWPGYWPTFFLQILIVQNALICGMTCDASTPLFAMICCGYFRHLRDRLLSVSHENFKRRERELINCIVFHQRLLTFCSSLTKFTRTMLFTQMFSTGYNISLILIKLSSSDRDKYKYVPIMCVSLCQLYLIQWAPDHLYDESEKVAYAAYSASTKCMENRHLSKCLLIMITRAQRPVQFKAGGFIPLSLETFSAMMKSA

>QM_comp05485

MSVFNSTYWQIPVLSQKFMGVWPYNEGTLHNKIMPVIFYIGMYCLAVPMGIRMINELGVNNMIALENLMGEMFFHGALIKFTINIVYKKELKAVYDLIANDWEKTLDKEELQIMEDYASLGRKMSIFYMISCLATAGSFLCIPTFKPLLDFVIPLENGSRPMVLPYYVEYYIDQEKYYYLLLVQGYVSVLISAIVYISYDISFAMCVQHVCSIFDIISLRLKRASQ

>QM_comp22594

MATIPNICINVYLLSQHRGGINLNIIDAFLCCSTCLMQIFLFCWYGNKITLESIDMGNAVFDTNWPAL

>QM_comp23347

MFVRKVSRLSCCYSGIILTTITLYVLSSQLPQLTAEHSNVTIHRVLPYPFYFDVQSTPNYEILLGIQVVCLLTVTQTSVCVDTSIAFLIMIACGHFRLIQVRLNGIARDIEDNEEGRKRPLAAVTKEIRDNDDDFQKIENLDFERSDEGIRKRLRDCVAYHSEILQFCDEIFTLSSEIFMIELISTTYNLSLIGILLAGNMPLAEKFKFAPVLLILTTQLFVCQYPPDLLLQESVGVADAAFIIPPFRKDRLRIDRLLLTLLTRSQIPYQLLAGGQIPLSIESFGNMIRGAVSFFTVLRNFN

>QM_comp23466

MCFVEMIGCCFNMCMLGYYILMERNQNFVVPCTYCLLFISFTFNIFIFCYIGDQLTEKCGNIG

>QM_comp23865

MKIFRCAKLLIIRLTENYDSVIRWNRSSLKCMGCWPNSNNKSNLLSTCQFFISAFMLFYFTTAPQTADLIMISRDIDSVIENLS

>QM_comp24669

MASDWRSVGSRVESALMLENTRSGRTFTIICITGVYMSCMSYATLFSFMMDPIVVDNRTTRIFAYPGYF

>QM_comp24798

MYAVCIEHCLGLFAIVKYRLQMPLKFIDDEHRACCCSESKGAAYNWMLHIIRLHTKILNFTVTLESTYKQSSFAIV

>QM_comp26249

MEIFESRYFAINRKLLGLIGLWPYEEARQKRLKRGFVVLSLIAMSLPHFTGMIVNYGKDKRMLFEHICVTM

>QM_comp26294

MMMRSLTVHKLTAGKMFYLSMETFG

>QM_comp21193

MSVILHLSGQFKLVNFRLQTLGRKISDGTLGINDSKLREELAQCI

>QM_comp21494

MYNSDWISLSATNRKSLYLIMLSCNREMSLSFHGL

>QM_comp22350

MMAMIMHVVGQFRLIGIRLKDIGIAMREGGESTRKIDVWTEIEYCVKHHQRMLKIADEVNSLLGPL

>QM_comp19418

MEYAEMIDVAKLGRSITKGMIIMTQSMVGVCLCLQLYLNIAGIYPTRRLYFKSYFPYNTSVTPVYEITWILQLITGILGALAYTSVDSFIGFSVLHVCGQLSILKNQLRSLSS

>QM_comp20582

MIFFFINVPQTVEMFRVNGDFNAILNLLTMTDVPIAIAFVKVFCVGLNNKVLDKLIVSIAEDWATTTKESELSTMWFIAKASRLISVVSISLGECTA

>QM_comp20892

MVTRNPLELDILKVDMLFLIILAQLLIWCWPGELIIEK

>QM_comp22574

MISFSSALFKALVMFIYRDDVIKLNRTLSYRFEKDLEVPENRP

>QM_comp22810

MAIGVSSVLLALIYIVSVSLYLHSRKSRGKDNEEREPEVGNCVVDVDPPGLVKCNPLLSVSRHFESDTNSATSENDLADKPLHSESESEIENVTSAVIHPYEECTEPSEPNSNPGPIIGERVPEENVRVIETTENANHQSMPVFPGTQRRKLYFNPAYFEK

>QM_comp24809

MNQVALFIGAFTHSNCLLASTAGPIQLASTQFLAITHCKWSANCVYWHPDFA

>QM_comp26358

MYELIANSLNPAKFILVSIYLSNMLIQLGVYCYFGNEVTVQVKKRKN

>QM_comp21252

MACNVAILSVGSVVVLMNLGKPMELFRYGLIYAGLMIHMFYLSWPGQKMIDSSGGIFYDAYNNEWYDCSLKSQKLLRFMMLRSSEPLQLT

**6. SNMP protein fasta sequences of *Q. mendeli***

Complete ORF:

>QM_comp09081

MGKLSLMKKLGIGGGSMFVLSMLFMFGFPPLLVSMIKSQINLVPGTDVRGVWSKIPFAIDFSVYLYNVTNADEIKNGAKPIVRQVGPYFFEEWHEKENLIDREEDDSVEYAIKNTFYFNAEKSGAGLTGEEELMLPHVFILAMIMTVKRDKPSAIPVINKAVGSIFKNPDNVFVKVKAMDLMFRGLMIDCTVKDFTGGAVCGMLKENPDGLIVYDEEHFGFSLLGAKNATASKQRMRVLRGRKNLMDIGKVIEYDGKPNISKWDDERCDAFNGTDSYVFHPFLYENEDIVSFSPDLCRSLSTVFQKKTKVAGLKTNRYTAYLGDPSTDEALKCNCEAPDKCLKAGMMDLHKCLGVPLVASHPHFYMADESYLTMVDGLSPTREDHELFLDFEPFTGTPLFAKKRLQFNIPITKIEKFKIMKNFPDAMLPIFWIEESVMLPDELIAQVKMIHTLLTVVAVLKWLLLVGGLGMGGGAGFLYYKATQSSNKLEITKVPKQNGKPSSTEKKISPLDVNTLQASQVPPVLD-

Incomplete ORF:

>QM_comp21591

MKNLSIWKDDKCNMFEGTDGFIFPPFSYMKNSKEDIILVNQFLCRKLWLRYDSDVEFSGLKLQRYVLDIGTDGDHVPEEKCYCITPDRCLRKGMFESFRCTNTPVIISNPHLYLAD
