# Supplementary material for: Sensory gene identification in the transcriptome of the ectoparasitoid Quadrastichus mendeli
Source: Sci Rep. 2021 May 6;11:9726. doi: 10.1038/s41598-021-89253-w (PMC8102506; doi:10.1038/s41598-021-89253-w)
Supplement: Supplementary file 9 — Additional File 9. [file 41598_2021_89253_MOESM9_ESM.doc]

**S9. Amino acid sequence of CSPs, GRs, IRs, OBPs, ORs and SNMPs from organisms other and *Quadrastichus mendeli* used in phylogenetic analyses.**

**1. CSPs**

>AmelCSP1

MRHNYIVILILSLLTWTYAEELYSDKYDYVNIDEILANDRLRNQYYDCFIDAGSCLTPDSVFFKSHITEAFQTQCKKCTEIQKQNLDKLAEWFTTNEPEKWNHFVEIMIKKKDEGA

>AmelCSP2

MASAIKALLIVCALFIYTVTAETEEGQSGRSRVSDEQLNMALSDQRYLRRQLKCALGEAPCDPVGRRLKSLAPLVLRGACPQCSPEETRQIKKVLSHIQRTYPKEWSKIVQQYAGVS

>AmelCSP3

MKVSIICLVLMAAIVLVAARPDESYTSKFDNINVDEILHSDRLLNNYFKCLMDEGRCTAEGNELKRVLPDALATDCKKCTDKQREVIKKVIKFLVENKPELWDSLANKYDPDKKYRVKFEEEAKKLGINV

>AmelCSP4

MKTILIALVPVCFLLGEVFSEDKYTTKYDNVDIDVVLNTERLLNAYVNCLLDQGPCTPDAAELKRNLPDALENECSPCSEKQKKIADKVVQFLIDNKPEIWVLLEAKYDPTGAYKQHYLQNRVKEESY

>AmelCSP5

MKIKILLFFTILALINVKAQDDISKFLKDRPYVQKQLHCILDRGHCDVIGKKIKELLPEVLNNHCNRCTSRQIGIANTLIPFMQQNYPYEWQLILRRYKIMKYY

>AmelCSP6

MKIYILLFVLVTITCVIAEDYTTKYDDMDIDRILQNGRILTNYIKCMLDEGPCTNEGRELKKILPDALSTGCNKCNEKQKHTANKVVNYLKTKRPKDWERLSAKYDSTGEYKKRYEHGLQFAKNN

>BmorCSP1

MKCLTIAALLFVAGLSIAEKYTDKYDNIDVDEILENRKLLVPYIKCVLDEGRCTPDGKELKAHIKDGMQTACAKCTDKQKVSARKIVKHIKQHEADYWEQMKAKYDPKDEFKEIYEGFLAGQN

>BmorCSP2

MKSVILICFLGVATVVIARPKTPFDNINIEEIFENRRLLLGYINCILERGNCTRAGKDLKSSLKNVLEENCDKCSEDQRKSIIKVINYLVSSEPESWNQLKSKYDPEGKYLIKYEAKMESN

>BmorCSP3

MNSLIAFCLFAVLAVALARPDDKYTDRYDNVNLDEVLSNSRLLQPYIKCILDKDRCAPDAKELKEHIREALETECAKCTEAQKKGTRRVIGHLINNESKSWNELTAKYDPENKFTAKYEKELREIKA

>BmorCSP4

MKVLIVLSCVLVAVLADDKYTDKYDKINLQEILENKRLLESYMDCVLGKGKCTPEGKELKDHLQEALETGCEKCTEAQEKGAETSIDYLIKNELEIWKELTAHFDPDGKWRKKYEDRAKAKGIVIPE

>BmorCSP5

MKTVIVCLLALTAVALARPEQYTDKYDTVDLDQLISNRRLLIPYVHCILEKGQCTAEGKELKSHIKEALETNCAKCTKAQKGGTEKMIGHLINHEAEFWEELKAKYDPTNEFTKKYETELKRVTA

>BmorCSP6

MKSLIVLSCLLAACLAADLSKYENFDVEPIVTSDRLLKAYINCFLDKGRCTPEASDFKKALPDTIATNCGKCTEKQKANVRKVIKVIQQKHSTEWEKLVKKHDPSGKHRADFDKFLLGS

>BmorCSP7

MKGFYVLCFALFAAVYCKETYSSENDDLDIEALVGNIDSLKAFIGCFLETSPCDAVSGDFKKDIPEAVAEACGKCTPAQKHLFKRFLEVVKDKLPQEYEAFKTKYDPQGKHFDALLSAVANS

>BmorCSP8

MKTILILCALVSVVVCRPEEYYSSQYDNFDVEQLVGNLRLLKNYAKCFLDQGPCTAEGTEFKKRIPEALRTKCAKCNPKQRHLIRTVVKAFQTKLPDLWEELAIKEDPKGQYKHEFTAFINAMD

>BmorCSP9

MRAVIFLYTCVFVVVGQDINAMMSMPKYDERYDYLDVDDIFRNKRLVRNYVDCLINAQRCTPEGKALKRILPEALRTKCIRCTERQKRTSVKVIRRLKNEYPEEWAKLASRWDPTGDFTRYFEDYLAKEHFNTIPGSGPTVNVLSLQTTPPPPPPPPSRPASVFTNPPPPVMSTSPRPVVLNRFRR

>BmorCSP10

MACVAVTWARPESTYTDKWDNINVDEILESNRLLKGYVDCLLGKGRCTPDGKALKETLPDALEHECVKCTGKQKSGADKVIRHLVNKRPDLWKELAVKYDPDNIYQARYKDKIDAVKGSA

>BmorCSP11

MKLTSFLLVGMAMVSAEFYSSRYDDFDVKPLVENDRILQSYTNCFLDKGPCTPDAKEFKKVIPEALETTCGKCSPKQKQLIKTVIKAVIERHPEAWEELVNKYDKDRKFRPSFDKFINEDD

>BmorCSP12

MFMLFIISFIIVPVLKCCGTETSTYTTQYDEVDIKEIMGNERLLVAYIGCLLDKNPCTPEGKELKRNIPDALQSDCSKCSDKQRENADAWIEFMIDNRPEDWTKLEER

>BmorCSP13

MKLLLVFLGLFLAVLAQDKYEPIDDSFDASEVLSNERLLKSYTKCLLNQGPCTAELKKIKDKIPEALETHCAKCTDKQKQMAKQLAQGIKKTHPELWDEFITFYDPQGKYQTSFKDFLES

>BmorCSP14

MKSSLFCVLVLTVVVSSSRQQSYPRNDNININAILQNDRILLGYFKCVMDRGPCTKDGKTFKRALPEALPTACARCSNKQKAAFRTLLLAIRARSEPSFLELLDKYDPSRSNRELLYTFLATGL

>BmorCSP15

MIENFYSKCTISKSVLFLCLIFLPYALNQKYYDSRYDYYDIDHLVQNPRLLKKYLDCFLGKGPCTPIGRLFKQVMPEVITTACAKCTPTQKRFARKTFNAFRRYFPETLMELRRKFDPESKYYDAFEKVITNA

>BmorCSP16

MIEWKRFKILHFLSYLGLLVLVVVCAAQQNRPQVTDTALDEALNDKRFIQRQLKCALGEAPCDPIGKRLKTLAPLVLRGACPQCSPQETKQIQKTLSYVQRNFPQHWAKLVRQYAG

>DmelCSP1

MWHCELKVFLILWTVSQVYIPCWGKKFISRFESINGIEGEKETLFTCSVRLVGRERMLNGSIMHQVDLDDSFDVWMDILHFKNGEWAQGNIKVRTKPCDWFTNYFGKYFLPLVKDSNLPPIQEMCVFPKGEYYLRITKIEPQNWPPILYRGLNQFNINYVRDGKSTGGIQFVIDLEDSTL

>DmelCSP2

MISISTILALVVSSVWATDYTLEFEDSDLYSECSEKLPGAIGLREAFDMRNIVTELDIDGLHLSGNCTTIWDVPSTDRISLRMTVMHFDRGTWQPTVFNTYARDFCAVMFDKELSWYKYWLKYFANREEISEKCIGTKGTVLVYKPFIVKPLIQNVIGPIYRGRVKAIFNFESFDKNNVKGATDVCFEVRGQVEKIK

>DmelCSP3

MGSSLCPVSYLAVLAIIVLTSNITVQAKRTFRIQKLEKVTEDTSYLRSRLRIAESEENELKVSGYLDLNQRLDNDWTVVLKVSRSPDSDGDYEKVLTFEMQLCDFMKSYYKDIFYERIKEYSNAPHPSSCPLPKERYVLEDYPFNVKLLKKLMSPGFYRIKYTLKNEETKILSYVLDLELEEN

>DmelCSP4

MKATFTILVLQVVICLAGATEYQLTLDKDGLLAPCENQPGNPSGFEAMVDTSSLKVHNLGSKVRIEGEQKVVWKDVQPGDTLKVFGQVYRLDKGTWQKTMFTASSNNFCKNMFDKNQYWYNFWTKYISNSDEIKEKCLTTPGAVLKYKDYELDLKTSLNVPNLDGRYKLVVQIEAFDKRNVRRPVPICIEFRGTAGQV

>DmelCSP5

MRTLMLFVFGFASSWAADYELLLEDPDIFSPCTEPPPGSIGFHDAFDIGDLVVDQDMDIIHLSESVTSIWDVEPTDRISARFAIMHYNRGSWEPTVFSMATPDFCASMFDENQSWFKYWTKHISNRDEVMEKCFKTRGTVIMHNPFDLQLRLTDIRGATLRGRYKAVVTFEAVDEKDVPRRNSICFEIRGEAEKIN

>DmelCSP6

MIFVLLLLLGVTSSWATDYELLLEDPDIFSTCTDGPPGSINIRQALNLDDIVIDQKGDILHVSGNATVVWDVQPTDRITARLDVFHFNRGTWEPTVFSMATQNFCSIMYDKNQYWYKYWTRFITNRHEVEKKCFRGPDTVLVHEPFDLILKFENFRGPLLRGRHKLVILFNALDERNIPRPNPICLEIIGEPLKLQ

>DmelCSP7

MGPITLLLSILLVHSCRAEKPYSVELNTFTMDDTIENQENWVDWGTLSMKKVSRNQFVVSGDFEFKLNMADEQKIVLMVYVYDSNANQRGSMVMAVKKPFCQFIKEDEDSYPSIQKASNLPDQDTCPFPKGKYTIDNYELETNFLPDNAPKGDYLLQLSLLDREVPVAGLVATVTLT

>DmelCSP8

MFATLLILILGSTDILATDYILLVEDPDIYTPCTDGPPGSVGLNEAFDVSEMQVEMDEEGIHVSGNITTRWSLPPTYRISARMSVLHFNRGNWEPTVFNTLTPDFCDAMFNPNLFWYKYWFKNFENREEIQEKCLATQGTVLVYNPFVVVPRLNNVLGPTLKGRYKVVFLFEAFNEQDERQPSSVCFEITGDAEKIKN

>DmelCSP9

MKWLVIIVLLQLLEKIKCEQSYEVTNERLEPFEGDSQTLVLFDGLKTIGRERALNGSFKFLGEMNNDDFKVSVELYSSPNGDGEFKRMVMDVPQTSICECFKKFYVQFVQPSLKTGETTNFPVVDDDFCPVPEGEFYVKNVILNTQDWPSQVPRGIVKAIITFFSGGKNVGGLIVEVKIEDRQS

>DmelCSP10

MDRQQVTLLNILISLTLQIISPSFALNHFFIPEREELFSECLDKPGFSYVNELADLSRFNRKKDADGAMNISGNITMLWDVEPSNRVAVEVNIEKFEGGTWNPTLFKGGDKDFCKNFYDKNTIYYPFSTKHVINKQQVKDKCITTPGTVLVLEPFILKILINFAVPLSPGIHKAVIIFSAFDKSGVKRPRDTCIEIVGEIVNI

>DmelCSP11

MIRGLVILVLALANTWATDYNALIDDEGIYVKCSEAPAGTLGPRDVFNIDNMVMHMEPEGIYVSGNMTVKLNFLPSDRISARFSVMHYERGSWQPTMFNLHSPNFCEVMFDEDQYWFKYWFRYIRNKEEIREKCLKVKDTVLVYDEFLMVLHLENVNTSNLQGRYKAVITLEAFDEHNVRRPSSLCVEIRGDLERVT

>DmelCSP12

MGCETSVLCTLLIFFQIIGRSFEMSHDFVPIKDDLLSKCEDKPEMGYLDAFVDLSNFSRKRGPGGVNISGNITTIWDVDPSDVVEIDVSILKFEGDKWIPTIIKGNVKDFCKSFYDKNTLYYSYSTKHVVNKKEAKEKCITTPGAILLWEPYLLKISFSYAVPLNVGRHKAVLIFTAIDKAGVKRDRDICMEIVGDIVNA

>DmelCSP13

MDAKIIILALVSLFYKANGAFYELSVADEEIFSSCPNPEPGTLDIHGLFDFSEFSTSLEADGLTVSGNQTLVWDIQRGDRVQLFIKLFYFDRGTWTSTAFSILSQDFCKTMYDKSNVLYEPWTGHVMNDVKDQCINAPGTKLILDTYFLSLSASVTVPLREGRYKTTIKFRAFDSKGTERPTSICCEVIGDVFKIRN

>DmelCSP14

MKQFLFCVLIMLIGKTCALIPRTYETRFISITSNGTNLFDFSQIRFLGRERMANGTFELKEDLDNESFSVVGETFIDSVGDGEYKQLPFTAPKQSVCTALKAYWSYFEPSIKYGVKTDFPAHTHPCPLPKGIYYIKDVVLKNDNWPVIMPRGYLKAVANLFKNDEYGGSLEIVSQISDLS

>DmelCSP15

MLKYTVLVLLIGIPKLLLQAQMSYEAIFVSVTSEENSKPFDLSNLRLIGRERILNGTFEILEDLDDEHFQISVEIYTNPARDGNYKLLPMSVPRQGVCTFFKKYGFYFRDCIKNGINTDLFLNTTSCLFPKGHYYLKNVTINVQNWPKIMQRGLCRHIAFFYKNNVPMGSYNLTSSIEDRAPNFNLRPL

>DmelCSP16

MLELILILNVVHLSLQISYEFEIEDESIYSDCSDVPPGTLNISGLFDLTNYTTTITADGLSVSGNMTSVFNAQPTDRIELTGNLLFFDRGAWQPTTLNMMVRDFCAVMYDKKQLWYTDWSSHVVNRDEIKDNCIKVPGTLFLIESYNMKLVFGSGIPLGTGRYSIRVQVFAYDQKGKKRPNNVCYEVKGNFYKESNICISKPILITINVLAESLKSQMSSTNHANI

>DmelCSP17

MSQQLFRIWQLLVLMAFIPHSIEVSYEFTMEDERITSDCQNEPPKTLNIDGLFDMSNIDFEMAEDGVQLSGYKTVVWDIQPTDRVELQGSVQYFDRGTWQPTTLNMLVKDFCHVLFDKKQVWYDAYSKHITNSAQINNTCFRVKGSVIEFETYTIGLEFGSGIPLHRGRYAIRLKFRAFDKNGKVRPNEICFEIKGQFSKKSLG

>DmelCSP18

MDFQFSVLCQILIFLTAAQPSIGGYFEYVLDDESVFSECFNTPPGYANVSGLFDVSTVNFEMGPEGVHIDGHVTSTWDIQPTDRIEGRLNVVHMDRGTWQPTVLNMVCKDFCKTFLDPNQYWYNVFPKHIINKDEARQKCLNYKGTVYFVEPYTLQMHFGLGLTLPSGRNRMVINLVAIDENNVTRPNGICFEVKGDFFKIE

>DmelCSP19

MSLELHKVLLVLSIAHSALVRAGLECRIESISKVFGDNETLFEFNFRVIGRQRLLNGTLNFHVDLDDDYEMSNEVLALKDGEWESTSVSARFKTCKYMAVIYDKYFAVSFKDSNIPKGTEACPIKKGEYYARNVEVIADNWAHYAKLGLVRSNMLVRKNNVVYGGFDIVLVLSQKIV

>DmelCSP20

MSFWWMIQSLLILWTVSVGSKKSNYEVRFESIDAVKGSTETLFLYQLRLLGRNRMINGTLIFLEDLDETFDVLFESHAFKNGYWVKGIVNAAASKPCEFFNRYYISFFLVKSTESNLPTTGAEMCPFRKGTYFVKNGVVSTEDWPPIVFKGLNRFTISYLKNGECVGGVQLTISIAEIIT

>MmedCSP1

MKMFIVLMLAAVTVASVSSFVSAEAVNNKGMYSTKYDNIDINAIIKNERLLNNYVGCLMDEKPCTPDGAELKKNLPDALASECASCSPAQKNIANVMYHHLIDNRPDLWSKLETKYDPSGGYRKRYLNQDHDQDQNEGNEEEIKSTTMAI

>MmedCSP2

MKYLGFLVIAVIFSAVSCDELYSDKYDNLNVDEALANAEVRQTYFNCFMDKGPCGEDATYWKGNFPEAIATNCKKCTEWQKEAFDKIADWYTVHEPDNWNSFVDKMVQGARNFGDSRK

>MmedCSP3

MKVAIIFLAIIAVALAATTKTYTSKFDDVDVDGILGSDRLLRNYVNCLLDRGPCTKEGVTLKEILPDALATSCESCTEKQKTKSEKVIRHLVNNKKELWDELAVKYDPNNEYRKKYEDQAKAKGINV

>SinvCSP1

SGCVVSLVVLAVADEKYTRKYDDVNVDKILQNNRVLTNYIRCLMDEGPCTAEGRELRKTVPDALSSGCDKCNDKQKAMTEKVIDHLKTKRSRDWDRLVAKYDPNGEYKKRYEKS

>SinvCSP1

MCILAEELQPYPSEYDIYVPKILANDVVRQKAVDCYLKKGPCTEQEKLATDLFRDALKTNCKKCGEKQKEHVKILTEWFVKNQPDTWKLIIENVDS

>SinvCSP2

MCVLAEDLHSELDDLDIPKILANDAERQGVIDCILENASCTELETKAAAAIKDALKTNCQACGDKRKENMKIITDWFNQNQPDTWTLVVAKVNS

>SinvCSP3

MNVLMCVFGEELELYPREIDDIDVLKILSDDAWRRRAEDCYFKRVPCAKEKQYLSDIFKDMLKTKCEKCTEKQKKLVKTATEWYEQNEPDTWKLILEDAHS

>SinvCSP4

MSVLTCVLAEELWFYSGEFDDMDVLSILEAQAEQEVDCYMKRGPCTLEQQRIADSIREAIRTNCRRCTPKQKQQIQLITDWYKSRMPQNWELIVANVDL

>TcasCSP1

MLILQIAHLCAQFCLLAAIFTCVKPQLTRISDEAIESTLNDRRYLLRQLKCATGEAPCDPVGRRLKSLAPLVLRGSCPQCTPQEMKQIQKVLAFVQKNYPKEWNKILHQYAG

>TcasCSP2

MKIIILAVLIATAVAATYDVYPTKYDNVDIDAILHNKRLFDNYLQCLLKKGKCNEEAAILRDVIPDALITGCRKCNDHQKVSVEKVIRFLIKERNSDWQQLISVYDPKGEYQTQYAHYLEKI

>TcasCSP4

MYSYLIPLYLFLFVHYGWSEDTTHKYTTKYDNIDLENVVKNERLLKSYVDCLLEKGRCSPDGLELKKNMPDAIETDCSKCSEKQKEGSDFIMRYLIDNKPDYWKALEAKYDPDGTYKKRYFESQKDEVSKVEA

>TcasCSP5

MKTFVILFFGVFFIIFSDFVNGKTLHRSTRDDKYTTRYDNVDVDRILHSKRLLLNYINCLLEKGPCSPEGRELKKILPDALVTNCSKCSEVQKKQAGKILTFVLLNYRNEWNQLVAKYDPDGIYRKQYEIDDDYDYSELDSAKK

>TcasCSP6

MIPLIAIAGILAVSAAPAEFYESRYDHLDVESILNNRRMVNYYAACLLSKGPCPPQGVDLKRVLPEALQTNCAKCTEKQRTAAYRSIKRLKKEYPKIWEQLRAVWDPDDVFIRKFETSFESGKPSGVISTNTSPPSPILSNRFGENEEADAASNVISSTPLPPTTSTTTRTTLTTKFTTKPSTKPTNKPVVVTKPPQAPPFATVGANLQATVSFGTNLVGGIVRSLGTLGSRVVESGTKLANMVISAAIRP

>TcasCSP7

MKLISAVILCAFLVAVSAAENKYTNKYDNVDVDKILNNDRVLTNYIKCLMDEGPCTSEGRELKKTLPDALSSGCTKCNQKQKETAEKVIRHLTQKRARDWERLSKKYDPQGQYKKRYEEHVATSRAA

>TcasCSP8

MPLVKSLVVVVLLIGVVYQVQGQLGLAGNNYIEKQLLCALDKAPCDALGNQIKGALPEIIGKNCERCDSRQVANARRIARYVQTKHPDVWNALVKKYSV

>TcasCSP9

MTAIVFLLALACLKTYVSSQEYLVPQNIDVDEILKNDRLTRNYLDCVLGKGKCTPEGEELKKDIPEALQNGCAKCNEKHKEGVRKVIHHLIENKPNWWQELESKFDPQGEYKKKYDELLKKEGLAN

>TcasCSP10

MKTFVLVAFAAVLGLALARPQEKYTTKYDNIDLEEILKSDRLLKNYFNCLMERGTCSPDGEELKKALPDALHSGCSKCTEKQKEGSRKIIHYLIDNKRDWWNELEAKYDKDGVYRQKYKDVIEKEGIKL

>TcasCSP11

MKTLVPLLFFVIAIASSLAENSKYTTKYDNVDLDEIIKSDRLLKNYVNCLLEKGKCTPDGAELKRHLPDALHTECSKCSETQKNGSKKIMRHLIDHKRDWWNELEEKYDKEGEYRKKYEAEIKGKKD

>TcasCSP13

MFLAIVLVVCACTNVLSEEYTNQYNDELDAALKSERLMKSYFECLLGTGKCTPSGEELKKDIPDALKNECAKCNDKHKEGIRKVIHYLVKQKPEWWEQLQKKFDPQGIYKKRYQNYLDKEGLKA

>TcasCSP12

MKTLVLVLFVAVLSVVFAADKYTTKYDNIDLNQILKSDRLLKNYVNCLLDRGKCSPDGQELKNNLADALQTSCSKCSQRQKDGSRTIIRYLIKNKRDWWNELEAKYDPTGIYKNKYADELKAEGIVL

>TcasCSP14

MFATSALFAFICIQGLVSAEEYLVPQNIDLDEILKNDRLTRNYIDCILGKGKCTPEGEELKRDIPEALQNECAKCNEKHKEGVRKVLHHLIKNKPNWWQELEAKFDPKGEYKQKYNKLLEKEGLQA

>TcasCSP16

MTAIVFLLALACLKTYVSSQEYLVPQNIDVDEILKNDRLTRNYLDCVLGKGKCTPEGEELKKDIPEALQNGCAKCNEKHKEGVRKVIHHLIENKPNWWQELESKFDPQGEYKKKYDELLKKEGLAN

>TcasCSP17

MFKVLFVVFACVQAYVYAEEYTVPQNIDIDEILKNDRLTKNYLDCILEKGKCTPEGEELKKDIPDALQNECAKCNEKHKEGVRKVIRHLIKNKPSWWQELQEKYDPKGEYKSRYNHFLEEEGLN

>TcasCSP18

MLFTVFLVLTCAHVVFLEEYVIPDNIDIDDILSNERLLKNYVNCLLDKGRCTPEGKKLKSTIPEALSTDCAKCNEKVKANVRKVLHHLIDNKPDMWKQLEAKYDPSGEYRSKYKDELEKNGIHV

>TcasCSP20

MRFFVIFFVACVSVALARPEDQYTIKYDNVNLKEILQSDRLTENYVNCLLEKKPCTPDGEELKRVLPDALKTSCAKCTDKQKQGAKTVIQHLYKNKQDWWKQLEAKYDPEHTYVKAHEDELKAL

2. GRs

>AmelGR1

MVLVGDTNLIFNAGLILGCTALCASKDNVKARRLRDIIFIVFSMTLYLVSLVVIYIYVFSYEDSDLKSTLIVIIRVFLIYLCLFTDASVTTLWNWKIRSVLSQLRNFDRATKFRDFSKGNKLRIICHVTMFVSFSYWAIVGYFTYRIEAKVPIFHGIIYFIMDASMNTQILIFVCILFLIEERFRHLCSMIELSKADKIIEAHRSIRHSTLQQIWWLHCSLANATEIINSVYAIQLLFWISSMSFNLMSRIYSLKVFKLSDYGKIRESMLVTDCAWNLVLITTVCHMTAHQANRVGELIFSPYSSVSLKRVHLQENIEAAAYFQLRKVHLFTVAGLIRVDLPLLLSIFSALTTYLVILT

>AmelGR2

MVLVGDTNLIFNAGLILGCTALCASKDNVKARRLRDIIFIVFSMTLYLVSLVVIYIYVFSYEDSDLKSTLIVIIRVFLIYLCLFTDASVTTLWNWKIRSVLSQLRNFDRATKFRDFSKGNKLRIICHVTMFVSFSYWAIVGYFTYRIEAKVPIFHGIIYFIMDASMNTQILIFVCILFLIEERFRHLCSMIELSKADKIIEAHRSIRHSTLQQIWWLHCSLANATEIINSVYAIQLLFWISSMSFNLMSRIYSLKVFKLSDYGKIRESMLVTDCAWNLVLITTVCHMTAHQANRVGELIFSPYSSVSLKRVHLQENIEAAAYFQLRKVHLFTVAGLIRVDLPLLLSVSSAYNDAAILYNISYNIDSEDIIRHLLAKFQGKTNYLLIVLYAFVMALAVTANILVIAVVFKYHYMRSVTNYFVVNLSVADLLVTTICMPVAVSQAVSLKWIHGEVMCKLSFYLQGVAVAASVFTITAMSIDRYLAIRSPIAFRRVFNRKSTVFVIIALWVVALIIFSPLLRVTSLQNPGIMILRNISFHGSNFAQNISTDLYFYMCLEDFKSVGIEAPLFGTMCFVLVYAIPGFVVVLSYIMMGRTLCARKPPFDCDGAQGSASSQQSFRLVRERRRIAWILLLLAVLFALCWLPYNVLMLLKDLDVISVRGTENGEAEGWNNAISYCLFLGHANSALNPTVYCFMTRNFRQNVTEILCRSPCGLTRETTHRRGVQGTGVIDDMCAGSASGTIRRSLLRKRKMLPGCGCGLPIGGHRAVLTLRRTTTTSCDSFYTRHSPHRRCYMLRSLRGRPDTAIGHIQLRKSQETDREKSNAVQPANTLVVTDEKR

>AmelGR3

MSIYDNTGKNLNGMRPLNAPAVKYQNTLLFNIKSPKSSSKFGKFDNLKTEIETIGMSEPVAFSANSFNPKTDSLHASMRPIIMLAQFFSLFPVSGVNSPDSSYLRFTWRSPKFIYCTISFLSSSIMTIFNVLRIVTTGISSIKMTTFVFNGTNLIASFLFLKLAMRWPCLMVTWEKLEKELSQRHRKISKISLSMKFKIVTIVVMTFALVEHSLSIIHGYFKAKECIEFHREQSILGVYFQMQFPQIFSRTSYSLWKGILVDIINILSTFSWNFVDLFLILISIALTDQFRQLNSRLYSIRGKAMPEWWWAEARSDYNHLATLTRQLDSHISIMVLLSFATDLYFICIQLLFSFNPMRGIIEKIYFGFSFGFLLARTTVVSLCAATIHDESLLPAPILYSVSSSSFSTEVMRFLSQVTTDNICLTGMKFFSVTRSLVLTVAGTIVTYELVLVQFNTTQQTDASNATIVCELVSLDSLSS

>AmelGR4

MSIYDNTGKNLNGMRPLNAPAVKYQNTLLFNIKSPKSSSKFGKFDNLKTEIETIGMSEPVAFSANSFNPKTDSLHASMRPIIMLAQFFSLFPVSGVNSPDSSYLRFTWRSPKFIYCTISFLSSSIMTIFNVLRIVTTGISSIKMTTFVFNGTNLIASFLFLKLAMRWPCLMVTWEKLEKELSQRHRKISKISLSMKFKIVTIVVMTFALVEHSLSIIHGYFKAKECIEFHREQSILGVYFQMQFPQIFSRTSYSLWKGILVDIINILSTFSWNFVDLFLILISIALTDQFRQLNSRLYSIRGKYHFIVKAMPEWWWAEARSDYNHLATLTRQLDSHISIMVLLSFATDLYFICIQLLFSFNPMRGIIEKIYFGFSFGFLLARTTVVSLCAATIHDESLLPAPILYSVSSSSFSTEVMRFLSQVTTDNICLTGMKFFSVTRSLVLTVAGTIVTYELVLVQFNTTQQTDASNATIVCELVSLDSLSS

>AmelGR5

MSEPVAFSANSFNPKTDSLHASMRPIIMLAQFFSLFPVSGVNSPDSSYLRFTWRSPKFIYCTISFLSSSIMTIFNVLRIVTTGISSIKMTTFVFNGTNLIASFLFLKLAMRWPCLMVTWEKLEKELSQRHRKISKISLSMKFKIVTIVVMTFALVEHSLSIIHGYFKAKECIEFHREQSILGVYFQMQFPQIFSRTSYSLWKGILVDIINILSTFSWNFVDLFLILISIALTDQFRQLNSRLYSIRGKYHFIVKAMPEWWWAEARSDYNHLATLTRQLDSHISIMVLLSFATDLYFICIQLLFSFNPMRGIIEKIYFGFSFGFLLARTTVVSLCAATIHDESLLPAPILYSVSSSSFSTEVMRFLSQVTTDNICLTGMKFFSVTRSLVLTVAGTIVTYELVLVQFNTTQQTDASNATIVCELVSLDSLSS

>AmelGR6

MEVKRVEEKRKILFNNELCQAIFPIYYLGKFCGLVPVRFFVHTSEGCQARLNIIDLIYSLCVLVLLLSAEIWGLWRDLKDGWEYSTRLKSRTAVIATCSDVLGVMSLTVVCIVGSPFRWKYLQLVINKLIEVDEKIGVSSAKVARRFTIVLTICSLSYLWFNSIIDFYTWNRKTKVDNKAMTGKGPINYAPLYFMYTVIISTEIQYTVSTYNIGQRFIRLNTSLKDLFNANSNNNDNAIDYFRKCPETAHDMDDKKIWNLKPKRQIVLGSYRLSRKLDENKMYVNNISELIMVHSSLCDAVSLINSTFGVVILAVTVTCLLHLVITPYFLILQAGERHEWIFLIVQGGWCIFHITRMLIIVQPSYSAIAEAKKTAVLVSQLLSCTFEANIRRELEIFSLQLLHRPLEFSACGLFSLDRNLITSIAGVVTTYLVILIQFQNADDTKDDFDIIRNATQILKNASPLQNFTGLKTIV

>AmelGR7

MHSEDQIQLMMLKTKDGLGEIPKGKGRGSNLKIWSSVMYHKDDNNIEDISANQENDLSTKRPRAERNYFRNSEALENFHCAIGPVLKAAQIFGMFPVSGIGSSSLSKLQFKIFSLLTMYSGFIALMISFMTIVSMIHMLKTFNASTFQIRGGLGAATVGAVFYGNSLVGSILFFSLSSRWVSLQYEWRAMERYIDSNSTEPTRLRWKFFIISTMVLVLSLIEHVLSIFNNIDGYEWNESNSTFHNFLEIYTLRSHSFIFDTLNYNFVYGLYVFVVSKLATFTWNFTDLFIMLVATGLAERYKSLNKKLAVTMTKCQAAFNWRELREDYAILSCIVKKVDDHISPIILLSFANNVYFICLQLLNGLSISDKNSVLSEAYFFGSFAFLICRTCAVTLLTARIHDQSKQALPYLYNCSTSSYSVEVQRLQCQLATDDIALTGLRFFSITRNFMLAVAGAIITYEVVLLQFNGK

>BmorGR7

MVLEAHTQIQYCTAKANYCEFHAGLRHLMRLARWAGFFPVQGLSQTNPDDVRFEFRSLYALYHAITVIGQTVMTFLAFYSFVDSNVSLSVVSNFLFYFTNYVTLVLLWRLSKNWSALISKTLEFEQSVTEIRTTRNLVSRTNTLTYVVLIFAMIEHALSKVFNIRSVMCCLGETSLNHTVINNYFKFKWKFVFDYFSTSTTYSYFVGFIAEFLCMQATFLWSFTDVLIMCFSIYLSSFFEDFNSTVSSFMKKASKTVPWSTLRVQYSQIVLIVKQMDEQLDYFVLISYFTNLFFICFQLYNSLNRIYDANDVCNENMDIIATASVTYLTYYVFSFLFLVTRALLLSIMAANVHSCAQVPQLALYEVPTADYSLDVQRFQLQLRYTTVGLSGVCFNVTRGMILRVIGTIVTYELVLIQLTKKNLDNDTSIRDYYLPKHLI-

>BmorGR8

MAPRSVRSMVGTSKKDMLKGGFYETVRIPLYIYRLIGILPISGLWHRSSKYNRFSLKSFYTIIYAPTIVMQTFLLLVHIYDLFAFFFGHQRLGRLIYHMNFYTITILIFMGSRKWKNVIKEIETIELTLPRLRNSKKALALTKSFVFAFFVFSLAEVVLILQFTLRLTKQRHVLPGDSGLYLRSYFVYIFPYLYDHFPFSYVMGFIVQIIKVQGIITLNMVNCSVVILSIYLTNRLKHYNRIVFAKGSKTNNTRLKWVELNLLYTRISNLVKIIDKNLNPFVFISFTANLSYICAQLFYILNKLTSSRTVKITSFLEDKRSDWETVLYISISFALVVLKVLLVSITAAEVHTTSREPLRLLYTLPTAEYTIETQRLMTQVYYSNLSLSGLNFFHITRGMLLGMVATLLTYEIVLLQI-

>BmorGR9

MPPSPDLRADEPKTPCLVGGAHAFILKISSFCGLAPLRFEPRSQEYAVTISKGKCFYSYILVTFLVICTIYGLVAEIGVGVEKSVRMSSRMSQVVSACDILVVAVTAGVGVYGAPARMRTMLSYMENIVAVDRELGRHHSAATERKLCALLLLILLSFTILLVDDFCFYAMQAGKTGRQWEIVTNYAGFYFLWYIVMVLELQFAFTALSLRARLKLFNEALNVTASQVCKPVKKPKNSQLSVYATSVRPVSCKRENVIVETIRVRDKDDAFVMMKTADGVPCLQVPPCEAVGRLSRMRCTLCEVTRHIADGYGLPLVIILMSTLLHLIVTPYFLIMEIIVSTHRLHFLVLQFLWCTTHLIRMLVVVEPCHYTIREGKRTEDILCRLMTLAPHGGVLSSRLEVLSRLLMLQNISYSPLGMCTLDRPLMVTVLGAVTTYLVILIQFQRYDS-

>BmorGR11

MKPFRFFLFVENVICVYRNYSFHKRYARAIILSRVMFEVSLIILTLHSCRNFGAVKYKTEIIFTYLATASSTILILLALYKTNRFTELFLNFKAFYRNRNLDVDHLEKWNRKQKMATVIIVLFCVIKFSTLIYTDLIGEYSTPCRGYFTEYLFYTNLFMCNARYLFEFSTACVVLHLVSEQLDYIAISMDCTMFLYIDISKKNIMSSAKKRKLKYFDIFKQFEKWTDAYMNVKRSANLCDTVFRAQLAIMITTITLYYIILLYGITSFNIERGKFSVVKSLSYLISLFGFLIALLLLSKAGQRIQKSAENLRRKLSKFLLHSLEDPEFHRAATNLLRLVCTHHIKMRCFGFIDIDMTLLPSCLMFVTSYTVIALQFNNVV-

>BmorGR13

MEDSFNRLLSIRNMIIFQNVCGFYHMCTEKLYISRIIKMYCVALAIVLSVFCFQNPDITYLSWDVVWVTFGYTLNVIICLRYNGNYFFQYWNGLHEIDIKMNLTSIDKEKVPISRAVFTVFLILRSTAFAMTIFVFGYLETGILSNTIISIYSINLTEFYRNMSNIPMILMFETFYVRIKILKEQLCSELSTVLGCNNDARQLKLILKYLRNYRSLVRHLMDTTLPFKILILVILVGSFLRSLLIGYAFVYNSDQIILLSLPVMFSTKILSEVVEIKLICTKELLKNKNEGLVLLDLDSKKPTFLTSKACGEQLQDALSFLNNRSYSYTLLQVIEFDCSLAFVFTSFCITHLIVVVQFTHVLD-

>BmorGR14

MNLHKNIIPIRNNLFANKVTAIALPKTLSVLFKLIHIFFLLDLGVYEYKTFKIKCIVKFLTISGSLTISVVCFSFMVSNLSEHTFVGWYGFFISTYIFVVLFFNLSNRMTFVEFYKTLLRFDANYGIDSNEYKFNFKIIFVNILFIANRMVLSFVYCSYYPQNCIRPRYAQILFMLPWLTLDVLLTTNMFLFYATYCRIAKFPMLIKNSMNIVALRNSYKLIVDSLEKTQTSFDIVFIIALVFSVPEIMMSIYSTLLEVISKHFLEVASILSLNYVAIAQSLLLTLAPSLCAGVLPWKTNNIKIILHEKLFTEKDKASAREIELFIKYIESRPLKLRACNLVPLDFSLTIIVLNICVTYLIVIIQFTHLY-

>BmorGR15

MISSSDINHKRNKVFAYNVPGIALSKTLTVLFKLLHYVLLLDVGIYEYKTFKNKCIVKFLTIATGVSVSIVYFCLIATVLRKNAFFYWFYVLFISQYMIIVFIFTLSNGMSFTDYYKMLLRFDAKYQINSNNYYFNIKIILVIIISILNRIGMAIIYCSYYTKNCYEMSFSQIIFVLPWLTRDVILIMNVFLFYVTYCRITKFPALLENTKNVGSLRNSYKLIVDSLEKTQKPFDFVFTISLVFNIPEIMLSIYFTLLQVIHSHFLEVAPTLSISYFSITHSVVLILAPSLCAGVLPWKTNTIKIVLHDKLFLEKDKNSARNIKLFIKYIEARPLKLRACNLVPLDFSLPVIVLNLCVTYLIVIVQFSHLS-

>BmorGR16

MIMNLTTDRISKRNKVFAYNVPEVTLPTTLKVLFKLIQFTLSLDFGVYKYKTFKMKCVAKVLTLAGCLAASAACVSLIISNIFENQLFFGWYTLFVCQYTIVIFMFTFSNGMTFIDYKMMLLRFDAKYQIDSNVYHFNIKIVLVVVISVTSRLFLCAVYCIYSTENCIKPWYNQLLFFPWLSLDIVLIMNMFLFYATYCRLAKFPSLFENPKNVVPLRNSYKLIVDSLEKTKKSFDAVLIAALIFNIPEIMMSIYYTLFQVMNKHFQEVAPVLSLSYFTIILSVLLILAPSLCAGVLPWKTRHMRLILLEKLFAEKDKNSAREIELFIKYIEARPLQLRACNLVPLDFNLPVIVLNLCITYLIVIIQFTHLF-

>BmorGR17

MGFSLGTTALSMFFFEKPVVFTIIQITMIIVKPAKYKLSDPFRPKDTSKLSESIIMYFKLFHIFLGIDLGGFRYQNRQVKYAVRLISLIQPLAIYGLCIYALLKIIANTEFLWYTISFTEYVAMSVAITLFSNEMTYCNFMINLKFIDTKLKIGDESFRIGVKLISSTILIGVTRCFTTTTYCLLGFCAKPTAAQILFQIPWLTIDLMLLQYMFIFYACYCRLVKILRILKKRNTDIEEMRRIYKTLVDVLDRARAPFDLAYLLGLLFSIPDVLYSIYESIIKVGEINTAKALSMSIIYITNIQSLALMFAPALTAGFLPSLTMKMRIILHDKLLEEQDKKTYRHIVLFIKYIETCPLKLKACQIIPLDFSFPIIILNIVVTYLIVAIQLTHFL-

>BmorGR18

MRRSTKVISMVNQSDKGEIKTCSRFMKIYFFVIYILTGFNFGFYTGRGLNFLRVIQASVLLLRFIIASNCIYIAFHFRLLEAIWYSLTFSESLAIVVCFMLSRSALSCKNLFEYLYSVDQELKKSVGPSIEVKLALYTVVVSVLRLTVYVFCAIAYYETLHEGFCVELVYNTPCYCSDLYLVIHFTIFHSVYCRLKALRISMNEKFDVYKGTLIYKSLIDNLEEIKKSLDVPFFVILLNAVAIAMINILVTLEISYGQTMKFIRTAPRYLETVLLFSSAFAPVLAADMMASEAQKIKVTLNNILQRDDSLLEDDRRKVKQFAGYVSARPFRLRACRVLSLDCTLPVTVLSICVTYLIVVVQFTHLY-

>BmorGR22like

MNRHDHRFSIYNPKRNEAMWKRELFVNNEGKDIKDFQIKDIYGPEITDKDGALLDKHDSFYLNTKSLLVLFQIMGVMPIMRVPKSIRTTYNWISKATLWAYLVWGLECIIVVKVGQERLANFQIGSNKRFDEVIYNIIFLSILIPHFLLPIASWRHGPQVAIFKNMWTHYQLKYLKITGKPIVFPNLYILTWGLCIFSWVLSFAVVLSQHYLQDDFELWHSFAYYHIIAMLDGFCSLWYINCNAFGTASRGLAINLHKALEAEHPALKLAQYRHLWVDLSHMMQQLGRAYSNMYGIYCMVIFFTTTISLYGALSEILEHGLSYKEMGLFVIVAYCMTLLFIICNEAYHASRKVGHEFQDRLLNVNLGAIDRSTQREVEMFLVAIAKNPPIMNLDGFTNINRELFTANISFMSTYLIVLMQFKLTLLRQGARKTVTAIVRAIFNTTITDNGAGGSDEDQE-

>BmorGR26

MNKTKIYRKKLDKNERLVCSVQPAMFARLIVGLYYDIKVSNRVKWMIKSYCISLSSFICYLIIFRDDNFSLHPKLTSVMEYITYVTFSFLTCDKYLFRYLRFNPRTDGYPIFLYLCKKFEKFFKIIICLFVSFKILGVVLMMQSWPILSTPKYIWGTLALHFLWLASHMGRLVFILVYGILFCRMRTIRIIFENRGFQNTPQNRLTPKRYILMYEAVLNSIESVDFPVKFLIFTFICCFAPKLVVSLFEIMEEMKKGELSLTTFIWFLVELSPSYLFLLLSAIALDLVSEDVQELLSITIDRRLNCKNEKERSEIQEFFQYLRNNPFNYTLWQVVSLNLRTLLVATSFSIANVIAIMQIKNSKI-

>BmorGR27

MVFKYKIMTKAPKSLPVLKILMLFRLVFGNYFRLSSNRYINFLVKSYCSTFTILLSVMCGKRLKNDSPYMLSLTEYILNKILNYATSEGYIFKYCNSIKTCDKIMGFKKLPIITIDVFIAIIITVITRTAITIYFGFLFPFDKYQVVLYVGCIVFSNDLNSLTIMNVFGLLNNRMNLLRKSLEAMTVPINIIGKNEVAPKVRLVRNAFRYYSNLLDNLDSVNHCVQYSLSVTLLLKFPKAVLLCYDSIKTYFVKIDNNFAMDIVDPTEIILSIVVMSFPAMLCEMITNEVEKIKAILTKHLIQCSDNSLRFELNITLLYICHRPFKYILWRAIPLDTSVPIGIVSLIITYVIVLIQLLHFST-

>BmorGR29

MYLRSKKSRFKLFSFERMIKILLMICGHYVQTDSSNVVSSIHRIFSIVITICLCPYFQFNPFFFHVIESVWYSILSQFTQYGFFFRYCSTIKTFDLLSGFKQIPLYTKRVCFFLLITLLVRLIIVLIHFSAHQTKLKTFCAFLIILSANTGHILMTIMFSILNTRMTLIQKLFANNPIPVNIVGKNQNASHIKRVRKGLICYNNLLDTLKVAEKEIQFTLTVTYLCHVPTIICYVYFVITVIYKSKFSGYNLIPMLDMILACMAVTAPALFAELTKNTVDKIKKILGSQLLRCSDESLRYELEITLEYVIQRPFSFSIWRAVSLDASLPVAMTSLCITYVIVILQLTQLRP-

>BmorGR30

MYLRSKKSRFKLFSFERMIKILLMICGHYVQTDSSNVVSSIHRIFSIVITICLCPYFQFNPFFFHVIESVLYSILSQFTQYGFFFRYCSTIKTFDLLSGFKQIPLYTKRVCFFLLITLLVRLIIVLIHFSAHQTKLKTFCAFLIILSANTGHILMTIMFSILNTRMTLIQKLFANNPIPVNIVGKNQNASHIKRVRKGLICYNNLLDTLKVAEKEIQFTLTVTYLCHVPKIICYVYFVITVIYKSKFSGYNLVPLFDMILACMAVTAPAVFAELTKNTVDKIKKILGSQLLRCSDESLRYELEITLEYVIQRPFSFSIWRAVSLDASLPVAMTSLCITYVIVILQLTQLRP-

>BmorGR33

MCYTNFVSRQVSKCIHFFSTIRYIIYLRMFCGLYYNCSSSFKIRCIARLYCFIIYCLNLHYNSYIFTTNVSLTNFFHTFIILAEVSVHILFSLYTGESNFISFCIEMNKLTSDPNEFIATKCVTTHFIAYLVIVSHILSSTLICGARASCFTFSVILTSMTFLTTLLSRFTTIIMFDVVWIRMRSLRKILVNALESDLAENEKAKSIESFLNAYKQIIASTRITKLATRNLVIFNFVSMFGRIMTLIYFCINNPGYLDTYHMSLWIFGILLAGFVTCAPPVLVEMNVNELDEIKYALADQLVDYTDDNYRTAIYNALDYVEVHSIRYTLWKNFPMDLTMFFGFAGFCATYIIGLLQFTY-

>BmorGR45

MKSPEYLSKDILDEDFVRVFSFPFLVQMALGSCRVHLKARFITVPTLGQKLYTVMCIIICSLMYFNMTKLYLPLYYEHSIVYYIFVTVTGLDQLSFFANLIHLRFLNGETNTAFYIMMQRIDRNMKIDHNNIFNKTVTLANILTITLIILHYVGLVISTIILKEYSLLSLFGLLYGQLMLMVEMALCSNLIIFFFMRVRFVNAIIKNHVHPENQNQPPKLVRYFITNRITRYLAAQTHDFIVNDTDVYLKQIFEGFSMFIDIYRFQVCPLCIKLVVLTLLNFEFCLVAIQRNVLGPNHIGNYYIIVNSVMGFFTALYVSGRCELFFREIRETKRLSVAVLLQYQEGPLREKATRMLKIIEESTPQFSIYDMWQMDGYTFVKICSLVTNLIVTLLQFAYL-

>BmorGR46

PFLVQMALGSCRVHLKARFITIPTLGQKLYTVMSIIICSLLYFNITKLYLPLYYQHSIVYYLFLAVTGLDQLSFFANLIHVRFLNGETNTAFCIMMQRIDRNMKIDHNNILNKTVIRANIFTITFIILIYVVLVISTIMLNEYSLVTLFGLLYGQLIFMVERAHCSNLILFFFTRVRFVNAIIKNHVHPENQNQPPKLVRYFVTNRITRYLAAQTHDFIVNDTDVYLKQIFEGFSMFTDIYRFQVCLFCIKIVVLSLLTFELCFVAVQRNLLETKNLTNY-

>BmorGR47

MKIRFLFGFYCDFPFNKRFQNILKFYCISVLVVLILGSWACSTGFRSDKKIVIYCEYIAYFLISLSTKDRYIFDYYKQQPLIDGSTTSKVLYKKLERLLKYFVTITIVLKMLNIFVFCGWNLTKCINELDGVLFINLLWIGLLLARLSLPVIYGLLYFRLRVLRMTLESKGFSNSPQNRFTPKKYITIYEKIMKDLLKMDYPLKYVFIIFLIGSVPKLLQNSWQFLNSLKNYGPEISKILEFTLECLHSYIVIILPIVVALDLSEDEIKKMKIITLNKRLACLNERQKMEIQQLFLLLKNNSLRYNLWRVVPVNLKSVLIFLSF-

>BmorGR50

MAGIRTISSKVKPLELPDVSENNFADDGLKIVQPFKFFIYIQAITGINRLYLLKCNKFVLMFSYLYAIFLISFVALVYWTTEPKKNSHLVIRLFTFFEYTLLACISVFLKKKKMIKFFENLSLLDKMLKINKNVNSTCCMKQVFFWVTGSIVYNLIEFYAMEFYDNTNKGLKTIICTYAIALAHDCEQIFFFTLQRVVYLRLLVVKRHIQEYFKVDEDSSRKKPNKYEMLSNNVQLNLTALHEVYALLHNCAEKLNTVMSIPVLLMLFTSGLSTTILLKFFVRVIQLTDPSNPGSAIGVCMYLIVRCIKYTLLVVISCYYSSITATQVSLIRITIHDAINTVPLGKLQRRKVKAFYLMTKEYSFVYALAGVIKLNMSLPLSYISLCTTYLVIIIQFSKFLD-

>BmorGR51

MAMGIRTILSKVKPLELPDVSENNFADDGLKIVQRFKFFIYIQVLTGINRLYLLKCNKFVMLFSYLYAIFLISFVASVYWTKEPMKNSHLVIRLFSFIEYILLICISVFLKKKKMMKFFENLSMFDQILKIDKNVNSTFCMKRVFFWVTGSIVYNLIEFYALEFYDNTSKGLMTIICTYTIALTHDCEQIFFFTLQRVVYLRLLVVKRHIQEHFKVDEDSNRKKPNKYEMLSKNVQLNLTALHEVYGLLHNCAEKLNKIMSIPVLLMLFTSGLTTTILLRILVRVIQLADPSNPGSAIGLCVYLIVRCIKYTLLVVISCYYSSITATQVSLIRITINDAINTIAFGKLQRRKVKAFYLMTKEYSFVYTLAGVIKLNMSLPLSYISLCTTYLVIIIQFSKFFD-

>BmorGR53

MAHIKDENQSKQQQKEHETLNKNKLKKVVYTLKPALMLENWFGLSDFLLVNEDELVLLMQTEKFGVILSIFFIVMFAVFVDFPDTETESIMELMDEVPSMVVLSQYFIASITTSSCLSAIAIRIFETFADLDSMLLITTTQDFYNKSRYQTNKYLIILGVSHIISSTLDLLTDDEIVWCKFFVLPIYFLQKLEVLTFCKLIVMIQCRLQIINKYLTNFIEEQEKNKALVFTLAESNPKKTDKFNWIGCPSPNNMKIRDLATMYDVIGTICSLINDLFNIQIFMTLVSTFTYIVIAIWSTLYFYRAPNFTFGTLTTIIIWCITIILSVVVMSFVCERLVSVRNNTKILVNKVIMNYDLPKTMRVQAKAFMELIESWPLKIMVYDMFSVDISLMLKFISVATTYLIVIIQLSHFV-

>BmorGR58

MSSRRVLYRAEVLLSNNVDAHVQDMLKPLNFFQFILFFPKYTIRDGYITPNSLIRNIWSATGAFVFISICVFRILTMNKIAVYDTFTTMLLISKYFDVALYCIGFIVNTYVNIAYSNVNVLLYLKLQTIKTFIPRNNEIMKNVKWYSVILIIVLFCGTLAMFSFFHLSFSYFNIFDLTTDLAVFSFDLNLVYACSVLNFLAQSLDELNKEIWRLGNAKVTVCKDGSKPDWNGINLTYINVLDAYNYFKEAFRLLIFFHTFKTLTHMFIYIQSIIELCKKFYPGDDYDAITVGAVVGVWFFRNITLQCLVGVSCQNFYSATSNTESICAVQVGSIVSDEHKLFLKAVRRLNNVVFYKWSMYGMFIVDATLPRRLIELIATYTVVFLQFAFK-

>BmorGR56

MKKIRLLRSIVFLENLLCIYRNFLFFNKKARAIILIHITIELVLYVLSIVNNSFIIYSYFHSDNRSMLIVFTTICCFYVVTFVSIVMGILRSEEFKDLVTSLELINKFFTNNKTYLKSLGRSNTMIIAITTILYCVTCIGIAVDKITLNDFYEFTSSDVIWTVSSTLLELRYQTECVVYFGIEYLFLIFTKHLNLLVKEAIKKVSLDNNGTVKDVPISSDAVTKNEVKRWATIYRQLMMSSKLLQACFSLQIICVFVSAVINFITTAFRMVKVSVLGSIATDMNEIIIVNLIFTLLYQNIGLVLIIVTGQRVWNQILLLNVLLARLYNGILIQPCRDTLRTLKNLQRMVVKNPVQIKMLSVLPVGSYMLPMFMTLSVSYIIVMLQFGHVV-

>BmorGR57

MEEIKAIKLVTFIENCICVYRNYAMCTKRNKKIISLRIIVEIIIVFFVNINNILLLHKYYNGSGLLYIIYLFLVVYYINYMFCIFYGALQGKAYRQLIFCFNKINAIAKRDKSYKKSLARLKNMCIVISIALLIISALSVFVDRSNSWNIYEVSLRDSLLILSKIHMDFFYHFEYVVYFTHIKIFHLTLRYLNSRVKMAQFEMKMTRRDVHDEGERNIRILLTKELTTEWAVLYKCLVFGTKTMKSLFGLQMLIAMVMSFVNFTLSLYGIILICSIEQSQTASQHNLLLILTYYTATMLLIFIVAQSVYNEVEMLKRNLARMYNILAVDSDETQQKLVKDFLRMVYKNKVEIKMLSIFPVGMPMLTFFLSLSASYVVVMVQFSNVF-

>BmorGR60

MLTPRSDLCNEKLSPSFPSGKTTAADKDDTEARCQVDSSLERLLLPFNLVQHVSFIPMYSIRRGLVSPDGPLAYLYSLLGFCLFTSVSVYRNAIMHGTRLSSLHLFTLYSDLVSFVINYSLSLICNVVNSKSNVEFVCRLQRLQTVLRRNQREQEQFARSNWAHLAVVTALYLAVVGLLNVVVLKQSLPDTLYLLLLFCIDVNVLYATRMLALLRCYLQLWTRKINEKAFNPVHHNMFTAYLDILQEYEVYTTLFKKIITYYVLETFLHGLLYVQVAIQICKSIRRSGRFSEQLMMIVSIFTWTIKNMIIMTLHNVECEKFYLAVEQAVAACQTQRASTTRCREEKRLYKNVCRVSRAAFSRERGWGLLAAGAALTLRFMDLATTYVTVLLQFAFVSRT-

>BmorGR61

MSIRFEKDLLHNYVEIELQYFLRPFNVMQSLFFQSKYRIVDNFILPNTLFKNIMSFVVSVLCALSFIYTIISVWQNTHATSFHALVTSVYLSYNIYGILIGSVLIIWLSDRNIEFVLKIQDLIKILEFNKCFLIEYAFINSIIMAAIFILNFLLYGYFVVHLQKFALGLTFSAIVCILNQDLDIIYVIIFANILKKCASRWTVEARQKNNFNDQGKWVKLFNAFLNLTESYQLYQKIFEFYELLRRVGIVFLGLQLTVCRVCSNDIKSIQCTVMLHAFQLICVWIVKKFITLSILSFEMEIFYEKLREIETVCIILVSSDNPSERELKIWKNIIRVSSCSVRKTTACGLCEVGAALPQWLLQATTAYTIVLLQFHITTFSRATNDIYDLD-

>BmorGR62

MNDLFLSKIVKWTKTTKYKLDDDFQSLFRVFNIAQAMNLCPKFLIYDKYITNNAWFIHILAISSFIVLVCLDSFFANFRLVLSEAMGPPFYGFSFYFISILYENIGVIIQITMNGYLTKNNVLIITKLQDTFKDFRTTDYITKSNRWTNWFIFFIYMNFIANYSYFNFYVNTFSFHKFCFAFIKMCFDLNIVYTIFIFKMIGDSLTMFKDTAFCSKNMKLYEVSNRVYWNKMLRLYSNILDVFELSKRTLNFFIFYFVSNILLRILSHVQLAILMNSINWLQHVAYSNIVMVLLTLAKEGIILIVLIAKCEKIYCVIGDVQTACQLALGNAACPEKRRFCKNVRRSSSAAFSKIYICNILAVDAKLAVSLMSVTTTYTIVMLQAILIK-

>BmorGR63

MQIGNAVIHLKSTKLTTMNTISPTTKLLKIFALNSNIEEIDLKCSTKLRITMTAFVLCSLIFYSLYYKFIYVFDYVNISIKITDCVQMVYDFCQYIVDLYFVTNYGRNISSEYFQQYKIIDKILEVVCYEIIKHRIVKLLWVFMCIWFSSSCFDFIAWFLNYGWITPLVYSVAYIFLLIKILTTLDLSAHIMNVEIRLKMIADLIHHYYMSCEDNFQAEETLCHKNWLNSKERAKYYELQFRIHALKQLSCNNNEIKLLSRCYLMLTEQVEIINRMYGFRILLNSLSLLIDMVRFTNISVRIMIGSQNLAYNCGYFPAVSSIFRLLTCGAVIINLVSHCERVYYQRTRICNVIDHMIVNKNLSRESTEALQEFRNLVQNHPIEFNMANFFQLNYSLLVSIASVVVTYTIILLQSVN-

>BmorGR64

MKISLRKIVSIRNMTLIQNMFGFYHKFTDNRAIGVLLKIFCGFYSLFLSFLCINCTPRFTNDFLTYDIFFFVIEYLTSVLVCLLYDGQYFLNYLYDLKLIDREAGIEESLEKLPISQPLFSLIFITRVIYLLSCLLMFDGIKDSLFLPAQSSVFGANFTEFARTIGYFPRVIMFEMFYKRVNYLKSQLRNDLAHANLYPIGFVCSKVIMKYINFYKLLLRNLQQNSLQFKILMSMSSLYIIIKALASAYAFIYREDGVHVFIFIEFATGVFLFFVMSSIIISIFNEIEDIRQIVLAQLRYCKQGANTKRVQDALTILNIRCFKYALCRIYTVDFTFILRILDVSVTYVIVLVQFTHILD-

>BmorGR66

MKRKLKKFFPNKEYNNIVEATHLWKLIRKLTGLSVLTLESKEGNRIETRFSSLGFVFFLLWFTIYFYCTYKAHNEDQTILRNIYSTKLQRYGDDFERITSIIYVLYSMWKLPFQISGNRLLLQEIVDIDKAIESVGVTIDYKKNATFALFIYIGQIATYLFRLFCVWGCLGNLNSPVPVEKLYQDIFTDALSLLLTSQYCFSLVILRDRCRYINKILCGIENRESSRLRLFVYSSMPGAEKDITCRKIKDCSKIYGMIYKAVESTNITYGFALVLTMLLYLIFIILYMFYFMEATAAGLFLDTKKYIDFLICVLSELLHAMLIIFLNIYFSEETVKETRTTSFV-

>BmorGR67

MRERKKKFNKLLNTRNYNNIVEALLPSDSIRKISGVSVVYLAVNSENRIVTKFSFIGTIFFLFWYILYFYCTYKAHSEDQTILRTIYNTKLKRYGDDFERIASIIYVTYSMWKVPFRMSGNQVFIQRIVDIDSAIENMGEAVDYNKNAKTALVISIAQLGDFLVRMFCIWLSLENLSVIVPTEKLYQVVYTDALSFVITSHYCFSLIVLRGRYKYINKVLSEIKTRSAWEYKVFVRNKVAPDLEKVQRLQDRIVCEKIKACARIYSMLYKATEAINRMYGTALVLTMLLYLVFIILYMFYFMEATASGLLYDIKKYVDFLICVFWQMSHALSIIYANVYFSESITREVCKF-

>BmorGR68

MRFGLKAGAAVVTILRPYNLCLKNIFKPFYVMLSLLGLFPYSIRFLGGKQFLIKPKSIYTNAVCALSLMLSMTLFLIFHIDHIIYKSTEDNSLTEGFMTQVNYIIEMLNLEIFCVVYYFSSFLNRNKFVKVLNTVAVWSDRISISGIKTLSFLRLKIHFSIGILMFLLISQVCVNFTRVDSLWKKVLVMFTFNIPQMIQFTAILFYYILVNMVITLLVIIQENISISTRDTKTSSFIRVEHRMPLSLKQLELIYIKAFELKRDINKAFEAPILLTTMQCFHSIVSESHIIYHGAVMEPHMVLHSIMNCSVWILYQLFKLYILASTGHLLQEKIQHFSNLIHFHGKGLTVYGLFPLDGTLMFKVVASAAMYLIILVQFDKRN-

>CfloGR1

MTENNYAFWKGLTAYIIVLYCTFSWNFMDLFLILVSVALVDQFKQLNHRLQAVKGKGMSEWWWTTARADYNCLTDIIEQIDSKISSVVLLSFSVNLYFICIQLLHSFTLKSTPLHTIYFTFSFGFLILRASMVSLFAAKIHEESNLPSSVVLQESQIYLIIAKHGKTVLYSNALTMYC

>CfloGR2

MEVWSDEPKKNKTTTTRPRNDRLEAESKVGPNSEKIKSDVFHTLAPIYHMSKIFGLLPVKFTKDSGGRYRGRLQGSQIIYGIVVLLGYMGAQCYGLYRDLRNGWQNSTRLSSETAITVTCSDVLAVISVVTSAIIGSSFRWRHLQAALNMIVDVDEKLGIMTSKNLRRGSIIVISCTFVYLVTISLLDYISWSYSFDKKNVQVYDDKGPINYSPIYFMYIVIMTFEMQYALVLFNVGERFLKLNKTIENLTKTNLIIEYFRKDMGLTAPSDPRKNLFQAISFVSSDIGHSRLRRTNKVSDFIVSSEGNGGTSEAIDQLITLHGTLCDCIIRINKAYGGTILIGTISCLIHLIITPYFLYNEIYLDNSSNWYVLALQVFWIIFHVYRLLLFVQPCYMVSVKAKITGALVSQALAQNWDPKAKKQLEIFSLQLLHRPVEFTACGLFFLDRGLVTSIVGAVTTYLVILVQFQNADDTKGTKHLLQNATELLKNASSFKNISVFKNTS

>CfloGR3

MLVYAANTFLTLTMYTYYFIKPVIIGSTTLPATILVYCFVQIISSTVSLVVLTKIANSTAVESKKTGEIIAEGMVNLHNPQVIDQLNKFLNYLNHKTLHFGVSNFFTLDGSLLMSIIGSITTYLVILLQFQENSDQKRKNEEIVHNAVLLRMNRKINEWI

>CfloGR4

MSELRFRATLAESPQHGRYETFKMDSIPIDASRSSASNSGANLIESTRSFHCALKPIVTLAQCFAVFPVDGVQSSDASSLKFTWKSFKVLYCCLSATGSIVLTMFSVYRLATTSITSSKTSNLVFFFTAGITVLLFLKLSRQWPSFAVSWENMERELAARHNSRRLNAISLILKFKILSAVVMILALVEHTLSILSGYVSAIECAHIRGHSDIVATYFSLQFPQIFTETNYANWKGVIVQYINILSTFSWSFMDLFLILISVALTDQFKQLNHRLYSIRGKHCFVVKAMPEWWWAEARVDFNRLAAMTRRVDDKISDIVMLSFSTNLYFICIQLLNSFKPLPNAIQTVYFCFSFGFLLSRTVAVSLYAASVHDESLLPAPILYSVCSASYSAEVRRFLNQVTTDHVSLTGMKFFSITRSLILTVAGTIVTYELILVQFNAVQSDHQHSVSNITKICESPDSSITDVKIEERYFCFLIAEHILSILSGYARSVQCARNHGNIDVANVYFILKFPELFTKHNYALWKAFIIKVVNIISTFSWTFIDLFLILMSVSLTDQFKYFNRRLYTTFDKIMPEDWWTEARKDFNYLATITRKVDSEISSIVLLSFSANLYFICIQLLNSFNPMPNVIHRIYFCFSFGFLLLRTVAVSLYAANIHEESLLPASVLYNVETTSFSIEVERFLSQVTTDHISLTGMKFFSVTRSLILTVVGTIVTYELVLMQFNSIQDDQQENSSNMTKVCNLK

>CfloGR5

MSILSNLSWNFVDVFIIAISMALTDQFRQLNRRIDFFKEKVWPFPSTIHTCYFFFSFGFLISRVLTVSMSAASIHDESLLPAYTLYSISSKSYSSEIASTIITYVIVLTQIYSNQIEKLKPNITTSENLEGVLPVTELTTRIE

>CfloGR6

MMLLFGNSKNNASRKRSINPIIFPKLHSLKRTSPQLFRRQSPDDSDDPECFHRAIGGILLLAQFFGILPIYYVRADTVEKMSFRKYTPRNFYAYFVFIGVCFMTVVSIVHMITTLGTISFQTRGGIADATVGAIFYGNSLLGNILFLRLCPRWITMQYDWRAMERVIDNRGKWSRPRLRARFKIIAGAILGLALLEHVLSVINNTPNELWGGNHTFEDWLDVYTNRSHAFILKHVDYNFTFGLFILFVSRVATFTWNFTDLFVILVATGIADRYKHLNKRIMRATPRELTFMDWHEIRECYAVLSALVKKVDSEISGIVLLSFINNIYFICLQLMNGLTPSESEHSIVNSLYFFGSFFFLIGRSVCVTLMTARINDECKIILPVLYNCPSVNYSLEAQRLQHQIASDDIALTGHRFFSITRNFMLAVAGAIVTYEIVLLQFNIAMQ

>DmelGR5a

MRQLKGRNRCNRAVRHLKVQGKMWLKNLKSGLEQIRESQVRGTRKNFLHDGSFHEAVAPVLAVAQCFCLMPVCGISAPTYRGLSFNRRSWRFWYSSLYLCSTSVDLAFSIRRVAHSVLDVRSVEPIVFHVSILIASWQFLNLAQLWPGLMRHWAAVERRLPGYTCCLQRARPARRLKLVAFVLLVVSLMEHLLSIISVVYYDFCPRRSDPVESYLLGASAQLFEVFPYSNWLAWLGKIQNVLLTFGWSYMDIFLMMLGMGLSEMLARLNRSLEQQVRQPMPEAYWTWSRTLYRSIVELIREVDDAVSGIMLISFGSNLYFICLQLLKSINTMPSSAHAVYFYFSLLFLLSRSTAVLLFVSAINDQAREPLRLLRLVPLKGYHPEVFRFAAELASDQVALTGLKFFNVTRKLFLAMAGTVATYELVLIQFHEDKKTWDCSPFNLD-

>DmelGR8a

MSGHLGRVLQFHLRLYQVLGFHGLPLPGDGNPARTRRRLMAWSLFLLISLSALVLACLFSGEEFLYRGDMFGCANDALKYVFAELGVLAIYLETLSSQRHLANFWWLHFKLGGQKTGLVSLRSEFQQFCRYLIFLYAMMAAEVAIHLGLWQFQALTQHMLLFWSTYEPLVWLTYLRNLQFVLHLELLREQLTGLEREMGLLAEYSRFASETGRSFPGFESFLRRRLVQKQRIYSHVYDMLKCFQGAFNFSILAVLLTINIRIAVDCYFMYYSIYNNVINNDYYLIVPALLEIPAFIYASQSCMVVVPRIAHQLHNIVTDSGCCSCPDLSLQIQNFSLQLLHQPIRIDCLGLTILDCSLLTRMACSVGTYMIYSIQFIPKFSNTYM-

>DmelGR9a

MSLWLEHFLTGYFQLCGLVCGWSGSRLGRLLSSTFLVLILIELVGEIETYFTEENPDNESVPAYFAKVIMGVNMAYKMIHAWIALSALFECRRFRYLLEELPPVKATSFIYRHLILEIILFACNAFLVLSEYTIRGIYLENLRYAYSLQAVRARYLQMMVLVDRLDGKLEQLHHRVISGSSDYKTLRLDYAHLAKVTRSLSHLFGLSLLLLNVLCLGDWIIVCNVYFMVAYLQVLPATLFLFGQVMFVVCPTLIKIWSICAASHRCVSKSKHLQQQLKDLPGQTPVERSQIEGFALQIMQDPIQIDVCGIYHLNLQTLAGMFFFILEALVIFLQFVSLVRT-

>DmelGR10a

MTSPDERKSFWERHEFKFYRYGHVYALIYGQVVIDYVPQRALKRGVKVLLIAYGHLFSMLLIVVLPGYFCYHFRTLTDTLDRRLQLLFYVSFTNTAIKYATVIVTYVANTVHFEAINQRCTMQRTHLEFEFKNAPQEPKRPFEFFMYFKFCLINLMMMIQVCGIFAQYGEVGKGSVSQVRVHFAIYAFVLWNYTENMADYCYFINGSVLKYYRQFNLQLGSLRDEMDGLRPGGMLLHHCCELSDRLEELRRRCREIHDLQRESFRMHQFQLIGLMLSTLINNLTNFYTLFHMLAKQSLEEVSYPVVVGSVYATGFYIDTYIVALINEHIKLELEAVALTMRRFAEPREMDERLTREIEHLSLELLNYQPPMLCGLLHLDRRLVYLIAVTAFSYFITLVQFDLYLRKKS-

>DmelGR10b

MRVGKLCRLALRFWMGLILVLGFSSHYYNPTRRRLVYSRILQTYDWLLMVINLGAFYLYYRYAMTYFLEGMFRRQGFVNQVSTCNVFQQLLMAVTGTWLHFLFERHVCQTYNELSRILKHDLKLKEHSRFYCLAFLAKVYNFFHNFNFALSAIMHWGLRPFNVWDLLANLYFVYNSLARDAILVAYVLLLLNLSEALRLNGQQEHDTYSDLMKQLRRRERLLRIGRRVHRMFAWLVAIALIYLVFFNTATIYLGYTMFIQKHDALGLRGRGLKMLLTVVSFLVILWDVVLLQVICEKLLAEENKICDCPEDVASSRTTYRQWEMSALRRAITRSSPENNVLGMFRMDMRCAFALISCSLSYGIIIIQIGYIPG-

>DmelGR22d

MFRPRCGLRQKFVYVILKSILYSSWLLGIFPFKYEPKKRRLRRSMWLILFGVVISSSLLILMVKQSAEDREHGIMLDVFQRNALLYQISSLMGVVGVVSICTVHLRTLWRSKHLEEIYNGLMLLEAKYFCSNAVECPAFDGYVIQKGVVIVVGLLAPWMVHFGMPDSKLPVLNVLVVSMVKLGTLLLALHYHLGVVIIYRFVWLINRELLSLVCSLRGNHKGSSSRVRFLLKLYNKLVNLYSKLADCYDCQTVLMMAIFLAANIIVCFYMIVYRISLSKMSFFVMLIMFPLAIANNFMDFWLSMKVCDLLQKTGRQTSMILKLFNDIENMDKDLEISISDFALYCSHRRFKFLHCGLFHVNREMGFKMFVASVLYLLYLVQFDYMNL-

>DmelGR21a_isoform_A

MSFWAVSRGLTPPSKVVPMLNPNQRQFLEDEVRYREKLKLMARGDAMEEVYVRKQETVDDPLELDKHDSFYQTTKSLLVLFQIMGVMPIHRNPPEKNLPRTGYSWGSKQVMWAIFIYSCQTTIVVLVLRERVKKFVTSPDKRFDEAIYNVIFISLLFTNFLLPVASWRHGPQVAIFKNMWTNYQYKFFKTTGSPIVFPNLYPLTWSLCVFSWLLSIAINLSQYFLQPDFRLWYTFAYYPIIAMLNCFCSLWYINCNAFGTASRALSDALQTTIRGEKPAQKLTEYRHLWVDLSHMMQQLGRAYSNMYGMYCLVIFFTTIIATYGSISEIIDHGATYKEVGLFVIVFYCMGLLYIICNEAHYASRKVGLDFQTKLLNINLTAVDAATQKEVEMLLVAINKNPPIMNLDGYANINRELITTNISFMATYLVVLLQFKITEQRRIGQQQA-

>DmelGR22b

MFGSSREIRPYLARQMLKTTLYGSWLLGIFPFTLDSGKRIRQLRRSRCLTLYGLVLNYFLIFTLIRLAFEYRKHKLEAFKRNPVLEMINVVIGIINVLSALIVHFMNFWGSRKVGEICNELLILEYQDFEGLNGRNCPNFNCFVIQKCLTILGQLLSFFTLNFALPGLEFHICLVLLSCLMEFSLNLNIMHYHVGVLLIYRYVWLINEQLKDLVSQLKLNPETDFSRIHQFLSLYKRLLELNRKLVIAYEYQMTLFIIAQLSGNIVVIYFLIVYGLSMRTYSIFLVAFPNSLLINIWDFWLCIAACDLTEKAGDETAIILKIFSDLEHRDDKLEMSVNEFAWLCSHRKFRFQLCGLFSMNCRMGFKMIITTFLYLVYLVQFDYMNL-

>DmelGR22f

MKMFQPRRGFSCHLAWFMLQTTLYASWLLGLFPFTFDSRRKQLKRSRWLLLYGFVLHSLAMCLAMSSHLASKQRRKYNAFERNPLLEKIYMQFQVTTFFTISVLLLMNVWKSNTVRKIANELLTLEGQVKDLLTLKNCPNFNCFVIKKHVAAIGQFVISIYFCLCQENSYPKILKILCCLPSVGLQLIIMHFHTEIILVYRYVWLVNETLEDSHHLSSSRIHALASLYDRLLKLSELVVACNDLQLILMLIIYLIGNTVQIFFLIVLGVSMNKRYIYLVASPQLIINFWDFWLNIVVCDLAGKCGDQTSKVLKLFTDLEHDDEELERSLNEFAWLCTHRKFRFQLCGLFSINHNMGFQMIITSFLYLVYLLQFDFMNL-

>DmelGR22e

MFRPSGSGYRQKWTGLTLKGALYGSWILGVFPFAYDSWTRTLRRSKWLIAYGFVLNAAFILLVVTNDTESETPLRMEVFHRNALAEQINGIHDIQSLSMVSIMLLRSFWKSGDIERTLNELEDLQHRYFRNYSLEECISFDRFVLYKGFSVVLELVSMLVLELGMSPNYSAQFFIGLGSLCLMLLAVLLGASHFHLAVVFVYRYVWIVNRELLKLVNKMAIGETVESERMDLLLYLYHRLLDLGQRLASIYDYQMVMVMVSFLIANVLGIYFFIIYSISLNKSLDFKILVFVQALVINMLDFWLNVEICELAERTGRQTSTILKLFNDIENIDEKLERSITDFALFCSHRRLRFHHCGLFYVNYEMGFRMAITSFLYLLFLIQFDYWNL-

>DmelGR22a

MSQPKRIHRICKGLARFTIRATLYGSWVLGLFPFTFDSRKRRLNRSKWLLAYGLVLNLTLLVLSMLPSTDDHNSVKVEVFQRNPLVKQVEELVEVISLITTLVTHLRTFSRSSELVEILNELLVLDKNHFSKLMLSECHTFNRYVIEKGLVIILEIGSSLVLYFGIPNSKIVVYEAVCIYIVQLEVLMVVMHFHLAVIYIYRYLWIINGQLLDMASRLRRGDSVDPDRIQLLLWLYSRLLDLNHRLTAIYDIQVTLFMATLFSVNIIVGHVLVICWINITRFSLLVIFLLFPQALIINFWDLWQGIAFCDLAESTGKKTSMILKLFNDMENMDQETERRVTEFTLFCSHRRLKVCHLGLLDINYEMGFRMIITNILYVVFLVQFDYMNLKFKTD-

>DmelGR28a

MAFKLWERFSQADNVFQALRPLTFISLLGLAPFRLNLNPRKEVQTSKFSFFAGIVHFLFFVLCFGISVKEGDSIIGYFFQTNITRFSDGTLRLTGILAMSTIFGFAMFKRQRLVSIIQNNIVVDEIFVRLGMKLDYRRILLSSFLISLGMLLFNVIYLCVSYSLLVSATISPSFVTFTTFALPHINISLMVFKFLCTTDLARSRFSMLNEILQDILDAHIEQLSALELSPMHSVVNHRRYSHRLRNLISTPMKRYSVTSVIRLNPEYAIKQVSNIHNLLCDICQTIEEYFTYPLLGIIAISFLFILFDDFYILEAILNPKRLDVFEADEFFAFFLMQLIWYIVIIVLIVEGSSRTILHSSYTAAIVHKILNITDDPELRDRLFRLSLQLSHRKVLFTAAGLFRLDRTLIFTITGAATCYLIILIQFRFTHHMDDTSSNSTNNLHSIHLGD-

>DmelGR28b_isoform_A

MIRCGLDIFRGCRGRFRYWLSARDCYDSISLMVAIAFALGITPFLVRRNALGENSLEQSWYGFLNAIFRWLLLAYCYSYINLRNESLIGYFMRNHVSQISTRVHDVGGIIAAVFTFILPLLLRKYFLKSVKNMVQVDTQLERLRSPVNFNTVVGQVVLVILAVVLLDTVLLTTGLVCLAKMEVYASWQLTFIFVYELLAISITICMFCLMTRTVQRRITCLHKVLKNLAHQWDTRSLKAVNQKQRSLQCLDSFSMYTIVTKDPAEIIQESMEIHHLICEAAATANKYFTYQLLTIISIAFLIIVFDAYYVLETLLGKSKRESKFKTVEFVTFFSCQMILYLIAIISIVEGSNRAIKKSEKTGGIVHSLLNKTKSAEVKEKLQQFSMQLMHLKINFTAAGLFNIDRTLYFTISGALTTYLIILLQFTSNSPNNGYGNGSSCCETFNNMTNHTL-

>DmelGR32a

MSPNTWVIEMPTQKTRSHPYPRRISPYRPPVLNRDAFSRDAPPMPARNHDHPVFEDIRTILSVLKASGLMPIYEQVSDYEVGPPTKTNEFYSFFVRGVVHALTIFNVYSLFTPISAQLFFSYRETDNVNQWIELLLCILTYTLTVFVCAHNTTSMLRIMNEILQLDEEVRRQFGANLSQNFGFLVKFLVGITACQAYIIVLKIYAVQGEITPTSYILLAFYGIQNGLTATYIVFASALLRIVYIRFHFINQLLNGYTYGQQHRRKEGGARARRQRGDVNPNVNPALMEHFPEDSLFIYRMHNKLLRIYKGINDCCNLILVSFLGYSFYTVTTNCYNLFVQITGKGMVSPNILQWCFAWLCLHVSLLALLSRSCGLTTTEANATSQILARVYAKSKEYQNIIDKFLTKSIKQEVQFTAYGFFAIDNSTLFKIFSAVTTYLVILIQFKQLEDSKVEDPVPEQT-

>DmelGR33a

MIQIMNWFSMVIGLIPLNRQQSETNFILDYAMMCIVPIFYVACYLLINLSHIIGLCLLDSCNSVCKLSSHLFMHLGAFLYLTITLLSLYRRKEFFQQFDARLNDIDAVIQKCQRVAEMDKVKVTAVKHSVAYHFTWLFLFCVFTFALYYDVRSLYLTFGNLAFIPFMVSSFPYLAGSIIQGEFIYHVSVISQRFEQINMLLEKINQEARHRHAPLTVFDIESEGKKERKTVTPITVMDGRTTTGFGNENKFAGEMKRQEGQQKNDDDDLDTSNDEDEDDFDYDNATIAENTGNTSEANLPDLFKLHDKILALSVITNGEFGPQCVPYMAACFVVSIFGIFLETKVNFIVGGKSRLLDYMTYLYVIWSFTTMMVAYIVLRLCCNANNHSKQSAMIVHEIMQKKPAFMLSNDLFYNKMKSFTLQFLHWEGFFQFNGVGLFALDYTFIFSTVSAATSYLIVLLQFDMTAILRNEGLMS-

>DmelGR36a

MFDWVGLLLKVLYYYGQIIGLINFEIDWQRGRVVAAQRGILFAIAINVLICMVLLLQISKKFNLDVYFGRANQLHQYVIIVMVSLRMASGISAILNRWRQRAQLMRLVECVLRLFLKKPHVKQMSRWAILVKFSVGVVSNFLQMAISMESLDRLGFNEFVGMASDFWMSAIINMAISQHYLVILFVRAYYHLLKTEVRQAIHESQMLSEIYPRRAAFMTKCCYLADRIDNIAKLQNQLQSIVTQLNQVFGIQGIMVYGGYYIFSVATTYITYSLAINGIEELHLSVRAAALVFSWFLFYYTSAILNLFVMLKLFDDHKEMERILEERTLFTSALDVRLEQSFESIQLQLIRNPLKIEVLDIFTITRSSSAAMIGSIITNSIFLIQYDMEYF-

>DmelGR36b

MVDWVVLLLKAVHIYCYLIGLSNFEFDCRTGRVFKSRRCTIYAFMANIFILITIIYNFTAHGDTNLLFQSANKLHEYVIIIMSGLKIVAGLITVLNRWLQRGQMMQLVKDVIRLYMINPQLKSMIRWGILLKAFISFAIELLQVTLSVDALDRQGTAEMMGLLVKLCVSFIMNLAISQHFLVILLIRAQYRIMNAKLRMVIEESRRLSFLQLRNGAFMTRCCYLSDQLEDIGEVQSQLQSMVGQLDEVFGMQGLMAYSEYYLSIVGTSYMSYSIYKYGPHNLKLSAKTSIIVCILITLFYLDALVNCNNMLRVLDHHKDFLGLLEERTVFASSLDIRLEESFESLQLQLARNPLKINVMGMFPITRGSTAAMCASVIVNSIFLIQFDMEFF-

>DmelGR36c

MDLESFLLGAVYYYGLFIGLSNFEFDWNTGRVFTKKWSTLYAIALDSCIFALYIYHWTGNTNIVNAIFGRANMLHEYVVAILTGLRIVTGLFTLILRWYQRCKMMDLASKVVRMYVARPQVRRMSRWGILTKFIFGSITDGLQMAMVLSAMGSVDSQFYLGLGLQYWMFVILNMAMMQQHMIMLFVRTQFQLINTELRQVIDEAKDLLLSPRHQGVFMTKCCSLADQIENIARIQSQLQTIMNQMEEVFGIQGAMTYGGYYLSSVGTCYLAYSILKHGYENLSMTLSTVILAYSWCFFYYLDGMLNLSVMLHVQDDYWEMLQILGKRTIFVGLDVRLEEAFENLNLQLIRNPLKITVVKLYDVTRSNTMAMFGNLITHSIFLIQYDIEHF-

>DmelGR39a_isoform_A

MSKVCRDLRIYLRLLHIMGMMCWHFDSDHCQLVATSGSERYAVVYAGCILVSTTAGFIFALLHPSRFHIAIYNQTGNFYEAVIFRSTCVVLFLVYVILYAWRHRYRDLVQHILRLNRRCASSCTNQQFLHNIILYGMLTILCFGNYLHGYTRAGLATLPLALCMLVYIFAFLVLCLLLMFFVSLKQVMTAGLIHYNQQLCQGDLISGLRGRQQILKLCGGELNECFGLLMLPIVALVLLMAPSGPFFLISTVLEGKFRPDECLIMLLTSSTWDTPWMIMLVLMLRTNGISEEANKTAKMLTKVPRTGTGLDRMIEKFLLKNLRQKPILTAYGFFALDKSTLFKLFTAIFTYMVILVQFKEMENSTKSINKF-

>DmelGR39b

MLYSFHPYLKYFALLGLVPWSESCAQSKFVQKVYSAILIILNAVHFGISIYFPQSAELFLSLMVNVIVFVARIVCVTVIILQVMVHYDDYFRFCREMKYLGLRLQCELKIHVGRLKWQSYAKILALGIGFLVTVLPSIYVALSGSLLYFWSSLLSILIIRMQFVLVLLNVELLGHHVSLLGIRLQNVLECHLMGANCTLDGNANRLCSLEFLLALKQSHMQLHYLFTHFNDLFGWSILGTYVVLFSDSTVNIYWTQQVLVEVYEYKYLYATFSVFVPSFFNILVFCRCGEFCQRQSVLIGSYLRNLSCHPSIGRETSYKDLLMEFILQVEQNVLAINAEGFMSTDNSLLMSILAAKVTYLIVLMQFSSV-

>DmelGR43a_isoform_A

MEISQPSIGIFYISKVLALAPYATVRNSKGRVEIGRSWLFTVYSATLTVVMVFLTYRGLLFDANSEIPVRMKSATSKVVTALDVSVVVMAIVSGVYCGLFSLNDTLELNDRLNKIDNTLNAYNNFRRDRWRALGMAAVSLLAISILVGLDVGTWMRIAQDMNIAQSDTELNVHWYIPFYSLYFILTGLQVNIANTAYGLGRRFGRLNRMLSSSFLAENNATSAIKPQKVSTVKNVSVNRPAMPSALHASLTKLNGETLPSEAAAKNKGLLLKSLADSHESLGKCVHLLSNSFGIAVLFILVSCLLHLVATAYFLFLELLSKRDNGYLWVQMLWICFHFLRLLMVVEPCHLAARESRKTIQIVCEIERKVHEPILAEAVKKFWQQLLVVDADFSACGLCRVNRTILTSFASAIATYLVILIQFQRTNG-

>DmelGR47a

MAFTSSQLCSLLTKFTALNGLNTYYFDTKTNAFRVSSKLKIYCAIHHALCVLALAHMSYSTASNLRVSVTVLTIGGTMACCVKSCWEKAQGIRNLARGLVTMEQKYFAGRPSGLLLKCRYYIKITFGSITLLRIHLIQPIYMRRLLPSQFYLNVGAYWLLYNMLLAAVLGFYFLLWEMCRIQKLINDQMTLILARSGQRNRLKKMQHCLRLYSKLLLLCDQFNSQLGHVAIWVLACKSWCQITFGYEIFQMVAAPKSIDLTMSMRVFVIFTYIFDAMNLFLGTDISELFSTFRADSQRILRETSRLDRLLSMFALKLALHPKRVVLLNVFTFDRKLTLTLLAKSTLYTICCLQNDYNKLKA-

>DmelGR47b

MQRDDGFVYCYGNLYSLLLYWGLVTIRVRSPDRGGAFSNRWTVCYALFTRSFMVICFMATVMTKLRDPEMSAAMFGHLSPLVKAIFTWECLSCSVTYIEYCLSLDLQKDRHLKLVARMQEFDRSVLMVFPHVQWNYRRARLKYWYGTVIVGFCFFSFSISLIFDTTRCTCGIPSTLLMAFTYTLLTSSVGLLGFVHIGIMDFIRVRLRLVQQLLHQLYQADDSSEVHERIAYLFEMSKRCSFLLAELNGVFGFAAAAGIFYDFTIMTCFVYVICQKLLEREPWDPEYVYMLLHVAIHTYKVVITSTYGYLLLREKRNCMHLLSQYSRYFSGQDVARRKTEDFQHWRMHNRQAAMVGSTTLLSVSTIYLVYNGMANYVIILVQLLFQQQQIKDHQLTSGKDVDIVGPMGPITHMD-

>DmelGR57a

MAVLYFFREPETVFDCAAFICILQFLMGCNGFGIRRSTFRISWASRIYSMSVAIAAFCCLFGSLSVLLAEEDIRERLAKADNLVLSISALELLMSTLVFGVTVISLQVFARRHLGIYQRLAALDARLMSDFGANLNYRKMLRKNIAVLGIVTTIYLMAINSAAVQVASGHRALFLLFALCYTIVTGGPHFTGYVHMTLAEMLGIRFRLLQQLLQPEFLNWRFPQLHVQELRIRQVVSMIQELHYLIQEINRVYALSLWAAMAHDLAMSTSELYILFGQSVGIGQQNEEENGSCYRMLGYLALVMIPPLYKLLIAPFYCDRTIYEARRCLRLVEKLDDWFPQKSSLRPLVESLMSWRIQAKIQFTSGLDVVLSRKVIGLFTSILVNYLLILIQFAMTQKMGEQIEQQKIALQEWIGF-

>DmelGR58a

MLLKFMYIYGIGCGLMPAPLKKGQFLLGYKQRWYLIYTACLHGGLLTVLPFTFPHYMYDDSYMSSNPVLKWTFNLTNITRIMAMFSGVLLMWFRRKRILNLGENLILHCLKCKTLDNRSKKYSKLRKRVRNVLFQMLLVANLSILLGALILFRIHSVQRISKTAMIVAHITQFIYVVFMMTGICVILLVLHWQSERLQIALKDLCSFLNHEERNSLTLSENKANRSLGKLAKLFKLFAENQRLVREVFRTFDLPIALLLLKMFVTNVNLVYHGVQFGNDTIETSSYTRIVGQWVVISHYWSAVLLMNVVDDVTRRSDLKMGDLLREFSHLELVKRDFHLQLELFSDHLRCHPSTYKVCGLFIFNKQTSLAYFFYVLVQVLVLVQFDLKNKVEKRN-

>DmelGR58b

MLHPKLGRVMNVVYYHSVVFALMSTTLRIRSCRKCLRLEKVSRTYTIYSFFVGIFLFLNLYFMVPRIMEDGYMKYNIVLQWNFFVMLFLRAIAVVSCYGTLWLKRHKIIQLYKYSLIYWKRFGHITRAIVDKKELLDLQESLARIMIRKIILLYSAFLCSTVLQYQLLSVINPQIFLAFCARLTHFLHFLCVKMGFFGVLVLLNHQFLVIHLAINALHGRKARKKWKALRSVAAMHLKTLRLARRIFDMFDIANATVFINMFMTAINILYHAVQYSNSSIKSNGWGILFGNGLIVFNFWGTMALMEMLDSVVTSCNNTGQQLRQLSDLPKVGPKMQRELDVFTMQLRQNRLVYKICGIVELDKPACLSYIGSILSNVIILMQFDLRRQRQPINDRQYLIHLMKNKTKV-

>DmelGR58c

MNQYFLLHTYFQVSRLIGLCNLHYDSSNHRFILNHVPTVVYCVILNVVYLLVLPFALFVLTGNIYHCPDAGMFGVVYNVVALTKLLTMLFLMSSVWIQRRRLYKLGNDLMKMLHKFRFNLGNDCRNRCLCKGLLTSSRFVLLTQQLLTRDSVVNCESNSSLRQAMVPYQSAAIVYALIMILLMSYVDMTVYMVEVAGNWLLVNMTQGVREMVQDLEVLPERNGIPREMGLMQILAAWRKLWRRCRRLDALLKQFVDIFQWQVLFNLLTTYIFSIAVLFRLWIYLEFDKNFHLWKGILYAIIFLTHHVEIVMQFSIFEINRCKWLGLLEDVGNLWDINYSGRQCIKSSGTILSRKLEFSLLYMNRKLQLNPKRVRRLHIVGLFDLSNLTVHNMTRSIITNVLVLCQIAYKKYG-

>DmelGR59a

MKRIGQAYNVYAVFIGMTSYETMGGKFRQSRITRIYCLLINAIFLTLLPSAFWKSAKLLSTADWMPSYMRVTPYIMCTINYAAIAYTLISRCYRDAMLMDLQRIVLEVNREMLRTGKKMNSLLRRMFFLKTFTLTYSCLSYILAVFIYQWKAQNWSNLCNGLLVNISLTILFVNTFFYFTSLWHIARGYDFVNQQLNEIVACQSMDLERKSKELRGLWALHRNLSYTARRINKHYGPQMLAMRFDYFIFSIINACIGTIYSTTDQEPSLEKIFGSLIYWVRSFDFFLNDYICDLVSEYQMQPKFFAPESSMSNELSSYLIYESSTRLDLLVCGLYRVNKRKWLQMVGSIVVHSSMLFQFHLVMRGGL-

>DmelGR59b

MVYWMIKLYFRYSLAIGITSQQFSNRKFFSTLFSRTYALIANIVTLIMLPIVMWQVQLVFQQKKTFPKLILITNNVREAVSFLVILYTVLSRGFRDTAFKEMQPLLLTLFREEKRCGFKGIGGVRRSLRILLFVKFFTLSWLCVTDVLFLLYSTDALIWVNVLRFFFKCNTNNILEMVPMGYFLALWHIARGFDCVNRRLDQIVKSKSTRKHRELQHLWLLHACLTKTALNINKIYAPQMLASRFDNFVNGVIQAYWGAVFTFDLSTPFFWVVYGSVQYHVRCLDYYLIDNMCDVAVEYHDSAKHSWSEVRWTKEISSYVIYANSTKLQLWSCGLFQANRSMWFAMISSVLYYILVLLQFHLVMRK-

>DmelGR59c

MVDLVKTILLIAYWYGLAVGVSNFEVDWLTGEAIATRRTTIYAAVHNASLITLLILFNLGNNSLKSEFISARYLHEYFFMLMTAVRISAVLLSLITRWYQRSRFIRIWNQILALVRDRPQVVRGRWYRRSIILKFVFCVLSDSLHTISDVSAQRKRITADLIVKLSLLATLTTIFNMIVCQYYLAMVQVIGLYKILLQDLRCLVRQAECICSIRNRRGGVYSIQCCSLADQLDLIAERHYFLKDRLDEMSDLFQIQSLSMSLVYFFSTMGSIYFSVCSILYSSTGFGSTYWGLLLIVLSTASFYMDNWLSVNIGFHIRDQQDELFRVLADRTLFYRELDNRLEAAFENFQLQLASNRHEFYVMGLFKMERGRLIAMLSSVITHTMVLVQWEIQNDES-

>DmelGR59d

MADLLKLCLRIAYAYGRLTGVINFKIDLKTGQALVTRGATLISVSTHLLIFALLLYQTMRKSVVNVMWKYANSLHEYVFLVIAGFRVVCVFLELVSRWSQRRTFVRLFNSFRRLYQRNPDIIQYCRRSIVSKFFCVTMTETLHIIVTLAMMRNRLSIALALRIWAVLSLTAIINVIITQYYVATACVRGRYALLNKDLQAIVTESQSLVPNGGGVFVTKCCYLADRLERIAKSQSDLQELVENLSTAYEGEVVCLVITYYLNMLGTSYLLFSISKYGNFGNNLLVIITLCGIVYFVFYVVDCWINAFNVFYLLDAHDKMVKLLNKRTLFQPGLDHRLEMVFENFALNLVRNPLKLHMYGLFEFGRGTSFAVFNSLLTHSLLLIQYDVQNF-

>DmelGR59e

MDSSYWENLLLTINRFLGVYPSGRVGVLRWLHTLWSLFLLMYIWTGSIVKCLEFTVEIPTIEKLLYLMEFPGNMATIAILVYYAVLNRPLAHGAELQIERIITGLKGKAKRLVYKRHGQRTLHLMATTLVFHGLCVLVDVVNYDFEFWTTWSSNSVYNLPGLMMSLGVLQYAQPVHFLWLVMDQMRMCLKELKLLQRPPQGSTKLDACYESAFAVLVDAGGGSALMIEEMRYTCNLIEQVHSQFLLRFGLYLVLNLLNSLVSICVELYLIFNFFETPLWEESVLLVYRLLWLAMHGGRIWFILSVNEQILEQKCNLCQLLNELEVCSSRLQRTINRFLLQLQRSIDQPLEACGIVTLDTRSLGGFIGVLMAIVIFLIQIGLGNKSLMGVALNRSNWVYV-

>DmelGR59f

MRSSATKGAKLKNSPRERLSSFNPQYAERYKELYRTLFWLLLISVLANTAPITILPGCPNRFYRLVHLSWMILWYGLFVLGSYWEFVLVTTQRVSLDRYLNAIESAIYVVHIFSIMLLTWQCRNWAPKLMTNIVTSDLNRAYTIDCNRTKRFIRLQLFLVGIFACLAIFFNIWTHKFVVYRSILSINSYVMPNIISSISFAQYYLLLQGIAWRQRRLTEGLERELTHLHSPRISEVQKIRMHHANLIDFTKAVNRTFQYSILLLFVGCFLNFNLVLFLVYQGIENPSMADFTKWVCMLLWLAMHVGKVCSILHFNQSIQNEHSTCLTLLSRVSYARKDIQDTITHFIIQMRTNVRQHVVCGVINLDLKFLTTLLVASADFFIFLLQYDVTYEALSKSVQGNVTRYK-

>DmelGR61a_isoform_A

MSRTSDDIRKHLKVRRQKQRAILAMRWRCAQGGLEFEQLDTFYGAIRPYLCVAQFFGIMPLSNIRSRDPQDVKFKVRSIGLAVTGLFLLLGGMKTLVGANILFTEGLNAKNIVGLVFLIVGMVNWLNFVGFARSWSHIMLPWSSVDILMLFPPYKRGKRSLRSKVNVLALSVVVLAVGDHMLYYASGYCSYSMHILQCHTNHSRITFGLYLEKEFSDIMFIMPFNIFSMCYGFWLNGAFTFLWNFMDIFIVMTSIGLAQRFQQFAARVGALEGRHVPEALWYDIRRDHIRLCELASLVEASMSNIVFVSCANNVYVICNQALAIFTKLRHPINYVYFWYSLIFLLARTSLVFMTASKIHDASLLPLRSLYLVPSDGWTQEVQRFADQLTSEFVGLSGYRLFCLTRKSLFGMLATLVTYELMLLQIDAKSHKGLRCA-

>DmelGR63a

MANYYRRKKGDAVFLNAKPLNSANAQAYLYGVRKYSIGLAERLDADYEAPPLDRKKSSDSTASNNPEFKPSVFYRNIDPINWFLRIIGVLPIVRHGPARAKFEMNSASFIYSVVFFVLLACYVGYVANNRIHIVRSLSGPFEEAVIAYLFLVNILPIMIIPILWYEARKIAKLFNDWDDFEVLYYQISGHSLPLKLRQKAVYIAIVLPILSVLSVVITHVTMSDLNINQVVPYCILDNLTAMLGAWWFLICEAMSITAHLLAERFQKALKHIGPAAMVADYRVLWLRLSKLTRDTGNALCYTFVFMSLYLFFIITLSIYGLMSQLSEGFGIKDIGLTITALWNIGLLFYICDEAHYASVNVRTNFQKKLLMVELNWMNSDAQTEINMFLRATEMNPSTINCGGFFDVNRTLFKGLLTTMVTYLVVLLQFQISIPTDKGDSEGANNITVVDFVMDSLDNDMSLMGASTLSTTTVGTTLPPPIMKLKGRKG-

>DmelGR64a

MKGPNLNFRKTPSKDNGVKQVESLARPETPPPKFVEDSNLEFNVLASEKLPNYTNLDLFHRAVFPFMFLAQCVAIMPLVGIRESNPRRVRFAYKSIPMFVTLIFMIATSILFLSMFTHLLKIGITAKNFVGLVFFGCVLSAYVVFIRLAKKWPAVVRIWTRTEIPFTKPPYEIPKRNLSRRVQLAALAIIGLSLGEHALYQVSAILSYTRRIQMCANITTVPSFNNYMQTNYDYVFQLLPYSPIIAVLILLINGACTFVWNYMDLFIMMISKGLSYRFEQITTRIRKLEHEEVCESVFIQIREHYVKMCELLEFVDSAMSSLILLSCVNNLYFVCYQLLNVFNKLRWPINYIYFWYSLLYLIGRTAFVFLTAADINEESKRGLGVLRRVSSRSWCVEVERLIFQMTTQTVALSGKKFYFLTRRLLFGMAGTIVTYELVLLQFDEPNRRKGLQPLCA-

>DmelGR64b

MPQGETFHRAVSNVLFISQIYGLLPVSNVRALDVADIRFRWCSPRILYSLLIGILNLSEFGAVINYVIKVTINFHTSSTLSLYIVCLLEHLFFWRLAIQWPRIMRTWHGVEQLFLRVPYRFYGEYRIKRRIYIVFTIVMSSALVEHCLLLGNSFHLSNMERTQCKINVTYFESIYKWERPHLYMILPYHFWMLPILEWVNQTIAYPRSFTDCFIMCIGIGLAARFHQLYRRIAAVHRKVMPAVFWTEVREHYLALKRLVHLLDAAIAPLVLLAFGNNMSFICFQLFNSFKNIGVDFLVMLAFWYSLGFAVVRTLLTIFVASSINDYERKIVTALRDVPSRAWSIEVQRFSEQLGNDTTALSGSGFFYLTRSLVLAMGTTIITYELMISDVINQGSIRQKTQYCREY-

>DmelGR64c_isoform_A

MQQSGQKGTRNTLQHAIGPVLVIAQFFGVLPVAGVWPSCRPERVRFRWISLSLLAALILFVFSIVDCALSSKVVFDHGLKIYTIGSLSFSVICIFCFGVFLLLSRRWPYIIRRTAECEQIFLEPEYDCSYGRGYSSRLRLWGVCMLVAALCEHSTYVGSALYNNHLAIVECKLDANFWQNYFQRERQQLFLIMHFTAWWIPFIEWTTLSMTFVWNFVDIFLILICRGMQMRFQQMHWRIRQHVRQQMPNEFWQRIRCDLLDLSDLLGIYDKELSGLIVLSCAHNMYFVCVQIYHSFQSKGNYADELYFWFCLSYVIIRVLNMMFAASSIPQEAKEISYTLYEIPTEFWCVELRRLNEIFLSDHFALSGKGYFLLTRRLIFAMAATLMVYELVLINQMAGSEVQKSFCEGGVGSSKSIFS-

>DmelGR64d_isoform_A

MLSTKIVLNDGLQLYTMGSLSFSVICIFCFGSFIKLSRRWPHIIRETALCERIFLKPCYANQEGLNFTRFLRRWALILLVAALCEHLTYVGSAAWSNYVQIRDCNLKVGFVENYFLRERQELFSVFEYRAWMVFFIEWNTMAMTFVWNFGDIFLFLMCRGLKIRFQQLHWRIRQNLGKPMAKEFWQEIRSDFLDLDSLLKLYDKELSGLILVCCAHNMYFICVQVYHSFQVKGAFMDELYFWFCLLYVISRLMNMMLAASSIPQEIKDISNTLYEVRSSPWCDELGRLSEMLRNETFALSGMGYFYVTRRLIFAMAGALMGYELVLFRQMQGAVVQKSICSRGPGSSMSIFFS-

>DmelGR64e_isoform_A

MARTTGDPAKRRRCMSRIKFWRRSRVGSEVVEKDTKRFKLSLIKAWLLRIRQEDYKYSGSFQEAIKPVLIIAQIFALMPVRKVSSKFAEDLTFTWFSVRSYYALVTILFFGVSSGYMVAFVTSVSFNFDSVETLVFYLSIFLISLSFFQLARKWPEIAQSWQLVEAKLPPLKLPKERRSLAQHINMITIVATTCSLVEHIMSMLSMGYYVNSCPRWPDRPIDSFLYLSFSSVFYFVDYTRFLGIVGKVVNVLSTFAWNFNDIFVMAVSVALAARFRQLNDYMMREARLPTTVDYWMQCRINFRNLCKLCEEVDDAISTITLLCFSNNLYFICGKILKSMQAKPSIWHALYFWFSLVYLLGRTLILSLYSSSINDESKRPLVIFRLVPREYWCDELKRFSEEVQMDNVALTGMKFFRLTRGVVISVAGTIVTYELILLQFNGEEKVPGCFEN-

>DmelGR64f

MKILPKLERKLRRLKKRVTRTSLFRKLDLVHESARKKAFQESCETYKNQIENEYEIRNSLPKLSRSDKEAFLSDGSFHQAVGRVLLVAEFFAMMPVKGVTGKHPSDLSFSWRNIRTCFSLLFIASSLANFGLSLFKVLNNPISFNSIKPIIFRGSVLLVLIVALNLARQWPQLMMYWHTVEKDLPQYKTQLTKWKMGHTISMVMLLGMMLSFAEHILSMVSAINYASFCNRTADPIQNYFLRTNDEIFFVTSYSTTLALWGKFQNVFSTFIWNYMDLFVMIVSIGLASKFRQLNDDLRNFKGMNMAPSYWSERRIQYRNICILCDKMDDAISLITMVSFSNNLYFICVQLLRSLNTMPSVAHAVYFYFSLIFLIGRTLAVSLYSSSVHDESRLTLRYLRCVPKESWCPEVKRFTEEVISDEVALTGMKFFHLTRKLVLSVAGTIVTYELVLIQFHEDNDLWDCDQSYYS-

>DmelGR66a

MAQAEDAVQPLLQQFQQLFFISKIAGILPQDLEKFRSRNLLEKSRNGMIYMLSTLILYVVLYNILIYSFGEEDRSLKASQSTLTFVIGLFLTYIGLIMMVSDQLTALRNQGRIGELYERIRLVDERLYKEGCVMDNSTIGRRIRIMLIMTVIFELSILVSTYVKLVDYSQWMSLLWIVSAIPTFINTLDKIWFAVSLYALKERFEAINATLEELVDTHEKHKLWLRGNQEVPPPLDSSQPPQYDSNLEYLYKELGGMDIGSIGKSSVSGSGKNKVAPVAHSMNSFGEAIDAASRKPPPPPLATNMVHESELGNAAKVEEKLNNLCQVHDEICEIGKALNELWSYPILSLMAYGFLIFTAQLYFLYCATQYQSIPSLFRSAKNPFITVIVLSYTSGKCVYLIYLSWKTSQASKRTGISLHKCGVVADDNLLYEIVNHLSLKLLNHSVDFSACGFFTLDMETLYGVSGGITSYLIILIQFNLAAQQAKEAIQTFNSLNDTAGLVGAATDMDNISSTLRDFVTTTMTPAV-

>DmelGR68a

MKIYQDIYPISKPSQIFAILPFYSGDVDDGFRFGGLGRWYGRLVALIILIGSLTLGEDVLFASKEYRLVASAQGDTEEINRTIETLLCIISYTMVVLSSVQNASRHFRTLHDIAKIDEYLLANGFRETYSCRNLTILVTSAAGGVLAVAFYYIHYRSGIGAKRQIILLLIYFLQLLYSTLLALYLRTLMMNLAQRIGFLNQKLDTFNLQDCGHMENWRELSNLIEVLCKFRYITENINCVAGVSLLFYFGFSFYTVTNQSYLAFATLTAGSLSSKTEVADTIGLSCIWVLAETITMIVICSACDGLASEVNGTAQILARIYGKSKQFQNLIDKFLTKSIKQDLQFTAYGFFSIDNSTLFKIFSAVTTYLVILIQFKQLEDSKVEDISQA-

>DmelGR77a

MPLPLGDPLALAVSPQLGYIRITAMPRWLQLPGMSALGILYSLTRVFGLMATANWSPRGIKRVRQSLYLRIHGCVMLIFVGCFSPFAFWCIFQRMAFLRQNRILLMIGFNRYVLLLVCAFMTLWIHCFKQAEIIGCLNRLLKCRRRLRRLMHTRKLKDSMDCLATKGHLLEVVVLLSSYLLSMAQPIQILKDDPEVRRNFMYACSLVFVSVCQAILQLSLGMYTMAILFLGHLVRHSNLLLAKILADAEHIFESSQKAGFWPNRQELYKGQQKWLALELWRLLHVHHQLLKLHRSICSLCAVQAVCFLGFVPLECTIHLFFTYFMKYSKFILRKYGRSFPLNYFAIAFLVGLFTNLLLVILPTYYSERRFNCTREIIKGGGLAFPSRITVKQLRHTMHFYGLYLKNVEHVFAVSACGLFKLNNAILFCIVGAILEYLMILIQFDKVLNK-

>DmelGR85a

MYSLIEAQLLGGKLVNRVMASLRRIIQRSLGYFCALNGILDFNTDIGTGNLRRYRVLFMYRLLHNFAVISLTLKFLFDFTDHFKYIESSTLITVNFFTYFTLVFFALLSSMGSCYQWQNRILAVLKELKHQRDLSRHMGYRVPRSKQNSIDYLLFALTVLLILRLSIHLATFTLSARMGFNHPCNCFLPECMIFSMNYLLFAILAEITRCWWSLQSGLKMVLLNRQLSTVAFNLWEIERLHTRFQCLIDLTSEVCSIFRYVTLAYMARNLWSGIVAGYLLVRFVIGNGLQDVELVYLVFSFITCIQPLMLSLLVNSMTSTTGSLVEVTRDILKISHKKSVNLERSIEWLSLQLTWQHTHVTIFGVFRINRSLAFRSASLILVHVLYMVQSDYISITN-

>DmelGR89a

MLRFPHVCGLCLLLKYWQILALAPFRTSEPMVARCQRWMTLIAVFRWLLLTSMAPFVLWKSAAMYEATNVRHSMVFKTIALATMTGDVCISLALLGNHLWNRRELANLVNDLARLHRRRRLSWWSTLFLWLKLLLSLYDLLCSVPFLKGAGGRLPWSQLVAYGVQLYFQHVASVYGNGIFGGILLMLECYNQLEREEPTNLARLLQKEYSWLRLIQRFVKLFQLGIFLLVLGSFVNIMVNIYAFMSYYVSLHGVPLTISNNCLVLAIQLYAVILAAHLCQVRSAKLRKKCLQLEYVPEGLTQEQAMASTPFPVLTPTGNVKFRILGVFILDNSFWLFLVSYAMNFIVVILQTSFEHINHGEI-

>DmelGR92a

MFEFLHQMSAPKLSTSILRYIFRYAQFIGVIFFCLHTRKDDKTVFIRNWLKWLNVTHRIITFTRFFWVYIASISIKTNRVLQVLHGMRLVLSIPNVAVILCYHIFRGPEIIDLINQFLRLFRQVSDLFKTKTPGFGGRRELILILLNLISFAHEQTYLWFTIRKGFSWRFLIDWWCDFYLVSATNIFIHINSIGYLSLGVLYSELNKYVYTNLRIQLQKLNTSGSKQKIRRVQNRLEKCISLYREIYHTSIMFHKLFVPLLFLALIYKVLLIALIGFNVAVEFYLNSFIFWILLGKHVLDLFLVTVSVEGAVNQFLNIGMQFGNVGDLSKFQTTLDTLFLHLRLGHFRVSILGLFDVTQMQYLQFLSALLSGLAFIAQYRMQVGNG-

>DmelGR93a

MFSSSSAMTGKRAESWSRLLLLWLYRCARGLLVLSSSLDRDKLQLKATKQGSRNRFLHILWRCIVVMIYAGLWPMLTSAVIGKRLESYADVLALAQSMSVSILAVISFVIQARGENQFREVLNRYLALYQRICLTTRLRHLFPTKFVVFFLLKLFFTLCGCFHEIIPLFENSHFDDISQMVGTGFGIYMWLGTLCVLDACFLGFLVSGILYEHMANNIIAMLKRMEPIESQDERYRMTKYRRMQLLCDFADELDECAAIYSELYHVTNSFRRILQWQILFYIYLNFINICLMLYQYILHFLNDDEVVFVSIVMAFVKLANLVLLMMCADYTVRQSEVPKKLPLDIVCSDMDERWDKSVETFLGQLQTQRLEIKVLGFFHLNNEFILLILSAIISYLFILIQFGITGGFEASEDIKNRFD-

>DmelGR93b

MSGLLVMPRILRCLNVSRISAILLRSCFLYGTFFGVITFRIERKDSQLVAINRRGYLWICLVIRLLASCFYGYSYDAWSGQYEDMYLRAFFGFRLIGCLICSVIILVMQFWFGEELINLVNRFLQLFRRMQSLTNSPKNRFGDRAEFLLMFSKVFSLLFVFMAFRLMLSPWFLLTLVCDLYTSVGTGMITHLCFVGYLSIGVLYRDLNNYVDCQLRAQLRSLNGENNSFRNNPQPTRQAISNLDKCLYLYDEIHQVSRSFQQLFDLPLFLSLAQSLLAMSMVSYHAILRRQYSFNLWGLVIKLLIDVVLLTMSVHSAVNGSRLIRRLSFENFYVTDSQSYHQKLELFLGRLQHQELRVFPLGLFEVSNELTLFFLSAMVTYLVFLVQYGMQSQQI-

>DmelGR93c

MIERLKKVSLPALSAFILFCSCHYGRILGVICFDIGQRTSDDSLVVRNRHQFKWFCLSCRLISVTAVCCFCAPYVADIEDPYERLLQCFRLSASLICGICIIVVQVCYEKELLRMIISFLRLFRRVRRLSSLKRIGFGGKREFFLLLFKFICLVYELYSEICQLWHLPDSLSLFATLCEIFLEIGSLMIIHIGFVGYLSVAALYSEVNSFARIELRRQLRSLERPVGGPVGRKQLRIVEYRVDECISVYDEIERVGRTFHRLLELPVLIILLGKIFATTILSYEVIIRPELYARKIGMWGLVVKSFADVILLTLAVHEAVSSSRMMRRLSLENFPITDHKAWHMKWEMFLSRLNFFEFRVRPLGLFEVSNEVILLFLSSMITYFTYVVQYGIQTNRL-

>DmelGR93d

MKATKYSVGILRFMSFYARFLSLVCFRLRKQKDNNVWLEEIWSNRSRWKWISVTLRIVPLCIYAFTYAEWISNRMLITEKFLHSCSLVVSIPCYLSIIHLKICHGPEVTKLVNQYLHIFRLGTLDIRRRSQFGGGRELFLLILSVCCQIHEYVFILVIASRLCGFQHIIWWVSYTYVFIICNSIMCFGFIWHLSLGVLYAELNDNLRFESGFQTAFLRKQQRIRVQKSMALFKEISSVVTSLQDIFNVHLFLSALLTLLQVLVVWYKMIIDLGFSDFRIWSFSLKNLIQTLLPVLAIQEAANQFKQTRERALDIFLVGKSKHWMKSVEIFVTHLNLSEFRVNLLGLFNVSNELFLIIVSAMFCYLVFVTQCVIVYRRRYVI-

>DmelGR94a

MDFTSDYAHRRMVKFLTIILIGFMTVFGLLANRYRAGRRERFRFSKANLAFASLWAIAFSLVYGRQIYKEYQEGQINLKDATTLYSYMNITVAVINYVSQMIISDHVAKVLSKVPFFDTLKEFRLDSRSLYISIVLALVKTVAFPLTIEVAFILQQRRQHPEMSLIWTLYRLFPLIISNFLNNCYFGAMVVVKEILYALNRRLEAQLQEVNLLQRKDQLKLYTKYYRMQRFCALADELDQLAYRYRLIYVHSGKYLTPMSLSMILSLICHLLGITVGFYSLYYAIADTLIMGKPYDGLGSLINLVFLSISLAEITLLTHLCNHLLVATRRSAVILQEMNLQHADSRYRQAVHGFTLLVTVTKYQIKPLGLYELDMRLISNVFSAVASFLLILVQADLSQRFKMQ-

>DmelGR97a_isoform_A

MRFLRRQTRRLRSIWQRSLPVRFRRGKLHTQLVTICLYATVFLNILYGVYLGRFSFRRKKFVFSKGLTIYSLFVATFFALFYIWNIYNEISTGQINLRDTIGIYCYMNVCVCLFNYVTQWEKTLQIIRFQNSVPLFKVLDSLDISAMIVWRAFIYGLLKIVFCPLITYITLILYHRRSISESQWTSVTTTKTMLPLIVSNQINNCFFGGLVLANLIFAAVNRKLHGIVKEANMLQSPVQMNLHKPYYRMRRFCELADLLDELARKYGFTASRSKNYLRFTDWSMVLSMLMNLLGITMGCYNQYLAIADHYINEEPFDLFLAIVLVVFLAVPFLELVMVARISNQTLTRRTGELLQRFDLQHADARFKQVVNAFWLQVVTINYKLMPLGLLELNTSLVNKVFSSAIGSLLILIQSDLTLRFSLK-

>DmelGR98a

MEQMSGELHAASLLYMRRLMKCLGMLPFGQNLFSKGFCYVLLFVSLGFSSYWRFSFDYEFDYDFLNDRFSSTIDLSNFVALVLGHAIIVLELLWGNCSKDVDRQLQAIHSQIKLQLGTSNSTDRVRRYCNWIYGSLIIRWLIFIVVTIYSNRALTINATYSELVFLARFSEFTLYCAVILFIYQELIVGGSNVLDELYRTRYEMWSIRRLSLQKLAKLQAIHNSLWQAIRCLECYFQLSLITLLMKFFIDTSALPYWLYLSRVEHTRVAVQHYVATVECIKLLEIVVPCYLCTRCDAMQRKFLSMFYTVTTDRRSSQLNAALRSLNLQLSQEKYKFSAGGMVDINTEMLGKFFFGMISYIVICIQFSINFRAKKMSNEQMSQNITSTSAPI-

>DmelGR98c

MEMEAKRSRLLTTARPYLQVLSLFGLTPPAEFFTRTLRKRRRFCWMAGYSLYLIAILLMVFYEFHANIVSLHLEIYKFHVEDFSKVMGRTQKFLIVAIATCNQLNILLNYGRLGLIYDEIANLDLGIDKSSKNFCGKSHWWSFRLRLTLSIGLWMVIIIGVIPRLTLGRAGPFFHWVNQVLTQIILIMLQLKGPEYCLFVLLVYELILRTRHVLEQLKDDLEDFDCGARIQELCVTLKQNQLLIGRIWRLVDEIGAYFRWSMTLLFLYNGLTILHVVNWAIIRSIDPNDCCQLNRLGSITFLSFNLLLTCFFSECCVKTYNSISYILHQIGCLPTAEEFQMLKMGLKEYILQMQHLKLLFTCGGLFDINIKLFGGMLVTLCGYVIIIVQFKIQDFALIGYRQNTSDTS-

>DmelGR98d

MEANRSRLLAAARPYIQIYSIFGLTPPIQFFTRTLHKRRRGIVILGYACYLISISLMVIYECYANIVALQKDIHKFHAEDSSKVMGNTQKVLVVAMFVWNQLNILLNFRRLARIYDDIADLEIDLNNASSGFVGQRHWWRFRFRLALSVGLWIVLLVGLTPRFTLVALGPYLHWTNKVLTEIILIMLQLKCTEYCVFVLLIYELILRGRHILQQISVELEGNQSRDSVQELCVALKRNQLLAGRIWGLVNEVSLYFTLSLTLLFLYNELTILQIVNWALIKSVNPNECCQYRRVGTCLLLSINIFLSCLYSEFCIQTYNSISRVLHQMYCLSAAEDYLILKMGLREYSLQMEHLKLIFTCGGLFDINLKFFGGMVVTLFGYIIILVQFKIQFFAQSNFMQNINSTELKAYTA-

>DmelGR98b

MVAQKSRLLARAFPYLDIFSVFALTPPPQSFGHTPHRRLRWYLMTGYVFYATAILATVFIVSYFNIIAIDEEVLEYNVSDFTRVMGNIQKSLYSIMAIANHLNMLINYRRLGGIYKDIADLEMDMDEASQCFGGQRQRFSFRFRMALCVGVWMILMVGSMPRLTMTAMGPFVSTLLKILTEFVMIMQQLKSLEYCVFVLIIYELVLRLRRTLSQLQEEFQDCEQQDMLQALCVALKRNQLLLGRIWRLEGDVGSYFTPTMLLLFLYNGLTILHMVNWAYINKFLYDSCCQYERFLVCSTLLVNLLLPCLLSQRCINAYNCFPRILHKIRCTSADPNFAMLTRGLREYSLQMEHLKLRFTCGGLFDINLKYFGGLLVTIFGYIIILIQFKVQAIAANRYKKVVN-

>MmedGR1

MLWLEITNDSSRNSAIEYSFTFRQNERPLTQRILSHIHRMKKKQFLGENSPLYTSVCPLVYIIRGFGLVPYEFEDNQLVPCDSYMIISFFWLFMYTYIVSGFIIEFIESEKNRKKVLLYAEQARTVFNFAVVISDLLLCMRTRKEITWIWNKIQDYDQAMRDLGYAKNEKSARMWVWFIIGGNTIIWAVVSSSGMNAFNEPWLHNVSFMIIYVGAAAAITKFSGLVMILGDRFKQLNEIARSSVQRSRWIHSYPIIDDKLIDCLHSELTVIGNNINKVYKFSLLLWCANLSFHSVCCGYFVLNWLLDGNFRWKYIECLTAWFVASVYQLFLIHYSCHYTSSEANCMSYIMLGWKRWLYTHDSKMEVETSIHLVNRQLHFSAAGCFYVNLPLLHSTAAILTTYMVILLQID

>MmedGR2

MIHPAGKSAQPSLIASEIKHKAVPCEPTFNTWNILKKDQVKPNRSGLMRNSDSLHVALRPVITLAQCFALFPVNGINAPDASGLSFTWGSFKILYCALTLIMSAFMTVASIIRILSTKFHTTKITTLVFSVTSCLTSLMFLKLARKWPKFAKSWEKIEGELTIRYNQPSKYSLVKRFKVVTIIIVTLAFLEHALSLASGYISARECACLLGDNDVAAIYFKTQFPQVFNKTNYALWKGIVVQCTNLLSTFSWNFMDLFLILLSTALTYHFNLLNKRLNNVKNKTMPEWWWAEARSDYNNLASLTRQVDSYVAHIVLLSFGTDLYFICIQLMYSFDRMTSVMRTIYLSYSFGFLLGRTTAVSLTAASVHDESLLPAPVLYGVNGSSYSSEVIRFLTQVTTDNIGLTGMKFFSITRSFVLTVAGTIVTYELVLIQFNNVQQVNHLNLTNVCEVK

>NvitGR1

MTAEAKPKGHRNASPRRIHFGIGRKMMSFFRSSKSGRGKPRSQHTPIFSKIHPAKRPALHSRRQSSNEDEPECFHRAIGNILLMSQFFGILPIRYIRSSSVRNFSFYKFAPRVIYSYFVLLAISVMTSISFLHLFRTLNANSFQTKGGIADATVGAMFYGNSLLGNLMFLRLCPKWISIQHDWRAMERLIDNNGKWKGPVLRWRFTLISSTILSLALLEHILSMVNNTPSDVWFGKKNLEDFLIIYTNKSHRFIVRNVDYNFTLGLFIFFISKVSTFTWNFTDLFIMLVSTGLAERYKRLNARILEATPAQLSVTDWHELRECYAVLSALVKKVDNEISGIILLSFTNNIYFICLQLLNGLSPSTAEHPIINSIYFFGSFIFLIGRTTAVTLLTARINDQCKLILPILYNCPSVNYCREAQRLQQQIATDDVALTGHRFFSITRNFMLAVAGAIVTYEVVLLQFNIALQRDEELNNMAAGSNG

>NvitGR2

MEAWHDSPTKSKERHFRKRADQLLKGQQEGSAERIKSDVYYSLSPIYHMSKFCGLLPVKFSQDKSGKYHGRLDLPQVVYGYALLAACLGAQSWGLYRDLRNGWQNSTRLSSETAITVTCSDVLAVISIIVVVEVGSPSRWKYLQDALNKIVDIDEKLDVVTSKHLRKASIVVIAVTFFYLFTISTLDFAAWTVSSSTKNIQGDTDKTPINYSPLYFMYIIIMTFEIQYALVLFNVGERFLKLNKTIENLTKTNTIIEYIRKDMGLAVTDTRRELTQSVSFLSTDMGQSKLRRSNKVSDFIVSAEGSGGSSDAINQLTTLHGMLCDIVINVNKAYGSAILMGTISCLIHLVITPYFLYNEIYSPNSSSWYVLWMQVLWIVFHVYRLLLLVQPCYMVSVKARMTGALVSQALATKWNPEATKQLEIFSIQLLQRPIEFTACGLFYLDRGLVTSIVGSVTTYLVILVQFQNADDTKGTKHLLQNATELLRNASSFKNFSSLKNL

>NvitGR3

MYKDLYNTLEPIMWVWRFVGAYPFVIRGPIGSQQYVLSTYSIMLSLIFLIVTLQYCYKSVDFINQSATEYSLFLITISVQQVSNVICFFDSVVLKLFFGKKITRSIENIAIQDKKLSKIGCSLNYSQAVRKGRLYIFILFIFLCFHIHSELVYFNGGAPLLLRIFGNYLAMLQVANVILFAWLMFNVGLRFQVINCKIKTCIFDIEWADQNSSCLLVLRTTAKAHAQLCQAAKIINGTFVLSIINCVLSAFIFTTTMLYYIFMELKNTLPFSHAVYYCSVILIHATTIVIIVQSCNWVHRNAHATVKVLHEFSKENNRFDDDKHLNQIIHNFSMQILHHNLTFSAWGLFPIDSTLLQSLAEAVTTYLVILIQFDPLVT

>NvitGR4

MQQDLYTVLESLGWLFRISGIYPFTLNDSGESRKYALNTISVTFSIVFFIVSAKYFYEYSIIIKNVYDAEEESLAVLMISVRQITSLICIFDASIMKIIYSRRFMLAINNLALQDQILKIMGYSLNYKLMFRISVLSIPCYLLVSNTLLNSEFLVVSSVDIPTAFRVFGNFSILVQMTNTILFVFLIINVGQRFKGLNFCILKLVNHEYTKLGMPISNKTPAEYMQMAAQVHARLCEIGKAINSVFYITLIVTTIGAFIFSTTMIFYIFTLAKYGFQNVGFSPVFLFFALLFIQIFTVLKIVLSCNWTSRQAAITSKILHSLSLSDMCVDDDSLSEMVRTFSMQILHHNFSFTAAGFFQIDSTLIQSFAGAVTTYLVILIQFDPKIA

>NvitGR5

MEVGSKKHRQRSTQRQKKSPGNEESPLYEVVCPAVYLARVFGLAPYELEDARPDNNRPKRLGASTVYCFFSIFWLIVYTYIVVISLIRFGGLDRDKPVLGVTEDGKLILNYLVSMVDTLLCIRCRERFVHVWNSIQDFDESFQLGNVPAGRDSPRYSPILRRARFWVWTILTTNVVGWTMINQLGMHAFGEPYLQNIGYMLTYVGTCVAVLKFVGVVMLLGQRFAYLNEELARQRKKEEGRRSRAADEIVKKIESSYNKLLSTSEELGNIYSFSLFLYLLNLFCHAVSNMYFFTIWTILDPGYLKNPKIVFCLFSWLLIYLVQMLLIHVACHFTSLEANRMASVLLDWRRQAYRQSKYEFSSTLHYLNRRLNFTAAGCFNVNLPLLTSIFGHLTTYLVILLQIPDSSNS

>NvitGR6

MKRTAKQSDSKFQVNFSLPPLRKASYAKNSSSGSSPSSSIQDIRPTFLIARVFGLAPYAITNSSINVSKRGILYSVPWLGFYLYALYNRLNLYTHSDLETKFRILSVTRTALAVIALLVDLVVCTFRDDRFQNALDCVRKYDLAVKYDVETNARLMRIHSWTIYSFMITYYLAIGWFTYVDEPYEGVMAAVIYVYLYLPLSIAVMKFVALITSILLRFRHLHRMLLPGFLSIMMELDSEPKRLHLRDVCWLHSCLCAAAANVNSLYSLQLMLWFANLTFNTISRINDFGQPQNSIDAFKLARDAGLVLIFVTLVFFIAGVCHVTSTQANKVGAVVFSPGSRYFRSRVVDHQDKEDKFYIGQYFALHPLHFSAASGFFQINLSLLLKIAGAMTTYLVILKSPSNC

>NvitGR7

MKLKFCKNLTFVQITNIYQLMRPYFFLYKLYGLFPYKISKNQIHSSKIGLCHTFFVAMSCIVYFVIAMYQCFYSLDIVFDTTESLMQFTSYFMLGTFIAVYSCASNKYKFLLLKKLILLSSMLSEKEFFEVAKVIYFKDIIGYIFLMGQIFNIASEDLTAQNISKMFALHITMIVFLMDMQYSNFVFLLKSCLKNVNNNLQLLTKSYEGCEIISCNKSMQLLQFNNLQLIKLRKLQHNHHHVSCVIKELNTVFTLQIIATVLMTFAEVTFGLYFFILHIQGKKGIDLDKQLWFNYFITSVTYYSLKMAVMVWICQETKNESLKTGIIVHDVILNNNNEQLKSELSLFSLQLLQCNNEFTSKCIVMNANLISGVVSGIATYLLILIQFLNTKKSTSKNNEQ

>NvitGR8

MFKKVKKKYSTGKKWKIFSATDFLSLIKPSLLVCRFFGLISYKILNGKIEQSKNCGSYCAIVTFVYICASLLILYIINVSPYMNRASTWMLQGNCFYTLVNFMLVSNFVFKSSTIKILQNLADTTAKLPSEKFVKISKWIHSKDLVLYLLLLLHVPKVFVGNIYAVLSKIIGTYAAMTIYLLDFQYNSYVFIIASCFEHINEELVQLNYNACKERGHLLRRVYHHQFNPLLFVKLRYLKQWHYELNEIIRKINSNFSLQVVATVIMTFTELTFGLYFYILDRRHKVRSLDKEIWYFYYHTMVMYFSTKLLLLTLTCQYANNENYKTRTIVNEIIISTDNKLFKEEIYLFSLQLLHTDNKFIAKGVQLDATLLTGMAKGIFTYLLILIQFLITN

>NvitGR9

MVNLSDIKPLFYAARFFGCAPHRVTDSDVLLTTSGLIYSGIWALGFVCCCCYGLRLICAGVYTGERNMLALTAVRTLLAYVCFLADDALTMRWNERLRSALLQLRNFDVAVSYGRKRSVNWKLRCCCWMLVGTIIAYWIGVGYVTYKCEMTNPLFNAITYVIANAAISMQLIKFAGLLILLRQRFRYLRELLPLEAAHPNSARRAIQLQDIWWLHCSLANAAETINSCYSLQLLLWIFTMWLNALSRIYAMNETLVDSGQFLLMLRESLLVTACIGNLMLIALACHYTAREANSVGRAAFAPQTSFSRKRSLLEHSLEVGVYFSLRQLHFSAAGGFIQVDLPLLLSIAGAMTTYLVVLHNNS

>NvitGR10

MKTTIINCILIKCVFYFMKLIGVCPFVLDKKEILKSSTSGKMYNLLLIVSYIYSYVIVIKCRRNLHYSEETQLGIIIDMIGITLKYSAVIVCWYTLAVHQTQVKSIIQHLKVIANNQTMLASKCRREKINNEFKTFRYGLIVINILGLTILTQNNFINNYYKNCTTDFTFTLFDIFQIVIYNVIFIFLRIVLYTQENYRIINKALNKCTNYNELDNVNVTYDTILSLKKLQSAGLAHKNISDLLENIVDFFRLPVLLIITAVFVQILIDVHLILYFIKTENWNHIKYYSLIHLLITFAIRVSATYFICSISDSTGIEGNNTKNIINVILNKWRFTKSHKNLAKMFIFNLHEHKIQISLYGLFNVDYSLLKNLCSSTIMYVIFMFQLDGIIK

>NvitGR11

MKKVNGIHIIKTLYFYEKLMGLCPFILSNKIVIKFSYIGAVYNLLITLIYTYYFILIIGLRFELHLTRESTLSIALDAFGLAFQYCSIVSAWLTLTFRQECLKKILVTFAKVNLLANNLSMTLTRYCLRKLQYIAVRLMLINLMYIVIFLSEHYLLKTYKKFEEHASTWIWFNLPKLVIYNIFGIFIELMIILQQDYRALNKVISYSFSEKIDATSFCNFSESPGVISKKLCTIAEFHENLSDILEYTTNLFSLPLLFALLASFLHLTLDSYIVYQHLLSKRMWEFNDFSSYVICLVWISTKILGFYFLCSVPDSTSAEANHTVIILIKIINNCYKVRSCRDMMKKLMLQFKQKKNYASLYGLFSLDYYLFKNIMSTSVMFLVFMFQLDDLVT

>NvitGR12

MQSRHTYTKLFVRCFLRIIGLFPLFLDINGNPIFSYLGLFFNLCLVFAYIFMSIIAFQKRMTLVLPKETVVAQIVDMIADGLENLNIISCLLVVAFRQKCLVKFYEKLKAIDLRLYDINVKHCKTFDINMNIISLGRKLTIVGISFFIICTVDHLRLLFDNYLLSIRFWIAYQSTKIVIYNLIIVFCETMIFFRKSFAKLNLLFQQSSSSYDKIQDIGQLHKILSELVDDFVGFYSFMVGSTIVHNFIHLSSNMYRIYLFLKFNGDWSLLDFLDFTSIMIWLNVKILILYFLCALPAAVSEEANKKSIFIHRMLKAVRENDLTSNNKRIARLLTILYQTNLEISVYGVFILDFKCFQSILTTSTMYIVFMIQLEHLK

>NvitGR13

MKKTLYVCVYYFVKILGLCPFTRKKGRFVKVSHVGMSYNVLITILYSRAFVKAIQNRHSIRLSQETPLAVIIDTFTHVLSYSTIVSSWLVCAFRQKIFIKVFQSFKNVENLENDLLPSMHCSKNGLEENLKEFRARFIVVNLICIIFTGSTVVIISMCEDMKNQSWFWFIYNIPINVTFNVAFILTEFMRCLRKHYKIINREASKLARSRKRSFARMPMAFSKKLQTIGRIHSDLTELGETVVKLFSLPVLMTVHGHSANIILAIHGLYRILKSDQSMGGPCVLYAPTVKFMIYSIIIFFICSIPVSTCNEADKTLRIIGQIPYEWHEEEMHNKMIKNLMFQLYQKRLQVSIFGFFNLDYSLFRNVWIVIIMYLIFVLQLDPSSFIFAILK

>NvitGR14

MVKGTKYLFDIFKVFGLATMSMTDCTKKNNFKNRKMFSYSYHGIIYNGVLICFLIIAGIYKMYYIRDKLIDQSRMSEVIDVFGNFIIYAVSVVLLSKYMISQTLAVRIGNNLYSINLVLKRFNLKYKNQYMIMHYKLVLLFDITIWLGVIIIGSFSDCTFIAAILTYIPNFIINCLVIQYVVIIIFIYGEAKALNNQLRKYVDRAFSNTLLYQFRRPVLSVHYYLPENNEIILLQKSCLSIYEVSNNVSKFYSLSILICIVKLFFSIILNTYFFLKPSIFGKSMITSTMNHVWSISWLTLDTFSLCILTQYITMTVNEIKKTGDIVHQILRHSTSLGVIKQLNNFSLHLLHKNIQFTAMDMFSLDCTLLHSIVGSITTYLVILIQFQENSSEKHKP

>NvitGR15

MFLPSNTADKLLFLIKLFIIIRIPTCGKNIHVIVHSAWQTFPAMRRSKCDDLFLKCLFYVLKFLGVAPMAIDNSPAPEKDSPRYVRFVASKLGVFYNGIIACLTVYPSYMTVRYLTSSEYTKNIELEKVIDEAQSTFAMITSTFIIVNICVRQKRAAILANQLSSIHSVIMDLAIDVGDDVRRNTIISYIKKIVFVNLVTTIVWVASTPPEEYQYLSYFIVMSFYNIIIHAMLLQYSLVLKLLQQLYRSVNADLSSLLKKSSSISDDNNCHLMLKRLKHLRQIHATLCQISQDVSNFYSLPMLFCVTHVFLTQIIYCYYVVMTLIVWNINEKPILVILNCVTLVTLLAVSMTILVRDAGVTAVESKSTGEIVSGSIDDCQDHEIERQLNVFSNYLLHKDVHFSVFNLFPLNESLLISIVGSITTYLVILLEFEVDSTKK

>NvitGR16

MICKPHYTSDFFFLKCLFYWFKIFGTSPMGIDFTSAVKSNDVPQDVHFVFSKLGILHNAILVCLAIIPSCVTIKEAYHTEYTDRLQLERVIDTVHAVATIMTFYFILINVCINQKRAVAIANQLNSIYSQSTSLFSKSKIRPNRAFRSVKKIVSIHMVLMLLLCLFAFTFVHQNLIYHLFINFTNVTIYVMALQYSLVLKLLQHLYRSLNADLRSFLITRDRRDPSSISLPIDELQCRLMQVYEIHASVSRVSQDVCDFYSLPLFFLFAIAFFTLVLFFYYFMLVLLLMNKMIDYGFVLPFFLTLLGLTILAISTLARIAGGTVKESNITREIVSESIMNQRNQPISGQLRDFLHYLQQKNAKFTVFDLFPINESLLMSIASSISTYLVILLQFKESNSTQQTAPSSAT

>NvitGR17

MFIKCLFYAFKIFGLAPMVIDTTSTEKNNKEAHKIIFLSSKLGVLYNAALAILITLPTYLAITFAYSDYVGRLEFEKITDTIQSVFTIFTSVFILINVCVHQKRAVDLANRLQTVNYLSMTKVSCSDKSVKLLSSIKRIVLANAVTTILYFAVTPSNETRVLIYFLVINVYNTIIQATLMQYSLILKLLHHIYRSLNSELSSLNKSLIFVGEFNQFLNNSVQTILRRLQTVWVTHLLLSHVSREVSDFYSLPMLLCLSNAFLTLIMYSYHFVRALFIWKEHSGNMVVLILRTFMQVVTVAVSLTILTRAAGLTVSESKRTGEIVSESTVYGHNRQIRSRLKEFADYLLHKELKFCVFNLFALNEELVMSIAGSISTYLVILVQFNETSSIEN

>NvitGR18

MIVSKCVFYFFKIFGLATMRLDENETVRDSWCSGSKKGQVYNAILTCSIIASNCYVARLVYKENLSHREFEKTFDVVQYVYTTVTVAVILTVFCFCQGRAVLIANNLRKTYVLVENINSQMSKKEEDPVISGLKRIWITSTVIWISVVFTTSKLQFAVVMYYMTVYPCILIVNCAFLQYTIILHLLKQLFTILNANFLYVSTRQSVVTRKVEAAHSSFQAAEQSSQQFSDLRRLYMLLCDLSMDVSKFYHLIMLFCVTYVFSTLTMWLYYITAPLVTGTTPSKLQYIHSLMIVTYHIFMLIILTKSVDAVVQEKNKRTGEITNGWLANLQNQQLINELNLFSNYLLHKNVSFTAYGLFSLDESLLMSITGSITTYLVILLQFQ

>NvitGR19

MKMWSTHCFLFNFDEMFLKSLFYFYKIFGVAPMTLDSTKNRPSDVSFAYSKFGILYNLFLVILTIFAFYQCAILVHFDVSVGMDFQRFVNTTHLIFFVFTTLIVLIIFCVRQERAVALANRLSSIYYLNKNIKLSAILPSIKGIVSMTFITTIMWLVTTPYDDAHLLTYYVAISLYNFVINSVFLQYSVLLKLLYHLYRSLNTDLRSLLESLDIAIEMDRHSNNEIKLTSGRLKRLREIHMLLCHLSGDVADFYSLPIFFCITNAFFVLIIYSYYVFRGFAIAIMPVLVTVHCTTMMVVIVVSLTILLRTASVTAAESRVSGEIVSESMASCSNQFIGRQLEVLSIYFLHKNVKFCVFNLYSLDESLLMSIVGVITTYLMILLQLDGSSNCK

>NvitGR20

MYTAKSDLFTKCLFYFFKIFGVAPISYRSGVIPPFATSRLAIFHNVLLSCPVIGFSYFSLRHALYSDNAGRIAFEKIVDTVQTSFTILTALFVLAVFCILQERVVDLANRLQAIYKLTTCMTYKMGHDVNIGPSSLHTSVTRIMLVTLMTAVLWFITTFLNFDVFLLYFLVMTLDNLVIFSMLLQYSLVLKVLHYLYQWVNADLTSLLKTANADDFPKTMTRLSQLRCIHVSLSHVSRDVSCFYSLPMLVCFINAFLALIIYGYYVVKAVVVQSSSIPTVVILHCLALVVMHLLSIAILARAAGITVRESKATGEIISESIAICQSERVVKQLKEFSIYLLHKSVGFCVFHLFPLNETLVVSIISSITTYLVILIQFKESNQQ

>NvitGR21

MIHPFGFYTRVTKQVKMLHKNIVNKESIILKFIFYYFKLVGLSCVSFSSKSRLDICFLTSKLGALYNVILALLITCFNYYVVLIVVKVSFGSLHFDRAIDFGRVCLAVISSVFILITYCFKRKKATIILNRINTIAELSVNLRSNGKSGHDGLCKPAKRIFFVFLVTWLVLIAVTPKLKFYALLYFAAQYSCEMVIMCMLVQYSMLLSILKQLFETINARFTISSEANFQVRRFSQHRSNEFGLEVKLNRFSYLRELHLSLCEVAEDLSQFYSQSLLFCIAYVFSSLVLYAYFFVKIVTQKGDGIINTATTRFIIVKLLHYIGPTVTVTWAACAVVNESNRTGKIVNKWMGDSRDQYVAIKLNQFSNYLLHQKLSFRAAGLFSLDGTLMMSIAASITTYIMILLQFQDSTKR

>NvitGR22

MHIRNIHCKITIGKVLHYSLKIFGLAPFSLDVEFLSNNKNQVSSEALTCSQLGVIYNLILAVLIMVITYLTFKISNKTHIFGSGTDLDMAIEAIKTVWACISSVIILFLFGTQQKKLVQCGNIMLMIRERLITINETLYLENKFLWKSITKISLVVIVMYILIIVTLGGYIDLARLIYVIGATLCDVIIIFTVIVYGIKLKMIKQLIKIINANINSISTEFNRASNNILQNNEMKNAQIICDKLSRLQNLHLLLFNATEDLTNVYATSILLCTLYIFLSIILNLFYILKNVMTGVASLSAILIRHIFIQFIHCTCSLIILTNSVTDLVLESNRTGKIVSEWLTKLKNSRVENEVTKFLLYLKEHELQFSVFGIFSLDTSMLLSITSSITTYLVILLQLQFQQN

>NvitGR23

MFLNSHRPKKFYQNARISDLWFAKCVYYYFKTVGLATVSLRLKSVKKNKKNSSSLCTSSKLGILINVVLSLIVIAIFSYTTIVIAEGTFKNSLKFDRAIGVIRIILGSSAALIILITFSCKQGSITEIANNMQVLTIFSVLSANFKTKIGNTNESFSIFRETGGVFFVNIIAWLLLFVTVPTTNWKVFAVTPYVPEVIMTSMLVQYNMVLNLVKRLMEVVNANLLYTSQYDDKYEDNQITMIKNDRNENSFKRKIIKFTQLRDSHYMLCDISEDLEKFYSRLVLLCITYIFGSLILCSYFNTKEVLKQGVEFLTLRATLFFGVTVIHYIMPLVNLTRSTSAVIAESKRTVKIVNRWSGNFHNQPEIAMFNQFPNYLDQPNLEFTAGELLALDGSLLISIAASITTYLMILLQVQDTSPN

>NvitGR24

MLNYFSIDLLYAKCLYYYFKCVGLATMSVSFKSTVENKKVPYSLFSPSKIGFLPNLVIVLIVIGTHFFSLKMAFEVDEIETSVKFDRTVESVRLTFGVGVSVFILVFFCAKQEAAIDIANNIKKASVLSANFSTKTVSQKELFSVYRATGWIFSAHMVIWFLIYCSTPWSFGLMIYYVSLNIYELVITSTLVQYSILLKIVRQIFRNVNANILDIFGDSCAIDFHTVGTIGNNRSEVRFRRKMRKFSQLKDLHISVCDVAASLGQFYSIPALFCIKYEFISFTFYFYFVTKLFTGMYHETITIHTIFFYVFGILHFIVPLIDLVGSTSAVVNEGKTSVELISKWIEVVKDQEQSTVRMSHFPNYFAQKKLKFTAAGLFPLDGSLILSIAGSITTYLMILLQFEGIKPYSS

>NvitGR25

MVISICSLFFKSNSGNMLIKNLSLNETVLEKCILYFFKLSGIATLNFDFKLSTNRSKKFSSTFTRSKTGIAYNAALISLITIVTNYLIEFQINHNMYKNFYDKLDIGYAALISVTAVLILVKFSFQQEKTLTIANELNEIRDSLSLNDCSVDGKGHALRRFIVLVFLAHFLFLTILFSTSYVLNNTTINIVRTTLNYIAIYLSNFIMHSMMLQYSIILKLIEHLSRGINDDLVEFSRPPQGLNSLTFTKKTTSQRVGQLANLRKNFSSLCKVSQDVSEFYSWPMLLCLSCNFIAFVRAAFYIAMPIVHGTDAFTANIYVRCCCYISHNAFSLIILTKSVTASMTENRKTREIVNDCIENCDDQEILKKLEKFSCYLMHKKITFSVFNLFSLDESLLMSVIGSITTYLVIILQFQNNNAE

>NvitGR26

MWFSNFHDTREAFFVMFVYLFFKAFGLATVKFNFESIKKTLIAGKEASEVLKPSRVGIAYNMLLIIILSILNYVAIRVSYERPDFTDRSELEMKIDTVKAVTACFSSFIILLIFSFQQEKYVLTVHEVLSIRQSLISINSAIYFENESIWKIITKLLIFMFVNWILLFITIEIQNDYQFLLYFVTTNLCDMIMTHTVLQFSIALKMIEQLFRVTNANFDHDSKASFRLNDEICLNVALKKVQVILNKLSRLQDLHLSLCNVFEDLAGFYAQSMLLCVWYIFVSMILSAFYVTKPIITGNTGLSVVMYLRTVIHFLHHTSLLIMLTKCVTDLIAEREKTGKIISVWLAKIDNQQFEKKLTKFSIYLMHQKVKFSVFGIFSLDNSILLSIIGTITTYLIILQQENLSSNSNNSNCH

>NvitGR27

MFGKFDETILEKLLFYFFKMFGMATMKYEISTIKNEKIKKRRLFTYSKIDIVYNSFLILVTTILNIILVGLKIDDKTSLPNVRGVQKITDVVQFGFATLTCVFILVYFCARQKKAIEMADQICQIYGSMIVNNCDTGEKRSILLITLFVFSINFIFWLVMIITSVNVTMATPNNLIFYYLAVYSCHVIMQTLLMQYSIILNMIGYFFLHINKSLVILLKKPNQLFLDAQCRNIDKTRGERLLKMRKTYLILCKISEDISDFYSPLMFFCLSVTFVTLIRSGLYIAVSIADKESNLTIKGMIHCIGYVVHYYFLLVMLTKKASKIVTESKRTGEIVSDCVHYVDNQEIILNQFSNYLLHKKIKFTVFNLFVLDESLLLLFAGSMATYLVIMMDF

>NvitGR28

MLRTYLNQASFPLKMPFNNPLTSGAAVLKCVYYVCKACGLAPIAIRSNDGRKLRFPPFEHSKAGLLYNAVLILIILSMSAAIIQCTFVYRTKAEILKFDGVIDVTHNTMASVTAIFVLTVFCIQQRKILELANRMRVLGEMSQSLCDVEICGGRKRLLRDVMMICLTTCCTWLSIFFTTQVESYKGLLYFSYIYLCNLIITLTLMQYSIVLRLMQQILRVVNANFHHFSTEPSQRKLKVQIIETSCQGIGRFTRLRELYLSLSEVAEGFEEFYSQPMLLCIAYIFLTLIFYAHLITKPMVVGTRSVTNPQLCHCVFRIMHYVISLITLAKSVSTVITESKKTSKIFNKWLGTLDSLQLDPKLYLFSNYLLHHSLQFSVFQLFSLDGSLLMSITASITTYLVIFLQFQNHQTTDS

>NvitGR29

MNSKVLFMILKNWKISDLLFLKCVFYLFKLFGLATTSIQTKPSTSRFHLPLFTRSKLGQIYNVILAIGISFVYACLIRWTIKHYEHHIISRSHQAIDYTHTTMAMITAIFVLLVFCIQQEKFLNLGNRTVRLGELLVGFYEVQRPSKQKTLTKHVKEIYIMVGMTWLSIFVTTETGGYFKNVVYFSMIYLCNQIITLTLLQYSAILRCLQQILWFVNENFLQFSKEPCQMDKIKIQFSKLRELYLSVCEIAEDIERFYAKPMLLCIVYVFVTLIFFASFITKPMVSAVVISDFQICHCSFRILHYVVALIILVKSVTAAVSESKRTGKIVNKWLGDCNTLQVDPKIYHFSNYLLHHNLHFSVLELFSLDGSLLMSITASITTYLVIFLQLQE

>NvitGR30

MLSIKHMFYFFKLSGVATMKFNTNLIETGRVQGSWFSGSRKGIAHNIVLICLIFVFNCFGVRTVYYWSHIQFERVIDVVLYAYTTVIAILILIVYCFRQKQAIAIANKLQILREFTMSINGQLNQGEQLVVSSLKRISVAHLVIWFALIMSTSTEMYMLVYSIATYPCILIINCTIVQYSVVLKYLRQLYVILNANFFNCSKQFSANTSSVRTNSLSDLRELYMSLCNLSADVSEFYHLLMLLCLSYLFVTLLLWCYYIVAPIFVKATVLPILYGYVRSLIIVIHHVLMLVILTQSVSALTRENKKTGEIINKGISNLGNQRVLNELNMFSNYLLHKDMKFTVYGLFELNESILMTFAGSITTYLVIILQFHSRD

>NvitGR31

MDILKITLNFFKMCGLATMRFDAGTTQNTSVQSSWCTSSKKGQVYNLFLICLITASNGYVATIVYEYNISTQEFDKSFDVAQYIYTSATTVAILMLYCFCQGRAVSIVNNLKIMHKLVTNVNSRLSKEEEPTMGGLKRIAIMTTVIWFVVVFTSCNLSFGVVMYYLTLYPCILIINCTFLQYTMILHLLKQLFAILNANFRYVSRQSIVPKIAVAATNSMLQISTKKTQPFSDLCELYTSLCDLSMDISKFYHLAMLFCVSHVFITLTTWLYYITKPLVTGAIELSIIDYTHSLLILMHNFFMLFILTKSVDAVVDEKTGKITNRWLANLQDQHLVNELNLFSNYLLHKNVSFTVYGLFSLNETLLMSITGSITTYLVIILQFQSGVQK

>NvitGR32

MFIKDFINKKDKVFVKFIFYYFKVLGTATISFNTESTKSRKRNEWKFTHSKSSIMYNIGLIIFVTTVSSFGFIYASFQHQTNFKKFERVTDRAEDVFNILSVTIIMMMFCFKYKNMAGIANKMSMIYQSLISSCPQTLYKSFTDNILLHIILIISPYIIIWPFIIVFNFINFPEFEIYNFTIFMNDIVITALLMHYSTVLILLKYFFKMFNVRLSFMLEEQDYLCEIQYLNCNNRSKGKIKELFHMRKLYASLYEVSQDVSSFYSGPMFLCIFKILVSATLSLYYVAKPIIIDSHTILNVEIIRNSMFGLIYATALLIFTTLVTQTARESVKTREITNRCIINFENKYIIKELSQFSTFLLQADVTFTVYGFFSLNQSLLTSMTASMTTYLVIILQFQQNN

>NvitGR33

MFLKCLFYAFQFLGTAPITIKALGTKKNKACHFLFVSSRLGILHNCILLCISLPTIYLMIEDILARSFLRKKTNLELVIDGVCAIYVVVPAFAFLLKISISAEKAIMIINKLNVIYQKRKIEFKKESPPVLLLRPVKIIVFTNVVPLIIGCFVSGQHHISNLLPLPSFLTYQMTFMQYTLILKLLHYLYQSTNTELQSVLRSKVPIFIVHNRFLGIDSQRISNKIELLREIHVLLCHLSKEVSDFYALPMFFCISNKFLILVQYFYYAAAILSDKQETATQYEILILTIWFTIVEALSLVVLARVAGLVVKESRRTGEIVGGLIAECPNKLILKQLNGFFCHLLLVQVDFNVYNLYQINEPLLTSFTSYITTYIVILLQFKGISCPSDSTDHTENVSTPPSVN

>NvitGR34

MFLKCLFYAFKLFGIAPMAIKALTSKKNKACHFLFVSSRLGILHNCILLCISISTIYFIIDDTLSRSLFTDKSNLELVLDTACGICVALTSVVILLKMSINREKAIIIINKLNIIYQRKVESDKKNPSILLVGSVKIIIFSIFIPTIFAAIALGLEQSSILISCLPFTTYQMTIIQYTLILKLLHYLYQSTNTELQSVLTSKVPISIVQNRLLGMDTQRVSTKIELLREIHVVLSHLSNEVSGFYALPMFFCISNKFLVLIQYCYYIATVLSHKQDTTGQYEIILHCMSFSTVEALSIVYLTRAAGLVVTESKRTGEIVSQLIVDCPNKLVLKQLNGFFSYLLQVQVDFSVFNLYQINESLLTSITSYITTYMVILLQFKETSCRSGSTEHTENMSTPPSVD

>NvitGR35

MILRKSTEDIIFFTTCLFYYFKVLGIAPISLYIKSTKKSASQCVVFTRSNRALVYDVVLILNLVTANIYKILYLCLRVSSTKIITIEAVTNCLEDFVTCLSAVFILIIICFSREKLSAMVNAISGLTECLDGFGVENPKKHKLQLEIGMIILVNITTWILVFVTTAVAEFSYLLYDTIMYSNVIVVNALLIQYGVVLKLLRHNFKMLNENLLVISQEVPIKIQSPVESNRRVERLSQLRKLHASMCKVSRDVSNYYSYPALACVVCVFYTLIYTCYYLTRPIVLYDQNLRGDMFVMSLVYGLLLVFSVVILTKSVTATIDESDRTKEIINAGLLRFEDDEKMSKKLNQFSSYLLHTDVKFKVSKLFSLDDSLLTSMASSIATYLVIVLQFLQK

>NvitGR36

MWSIKKNMLFGKQIKKFDYLKLLYYYFKVFGLATMTFVTDSTKTTPNRFGTFSRSKYTIVYNVVIILVFVMPCLYNMTIFCVGTNRVKFEDFADCIQINMALFVTIFILSKFCISSDSLISIANSISRITESLLTLSSISLQKRIKVSFEIKQAFIVNITMWIAFIVINLSEIEPWMKNAVNMYVSNFLVSVLILQYSVILKFLQYDFKILNENLIEFRNEDSMKIRSPTETKAKIDGLLKLQKLHESLSDTSRRVSMFYSYLMLVSVLNIFIMLIFVCYYLAKPIILTHDSNFSSIMLLRCFWYGLLFVVLLVTLTKFVTATIEESRRTKEIISSCLMIPDADEKLLNKLNQFSLYLLHRDVKFTVWGLFTLDESLLTSMAGSITTYMVIVLQFQQKD

>NvitGR37

MLFLKKSNNKNLDYLKLLFYFFKVFGLASMTIDATTAKNTRNHFWTFTRSKSTVIYNVIFILVFVISNIYSMTFFCRGTYVVNFETIGDCGQTTLSLFVALFILTKSCISRNTLIIIANSISRITDSLLSLSSTSIQENSKISSEIKKMFIINISTWTVLFGTFAFDLKPLTKYGIVVFFSNCIINHLVIQYSVILKLIKHNYKILNENLIEFGDQESMAIRSPSEAKIKVDRLLKLQKLHESLSDTSREVSNYYSYPMLVCVLHVFIILIFVCYYFFKPMILHSKNLSTFTFLRTIGYGFAYGLLLVTLTKCVAATIDEQNRRTKEIIGSCLMISADEKVLNKLNKFSTYLLHRDIKFTVLGLFSLDESLLTTMVGSITTYMVIVLQFQQNLKR

>NvitGR38

MFFGKPINKKIHSFKLLFHYFRALGLATMTCDLAPAKNATKYYWSFASSKSAIVYNVILVLIFVTSNGYSMTYFCSSNYQVDFDTIADCGQTTIYSFVALFILVRSCISRNGLINIANSISQITESLQSLSSIEIREKNKVGLDIKKLLFVNITIWTGLFVSTVLEVMPWTKYTITVHISNFIIIVLMIQYSVILKFLQYDFKVLNENLVEFRNEELTKVRLPTETKTKVDRLLKLEKLHESLSDASRDVSMFYSYPMLASFLEVFIYLIFVCYYLTKPMILGNTYTTFMLIRSFWSGFTYAILLVTFTKSVTATIDESRRTKEIISSCLIIPVDEKVLSKLNQFSNYLLHRDIKFTVFGLFSLDQSLLTSMAGSIATYMVIVLQFQQNNR

>NvitGR39

MRRTKPDADALPSVENVTRTLTPVLWLSRFSGLTVFEMPAGTPWPKFSAAYALFLCSAYGTMIWFGETFIVKETTSVPLVIFIYALVKYVNAFLAAVSFAVGLLHYKKTMKFTKRLKHVDETLKVFGIEPEYAASRKENIRIVLIWIVATVFQIIGDAAICFVIYDPAYIAILKFFIFHIPFQSMSLLELTFAMKISTVRSRFEKLNALFQNVLENPVLPMHFKHVNKYHNILRRQHSEEGDRNRKNLELLLMTSRQLHLELCAITREINEVYGKQLAMSIAAKFIYVTGYGYVFYLYYNEPSISLSIKILNCTYIAYNLTFITVMMIYFIGQSVAAQKTTQIAHEMPVTQSQTKIIDEIHQFSLQVTQHPLEITAASLFTLNFAFMRGFIGSMTTYLIILIQYQPNIAAAAKSMIDEIMANMQNVSAFYHFANFSTD

>NvitGR40

MYSGKVSPPASTSKKAPISFRDTCLPILWLNRILSMVVIEVPEGRPWLTLSIIYATAKWVGYGYLLWYTVKNDESRINSIPIMAAVFQVILYVNVVIAVLSTYLGLANYKKYELYFKKIELADETLEIFGIDPEYSSGFKDYMKITAIWSLGGALVCATDFAIACYTFSSVPYAIIRILVFEIPMVLNPMVELNFSLMINAIGTRFERLNALIQSVAVTPLQSMNSRNFDKYQNILNRNQSKIAVKPNYYYYKNRNNIELLLRTARQLHLDLCATAREVNDVCSRQMSMQMAAKFLLITGFAYCLYLIYNDPNIPLSGKLQHYVSLGAWIVINIARMIFVVRTSVNAQKTSQIAHEIQVSKSQSNLIDEIHQLSLQIMQHPLFFTASGLLVLDFGYVRGFVGSVTTYLMILIQNQSDMLKAATTLANPNNDTSATTPSP

>NvitGR41

MFRIRNNKINPRKGRPILNIADSFRPFVWINGLMGFGMIEMPYGRPWPKLSVFYGLLRAIAFSILSWYVFKNIQPNTRISALMFLIYKIIIAASVGAVVISSIMGLINHEKSKKLYKKIKLVDETLKMFGVEPEYASDLRRNRNIIINYYRAIIVLFIIKLGSSYIFSREAFCTRNILNVLYFNIPSIINPLVDINYTSKIHVLEKRFERLNALIHNVTTSPSKMMHTHDFKKYEMILNNRGVISVIPKYCFKNRNNIEHLLKVTRQLHLDLCNTARNMNNLIYTQMSAQLSAIFVHLTAGTYCFYFIFNEKTIIPLQAKIHSYVLLIFSIICGIVRIILITYATAGKISKILHEIQIQNTEKKLTNEIHQFCMQLKQHPLSFTVCGFVELNFSYVTGFVGAVTTYLMILIQNQTDMIEAAKTMVDPIKSNSTSVTPA

>NvitGR42

MGDIFEDMEKPFLPMLISNWIFGIGIIEYPIRRQLKLLSIVYSTLILVVYAYLVYICHAHIYLTAVAQIKPIIEMLHYYTNILITISIIIFGWFQTQGLQKCMLKAAQANLLMQQIGIFKNHSNILKNELRKFMAFFLFILSIIIINSTVTFYNFVPNYQQIIFIVILQNVPLLYGYIADSSFLNIIGYAYFKFDSLNKLLKSISITKADNPMHKIIAKQPFYEKVYPQVYSSIDYKDYTFLIKKIKLAHLRLVKLCREANNLYSFHILLSIAIAFVMIINKIFNIYVVLNDDDIDEGSKFRTIVRSVNWLIYYIVRNLTSCCLCTTVLNTATKTGDLICELYDEPYITENTRAEIRYFNIELVQNKLEFSAYGVVNIDLTLLQVMASTIATYIIIIVQFQKLHFVPNALVGNQTNTYRI

>NvitGR43

MVDIFVDMEKPFLPMLVSNWIFGIGIAQYPIGVPHRVLSFTYSLLNITLYCVVAFFAYPYYIKFIDVTKSTLTTMIFSFSISILLTIIMITSGWFQAKGVRKCIIKAAIVNYLMQQIMIPKESTVIFFKEFIKFLIPLTSIFLIITFNLFITFSESFAHIGQIGANFTMNYPIIVMFIIDSSFVNIIGKVKNYILILMYANFKFIKLNELLYSLLTSSADVPQHKRTFKEFIYKEKNSKFQYLKSIRKDYSDVIKCAKQIHLRLVELCQHASNTYGLHFLISTAFAIGIMIYNTYNIYNILASIVITSAEYNIHIMKPILYNCNWLTYNILKIVRLSWFCDSICKESVRSGDIASELYDEPFISENTKSEIRDFENELISNKLTLTAYGFFHLDFTLVHAMICTVATYLMIIIQVKSTCR

>NvitGR44

MSDIINKLEKVVLSIAYYTNWFCGIGIIEYPIGKQHRVMSFLYTGVVLIVYSVLSVYVYSDFAIVSRDYEVNQTMMKGVYCATFILTFSTIVMGWYRNKEIRSILQRMNIAMRIIDKLGASKNYTKAFTVQVGYAVGTLTFLIIIVIINALVVYKRDPKHDSHVLTVMAVNYPLIILQVVDTLFINIIQYARKNMRVINDVLREMLTSTQDFPQHTKIVRRYLRTLDSPELDTDIVDQKTDAEDKMYTINMSKKAHLTLVKICQETDDTFGLHILLSVIVAIITITVSIYHIYMLVDYLSISRAIYDNTLLPVCILLIYYYVKIHAISHFCSSTSEEVAAVITGDIISELYDDSSIGIESQTEIRQFGDQIVQNSLTFKAHGFVTLDFTLIQNVVGFVTTYLMILIQFGSSTSIEISR

>NvitGR45

MGKKPMTRPILPLLISNWVLGIGIIEYPIGTPRPTFSFIYSTTLLVIYCTTSIMIRHEIFRVSIILKNNTVPMTIVFYTNIFLTISIVTLGWYRSKGLRRYVAKAAVADDLMERIGIPNNHGKMLRAVAGQVIKGFFLVTVLIAIIAVIVLVEDAPLQTKILISSVMSFPLFTMFVSDAMFTSCVRCACYRFTELNKVLKAVLTSTHAFPQHKRVCSSVFESGGQDSNFVINNVSQRKNPAVIVKLAKEIHLQLISACQEINNTYGLHLLLSIIFAFAVITGNMYLCYMSSRNSNIPHYILVKTLVVSGIWIVHYGMKICYFSIVCGCCTENSIKTGDYINEFYDEPSTTNETKLKIRQFNMQLIQKPCKFTAWGFVDLNCHLIQVMIGTITTYLMILIQLGTTTYVADDSYSKYLKSFSSLTY

>NvitGR46

MRSYHIEPAKTTDQAVQLPVWTITKRSLANTSELRSSKIDSSPKRGRYESLKMDSLPADVSRPSASNSGANLVESTRSFHCALRPIIILAQCFAVFPVSGVRSPDATHLKFTWRSFKILYCCLSTLGSIVLMFFSVYRLATTTISSNKTSNLVFSLTAGITTLLFLKLARQWPSFAVSWENMERELATRHNPRRSSGINLATKFKILSVVVMVFALVEHTLSILSGYVSAVECASLRGDKDIMATYFALQFPQMFTDSNYTLWKGLIVQFVNFLSTFSWNFMDLFLILVSVALTDQFRQLNQRLYSIRGKAMPEWWWAEARIDFNRLATMTRRVDSQISDIVLLSFSTNLYFICIQLLNSFKPMPNAIQTVYFCFSFGFLLSRTSAVSLYAATVHDESLLPAPILYSVCSASYSTEVRRFLTQVTTDNISLTGMKFFSITRSLILTVAGTIVTYELVLVQFNAVQAEHQQSESNITKVCETLEITDVKIEDF

>NvitGR47

MLKIDDSHQSFQYKLSEKNMITLKRIFYFFKICGLATMKFDSNMAVKERLQGSWFVNSCKGKVYNAILIILLSIATYYVTAFVYDLSISSGEVGKLFDIVSYVFTTITTIVILAVFCLCQKDAVLIANNLRRTHMLIANINSQLSNEKINILRTAKIICIVNLVMLILVFITTLRQEFGIIMYYTTVYPCLFVINFTFLQYSLILQSLKQQFTILNRNFHYVLRQCIMQKNLGATGSSFQTHKVRAQSLSKLCELYASLCDLSTDLSKFYYPTMLFCVLYTFMMSTTWIYYTVEPVIVGKTKLTTFKYIHSLIFLIHHISMLIILSKSVNAVVLENKRTGEITNRGLANMQNQQTINELNFFSNYLLHKNVSFTVYGLFSLDESLLMSITGSITTYIVILLQFQSSVQ

>SinvGR1

MERESHVEVEDSPSIEYILRPITYISWLMGVGVARPRKCPKAITIFIRIVHLAVCSISAMYCVLDFFSFNSIFAFKTNVYKLMHCTNRMISYVAAYYYVCHGIRQHNKWPELMDRMKNLDQKIRRETSINDLPIKNAQVLAILATFACCPLSLIAHVLYYYFTNPEYIFISDLLFYFILAQSLIDSFVFDIVVYVLYCRFQTINKLIGQMDELFGAQQIALKIRRIRGLHNGISDLVVMINDIHGLHLLLLSANCFAVVVATLFRIYIGITEKNYTLIAVLNGCWILYVTQFGLICWICTLSRQESERTGIIIYGFVLNCKNLDQDAGVRNEINDFSIQLQQYQAAFMACNFFEMNNALFSGLVGVISAYLVILIQFYKPSDSN

>SinvGR2

MGVGVARPRKCPKFQAINELIGQLDQLFDASKIAPKIRRIRVLYNSINDLVVVINDIHGLHLLLCSANCFAMAVATLFRIYIGVREKDYMLVAVHNVLWIVYVAQFGLMCWICTLARQASLRTRIIIYTFLLNRENLDQDFGVKNEINDFSIQLQQYRVTFTACNFFEMNNALFNDMVGVITVYLIILIQFYQPPKDSKHNELIHGLTLLRADNSYRQTEITLNNTLTFY

>SinvGR3

MEETIHVPDNPPSIEHILRPISYISWLMGVGVAHPRRCPKVITIIIRIVHLAMYFYVLVNFIVIVRKISFNVLTIQILFSGSISSVISYVSGCYYIYYGISQYNKWPELMDRMKKLDQKIRKEISMDDQPAKNFQAIAILAVFAWFPLYPIICTLYTHLYSSPFSIKWWNVYIKAQSWVNSFVFDVVIYVLYCRFQTINKLIGQLDQLDAQQIAFQIRRIRELHNGICGHIIMINDIHGVHLLLSSMDCFAKVVTLLYQLYVCAMVYKCIIMTDIYLYSIYTLYVIQFGLMCWICTLACKEFQKTGTFLYNIDLNSKNLERNCITKEVNDFSIQLQQHRVAFTACDFFEVNNALFSRFFEVTITYLIILIQFTPKVGVHHFYLGRQILHRGITLL

>SinvGR4

MFNLLSKFQKLKKNKKWKIRWLFHATNFESLMYPCFTFCRIIGLFPYKIDAMTFKISKPCYIFSGVITCIACVYNLIILYDINLSKNIKYKSLPMTLERNCFYVFGGFIAVVTFILSGPRMHLIQTILEVSLRLPQESYQNLSRLIHAKDIFGFFCIIVQAVIFYSWMQFSVLRKILTLYFVIVLFIMDMLYMNCVCVLKACFKQINDNLMNLPGLVTNREIYFLRRTYYEQRNPFLLMQLKALKKQHLEISNTVQMLKIIFSLQLLSTISLTFIQITLNLYFYLVRVQVRSMSYRENQVYYNYFITAITFCLTKIVLIIWACETGKNQAAEISITVQDILNSTSDKETKYELHLFSLQLLHCENEFSVKGLSVDFTLLSRMVGAITTYLLILIQFVFLLNSCDENSVIGFANII

>SinvGR5

MSRKDVHIRWNNFEKQQMANMNNKSTNVEYDVNSDETNDKLVNSDLYQAVFPIYHLSKLSGVFPTRFIVQVPGRYEGRLSIIDSVYSMCLLICLIGAEIWGFXRDLRDGWKHSTRLKSQNAVIVTTSDIVGVMSLTTASIVSSILCWKQVQTIIDKLVDCDKKLGIVSPKKIQRYTILVTLCSLFYSITISCLDIYTWNYEVKLNKKLDDKGPINYVPLYFMYIVIMMMEVQYAVVTYNVCERFCRLNKNLENILNCGRITNQFRKDLGLAGDLHDQGQLTTYIRQDMGNVRMFRKSKIMDSSITNDEKSFTDTISQLMMVHASLCDTVTLINAAYGVIVLVITITCLIHLIITPYFLIMEADGRREPLFLAVQGLWCTFHTWRLLMIVQPTYAATTQGKKTAVMVSQLLSMSSNKEGRKQLEIFSLQLLHRPLEFSACGLFTLDRTLVTSIAGAVTTYLVILIQFQKDDDTKGNFDNIDNILKNATQMLKNASTIHNLTAGRLGLN

>SinvGR6

MNGSLNINNNTTLKTNRILCTNSKPNSATFQKILSFLRKDKVQMIETQFSEPPMRARPFLQSGFLSGQILSPNNNDPESFHCAIRPVLSLAQMFGILPVLGIRRPSPLQLKFVKFSFCTIYAVFISTMVLVMAILSILHMIKTLNSVTFEVKGGIAAATAGAVFYTNCVIGQLIFFRLSPRWVTLQRDWRSMEQFIDSNKIERPKLRWKFNTISSSILFLALIEHIFSIIQNTLEYDWSGKSENSTFQNFLQVYCTSSHSFILQSLKYNFVLGIFIFIVSKLATFTWNFTDVFIMTVSTGLAERYRILNKHVMSSISKHQRIDWCGLREDYAALSCMVKKVDKNIAPIILLSFANNLYFICLQLLNGLSKPENGILSDIYFFGSFVFLIGRTVIVTLLTSRIHDQSKVILPLLYSCSTSTYNTETERLIYLLTTDDVVLTGMRFFSITRNFMLAVAGAIVTYEVVLLQFNVAIKGK

>SinvGR7

MNEMSEDKSNAKNTLRSAWTAGSSKYPVMIKTQSPKNNLRNSRFDSMKSDVMPEMLAPVVSSSDVFNPSTDSMHAAMRPIIMLAQCFSMLPVCGVNKPDASYLRFTWRSLKILYTAVVFLGVLLISITNILRLLNTGINSAKMTGFVFYATALVTAIQFVRLAMQWPCLALTWEKLEREFISRHRRISRTTLATRFKIITVIVMLLALVEHTAAVLSTYISAVECAEYRGDPDIVGIYFQKQFAQVFTLTSYSLWKGIVAETINVLSTFSWNFVDLFLMLISIALAGQFRQLNSRLFSIREKAMPEWWWAEARIDYNHLATLTRKVDSYISSIVLLSFATNLYFICIQLLFSFNPIRGIVRKVYFFYSFGFLLSRTTAVSLCAASIHDESRLPAPILYSVSSSSYSNEVSRFLTQVTTDNIGLTGMNFFSVTRGLVLTVAGTIVTYELVLVQFNSVQQVDQSNITNACELFFQ

>TcasGRa

MCQIKNKLLLLDSSTQPNYLILSNKNNTYLVLNKYLLVKHSKPKMPLLPKTPLPLTLLYKLLGIIQFPISAHFSFMSRFLCLPFYSYFFYLSYIYTTYSRKLSGIFKYIDQMAGYTGFLAMLTSMVMFYKRSNDLKTLLSNLESIQIYSIKPKERNSNHWVRSGLFALITGNVLFYPFLPSDVSYNLFSFVPLVVNALDHLFLNDILSDICDKFEQINQHFRRQIKSVDLFVIFPLTKAEKVRNLKEDEVTFSVQKIQELSHLHYKLANFTVKISGLFEITTITAMVMWFGYVIDTMYLFIHIRSRQEDTDTLVVIYTFNLFYLCFCFYWLLVMVAMFSRTQQSANKTATFVHEIWNKYALKNEVDKRVRHLQLVAIRLLNTKLQFTAKDFFNLDWTFCHMVSHKWLNYINQFEANFR-

>TcasGRb

MHTLQHFTIDTRSTHSMKNFKYLKVLVTFAHFICLFPITINIRKNGLNYNFTKKCYFLRMVLIDLLIVGCILKHIFNILMKNVTLNDVVFIFCSAPIIITILDIEILGYVNKAKFGKLLINLYTINYNFKESERNDYVSLLQLVFVFCYFIYLFTVYYIYYSVHEASIINFGYTLAKFMIFTSTCIYTNLLRIIEADFSKLNHLLSGTENLDLVLPIYSQLVFMCKKINKLYGHQLLLTILTYLIWTIYEMYHLAILWSCTSTNCPRFLIMLALSYTVIQEVMLFTILWNCQNTRVASEDFKAIWYSILIKKADSFCKKKLENYSLQLINHRVVFTAMGLYVLNMEHFFSVKCSIQSESLLNDILFPVAGFISDFDCDINSIQYFCLMARCLNTLIFYYHISTYEYSI-

>TcasGRc

MSEREVILKAALTSRFFLITIQYVSNLLIPDHEADAYTYPKNEDNGIFDKLVTHLLGGFVRWDAHHFMHIAIFGYTYEHTLAFFPLFPYSAKPVVAILSYLLPFLSTDSLTLITLITVNIFCFAQSALCLYQLSALIMNKDLALKAAILFCCNPASVFFTAPYSESLFCYLTFKSMLNSVLLYKKYKNQGYLLSDVAYIIPICLSTCTRSNGVLNIGFLAYALICLFLEKIKLEKQICNLLLCLAKFITLAAVLVLICLVPFICFQFYGYQTFCKNFKSYQVPLVLGHKNIDNFVLPGTFSQHNQSWCYKKMPLAYSYIQSHYWKVGFLQYYELKQIPNFLLASPIILIILGHSLHFLKEFPKSISKLFNFDLISVKSVRTKKFFPVMAVFIVHASVLTLFCVFNIHVQVTTRMLCSASPVIYWFCSYYVTDVNLFKNLIARKCNWGELLVLSYFLGYYFVGTVMFCNFLPWT-

>TcasGR1

MRNDHGSNTHLHPDDAIRRAKIVKVAASPTSANPDEEPDPELLDRYDNFYQTTKSLLVLFQIMGVMPIERSGKGRTTFRWLSSTSIYAYFIFGAETIFVTMVFKERLYLILRPGKRFDEYIYGIIFLSILIPHFLLPVAAWTNGTEVAKFKNMWTRFQLKYYQVTGTPIIFHNLTLITYSLCVISWAVGIGIMLAQYYLQADMLLWHTFGYYHILAMLNCLCSLWFINCTAKGRVAVWMCNNLHKALESRNPAKILGAYRDLWVDLSHMMQQLGKAYSGMYSMYCLLILLTTIVASYGSVTEIMDQGISFKEAGLFMIAFYCMTLLYIICNEGHHATRKMGPEFRERLLNVNLSAVDQKTRQEVHMFLMAIEKNPPIMNLNGYANVNRKLISSNLNERNCFSNIVKLRTLSPTQT-

>TcasGR2

MEISDLAQLYGNELHIKQISKWLRGSARAQEIQKRSELDSKDGHVIDEHDQFFRDHKLLLVLFRVLGVMPIQRGEIGRITFGWTSIPMLYAYVFYVVTTVLVVLVGYERFDILLNKSKKFDEYIYSIIFIIYLIPHFFIPFVGWGVAYEVCDYKNSWGGFQLHYYKITGKNLQFPLLSTLIIIISLGCLILAVVFLLTLSALLEGFTLYHTTAYLHIITMINMNCALWYINCRAVGNASTALAESFQNDVDRNCSAYIIAHYRVLWLSLSDLLQKMGNAYARTYSTYSLFMMANITVAVYGFTSEIVDHGIRFSFKEIGLLVDSTYCLFLLFVFCDCSHQASLNIARRVQVTLLQVNLSQVDPATRKEIDIFLVAIQMNPPKVSLKGYTVVNRELVTASVATIAIYLIVLLQFKISLLNMRG-

>TcasGR3

MPKTHRSIPKSALNSNAAGWGCLPSSSAFSAGMKSGCGSAQSMGKRLDTHLGLVRALSRRWPTKNTDTTHAIQFSSALSWPQADASHPRPGCVTSRLSRTLLLLLLGSAQRRRQRRVWTTPRRPKAAGEELGVVSTINSSTMYHQDQAVSILGEAIPKRRSVFLESGVNSADSFKASKVGPAPPIKFINKSSTDKFGNGAIYEVLKPIYALMRIVGIFPIKNTEPGMFRVAPELLGYSVVVFVVVMGYIGFIEWDKVEIVRSQEGRFEEAVIDYLFTVYLLPIIINPLVLYEARKLANVVTDWVNFERIYYKLTKKKLSVFFGNKPVILTVVLPLLACGVMVVTHITMAHFKIIQVVPYCYINCLIYLIGGFWFMQCDVVGKVASQLAEDFQMALKHVGPSSQVADYRSLWMLLSKLIRDVGNASGYTVTFLCLYLFLIITLTIYGLLSQLQAGFSTKDIGLTINAGLAIFILYFICDEAHYASNCLRVQFQKKLLLVELSWMNDEAQQEINMFLKATEMSPTDISLVGFFDVNRNLFKSLLATMVTYLVVLLQFQISIPEEASPTNSTTITTQTPN-

>TcasGR5

MSLKLVNLVFKIGSLLALTPAKIEKNGLVFPTKAYSLLWAVLFSGALSITAIFRKASYEKLSPVVLFIQVAADTVLFILNISTIIITARKKQQWNSLIKILKTVSNRNDKGDIFWFSPFLVANLAFVTIVTYETFVWTQIMGAEFFKLYAVEYFQMYAQFIVYYLIYVFLNSILEGFQHLSKTMCKYLKLPNRSNNFSLKKIRSEFCALAIFVDVFNDIFGWLILQSIGFTFLQLLSYMQHLIVGTGHTIPTLIYRLSFITWYMVGTFNSVFICDLIEQKVKNIQMLVYQNEAEEVKILLDVINHFPHFTAARFFDLNRKTILGVLNALFTFLIVVVQFENLTS-

>TcasGR5a-trehalose-like

MTEFWLEMRRDYDRLSHLCKELDDGISGLILMSFAYNLFEVISYLFHQLMMDSQQNVAFYFFFPYMVLRLLAVCLYTSWINDESLAPVNILNSVPSRNYNPEIGRWLVQMSFDNVALTGWKMFKVTRGIFLGVASIVVTYELVIMQFYGFSGKT-

>TcasGR12

MKKNSKYVQNCIFYAMKRPLLVAQIFGYFPLYGTNSDPTCLKFKWISFKTTYSVFTLFVTFFIAVCQLHKMIAVEMNILQMNYFVFLLCSILVNIAFIKLATEWPQLMKAWLKIELLVGNLGMRRNFRKKLDFIFTFITLLTIVEHLLMELSRAIDSVACSKTVSDGIRHYYVNVTFPHLFNGLVDYSLWKALIFQISNLQTTFGGTFGDTFIILLSMAFATRMKQSRTKIEALVKSHVKATTPWRKIREEQCSLLYLCTLLEQKISYLVLLSFCSNLYFVLVQLFSALKQMGDTLQKTYFFISFGILIFRIIFVSLSAASINEESRKILILLLSTPSELYSVEVERLTNQINYKAMAISGKNFFIITRGLILKIAGAVVTYELVLIQFNKKLLNEFDETSVQQLEILHNNTYWLC-

>TcasGR26

MANCATALTLVCWGFWGFVHDMEIASFVSLGFTGSVDVVISSFDISDVLLSCLYFIVSMPFKCAKLSIVFHNLNKVDAIITPVFCDRFYSNLVWFSRCWFVFLPVLYTLDVFMWGNTSWLGVNNYFAYYVSYSIVVLHELQYYQVVKMAQLRVSGINKTVKENIKKDTSRIKLEFIFDLIHCYNNTTDAIETINSSFNKTVTLMLFSCYVHLVTCPYQLFVMITSNETSILNYVYCLWVLLQIFRLVLVVEVCHNCEEEIQNTRILVSQLLNCRLDKNVKKEANTFLFLMVKKKIKFSAYGLPKVGRHLLLSVASSIGGYWMILLQFSSRTSKI-

>TcasGR27

MQVASFTALGFRGTADFVIACFDVNDVIVSAIFFVTSTPFKFKHFVQIVENFDRIDARISPILVEQIRKRSNIFVKVLVTFLPTLYVLDLFMWGKNNWEGLNNYFAFYIMYSIVVVHELQYWHIMTMMYARILGLNKTLRDYFKNKTGFCEHEILVVTQSFNSINDSVEEINKCFSYSTTTIIFSCYIHLVISPYQLFVVVSSTETSLFNYVYLLWISLHIMRVLTIVEVCQKCENENRKTRSLVYQLLLCKLNEKVKNMVRVLFFLVTTRKILFSAYALPKINRRLIISILSSISTYWMILMQSTSRTIQVV-

>TcasGRSG43a

MTITISKELFHVLSPVLYLSRFFCLQPLKWTKTSAGNYIITKSRFYTIYTLAASCLLVITSITGLSQVYQLDVIYLVRLGDTTRRFVTYSDIVVVLLPCVIGPVFALFKTNQTINYLSHLKQFDSLQNQPTKSTKIFQITALTTFCTAFTLSMDLFLWLKLSHNYIFLLCLPYYISYWSTVVIELLFWHFVHLIQIRISVINKKLAKMVVTGLNSVTTLKKPHAEVEDLVKGYEKLIEATNSINYCYGFPILVIILGCLIHLLVTPYGLYSIIMSTGDSTSILSQTVWMTAHILRLFLIIEPCHECFIKTKETSQLICKLLCLSVNQEVKKSLEFFLTYLGECKIEFSVYGFTKINRELLTTIAGAITTYLVILFQFK-

>TcasGRSG64b

MNILQMNYFVFLLCSILVNIAFIKLATEWPQLMKAWLKIELLVGNLGMRRNFRKKLDFIFTFITLLTIVEHLLMELSRAIDSVACSKTVSDGIRHYYVNVTFPHLFNGLVDYSLWKALIFQISNLQTTFGGTFGDTFIILLSMAFATRMKQSRTKIEALVKSHVKATTPWRKIREEQCSLLYLCTLLEQKISYLVLLSFCSNLYFVLVQLFSALKQMGDTLQKTYFFISFGILIFRIIFVSLSAASINEESRKILILLLSTPSELYSVEVERLTNQINYKAMAISGKNFFIITRGLILKVSNKH-

>TcasGRSG64e

MTTTTHSSLRFILIAAQIFGMFPVSGVAKKDPTFLKFKWTSKRTIYSIIFALAAVVNTIIFLVHRVSLGRLKFADWVTLVFFSVTFLIVVLLLQIAKHWPSLMKKWTQVDEAMSGYGFPPKLERKLRIIFAITVVASLVEHGLFIGVEYMSCRGNNLSEALDRFLMFHYDYVFALVPYHVVLGIILEIVNIFSTISWTFMDLFIILVSLSLSARFKQVAKYIKFLVERNVLNKNSWQRARQDYTRLTNLCKDLDEVMSSTILLSFGNNIFIILVHLYNSLQKPLEFGYLDEIYYLYSFICLLVRISAVALHAATINTESKRPIYLLSTIPHHRYNLEIDRLLLYTKYETAALTGYKLFRITRTLILKITLAIVIYELVLVEYLKVESY-

>TcasGRSG64f

MKNIISHNTENTIHSSLKFSLKILHIFGLFPVSGLSGPDYKSLKFSWRSFKFLYSLCFFCILCLFVLTLLYNVFFVKEATQEITNVLFYLSAAATNAVFLQLAKNWSRFIHEWHCVEVIMGSVAINHSLKKRLKIITIVILVVATVEHLLIQCYIAVSIFGSSSFEADLRQFYKTAYSAIFTVIDFSLCKAILVHAITIRSTFSWTFIDVFIMLTSTAFVFRLKQLNAKVEMLKNARVKNTALWKQLRYEHYRLYQLSVLIDNNMSYIIIVSFATNLYFIIIQLFGSMKIVKGTLKTAYYLISFALLIMRLISVCLCGASVHSESSKVLPLLFSVSSSSYNCEVERFIDQVIKNEIILTGKKFFKITKQLILQIAGAIVTYELVVIQFNLRLTEDNDSNEANLLA-

>TcasGR82

MPTRGTISPFHRFVIHLCNVFSEILSCTQILFFMVLTSFMIHHMLRVNIAIAIVEMVVKPNTTTPTEENFGPKYAWNEREKNDLFAWFFWGFLITQIPGGRFSEIVGSRIVLGLGILVASVATLLLPLCCNVHYYLVVASRFCVGLGLGVHWPAIPPIAIRWSSSATARTMFMTHLFAGSLGAAIVLPVSGHLIAYVGWPSVFYVTGGMGVLWSVMWFYLIYDSPGQHPRISAKEKEILEQKIRNEITPQVRHIPWIKIFTSLPVWAIVVANASICFGFYIIFNHLPTYMSSVHNVEIEKNGWISSLPHLGEFFREKFVISAYFISGRYITTVAVSYLGARMLHKNKFSTLTIRKFLSVVCSWSAVLLFGMEALFGYHYYVTNFVSITFFLFLSLSIPGMIVNILDISPAFSGTIIGFNQVIVCLSGITSAKVVAFFTATKQSFEQWRYVFIIVAIVNFVGGLFFLIFASADVQSWNPKENVSQKKNTLLNDTTSKDHEEF-

>TcasGR87

MKEVPGWRNMKPLYYLCNFLAVTPPHSLGNTVTPVSLRFKLYTIVHIFIIIALYAHSSYGRENFIYGSMNMTVAITDKIANFMLTFFNVSLRIILVFSKGKVIKSFFNQGYELSKQEIFNCCSKKSFRMNFAIFNLYMVLLLLFDAYLWISSVGVRMFQYYIGRSFTYYVCNTTIFLIFHSVLPIKRFFTSLGIAFDNIMKNLICEIDGRHEFFLAEFKSAKTPPNKLNCYDLRRVRKNYNSICELVDAFNNIYGLAMLEIIIVVITYVLNLTDLFLVYGMSKSRNIEGVSFGTNLVILCALWITTLLLFTILLAYGCAGATSEAEKIAKICFYWLNEIPTMPVSIKDQTIKEELALLAQQSTSRTSKFSAAGFFPVDFTLLGFIFGSVTSYIIISIQFIE-

>TcasGR93a-bitter-like

MVTKMVCQFSRNNDLLRNSVSKPLFWYLIVQVAITGNKILGAVLTISPDAEVYLNVLFYNFAELFSYNINILIFFYLIIFQNAYTSYFKAITRKNTYNDPKIVVQIFYKFYEISNNINETLQVPILLRIFTDFVFTISSMFYLSVFLYIIPELSFFVLAKMVLWLVNTLFAIFIMAYFFDQMTEKRNTIMETLEDLPSSHTLISKFHSSYKRKELLRLRLKHERFCFNVCGFFPLNNSFIYLMIAGIVTYTTYLVQFGKAANAKQ-

>TcasGR97

MKNHFSKLMKNRRGRKTSKMMNGMTKQIQDVMPGGNRLKISLTLFAELTMSSQSLHSTLETLCFLNQLIGAPFSQTRHKLYPTYCKILLFTHLTTLLFTFCNIWWRCSNSQRISTKLIKTLNYFFKVTIIMSTILNSMILKKKNVAQLIPTLKEAKVMLPDVKQKWSCFTHFDSIFVLFSIMTSFIFHLTYKSGCYLIQDLLDDVEMVQSLIVLLTSLKFLELLSNYFDQTNNYFDIILKSEIFISDMKMRRVNQLYRTLYRILELWNNIYGVFLLQLFLHIMVVLIDNLLSLVEEFKLGYSFSWKVVIRSQTIAIYLVLALLLSKIGYQISEKKYKTSKTVLNGLVEITLRGGKQSQLNLLLLKLKTWPNFVSASGYFRLDYGFFLSLLAAVVSYIVILLQLS-

>TcasGR117

MCLAIILMNNKLNMVLVKSMLQRIKMEEKLVVEKFLNSLQIYLQHNQIFGFVTFTCTRSNFRSSKLLILYNIILQVLFVSFVSYWLYLVLEADDMLPIYKNTYLIILFADFAYLETTWICTLLKKDKLLELFKRLIHFDTKCQENSTVIDYKRHKKRLLCYLLARYVALALVILFSEILVIVSEQEWSFSTGLLVMIFNSALSYKASEIVVMLRSRFAILNKQIRFLNQYLRLKPEGRISNRRVFISFSKICYLHQHLSKSVKLFNEVFGVSLLVLFGNSFLSIVLALFRTAAELQASQIKWTRIAYMALASVPFIFDSIHLCDVCYSTIGTVSKAGELIHQIQTEDHDIIDEIEMFSLQIANEQVEFNAAGFFPINYTLVFSIIGGATTYIIILIQLSATLNE-

>TcasGR118

MRNVPQSYCFVTILFDSVNKLGTQIVNPYSVKQYVMQQQVKNFRHSVRSVFFLSEIFGLVNLKYRETYFRLSKTKTFCTLVTALVYCSLAIFVLCELLIEGTTSILINVPSLIIHVSTSAYVATVWINSVINRWKFIEFIRKVLEFDVKCVSNYTKQQSKIHLIVRSVFVTTYLMFDYCTVLRVQRFNNYQSLAHYLRVFFTVFNVVHCYLASELVLMLKNRFVTLNVQLTKLTKNCATKAQSVVLGRICTLHHHLSKLVTRFNEIFGLGLLLMFGVSFLLITQTIFIICVIVQSEQIAWLHLLYIFLVGIMYAADVFYICHVCCSTIHEVSKAGELIHKIETNDHEIIDKIEMFSLQILNERAGFSAAGFFPIDYSLVFSFLGGVTTYIIILLQLSSSVPV-

>TcasGR125

MKKLNYTVEISYDLEEGVKTAHCTCPRGNVACHHMAAALYYAHYNVSATDIECQWSAPSKTTPQTEVIKLADVYKPKLSNYTALSRSSTEDEIIQFRAEIGVTNVVGFTWLLRPEASEEARKIIADIEEILQSLEYVQAIDKQKFLLEKCRIDEARIKLVEACTRGQHVNENWHVARKHRLTASRFGMVLSACSRRRFPPSLFKNLAEGYSLDRVAAVQWGKTHEKTALREFEEATNLKVQETGFWLEESGFLGASPDGLVEEDGILEIKCPYKYRDTDSLSEALKDKKYFYWRDENEDINLNSNHNYYHQVQGQMHITGRINVKSSSNMIKFSKVKSYFTTHHDTLHNIYTSMKPVYAICKLIGLNTLRIGRKGELKQHKSDYFYFSFYITSYTLLSVYSLFRIATNENNSLVINKRLIFIECFVMMALTLIVTLFTFLARGTLIKSFDMLSHVDVSFIKAGFRLEYKQLLKRSYLIISFVLFSLLARVPVMLMTISADFIQQIMLFVSALIKAFSKYQFVVLVLQLQHRFGKINRTMRSFFSDNKQDDKIPQISDNLYILCRLHYKLTSVMQKINSAFSVQLLVSIGVSLFDVLFQAYYLYYVATGKASFVTVPMIVCPIVWLMDEVVEIYLLVYACASTCEQANDTPSILHELRNNYFHMDLENNVQSYSLQLLHQKVQFSVLGFFVVDYTLLYSIVGAVTTYLVIFIQFDQSSNSRNNYVLTNNSTC

>TcasGR153

MTIITRSNNVVGGKFYKKRNKVSNFGLTFEFIFGIIILYTTYSMSFVKRNHVRDVVLQLNRIDELLAKMKQKFRYTRAVWYQLIIFFSGFLMILIIASMQIQNIRIENFPPLSLFMCIIFLFPLVILYDLNSQYGFTAILIYERCFIKCKTTQKHTFRYFKQNNDFDGNPRNLMQGGKHHKLKLQHSDATSHNARIFHQSLLNFLLLLDGNVTVVDTILNLQIFQHSSKPFSASCWLAWGFLKSYEILHVTISCHLASQQANIVGRKVHKVLIRTQNDEIEEKLLMFSKQVQHSSFKFTLCGLFNIDAGLLFNMIGSSTTFIVIMIQFQETITPTICVSNKKAMF-

>TcasGR154

MSTQDIYDAVYPLLLTTASFGLSAIFVETKNNTRKLAVFNLLKILNIIYLSLFSALLYVAFTSLKNTHLHVNYNSGVTKIGIIFQILANIMATYVIYFINISKSHNILTCIENIKKADLMFRDLGEKIIYRKHFLYEVCMILFGVLAILGRSIVTHIYMGRNIFLTENETHFALFFPLFVSYLVQINFVLLITLVHERFALINRLLDNFNEEKVKHNYLSLKPPYEYQTKYNQAKIELLMELHDFLTDVGNKLNDSFSIQILSCLTSQFLTEVFTIFYLYYESLILKNKIAALVWLMWSIWTTLEIFYVTVNCHLTTKEAKNTGIAIHKVLMNESDPDAKRKLMVFSQQVNHRSLQFTACGLFYIDATLIFTIVGAAATYLLIMLQFQEGIEAQCSNNTLLN-

>TcasGR164

MSAAHDIYSVVKPLLIFSKVFGLYPNTVENFETQQLTSPWSNGLDIVWSATIMIFLTFYSIYTMRMGEKPATSTELIVEIGDIIYMSVSLIDAFLLSLFSITKRTKMVQFLQKFANFEKKMQQLDLPLDLANEYTRAKVCVLFAIATAISLIISYVIIDIILFLGNVTFEDIGAEIVAYVYPLVTMMTMVMQFCTLCLLTRQKFRWINLKLDQIRKQWQGKNKTTYYSNKIKFVSPKETTKAITLEKLIYFLEQFRRRHYELCSLTENLNRIFNVQILFTCLNLFITITFTVYFYFISNSDAKIRSPVLYSTYYALNGFINVSALFGLVWAASQTKNEVKSEPKKTLSLKTNIERRSGKLGADVKSPSQFKQFQFPDNAFLVPTFAKQCRIFRLSCVVGAITTYLVIVIQFQYQN-

>TcasGR166

MLPKQFLKLFKDPCDVYTAIHPLFYVCTFFGLAPYSLVRVENGKKVFKFAWWPLTRNALLVLILLGALTYHAIFDLISFKDSDLQQKLRYFEEVFSSLLSCCSVIFGCIFALKVIEVFKNIEEVDVAFRSLAVWVPYKHLYVNILIHLSGLVTIVATLTVTIIFFASYQYGTKTYSLFIVFMTVILPYFINLLMELQYCHYLNILRVRYQLLNEYLETLVQETNRTSVEGWTDVSNVKRKSKEISKLPKSMLAISDPVFIVDQVAALHIKLTDTAHMINYAFCVQQLLRITVAFISIVTALFLVAINFNKSSSEENEGKTTQLDYFFTFWAFSNACEVMAIVWITSETCEEANTCPRILHKIRNNTTNTNLQDTIEIYSLQMYHNRLYFTVCGLFPLDYTLLYTIVAGVTTYLVILIQFNNSDFVQRNSTEFDNATESY-

>TcasGR168

MPLLDEIPFYWKYIYKIIGIIQFPTEENASNIVPHLWPLPQNCLFIYICVKYYVSINHLKFLGIFYFVDALTSIGTALSLVISITIFIQRSRDLKKLLLQLKEIKIDSVHRKTRSKANHYLRIILIITIFYYFLFIPFESEPLFYTLFFIYPGIILLFDHIFLSDILEIICNEFEQINREIKYQTTSHRIIFKTKKINDEEINDKMVRAEQLSLCHCDLTALTLNICHHFETTIIVAMITSFNYVTSTVYFLIYFLARSYEANLFVLFGNNFAFLSFYVFWLLLILEMFTRTEKEANITGRYIHDIWNKFEANGNMTKKLRHLQLVSIRFLNNKLEFRARNFFRLDWSFCHTMIAAITTYVIILVQFHI-

>TpreGR1

MRLLPRTAVEKPLYLTYLVNWLCGIGVIEYPMGRPRPWLSFGYSGGCLVVYCTLAVLAAPELAHCFPAEMLLPTTILFYSHIVLTVSTIVLGWLRSEGMRACILNLAATDSLMDRFEATSKNYSRVAPCRMLEIVARLAMVLFIMILSSYLFYEEDTPSRTRVLVSVVLSYPVMLMFVADTTFINIVNCVTYRFRNLNDLLENMLSESNEFRRSDRSKRSFEEKYTKWNMNISESAKKDQWNNIKLAKKIHLALVNICQEADRSYGLQIILSSVVAFAIITGNMYVNYLVLVELHLPRQIMMKSIIGGAIWIAYYTLKIRCFSCVCTRCVEQSVTIGDIVNKFYDDPVIQLETQAEIRDFNIQMIQQPLKFTACGFLTIDFTLVQGMTATITTYLMVMVQLRKSSTSYAMMNAINSTK

>TpreGR2

MKREIKNIVEAYQPLLWLNRILGLAVFEVPIGRVWFFFSIFYALIRSLAYGFLLWYAFLLIPPMPLSYHSVMLMFRIIVYINVVIASVTTILGIYNHKKTKKFFKNVSMIDATLECFEIEPVYYDDLKENLRLVLAWLTGVIIIFTCDLILIYAIFDHLGHALAVVVAFEIPLQINSMIEVNFIVYIRTIGRRFEKLNELIKNITTHPSQNDLQHVKSLDLLSPKKTKKNKNKISVRSSSYIKNKYDVEIILKMSRQLHLNLCTTSREVNQTLSKQISMQIASSFFILTGFGYCIYLVYHLPNVSLPRKIQLFCSLGAWIVVIVWRMLQVVRTTVKVSSEAHKVSQIAHEIHVTKFQSKLVDDIHQLSLQVMQHPLFFSACGLIVLDFKYVRGFVGSVTTYWMILIQNQPDMIRAANVLVSSIDDNSTLSTTAP

>TpreGR3

MRPHRIDPLANSLTTVMNNNNLNNNNINNNNNNNGSICDNNAEDSPADGKDYFYQPAWAIISRRNVFNQHQHQHHQRRAPGGSAEIQEVVEQQSNRREMHKLDTMPMEGSTQPSAPSNSGGGGLDDPVDDGQRSLHCALRPIIILAQVFAVFPISGVNSSDASTLEFTWRSPKIVYCCLSSLGSLVLTLFSIYRLATTSITSSKTSNLVFFFTAGITTILFLKLARQWPAFAATWQSMERELATRYTRTTTTTTTNTAEEGENRGDDNPSGLDALSLSAKFKVLSTVVMSLALVEHSLSLLSGYVSALECASLRGHANTAATYFSLQFPQIFTESNFALWKGALVQFINVLSTFSWNFMDLFLILLSVALTDQFKQLNRRLHSIRGKHGVAVKTMPEWWWAEARIDFNRLASMTRRVDSQISDIVLLSFSTNLYFICIQLLNSFKPMPNAIQTIYFCFSFGFLLLRTAAVSLYAAAIYDESRLPAPVLYGVCSSNYSTEVRRFLHQVTTDSISLTGMKFFSITRSLILTVAGTIVTYELVLVQFNAVQADHLQSDSNITKVCEVK

>TpreGR4

MQQAELEEDPYELKRIIVLDNDKNTMANGDAADGTDHNHHHNHHHHVTSNGLIGKSDVANGRIFSKAVVTTNNNNNSNNSKKRQQVGSDEPTLFAKVHHRHDRAEAARGALDARKLSTNSDDPECFHRAIGPILLLAQFFGILPINFIRAESITKLSFRKLGPRVVYSYCVLCCIVFMTSVSFLHLFVTLNASSFQTRGGIADATAGAVFYGNSLLGNFMFLRLCPRWIAVQYDWRAMERLLDKVKTKRPRLRWRFATIAAAILGLALVEHLLSMVNNTPASVVGGNHSFEEFLAVYTQKSHGFIIKHVDYNFTLGLFIFFISKISTFTWNFTDVFIMMVSTGIAERYKVLNARLVGLTTSQLTVSDWHDLRECYASLSVLVKKIDDEISGIILLSFGNNIYFICLQLLNGLSPSGDDASLVNSIYFFGSFIFLIGRTISVTLLTARISDQCKVILPVLYNCPSANFCMEAQRLQQQIASDDVALTGLRFFSITRNFMLAVAGAIVTYEVVLLQFNIALQREEDFLEEQLLYANLDDDDD

>TpreGR5

MTARRFTAVRSNSDDCTHSAARDPTTNNEYSQIGRYGCLVHQSQRSEADRRWGSRSAKAAAATATAATPWTQEIDDGGDGGNKSDVFHTLAPVYHLSKICGLLPVKFKVNKAGKYEGRLDVAEVVYGIVLVAALAGAQCYGLYRDLRNGWENSTRLSSETAITVTCSDVFAVISAAFVAILGSGYRWHHLQDALNKIVDVDDKLLDVPTSERLRRVSIIVIVASLVYIVVISSLDFVSWRASSAGKNNAHFGDKGPINYAPIYFMYIVVTVFEVQYALVLFNVGERFLKLNKSVANLTKSNMALEQLFRRFTHRHQQQEQSMAFFSNEIGHIGRFRRANNNNKISDFGPGAGIGTEAPSKTAELIGRLIGLHGILCDSVKNINKAYGGAVVVGTISCLIHLIITPYFLYNEIFSDSISSWYVLTMQVFWTFFHVCRLLFLVQPCHMVSVEARKTGTLVSQALASNWQPEAKKQLEIFSLQLLQRPVEFTACGLFFLDRGLVTSIAGAVTTYLVILVQFQNADETKGTKNMLQNATELLKNASSFKNITFKVR

**3. IRs**

>AmelIR1

MSTTLVAVCALLLTFLGVSICQISMNMLIVIEEPDKSILNILNEALPQAEKNYGNDIISVHISTIEVERSNTDASFKKVCAALFKGISIVLDMTWTGWDTLRNKANENGIIYKRGDSNINPYIQAIDDLLMLKNATDVALIFEDERELNQSLYYLIGNSILRLIVIDEFTEKTVSKIKSMRPSPSYYAIYASTAKMEDYFRTAVQGGLVKRNGIWKLIFTDNNYKDFKYINGDLQLNVSITVLWMKMDVCCRLIGESLCNCPSNVKIFSNYFKRLVGLIVSLMSELQASGVSVEPKSVKCSSNANQSSNVTIEAFNKNIVAKLGGNDTFEYWPEKGMITYKAEIELKILENGLLEPLATWTRNGKIKEAENKKILPAKRFFRIGITPSVPWIIPKIDPATGKVMKNENGNDMWDGYCIDFVKKLSEEMQFDYDLIIPEDRQFGKKLPNGQWNGLIGDLAKGETDIIVAALTMTSEREEVIDFVAPYFEQSGLLIVMRKPVRKPSLFKFMTVLKVEVWLSIVGALTLTGIMIWILDKYSPYSARNNKQLYPYPCREFTLKESFWFALTSFTPQGGGEAPKALSSRILVAAYWLFVVLMLATFTANLAAFLTVERMQSPVQSLEQLARQSRINYTVVANSSQHQYFINMKNAEDKLYTVWKEITLNNTSDEVEYRVWDYPIKEQYGHILQAITQVGPVANSVEGFRKVIESENAEFAFIHDSSEIRYEVTKNCNLTEVGEVFAEQPYAIAVQQGSHLQEEISRKILDLQKDRYFEMLASKYWNQTQKAQCLNSDDNEGITLESLGGVFIATLFGLALAMITLAGEVFYYRKRNTETEKSTKDKKRKVKNKIIQNLTKMSLQMKPAPINPFFEKTNNPPRVSHISVYPRNLPFKE

>AmelIR2

MLLVTLFLQFIVLASSKRVLYKLHQCENNEANLKSLAEEIVEEIIEQTNCIIFITDSTYQNLIDIKNIKGSSNVSKYEILLRDNEQFSRPRRRIQRILVDGRTVDCNAYIMLISNGYLTAEFLQYTERERLINTRGLFLLLYDLRLFQLNLYYLWKKIINVVFIRQYNAYKHRSGEISFKERIDLNTVYFPPRKRRLTATKYIDTWYQGKLRYGTNHFTEKTNNLQKKHLQIAVFEHIPAVTEKSKLYYNKQPNNIIQGLGIEFELIQIISKAMNFKPKYYIQQNIPLKQKDIEGSNQTDTGLISKVIEENAAFYLGDLHYTLQNLNYLDLTIPYNIECLTFLTPESLTENSWKLLILPFKFYTWIALILTLILGSIVFYFLSLSYKKHISSYKSQNTSIKNETKGLYLFTEIGNSILYTYSMLFQVSLPHLPSPWAVRILIGWWWIYSILVAVAYRASMTATLANPVARVTIDTLAQLAKSSMEVGGLNEESKNFFLKSSDLSSQEIGNKFIIIKHEDEAIEKVANGSFCYYENSYFLQYARVKRQIFEKEKKRNETANNRSSKHNLHIMEECIINMPIALGMEKNSPLKPKVDILIRRMIEIGLVKKWLNDVMEWPKIMEIRQEAESEKALVNLHKLKGAFFAIIFGYLLAFMILIGEILYWKYIVLKDPKFDKYHLDIFYNSNNNSKI

>AmelIR3

MYVQNIFYLQLIVASYASNIDIIRDYFIFKNVPRVAGFSCGNIENDYQILKLLNEVGIGVSITQFTSIINIPQFLHTTYWNLGIFVDLECLVSDENIVKLFYETSTYYMFDHLHQWLILEKNMTHILQLLNDNMFSIITDVTIAISKDNDYILYDVYNHCKNYGGLLNITKLGTWTKNNGLQIILETNKFSRRWNYHRMKIKVAGLVVKRPKNQSLIDYLQEENLYEHTDNWSKFGYAIMKHIKQLFNFTFELIELNHWEKNDSNGPLIAGLKNGIYDLGYFPSILTKERFNYADVILQVWPIRTCFMFLTVPSLKVDMDIIFRPFARNVWYMILILIVAIILGLWIIFKLEENDSAYGSTILIIIAALCQQGLPFFNNQFSSRIAFLQTMIFGLLVYNYYSAAIVSSRLNAPLDKMNDSLYSLVNSRMKLAAYKDIYFNILLHSSVEEVQYFKKYWEKIPEKKRYLSIQDGLKKMTTAKFAYHADPMNVYPFIERVFDKQMICQLTEVHLLRPSSLGLWSTRHSQFQEITKIGLIRISTSGIRKREVIRWTYRKPYCDKDKHYVSSITIHETIPILLVLCFGIILSIVICFIENIIFHTIRKKQRQIKESEF

>AmelIR4

MHYSQVSSVIYCANNTRGYFSLSIEMISVLLLVWWINYGSSYNNFPSLITSNATMAVIIDKGFFSNKDEYQNATKVIQDLITDAVKKEMNLGSISIRVFRDMNVNFKDYTILLSVATCYLTWRLHEVAQKEELTHFAITDPDCPRIPDTDGITVPSIVPGEELSQIFLDLRMTDILSWNVINILHDDTFDRDTISRVLKAISNKLPNKRMNLISRSIFSLRYGNTGSGRKSSVKKMLNDFHVEQLGHCFLVIATVDMVADVMSVANSLNMVHPGSQWLYVITNSVSGNLINTSFINLLAEGGNVAFMYNATNLDGFYKIKLKCYIKDLIEALAKALEYSLKNEIELFKRMNEDEFEMIRLTKSKKRAELLKNVRIHLSRNTSASNSVCEQCLLWRFFSSITWGNFFSHDRNMAHLLDIGTWTPIIGVNLTDVIFPHIVHGFRGINLPIATYHNPPWQIISMSKTGKKLYEGLIFDAINYLSMKLNFTYTVIMPETSQISRSWNTSQFAKLGEKIKEMTMSTTKKVPLEIIDLVRQKKVLLAACALTVNECGNTTFNYTVPIFVQTYSFLTAKPSQLSRVLLFASPFTKETWACLAVSIIIMGPILYLIHKYSPYSTKASGLNSSWQCVWYVYGALLQQGGMYLPQNDSARILIGMWWLVVMVLVATYSGSLVAFLTFPRMDTSILSVEDLIAHKDSISWGFPNGSFLEMYLQNAEEPKYHVLFSRAERHNDTEEERLVERVKEGKHALIDWRSSLRFLMRKDFLLTGSCHFSLSMDEFLDEPIAMIIPYGSPYLSVINAELHRMLESGLMNKWITEKMPMKDKCWEAPGSNQMVNKRKVNVTDMQGIFFVLFIGITLAFFFLFCEFYCHRRKIAKERKLIHPFVS

>BmorIR7d.1

FNLPLKLTILSLFLGIILLNMLRKTIFFNNIRRVCNITPPKRNSLFYAWLLFLGLPLEKFSSRKHFKIIILAWIWFSFVIRCAYQVTLVTSLKSITYNYNLRYDSDILKYPFGGMSSIRDYFIEDKDFYENWTSVDMQKAYKLLDEIMEEKTDFVLALNKDTILHHAAEHIGSKRIQVIDNCIVNSPIVLYFRKHSPMTDPIAKIMNAALECGFIQYSYQTNWKRQKHLLNSHYAYNLQPLTLDNFSGCFFLLIIGYGISILYFVLEVVCHKIDKTNQRIDLRVDQE-

>BmorIR7d.2

MSPRNLSDASHFCEENSNEITTAALNIALHNFKWRILTYVFFNATFLCNLNIFLKTYNKGVVVGNGLVEPRIDGKIQQLVLFCDDIVGITLALNSLPNQFDETGKVIVICQSPISWKCSAEEAMRSFWSVKITNVVFLKKDVFVMAYTYMPVYNEQCEISDPIPLFGLKPCIINATKCGVFDKKLDNLNKCKIVVSTLIRRPFMIINNGIPEGADGDLLLLIMERLNATLEVIIPGDHNYWGKLDSNGTWSGSLGDVYYGAADISMTSAALTASIISYFKISIPYRSTNVVWISHPPKALSPALKLLHPFKPSTQIALGIIFFIVIACVLFVSSKKMWLLCCRRVRPTKKKPSLLFNTWMICIGVPIAHLPSTSTFLSLIVLWIWYCFLIRTFYQVWLINSLQGKFYLDGFEKIDEAIEAGYDIGGGIFLKEYFVDYPYIYNNWKETVSLNVTLHEISEGSNFIAATIYDLAKSLTNFEKINVHFLAEKVVVSPSVLFFNKNSPLVAPINELLQQLTESGFVEKISRNYFTHNVTNWKRQKHLLNSHYAY-

>BmorIR7d.3

MRTEPEDITLFLQHFHGSAVIVPLDYQNMKAVSELNKATGFKQTVLFAVSVEEFILFITTLNLDLIVPIRMVLVLTTQLTDLAMITKEAWKHDLAEIIIISKDENEEIRLTTYFPYKNGICGDYTPHSISNEKELFPEKFKNLHGCPIKVTLLNFLPYVGLQKVNGTITFIFGIDGSVFILLIKELNAIMDIVSSTDHGGMGVFVNGSWKGSFGDIVRREADIFAPAGIITQKRFSVAQMSHTYETLNIHWCAPPRREIYAWAKVLLPFLTNITPFLVLAFTVFVITIVLVKRSKLHGIKSNKNVFLQSFMIFLGQGVKFETKSSVINSFFVAWLWFCLIVRIAYQGDLVNGLQKKIYEPPFESVEQALQELDGYGGTELFREYYAGSPIADNYQVIKIGDLPRYIRDVIAGKRFLIATDILMHQYAKKFQILQEPLTHSPTCLFMRPGWPVSRRVDVIIIRAIEAGLVQKIIYDFHYTVRLRRHEKEEETGTRPLGMSTMFACYYGLILLWIFSFVIFLFEVLYYNWKHKIAYIKRKRNKLFKFHH-

>BmorIR8a

AVPWTLPKLDPETGDPLYNEDGQPIYEGYCVDLIQKLSEAMNFDYEIVSPRSGGFGRRLPNGSWDGVVGDLTTGETDIAVAALTMTAEREEVIDFVAPYFEQTGILIAIRKPIRKTSLFKFMTVLRTEVWLSIVAALVLTGFMIWLLEKYSPYSAKNNPGAYPYPCRDFTLKESFWFALTSFTPQGGGEAPKALSGRTLVAAYWLFVVLMLATFTANLAAFLTVERMQTPVSSLEQLARQSRINYTVVEGSSTHQYFINMKFAEDTLYRVWKEITLNATSDQAQYRVWDYPIREQYGHILLAINASGPVADAETGFKQVNDHTDADFAFIHDSAEIKYEVTRNCNLTEVGELFAEQPYAIAVQQGSRLQEDISRALLELQKERFLEQLTSKYWNETLRQSCSDADESEGITLESLGGVFIATLFGLGLAMITLAWEVFYYKRKEKNKVQSTKENVERPPIKSAKLGGKMAVGVARLRKRATKIGKKKNVTIGDSFKPSVSYISVYPKGDYR-

>BmorIR21a

MDRRSLYGLLFIFYIISSQEIISYHSESLLKNASRNLLWNKKITSIIKEHNDFAYHYESDLHFGNRIKNVKSKRAVDPVFHGHPKTREELWYERFLNRSSVFDQTPSLIKLIQNITLTYLNECTPVILYDSQIKLKESYLFQNLLRNFPVSFVHGYINEHSQLQEPKLLQPVRECLHFIIFLSDVKVSAKVLGKQSESKVVVVARSSQWAVHEFLSSSFSRGFINLVVIGQSFKEDDDSTIESPYILYTHKLYTDGLGASKPVVLNSWSHGKFSRNVNLFPPKMTGGYAGHRVVVAAANQPPFVFRRIFYKKIYRIKSDLDGGNPRVVWDGIEIRLLHLLAEKNNFSIEIVEPQELHLGSGDAVAKEIAKGRADIGVAGMYLTIDRTREMDVTFAHSQDCAVFITLMSTALPRYQAILGPFHWHVWVALTLTYLFGMFPLAFSDKHTLRHLINNSGEIENMFWYVFGTFTNCFTFLGRNSWSKTDKITTRLLIEIGKIFPCYYFRMVLDFTIIITSCYTGSIIAFVTLPMFPETVDTIHQLLAGFYRVGTLDRGGWERWFLNSSDPNTNKLLKKLELVPNVEAGIMNTTKAFFWPYAFLGSKAELEYIVQSNFTKTTSKRAVLHISNECFVPFGVTIGFPNNSLYTAKLNNDLRRMVQSGIVDKIVDEVRWEMQRSSNGKLLSAVGGSLKVSAAEEKGLTLEDTQGMFLLLAAGFLIAATALISEWIGGFSKLCRFRKKKNTLVNSSTKEDSINMPPTDSKDFKTETESVLHFCSRSTSPGSNESLDGQIINVTEESIEIHKQFTSEWDSRRSSSVDLEKEVKEIFERDLRRRGAALXXXXSTASNNAFGDAVK-

>BmorIR25a

MCRSSHIFQNSMPLFVVFLQFFIFRLIVSQTTQNINVLLINEENNALAEKSFEIAKEYVRRNPSLGLAIEPVIVVGNRSDAKTFLENVCRKYNDMLSSKKTPHVVLDFTMTGVGSETIKSFTAALALPTISGSFGQTGDLRQWRSLNANQTKFLLQVMPPADILPESIRAIVTKQDITNAAIIFDELFVMDHKYKSLLQNIPTRHVITPVKSFNKEDIKTQLRSLRELDIVNFFIVGSLRTIKNVLDAADENQYFGRKTAWFAFSLDKGDITCGCKDATIVYMRPTPDAKSRDRLGKIKTTYSMNGEPEITSAFYFDLSLRTFLAVKSLLDSGKWPNNMKYITCDDYDGKNTPNRTLDLKLAFQEVKETPTYAPFYIPGDDPMNGRSYMEFSTDLSAVTVKDGASIGSKALGTWKAGLNSPLSLTDSDNMSDYSAQLVYRVVTVEQQPFIIRDDNAPKGFKGYCIDLIEEIRQIVKFDYEVTLSPDGNFGTMDENGNWNGIIKELIEKRADIALTSLSVMAERENVVDFTVPYYDLVGITIMMKLPRTPTSLFKFLTVLENDVWLSILAAYFFTSFLMWVFDKWSPYSYQNNREKYKDDEEKREFTLKECLWFCMTSLTPQGGGEAPKNLSGRLLAATWWLFGFIIIASYTANLAAFLTVSRLDTPIESLDDLSKQYKIQYAPLNGSAAMTYFERMAAIEVRFYEIWKEMSLNDSLSDVERAKLAVWDYPVSDKYSKMWQAMKEAGLPNSIEEAVQRVRDSKSSSEGFAWLGDATDVRYYVLTSCDLQMVGDEFSRKPYAIAVQQGSPLKDQFNNAILQLLNRRRLEKLKENWWNNNPKAMKCEKQDDQSDGISIQNIGGVFIVIFMGIGLACITLGVEYWWYKWRRRPIVGDVTQVEPAKSTRNNIGNFVKGEGFTFRSRNFGLSDLKQKF-

>BmorIR40a

MTKLPKDFNVAIKDIAESLPSKEMTVVRGNSTNIRSQDVFELLRLLCQHNIQVVNLDIAAMENKEMYYGYLKKALDVSDERTNLILCEPYECENLLLELRENNLIHRTILYIFFWPYGSVSDRFLNTMVEAMRVAVITNPRESVFRIYYNQATPNRLNHLSLVNWWAFRLYKSPLLPSADKVYKNFRGRVFDVPVLHAPPWHFVKYNNDSSINVTGGRDDKLLKLIANKLNFRYRYYDPPDRSQGSGIIGNGTFKGTLGLIWKRQADFFLGDVTMTWERLQAVEFSFLTLADSGAFLTHAPAKLSETLAIIRPFRWEVWPLVCATLFITGPALWIVIAAPSLWQRKKRDQMGLLNNCCWFTVTLFLRQSSTKEPSSTHKARLVTVLISLGATYVIGDMYSANLTSLLARPAKEPPIGTLPALEEAMREHGYELVVESHSSSLSILENGTGVYGRLAKLMKRQRVQRVHNVEAGVRLVLNRRRVAVLGGRETLYYDTERFGSHNFHLSEKLYTRYSAIAFQIGSPYLETINNVVMTLFEAGILGKMTTDEYKNLPEQSRRSEPVTESENLSTEKTGETAAVTQIQNETSKGLEPVSLTMLRGAFCLLGIGHLLAGVTLLIEIQLYRRARKRALPPQTRNPTNTFKAKAKKCILRGWRRIKAAAILAIDRALAPDRGID-

>BmorIR41a

IEILLQIIINKYLSESYCLVVISETPLSVKLPMSFTYLDPKKEHFSVETLLKLSEEGCSDYIIRMEDPRQFMNALEEIRPMSMVRRSDKKLVILPVTDDENSMEPILNLLTMKESSYYAHILLILPTQTERFECLAFNLITHRFVGSDSESKLPIILDRWYSCTNHFENNVYLFPNDLKNLNGKTMKISTFIYKPYVLLDVDTAVAPLGRDGIEIRMIDEFCRWINCTVQIIREDVDLWGEIYENETGIGVIGSVVEGRSDFGIAALYSWYEEWKAMDFSVSVVRSAVICLVPAPRVLESWELPFLPFGKSIWIAVVITFVYASIGLTIAQGCSSNKALLIVFGTIISQSQYIVSDSWRIRSVIGWLLVSSLILVSAYGAGLASTFTVPQYEPSIDTVQDLLNSRMEWGANHEAWTFSLALSSEPVAKKLIKQFKIYSFEELQRRSFLRKMAFSLEELPAGTFAIGEYLSKEAVQDMQLMLEFFYFDHCVAMLHKNSPYTEKLSELIGRLHQSGLLLAWESQVSLKYLDYKIQLEIRLSRARSDVGDLKPLNFNHVEGIFLIFITGTILSTLFFALEIFIGKQARKK-

>BmorIR64a

MNILGLNIISFLCSLDISSVIEVFKCKHVRDVIVFHCFKENQLILPQRMFHFNNFRTVFVFISNNISWELPNSYPKIGVLINTSCDGWEKFQEFQNSHTWVYYTDNLTSTITALSTFPIEINSDVTVVYKENSAYQVFDTYNTGRKNNGVFNVNYIGHINPGLQTNLKFTTRNLNGVTLKSTVVILKKVQYESFEEYLRKTEQTGLDSVHKHKFFQLLQYISEMYNITYDLIRTNTWGYAHDGRIDGMVGSLQRHEADVGGSPIFFKTDRAYVVDYVAETWPSKQSFIFRHPKHPTGVHTVYSRPLSNSVWYCVIAFLFVTASTVFFMLKFNIDEIERAETSQSLAFLFAWSAICQQGMSLRRNSLALKVVVFVTFVCSITLYQYYNATVVSTLLKESPITIRTLKDLLQSDLKVGVEDVAYVKDYFAHTKDPIAITMYEKKIVTGNNRNFFDPEYGMSLVKKGGYAFHVDTVYSYGIMKKTFTEREICEIHDVTMYPPQKMGAVLKKNSPYRNYFAIGIRRLWETGLMQRMKHIWDEPKPPCVRTQDSSIFSVSILEFSTPLFIVVFGVIASVVVLLCETLFDTLFNMR-

>BmorIR68a

TIMPLTLFSRSPRSGKTRAVSKASPILEDIYEQKDLEFVLVDLLNHAGRYHDFTCVAVICDAIYYNVFDGAFFKRIDTVPFVMIVVEEYDDLLSPNFDILEALREARRDGCNMYIILLANGLQAARLLKFGDRHRVLDTRAKYIILHDYRLFHSDLHYLWKRIVNVIFLKHHRKIGSVAKSQAWFDLSTVPFPNPIKGVFVPRRVDLWKSGKFHYNTVPFDDKTSNLNDEVLHVVYLDHVPSVVVVNSNETGQIGGVEIEIINTLSEKMNFRPKLYQPMNVELHKWGQKQPNGSFSGLLGEMVNGRADLALGNLQYTPYHLELIDLSIPYTSQCWTFLTPEALTDNSWKTLLLPFKLYMWIAVLLVLXITGTIFYGLARYQTYLHGLKRQEEMKKPVYSKPVGLYLFGEIINSILYTYGMLLVVSLPKLPTGWSIRFLTGWYWLYCILLVVSYRASMTAILANPAPRVTIDTLVELAASKLTCGGWGIETKNFFQDSLDEIGQKISDRFEISNDPNIAADKVAQGTFAYYDNKNFLKYITVRRQNGFIMETIDNTTNFTSISTKSNNERNLHIMSDCVVNIPISIGFHKNSPLKPLTDIYITRIVEVGLVEKWLNDAMYTIKTLETNEEEIKALMNLKKLYGAFIALAIGYFLSVMCLIGELAHWNCVVKKDPNYDKYALHKYYEKINKK-

>BmorIR75d

VNIQNNFANAPELPAYDFRREGVVLDLNCPNSKLILEKASKNRAFIHRYTWLLIHNSTYKLETIQKILSDAAVLPDADVTWCAADDILDIHRLNEHQPYVVMDLGLSVNSTIEDLDAVWSTIPTAATRRRHLNNLTINAVVIVSQPQYFKGWSDLSNRQIDTFPKLTYPMLMLCAEDLRFRFNLKQVDEYGVELNGSFTGTVGLLQRGRAELGVASMFMRSDRWRVLHFSSATVALLNAFMLRAPAQSSVSNIFLLPLSRGVWCCAAALLCGSAVLLAVLSCRLVAADPTLQLLTLPEIFVFSIGTVCQQGFYIMPKLSSIRMIMFLTLLTSLFTFTAYSAKIVAILQTPSAAVRTVADLADSHMDVGIQETTYKKVYYAESTDPSILRLFHRKVAPLGERVYMSVVEGVERMRTGLFAFQVERSSGYEIISKTFTESEKCGLMEIEAFKLPMVAVPLRKHSGYRELFGTRLRWQREVGLMSRVRAIWLAARPRCEGRGVGFRAVRLLDMLPALQMLAAGGLVAVVLLILENVYHHYSRTGNVLRRTYRNVFKLVCYKCYTF-

>BmorIR75p

MVDVRPHRELFKRRRDVMGRPLTMANVIQDSNNTRYHLPREDALELQYDVIPKICWMTAKLAFQMLNATPRYTFSYRWGYKVNGQWSGMINDLHTSKADLGTNCVVSDVERLSVVTYTDMLAPFRVRFVFRQPPLPSVANIFYLPFTGRVWAAVAVCAMVYTAAIYWASKWEFNLEKRSASQFDGTVGDAMLLTMSALSQQGCFIEPKRAPGRIMLFVLFTALMALYAAYSANIVVLLQAPSNSITSLAQLAASKVTLAANDVDYNHFVFSLYKDPVRVMIHKRIDPETGNGQFYSLEDGVDMIRQGFFAFHSIVEPVYRRIEETFLETEKCDLTEVDFLSSFDPFVPVKKDSPYLELLRVVKKKKIRCSFKQIRESGIQSALNRRYQVPKPRCSNKVAAFSSVGIVDLRPVLIMMIYGIISSCLILIMEMLVFKM-

>BmorIR75q.1

IFKIKNPVAITFGKIGNVEEVTPSNHILFLVDTTCNNSHIVLQEADAHQQFRRSYRWLVLETQGSGYNSKLLEIEPLNILIDSDVLLATKIENVTYVLKKIYKISTQSEWITEDYGNWTAEHGLIMSNIISSDASRRRNIRGHPVTTSIIVTENRTKSELDDLKNLLSDSLAKICFRHTKNLCQFMNASHKIGFASMWGYKTNGTWNGMMGDLAKGTVDFGGTIAFLTSQRLQVVDYLSSPVPINAKFVFREPPLSYQNNLFLLPYKANVWYCTAAFVVLLVIILYINAKWEIKKAEYEQAVTLQPSVSDVTILVISAISQQGSSNELKGTLGRAVLFLLFLTFLFLYISYSANIVALLQSNSKQIRTLQDLLNSNLNIXDGKPLFQTATEPIRKAIYETKVAPKGSKANFMSIEEGVKKLQKSPFAFNMNIGTGYKIIERYFEEHEKCGLQEINYIESSIPWMSCRKNSPFREIYKLGLFKLQEHGITDRENRLLFARKPVCIVRGGNVGSVNMVDVYPVILMFLYGLFLAFLILLVEIVVHRKL-

>BmorIR75q.2

MSGGFQKNTNINEVIAVRRRDLEGYEIKICYVLTDNDSIHHLSDEVNDHIDTITKVNFPSTNHLLDFLNAERKYVFVNTWGYRINGTWNGLTGFLVNGDVEIGGSPMFFTAERTAVVDFISSPTPTRSKFVFQQPKLSYENNLFLLSFRTAVWYSTLALISLIFTMLLSVTAWEWKKMSQIKTRDIDAGVLRPSVTDVTMLVFGATCQQGSTVELKGSLGRVVMLILFLTLMFLYTSYSANIVALLQSSSSQIKTLEDLLHSRLKFGVHDTVFNRYYFSTADEPVRKAIYEKKIAPPGVAPQFMSMEEGVKKMRKGLFAFHMETGVGYKFVGKYFKESEKCGLKEIQYLQVIDPWLAVRKNTPYKEMFKIGMKRIQEHGLQNRENRLLYEKRPKCSGRESNFVSVSMVDCYPALLVLSYGIIIAIALVIMENLWQYRHLIKGKLEFFSSVNTIENFNQFEKHPSHNWKKFYVIDSAKIKKINPSTN-

>BmorIR76b

MLFSNAQRSGQNFPLSWIERDENGTVQAYGVAFKIIDILQQKFNFTYEIVIPHRNFEIGGSKPEDSLIGLTNTSKVDMIAAFIPRLVRFRKLVTFSRDLDEGVWMMMLRRPKESAAGSGLLAPFNNFVWYVTLASVLCYGPCICFLTHVRSKLIKNEERPLRLSPSFWFVYSAFIKQSTNLAPEANTTRVLFATWWLFIILLSAFYTANLTAFLTLSKFTLDIETPEDLYKKNYRWVSVEGGSVQYTVKTQDEDLYYLNKMVTSGRAEFRTLSPDQEYLPIVKAGAVLVKEMISLEHLMYGDYLTKTREGVEEAKRCTYVVAPKPFMKKPRAFVYPVGSKLKSLFDPTLAYILQSGIIDYLEHKDLPSTTICPLDLQSKDRQLTNSHLMMTYYIMCVGLASGLAVFVVEILVKRYINIKIKPIDKVKLKKFKRSKRSPRYDDSGPPPYESLFVKPKFKDSEKRWKMINGREYYVYEDARGGTRLVPVRTPSAFLYR-

>BmorIR87a

MTTGNSDQIAKTAECVLKLSAKYFVERKALSGSIVIINVNSYSSTTQGLLLKTIHSSIKYSVMAKDSFYPHANASHFPEKAKNYMLILEERTELKRNIFQLNKLPSWNPLAKAVVFYQIKGNESAQRIAIEFINELREHKFFRSIIFINNGTESGVTSYTWRPYSENNCGGKCDSVYVLDRCKNNIVEQIEPQPEWFPSNMNGCPLTTYAIVSEPYVMPPIRKIPNAKFDDVYEFQKGGETNLVKTIAEFSNMTLIVRLSAIEENWGIIYANGTATGAYGVLRNDSVDIVFGNIEVTKQIRKWFHPTISYTQDEITWCLPKAGQASAWDNLVIIFQWTIWVATFTSLILMGLLFHYMYYREKNKKITKWPTNSLLMTFSMLLGWGSHFEPKTATFRILIFGWLCFSINMGISYESFLRSFLMHPRFEKQIATESDLIQSGIRFGGREIYRTYFESNDASSSYLHTEYSSTTFSEGIRRAALNRDFAVVSSRRQAEYQDQKLGKGASLIYCFPESDNLYKYSVVLLARKWFPMLERFNGIIRSVSENGLINKWNDEMFIHRVSLEGASTIVPLSIQHLLGAFMFIGFMYGTSAFIFLVEVFVGFVQRRAFLSAFFCGKKKRFSAVFKVKV-

>BmorIR93a

MKIWVLGVLCLAISVQGEDFPSLITANASIAVILDRQYLGDKYQTVLDELKDYIKELARVELKHGGVLVHYYSWTNISLNKGFLAVFSIASCEDTWELFSRTEEEDLLLFALTEVDCPRLPQRSAITVTYSEPGEELPQLLLDLRSSNAISWKSAVILHDDTLGRDMVSRVVQSLTSQIDEESARPVSVTVFKMKHEMNEYLRRKEMHRVLSKLPVKYIGENFIAIVTSDVMTTMAEIARELLMSHTMAQWLYVISDTNAHASNLSGFINTLNEGENVAFIYNITENGPDCKNGLMCYSQEMMSAFISALDAAIQAEFDVAAQVSDEEWEAIRPSKVQRRDILLKHMQQYILAKSVCGNCTLWRALAADTWGVTYRQNDVPEQINEHANGSTGVIEHLELMNVGIWRPIDAMTFADLLFPHVHHGFRGKELPIITYHNPPWTFLQANESGAIVKYSGLMFDIVNQLAKNKNFQRLPHPSNRNALLLHGRNRQGGGTYPCGLTKGPITYNNIPLYFRAVFIAHQAGVNLKNNYYRCINYTIPVSTQPHTFIVARPRELSRALLFLLPFTTDTWLCLGFAVILMGPMLYIVHRLSPYYEAMEITREGGLATIHNCLWYIYGALLQQGGMYLPRADSGRLVIGTWWLVVLVIVTTYSGNLVAFLTFPKLEAPVTTISELLKNSDAYTWSVTKGSYLEMELKNSEEPKYKRLIKEAELLKETGGIEGTIHAARGTLDRVRGQRHLIFDWRLRLTYLMSADHIATETCDFALAVEDFMEEQVAMIVPAGSPYLPVINKEINRMHKAGLISKWLSAYLPKPNRCLKISTVTQEVSNHTVNLSDMQGSFFVLFLGNDKIYVYMYIAELI-

>BmorIR143

MFSVKYPRFYLFIIQIASNFGDAVTMPQLNSSINSKSATDCLIKVCYADLSFRRTVILKHVSYESDEENAFYNEIIHAVNNNNIQLVVLEEIDNLNDTINIDDADWLVVVYFKNCKALTEFNVKIVFEKIKYFIIVSDDLNEDCTSKMKTIGNVINKYDVTFVFNENKEDNFKFMTFIPQIDEETCKEIVTLPKIVNICANGQIERKSIFPSKNPKDIKKCPINVGMGSLYPFGIINHKEKYKTFDPLNETEVRGLDVDLVKVLVNQFNGTLNLYFIYKKEENPFGQLDFIPLVLNGSLDVIAGGFYRIYGNVVAYSGIYTSQAVTWMYVANRTTKSWQSLIVKIDGLYIFVIFHLIYSYVWYFVRKFDEQAVDFRNTILYSWGALVGTTSLQDALSLKQRILNLTYLIMCVHLSAYVSLHLYYFLTVLEPPELLKSNDDVMRSGRPAFLIPISKYFVLDEKYLSFANASEECTKFQDCSDLSLLRNGVTIILQGFFLNYQARTAINYEAKVLSAAENVLTVYYEMLLRKNSPYVERLQKLMTHLFEAGIPDRFYRHAIGLTVIGKAHSACQNTVSNSYSCQSGCKITFDQFAGVFYLWLFGCVLSCGAFIFELFSKFGRA-

>CfloIR1

MTRVARQRPGDCSLAVVYEPSYEQRHAPGLRQLLKFHEQDGGFSSVTWLRFDDARGPIEPRRDVCTDYILLVEHVSSVSRVLFHNVLSNIVVVTADSPWEVKAFLRSQLARSYLHLLVVAHSTSQRTPNGTYVLYGHKLYADGSGSSEPLLLASWINDSPTVEGVEPFPDKLRDGFMGHRMLVSVVENPPFAIRRPSAQGVDDATWDGLEVRLLRLAAAQLNFTVEFAEPRSTATSPLESAKKDVLLGESSMCIGGVYMTLDLAGQFDATTAHTDDCAAFISLASTALPKYRAILGPFQPSVWLFICVSYFVLMVPLSFNSNYSLVSLVKHPKGLNHMFWFIFSTYTNSFVVKNPLLDHGLAKNSTSMLLGIYWVFTIIVTACYTGSIVSFITLPAYPSALESAKELQAYRYRIGTLDHGSWVSSFNVNATEDPFLKKLFRKIEYVPSVLEGIQNASRAYFWPYAFLASRTSLDYIVQTDFAPTWATKRTLMHISEECFVRYNVVQLYPNGSLYTRSMNDFVLRATETGLIGQMVTDIDWQLQRNTMLMNKQITKRMSQKIQVVERNLTVEDTQGMFMLLGIGFLLALAVLSVENFTGRLDKRRIARDNAIEHLTFDSMARSNVHFLQFQRAVSARRSLGLHACCLVRSILLTVTTNAPKIKVKLSNIFKGKINSLKGRPPFAYRDNNIYLLVI

>CfloIR2

MKSYRVLTVPNEPFVILDKSTRSYTGMLIDLLDKIALSLNFTYTIGTSIRDGEYGRFEEGSDKWTGLIGDLVAGKADVGLATVSVTSQRSAIVDFTDPIHPPTGLSILLQKPLPRTSLFRFFTLLDARVWLCIGALYLLTSCLLWVFDLYSPFSSRNRRAKAKEYEGRRVFGFRESLWFCMTSLTPQGSGEVPRNYSGRLVATTWWLFGFVIVTSYTANFAAFLTVSRYERTIDSWEQLQAQYKFNYTVVKNSGALAYIENRMHVEREFYEIWKSLTLSDKLTPYEKSKLAVWEYPLEDKYHKIHAAVMSHNPVEDLEEGLGRFTSKYTRFALITESTDVRFQVMTNCTFEEIDLNVAKKPFALVLRKYSPLTAKFNAVIRDLGSKGWLSELRKKWWDDNPKRKKCYDTDERTAGILIENLGGLFITLFIGVGVAYGTVLLKYLQIRWAKTMKVAPE

>CfloIR3

VIENENADFAFIHDSSEIKYEVTLNCNLTEVGEVFAEQPYAIAVQQGSHLQEEISRRILDLQKDRYFETLSSKYWNQSRKGTCSNTDDNEGITLQSLGGVFIATLVGLLLAMLTLAGEVFYYRRRNAKLEPQEHMRSREKFKGSITESDQMMIQKLASRLQLKPAPTFAFDGKSLGSKQPKISHISVYPRDPFPFKD

>CfloIR4

MSLILLAELLAVSFLAGPASVFLVQQAAAARPVNVFVINDAENDVANKSVTNALKMLKEKSPNKLGKVYVATVNASGSTETLDSICPVWKSALQEKDENIPDFVLDTATYGLGAETVNRFTAFLGIPTLSAQYGQDGDMLGWRDITKEQQQYLVQVMNPVDLMPEVVRQQCSTFNISNAAILFDDTFVMDHKYKSLLLNVATRHVMVPAKAPGIALERQITMLRNLDIVNFFILGSDGLISAALQAVNKLDFVGNKYGMFAVSMNESPSITCPKCPTGKVMLFHPQPATNQQQLSELASKGVLAQPLITSAFYYDLARIGVLGMKAALDAGEWKRPTFVTCEDYNENATLPARNLNFRKYLQQASGTGFTPIYAGFAWGENGVSRAKFDVNADIIEITSSRIVESDTVETWPAGIESPLNVKKMNAAQKNTAITSYRVVTVIKPPFVMYDHQTRKWSGYCIDLLNHITKHVPFVYDIHEAKDKDYGAMDEEGVWTGMVRELKDKTADIALGAFAVMAERENVIDYTVPYYDLVGITILRRRPKTTTSLFKFLTVLEIDVWLCIFGAYLFTSLLMWFFDRFSPYSYQNNREKYKDDEDKRLFDLKESLWFCMTSLTPQGGGEAPKCLSGRLVAATWWLFGFIIVASYTANLAAFLTVSRLELPVESLEDLSRQYKIQYAPIANSTEYRYFERMAEIEMKFYEIWKDMSLNDSLSDFERAKLAVWDYPVSDKYTKMFQTMKDAGFPKDLDDALARVRSEGQTEFAFIGDATDIKYLTMVNCDLMQIGDEFSRKPYAIGVQQGSPLKDQFNNAILMMLNKRDLERLKDKWWDYSKYKKNCPRPEEQNEGISINNIGGVFIVIFVGVFLACVTLLIEYFYFRRPKMRRKKLQEQIEQQQQQQQTDAIADDKSAQVVGNVSKSMKLNFRPAPTPVVEPTDFGARF

>CfloIR5

MLLIIAKALNFKPVFYQPENVKTERWGNMKNDTLTGLLGEAKKGNAVFYLGDLYYTREHLQILDLSWPYNTECLTFLTLESLSGNSWKLLVLPFRLYTWIAVILTLLFAGLVFFVIAIFYKHKVSRVQEMPSENARVPKMVIDCKNFKILKNQPKRVEEWKGIHLFAEFQNSILYTYSMLLQVSLPTLPNAWSLRVFIGWWWIFSILIAVTYRASMTASLANSVQRVTIDTLSQLVKSSVSIGSWNYETKDFFLNSSDFNLQRLGNRYVIVTDERDAIAAVSNGSLCYYENYHVLQRERVKRQILEAEMQKNGTHVNKHKISDHNLHVMDECVVNIPISLGMDKHSPLKSHVDKLVSVNWCNFSGIIIAHIKSFNPFVFLDKTYNRSRFCGKVVKRHKSAIKSIRTTTRRHCTESTGGLG

>CfloIR6

MLFVFPFVVLLIIRDTAGYNDFPSLVTANASMAVVIEKDFFEHVEYYHKMLSEISDIIAHVVGKTMKTGGIDFAVYGDSNVNLRRVDYTVLFTVATCESTWHLHKQAEKEKLVHLAITDPDCPRIPENFGVGLPLIVPGEELPQIFFDLRVSSTLSWPKINFIHDDTLARDTISRVVKALSVELPDERLTLSARALFSTKFERNEVAMRQRLHKILADFRVEQLGTCFMAIVTFDMVFSILEVARSLKMLHPESQWLFVISESSDQETNFTSLVHFLREGENVAFVRNATDVQPNCNMGLACHVKEMIRALAKSLENSLMSEQELYDRVTEEEFEVVRLTKAERKVEIIRNMNRELSSARSLPGSGCGGCLTWKLVSAIVWGMSFDAPESAARGGPWLQETGDWSPGPGIRMTEPLFPHISHGFRGISLPVVSFHNPPWQIIKHSNNSGPVYGGLVFDILRQLSAKLNFTYTVRLPSTLTPTASTALRSDDDHSSKDTAIAAISVGHKVPHQVAELVRSRKVFLAAVALTVGEKVHEVNFTASVADQTYALLSAKPKLLSRALLFAAPFTGATWICLSSMILVIGPILHLTVRYSPRPLDPRDKTALSSLWQCSWYVYGALLQQGGMSLPRADSARLIVGTWWIVVMVVVATYSGSLIAFLTFPRMDATIDTIDDLLARGSDYRWSFPNGSALEAYLGYAAAANERYKELLAAAERQDPTKPLEILERVKSESHVFIDWRVSLTFLMGIDLNDTGTCNLHLSADDLMSENLAMLVAADSPYLRLVDAAIKRMHESGLIKKWTEDKVVAKNKCSEGMSAIQEATNHKVNMSDMQGIFFVLAIGFTCAFATIATEYFWHRRKEAAAQKLVRPFVS

>DmelIR8a

MELPLLVLLLALRFAGSEVLKITFWIEPVQRAEFDTDIAMVLKELDALRLDVKVDDTTLTLTRSEDGLDMQRFCEILSTVGASAVIDLTYSHWEEGYNLVRSLGIGYVRLERIMRPFLDMFGDFMRQKRANNVAMVFMNARDAVEAMQQMLVGYPFRTLIMDASQTDPGQHFLERIRSLRPAPTYIALFARAAAMNGIFEKVQKADLFQRPLEWHFVFLDTRDRVFKYRRQAELCTRFTLNPRAICRSMPMPDLYCGSGFTMQRAMLLNVLRSLINAAQVSPGYPLAIYQDCNATASSSEVSDPLEKDDYNWLDMVHWSNFLAYAPPLPHIQDQFQSPVPGLTFAVNISAGYYSSEHEAKTDLAAWSSVGEMRLLNETISPARRFFRIGTAESIPWSYLRREEGTGELIRDRSGLPIWEGYCIDFIIRLSQKLNFEFEIVAPEVGHMGELNELGEWDGVVGDLVRGETDFAIAALKMYSEREEVIDFLPPYYEQTGISIAIRKPVRRTSLFKFMTVLRLEVWLSIVAALVGTAIMIWFMDKYSPYSSRNNRQAYPYACREFTLRESFWFALTSFTPQGGGEAPKAISGRMLVAAYWLFVVLMLATFTANLAAFLTVERMQTPVQSLEQLARQSRINYTVVKDSDTHQYFVNMKFAEDTLYRMWKELALNASKDFKKFRIWDYPIKEQYGHILLAINSSQPVADAKEGFANVDAHENADYAFIHDSAEIKYEITRNCNLTEVGEVFAEQPYAVAVQQGSHLGDELSYAILELQKDRFFEELKAKYWNQSNLPNCPLSEDQEGITLESLGGVFIATLFGLVLAMMTLGMEVLYYKKKQNALEITQVRPVNDSSGSGGNSSTAPPTATSTTKQAWHIPVLEAEEKPAKVSPPPSFETATFRGKKLPARITLGDGKFKPRHGLYARRNLGASDSHSGYME-

>DmelIR25a

MILMNPKTSKILWLLGFLSLLSSFSLEIAAQTTQNINVLFINEVDNEPAAKAVEVVLTYLKKNIRYGLSVQLDSIEANKSDAKVLLEAICNKYATSIEKKQTPHLILDTTKSGIASETVKSFTQALGLPTISASYGQQGDLRQWRDLDEAKQKYLLQVMPPADIIPEAIRSIVIHMNITNAAILYDDSFVMDHKYKSLLQNIQTRHVITAIAKDGKREREEQIEKLRNLDINNFFILGTLQSIRMVLESVKPAYFERNFAWHAITQNEGEISSQRDNATIMFMKPMAYTQYRDRLGLLRTTYNLNEEPQLSSAFYFDLALRSFLTIKEMLQSGAWPKDMEYLNCDDFQGGNTPQRNLDLRDYFTKITEPTSYGTFDLVTQSTQPFNGHSFMKFEMDINVLQIRGGSSVNSKSIGKWISGLNSELIVKDEEQMKNLTADTVYRIFTVVQAPFIMRDETAPKGYKGYCIDLINEIAAIVHFDYTIQEVEDGKFGNMDENGQWNGIVKKLMDKQADIGLGSMSVMAEREIVIDFTVPYYDLVGITIMMQRPSSPSSLFKFLTVLETNVWLCILAAYFFTSFLMWIFDRWSPYSYQNNREKYKDDEEKREFNLKECLWFCMTSLTPQGGGEAPKNLSGRLVAATWWLFGFIIIASYTANLAAFLTVSRLDTPVESLDDLAKQYKILYAPLNGSSAMTYFERMSNIEQMFYEIWKDLSLNDSLTAVERSKLAVWDYPVSDKYTKMWQAMQEAKLPATLDEAVARVRNSTAATGFAFLGDATDIRYLQLTNCDLQVVGEEFSRKPYAIAVQQGSHLKDQFNNAILTLLNKRQLEKLKEKWWKNDEALAKCDKPEDQSDGISIQNIGGVFIVIFVGIGMACITLVFEYWWYRYRKNPRIIDVAEANAERSNAADHPGKLVDGVILGHSGEKFEKSKAALRPRFNQYPATFKPRF-

>DmelIR21a

MSYYWVALVLFTAQAFSIEGDRSASYQEKCISRRLINHYQLNKEIFGVGMCDGNNENEFRQKRRIVPTFQGNPRPRGELLASKFHVNSYNFEQTNSLVGLVNKIAQEYLNKCPPVIYYDSFVEKSDGLILENLFKTIPITFYHGEINADYEAKNKRFTSHIDCNCKSYILFLSDPLMTRKILGPQTESRVVLVSRSTQWRLRDFLSSELSSNIVNLLVIGESLMADPMRERPYVLYTHKLYADGLGSNTPVVLTSWIKGALSRPHINLFPSKFQFGFAGHRFQISAANQPPFIFRIRTLDSSGMGQLRWDGVEFRLLTMISKRLNFSIDITETPTRSNTRGVVDTIQEQIIERTVDIGMSGIYITQERLMDSAMSVGHSPDCAAFITLASKALPKYRAIMGPFQWPVWVALICVYLGGIFPIVFTDRLTLSHLMGNWGEVENMFWYVFGMFTNAFSFTGKYSWSNTRKNSTRLLIGAYWLFTIIITSCYTGSIIAFVTLPAFPDTVDSVLDLLGLFFRVGTLNNGGWETWFQNSTHIPTSRLYKKMEFVGSVDEGIGNVTQSFFWNYAFLGSKAQLEYLVQSNFSDENISRRSALHLSEECFALFQIGFLFPRESVYKIKIDSMILLAQQSGLIAKINNEVSWVMQRSSSGRLLQASSSNSLREIIQEERQLTTADTEGMFLLMALGYFLGATALVSEIVGGITNKCRQIIKRSRKSAASSWSSASSGSMLRTNAEQLSHDKRKANRREAAEVAQKMSFGMRELNLTRATLREIYGSYGAPETDHGQLDIVHTEFPNSSAKLNNIEDEESREALESLQRLDEFMDQMDNDGNPSSHTFRIDN-

>DmelIR31a

MNLLISMFILILAAGEGEIIPSMEESVVTNFVKSLVKTKQAIVFSCLFKDFKEISLALMRINQFVSVVNLNQSYSLTSILTRENYARTSVMVNARCSGSSELLFEASENRYFNKTYQWFLWGVDLEVQSLFPLNLNYVGPNAQITYVNETADGYAYWDIHSKGRHLKSNLEINLIATLINDTLNIARDIFHLQSIDFRGQFNGLTLRGASVIDKEDIISNEQIESILSRPTKDAGVAAFIKYHYELLGLLRERFNFTVNFRNSRGWAGRLGNTTFRLGLLGIVMRNEADIAASGAFNRINRFAEFDTIHQSWKFETAFLYRYTSDLDTHGKSGNFLSPFSDRVWLFCLLTLGAFSIIWVLFEIIDYKILRIRVNSQKLEHLNQKSSVICIKTTCIERILQTFGACCQQGLDPNPVDRSVRFLVMTLFLFSLVMYNYYTSSVVGGLLSSSDQGPSTVDEITASPLKISFEDIGYYKVLFRESQNRSITRLIEKKLSSSRSLNELPIFSHIEDAVPYLKAGGFAFHCEVVDAYPVISEYFDANEICDLREVSGLMEVEILNWILHKNSQYTEIFKTAMCNAQEKGFVERILRRRQIKKPACQSLYTVYPVSLSGVLPGFVILICKSINKFS-

>DmelIR40a

MHKFLALGLLPYLLGLLNSTRLTFIGNDESDTAIALTQIVRGLQQSSLAILALPSLALSDGVCQKERNVYLDDFLQRLHRSNYKSVVFSQTELFFQHIEENLQGANECISLILDEPNQLLNSLHDRHLGHRLSLFIFYWGARWPPSSRVIRFREPLRVVVVTRPRKKAFRIYYNQARPCSDSQLQLVNWYDGDNLGLQRIPLLPTALSVYANFKGRTFRVPVFHSPPWFWVTYCNNSFEEDEEFNSLDSIEKRKVRVTGGRDHRLLMLLSKHMNFRFKYIEAPGRTQGSMRSEDGKDSNDSFTGGIGLLQSGQQADFFLGDVGLSWERRKAIEFSFFTLADSGAFATHAPRRLNEALAIMRPFKQDIWPHLILTIIFSGPIFYGIIALPYIWRRRWANSDVEHLGELYIHMTYLKEITPRLLKLKPRTVLSAHQMPHQLFQKCIWFTLRLFLKQSCNELHNGYRAKFLTIVYWIAATYVLADVYSAQLTSQFARPAREPPINTLQRLQAAMIHDGYRLYVEKESSSLEMLENGTELFRQLYALMRQQVINDPQGFFIDSVEAGIKLIAEGGEDKAVLGGRETLFFNVQQYGSNNFQLSQKLYTRYSAVAVQIGCPFLGSLNNVLMQLFESGILDKMTAAEYAKQYQEVEATRIYKGSVQAKNSEAYSRTESYDSTVISPLNLRMLQGAFIALGVGSLAAAALNNTINVRSLNSRDKFICGGPVKIWYYLVLLLWYYFNRGLVGIYQLWHKTSIRNTGKGMPFLGE-

>DmelIR64a

MHWWLLVFLPLSCQGLPEHELLELELDYGLAEPQRTSLLQSSLILQFSQDYKHIPRITYFTCQKPHLQTPNQIPNAAEHRDAFAAKNFQLIKSLYESELFVRIVLLDVLAQSPTSGRPNRPGNGPTGGFSQTPSQAQSNSEWLEGVLRMEALRQIAVVDLACGAVSRRFLELASAKMLYSEKFHWLLIEDFAWHGRTQTAEGSGKRDDGEMEEEEPPGQQIQATDDEDLPSIESFLGGMNLYMNTELTLAKRMSEAAHYTLFDVWNPGLNYGGHVNLTEIGSFTPTEGIQLHTWFRTTSTVRRRMDMQHARVRCMVVVTNKNMTGTLMYYLTHTMSGHIDTMNRFNFNLLMAVRDMFNWTFVLSRTTSWGYVKNGRFDGMIGALIRNETDIGGAPIFYWLERHKWIDVAGRSWSSRPCFIFRHPRSTQKDRIVFLQPFTNDVWILIVGCGVLTVFILWFLTTIEWKLVPHDGSALIKPKGGAPPRHHYQQQQQQEQVEAPVRPITAVSVVVSKEKVEEKQEEYEDSTPIDAGTLWQRCYQKLNKYIKDRKAKQKKAPERVGLFLESVLFFVGIICQQGLGFSTSFVSGRCIVITSLLFSFCIYQFYSASIVGTLLMEKPKTIKTLSDLVHSSLKVGMEDILYNRDYFLHTKDPVSMELYAKKITSVPTTKENEADEDEPVDPNPVSTDPAKSYRDIVHSHETGAHAKDNAASNWLDPETGLLRVKHERFAFHVDVAAAYKIIAETFSEQDICDLTEVSMFPPQKTVSIMQKNSPMRKVISYGLRRVTETGILTYHFNVWHSRKPPCVKKIETSDLHVDMDTVSSALLILLFSYAITLMILGTEILYSKWHNRIQLKWVGAT-

>DmelIR75a

MQLVQLANFVLDNLVQSRIGFIVLFHCWQSDESLKFAQQFMKPIHPILVYHQFVQMRGVLNWSHLELSYMGHTQPTLAIYVDIKCDQTQDLLEEASREQIYNQHYHWLLVGNQSKLEFYDLFGLFNISIDADVSYVKEQIQDNNDSVAYAVHDVYNNGKIIGGQLNVTGSHEMSCDPFVCRRTRHLSSLQKRSKYGNREQLTDVVLRVATVVTQRPLTLSDDELIRFLSQENDTHIDSLARFGFHLTLILRDLLHCKMKFIFSDSWSKSDVVGGSVGAVVDQTADLTATPSLATEGRLKYLSAIIETGFFRSVCIFRTPHNAGLRGDVFLQPFSPLVWYLFGGVLSLIGVLLWITFYMECKRMQKRWRLDYLPSLLSTFLISFGAACIQSSSLIPRSAGGRLIYFALFLISFIMYNYYTSVVVSSLLSSPVKSKIKTMRQLAESSLTVGLEPLPFTKSYLNYSRLPEIHLFIKRKIESQTQNPELWLPAEQGVLRVRDNPGYVYVFETSSGYAYVERYFTAQEICDLNEVLFRPEQLFYTHLHRNSTYKELFRLRFLRILETGVYRKQRSYWVHMKLHCVAQNFVITVGMEYVAPLLLMLICADILVVVILLVELAWKRFFTRHLTFHP-

>DmelIR75b

MNFSVLESHFKEAQIFVDADVTYVTHDPFSKNFLLYDVYNKGRQLGGELNITADREIFCNKTNCRVERYLSELYTRSALQHRKSFTGLTMRATAVVTALPLNVSIKEIFDFMNSKYRIQLDTYARLGYQARQPLRDMLDCKFKYIFRDRWSDGNATGGMIGDLILDKADLAIAPFIYSFDRALFLQPITKFSVFREICMFRNPRSVSAGLSATEFLQPFSGGVWLTFALLLLLAGCLLWVTFILERRKQWKPSLLTSCLLSFGAGCIQGAWLTPRSMGGRMAFFALMVTSYLMYNYYTSIVVSKLLGQPIKSNIRTLQQLADSNLDVGIEPTVYTRIYVETSEEPDVRDLYRKKVLGSKRSPDKIWIPTEAGVLSVRDQEGFVYITGVATGYEFVRKHFLAHQICELNEIPLRDASHTHTVLAKRSPYAELIKLSELRMLETGVHFKHERSWMETKLHCYQHNHTVAVGLEYAAPLFIILLGAIILCMGILGLEVIWHRHCTLH-

>DmelIR75c

MTSWPLYRLIVFNLLEINLSNLMVFHCWSIKEAFPLVEMLNQNGIFSQYIDVQNPDNLANVHKEYLDSDLVSLNADVTYVSREDEERFILHDVYNKGSHLGGKLNITVDQTLQCNRSHCQVKEYLSELHLRPRLQHRMDLSSVTFRLAALVSVLPINSSEEELLEFLNSDRDSHMDSISRIGNRLIMHTQEILGFNVQDAFGGAIGMLTNESAELCTTPFVPSWNRLHYLHPMTEQAQFRAVCMFRTPHNAGIKAAVFLEPFMPSVWFAFAGLLIFAGVLLWMIFHLERHWMQRCLDFIPSLLSSCLISFGAACIQGSYLMPKSAGGRLAFIAVMLTSFLMYNYYTSIVVSTLLGSPVRSNIRTIQQLADSSLDVGFDTVPFTKTYLVSSPRPDIRSLYKQKVESKRDPNSVWLSPEEGVIRVRDQPGFVYTSEASFMYHFVEKHYLPREISDLNEIILRPESAVYGMVHLNSTYRQLLTQLQVRMLETGITSKQSRFFSKTKLHTFSNSFVIQVGMEYAAPLFISLLVAYFLALLILILEICWARYAKKKFSTIIPQNQ-

>DmelIR75d

MKVQVAHWLPLIFFLLVSGTPRVAGSWRSEYSRQDPDPKTRWGNQLPDMLVAYYRHHGVHSLMLVVCHTDIADFRLWKLWQHFNLNNFYVQVSTESSLRDLQHVDALDEHKDAPPPKSFHANNSTHWETSFLLPALPYKMGILLLEFSSECALNLLRWSAASEHNYFTTNRFWLLLTEDPGDIDLLEDPEIFIPPDSELRVLHYENVGNFSCSLIDLYKVAAWKPLKRTLVGHNIRNSRHVIHALQHFGSAITYRQDLEGIVFNSAIVIAFPDLFTNIEDLSLRHIDTISKVNHRLMLELANRLNMSYNTYQTVNYGWRQPNGSFDGLMGRFQRYELDLAQLAIFMRLDRIALVDFVAETYRVRAGIMFRQPPLSAVANIFAMPFENDVWVSILMLLIITTVVLVLELFFSPHNHDMSYMDTLNFVWGAMCQQGFYVEVRNRSARIIVFTTFVAALFLFTSFSANIVALLQSPSDAIQSLSDLGQSPLEIGVQDTQYNKIYFTESTDPVTKNLYHKKIASKGENIYMRPLLGMEKMRTGLFAYQVELQAGYQIVSDTFSEPEKCGLMELEPFQLPMLAIPTRKNFPYKELIRRQLRWQREVSLVNREERKWIPQKPKCEGGVGGFVSIGITECRYALGIFGCGAAVSFVLFLFEFIFRHFKQVYRIIKGYREVQR-

>DmelIR76a

MENLLVESYYFSTVLSFFAQQFFADSHATCIFWHPAFDFRLETVHPMPLIIMDWHRWANRSDQDVYDYKIKEDEFEGKGIPYNDWTLRLTVAIERSHCETFIAFQEQIPEFARYFYHASIYSIWRSLRNRFMFVYTKEFEDKKDSYLSGYIFQDQPNILVITSQYLNSSTFEIKTNRFVGPRNFNKNPEPVEFYILQRFDAKGTKATWETQSAMSSKMRNLKGREVVIGIFDYKPFMLLDYEKPPLYYDRFMNTTDVTIDGTDIQLMLIFCELYNCTIQVDTSEPYDWGDIYLNASGYGLVGMILDRRNDYGVGGMYLWYEAYEYMDMTHFLGRSGVTCLVPAPNRLISWTLLLRPFQFVLWMCVMLCLLLESLALGITRRWEHSSVAAGNSWISSLRFGCISTLKLFVNQSTNYVTSSYALRTVLVASYMIDIILTTVYSGGLAAILTLPTLEEAADSRQRLFDHKLIWTGTSQAWITTIDERSADPVLLGLMEHYRVYDANLISAFSHTEQMGFVVERLQFGHLGNTELIENDALKRLKLMVDDIYFAFTVAFVPRLWPHLNAYNDFILAWHSSGFDKFWEWKIAAEYMNAHRQNRIVASEKTNLDIGPVKLGIDNFIGLILLWCFGMICSLLTFLGELWRGQG-

>DmelIR76b

MATGIELLVAAALCVACPPLNDSPPTNLIQMGENGTLSPVTELPMDVDASEAGFDADAPVETLETINRKKPKLREMLDWIGGKHLRIATLEDFPLSYTEVLENGTRVGHGVSFQIIDFLKKKFNFTYEVVVPQDNIIGSPSDFDRSLIEMVNSSTVDLAAAFIPSLSDQRSFVYYSTTTLDEGEWIMVMQRPRESASGSGLLAPFEFWVWILILVSLLAVGPIIYALIILRNRLTGDGQQTPYSLGHCAWFVYGALMKQGSTLSPIADSTRLLFATWWIFITILTSFYTANLTAFLTLSKFTLPYNTVNDILTKNKHFVSMRGGGVEYAIRTTNESLSMLNRMIQNNYAVFSDETNDTYNLQNYVEKNGYVFVRDRPAINIMLYRDYLYRKTVSFSDEKVHCPFAMAKEPFLKKKRTFAYPIGSNLSQLFDPELLHLVESGIVKHLSKRNLPSAEICPQDLGGTERQLRNGDLMMTYYIMLAGFATALAVFSTELMFRYVNSRQEANKWARHGIGRTPNGQSVAPSRWLRGWRRLNSGHGQLLGASTHGQNVTPPPPYQSIFNGGSHGDPLNRWRRPLANGNALGNGVLLGGDSEGGVRRLINGRDYMVFRNPNGQSQLVPVRSPSAALFQYSYTE-

>DmelIR84a

MIKLQVKVISWPLIILTAFLRVLQIESINTNFLELAAFEDFLRSEHLSHVLVVRGDDADGDWKIECHQKLLANYRVQFYRPEMSANFEDLMFYGSPRTAVLVLNSEHVLVRRQVFGVASEAGYFNNSLAWFILGSGRESLPVEQLIDQLLSGYRMGIDADITVALRGPDNASMLFYDVYRISRQANTPLIIEKKGLWTHSGGYQKFGNFKNTWVIRRRNFLNVTLIGSTVLTEKPPGFGDMEYLADDKQLQQLDPMQRKTYQLFQLVERMFNLSLAISLTDKWGELLDNGSWSGVMGQVTSREADFAVCPIRFVLDRQPYVQYSAVLHTQNIHFLFRHPRRSHIKNIFFEPLSNQVWWCVLALVTGSTILLLFHVRLERMLSNMENRFSFVWFTMLETYLQQGPANEIFRLFSTRLLISLSCIFSFMLMQFYGAFIVGSLLSESARSIVNLQALYDSNLAIGMENISYNFPIFTNTSNQLVRDVYVKKICKSGEHNIMSLQQGAERIIQGRFAFHTAIDRMYRLLLELQMDEAEFCDLQEVMFNLPYDSGSVMPKGSPWREHLAHALLHFRATGLLQYNDKKWMVRRPDCSLFKTSQAEVDLEHFAPALFALALAMVASALVFLLELFLHWLPDFRRRLGTMST-

>DmelIR92a

MLLQPLVMHLSQLLRIIVGQYFAEFPSILIVYNNSASTTPLQLEYLSALELVLRELSKPIRLQWINVAFLKDLNDLEDQVMGALNSSVTEGFITILSQTHHFIHARYYATRNANVRLKDKRYLFLCEDESPAELLCMDILQFYPHHLMVRPGTETAPTGPTGPHPDPRRGGGASVSTKNKDDGEGGAGNKTTSPYRDINFELWTQKFVGAVGNLDALLLDAFLPNETFANRVELYPNKLLNLQRRSLLVGSITYVPYTITNYVPAGQGDVDPIHPQWPNRSLTFDGAEANVMKTFCQVHNCHLRVEAYGADNWGGIYDNESSDGMLGDIYEQRVEMAIGCIYNWYDGITETSHTIARSSVTILGPAPAPLPSWRTNIMPFNNRAWLVLISTLVICGTFLYFMKYVSYRLRYSGTQVKFHHSRKLEKSMLDIFALFIQQPSAPLSFDRFAPRFFLATILCATITLENIYSGQLKSMLTFPFYSAPVDTIEKWAQSGWKWSAPSIIWVHTVQSSDLETEQILARNFEVHDYSYLSNVSFMPNYGFGIERLSSGSLSVGDYVSTEALENRIVLHDDLYFDYTRAVSIRGWILMPELNKHIRTCQETGLYFHWELEFIDKYMDKKKQEVLMDLANGHKVKGAPQALDVRNIAGALFVLAFGVAFAGCALVAELLIHRMDLSK-

>DmelIR93a

MNPGEMRPSACLLLLAGLQLSILVPTEANDFSSFLSANASLAVVVDHEYMTVHGENILAHFEKILSDVIRENLRNGGINVKYFSWNAVRLKKDFLAAITVTDCENTWNFYKNTQETSILLIAITDSDCPRLPLNRALMTVECRINAVVFVDQTILEENALLVKSIVHESITNHITPISLILYEINDSLRGQQKRVALRQALSQFAPKKHEEMRQQFLVISAFHEDIIEIAETLNMFHVGNQWMIFVLDMVARDFDAGTVTINLDEGANIAFALNETDPNCQDSLNCTISEISLALVNAISKITVEEESIYGEISDEEWEAIRFTKQEKQAEILEYMKEFLKTNAKCSSCARWRVETAITWGKSQENRKFRSTPQRDAKNRNFEFINIGYWTPVLGFVCQELAFPHIEHHFRNITMDILTVHNPPWQILTKNSNGVIVEHKGIVMEIVKELSRALNFSYYLHEASAWKEEDSLSTSAGGNESDELVGSMTFRIPYRVVEMVQGNQFFIAAVAATVEDPDQKPFNYTQPISVQKYSFITRKPDEVSRIYLFTAPFTVETWFCLMGIILLTAPTLYAINRLAPLKEMRIVGLSTVKSCFWYIFGALLQQGGMYLPTADSGRLVVGFWWIVVIVLVTTYCGNLVAFLTFPKFQPGVDYLNQLEDHKDIVQYGLRNGTFFERYVQSTTREDFKHYLERAKIYGSAQEEDIEAVKRGERINIDWRINLQLIVQRHFEREKECHFALGRESFVDEQIAMIVPAQSAYLHLVNRHIKSMFRMGFIERWHQMNLPSAGKCNGKSAQRQVTNHKVNMDDMQGCFLVLLLGFTLALLIVCGEFWYRRFRASRKRRQFTN-

>DmelIR7a

MFHHLWLLMGLRSLAMGALHPPQPEAMTPLVAAALEILAEQVSPSQSTLAVMDLTQDAEHRDERQEQLMTIILRSVGSEMALRTFQKPPAEVPASFVVFLVNSAQAFNTLGFHFTDIHSTREFNFLILLTHRMSSRAERLQVLRDISRTCVRFHTSNVILLTEKRDGVVLVYAYRLLNMDCDLSVNLELIDIYKNGLFRHGHEARSFNRVLSLSGCPLQVSWYPLPPFVSFIGNSSDPEERAQIWRLTGIDGELIKLLASIFDFRILLEEPCNKCLSPDIKDDCSGCFDQVIISNSSILIGAMSGSHQHRSHFSFTSSYHQSSLVFIMHMSSQFGAVAQLAVPFTVIVWLALVVSSLLLVLVLWMRNRLVCGRSDLASHALQVLTTLMGNPLEARSLPRSSRLRILYAGWLLLVLVLRVVYQGKLFDSFRLPYHKPLPTEISELIRSNYTLINQEYLDYYPRELTVLTRNGSKDRFDYIQGLGKEGKFTTTSLIATMEYYNMMHWSTSRLTHIKEHIFLYQMVIYLRRHSLLKFAFDRKIKQLLSAGIIGYFVREFDACQYRKPFEEDYEVTPIPLDSFCGLYYISLIWLSAAVVAFILELLSQRIVWLRRIFE-

>DmelIR7b

MKYWLYILSCCSLVASTMESSSDWDLAEALAQVVANSEMGRFKTLYIYTHTNSQSTGGHLEELLDQVLMIVPNNLQARRLLLQQSMEYKPYVHAVLALVDGLPSLSAIYARIRATQDLSHTLIYMSMPTDAYGEEMQATLRFLWRLSVLNVGVVLRPPGDHILMVSYFPFSALHGCQVISANVVNRYQVGTKRWASQDYFPSKLGNFYGCLLTCATWEDMPYLVWRPDGSGSFVGIEGALLQFMAENLNFTVGLYWMNKEEVLATFDESGRIFDEIFGHHADFSLGGFHFKPSAGSEIPYSQSTYYFMSHIMLVTNLQSAYSAYEKLSFPFTPLLWRAIGLVLILACLLLMLLVRWRHHHELPRNPYYELLVLTMGGNLEDRWVPQRFPSRLVLLTWLFATLVLRSGYQSGMYQLLRQDTQRNPPQTISEVLAQHFTIQLAEVNEARILASLPELRPEQLVYLEGSELQSFPALAQQSGSSARVAILTPYEYFGYFRKVHPMSRRLHLVRERIYTQQLAFYVRRHSHLVGVLNKQIQHAHTHGFLEHWTRQYVSAVDEKDESVARIASTSYSTLDGIDGDPSLSESEEDQQVAPVRQNVLSMRELAALFWLILWANLGAVVVFVLELLLPRIKLRKILRKMKKSTRASATTTSTLSSPSTTKDIPFSCKDGFQDSWPKCSLLVS-

>DmelIR7c

MLHSAVHNVSLVYALVWAIDNYYGMATSTPLAVVQFPTSRESRRLHNDLIDAALGRSSGTGRIQFLLEDDRVEMTETDTDPPPPSGLTGRPIAIWFLDSLRSYFRLEMYLNQLGSPYKRNGFFLVIYTGLEDQPMESLKIMFRRLLNMYVLNVNVFLQRDGTVHLYTYYPYGPHHCQSSLPVYYTAFQDLAAPANGFGLTKPLFPRKLTNMHGCEMVVATFEHRPYVIIEDDPKTPGGRSIHGIEGLIFRSLAERMNFTIKLVEQKDKNRGEILPDGNFTGILKMMVDGEVNLTFVCFMYSKARSDLMLPSTSYTSFPIVLVVPSGGSISPMGRLTRPFRYIIWSCILVSLIFGFVLICLLKITALPGLRNLVLGRRNRLPFMGMWASLLGGLALYNPQRNFARYILVMWLLQTLILRAAYTGQLYLLLQDVEMRSPIKSLSEVLAKDYEFRILPALRTIFKDSMPTTNFHAVLSLEESLYRLRDEDDPGITVALLQPTVNQFDFRSGPNKRHLTVLPDPLMTAPLTFYMRPHSYFKRRIDRLIMAMMSSGIVARYRKMYMDRIKRVSKRRNLEPKPLSIWRLSGIFVCCAGLYLVALIVFILEILTTNHRRLRRAFNVINRYAA-

>DmelIR7d

MDIRCVVALLLGLCKVQAVVWPHQHLLEEQLASQISATLQKIFINGLAVYNFGVFISTSYEEMDRDRVILVHQVLNRNLYPPNFPVAVVLASKMNRKITAQVFTQLLFVQNAEQAIAIAEGVNRNGLCVIVLLTSQPERPIMTKIFTYFMQERYNINVVILVPRLHGVQAFNVRPYTPTSCSSLEPVEIDIKDGDLWDVFPRRLKNLHGCPLSVIVWDIPPYMRINWKSSDPMDGLDGLDGLLLRIVARKMNFTLKLIPNEPNGLIGGSSFMNGTFTGAYKMLRERRANITIGCAACTPERSTFLEATSPYSQMSYIIVLQARGGYSIYEVMLFPFEKYTWLLLSTILGLHWIVGSRWRMPSPILAGWMLWIFVIRASYEASVFNFIQNSPVKPSPRTLDQALSGGFRFITDHASYRMTLKIPSFQGKTLISAGQPVDVFDALLKAPWKTGAFTSRAFLADHLVRHRKHRNQLVILAEKIVDNMLCMYFPHGSYFAWEINKLLFNMRSFGIFQHHSQILAWDNLPTTTDTDTPGKRIHSSTESVATGFAESMSFVVAALNCLMGALCISIVVFGLELLSRRRHWTGLEWLFERV-

>DmelIR7e

MNHINEFVARAVLHVVHHYILSVTPSLVLTLCCRSNHTCNFYNKMMSTLFREWGLAPLQIVNVLRGVPWHPVPGRRHFNVIFTDSFAAFEEIRMEYYSREYNYNEHYFIFLQARDRLLQGEMRLIFDYCWRYRLIHCSIQVQKSNGDILFYSYYPFGEHGCSDMEPQLINRYNGSMLVEPDLFPRKLRNFFGCPLRCALWDVPPFLTLDEDQEEVLRVNGGYEGRLLLALAEKMNFTIAVRKVHVNMRDEALEMLRRDEVDLTLGGIRQTVARGMVATSSHNYHQTREVFGVLASSYELSSFDILFYPYRLQIWMGILGVVALSALIQLIVGRMLRERMGSRFWLNLELVFVGMPLLECPRSHTARLYCVMLMMYTLIIRTIYQGLLYHLIRTHQLNRWPQTIESLVQKNFTVVLTPIVQEVLDEIPSVQHMRFRLLEANSELDPLYFLEANHQLRQHVTASALDIFIHFNRLSADKVHQRGEQGSGAHFEIVPEDIISMQLTMYLAKHSFLIDQLNEEIMWMRSVGLLSVWSRWELSESYLRNEQSFQVLGTMELYAIFLMVLVGLIVGLLVFILELVSMRSIYLRKLFT-

>DmelIR7f

MQGEDANLYVARALRLVIENVLAQLSTTLVVTISTRHLGTAHWFEYMMNILMDSWRMVAVQLLRIRPDLVVNPVPGRKRVSLLMVDSYQGLLDTNITASNANFDDPDYYFIFLQARDHLIPKELQLILDHCLAHFWLHCNVMIQTAQVEVLVYTYYPYTADACQKAYPIPVNTFDGRKWKASQMFPDKLSQMHGCPLTVLTWHQPPFVELVWDPKHNRSRGSGFEIQLVEHLARRMNFSLELVNIALLRPNAYRLAEGSSEGPIEKLLQRNVNISMGYFRKTARRNQLLTTPMSYYSANLVAVLQLERYRIGSLALLVFPFELSVWMLLLLALLIHLGIHLPSARRGNEEDGGGGLQVVALLLGAALARLPRSWRHRFIAAHWLWASIPLRISYQSLLFHLIRLQLYNTPSFSLDQLLAEGFQGICTANTQRLLLEMPQLARDPDSIQSVDTPFDWDVLNVLTRNRNRKIFAVANQDVTLSFLHSSAHPNAFHVVKQPVNVEYAGMYMPKHSFLYEKMDDDIRRLDASGFIHAWRRASFASVHRKEQVHMTSRRYINHAKLSGIYMVMAGLYLLAGLLFAGEVLLRQRN-

>DmelIR7g

MNVTSLLNFESMKYIGAQTQAASINHHVAQALRVFIEDFYQRIAPAFIVVLSCRRPSPMNFYRNIMQLLYESVDTMIVQLVLVELGRPRRIAGPRTHNLLLVDSLDALLDIEIHTYTAQSDTSEYYFIFLQQRDALIPHDMQGVFAYCWRHQLINCNVMTQSSGGQVLLHTYFPYAPGQCNDSQPTRINMFLGESWKHRDYFPSKLHNLNGCPLIVLARKVSPFLDLDEGQRELRGLEGRLLQELSRRMNFSIQFSGLQDQLKNRTTWTEKQLLQKLVQERIAHLAIGYVRKRIQYATNLTPVFPHYSNRVVGCLLLNAHNLTSLEIWSFPFQALTWICLVAGDRLALVLAVYAASLGLPIDPPERPSLQLLFASWLIFGLIVRSMYSALLFFILRYHLHQRLPGNLQDLTHGDYAAVMGRTTLQDLREVPSLQDLLGLKSVIVTSEREEEVLRTLDRCTLREGAGSHPLFFGLISQDALLHLTQRGHRAGAYHIIPQDVLEQQLAIYLQKHSHLASHLDHLVMSIRSVGLVHHWAGQMASERYFRSRFLYREKRIRQPDLWAVYILTAGLYLLSLVVFICELLASRRAGL-

>DmelIR10a

MAVLGTVFLLFMLDLKTLNLTRLNGLLVEPTRDLPQLELWLRAGSDHQDAENPYVQWFLLRTEIPLSIVTYQENRYWMDDPFGRRNLVLVMSLDQLLTNRGAAAPIQKASTFFYILADQDKDLSADEQLRLEGSCRQLWTQHKVYNRFFLTRDGVWIYDPFKRRDSAFGRLVRYYGSETLDKLLFRDMAGYPLRIQMFRSVYTRPEFDKETGLLTRVTGVDFLVAQMLRERLNFTMLLQQPEKKYFGERSANGSYNGAIGSIIKDGLDICLTGFFVKDYLVQQYMDFTVAVYDDELCIYVPKASRIPQSILPIFAVGYDIWLGFVLTAFACALIWLTLRVINLKLRIVSLGNQHIVGQALGIMVDTWVVWVRLNLSHLPASYAERMFIGTLCLVSVIFGAIFESSLATVYIHPLYYKDINTMQELDESGLKVVYKYSSMADDLFFSETSPXWNRDLRADVIDEVARFRNKAGVSRYTSLILESSHFTLLRKIWVVPECPKYYTISYVMPRDSPWEDAVNALLLRFLNAGLIVKWIQDEKSWVDIKMRSNILEADAESELVRVLTIGDLQLAFYVVIGGNLLAFLGFLAEHFRWKLQKKGV-

>DmelIR11a

MRFAILWLFSGCLLPGIQVGIWVVVRAQPTGRDVLLSRLGNQQNELNTRRLANASSYLTRNYIANRINTLVVREICVECPYELSERQRQLVDQILASLAPELSVLLHKGTAEETTWEYTLFVVNDHTAFTGQVFIFPDELLEREFFCIVVVSEIQSRQFVRQTVGSIVKSNLQMHFVNVVVVAQLEDGTVGTYSYKLFKANCTPGITVRQINHFDRITGKPQQSMPDLYPVRNGHLGDCPFNVGAAHMPPHLIYKRHKDPPPASNVSIPAEDLAGIDWDLLQLLAKALKFRIQLYMPQEPSQIFGEGNVSGCFRQLADGTVSIAIGGLSGSDKRRSLFSKSTVYHQSNFVMVVRRDRYLGRLGPLILPFRGKLWGVIIVILLLAVLSTCWLRSRLGLSHPIEDLLTVIVGNPIPDHRLPGKGFLRYLLASWMLLTLVLRCAYQARLFDVLRLSRHRPLPKDLSGLIKDNYTMVANGYHDFYPLELTCRQPLDFSARFERVQRAAPDERLTTIALISNLAYWNHKHPNISRLTFVRQPIYMYHLVIYFPRRFFLRPAIDRKIKQLLSAGVMAHIERRYMQYENKRKVASNDPVLLRRITKSIMNGAYRIHGLVIVLATGMFILELLAGRSNGRLRRWMEWVHQ-

>DmelIR20a

MLASLNRSTGLSAELLDLYGLVVHFLLSGEHTTLVYFNPAGLDCSWGVLWQRNLTAHPQIVWQRNYSYPDLYYQFNAKLLVLACLPMDSRAAIQLEILANSLSHLRTVVRLLIEVAGPDQVTLARQYLSFCLRRSMLHVELYFRDYHHSLILYSFRAFPSFELVMRWISVGQGVKLFLHKLDDLRGHRLRVIPDLSPPNTFFYRDARGDNQVTGYLWDFLATFAGRLNAGLEVVRPSWRAGSASDSSYMLEYSAKGLIDVGLTTTLITKWNLWAIHQYTYPLLVSSWCTMLPVEKPLATPDLFGRIVCPTLAMTLLLIILVTWLVFRQLRCLTRLKNSRPARIVPHLLTLLLLTTCSAQLLSLLIFPPYHVRIASFEDLLRGDQKILGMRNEFYNFDGAFRARYAGVFYLIDDPNELYDLRNHFNTTWAYTMPYIKWLVIKTQQRHFSKPLFRWSKDLCFFDFMPTSVIVAPDSIYWESIKDFTFRIHQAGLMKHWIRKSFYDMIKAGKMSIKDYSDLETLKPLNIGDLEIVWRVCGAAIAVASAIFIMELLYFYINVFFNSL-

>DmelIR41a

MFIDLSWSLVLSAIVGKYLNESTICIFWNDKFEFQLLHKSDYISFVGINIKSFDDNGGHYIIDTGLKKKELQNKHLFLDELVIKIIISIEVTHCETFVVFDKDIDRFVNAFNKASVYSIWRSLHNKFVFAHIANESPESRNHFFEDQPNILFVVRDHSSASSFDIKTNKFVGRKAENPSQMILVDRYLASEQRFQFGKSLFADKLNNLQGREVIIAGFDYPPYTVIKHNMSTNAQDMGVSGESDFKNVYIDGTETRIVLNFCEQFNCTIQIDSSAANDWGKVYPNMSGDGALGMLINRKADICIGAMYSWYEDYTYLDLSMYLVRSGITCLVPAPLRLTSWYLPLEPFKETLWAAILLCLCAEATGLVLAYKSEQALYVLPGYREGWWTCTSFGVCTTFKLFISQSGNSKAYSLTVRVLLFACFLNDLIITSIYGGGLASILTIPSMDEAADTVTRLRFHRLQWAANSEAWVSAIRASDEALVKDILYNFHIYSDDELLRLAQDQHMRIGFTVERLPFGHFAIGNYLGPQAIDQLVIMKDDIYFQYTVAFVPRLWPLLDKLNTLIYSWHSSGFDKYWEYRVVADNLNLKIQQQVQETMTGTKDIGPVPLGMSNFAGFIIVWILGSAIATLTFLLELSLTYILKQSNLK-

>DmelIR47a

MRQIKLLVWLLVVGVVSSTEQLQFLKNFLEAVHKERSISTILLIQRKVHKNDFLHGLYPIFWPIICLDETKRVELVNNFNKDFLALVYMESEADTLLLSALAADLNHIRDARIMIWLQMSPSENFLDRIVFQASKQKFLNLVVIENTLKTRRFYPFPQPKVQVIDKPFEEKEIYPALWRNFMGKNAIAVPDLVPPRSFNSFDPKTGHRRESGSIYNVFKAFTQRYNITMLLKWPLIRNTTQEEIIGKSVRGEIDLPITGQLISFRHPNGSRSQPLLGMTALSIAVPCGPELPMFDRFFLFYGLATPITITGYYVLLNTIEIILGTLSDRIKRHPRRKKILNLVLNLRVFSCILSLPTPQGNRLRSVKGQLTMVMSITGLILSCIVAAQTSTILTMKPQYRHIKNFQELSDSNITVVCNHLNYLTIKQQMDPKFMAKFMQNIWIVNSIEQMKMIFDLNTSYAYQTFSYKKDPFTLLQMHTTRKAFCRTPGLDLVSGLAYTAVLEKNSIYALALQDYTLKAFSAGLVYYWAEESIRDLISTVGRTQFEKLPIVIGYQSLKLQDYNVCWKILLIGGALAFCVFIVEVVVGLINRRI-

>DmelIR47b

MREAQIIIFLLTSAAAVTLKQYEFLXSFLKAGEQEQTITTLLMMQKHVHTKNLLQGLYPXPWPIIHFVETQRIKFIALLYMSSEKDIFLSSLAANLKFERLDKPFGKSNIFPVLWRNYMGXIALTLDHLVEPRSFYWTDPRTNIKRRTGYIYMLITNFAEQHNITLQLXSPPNEDMSQMVIIERTHKGPRSTHNWADDQLETFERXQDSLLPWHGSMAIVVPCGQEMSAYERFHAAHAFRAPIIFFGFHIFLSLIDFLLRTISDRIRCNPRRIQLLQTVLSLCVLRCILSTSLPNSNXLRSRLRDNSPXXXVLQAXSYSALWXLTGTAXQXHNRDFQSHKLHDYXTTDGSXHSIEVPGLLKARNXXIXLFHIFSSLGTKFDLRIGSAGSHTSGVEFRYYELLDRXSSLENNIVSQVFTILKLPYSRFRVLKLEDCRGCWQTLFVGFSIATFVFIVNVLMGFFRNINQKK-

>DmelIR48a

MHLLITETYMIIGKTLHDILNELNERLIISTNIIFCKQFDNLIHFEAQTSRFVYSSLEAFNITSLWNHVGNDNKLFVIVGNVPPYELFAKLELSSPENCTQFILNNTVDMCADALVKNSKAFSVSRELRIAPANVIVPHGKPLLSYRYLAAPFNTKVWIALGTYVFLISGFLCLIHWLRSGKWDFSQNLLEVYSSLLFTVFHLKATNGIERYILFGVLFISGFVYSTSYLRLLKSMLIAETFEKQIQTFEELAESNIPLLINPYDRMIFQHHHIPKSLWTAVRTVSSETLLNHRSHGYVRLCPAILTASKIPSHTHRHLFSVCRFSHEQEVVPKGSSXXSLVPCIRKRNREXNHLGCLSGVSWPGISXFFHYGALGGEAFGSILLHDANYFPSPRLFRRLAELHYGSY-

>DmelIR48b

MILQQSSNLLKLLLLLAISSVRTQGLNDIIIELNQRLLISNNFLYCNQSDKLNEYEIKYLQHMPPISLMIFTSIESMNFTQVEYNLGADNKLFLIMGNEEPPYDFLHALNLHFQFAEYIIVIDEPVDLKKSTKWLDFVNHLWQQGYVQLLIYTSYDEKLYHKIIFPETVIEETLVEQYISIRGSFNNLYGYPVRVAAYNNAPRSMLYVNRWGKHIFAGFYMRFLRAFIDARNGSFVPVLTPSNSPGNCTLNLVNETVDVCADALAANPAAFSLTHGFRIASANVLVTHAKPLHSYRYLTAPFQWSVWACLVIYVLLVVNFLSFIGWLRSGKWEFSKYLLEVFSSLLFSGFYLKEIRGRERYILFGVLFIAGFVYSTEYLGLLKSMLISEVFEKQIDTFEALVESNITLMVDPYDKILFAKYNMPEILSPIMELVSFETLLKHRNRFDQDYAYILFSDRMALYDYAQQFLKHPKLLRIPIDFSFLYTGIPMRKRWFLKHHLGRAWYWAFESGLTRKLALDADFEAVRVGYLSFLITEHVEAQPLNVDYFVMPAIALAIGYILALLSFVIEMTAWRIREFLGCRKATMTSTGCSEGGHVDVD-

>DmelIR48c

MSLLRIILIIIFLRIVSSIPDTIISHLSAELQIKIQIYFGLGNDLYDFSRLDGNYQKIIISHNISEEFKTYHDEPVLIIIRLERDLNLNLATLDVLRSYLTDRQYNDILLIDNDEENLNSYVDIRKAYWNAGFSQVLIYNSQQRTWSIKPYPYLQIRPTSLKEYIENRNTRNLMGYPLRVLVTNDPPHCFVDKDELPGSPNRYKGSIVTMLKIFADQLNATFQANPFREFRRYSTADCVQMVSDDEIDACGSIFIRTYTYATSQPVRLNRVVIMAPFGNPIEKFYYFFRPFDLYVWIGTGIIVVYIAVMGSLLHRWHFKEWNVGQYLLLAVQTLLNRELSLPQSSSGSKFMLLLLLFAIGFILSNLYVALLSMMLTTKLYQRPIENLADLKAANVNILLQTHNIRPNSVYGSSEELRERFLLVEESQHLEKRNGLDPSYAYVDSEDRMDFYLYQQKFLRRRRMKKLSNPVGYTWAVQVIKQNWVLEKHYNDHVQRFFETGLQNKLVDDVHELAVKAGFLHFFPTQTQTIEPLRLEDIVMAAMVLGGGHALAVICFLVELFA-

>DmelIR51a

MYNVLVLFLLLFTRAQMEPHRRGHNMTLLRSVLTVIRGRENWKNTPIFLGGHCNSDDLNNLMSWLQNTMEVTCHTVDTSTSAKNENALGHFNINADNSLGLLFCQSSHELIWFNMDKRLRRLRGIRLIVILSDKRSSSSKAIMSTFKRLWHFQFQXNFQGYVVSTPVENDIPRVFFVKDKKTGRKQIRGFGYRTFVEYLHRYNASLHVSNSQQEHAINSSVNMGRIINQIVDGQLEISLHPYVDVPENMGDNSYPLLIASNCLIVPVRNEISRYMYLLLPLNQSSWILLLGSVIYISGVLYYIQPGLLHRTWDQRIGLNILDSISRIINICSPSRIYNPSLRYFIVSVHLSILGFVVTNLYSIMLGSFFTTLVVGEQVDSMQQLIQXQQKVLVKYYEVSTFLRHVEPDLVDGVAQLLVGVNASEQVSALLGFNRSYAYPFTLERWEFFSLQQQYAFKPIFRFSSACLGSPIIGYPMKSDCHLQSSLNMFIMRIQAAGLLRHWVVSDFNDAMRAGYVRLLENFLGFHSLDVDSLRLRWAVLLCGWLLSTLIFLCER-

>DmelIR51b

MCKVLTLLVVILLLALTNAAYNVTLLKSVLSLISTREPWINTPIFVGHNTQGGDLNDLIIWLHQTMGVTSLTMNLFLQPEHIRPLGHFKITRYNGIALFFCHDKHDIMWLTLDRNLRKLRRIRLIIILRNQRSGSQGAIKSIFNALWQYQFLNVLVLQRDQLYSYTPYPAMRFFKLDIHTEPLFPHAARNFHGYVVSTPAENDIPRVFHVHDPLTKSRKVLGYAYRTFVEYLDHYNASLRLTNPDENLDPTTSVNMNHIVQLIIDGQLEISLHPYVFTPPTATKSYPLLIYPNCLIVPMRNEIPRHMYLLRPFQLYSWYILLFAVFYITGILYCISPKLNKSSWPQRLGLNFLDAISKILFISPPITIYRPTWRHLIIFLQLSVLGFMSTSWYNIELDSFFTTIVVGEQVNSMDQLVHQQQRVLVKEYEINTFLRHVEPRLVEKVSRLLVPVNASEQVSALLSFNRSFAYPFTEERWQFFAMQQQYAFKPIFRFSSACLGSPHIGYPMRVDSHLETSLNHFILKIQDTGLLNHWVVSDFNDAMRAGYVRFVDNVLGYQSIDVDTLRLGWCVLGIGWILSALVFSCEYWHLYPWRFIA-

>DmelIR52a

MALGWSVIILGFIGQLSAQILNYTQSRDLELLEGSLFRVLSRLNLEEEYNTLLIYGKECVFHSLLRKLEISAVTVPSGSTDYDWSFSTAILILSCGYDAENEENSYTLMKLQRTRRLIYLEDNSEPESVCMRYSLKEQHNIAMVKSDFDQSDTFYSCRLFQTPNYVEGHFFKDQPIYIENFQNMRGATIRTVADSLVPRTILYRDEKSGETKMMGYLGHMINTYAQKLNAKLHFIDTSKLGAKKPSVLDIMNWVNEDIVDIGTALASSLQFKNMDSVWYPYLLTGYCLMVPVPAKMPYNLVYSMIVDPLVLSIIFVMLCLFSVLIIYTQHLSWKNLTLANILLNDKSLRGLLGQSFPFPPNPSKHLKLIIFVLCFASVMITTMYEAYLQSYFTQPPSEPYIRSFRDIGNSSLKMAISRLEVNVLTSLNNSHFREISEDHLLIFDDLSEYLVLRDSFNTSFIFPVSVDRWNGYEEQQKLFAEPAFYLATNLCFNQFMLFSPPLRRYLPHRHLFEDHMMRQHEFGLVTFWKSQSFIEMVRLGLASMEDLSRKRNEEVSLLLDDISWILKLYLGAMFISSFCFILEILRCGERCKRLWRCRW-

>DmelIR52b

MTWLVILLCFLGYMAAHIADISVQNQSLMDNELINLLLKLRNEEFYDTLLVYGKDCEFHSVIKNVDVAVVLVSDSMNFEWNFSSLTLILSCGPDIDNGGPNSTSIKLQRNRRLVLLKEDFQPSNICNIYTQKEQYNIALVRENFTKSKSIYTCRYFQDPNVDEVNLSGTKPIFIEQFQNMKGKAIRIVPDLLPPRVMLYQDANDGELKMIGYVANLITNFAQKVNATLQLDFLKPSTSITEISRMAKDDELDMGITLEASLNTSNLETSSYPYLLTSYCLMVQVPAKFPYNLVYALIVDPLVLGIIFVLFLLLSVLLIYSQKMSWQDLSVANILLNDKSLRGLLGQSFPFPLNASKKLRLIFTILCFASIMLTTMYEAYLQSFFTNPPSEPEICSFQDVGSYNRRIAMSALEVNGLIKTNNSHFREIRMDDLEIFDNMPECYELRDAFNLSYNYVVTGDRWRSYAEQQTLFKEPVFYFARDLCFSRLIFLSVPLRRHLPYRHLFDEHMMQQHEFGFVNYWMSHSFFDMVRLGLTSLKDLSRPLAYTPSLLMDDISWIMKIYLAAIVLCVFCFLLEIGVDKWKRWMKFRNLQILNTC-

>DmelIR52c

MVWLIIILFCLGNSSSQILDVTNNSHLDFDYRLFGLLQRLQVEKSYDTLLVYGEDCAIPSLFERLQVPAVLVSSGSTNFDWNFSSLTLILSCNFQDEREENYRTLMKLQTSRRLILLKGHIKPESVCDFYSKKEQHNVAMVKENFYQLEVVYSCRLFQDQNYEKLNLFDGKSIYKDQFRNMHGAPIRTLSDKEPPRTIPYIDSKTGEEKFKGYVGMLISQFVKKVNATMQIREDLIKDDEEVSFVDITNFTSNDILDIGICEARTLEMSNYDAISYPYLMSSYCFMAPLPDSLPFSDVYMAIVAPSILIMFLIIFCICSVLIIYIQERSYRSLTIRSVLMNDICLRGFLAQPFPFPRQYNRKLKLIFMLVCFSSLISTTMYTAYLQAFLWGPPIEPRLTSFDDVKKSRYTMAINIYEREFLEALNVSLEDVEIYDYGKFSKLRSTFNTNYLFPVTALQWFTINEEQKLFKYKIFYYCDAFCLNQFDILSIPLRRHLPYRDIFEEHMLLQKEFGLTKYWIDQSYRDMIRANLTTFKDFSPLLENDYIEVHNLYWVFTMYFVGMGMGLCFFILEILRPLRYWRNCKIKCEYCYAFLKNFAK-

>DmelIR52d

MVRIIIILLCLGYTKARILDATNTNHTDLEERLLSLLLRLQQEQFFNTLLIYGEDCAFSSLSRRLQVPTILVSSGSTSFEWNYSSLALILTCEFKAEREENYQTLKKLQMNRRLILLNGNIKPDSVCDFYSKKDQYNIAMVNNNFHQVGIIYACRLFQERNYEKVYLSEGNPIYVDQFRNMQGALLKSITFNLIPGSMAYRDPKTGQEKHIGYVANLLNNFVEKVNATLDMQVKLHKAGKKTSFYNITKWASEDLVDIGMSYAAYFEMTNFDTISYPYLMTSTCFMVPLPDMMPNSEIYMGIVDPPVLVVLIAIFCIFSVMLNYIKQRSWRSLSLVNVLLNDICLRGFLAQPFPFPRQSNRKLKLISMLVCFFSVITTTMYTSYLQSFMWGPPIDPKMCSFADLENSRYKLAIRRYDIEMLRPFNVSMDHVVVFDESSQLEYLRDSFDDNYMYPMSALSWSAFKEQQKLFAFPLFYYSEKLCLKPISFFSFPIRRHLPYRDLFEEHMLQQNEFGLSTYWIDRSFSDMVRLKLATMNDFSPPRLEDYIEVSDLSWVFGMYFTGLGISCCCFGLELLGLPSWTRRLRLTNWLRVRN-

>DmelIR54a

MWTVITGIVLWAPVLVAGSAVDFIFRAAAEHSLSVIMIRIDYCPYNWAKDIFENQTIPVVVLSDSETFINIRMFSRPLHVACLPGHELQKDLALLENFTSSLMDFPSQKKIVYISNNFSDPTRMDYIFETCYHRRIWNIVGLLASDEHRYFYRYHLYPSFRTEYRSLESSTIFDKDFPNMHGHPLTVMPDQWLPRSVLYVDRRTGKQILAGSVGRFFHVLSWKLNATLQLSKKVTTGRFLNATALKELSESFSVDVPASLTIMERVEQLASTSYPMEVTHVCLMVPVARRIPIKDIYFILSSASNMFLAIVIVSSYGLALNLLRNMTHRDVRLVDFVLNDKALRGILGQSFNLPLSRSFSTRLIFLMLGIVGLNVSSIFGAGLDTLMAHPPRQFQARSFAGLRRTKIPLVTTEEDFPTWMKLRVPMLVVNVSEYNHLRNGRNTSNAYFASRLYWNLFSEQQKRFTRELFIYSTDDCLWSLALLSFQWPQNSLFTEPVSQLILEVNANGLYDFWVGMHYYDMTAAGLSGLEDPSLQLKEREHPTSLRIVDFQWMWQAYGTFMVIAILVFLLEVSWHRITSLFVSLVY-

>DmelIR56a

MGSRFFIRNLILFGLLASSNMQIPFGELEKKFELDVDFLLGVTELVGHIQGLYSITVYADCIDIHPSIQQRIMDKFMVPVNTIGSNLSRPNYHKLDNSRIRIVLFTGLNDTILVNLNKTDVPYSDNFYMLAYASAIKNKCIELDFIEEVFTLLWKMSIQNAILLIRGEFMMEMWSYLYMGKIHKIKLTKPNSYLESLRKYNYRFSLEVINDPPAIFWYNSSEQADVTGGGNLSVSGPLGLIIINFLRHLNVTIDIVPIPGKQTSQYELFQQPDNLRAENGVNMVGSALLKYSPMVTQSRMCLLVSNRRMIPFSRFLDRLVSPGVHKLTFVSSIGIFVIKYFSHRPRSFVDAIFCTIRFFFAIPLPSIILNRLPVVDRFIEVFIIIFVQILLSSNISITTSALTTGFWEPPIINVETMRASGLHILTEDPTILQAFKENILPSSLADLVILVDEDTYFHHVTTLNNSYVYVVQAHNWQIFRLYQQQMTNEPFEIASEELCSKWRILGIPLNPKSPLRFMFKDYFYRILESGLREQWVHSGFKKFCEFNNLKKLPVDSVDSWQPLSIEFYSNVIRAYIIGLVIATLAFVAELLHNGYRRKNVKKT-

>DmelIR56b

MLLDTDLASGVIRSPYSFDIPHAFIFNETQFVVPKFCGPYMEIVKHFAEVYHYQLFLDSLESLPKKSVVEQDIISGKYNLSLHGVIIRPEETSDFFNATQHSYPLELMTNCVMVPLAPELPKWMYMVWPLGKYIWTCLFLGTFYVALLLRYVHWREPGNATRSYTRNVLHAMALLMFSANMNMSVKLKHASIRVIIFYTLLYIFGFILTNYHLSHMTAFDMKPVFLRPIDTWSDLIHSRLRIVIHDSLLEELRWLPVEYQALLASPSRSYAYVVTQDAWLFFNRQQKVLIQPYFHLSKVCFGGLFNALPMASNASFADSLNKFILNVWQAGLWNYWEELAFRYAEQAGYAKVFLDTYPVEPLNLEFFTTAWIVLSAGIPISSLAFCLELFIHRRKQRRPQYERFECYDY-

>DmelIR56c

MRSSFRICLFLLTTYHPSHGWNMQHLLNLLAPFGRMNVFQEIVWFVSPHQRLDQLDEFIMRIDEAFGKSATQTVVNNNTEMRMIYSSARRNHMSFVFTTGAEDPIMKVFSKVLLGRHFYVSMVIYVDKVGDMHPIYDLLTFAYNQQFFNSMVHFESMEGVNQLFGVSKFPVMSFENRTDFLKYMGKIWKQVQNARSDVGGFGFTTPLRQDLPHLFQSQGHYDGSTYRIIETFVRFINGSFKELIMPPDSLGGQVINMKDALQLIRERKMEFCAHAYALFMSDEELEKSYPLLVVQWCLMVPLYNSVSTYFYPLQPFDWNVWFFALGALLALVLLELMWLRMFGGWSGYRGAVLNSFCYIINVPIEGQLQQPCLLRFLLLATVFFHGFFLSAYYTSNLGSILTVNLFHAQINTMNDIVSAQLPVMIIDYEMEFLLNLNKELPQEFLELLRPVDSAVFSEHQTSFNSSFAYFVTEDHWEFLDEQQKHLKQRLFKLSSICFGSYHLAFPLQMDSSLWRDIEYFTFRIHSSGLLNFYARSSFGSALHAGLVQRMPDTQEYTSAGLQHLAIAFILLLVMSFLAGIVFVLETLSR-

>DmelIR56d

MDNRAAELILRERNIFPTNGSDNITLLNNMFVLEMFYRITQLYHFKNFIFYISERLDLNNKDSQEFFHNFWTYFPMAPNLIITREHHLGIPMMQFISTPSLVMVFTTGKDDPIMELASHNQQGIHWLKTIFVLFPSLQSRDFETNPESLAQFTAEIKDVYDWVWRKQFINTFLITIKDNVFILDPYPTPSIVNKTGVWQAEEFFHKYAKNMKGYLVRTPILYDMPRVFKSDRPTNRYEKNFIHGTSGNLFLGFLEFVNATLMDTSANVTADYLNMTNLLDLVSQGVYETLIHSFTEITTKFVVSYSYPIGINDCCIMVPYRNQSPADQYMHEALQENVWVLISLFTLYITVAIYLCSPLRPRDLSAAFLQSICTLTYSVPTFIIRTPTLRMRYLYILLAIWGIVTSNLYISRMTSYFTTAPPVRQINTVQDVVEANLRIKMLAIEYERMAKSPLQYPESYLNQVDLVDKHMLDLHRDPFNTSFGYTVSSDRWRFLNLQQLHLRKPIFRLTEICEGPFYHVFPLHKDSHMRSVMTEYIMIAQQAGLMNHWERETFWEAVHLHRIHVHLFDDEPMALSLDFFSSLLRTWTLGLILAGLAFAAEMKWHEHVTFKRRPVIRITRKPRSFLRRFMKL-

>DmelIR56e

ERXAFRNQWAFCFPRTXAIEVVLSAWSPXCPGQRSKPQPISXPHHXGSCWRKRKWKXKPRLLVVDKRTLVEHLNSLNDGYAYCIIAGHWQVGMM-

>DmelIR60a

MWCNNPGLIIIIFLGQILNLCQGIVNLSNETANTVIFMLPEKDLGPDVWKAGVGCLDSFAQIFFFRNPKERFTRAYNLMLVHAFHLSSPADQIQEGFSKLINEAVTNPGPPDREELFQMRVASDYNITNGTEDKGELILADNYVIVVDSVDRLKELMKKKIVEMRSWNPGARFLVLFHNATCRNRPLGVASNIFKDLMEMFYVHRVALLYANSTMNYNLLVNDYYSNVNCRILNVQSVGQCHDGKLYPNNAVVKASMQDYVSGFSPRNCTFFACSSISAPFVEADCILGLEMRILGFMKNRLKFDVNQTCSLESRGEMDGPANWTGLLGKVQNNECDFVFGGYYPDNEVADHFWGSDTYLQDAHTWYIKMADRRPAWQALVGIFEAYTWIGFILILIISWLFWFTLVMILPEPKYYQQLSLTAINALAVTISIAVQERPICETTRLFFMALTLYGLNVVATYTSKMIATFQDPGYLHQLDELTEVVAAGIPFGGHEESRDWFENDDDMWIFNGYNISPEFIPQSKNLEAVKWGQRCILSNRMYTMQSPLADVIYAFPNNVFSSPVQMIMKAGFPFLFEMNSIIRLMRDVGIFQKIDADFRYNNTYLNRINKMRPQFPETAIVLTTEHLKGPFFILVVGSCWAALTFIGELIIHRWRTQLVSTSEQQDRRSDKRRRRRRRRKPEKDNRWQRQVQVAPVVRFTPVKRRKVFQGQTSQK-

>DmelIR60b

MRRSLYLIIAIGLVDVHCVSLRYILNALENELQYRAILLVESASEIESCWEQKYIQGAVPILNFNANQSLYLKDALNTNILALVCLNENVESTMQALYENLEDMRDTPTILFVLSDSKVQDVFLECLRRKMLNVLAFKGLDRGFVYSFRAFPTFRVIERNVMDILQYFEQQLEDLGGHTLTTLPDNIIPRTVVYKSPDGSRQLAGYLYPFLRNYVSTINATLKVCWHLVPEDGMIQLGEVVRLSEIHDVDFPLGMHGIEHGSTSQNVPLEVSSWFLMLPMEPSLSRAQFFIMLGFEKVTPVLLLLTILLSTAHRIEMGLRPSWRCYVLGDRVLQGTLGQAFFLPRRLSVKLMLVYSLILLNGFTFSNYSITSLETWLVHPPSGHPIHSWEQMRTLNLKVLIVPSELDSMTKALGKQFTESNSDLFELSKSGNFQDKRLAMDQSYAYPVTCTLWPLLEHAQIRLPKPEFRRSREMVLIPLLIMAMPLPKNSMFHKSLNRYRALTHQSGLYEFWFKRSFNELVALRKIHYKVNGDHQIYRDFEWQDFSYVWLGFVGGTIASILVLLAEIGYHRWQLNQN-

>DmelIR60c

MEMRLALFFTFACLAGAHDGSLRNMLKSLEDELGYRTILLLEGFVYSFKAFPTLRVVKRRVKDVRRYFEPQLEDLGGCVLKVVPDGIMPRTMVYQGEDGELQMGGYLSHFIRNYVSTINASLQIRWDLFPEDGDFDMDSLTGSNHVDFPLGLGSLSFQTLHKDVAMEISSWFLMLPMEPSLPRARFFIRFGISLYLIPLIILLAIVLSNAHRFEAGLTPSWRCCSMGNTVLRGVLAQAFVLPKGLSPKLMFVYWLLLVSGFFVSNYVIVYLTAWLIQPPTSDPVTDFDQMRRAKLKILMVPTDMDYLKSIRGAEYVDAHSDVFQTADSTDFQTQRMSMELHFAFSVTGTLWPLLRQAQVKLHRPIFRRSKEMVFLPFVIMGMTMPNNSIFLSSLKQYRLRTSEAGLYLLWFKKSFSELVAIHKISYKEDWVHDSYSDLKWEDFLFAWLGFLGGTTVSCLALLAEIGYHRWLWKRTHQ-

>DmelIR60d

MRLAIYVAFLSSIGNRSGFLSSLLMSLGKELHYKTILLVGGSSTCWSLEPFETGVPILNLRGENNAYPQDTFNSQMLALACLQTESEDAVKLLYRSLKDMRDTPTLLFASSEEHIHDTLFLGCFRENMLNVLALTASSKEFIYSYQAFPTFRVIKRKLVEIHRYFEPQLKDLGGHIVSALPGNIMPRTMCYRNAEGERQLAGYLNTFIRNYVESINGTLRISWGLVPEDDMRHLTISRLSKIQHVDFPLGIIPLYNKTDKQHVYMEISSWFLMLPMETSVPRAHLFVKLGLERLLPIIVVVGAVLGNAHRIEVGLGPSWRCYYLADKVLRGALAQPIVLPRRLSPKLMLIYSLLLLSGFFLSNYYMASLTTWLVHPPASDRILEWDQLRYLHLKVLTIPEEFKYMSLILGTDFMTAYGSIFQLTNSTDFQRRRISMDPSYAYPVTTSLWPFLELSQVRLRRPLFRRSYDMVLQPFQVMSLPLPRNSIFHKSLLRYAALTRETGLYYYWFRRSYYELVALGKISYKEEEGNPYCDLKWNDFRIVWLAFLGGTIISCLALLLEVAHYRWHLGNSSL-

>DmelIR60e

MVIKMISFLLVSVLLCLVGASDSESMQVQVLQDLNLALQTELNVFIDFECCATSEILHKLDSPRILLSSNSREARDLRIRGNFTESTLIIVSVMDSDLNPLVASLLPRLLDELHELHIVFLSNEEPGFPKQDLYTYCFKEGFVNVILMSGKGLYSYLPYPSIQPISLSNVSEYFDRARIIRNFQGFPVRILRSTLAPRDFEYSNEQGGLVRAGYLFTAVKELTYRYNATIESVPIPDLPEYDVYLAVAEMLHTKKIDIVCYFKDFSLEVAYTAPLSIIREYFMAPHARPISSYLYYSKPFGWTLWAVVISTVLYGTVMLHLAARGARVEIGKCLLYSLSHILYNCHQKIRVAGWRDVAIHGILTIGGFILTNVYLATLSSILTSGLYDEEYNTLEDLARAPYPSLHDEYYRSQMKAKTFLPERLRRNSLSLNATLLKAYRDGLNQSYIYILYEDRLELILMQQYLLKTPRFNMIRQAVGFTLESYCVSNSLPYLAMTSEFMRRLQEHGISIKMKADTFRELIHQGIYTLMRDDEPPAKAFDLDYYFFAFVLXTVGLISSLLVFFAELVSGHL-

>DmelIR60f

MRFHLNIANSGLLGLHLCPTRSALPEQNPCFSKAGAVIXNLTLPWRRWRERCLLGALRPXTLPTPELQCXSKYLPXRKSQQENASSGLPGFCXGDXQTELHRGSRAIALPRSPYHDLYYVWIAYLGGTMIGIGMLAVEIACFKWDLLRRPPIXMY-

>DmelIR62a

MYLQFLFALFLSRYQIVATENFDRAFELALFLDRIGRVHRLHAITIVNSLGSVDPSYLDDLHRGLMCNSSNHFYMLPQMTATDKDSSHVHFSSLQDEETIYLVFARDSKDAVIYLQAERARGRRYTRTMFLLRKQESQKDIKYFFELLWKLQFRSALVVVAARNFYQMDPYPTVRVIRMRRLSSYDPHHVFPPANRKNFRGYRMRLPVQQDVPNTFWYKNRRTKAWELAGLGGILINQLMMHLNVTMDLFRFEVNGSSLLNMAALTDLIVKGKVELSPHLYDTLQSNTSVDYSYPTQVAPRCFMIPLDNEISRSLYVFLPFSLTMWLCLLFVLLVVHFVYVRRLIPDGHFWAILGVPGAGQVRYGNRKPVRRFSTFLILFGIFILGQTYSTKLTSSLTVTLIRRPDNSLEELFLLPYRILVLPTDVYAIVDSLGHAEQFSTKFSCTDAENFSQKRISMHPEYIYPISTIRWRFFDMQQRFLRKKRFYFSKICHGSFPYQYQLRVDSHLKDALHRFLLHVQQAGLHDLWLDTCYRKAHRMGYLKDFSTLAELEEKLRLRPLALNLLVPAFSLFLCGMLGSGIAFLVEIRHSFGCRQKPPSINRNPGD-

>DmelIR67a

MLPILVPVLLLFNETSWINPILTSIYKDRHHETVLLLQHSQHGNASGLERFPWPVFSFNEQMDFYVRGKYNSEMLVLIWQTGNSDWDLDLWQALDRSLLNMRKVRVLLLRKWEKIPTADVAATAEHLLFLHVAVIGQGNRIYRLQPYAPQSWLQVDPIESPIFIKIRNYFGRYIVTLPDQFPPRSIVYRNPKTDEIQMTGYVYKFLLEFIRIYNFTFRWQRPIVQGERMNLILLRNMTLNGTINLAISLCGFETPSXLGVFSDVYDMEEWYIMVPRAQEISIADVYVVMVSGNFLIVLIIFYFIFTILDTCFGPLLLKERVDWSNLMLNERMISGIMGQSFNMSARNTISSKVTNATLFLLGLVLSTLYAAHLKTLLTKRPTSQQISNFKQLRDSPVTVFFEEAERFYLKHAWDRPIRYIKDQLNFRETIEYNALRMGLNRSNAFSALTSEWMIVAKRQELFKQPIFTVQPELRVIQTSVLLSLVMQSNSIYEDHINDLIHRVQSAGIVEYWKHQTLREMITMGMISQKDPFPYVAFREFKVGDLFWIWLLWVSFLFMSFVIFLCELLVDCFISKTLIRNKRPH-

>DmelIR67b

MELLYLNTLQSLSLLEGNRLVQTVQELNNIYQTELNVFLEFGNGADILESAQGTFVPTLWIKNPQNQKVMKGNFTSCTLTILYLEDEHLDRGLYYLANWLWEYHHLEVLIFFNGGSYDKLIQIFSRCFNEGFVNVLVMLPGSDELYTFMPYQDLKILNLKSIKEFYSLSRKKMDLNGYNITSGLVIAGAPRWFSFRDRQNRLILTGYMLRMIVDFTNHFNGSVRLMNVLTVNDGLELLANRTIDFFPFLIRPLKSFSMSNILYLENCGLIVPTSRPLPNWVYLLRPYAFDTWIAWLIMLIYCSLALRILSKGQISISAAFLKVLRLVMYLSGSRDMGTRPTTRRLFLFVILTTSGFILTNLYVAQLSSNSAAGLYEKQINTWEDLDKSDSIWPLIDVDIKTMEKLIPDRTKLLKKIVPTLEADVDTYRRNLNTSCIHSGFFDRIDFALYQQKFLRFPIFRKFPHLLYQQPLQISAAFGRPYLQLFNWFVRKIFESGIYLKMKDDAYRHGIQSGLLNLAFRDRHLEVKSNDVEYYYLIAGLWFGGLTLATVCFLLELLIGYAKIKVTISCKMNIM-

>DmelIR67c

MFCWLIFLNIILLSDRSESWSAREVIHQFNHDQQLQLNIYLDCNDVELQIGQEVSNLFVNSTADKMKILGRFSSHSLIIACFKDSTRNRTLNGVKELLWGLQYLPILFVVDSNMDFYFQQALRHGFIHVLALNFMNGSLYTYKPYPKVEVHQIKDMQKFYKLTKLRNLQGQAVRTTVETMTPRCFRYRNRHGQLVYAGYMYRMVKEFISTYNGTEEHVFGNVDTVPYKEGLAALKNGEIDMMPRIIHALEWYYFYRSHILYNIKTYIMVPWAEPLPKSLYFIQPFRGTVWITIMVSFVYASIVIWWIRYRQQGNSSLTQSFMDVLQLLFQLPLSKIWHFNMGTHQVVSFIVLFVFGFMLTNLYTAQLSSYLTTGLFKSQINTFDDLFREKRTLLVESFDAEVLHNMTKEKIIQKEFESIILITSIEEVFKHRKSLNTSYAYEAYEDRIAFELSQQRYLRVPIFKILKEVYDQRPVFVALRHGLPYVELFNNYLRRIFESGIWIKLQEDSFLEGIASGEISFRKSKSREIKIFDKDFYFFAYILLGMGWCVSTIALFLELWSFKYSVTNVLHEG-

>DmelIR68a

MRCLWILIVAFISLAMATSIPIPIANPAPLSGYEMQLKILLQKILWVANVKRCFAVITDDLHYPIYDRIFFESVGRRVIPFFVMRTNESDDLQRPSRQVELFVKAIKSSDCELNVITILNGWQVQRFLGYIYDNRSLNMQKKFVLLHDLRLFESDMIHLWSVFIDAIFLKRQLDNKYTISTIAFPGILSGVLVMKNIANWELGKGLNGRILFADKTSNLFGTSLPVAISEHVPMVLWANATKSFQGVEVEIMNALGKALNFKPVYYKPNQTENMDWTELDGGASVAYGSGNPDGYAQNGTHIDSMLVDEVAAHSARFAIGDLHLFQVYLKLVELSAPHNFECLTFLTPESSTDNSWQTFILPFSAGMWVGVLLSLFVVGTVFYAISFLNAIINGNVSSEFFRCLRPNRNVPMDPKIYRRISFRIAISRYRSSKGDRMPRDLFDGYTNCILLTYSMLLYVALPRMPRNWPLRVLTGWYWIYCILLVATYRASFTAILANPAARVTIDTLEDLLRSHIPPSTGATENRQFFLEANDEVARKVGEKMEVFGYSDDLTSRIAKGQCAYYDNEFYLRYLRVADESGSALHIMKECVLYMPVVLAMEKNSALKPRVDASIQHLAEGGLIAKWLKDAIEHLPAEALAQQEALMNIQKFWSSFVALLIGYVISMLTLLAERWHFKHIVMKHPMYDVYNPSLYYNFKRIYPQH-

>DmelIR68b

MKFLVGLLLQWYLPGIYALAEIACRIAVEQNVQVTYLYRCASCPASFDADYSALELDLYRCVGSRLPVITRNMEAHELEPFRRTDSLSIFQIPAAEKGDSLVRRILDMLNPHQRRKHMHKYLFVWPNAGRHQLLRLFRGSWAKKLLYGLAITGRENGTFDFDPFAWGGLQVIQRLDGEVPYARKVKDLRGYPLRFSMFTDPLMAMPRSPVETAGYQAVDGVAARVVGEMLNASVTYVFPEDNESYGRCLPNGNYTGVVSDIVGGHTHFAPNSRFVLDCIWPAVEVLYPYTRRNLHLVVPASAIQPEYLIFVRVFRRTVWYLLLVTLLVVVLVFWVMQRLQRRIPRRGVIQFQATWYEILEMFGKTHVGEPAGRLSSFSSMRTFLMGWILFSYVLSTIYFAKLESGFVRPSYEEQVDRVDDLVHLDVHIYAVTTMYDAVRSALTEHQYGLLENRSRQLPLGIATSYYQPVVRRRDRRAAFIMRDFHARDFLAITYDSQAERPAYHIAREYLRSMICTYILPRGSPFLHRLESLYSGFLEHGFFEHWRQMDLITRVGASPDAEEFLEDLGDQTDTDSGSNELAIRNKKVVLTLDILQGAFYLWSVGIGISCLGFAVEHAHWFWRRQTLRNAVEARTS-

>DmelIR85a

MSIQWLKHILLLAILVNLAGTRENHIPLDLKKSSIVMVKMSQILCKARIKVLFVYFENQTSHEHTGQILKEVTKCDISNQNTPLEAVKDDGILMYMVMITTNISQPLELSLIRKKSAAKHRSHVFLLVRDADTVSDAWMRASFRQFWKIWLLNIVILYWRDGRLNAYRYNPFMDNYLIPVDNKPNEVPTLEQLFPKTIPNMQRKPLRMCIYKDDVRAIFWRQGTILGTDGLLAAYVAERLNATMMITRPHSYNNHNLSSDICFLEVAKEYVDVAMNIRFLVPDTFRKQAESTVSHTRDDLCVIVPKAKTAPTFWNIFRSFGSLVWALILVSVLVANVFCYILKSEVGRVPMQLFAGALTMPMTQIPPNHSIRLFLIFWLYFGLLICSAFKGNLTSMMVFQPYLPDINQLGALARSHYHIIIRPRHVKHIQHFLTLGHKHESRIREQMLEVSDTQMYEMMRNNDIRFAYLEKYHIARFQVNSRVHMHLGRPLFHLMNSCLVPFHAVYIVPYGSPYLGFLDSLIRSSHEFGFERYWDRIMNSAFIKSGVKVVNRRRGSGNDEPVVLKLQHFHAVFALWLVGIGMACIVLAWEHLTHNYNLAVTKRRD

>DmelIR87a

MSTPEQRFWLAALLFLLSQHSEVRGFGINLMKVQTEDKGQEACILALLRKYFDSGDGLSGSVLCINRNYQLPNIEEQLLRGVNNYENYPWSLLITNSREGPSPAKFLMNEKPQCYFLIVDNLEDEDLDEVFEHWKGMVNWNPLAQFVVYLASLEETDEEMNDLMVELLLTFINKKIFNVNVIGQSEENQFYYGKTVFPYHPDNNCGNRVISVELLDACDYPSEETDSEDENDEDEGDGAQEEDDGPQEEGDGEQEEEDGPQEQEDGDQAKGDEGQENDDGGLENKVENEFRIGASDDDELENDLSSNSSEPEAIIEEFFRAKFEDKFPRDLSGCPLTASFRPWEPYIFRNSEEQPVDDYYYGLQGDEDDYNDTSPNYGESDDESYADPGEDGDGAIPDTETQSGGKLKLSGIEYEMVQTIAERLHVSIEMQGENSNLYHLFQQLIDGEIEMIVGGIDEDPSISQFVSSSIPYHQDELTWCVARAKRRHGFFNFVATFNADAGFLIGIFVVTCSLVVWLAQRVSGFQLRNLNGYFPTCLRVLGILLNQAIPAQDFPITLRQLFALSFLMGFFFSNTYQSFLISTLTTPRSSYQIHTLQEIYSNKMTVMGTSEHVRHLNKDGEIFKYIREKFQMCYNLVDCLNDAAQNEHIAVAVSRQHSFYNPRIQRDRLYCFDRRESLYVYLVTMLLPKKYHLLHQINPVIQHIIESGHMQKWARDLDMRRMIHEEITRVREDPFKALTFDQFRGAIAFSGGLLLVASCVFAFELCYVKYVYRTEKRERKTKKITKKVHNIKIQHD-

>DmelIR94a

MALPKQLKFINIFLVLLIIYGSSDGTENQHEIFLNRLLQAVHNERSVETLFLLHHSNLANCSLQDWNPPRIPTIRSNELTVFNVEKTFNHNALALVCLMKNSYREILNTLAKSFDCMRQERIILMIHRKSDSKFIEDITHEVKNLQFLHLIVLIVQEKYNGQVFASTLRLQSFPEPHFKRIRNVFAIQRIFYRPINFHGKVLNAIPNDIPILFVALNEMFTEYARRYNSTLRIQNRTIKEDIEITEDNYDIDMKIQLHNSQNFLHHMNIAMDIGSNSLIILVPCATELRGLDIFKELGVRTLTWLALLFYIIFVLVEMLFVFISNRFNGRNFTMRYTNPLINLRAVRAILGQTSPISNRYSLSIQHFFVFMSLFGTLFGGFFDCKLRSFLTKRPYYSQIENFSELRKSGVTVVVDHTTRQFIEQEINANFFRDEVPNVRTTTIQELINHVYSYDRKFAFVANSIPWRTFREEMKSINQKILCDSKNLTILENVPLTFSIRRNAIFSHHLRNFIINAADSGMITCWFKMAGKVIRKHIKTTLRESEQQPSHLPLSFDHFKWLWAVLCIAYVMSFMVFVMEILWSKYQRRTRSVSIV-

>DmelIR94b

MSLIFNLLFILILSQAVSQETEFLQLKYLNNIVRSMIKLHKMETLVIVKHHLDNNCSLQNWNAHGMGIIRTNDQGKLIMKDTFNSRTLAIICIGQNSHITLLRNVFETFGKVQQKKIILWTQMELKEKFFQEISKKSRDLKLLNLLVLKAVTKDKLLIYRLNPFPSPHFKRIENIWTPNDTLFMDTKFNFHGMTAVVKHDYNWTIQMGNIRKFPISRIEDKEVIEFALKYNLTLQFFNDVERFDIELRKRIILKSNSTQPIDSGIPMVFSSLLIVVPCGNYLSIQDVIKVSGIEKWIFYIILVYVIFVLIEITFLGVTILISRQSRHQMIPNTLVNLCAFRAILGLPFPETRRTSLSLRQLFLAIALFGMIFSIFINCKLSSMLTNPCPRPQVNNFEELKTSGLTVVMDHDAENFIEKEIGVDFFNQYMPRKVTLTFTERAKLLFSLKGNHAFTLFSESFAIIESYQRSKGLRAHCTSEDLIVAERVPRIYILENNSILDRPLRRFIRQMQESGITNHWLKNIPSSLEKNLMQITIPYDRERVHPLSIEHLTWLWCILILGYSISMIVFFVEMSLKRRKKNLENRAPNICIC-

>DmelIR94c

MSKVFKLLVLPLIYLSLTKGSKNPQLKFLRELINVIEEGREIRTIMVIKHSRDEYCHLDQWNPRGSPILRTNEMGSIRISGYFNDQAVILACMGENSDYGLLKSLANAMDNMRQERIILWSEREPTKMLMDYISQQADRYNFAQIIIVTMNEDVDAVPSLHQLNPYPTPRFRQITNISNIRRTSFFGCGLSFQGKTAILKESVVSNIRFKVWSPSGPIPLSELKDYEIVQFAVKYNLSLKLYDQNESKSDHFDIQLGPLFITKDFPTQMAFVSPNTACSLIVIVPCSPKWRFMDVLHKLGVLKLIGCLLIAYAVFVLIETLILWLTHRISGREVRLTSLNQLLNPRAFRGILGLPFPEFRRSSISLRQLFLVISVFGLVYSNFVSCTLSALLTKPAQNPQVRNFKELRDSGLITIMDKYTHSFIEKHIDPEFFDHVLPHYLILQKKEALRMIWNFNDSYSYVMYTTTWKSLNTVQKSFDERVFCESESLTIAWNLPRMYVLGNNSVLKWMLSRYITYMPQTGIPDSWTEQLPKVLKLLYNVTSPRRIKEGAVPLSIQHLSWIWHLLFIGESIATLVFIVEILLQKSNQHTSNMRERSSEDDDFV-

>DmelIR94d

MGQLHLLLVALVLLSPGGDSFYHSLIHHLNRELKIEYVLLLGNFDTTWLDILWQLPVSVLQIKEHSRETYSLLENPSHNVLTIAFVNDSPEDILEILYRNLRMLNTQPVLLVIRKSTIRVNSLLEWCWHHQLLKVVAIAQDFMESLIVYSYNPFPVLQFIERRLDNSTVIFEKRLENLHGYEVPIALGGSSPRLIVYRDLEGKLIFSGPVGNFMKSFEQRYNCRLVQPYPFDESAISPARDLIASVQNGSVQIALGAIYPQVPYTGYSYPIELMSWCLMMPVPEEVPHSQLYSMVFSPMAFGITIVAMVLISLTLSMALRLHGYRVSFSEYFLHDSCLRGVLSQSFYEVLRAPALIKAMYLVICLLGLLITSWYNSYFSTFVTSAPRFPQLTSYESIRHSNIKIVIWKPEYEMLLFFSENMEKYSSIFQLQEDYKEFLHLRDSFDTRYGYMMPMEKWSLMKEQQRVFSSPLFSLQDDLCVFHTVPIVFPMVKNSIFKEPFDRLILDVTATGLLSRWRDMSFTEMIKAGQLGLEDRGHPKEFRAMKVGDLIQIWRFVGWMLGLATIVFLLELICFWRHKMWQNMKYMFCRNKNI-

>DmelIR94e

MDCPKWILSGLCLISLVSGATVIELLGTLKLELDFEYVLLMKNRNFSLSDQVWNGTSLTKDVMDEVQVPVLQFNENVSYFLHNSISRRLVTLGFMSDANLDEHRGLLTALVANLRHMTTSRVIFLVQSKASTDFLYELFRNCWRKKLLNVIVIFQDFETTSTFYSYSNFPILQIEERIYETSLQTLPIFPDRLRNLHGYEMPVILGGTAPRMIAYRNKKGNVVYDGTVGHFMTAFQQKYNVKFVQPLQAKNPLDFAPSMQTVGAVRNETVEISISLTFPTIPPFGFSYPYEQMNWCVMLPVEADVPPFEYYTRVFELAAFLLTLGTLVLISCLLASALSLHGYATNISEFLLHDSCLRGVLGQSFVEVFRAPTLVRGIYLEICVLGILITAWYNSYFSSYVTSAPKQPPFRTYDDILASKLKVVAWKPEYAELVGRLLEFRKYETMFLVEPDFNRYLALRDTLDTRYGYMITTNRWVLINEQQKVFSRPLFQKRDDFCFFNNIPFGFPLHENSVFMEPVQKLIMELAETGLYYHWITTGFSELIDAGEMHFVDLSPHREFRAMQIQDLQYVWYGYAFMVVLSSLVWLLENLAYTVKSKTIFPTHFMQRNKK-

>DmelIR94f

MSGMWQQVLLAETSNWFRSDVLQRFWTHLRVEIRFRTMLNYRLESCDCWFDNVLGSDNSTALLWNDQTYPHYLRRRQDTDILVVSCLRFHQYQEVLLALSLMLDQMRSMPVVLQLCGDEDSMQELNSARLLLKHSQDLKMPNVVLLSSTFFTSATLYSYEMFPEFNVQKLVYQAYLTLFPYKLGNLKGHPIRTVPDNSEPLTIVRKTLNGSIAIDGLVWQFMIEFAKHINATLQLPIEPHPEKSIKLVQILDLVRNQTVDIAASLRPYSLNVQRSSTHIYGSPMMVGNWCMMLPTERVIGSHEALTRLMKSPWTWLILLLFYSVHRFLAQKTRLRSSLIHLIKLLINLSLICFLQAQLSAYFIGPQKVNHISNMQQVEESGLKIRGMRGEFMEYPIDMRSRYASSFLLHDLFFDLAQYRNSLNTSYGYTVTSVKWELYKEAQRHFRRPLFRYSEEICVQKLSLFSLIQQSNCIYCYRSRIFILRMHEAGLIRLWYRRSYYVMVTAGRFPIGDLSTVHRAQPIRWTEWQNVVLLHGVGLLFSVVVFVIELTVHYANVCLNNL-

>DmelIR94g

MSTAVNSVHSKLVSLISRGQELTSIFFYAPAKEKCHLEDTISSATWGLPLVIWRTDRTVILNGFIGEGLLVLACLPGFHWRALLGSLARSLKYLRQARILIELMQDRDEFLVSEVLQFCLSQDMINVNAIFDDFPETENLSSFEAYPSFEVVNQTFTPDTQVSDLYPNKMLNLRGGVIRTMPDYSEPNTILYQDKEGNKEILGYLWDLLEAYAHKHNAQLQVVNKYADDRPLNFIELLDAAQSGIIDVGASIQPMSMGSLSRMHEMSYPVNQASWCTMLPVERQLHVSELLTRVIPYPTLALLLLLWIFYEVLRGRWRRHSRLQSIGWLVLATLVSSNYVGKLLNLFTDPPSLPPVNSLAALMESPVRIISIRSEYSAIEFTQRTKYSAAFHLALHASILIGLRNAFNTSYGYTITSEKWKIYEEQQKRSSKPVFRYSKDLCFYEMIPFGLVIPENSPHRAPLHSYTLLLRQAGLHDFWVNRGFSYMVKAGKINFTAVGERYEAKTLTITDLRNVFIIYVSVLLISLILFTCELFVSWVNYWLGF-

>DmelIR94h

MLSNISFSSAPELVDLYGLVLKFLVSSETTLFYFNPTGQKCSWETLPRTILSNHPQIIWFREETYPGLYKRHSSNLFVMACLSSTSYDGQLQLLAESLTRYRSVRVLIEVQDKEGSFLASQILLLCQQHSMLNVVLYFSRWTRTLNVFSYLAFPYFKLLKQRLSGSLRPKIFINQLKDLQGYKIRVQPDLSPPNSFSYRDRHGECQVGGFLWRIVENFSKSLKGDTQVLYPTWAKAKVSAAEYMIQFTRNGSSDIGVTTTMITFKHEERYRDYSYPMYDISWCTMLPVEKPLSVEILFSHVLSPGSALLLILAFILFFLIVPQLIKCLGITFRGRLIGMASRIFALVMLCSSSAQLLSLLMSPPLHTRIKSFDDLLTSGLKIFGIRSELYFLDGGFRAKYASAFHLTENPNELYDNRNYFNTSWAYTITSVKWNVIEAQQRHFAHPVFRYSTDLCFSSETPWGLLIAPESFYREPLQHFTLKINQAGLITQWMTQSFHEMVRAGRMTIKDYSRTNLMKPLRIQDLRKCWVIFAVGLGTSTVVFTIELLLIYTNVFLNSL-

>DmelIR100a

MATTLQLIMLALVGGTLGQANNTDHKQVLTSIVKQLEGGLELHLRTSEDGGNDLVQFLMQEKSSIIISAKQEEVPSRAKIMRHHFFIFDGVHQMQEIRTSLFNTDGFYILALENNTIEDDVLLMEFAADVWLQHGHSRIYYVQLSKKSVLLFNPFLQRLVVVQDSKTYSRIYKDLEGYHLRIYIFDSVYSSVIGDGENKVLSVTGADAKLAKTVARQLNFTADFVWPDDEFFGGRLANGEYSGGVGRAHRGEVDIIFAGFFIKDYLTTHIQFSAAVYMDELCLYVKKAQRIPQSILPLFAVHMDVWLCFLLVGLLGALVWLILRAVNLILGIEGVPDGSRATRISYFGAARRIFVDTWVIWVRVNVGRFPPFHSERIFVASLCLVSVIFGALLESSLATVYIRPLYYRDVNTLRELDESGQPIYIKHPAFKDDLFYGHNSEVYRRLDAKMMLVAEGEERLIEMVSKRGGFAGVTRSASLQLSDIRYVMTKKVHKIPECPKNYHIAYVLPRPSPYLEEVNRIVLRLVAGGIVGLWTGEAKERAKWSIQRFPEYLAELDVGRWKVLTLSDVQLAFYALTIGCLLSAIVCMAEILLGRQRRLHSPK-

>MmedIR1

MFSAPTKWLILQDIRYDNDSNSSKNYNDQENKLKMFSAPTKWLILQDIRYDNDSNSSKNNDDQENRLKDKLKDFEIFPDSELFISQRFDDDKIKILSIYRPSFYVDLIIEEQATWDINNGINFFDSIPTSRRRRNLQLTPLKTCLVLTNPDTLNHLTDYKDKRIDAVTKANYIWMMHLVNQMNATVTYTWRDTWGYPDKNGTWSGMIGLLDRGEIDFGGTATFLIKQRIGVIEYLQLYTPVGSKFVFRKPPLSYVSNLFTLPFGHTVWYAIGVMSCIVLGFLYITMKWEWKEIEKSPGEQDELKSNPTISDNLLILLSAISQQGLSYEPRTISSRIVTFMLLIAALSLYASYTANIVALLQSTSSSINTLKELINSGLKLGIYDIVYNRYYFGVLDDPIRREFRERFVTNKTSIWITLEDGIEKVRQGLFAFHVDTAAGYQLMQETYEEEEKCGLQEIDYMGVLDPMLVIKKKSPYREIFRVGSLWLRETGLQQRDTPRLFTKKPVCVGHTSFISVGTTECYAAFYTITYGAAIAFGFFFLEIILYKCFGNKMTDDEESTECDADIEINTTEISRQSSESLEATLE

>MmedIR2

MRGIELMLIGIYALTSNNTIDYTLPGRYDWEENLEEVIKKSFLTSVQKEQEYNEIPKEIKVTSWDESLYSSISEVNGEYKGGGYALKVFDIIAAKLDFKYKIVLPEKPILGDNVTGVIGMLNQSKADVAVAFIPIIPEYLRFVQFSPILDYLEIAVLLERPLQSAIGSGLLAPFSRTVWICIFVSLALVGPIIYIVTSYRAYLWGRTKKDKYKFIDCVWFAYGAILKQGSALTPEADSNRLMFASWWIFITIITSFYTANLTAFLTLSEFTLAFTSVKDIVSQHKTWSAQQHYIVDITINRNDPEELTPLRTSYRQHRGIFYHISNKDNKTEKVASYLTNRRLFIDEIQFVENFIKNDYLNRTRQNMVEKKKCFYIKMPNPVFQQNRGFAYPSNSTISKIINRELLHLSESGIIQHIESVNRPMVTYCPLMLGSTERKLDNNDLELTYQVIATGFLFSLISFIVEVSQHYFRCRVCLCCADSCTCCKTRMKEPVSPSKRPQLPLPPPPTITTVPIKTEFSSFTKPPVIPIYDTIDNFKNSKIHYVNGRDYFVVHETTGERRLVPVRSPSALLFQYNH

>MmedIR3

MNDILPEAEKAAGGNDIVKVDVEKVELDRNNVSESLNQACAVLSRGIAIALDMTHTGWDKLREIIANNSIIYLRSDGSIIPFIQATDEFLKKKNATDFALIFENPSDVDPALYYLLGASTLRLVIIDNLNNASISKIHDMKPQPSYYMIYSSTKGMEKLFQAAIDGNLVTRSGVWNLVFTDMDYKQFRYIRGPDVLDHNIGIFSMNSTVCCTPLEKIICECPPDFKIFDQYLQVLMKQLVATFVKINSESGPKVRSIKGQCPLVEKPAEEDKIIQVFKDAIIEVTEADPLHTLEYMPKSSIVVHECLIDIEILENGTLNLLAEWTKDAITPVAGQVIAASQPHFRIGTTASIPWVTLHTDPHTGEVLRDANNNLMAEGYCIDFIRELSKKMKFTYELVVPKDRKFGDKLPDGQWTGLVGDLATRRTDIAIASLTMTSEREEVIDFVAPYFEQSGILIVMRKPVKETSLFKFMTVLKYQVWLSIVGAIVATAIMIWILDKYSPYSASNNKQLYPYPCREFTLKESFWFALTSFTPQGGGEAPKALSSRVLVAAYWLFVVLMLATFTANLAAFLTVERMQVPVQSLEQLARQSKINYTVVNNSNAYQYFSNMKNAEDKLYKVWKEITLNSTSDQTEYRVWDYPIKEQYGHIFQTIKTVGTVANASEGFVKVLDSTKAEFAFIHDSAEIQYQVTKDCNLTEIGEIFAEQPYAVAVQQGSHLQEEISRAILDLQKDRFFENLSAKYWNASLKDKCPTSNENEGITLESLGGVFIATLFGLALAMITLGIEVLYHRRKNNLNDDKAQEANNKSVKKIPSKLIKPKLHQFKPAPTVAFIGRNRGLRSRVSHISVYPKTFPYKD

>MmedIR4

MGTMGNWFWWNYIFLTNFLIVFAQQNQPPTGVRPVNLFIINDEENSLANNSVKNALMGIKDKDKTVLGNVIVVQINGSDPRGSLDKVCAAWDPAVRDGGPGVPDLVLDTTRSGFGAEAINSFTASIGVPTFSSQFGQKNDLRPWNNLTPDQKNYLVQIMPPADLIPNAIRQLCSQMNITNAAIIFDKGFVMDHKYKSLLLNIPTRHVIVQSKIDTNEILNQLQRLRDLDIVNFFVLGEEVTLKSYLDAAESKNFTGRKYGWYAFTMNENLDLKCECRNMSVVFFKPILTTSNQQHLSTLTTQGLLPAPWLASAFYYDFVRVGVEAIRSVIKNNKWPNEPYHITCDEYNGTNTPIRNLDLLRHLQEATTNGFEPTFAGFYWGKSNGEHHAEFDMRIQMVVIENGNIVSADDLGTWKADIESPLNLTDTESKVAKHTAVTSFRIVTVRIAPFIDINATGHWEGYCIDLINEVQKIMNFEYEIYESPDNKFGTMDESGNWGGMIKELIDKRADIALGTLSVMAERENVVDFTVPYYDLVGISILMKKKKEETSLFNFLTVLNQTVWLCILGAYFFTSLLMWIFDRWSPYSYQNNKEKYKDDEEKRVFHLKECLWFCMTSLTPQGGGEAPKNLSGRLVAATWWLFGFIIIASYTANLAAYLTVARLEQPIESLDDLSKQYKVQYSPINPSEAYTYFKRMADIEERFYEIWKDMSLNDSMTEIERAKLAVWDYPVSDKFTKMFQQMHEAGFPKTREEAIGRVRKEIPKYNNTDFAFIGDATEIRYLEMTTCDLVMIGDEFSRKPYAMAVQQGSPLKDQLNNAILKLLNQRKLETFKSKWWSRNPYKVDCTKKDSQSDGISIQNIGGAFIVIFVGILLACCTLAFEYWYYRYRPRAQAKKLKNLNSKNGGGNKISSQRIKPTRFNLKPARKAFEDSGTEFRARF

>MmedIR5

MKSRTSVYLLFIIFLALSLSAIEGYDVLAKFTGDYFKKFYVTQIVVFGCWENHISVEFSKSIMLNTHSKLMYVNINDNLNLDKLLKVDYWSLGIVLDLDCQRSHIIFNQFSEQNLRHNESYFWLMPTSKEKIPDYFHKLPLNIANEMTLAVRKNNNLQNSGNETNNNNISYILYDVYNPSYRHGGKLNVTYMGHWSLNDRDEGTLTISLTQYKYKRRGNLYGLVLNASIVVDHPPVPDYNTYIHNPINPHLDTMHRYNYALTLQLRDYYNFTMNLKRGSTWGYLINGTFNGIIGDMMKGLVDFGATPFQYKPERLDAIEYTVQAWMARPCFIFRHPTTNELSNPFLKPFEMKVWYVIGIFAAVNWIFLYTSVKLEHKLVMKQPVCTLDTYPASEIIMITTSAICQQGLSDGPRFYAGRIVFIFLFIWGFLLYQFYSASIVGSLLAGKPRWINTLQDLADSNLEVGIEDIAYNYDFFATTTDPVAQQLYREKVAVNKKRKREPYYTIEEGLQRMQKGGFAFHVDVATAYKIIEETFDVNEICDLVEIQLFPPKHTATATARFSPFKKMVTYGLRQVVEHGMARRLRNVWMHRKPECPESHKDDPVPIMITEFSPALFLMSCGWFVSMCIVIGEKLAMKKQEIGEMKDGDADAKGYEDPEDFDRETASTNSQTSRKSG

>MmedIR6

MYNFHWRNILAFILTQCLIQTLQQNQPQNTVRSVNLFIINDEENIIANNSLNNAITNIKDKDPTVLGNVIVVQINGSDPKPALEKVCAVWDPIVRKGGPGVPDLVIDTTRSYFGTKTVNIFTSSLGIPKISGRYGQQSDIKHWGNLTADQKNYLIQIMPPVNLIPKAFRQLECEINASNAAIIIDNNYVRDPAFKSLQQNISTRHVIVQAQINNDDIDKQLNRIHDLDIVNYFVLGRENTLTNYLDVADDKNLTGRKYGIASNQQYLRKLTAQNLLPAPWLASTFYYDFVHISVEAMRSAIEGNLWPKEPRYFTCDEYNGTNTPMRNFDLLSQLRNATTNGIKPTFTRFHWGRSNGEHHAEFNRRVSMVVIGDGNSIYSDDLGLWNSEIRSPLNLTENFDTGLAHYDSIPIYRIVTVRRPPFIDFNNETNDWEGICIDMLKEMQKFMRFEYKIYESPDDKFGTMDESGNWDGMIKELMLDNADIALGTISVTAAREYVIDFTIPFYEPVGYSAITKRLLDRTRLFSFVGSLSVKVWSSAIVGFFTSSILIWVFDRWSPYSYRNDPNGRFSENYVRKFRLIDSFWFVFSSLSPQGGGIPPKNLSGQMVAGVWWLFGFITLAAYSANLAAYLTVSRLQPILKSWDDLKEQFDIQYSPVNPSDAYTYFERLKDIEERFYFVWKDISLNESLTDRERAALAIWEYPVDDEHTRLLGQMNEYGFPSSTEDGINRVLGLPPYPNDSSYALIAEATTVRYLAMTDCRFLLLEGEFSRKPYAIAVQQGSPLKELFDNAILKLLTEDKISELKKKWWDNNPKRQPWCPQRINKSDGMDIRQLGGIFIVLWVGIASAIVTLAVQYWWYRYRSRMRFKKDLENAGSLDNVEPKKFKVKPALRRISRVTNN

>NvitIR1

MNATAKPTYRVITIPKPPFVIYDPDSNWYGGFLVDLLNEIARRLNFRYEIEMQNESEYGFMDDQGNWNGLMRDLKEGKADIGLAAVSVMSERMKVVDFTEPIYKPTGISVLMQKPIPKTDFYRFLTVLELDVWLCIIGAYIFTSLLLWIFDTWSPYSYRNCKAKYKDDTEKRIFGCKESLWFCLTSLTPQGGGEAPKNLSGRLVAATWWLFGFIIIASYTANLAAFLTISKFEKTIETFDDLISQYKYSYTCIQNSSTNRYFQRMNDIEYVCYEKWKDMTLNDSLSPYERAQLAVWEYPLSDKFIKIYSAISHHGMVASLQDGLDKFNSTDSRFALITEASDVQYQAMIDCSVKEIGPEFSKKPYAIVLQKNSPLTKQFNRIIYNMKNDNWLEALTDKWWKYNPLRQRCHDKDEMTNGIIFENIGGVFVLIGVGILSAFSTLVYEYFYFKCLQDKFERIFEHKLKSIFRRQKNFARSISVKP

>NvitIR2

MQVYGFQISQETLQLIILVSKLFDMFIMFILDASYRNDLSSAVLKLQEKGVLTSLKNKWWKEKGGGGRCQEDSSGNQAKELGLANVGGVFLVLIVGVALSFIGTIFEFMYSIIINSNKNKSIKIKDNLKSELNFIIKFGVSTKPVNDLDL

>NvitIR3

MQHFNPSRSGNAAMGSQDEPPPFPSHIKITTYNDWPFSRYREENGTIIGEGYAFELLKLLIRKFNFTYTIVPPREDIIGNEERGMLQQIYDGEVDMAVAFIPILSSFRNICDYSAPLDEMDTTFLLKRPGTSATGSGLTAPFSTRVWYLILISLLVVGPTIYLIIYLRGKFARDEKAEKFTFLTCMWFVYGALLKQGTTASPMGDSTRLVFATWWIFITILTSFYTANLTAFLTLSRFTLGVNSLDELVYGRYSWVIVNGRSIHTLLPIESDDTRMLVKSKSWGYGYVTREYNMSYNSILKKVKDGRVFILERTLAQMAIFEDYRNKTRDAMDEERKCTYVISESNVLARPRGFAFPVGSTIKQHVNSEMIPAVEGGLVKHFKLEKLPQAQICPLNLKSKERRLKVSDLMMTYKVVFAGMGVGVIIFLMELFTIFIRWVARHEAFRNCCRRNEVKGPSEPEDPWDYPKKTDTNFWIRTPPPVYQMNEEVSHKTYSINGRDYYIVKEETGDQRLIPIRTPSAYLFQYIH

>NvitIR4

MALLFALIVIMVSTAMSLPPVIRIGAIFTEDQKDSPSELAFKYAVYKINKEKILLPNTTVVYDIQYVPKDDSFRTSKKACKQLSRSVQGIFGPADPLLGAHIQSICEALDVPHLEARVDFEPSFKEFSINLYPSQDHLNRAFRDLMSFLNWTKVAIIYEEDYGLFKLQDLVKSPPSPKTEMYIRQAGPGSYRQVLREIRHKEIYKLIIDTDPMYMPQFFRAILQLQMNDYRYHYMFTSFDIETFDLEDFKYNSVNMTAFRLVDTEEPSVAETLRQMERFQPVGHAILNRSGIIQAEPALVYDSVQVFAHGLAALDRSHILRPANLSCEREEPWDDGLSLYNYINSAGLQGLTGHIEFNEGKRTNFKLDLLKLKKEELVKVGEWKLGSGVNISDVGAFYETTATNITLVVMTREEKPYVMVREDKNLTGNARFEGFCIDLLKWIAGQVGFQYAIRLVPDHMYGVYDPETKEWNGIVRELMEKRADLAVASMTINYARESVIDFTKPFMNLGIGILFKVPSSQPTRLFSFMNPLAVEIWLYVLAAYMLVSFTLFVMARFSPYEWNNPHPCMGETDLVENQFTISNSFWFITGTFLRQGSGLNPKVSNRMPSRLFSFMNPLAVEIWLYMLAAYVLVSLTIWIVARFSPYEWAEPAPCPACKCPLQGGHVGSLDPEAEDLALPHNVNDFTLANSFWFTVGTLMQQGSDLNPKATSTRIVGGIWWFFTLIIISSYTANLAAFLTVERMITPIENAADLAEQTDIPYGTLEGGSTMTFFRDSKIAIYQKMWRYMESKQPSVFVSDYEEGVKRVLEGNYAFLMESTMLDYAVQRDCNLTQIGGLLDSKGYGIATPKGSPWRDKISLAILELQEKGVIQILYDKWWKNTGDVCNRDEKSKESKANALGVENIGGVFVVLLCGLALAILVAILEFCWNSKKNAQSDRSLCAEMASEFCFAIRCGSRQRPASKLRATSSSGPVVGCSRCSSSSMRRSRYCPASHQTSSSIAAVGHEETTYVPSIEIPWLNGQEAGALQMTEMKKSLSYDQMTGEPGSGDT

>NvitIR5

MVRLQGPLWLWLLLPAVSLGGGGGGGGGGGGGVQRVGSSIKIGKSERSSSTLTGRGSSSIKIGEAAKFLNSTRVLRPALSTSMTSTSIPLAYASPQPESASSEYGNSNVTKMLKVGLAVPYKSFGYREYTRAVSRVVTALQKSTKRPNLGLFQHYDIFVKVAMQELTPSPMNILNSLCKEFLSLNVSAILYLMNYEQYGRSTASAQYFLQLAGYLGIPVIAWNADNSGLERSSLHLQLAPSIEHQTAAMLSILERYKWHQFSVVTSQIAGHDDFVQAVRERVSETQERFKFTLLNAVLWTKTKDLMELINSESRVMLLYSTREEANSILRDAHELKITGENYVWVVTQSVVENLQPRHHFPVGMIGVHFDTSSSSLVNEIATAIKVYAYGVEDFVNDPSNYGISLNTQLSCDDLEGESRWSTGEHFFKYLKNVSVEAEYGKPHVQFTQDGLLKSAELKIMNLRPGASMQLVWEEIGTWKSWEKDGLDIKDIVWPGNMHTPPQGVPEKFHVKITFLEEPPYINLAPPDPVSGKCLMERGVHCRVAKESDLQEADLQTAQRNGSSYQCCSGFCIDLLQKFSEEMGFTYELVRVEDGKWGTLENGKWNGLIADLVNRKTDMVMTSLKINSEREAVVDFTVPFMETGTAIVVAKRTGIISPTAFLEPFDTASWMLVGFVAIHSATFMIFLFEWLSPSGFGINDSSGYSNRKPAGRSLRHRFSLCRVYWLVWAVLFQAAVHVDSPRGFTARFMTNVWAMFAVVFLAIYTANLAAFMITREEFFDFSGVDDHRLARPTSHKPMIKFGTVPWTHTDSTLGKYFREMYAYMKNYNKNSVAEGIEAVINGDLDAFIYDGTVLDYLVSQDEDCRLLTVGSWYAMTGYGLAFPRNSRFLKMFNQRLLDYRDNGDLERLRRYWMTGTCRPDKEVQKSSDPLALEQFLSAFLMLMVGILIAAILLLLEHIYFKYIRQHLAKDSRASKCCALLSVREMLNPEMDVKT

>NvitIR6

MLKFLLLLLALTLGLRCRGVEGNDRVASTVIGALFPPNSNMRNVFSRAVWLMNNCQVNRELCEKLAPCDDDINCLLFHRLKISALMLETDQDLLNNYAKFGELAGHRSSHKTPNSHKMLGNYYGLAAIFGPHRGLASKYIQGLCDIHDLPQIVIRKEFYHDRLMSINLYPDTQTLGNVYVEMVKKMNWTSFTVLYEHAEDFIALNKLLGVAKNLNTTDFPIFAFRLGSGPNYSEPLLQAKEKDAKSIVIDCSHEKLSEILKQIQEVGMMTNNYQYMITSLDLQTVDLRPYQYSGVNITGIRILDTEDPFVQKVVGENLEQFNLPSIDKMLTQEALMFDAVALFAQAYKDLIYGYRDLKGVVLSQKYSEPQPWKHGLSMRNYLMYNTINGLTGPVMLDSDGSRRRFKLDILNLHKTGLKKVGIWNPDDGFDDVNLLNELKLKHFNILITMNAPYAMKVNFSKSLEGNDRYEGFVIDIIKELSAEIGFNYTFHVQEDKQNGNCNKTSEYAACTCTGMMHKILTGAMDMAITDLTITEERAACVDFSTAFWNLGMSILYKKPKKAPPTLFSFLSPFDMWVWIGLVGIYALVSLLFWVLGRLSPAEWTNPNPCIEEPTELQNQFTLNNSFWFTLGAIMQQGSEIAPISVPLRLLSGCWWFFCLIFVSTYTANLAAFLTIEKPVKVVRGIEDLYNQTAIKFGAKKDGSTFMYFKSSKNIKHRQLAEQMMTKDFERYMVTDTEDGIRLAQEENYAFIMESSSIEYIQYRKCDLEQAGPLIDQKSFAIAYKKNFEYHQQISRTISMLQERLVIKELYDKWWKERGAICFNEPSSTAEAMNLDQLNGVFLVLLIGVVISLGLTFFESALGIFVVSKKEKVSYMQTMKSEIKHICSSKATKPVLRRDRSSDCSLSEVSCDYTMTTQI

>NvitIR7

MLQLAMKKMKLNKFYSFLIIVCTCTIISTTAAVQNIKIGAIFHKGDEDLILAFDEALRDLDNEQNKIFNLKAVKKINIEFESFKVATVVCELLADGVAAIFGPKFAQTRDIVSSIASRFNIPHIEFSFREISENDTSANSINIYPSSKMYGKVIAKSVKHMNWHSFTFVYQTDEALSRIQETLKNQGRPNYPITIRELQPLYKINLLASEKERDNIYESLVKEIKSSQQYSLLLDVENENLSQLLDVMNKMDLMSDYFMCLITDLVEAALLYDSVFAFNDAISNSYKDYDSSRNIKKIIPKPLSCTGEAKYEIGLNITRNFIKKTKFGKKTGYMSFTAKGERLFELDIINIQAGRSVTVGKWEKEDLKINRDEKEINNTLTEAAKSKIFKVTTRVGPPYVMLAEDETKGRSIGDKKYEGYCIDLIGKIEELLGIRCEFEIVPDGNYGSVNTITKQWNGLIKQLLELKADFAICDLTITSERQSAVDFTAPFMNLGISILFSKPEKEVPKLFAFMSPLSTEVWMYMATAYLIVSLMLFFQARMSDPSIDEDKLLQSDKETVELTDYVLEISLSEMADKKRQTTR

>NvitIR8

MLKLLCRQATFVLLFSIGNRALEIDPEDYVPFLSDVQEYYKSSGIIFLYDDDSDYFQTLTSLGIFFKLLSDRGILSQAIRFSKIREKYDLSRNVQNLFVVFMTSRRNFDNFCLATWDHDMSYHTWLVIFADSVEAEVKEICLKPSGNPFHLVFNSRMIVRCHNEDWIRKWYSLQENQTEVLDLAKWNRKLGISYKNKNYYESRYDLMGKTIRIATHTFMTSSTNGKMSGFLEDILQELENFMNFTVARVREEDAFGKWDNEAHRWTGLIGSLTNREVDLVVAPITINKFRLRFIDFTMPLLLSKNRIYIKQPDGARVQWSAYFKAFSISSWSFIALVLIAASLMTALIRLIVHETKVLQLSLNYLLENFIRVWGIYCQQGIPEFPSSTSLRLAYFTMLLLTLIIWAIYSASITSYLTFLSPSLPFSDTEGFVRDGSYKLIVLQNSSDQDIILSGLDPILATFQPLLKDLDELPKNPHSGFLQVCNERVGFYASEAVLKGVSGYIPCTLTYIETGQFECLALALRKRSPYITSINYNLRKLEDNGVVKKLNNWYFHKVDNAVESTHSPIGIWGVAPLLTIFATGLALSSLVLLLECLCLPNGKVAHETKNRQRVFYPK

>NvitIR9

MKYNHYQEIVDFAALVYDINDFYKSSGVILVASRDILNENWACQKIALNLIKQFSYHSITSIYMDIDYFSFKNVMKLESEISQTPSKEPLIVLINIDCKNPQGNPFSLQMNTRMMVLCQNHVYIKKYYAILANTTEVVDLATWKPGEHVVFQNDDNLLQVRKNMKGITLRIAKHNHSSIERNKNVEDYLENVLKALENVSNFKTDTVIQTDALGHFHNNRWTGLVGTLAYKQVDLALALLSMSSQRQIDIDFPMPFFQTQIKIYAKKPKTTSIQWSAFFKVFHLQAWIALALLLLLITLLLTLIKSYFKNKSVTMNLFFENLVNVWGIYCQQGLAENPFNIPIQAIYLSALVLSLIVFSIYSATITSYITVLKTNLPFSTYEEFVEDKNYKLVVLKDSRDQTLMDKGDWLLKPLKDRIKKYEELPTNPYDGFEQACEDNVAFYSDEVMFDGISLNTKCVLKSLNMPRKENQAIALVKNSPYTETFNY

>NvitIR10

MWHFMENANPSNEVFTNSNVEGVDRVVKGKGSYAFLMESTSIEYAIERNCELTQIGGLLDSKGYGIAMPPNSPYRTAISGAILKLQEEGKLHILKTKWWKEKHGGGSCRDETSKSSSTASELGLANVGGVFVVLMGGMGIACVVAVCEFLWKSRKIAVEERTNMCYSD

>NvitIR11

MKGDHPGFLLACCVLLTVHLLQLASDVRALPPVIKIGAIFTHDQRNTSTELAFKYAVHKINKDRIILPNTTLVYDIQYVPKDDSFHASKKACQQVKFGVQAVFGPSDPILGQHIHSICDALDIPHLEARLDLDSEAKEFSINLHPAQSLLNAAYQDVMTFLNWTKVAIIYEDDYGLIKLRELVRSRKAQDMEVYLRQADLDSYRQVLSEIKAKEVHNIIVDTRAENMHHFLRMILQLQMNDYNYHYLFTTFDIETFDLEDFKYNFVNITAFRLVDAEDVGVRGILRDMEKFQTEGNNLLNKSRVIQAEPALMYDSVQVFAVGLRTLEQSHALRPMNISCELEHPWDGGLSLINYINSVEMKGISGPIEFKEGRRIQFKLDLLKLKQHSLVKVGEWRPGLGVNVTDTSAFFEPGATNVTLVVITILEQPYVMLRSRGNFSGNERYEGFCIDLLKEIAHMVGFAYRIELVPDGKYGVYDYETGEWNGIVRQLMDKKADLAVGSMTINYARESVIDFTKPFMNLGISILFKVPTSHPARLFSFMNPLAIEIWLYVLAAYILVSVTMFVVARFSPYEWNNPHPCHSGPEIVENQFSLANSFWFTIGTLMQQGSDLNPKAASTRIVSGVWWFFTLIIISSYTANLAAFLTVERMITPIENAEDLASQTDISYGTLESGSTMTFFRDSMIETYKKMWRFMENRKPSVFVPTYEEGIQKVLQGNYAFLMESTMLDYIVQRDCNLTQIGGLLDTKGYGIATPMGSPWRDKISLAILELQEKGEIQILYDKWWKSPSDTCLRNDKEKGSKANALGVDNIGGIFVVLLCGLAFSVLIAIFEFCYNSKRNAPAERQRTPIPPPTCSGSLQGIPQAAQMQQQSQQQQESLCSEMARELCRAIRCHASSRRRKACEKCSTHVVGYPIDNSTPTPINGVRSQRSSLRLDMSPHIHMHHHSPQQDYDGN

>NvitIR12

MIRDLENHNFQRARITVAYYQERNVVSFDDNDTEISGVCGDLWTILSDYLNFTLVPVRLEDKRFGTKLKNGSFEGLLGMIEQNKALVIPRTGVFINRLSLLDYTLPLWTVRYYLYIRPEWKHDEAWIFHLFTVKLWYSILLSFLVICIVGYLYEKYTNLKGIRVQHQTNFQDHLLYMVALASSRGGIPSHYYCRSKIVYITTTMFSWFILIAFSSNAIFLMMNKKYILPFTGLRSLIQKSKYEVIAFSGSMVHEAFDKLVSRYYKRTSDATRVSYTSSTFELYQKVCFSNSIKFAAFDSEDLHKANGKYFCRLVHTEHPYFETWIASAVKRGFRYKRSFDNGILKLAETGILDGLKDRWMNWKLTENEDKPFQSIDISQVYMIFAILFVGFTLSILLLLIEYVIFFRYKICRTCIKKYPFKMEVKKNEKKKITFMLK

>NvitIR13

MSLVLLLLLCYLSTLLSAPATSSKTSDTGRLARLLEYAGPLYSGAFSMVTSFSCSDVDGRFELLRDLSRIQVGSNVLDLEKSVDRLSEIAWEKGYAGQNEIPSDAHRVLFTLDLDCPKIDDFIRKADREYMFAAPFKWLLTQRSNDTDDLVSRFAEVGAYPDSEVTVWQKETGQLLSIYRTNGNNDHLIEDRGSWMEEDRRMLVGDTNVTSRRRKNLGGIGLKSCLVITDPNTIHHLDDYHDKHIDTITKCNYPWVLILMNMLNATVTFEPVGSWGYKGPNGSWDGMIGMLQRGEIDFGGTGCFLTPERMGAIQYISLPTPTRNRFIFRRPPLSSVSNLFKLPFGNSVWFGSCALVIILITMLYPAMKHEWMQFNEVEKAREPIPPNLSDDLLVIVGAVSQQGSWYEPRSVSTRIIVLFGLLAALNLYAAYTANIVALLQSTTGSIKTLQDLLDSPLMLAAHDNVYNRYYFKSFKDPIRRTIFEERIEPRTSKKTNWLTIEDGIEKLRGGYFAFHVELGPAYKLIQERYEEDEKCGFQEIDYLNVFYAHLVVRRRSPYLELLRVGAMRLYETGMRTKDINRLYTRKPECGGSSSRRFLSVGLAECYGAFSTLGYGLALALGVLIAEVVSTKLKVRCKSSQLSSTNY

>NvitIR14

MMLIRCYDSPMIREWYSIKSRTEVKSFDLFEWTLKRGLILKSDRQLYQRRSDFGGAELKLAAIKEGKSGPTISNGKISNIDVFQHMMLEVLQQMNFTFQLLLAVDTFGSWDKTANRWTGLLDKIDSGEVDVPFTEFTILKDRAEYFDYTWAISMPRNSLYIREPEMNNFKWKAYLRAFSSDVWISLGALLLASSIMLYYMKVKIEKTNLMGNSFMKNVLQVWGIYCQQGIPNFPRASSLRTAIVFLFFSQIIIIAIYSAFIISDLTTLSTTLPFETLSEFVEDGSYKLTILRNSAEEIMFSTSQDPVYSKMSKMLKPHNELPVSGIEAFRQLCNEKLAFYGEENKVKKISTSEYPCKLTMVFTNKNRFMGFPLAKNNPLRDFMNYYIQRFKENGIMNRIKNMHAQTHDNTVNGFHPVEFSDISVALAILFLGFVFSILLFLLEQMQYRIRICKNSGYTVV

>NvitIR15

MLVKCYDDAELREWYSVNSSNKVITTDLMEWRRETGLVLKTRQNLYQRRYNVGGTVLRMSSVKEHQSLLKNQANGEGVAMFAKIMIELATQMNFRLRFVSSEDAYGVLNSSNNKWTGILGRLQSGEVDIATSEITMTKERMDAFDFACPLIITRAKLFIREPGGADVQWNSYLKAFSTSIWVSLIILIILSAILLTYMKTKIAKIRCLKNYLVENHLYVWGIYCQQGLSEFPDSSSLRIAYVSIFFSAVVLSAIYSASIISYLTIFTPSLPFDTLASYVGDGTYKLIVVRHSAEHEMFATSHNHELKRMSKLMKRDKDLPVTDIQALEQVCHERVAYYSTDAMRDKMGDMIPCNLKGISTGRIENLAFTLTKNNPFTEFINYHILRFQDNGIMQRMRRMYYKKLNKPTKDYDAVGLWGIAPLITILILGTILSVLMLFIEKLYHNMSSQLDDDGDNDSSSTAKSQNLRDSLTWRRRRKSTRSVHSCCHC

>NvitIR16

MLLALLVLLAGWIEIGTGYNDFPSLMTANATMAVIVEKGFFKSADNYRHTLDEISDVANAVIRKNMEISGIALHVFGDADVNLARDYTVLLSVASCQTTWHLFKRAQKEKLVYLAVTDPDCPRLPEDAGISLPLTNPGEELPQIFLDLRTTGSLSWPKVNLIHDDTFARDTISRVVKALSLELPDKRVSLSAQALFSTRFEKNENAMRQRVHRILSNYHVDQLGSCFMVVVTVDMVSIVMEVAKSLRLVHPGSQWLYVISDAAGREAKVTSFAELLAEGENVAFVHNATKHVANCNMGLMCHVKELVRALAISLENSLLNELELYDRVTEEEFEVVRLSKAERKQEIVKSVNRELSYARAHTSSCGKCVNWRFSSAITWGTSFASSEEKQRRESGEKRRRENSKRHSEDDLGEKSLGLGELLDAGTWSPGPGVNMSEPLFPHVEHGFRGRSLPVSTFHNPPWQIIKYSNTGAQEYGGLIFDVLNYLSLKLNFTYTVRLASSPAAEAPTRLPSAGDSSKSMDLAAMSVAQKVPQEVVELVRSKQVFIAASAFTVGKNSGGLNFTAAIVMQNYALLSAKPKPLSRALLFTAPYTNETWACLTSVLIVIGPILYLTVKLSPRPRDIDNSLSLSTTWQCSWYVYGALLQQGGMSLPKADSARLVIGTWWLVVMIVVATYSGNLIAFLTFPRIDAPIDNVDDLLARSDAFHWSFPNGSALESYLIAAVNDDPKYKQLLDGAERQDPSKPKQILDRVKAGNQVLIDWRISLAFLMREDLIDTGGCHFHVSAEDFMHENMAMIISGDSPYLPLINDAIERMHESGLMKKWITEKMPMKDKCWEIAKTNQEATNHKVDMGDMQGIFFVLAIGFVIAAIAIGVEFAWHKRKEAFERSLIRPFVS

>NvitIR17

MIRQESYFKLKKYPNFRTEHALIYDSVKIFAEGFKRLKSATKGNLKKIVCNKTESWEHGISLNNFIKSTKPYGMLKETVSKMSGNEQYEGFAIDIIHEISKMLGFNYTFSVQMDNVYGSLNQDTKQWNGMLRKIIDDEADLAITDLTITEEREKAVDFTMPFMNLGISILYQKPKAAPPSLMSFLLPFSTNVWLYLIGVYIVVSTLFFVIGRMCPAEWNNVYPCIEDPDELENQFTFRNSLWFCIGAVMQQGSEIAPIGNSTRMLAGCWWFFCLIIANSYTANLAAFLTVETIDRPIKNVDSLANQNIIKYGAKKGGSTLDFFKASNSSTYVKMYQYMIANEKEVLTADNDEGKKKVLTENYAFLMESSSIEYIQERECNLSQIGGLLDQKGYGIAMKKTQVKYYFLY

>NvitIR18

MKQTETALMYDAVHLFAHALHVLDASQQIDIKPLSCELTNTWDHGYSLINYMKNVEMTGLTGSIKFDNQGFRSDFILDIIELNTKDGLQKIGRWNSTRGINFTRSYGEIYTQIVDNLHNKTFIVTTILIPEINKIETLKGQLKNEKRLKNQAVHLNN

>NvitIR19

MCLISAESASQDAMQALATPSIRMVAGMWWFFVLIMVSSYTANLAAFLTAVKMEDSINDVEDLAKQTKISYGALRDGSTYSFFKNSNTSLYQRIFNTMSDAKPSVFTQTNDEGVDRVIKGKRKYAFFMESTSIEYQIERHCELQMVGSLLDNKGYGIAMPPNSPYRTMISTAILHLQEKGELQQLKQKWWKEMGGGKCNEETDEPTNSNELGMSHVAGVFIVLSFGCVISILIAIIEFVWNIRKIVIEEKVLGILPKSAQQHKIEVTVVTNGGAGNIMQDYVAVNRTAFSVREEAQKRNLNMQLVATHVQSTDN

>NvitIR20

MTINYARETVIDFTKPFMNLGISILFKAPKPQPTELFSFMNPLALEIWLYILFAYVLVSITIWIIARFSPYEWAYPNPCQDRNRLVFQNDFTLPNSFWFAIGTLMQQGSDVNPKAASTRIVGGIWWFFTLIMISSYTANLAAFLTVERMATPIESAEDLAKQSNIGYGTLAGGSTMTFFRDSKVGNYQKMWRYMESKGENESFVTSYESGVEKVLSGNYAFLGESTMLDYLVQRNCNLTQIGDLIDSKGYGIATPKGSKWRDKISQAILFLQEKGVIQMYYNKWWRNKSQSTSSSTHCSTKPRVVDSVKANALGIVNIGGIFVVLLCGLAVAVIVAIMEFCWSNSSSEMQLWSSVGVIPALKFLLLSMEKTFLILTSLSSPIVKRNMKLLLNQIAPSVFYSAMEITDPRQLQ

>SinvIR1

MLTRTSFLLILIFFLPFVICQTPMELMVVIEEADSSILSILNDAVPEAERNFANDMITVHVSTVTVNRENVDASFEKVCAVLYKGISIILDMTWTGWDKLRNVADERGIIYKRGDCSISPYVQAIDDLLIMKNATDVGLIFEDERELNQSLYYLIGNSIIRLVVIDELTERTVTKVRSMRPSPSYYAIYASTAKMEELFKTAIGGGLVRRDGIWNLVFTDNNFHEFRYITGDLQLNVSFTILSMKTDICCRLMGDVTCSCPSNFKIFPYYFKRLIGLIVSLMSDVQRSGISVAPKTGQCDKPVSPTSVSNITAETFNKILMTRLDSNDTFEYWSEKAMITYKAEIELQVVNRGYVDVLATWTRRTKIKEADGRKIEPARRFFRIGTAPAVPWSVTKKDPVTGLEIRNEDGKEIWEGYCIDFVQKLSDEMQFDYDLIIPEDQEFGKKLPNGEWTGLIGDLARGETDIAVAALTMTSEREEVIDFVAPYFEQSGILIVMRKPVRKTSLFKFMTVLRLEVWLSIVGALTLTGVMIWILDKYSPYSARNNKRLYPYPCREFTLKESFWFALTSFTPQGGGEAPKALSSRTLVAAYWLFVVLMLATFTANLAAFLTVERMQSPVQSLEQLARQSRINYTVLANSSAHQYFRNMKNAEDKLYTVWKEITLNSTSDQVEYRVWDYPIKEQYGHILQAITQVGPVKTTEEGFKKVIESENAEFAFIHDSSEIKYEVTKNCNLTEVGDVFAEQPYAIAVQQGSHLQEEISRKILDLQKDRYFETLAAKYWNQTLKSQCPNSDDNEGITLESLGGVFIATLFGLALAMITLAGEVLYYRKRGVSQKGKPNDKKTIKDIENDKIIMQKIASKLRMKPAPTDAVFGKQFSQPPRVSHISVYPRHFPKE

>SinvIR2

MVSAKICMLYVLSATFTAYGVEDMSKEKQKPTQSRPANLFIINGVKNEAGNASVTAALKTLAEKYPGHLGNVWTVQINETDSSDSLEAICNTWSSAVSKGGATVPDLILDITTANLGAEASSSFTAAMGVPTLSGQFGQEGDIRYWRDLDLDQKNYLIQVMSPSDLVPEAIRQLAIQMNITNAGILYDKNFVMNHKYKSLLLNVPTRHVINGLQDSIDNTKEQLSKLRDLDVVNYFLLGDGDSINMLLDAGALLSFTGRKYGWFFLTLDEELWPSCECENVTVLFLKPESPMVNSEKQAESVVRASLPKPLITSAFYYDLTLLGVKAIKLALDNGDWPLEPRHIDCNSYDETNTPTRNFDFLAKLKAVSQNTTPTYAGISWGNKNGEHHADFKMFMYMLNIEREKITSKEESGEWQAGIEMPLQVTNEKIMNNTAVTSYRVIAVQHPPFMMYNNKTKEWYGFCIDLLDAIQETVPFEYEVREVEDHEYGSLNENGSWNGMMRELIEKRADIALSSLWVMAEREKVVDFTVPYYDLVGLTIMMQKTKTSTSLFKFLTVLENEVWLCILAAYFFTSFLMWLFDRWSPYSYQNNRDKYKDDEEKREFNLRECFWFCMTSLTPQGGGEAPKNLSGRLVAATWWLFGFIIIASYTANLAAFLTVSRLEIPVETLEDLSKQYKIQYAPIVNSPAYVYFQRMADIETRFYEIWKDMSLNDSLSDVERAKLAVWDYPVSDKYTKMFQAMKEAGFPADIDEALKRIRRADQYANNEFAFIGDASTIKYLAMINCDLIQVGEEFSRKPYAIAVQQGSPLKDQFNNAILILLNKRRLEKLKDKWWKHNPVRKNCDLENSQSDGISIHNIGGVFLVIFVGIIFACLTLAIEYWYYRHRAKVNELHQTTLPKTKLTKMTKPIRYNLQPAPTQGFQTISQARPRF

>SinvIR3

MFNIILMIKNSKHVAADSCKLACELHFYFCYLFSLFACKLDVSQSPQNFYFFHSDMCRNLKLKHSGTYVALFLAFSRFTCNVNAGDSDFIRDYFVNKATRYVVGFSCGDFTSDFYFMKSLSRAGIFTIIRKHDSSINLQRFLSTDAWSLGVVVDLRCRNESAVAIFAETSKYRMYDYSYHWLVLGSKLSMSVPLLNDSAYSMTTNFVLAIMNGSGYDLYDVFNHCKYRGGTLNVTKLGSWRQGEGLIITLTQPLVERRANMHGMTLKMSGVIQYRPKNMRLEDYMHDANTRSLDSMHKFLYAMITHTAELFNFSVHASEIIYWDRHSVHGLIFEILQTNYIDFGSNPRIMVSERLNYASLIGAAWPIRPCFMLLSTTSSNIKLEVFFKPFSIYTWYLSAVFIVLFICVMRIIIRREETREAEETRIEKYSNAVILTIGITAQQGANFFPKRIPGRIAFLQILLFNWIMYNYYSGSIVSARLSEPLDMMEDDVTVLADSDLKIAAEAVPYLNYFLYKLSEESTYFRKKRWDPLPESKRYLPLEEGMKQVSEGTLAYHTDPNTAYPYVEQMFDPSKICALTEIHLFKQSTMGMYASHNGQFTEMAKIGLYKMFNTGLRDRQIKYWSSRKPQCTLDTLSTRSISVYETAPALILLFFGLLVGSMICIAENIIYYRFMREEMLTRMSSNDGDGGGNGGNGDSGDGRDDDGGNGGGGRDNSGGNAEETAGTSKHNLPE

>SinvIR4

MPRASWKLLFLSRLLLVSCGNDTIMARDYFMYKEVSNVVAFSCGGLESDVKFVRTFNDVFVLTSMWSLGSVDASSIRTVRKLLQSNYRNLGVYVDARCSGHNYTIIYSEATKAFLFDETHKWLILGRKLKDILKLLNDDSFGIVTDITIAIETVTGFDLYDVYNPCKARGGTLNVTTLGYWTEKSGVAIRLRQSKYERRSNLHGMKVKIGVLLPRKFYNLSTEAIVLDQDMKAKYGRSRFLYTILLHMADLFNFTMDIVEVDPRKRQDNSAPMFVAFQKKTVDISASPIVMKAERLRSGDIIGPVWPMRSCFLFRTISSASLKTKQFLRPLHIKVWYVVLGTMIGSTAILIILIRQQENVDSLSEACSICILFTIGAVSQQGSVFVPVHYASRIAFIHIMLFSVLILNYYSASIVSDRLKNVGVKMNDSLISLADSYLKVAAEPTPYIRSFLQTPEKEIRYFNAKRWDKLAESKRYLPLAEGLDRMAKGGLAYHTSIESAYPYIENHFTPRMICELTEVHLFRAVLALWGRHRSPFTPLLRIGLTKMYDVGIRRRQLKFWSARKPLCPRNVLVAEPLSIQEATPIFVFIGIAATLSLVICIMENLVFWLGPRLIHSISTFHGRKLCCPTFKRDTPTSPGEIFSRE

>SinvIR5

MCRNLKLKHSGTYVALFLAFSRFTCNVNAGDSDFIRDYFVNKATRYVVGFSCGDFTSDFYFMKSLSRAGIFTIIRKHDSSINLQRFLSTDAWSLGVVVDLRCRNESAVAIFAETSKYRMYDYSYHWLVLGSKLSMSVPLLNDSAYSMTTNFVLAIMNGSGYDLYDVFNHCKYRGGTLNVTKLGSWRQGEGLIITLTQPLVERRANMHGMTLKMSGVIQYRPKNMRLEDYMHDANTRSLDSMHKFLYAMITHTAELFNFSVHASEIIYWDRHSVHGLIFEILQTNYIDFGSNPRIMVSERLNYASLIGAAWPIRPCFMLLSTTSSNIKLEVFFKPFSIYTWYLSAVFIVLFICVMRIIIRREETREAEETRIEKYSNAVILTIGITAQQGANFFPKRIPGRIAFLQILLFNWIMYNYYSGSIVSARLSEPLDMMEDDVTVLADSDLKIAAEAVPYLNYFLYKLSEESTYFRKKRWDPLPESKRYLPLEEGMKQVSEGTLAYHTDPNTAYPYVEQMFDPSKICALTEIHLFKQSTMGMYASHNGQFTEMAKIGLYKMFNTGLRDRQIKYWSSRKPQCTLDTLSTRSISVYETAPALILLFFGLLVGSMICIAENIIYYRFMREEMLTRMSSNDGDGGGNGGNGDSGDGRDDDGGNGGGGRDNSGGNAEETAGTSKHNLPE

>SinvIR6

MRPMYLCIFLLQAFIVVANAQTSQIIRDYFVYKNVTRVAGFSCGDVDEDLLTLKLLNNVGISTAIKPAGLQQIDIPRYLDTDRTMGVFVDVRCPNQNYSGIFDEGSAHRMYDYSYSWLMFGNNLSHSLESLNDSSFSIVTDFVILLSNETDYVLYDVYNLCKIRGGVLNVTWLGSWQEDDGLAINLTDSRINRRRNYNRLRAKAAGIVLNRPEHVSLIDYLEGDSLEVMDNWPKFGHTILSHVANMYNFTMDLIEINHWEKNDSNGPVMGTLKRGEADLGYYPSILSIERYADARVIFPQWPSRTCFMFRTIPAMKMKPWIILKPFAADTWYMIVVMMVITIIILSCILKLERADDYGCSISALITVAALCQQGFPSLTDQSASRIAFIQITVFGLLVYNYYSAAIVSARLNEPLQKMNDSLYSLAHSKMQMAAEKNIFFNFLLRNKRPDVQYFKYFWDAVPEEKKFITLEDGVQAIKKGGFAYHADPDDIYPLIEQEFDKQMICQLTEVHLLQPSELGLWSNHKSHFHEICKRAFLKISTSGIRRREIRRRSARRPYCPSDEIFVSSVTIYEAAPILFLLLFGMIFSFAVLGIEHIIFYMINSKRISVITPDTKLNTKQHIKSDVKKGIKINKKNKVSLKDPPGNNVILVLPEASRLFEQVLMSKRYQNR

>SinvIR7

MISVFFLAWILNSGDAFGDFPSLISANASMAVIIDKNFFDDKVEYRDVMKNIHGLIASITREEIHTIDIDIQIIRGTKINFRDYTVLLSVTTCHQMWSLHDAARKEELIHLAITDEDCPRLPDTEGVSIPIILPGQELAQIFFDIRSTDALLWNNVNIIHDDTFDRDTIGRVTKALSTALPNKKFNMVSRALFTFKYSDSATTRRYYIKDSLENFHVDQLGRCFLVIVTIDTASDVMEVTKTLNMALPDSQWLYIITDSVVRNSTNITILTDLLSEGSNMAFIYNATDNDTYCNVSLKCHIQELVAAFVNALKISLMTEIELFSHLSDEEFELVRLNKAERRREILKNIRIKLIDENFATGGVCGKCLFWRFASAITWGNFFLHSKNVAHLIESGTWIPGLGLNLTDEIFPHVVHGFRGISLPIATYNNPPWQTISLNNAGEKEYGGLVFDVIKYLGKKLNFTYTVLTPASNRAVKFIRNETADVVKLHIRXLRFSSSTTREMPPQIIDMVLEKKVLLAACAYTVNNFGRGKVNFTLPIFMQTYSFMTAKPGQLSRALLFTAPFAKETWACLASSIIIMGPILYLIRKYSPDNTETSGLNSCWQCMWYVYGALLQQGGMYLPHSDSARLLIAVWWLIVMVVVATYSGSLVAFLTFPNMDAAILTVDDLIAHKNRITWGFPNGSFLEEYLKNSEEEKYHILLERSIIHNETTASKVIEKVKAGKHALIDWRSTLRFSMRSDMLSTDGCAFSLSSEEFMDEPIAMIIAQDSPYMKIINFELHRMHESGLMNKWIAEQIPTKDKCSDSITTQVIEERKVNVADMQGIFFVLFMGVTGSIFLLCCEFCCHKRKMSKRRKLIQPFVS

>TcasIR40a

MRRDHGGDLVSASFDIVAGFLFEEICICFDKNTNINFLQHLLVRFVSNNIAIKLFNITTVEVQDKYFAFLNYQVTNHLGANTIFFSSHKFYEHVLLEINERDFIRRNLIYIFNWGRRPFSRYFVRNIINVMKVFVITNPRNDTFRIFYNQAVPYKKHHLEMVNWWQHGVGLFNHPTLPAKYNNVFKDFKENVFKIPVIHKPPWHFVQYGNDSIKVTGGRDDRILSLLSKKLNFRYDYFDPPERIQGSSASENGTFKGVLGLIWKRQAEFFIGDVALSHERANYVEFSFITLADSGAFITHAPSKLNEALALLRPFQWQVWPAIGVTFVVVGPVLYAIIALPNAWRPRFRVRSHARLFFDCTWFTTTVLLKQTGKEPSSSHKARFFIIILSISSTYVINDMYSANLTSLLAKPGREKAINNLNQLEKAMATRGYDLYVERHSSSYSLFENGTGIYSRLWQMMNRRQTHFLLESVEEGVQLVRDSTNKAVIAGRETLFFDIQRFGASNFHLSEKLNTAYSAIALQLGCPYIEEINKILMAIFEAGIITKMTENEYEQLGKKKQTTSETEKELIPGVKKENRRVAKVSEDNEKLQPISIKMLQGTFYLLCIGNIFSGFILLAEILVYKHRKTYKHKKRRHRFVYLRKIRHSVASKFGAVVDAVRRVYRRAMHDAFVATLEYLE-

>TcasIR21a

MQRGLIVLKLCLTALALKSLDKRALQKSHEKSQLEKWEDKFLNRDPSFDQTASLVNLISKVALDELSGCSATILYDKFTETSSDLLLEKLFRTFPIPYLHGQITDKYHMKVPKLQTSQDTCTGYILFLKDVMRSKDVVGPQTNNKVVLVSRSSQWRVYEFLASEQSQSFMNLLVIAKSEKIVSSSIARLICLALHLKFGTALAIYAPNGGKSAVYPSVIANVPKLGFRSAESVTSVITQNGANLGIGGLYITDTRLKATDMSHIHSQDCAAFISLASTALPRYRAIMGPFHWTVWLSLTLVYLFAIFPLAFSDKHTLRHLLDKPEEVENMFWYVFGTFTNAFSFFGKDSWSKTDKFATRLLIGFYWIFTIIVTACYTGSIIAFVTLPVFPATVDTPEQLVRGKYTVGTLDKGGWQYWFENSTDPITQKLLTRIDFVPDIESGLKNTTKAFFWPYAFLGSRAQLDYIVRTNFTTINKRSLLHISSECFVPFGVSIIYNKNALYSKIIDQGVLQAVQSGIVDKIKNDVEWETMRSASGKLLAANSYGKSLKALTVDDRALTLDDTQGMFLLLGIGFLLGGASLLSEWMGGCLHLCKGNRNQSATSIQSNYRSHEVPTPREKLDSMQFNSFENHKIEEEIVEERNCIIHRQDDDDIEEHINRLFDFEGVFGEANPDSRTGPEEELSFKNTTKAFFSLYAFLDSRAQLDYIVRTYFTSMNKRSLLHISSECFVPFGVSIIYNKNALYSKIIDQGVLQAVQSGIVDKIKNDVEWETMRSASGKLLAANSYGKSLKALTVDDRALTLDDTQGMFLLLGIGFLLGGASLLSEWMGGCLHLCKGKRNQSATSIQSNYRSHEVPTPREKLDSMQFNSFENHKIEEEIVEERNCIIHRQDDDDIEEHINRLFDFEGVFGEANPDSRTGPEEELSEENGKK-

>TcasIR76b

MGLFEIALAALCLNATCPGEEEPPEFPEVQYLAPDSNDRKTLFAQLTEQLKNENLIITTLKNDRLSGTEKRNNTILGKGIAFDLLNILQDKFQFNYTLIEPKANVWGAEKFGVLDLLKDKKANLSAAFLPVLTQYSNHISYSPSLDTGEWVVLMKRPKESATGSGLLAPFNLPVWLLILLSLVVVGPVIYFIIYLQAKLCKDDNNKVFPLPACIWFVYGALLKQGTTLNPMTDSSRLLFATWWIFITILTAFYTANLTAFLTLSKFTLPITEPKDIGEKRYKWVTTKGNALEDTVTVNESLTELGKILGQPQRYLYVSDSDILRNYVHKRNWMFIREKPIVEYVMYDDYKEKTRNQIEEAKRCTYVITKFSVVSFSRAFAYSKDFKYKPLFDSTLVQIVKCHKCFSLLSRIQYLVESGIIKFKLREELPDTEICPHNLGNKERQLRNSDLLMTYEIVGGGFIISAIVFIIEVIIRRQKKPKTKSLPLQNPNKHTFEINLNNNYEKFGHFPYSSKFVTPPPPYHTLFNPPHKSDNMKKRNFNGREYWVYDSISGETKMIPMRTPSALLFQYTN-

>TcasIR93a

MLLELVLSSAFVCVIRGDSFPSLLTTNATLAVIIDREFLSNEYEVIKHAIESYLVFAKREILKHGGVNVQYYSWTTINIKKDVTAIFSIASCPDTWRLFRQARDANLLHMAISESDCPRLPPDEAITVPLITRGEELPQLLLDLRTRQTYNWNSAFILYDDTLSRDQVTRVVKSITAQYSNLRVNAAAISFVKLETRLPMDEIRRQVKEILSSVSIKTVGGNFLAIIGYELVELLMEYAKMFGLVNTRTQWLYIISNTHFRHKDINRFRQLLSEGDNIAFLYNNTVNNDTCTGGIQCHCEEILSGFTRALDEAILFEWETSSQVSDEEWEAIRPSKLDRRNSLLQGIKTFLLQRGQCDNCTSWLMKTGDTWGREYQQNGTDSGGLISVGNWRPSDGPSMSDELFPHIVHGFRKRNLPIVTFHNPPWQIIRSNESGAVSEYAGVIFELIKELSKNLNFTYTVELAKIGQEFSANLTKNEAQVVTNFIPDSILDMIRNKSVAFGACAFTVTEESKRLINFTSPISTQTYTFLVSRPRELSRALLFMSPFTGDTWLCLSASIVSMGPILYYIHKYSPVYEYKGLSKRGLSSVQNCIWYMYGALLQQGGMHLPQADSARIIVGAWWLVVLVLATTYCGNLVAFLTFPKIDIPITTIDELLAHSGTVTWSMPKGSYLERTLKYTTEPRFRYLFDKKVEVGNFKNMIEDIENGKHVHIDWKIKLQYIMKQQYLDSDRCDLALGLDEFLNEQLAMVVSQDTPYLEIINDEIKKLHQVGLIQKWLTDYLPKKDRCWKNNRHIVEVNNHTVNMDDMQGSFFVLFLGFLLSFFITIGEKLWHKYVTKKKMKIIQPFTT-

>TcasIR64a.1

NKISLILVILSKTETYIIKSCLSNAIVDFAILANVAFSLRISCYKLFMHIKLIANVFYNQLDQVLNRNHYHLAVIIDSGCIDYADFAIQDKKYFYETYHWLVPTTPQNLNNSLNFLQKSPLNINSDVNVAILNGEGTKWSILDVYNPASSHHGQFTVTKLGLCDETNGYQAKIAGNKYWSRKNMTGVQFKSAVVVPDPSIKLNDYLTSDKNRQLHSMHRFQSVTVNYCREMYNFSLEIQRTNSWGYLTPNGHFDGLVGLLERRLVDFGSSPLIYKLDRMPVIDYSYGNWVLRSTFIYRRPKIIEASYKIFLRPLSRTVWICIVLMMVLLMLFLKVVFSREKRLLQKRNLVDSSWSFLFLFTLGAFCQQGATCHPQLLSSRTLSIFVFLFCILTYQFYSASIVSYLLIDPPRKINNLKDLSDSNLRAGIEDILIDRNYFVQTTDPVAIELFNKKIKFSNNNSGFYEPWDGLDLVKQGGFAFHVETSTAYPIIEETFTNEEICELEEVQMYRTQPMHTNLQKNSPFREMMNYCMLHLVENGLMYRLRKYWDARKPMCIESAKKFTFNVGLKEFSSGLIVLSYGILISLGLLLREVIVHKK-

>TcasIR64a.2

MSPPLPFMILLSVLTQTHALLDINLIENYFTEKSIKSATVFGCFRKTEQLNLVKIFSRGSSPISVLNLNQAGVYQSIKSNHQQIGVVLDGDCPESESFLITVSPGFTHIAPNVVFISVRSTETXFDVKHHWLILSKSIQFLEKIKNAVVNINADIHVAVQSGTNWTIFDVYNPASEHGGSLKYTRVGFYSRGRGYNAQTNEAKYWRRKDMTGVTFKTMVVLLVPFEGPLEDYLHNDDNRNINTFNRFQNKLLRFCRDYYNYSMIVELGSSWGYPFPNGSFDGMVGAMEKKLIDFGSSPIFVREDRARVIDYGRNTWSWKAGFLFRSPKSRTSIEIFLKPLSTSIWLITGVLATASIVILKMVTTFERNRYHSTSETSWSLSFLFTLGALCQQGSPWVPKMACGRITAISIFLLSLIIYQFYSASIVSHLLMKPTNKIRNLKDLTDSSLKVGCEDIIYNKDLFAHTTDKVLKDLYAKKIYGKGNTSHFFPPEKGLDLVRQGGYAFHIEVARAYPIIETTFPDNAICELREVKLFKNTDLYNTMQKGTPFRDMLESCFQRLAEQGILDREKKHWHPRKPECIQSSQAFVTFHVGLDEFYPALLVLLIGIVISLTVLVVEKQIHIAREKMEREKGVVF-

>TcasIR64a.3

FQLRVLMERLFFLSVLAVIIYTTNCTDNHDIITSYIKEKSVKYATVFGCFTKKEKINLVKIISHICPISVFDINRLNIENRMESRHFHTGIILDGDCPSAEKFLINCGRSYLFDVKHHWLIVASSEKIREKFNNVILNINADINVIIPEKPSNWSIIDVYNPASQHGGVLNFTRVGFYNKHDGYKIKYTGVKYWNRKNLTGVTFKSMVVVTYSKTXKNSAYTIFQLPVPFEGTLQHYLDSDDNRDVNTFNRFHSRLISFCRDYYNFSLDIEVSKSWGYTNEDGTFDGMVGALERKIIDFGSSPLFLREDRARVIDYGRNTWILRSLIKQQFRIISNWGFSAAFIFRNPKVRTSLEIFLRPLPSSVWLITGLLAIVSIIILKLATSFERRRYVYDVETSWSISVIFTLGAFCQQGSPSTPKMACGRIATFFIFLLSVLIYQFYSASLVSHLLNKPLTKIKNVRDLLLSPLKAGCEDILYDRDYFLHTTDKVAKELYAKKILGKSNSSNFHTPEAGLKLVAEGGYAFHVETATAYPIIESTFQDQAVCELREVPLFRTQPMHANFQKKSPFRDMFDTCFQRLAEHGLLVRERKHWHPRKPECIQSSKSIRFNVGLDDFYPALVILLVGIVASLLILVIEKEFRILTENPA-

>TcasIR75q.1

SFLGTILTVYKQLAEKKIVLNVLTNHWKINQTKLSQHTFLVGDTLCPQFNSLLSHVSKFFCYQNSQQTLGQIITSSXKWLVFDQNSTVNTNDLLLDSNFAVASQISNGRFHLKLCYKRAPNETIKFNEIGVFSNGFEYYNHFIPTRNRSDLSGVNITVSYVVTKPDYPFDVEDYRFRHLEAFSKLSYAMVYPMLEMLNCTKKFIQRSSWGYKGANETQFVGGMFGDIQNGTAEIGGTVSFYTVDRMSVVDYLSVTTPSDLKFILRAPPLSYVNNLFTLPFDTKVWYCLYFIVGVTVLILYVIVRCESTYENALERRNNIDNIKPKFFDVVMLQIEAITQQGSENEPKTMSGRIAVFIVFLVLMFLYTSYSANIVVLLQSTSANINTLQDLLNSKITLGVEDVVYSHHYFETQTEFTRKSIYEKKVAPKNQKSNFMTTEMGIEKMKDEFFAFHVETTAGYKQIMDTFQEHEKCGLIEIDYLNVLYPSITIRKNSPYKEIVKVNFRKIYESGIRHRQLNRIYYKKPHCVGKGGSFKSVGIVDIYFSVEIFAIGCFMALWLLLLEVLFKKKIKFLVQ-

>TcasIR75q.2

MKILIVFICLLINETTQNNFTDNLIVNTFNFIKILNVPVKISAHICWTRGKFDSLLMKLYXTVLANTIHFIKSISDKYNTNLIKNVSPKYANPEHQLFIIDLKCNDSLSVLQQAEKFKLFKSPFKWLLLGNSESLPNLYFGTDSQIFVTEPRSQLDDIKTIYKYSPMVPRFVQHSFDRFYTNTKRTNLMGTTIKISYVITNLDSLNHLWDYRLQELKKKLYHFLICRNSHIDAINKLNYILVHNLMDFLNASRQFTMQPTWGYKNSTTGLYSGMAGDLQKGLADLGGTPLFFTPDRIDIIDYIAATTPTYMKFIFRAPPLSYVTNVFTLPFDSAVWHYCFVMVAVVVVCIYVIVVWEWKETKFEEKDTHSHIDTLRPNIFDVVMFEIGAITQQGTNAEPKSNSGRIITIFSFLTLMFLYTSYSANIVALLQSTSDSIKNLEDLLNSRIKLGVEDIVYAHYYFENAQEPVRKAIYQQKVAPKGQKPNFMTAEEGIRKVQQGFFAFHVELSTGYKIIGEVFQEGEKCGLKEIEYVNLIEPWLATQKKSPYKEVMKIGMRKMHETGVQNREIRKIYTRKPQCHSGGSNFGSVGLIDCYSAFLTFGVGIAFAFLLFVMELIVRRYFIRREKERLK-

>TcasIR75s

IVLPMINDLIEHFNKTQIILAYLCDKNGTNLLLIRNNNNTNFRRLSGSEPLFXKKLYQVNVLSPNSRDMPYPTPPAFLTYVLDAGCSNTKQLLLLVPVITHXLIFGNNILKASEQKQFATPFKWIVYYNNPVELSFFIDEYFTKTNILVDSDVTLATINPTSGTFDLNKIYKRKINGSIIIENIGIWGRGLGVTDTGYEKITYKRRRNLTKTVLKSCIVITNNDSLNHLTDKRDIHIDSIAKVNYVLVQHLSDTINASLEYSVRGTWGYKDNKSQWSGMIGELTRNEADIGGTALFLTSDRIRVIDYIAMTTPTRSKFIFRQPKLSYVANVFTLPFDASVWASVCGLLVIIAGLLYVVVRWEWKKKDYVQVVVFFAFWVDFPSSVFCRTNRTSRKFTILGSXVFITFGALCQQGSSSVPFSIPGRITLIFLLVSLMFLYTSYSANIVALLQSSSSSIQTLQDILNSRLDVGVDNTVFNHFYFPNATEPIRRAIYQQKVAPPGQKPKFYPIEEGIRKMRQGLFAFHVETGPGYKFVSEIFREDEKCGLQEIQYLQVPDPWLAIQKNSSYKKMLKVGLRLLQENGIQEREVGLIYTKKPQCLARGSSFISVGLVDCYPAAVVLAGGIGAALAVLILEIYVHQRFVGFLL-

>TcasIR8a

MVISENLDKTTANRLKAIRPIPNNFAIVATSSNMEELLQTALDENLVTLPERWNLVFLDFQYQQFDKKRLKNMPINLLHMDEEICCRFLQSEKCECPHDFNLQENFLSLATNTLAKILKTLTMENLLRADLNCDDSRYSEATRTRFYELLQQEVDSNDLVFKENFGLHVNINGVIETGDEKVAEYNYKTGVTVLDGKKVEPITPFFRIGITHALPWSYKETDSSGNTYWTGYCVDFTEELSKLMGFGYEFVEPKSGTFGKKRDGVWDGVVGDLATGETDLAITALIMTADREEVIDYVAPYFEQTGITIVMRKPVRKTSLFKFMTVLKLEVWLSIVGALIVTGFMVWFLDKYSPYSARNNKKAYPYPTREFTLKESFWFALTSFTPQGGGEAPKALSGRTLVAAYWLFVVLMLATFTANLAAFLTVERMQTPVQSLEQLAKQSRINYTVVKDSDTHKYFINMKHAEDTLYRMWKELTLNASTDDTQYRVWDYPIREQYGHILLAINDSNPVANASEGFRIVNEHTDADFAFIHDSSEIKYEISKNCNLTEVGEVFAERPYAVAVQQGSHLQDEISKTILNLQKDRFFEQLQAKYWNHSGKGSCPTTDDNEGITLESLGGVFIATLFGLALAMITLVGEVLYYRRKSKIQNSETKKPKTVQTSENWKTDTLMPVSLINKDKQSVTIGTEFKPVNRNRDLSEFGHITLYPRARNRITQTSNE-

>TcasIR25a

MASSSAIIYRIAIYSRIATAHLNYSDFLNNVLTETHKMLKLVAFILYCTNLANGQTTQNINVLFVNEEGNLVAEKAVDVATNYIKKNNKLGVNADPVKVVGNRTDASGLLDSLCSSYNEMIANSMNPHLVLDTTMTGLASETVKSFTAALGLPTISASFGQEGDLRQWRNIDENEKEYLVQISPPADVIPEIIRSLVLSKNVTNAAILFDDSFVMDHKYKSLLQNVATRHVIAPIKEADKIGDQLRQLRKLDIVNFFILGSFENIKRVLDAADSVGFFNRKFSWHAITQDKGELKCNCRNATITLAKPLIDAQYQDRLGLIKTSYQLNAEPEIAAAFYFDLALYSFLAVKEMIADGVWKRNNATNYITCDDFDGKNTPRRAGLNLKKYFSKEVSETPTYGPISIVSNGYSFMEFTMQISAVGVRESSSDKSVPLGSWKAGYDNNLTLVDPQIMKNYTADVVYRVVTVEQKPFIIKDETAPKGYKGYCIDLIQRISEILNFDYEITPVGDQKFGNMDENGKWNGVVRELMEKRADIGLGSMSVMAERENVIDFTVPYYDLVGITILMKLPKTPTSLFKFLTVLENEVWLCILAAYFFTSFLMWVFDRWSPYSYQNNREKYKDDEEKREFNLKECLWFCMTSLTPQGGGEAPKNLSGRLVAATWWLFGFIIIASYTANLAAFLTVSRLDTPIESLDDLSKQYKIQYAPLNGSSTMTYFERMANIEAKFYEIWKDMSLNDSLSEVERAKLAVWDYPVSDKYTKMWQAMKEAGLPNTLDEAVKRVKDSRSSSEGFAYLGDATDIRYLEITSCDLQMVGEEFSRKPYAIAVQQGSPLKDQFNTAILQLLNRRELERLKEKWWSKNPEAKKCDKQEDQSDGISIQNIGGVFIVIFVGIGLACITLAFEYWWYKYRKGGKVVDVQAKHSDVATKINDGFHAKINKLYPRSRF-

>TcasIR144

MQVSKILLLSSLLLNRDETSKCLDAIFKQPVVVLRGVPKNLQNFDAWKPETYLILAPNATVLEQMLEKWSTIESFNPRAKFWLLTHWHEIKPKTLTILAKFYIVNVAIVTRTGQVFTYYPYKYENIAQPDTKPVLLGQCDNVPSFPDKLPKFWRNTTVQVLTKCLLPYVDCSDLDQGLETQIFDLVQEFLKFKVRRIFDKSFKFGLAKINGSYSASFRFLQEREVDMAMGSFRSVGSTQFRDFEFSTNHMEDKLVWVVPKARPMVHWVRLVKIFEPSFWGLLVVLTVAMARVFEKMARFTDEPMGIYRKSGFRVAVLILIGSYLKKTPKRFEMRIIFIFWIYFCMVLNIVFNSNLTNVFFGTFNTFQVNSFDDIIKSNLEMGLTDDVMHILSQEQNWPEITSTKVISSCAFGPACLNRTIFQRNLVCCWGERSIKFRMAKFYTTQVHYVDDHLLFFYLLFYFVKGYPIVPQISKMIVQLKSAGFVQFIKSKVDKLEPRQGNELTTKILTLKRLEGPFYFLLVGWVGGIMIFGYEVVTYERKRRKKVRQEVTKILKKKKMRQNEKVKILEI-

>TcasIR41a.1

TKMLFNNFCINILVNFIINNYHKNSRCLLIFTDGDFDYKGEIPTVRIKATNGSFNSYLIFNYHGCQSVIIYTSNVTALLIKFETEIRLKMERFNERKFLIVPQNPSEDFDKFFNLKQLYFISDLLLVLPTHNDTIFDLKTHKYVGVIDNNEPVLLDRWFSQNQSFLFGKNLYPNKLQNQLGRPLKMATFTYEPYSIIGNVFEQFFENDFILQGKSVGEHHGSELMSAVQFALKYNMTPVPVINEKDYWGDIFPNWSGNGLLGNLVDDKADVGFSALYTWEFCYHFLELSKPLVRTGITCLVPAPKLSERWLTPLFSYSSYLWFCIILTLVIAIFVLSLVLFCYNHNKTLNLNYPLKRKTTYIHFLESAVTIVLKPVFQQSLTLRELPIEIASKLLMGLVLLLALFLTSSYGSGLATVMTIPTYENAINTVEDFANSGLDWGATQDAWIMSIQNAEEQRYVKIVSKFHPISEEELFQFSKSGKFGFSIERLPFEDYAIGDYIKEDVIDNFHLMKEDLYWEQCVIMLRKNSVLLPALDLFILKIFEAGLISHWQNEAVDLYMNPKVQRAVKFYRQGQEHTVVKLQWSHVKGPFALLLIGLCISFIIFILELTLKKKRNQF-

>TcasIR41a.2

TLGCLTMTNLNVLLQILLKTYFLNTRCIFLFTDSTIDLQVETPIVYFKVSNTLNPSLIFQHHGCQNILIHHENASDIFVQFENLIRLNNERFNERKYIVTGHNSLKILLTKQLEYVSDLLLVVPKQTGHYELITHVYRHQNRSKINEPVLLDVWYSQNHSFRQENDLFPNKLTNQNQRVLKIGTLSYEPYSVIGKLTVNXSPYYLNLGKDDYSFDGTETSLVYEFVHKYNLTPSFTIMGDDLWGDVYANWTGIGLFGSVLNDEIDIGYAAVYTWEEYYKFMDYTKTLIRSGVTCLVPAPQLAAGWVTPLRSFSLGMWIALVIVLLSNTIVLNLLFYRNQKYHXNQLFQILLFNAFSKRFFIDSLTTAIKLYVQQPLTLTLKRGLLKYFIVTNMIMVLFISSSYSSGLSSVMTVPRYGKSIQTVKDLASSHLNWTGTTDAWIFSLRQVEEANYENIKNRFVVKTQNDLVTASKQYNFGFSVERLPYGHYAVGPYIQRDVICNYRIMQEDLYWGQCTFLLRKNSVLLPLLDKLILRVFEAGLEAYWENQVKCFGRKNMNLRDFLGCLPIHGHVCPKRHYVLYTTYXEHDTIKLTWEHVEGAFAVLVLGYAASIFTFVIELILDKVRS-

>TcasIR68a

MIKNLLPYKCVVLISDDIYGGTFTKSWYRRFGPFITFVVIRVDEYEDLLSPFEETQACLDTAKNEGCQMYLILLSNALQVSRLLRFGDKYRVINTRAKFVLLYDNRLFDKPLFYLWKRIINVIFIRRYSGQKSDTKKNMPWYEITTVPFPTQITSILIPRRLDIWTKSKFRKGIDLFRDKTSDLRNQTLKVAAFSHIPGTTKSLQEKTARTVIGNFSGTEVEILQTVSAAMNFHCELYEPVNVDVDLWGGKQSSGKYTGLVGEMVSTNADIALGDLYYTPYILDLMDLSIPYNTECLTFLTPESLTDNSWKTLILPFKYFRPAMWAAVLVCLLICGAVFHALARFHETISQNKSQVLEIHTKRKKIIILSICPEIEKLDSNLKYTKMREQYKPPRFEGQSIGLYQFSEPFNSVLYTYSMLLLVSLPKLPTGWSLRMLTGWYWLYCLLLVVAYRASMTAILARPTPRVTIDTLQELVNSRLKCGGWGEINRQFFKSSLDPITKLIGENFELVNDSNEAVDRVAQGVFAFYENSYYLKEALVKRQLRFQIARTTQNQSEREMRDIAREDRNLHIMTDCVIKMPISIGLQKNSPIKPRVDKYIRRVLEAGLIKKWLQDVMASILNAEVQSTQEEMKAIMNMKKFFGAIVALFIGYFISVVVLIVENVYFHFFVKRNPHYNKYTRSIHHVKKAE-

>TcasIR100l

MPRKLFLWIFFLLVSCYGNLSETHLQFLKRYFVSANSVAISMLQTHHQEVKIRDLAEVISRKLNSIGTPVVVHENHKSGSLNIIMIVWSLKILRQFLDSLVVPEEKGTYYIIILEQDCATVHSDFAQILEQFWCEHNVLNVVVQNPCSGGTFYLFLPFEHRDNFWGSCKSWDFNEQMPNKLRNLNQFPLKISLFLYNPTLIAKLPKGLKTNPRYHNLSASKGYGGLDGFLLRELVDYFNFDPVIVENLEEYGRVLPNGTAFGSLGDVVNQRVHFSINSRFLMDYGTKEIEYTFPYISDEICMLVPKSLKVPTWKTLLKCFNTLSWVLIFVSCLCSTFAWYFVGPSKNLHKLIWQIYCFIVGIPQKIEPSFSQFVFLLSCFFFNVTIFGIIQGSYFTEFATTSFYPDIDTLEELYESNLPVATHFWFLLDGDTSDLMTKLKTHKIEATGDCLEQTARQRNIATLGRKSESDLIIRTKYTSRDGTPLVHIVEECHTSLYLCGIVPKGSHFLAPFNQIITRLFEGGFTTKWYRDVFDGIISEEKPQLDETVSFNSLNMNDLQTAFHILTIGHLFSIMVLIGEVVIKGKHNKKLLT-

>TcasIR100

KVTIIILIMMCLSLPKIQTCPIKINHLKEHFKQVKSARIMILQNEIIVTDWLIMELIKDNKITVTVQKAIRNFEPFNTSNLTRFEALEFNDTIPTLQTDSTCGHLIIVKNEERLYQYLKSDPGFLILNPRHFYAIVAMELFKTNVLREFWSLQVSNILLLDCDTSYTVLPFNGTTIRINAYTQRKLLRNFHNYFLQVSMQPKPPTAIVKFPKPLRENPIYKDLVPFKDYAGLDGCLLKVLTQRLNMKYVIVGNGQKYGTVLKNGTTTGTLAWIASNKVQISTNGRFLMTYGTNKLEFTVPYSSDQVCAVVPKALKIPKIIMLAKSLTPSSWFMIFLIYVICVLIYTLMGSTGSTWTLYAIFHGFPVKIVPTSRQSFFLTSCMLFSIIIMTIIEGSFFKTFTTTTYYKDINTLEELDESELPIAETFFSFTNDKSRIMTSLKRKKLVINRDDILEQVARKRNIAKLERKRDIKVRLKTEFLDEEGESRLHVVEECFTTFYIGFIVPKNSIFLPTFNNVIRRIFESGLTQKWYGDVEFSIFLEKIFKLENNIKHHSFSFDNIVSALCVLFIGLSLALLVFFWEVTKXKQITLIYVSLIYCIISRH-

>TcasIR100j

LTLVQVVICLLEVSHYDNEKFVNVYQHFTLVRYLTLTFLNDGVHRIDLNNLVVDLMSRLNFSMMIKEKRLGKNSTTFQESDPFQGHIMVVYDVKVLLAFLEESTEVVPKARGSFAILFTSLKCPTHYETNHALKQLWTNHGTANLIAFCDNIYVYHPFSKNDSTWGATLDYSPATETPNLFRNFNGYLLRVSLFKRPPTALKQVPSYISNNPIYRDLKPGDFAGLDGTLLRFLSNYLNFTVVIDESHPTHGRVLKNGTITGSLSDVVSHRVDFSANDWFLIDYQTPEIEPTVPFSYDQVCPVVSKALKVPQWKAFFFIFDLTSWVLIFFMWLCCVFVWHVLNPFRDLSTIIWEICSVLFGNPVNVVPLSNQHMFLGSCMVLNIIIMGIIQGSVFTDFTTTTFHKDINTLEELDEAGLKIASSAWYLDFDTTDLIKRLKTKQIRNYIGSYKDTAFKRGMAVLGRKQDVEHMVKVEFVAEDGSPLLHVTSECLQTFLLVSLFPKGSPFLPTFNNVITRLFEAGLTVKWYQDVTSTGTMLQQMKNFANRRPTGLFSLNDAKLAFYALFVGYIASFVTFLTEILTKNHHNNVHNHVDVLKAQHHGQVQVDQ-

>TcasIR100n

DTFWIVYQTHFLLTDYLTLHILETEDHKFELRQFTQNILKRVNKYGYFLSVRITKSSLNKRNKSYHFPSTAYAPSQNLAKLSDDQEFYKAKRLSTDSKHGFALIVWDLTTLHLFLDQDYRTIVPEGRGTYAIQVVSKQCDVKNEIAFTLQRLWTEYQVINVVAQTPCSCDKTHIFIYHPFVKREGFWGLATSHTLDQIKGDSRLISNTLSDFNGFPLRISIFPRTPTAMQTLPKLLHYNPIYRNLTWSKGFAGLDGLVLATLAEYFNFEVVLVGSLLEDDFGKVLPNGTTVGSLADITERRAVYNANERLVAYFNLDQIDFTVPYTREDICLVVPKAAKIPKWKILFQSLDPQSWCFTLFAYVSCFMFWYNIGPSRSLPKVSWQMFSFFLGIPTKSFARKLDQVLFLIPCMIFSVVMLGVVQGSFFTKLTLFSFYQDVNTLEEMADLELPIGAFIWNLIRDDSDVIRRLKSKSVKPPDNIFDMIAAHRNIATIETRARAQLLIGSKYVDDDGFPLLHIVNECLTTFLNANIVPKGSALLTVFNAVLGKLFESGLTRKWNNDVVDSLIAEKMISVNRKRVRTKSFSLYDAQGAFFVILVGYACSVFVFLCEIVLKXDKICYLALIINKT-

>TcasIR100e

DDFWRVTKNHFLLVNSLTIQVLQTEEHQYDLNQYTVTLLKRLNSLNLLVALRMQEKFLSGRNFPKHSVTNHTFSTTKPKFDPIGGEELTQLKRLSSDSSKGYFIVIWDVESLHNFLDEDFQVVVPEARAXYMIHFAFTYSTEACKIVKLQVSSVLTRLWIDNNVFNIIAQTSCLCDLEVYVHRPFVKRGGFWGLTNSYQMSEIVENPRIIANPLINFNQFPLKIGIFPRPPTVIETLPKLLTDSPIYKNLSFSKGFAGVDGLVLGTLAECLNFDTTVITSKPNSYGYIYKNGTATGAIADVIDRRMVFSANSRFLLIYNTDQLEFTVPYTAEKMCLAVPKALKVYKWSSMFRCFNKLTWVSIICSFGICTIFWYLLKWQKLVTALATIAQFLLGVPANVRPNVPQMLFLNSCMGFNIVIMGIIQGFLFQSFTTTSFYPDINTIEEMVDSELPLRSSIFYFLRIDNSSLIHKLKSRTMAAPPNVYDLVAFHRNIATTDIKSHVDFMVRSRYLDEDGWPLIHTVDECFETFLIANIVPKGSAFLTVFNNVITKLLEGGLTQKWYEDVINSLILENWINLNRNKSKTHAFSLYDLQVAFYVIIMGCAVAILVFVAEIVHKRRNXNNCCNNHHKNIIFAA-

>TcasIR100f

DDFWVIFSTHFLLATSLTFITVQTNSKQYDLRLLAQAIIQSMDKDQVMTTRHVILHNYAENINFNVVFKTGTKKNARDFVTDLLAKTKKLASDSREGFVIITWNVNVLQKFLAQHISEINPRTRATYLFILISSSDSLRKIKHCLHFLWHKYDILNIVVHVLGCGTTTTLIYRPFCKTKNSWGEITAHQIEEIVQQPLLLTNSLQDLNQYPLQVSLFARDPTALTQLPKLLQNNPIYKNLASFYGLDGSMLSTMAKILNFEVVIVENHDRLPFGRVWPNGTASGTLGDVVNRRVALSSNSRILADYNTQEIEFTVAYNGDSICVAVPKSLKVPKWRVLFECFDAASWLLTLSVFIVCLCFWYCVALKNFARILWDVYSFLMGIPTRIVPSRQYFFLSSCMVFNVIILQLLQGWLFTAFTKTVFYPDLDTLEVLEKTNLPVATNMWFLFKDNSEVIQKLSSRGIGKTPNSLDLVAYSRNICVLDKRQDLELYSQAKFVGPDGLSLLHIVNQCLTSVLLVNIVPKGSPFLPVFNDIMSRLFESGFTKKWYSDVVTSRVTEKMVSLGRKERNFSFKIKDLQAAFYVMMAGCVFSLFVFVGELVTHXVFVMNKSSQSKSHRFLLNCNYGV-

>TcasIR100g

TLFKIAEVTFFMVTMHEEFLSLLFGNYYHTNLYQTVKIQEKFARTNNKTGAWYENVALDQKLDPPIDQNWQRVKLRTSDSFEGFIIIVWDPQTLDQFLNQNFSLVVPRARATYFLLFVFSIYENCKLVNHILKRFWSEFSVLNIIAQTPYCCNKVYIHRPFVKTTNSWGVTQSYTLTEVTQNLALITNPLLDLNQFPLRIALFEKNPTAIRKLPKALQNNPIYRNLSRSKGFAGSDGFLLSAMVEYLNFDPLIDETLEPMNFGHVLPNGTVCGVLAEVVHKRTDYAGNCRLMTYFGTDGYEFTAPYSSEKIAMVVPKAGKVPRWRSLFNCFNALSWSLIFSIAIVSTVFWCFLRRSQHLKRASWEMFAHFVGIPCRVVPSRGQFMFLTACMMFNIIILGIIQGSFFTDFTTTSYYPDLNTLEQVLDSNLPIMAFAWRLLRTNSSPILQKLEQRSIPYEDNVYELVALYRNVAALDRRLDLELEIKTKYSGRDGVSPLHIVDESLVTFLTTSLVPKGSPFLVVFNHVIRSMFEAGLTAKWYDDVVTSLIIEHKHKTPSFGVKYRPFTLQDVQAAFYVIAFGYSCSVFVFWCEIIVKFSGKIKHFHYYFVLI-

>TpreIR1

MIVPGRSAFPSPSLELAIQFIEMLRACAFFFLLVGSEARRDLFLKKDVFRSSLAEQNRTSSLANLLRHLIDAYFDHCTLKVVYDSNYEIIHPVDFHRYFGGLKLSVVQDSVDISRGVGTKNDRSDKCMNYVIFLYNIYAVTHILSQENEAKIVIVSSESPWEVKDFLRNQASRIYKNLLVIAHSTSRKNGYGSYLLYTHKLYAEGSGTTLPILLGSWINNSMTVQGLDLFPEKLGTGFMGHRILVSAVHNPPFAISRNSPGAEDSLWDGLEVNLLRMMANYLNLTMEFSSPRSSDGLSPLESVKQDLLLGTTSSAVGGIYQTAQLHEQFDTSMPFLDDCASFISLASTALPKYRAMLGPFQLTVWLMLCASYLALIVPLSFNSKYTRRQLLRQPSAVNSIFWYIFSTYTNSFTVENPLLDYGIAKNSTTLLLAIYWLFTIIVTACYTGSIVAFITLPVYPEAIETAAELQAYRYRIGTLDHDGWEQWFGAESGHDEPFLERLFRKIEYVPSLMEGVRNASRAYFWPYAFLGSRAALDYLVQTDFALARQSKRSLMHISQECFVRYNVVQLFPRKSLYTRYVDGFVLLAQQSGLVDRMRSQVDWQVQRAAINDNKQIIKKMSRKIMVEDRVLMVEDTQGMFMVLLAGVLAGLLSLGIEAITVRLKKRARVQAVATPSPEPSSLADSCLVHDEYYARLRSRFFRSSI

>TpreIR2

MNFSRTWILIFAIKLCHCEKTNPIAKYEDFRFSFVLMICDFNSDRWRKENDVHKLIRDYSNHSIMAQAVGYEELEATREIVDKLNTRLLMIFEIDEQRLFQRGNYSDDWSVFLESHRNFVLDDIVRISVWDSRNGTVLEDYFERSLRDDRSKVVNVARMNFDFRSENFYYFFENFSGLIQELSTYRYNTVVEYDTRKYNTNWSDLIKDLSKQRMNMALTPITMSMSRINQIDFSLPIIVTQAAFYMQTVDSTVIHWSSYFRVFSRPVWLVNAAMVVLVSCLLMITKKRHHRDWHWSDLWSYNFVNIVGIYAQQGLAERPRNPSSRLLYLTVLWLAMNVHMLYSSVLMSFITVPETGLPFSSYLELKDDRLFKIIVLNGSRQEARFHESGSLLHQFEFKLNKTQPLEILDAFLRTCNRNQTVAFYTLDTYYNALSSKIPCVLKPLYLPIKEYMSIGLSKNEPYREELDNLIITLSSNGFLARLRAKYFFLFKDIHFAKVNRITINSVMPILTIYVNGVMLSLIILTVEIIVWRKRTVSSTSDFQKAVAWKK

>TpreIR3

MENSAELLDYCRSPGENNTLNLTFDTEMLVWCENEATINEWYCRANGFVDVHRVANWSLVSGLVQVEQRSLWQRRRSLDGARIRIATVKDTAYDSLTSLATYDGAAARQQQQQQQSAPTLLGLFGQTLRELSATMNFRVELVASETMYGNYDPVTGGWSGVIGLLAQRRIDLGVAEFGRSTDRLEVVDYSSPLVLTRMNLYFEQPTVSAITWSSYAQVLENKIWYASCTLMIVCSVLISFMMTMFRKEGIFSLIVENLFIIWGIQCQQGLPVFPSERSLKLAFVSLFILFLVINITYSATLTSYLAVNVPVLPFSNEQEFLENQSYNLTLLRGNLYHDFIVRSPDRFTERIRERLKRVEELPYSMIDAVSQVCTEKVVLYTNEIFVDKIGPLIPCQITAIDTGRLDSAAFTMTKGNPYRRIFDYYLENYRSYGILKVLKSRYLPKREAHRSVYKPVRFTSIVPVLTMFLTGTLLSLTLLALELICHRRARKCCRLRRHKLVLS

>TpreIR4

MEAEDNETQFPAHITVTTYSDWPYSSYGKEDGEWVGRGYAFELLKLLQEKLNFTYTIVPPKIDVIGDDNRGMLQQLFRKEVDVAVAFIPALSEFRSWCNFSVPLEEMDTTFLLKRPQTSSTGSDLLAPFSNKVWILILLSVLLVGPTTYIMILLRMQFATDDRAKKFTLFNCLWFVYGALLKQGTTTAPIGDSTRIIFATWWIFITILTSFYTANLTAFLTLSKFTLDINSIDDLIRYGHSWFVIEGKSVQTLMKYDYGDAKILRQYSNKYGFRSYDYNMSYQSTLALTKLGKVFITDRPFAQLAIFEDYRSKTFAGVKEENKCTYVISEANVLPKSRSFAFPADSPIKKHFDYELTNAVEGGLIKHLISEKLPLAKYCPLTLDSKERRLKVSDLWLTYRVVFCGMGVAGIVFFLELLTRIMRKIVKYHENRRKVRQSAAASTNRSKASWDGGGIVNNNNNNNITQSIEKSVWMRTPPPLYQQHLSVDYPKSPMKTYTINGRDYYVMKNDHGDRRLVPTGRTPSAYLFQYMH

>TpreIR5

MASRRLRIVALLAIVLTAASSAASQAPVNLLLVVEAPDAAILQSLNNIVSEAEKAYGANLIALDVQVVQVDRENVDENYEKVCKQLYSGISMILDLTWTGWDKLRELARDFNIIYKRADTTISTYAQAIDDIMMYKNTTDCALIFENEKELNETLYYLIGNSIIRLVVIDYLSSQTVERIGNMRPLPSYYVIYASTKQMEVLFKTAVDGGLVRRDNTWYLVFTDNNWPEFSYFQSNAQLKVAVNVFTMKEDVCCHLIYSSSPCNCPPDFKIFDHYFRRLVNLIVETLSEMQSANQLQEPQTGQCNSRNASSPGNGTNVEFDKRLLSKITKNDTFEYEEQRTMIAYKAEIEIKVLSQGQLVQNGTWNRESRIQPLANRTLQAARRYFRVGTAEAIPWTIKKRDPLSKLPMKNPDGSYVWDGYCIELIQTLANMMDFDYDLVVPDDGEFGQKVNGVWNGLVGDLAKGQTDIAVAALTMTSEREEVIDFVAPYFEQSGILIVMRKPVRKASLFKFMTVLRLEVWLSIVGALTLTGIMIWLLDKYSPYSARNNKHMYPYPCREFTLKESFWFALTSFTPQGGGEAPKALSSRTLVAAYWLFVVLMLATFTANLAAFLTVERMQSPVQSLEQLARQSRINYTVLDNSTIHQYFKNMKSAEEKLYQVWKEITLNSTSDQVEYRVWDYPIKEQYGHILQAITQVGPVKTIEDGFQKVIESENAEFAFIHDSSEIKYEVTQNCNLTEVGEVFAEQPYAIAVQQGSHLQEEISRRILDLQKDRYFEALSSKYWNQSLKSLCPNADDNEGITLESLGGVFIATLFGLSLAMITLAGEVIYYRRRNGANLDDSAAAAQQQQQQQQQQKQITMKEAESSVSAMDQIMIQKLAAKLQLKAAPAPIAFGDEQIAVKPSKPRVSHISVYPRPFPFKE

>TpreIR6

MTSRSGAKSKRPRANPLLVSLLSLLLLLSQQLQQCSADGPGLLDGSVQRTPSSIKIGKSSRSHASGGGQGGSSGPVRGNSSIKIGKGNSLLNTTRMSRPVLTTSMTSTSIPLAYVTSASTTPQAADMSLSSSGVEYTGSNNGTDIKKLKVGLAVPYKSFGSREYTRAVIRAVSAMQKTSRHKNLSLFQHYDIHVKVAMQELTPSPMNILNSLCKDILSNNVSAILYLLNYEQYGRSTASAQYFLQLAGYLGIPVIAWNADNSGLERRSSQSSLHLQLAPSIEHQAAAMMSILERYKWHQFSVVTSQIAGHADFVQAVRERISEMQERFKFTLLQAVLFTGKQDLRVLKKSESRVMLLYSTKDEATAIFRDAQELNITGENYVWIVTQSVIENRQPGYSFPVGSIGVHFDTSSTSIVNNIATAIKAYAYAVEDFMNDPSNAQHSLNTQLSCDGAEGGESRWSIGDYFFKYLKNVSVDSDTPGKPPLEFTQDGAPKSAELKIMNLRPSVSMHLAWEEIGTWKSWERRGLDIKDIVWPGNQHSPPQGVPEKFFVTISFLEEPPYINLAPPDPVSGKCLMERGVHCRVSRDPESVDVAATTESSNGPGSSTTAQPQQNGSTYQCCSGFCIDLLQKFSEDLGFTYELVRVQDGKWGTIENGKWNGLIADLVNRKTEMVLTSLKINSEREAAVDFTVPFMETGTAIVVAKRTGIISPTAFLEPFDMPSWMLVGFVAIHSATLMIFLFEWLSPTSGFRGLDSRSHHHRHHHHHHHHHHHHHHHHQHNHHVAGTTNLSSSQRHRFSLCRVYWLVWAVLFQAAVHVDSPRGFTSRFMTNIWAMFAVVFLAIYTANLAAFMITREEFWDFSGVDDHRLARPMSHKPMIKFGTVPWTHTDSTLSKYFREMYAYMKTYNKNNVAEGVEAVVNGDLDAFIYDGTVLDYLVSQDEDCRLLTVGSWYSMTGYGLAFPRNSKFLKMFNQRLLEYRDNGDLERLRRYWMTGTCRPDKEVQKSSDPLALEQFLSAFLMLMVGILIAAILLFFEYIYFKYVRRHLAKDSRTAKCCALLSVREMFMNPEMEVPN

>TpreIR7

MSIECSLKSAAAWRLLPLLLLLLLISVQVHPSTSYVELPKTVIGGLFDRNGSSMRRSFSLAVRSMNSCLFDRHWCDRIQKCEPRHQDCDQFHFLQISALVLEVDGGVIDAHRKFGELAGHGARDPVVNDYGLAAVFGPHSELSSRYVQGLCELHDLPNIVVRREIEHDPVRSINLYPDLGTLAHVYVEMVKKLNWTSFVVLYERPDNFAAVEKLLKMYGPYDYPVYPFALGGGSNYSLPLLRAKATNCKSIVIDCSYERLAAILAQTQQVGMMTDSYRYFVTSLDLQTLDLGPYRHSGANISGIRLLDPDDPFVRDFVRAHLDELEIAGPEQLRTEDALVFDAVAMFAQAYKHLAYDYEPAELRGNKLPQNYDEPVEWPLGLSLRNYLLYNTINGLTGPVMLDTDGSRRRFELDVLNLQKTGLKKIGKWNPDDGFDELHIWNELKMKHFNILITITPPYAMRVNYSKSLEGNERYEGFVVDIIKELSRIIGFNYTFHVQEDAQNGACNKSQSGVCRCTGMMERIISGEMDMAITDLTITEERELCVDFSTAFWNLGMSILYKKPAQAPATLFSFLSTFDTWVWIYLALAYVLMSLMFFALGRISPAEWTNPHPCVEEPSELHNQFTINNSFWFTSGALMQQGSEIAPIAISTRAMAGFWFFFCLIIISTYTANLTAFLTVESPVRVVRGIEDLYNQSKIKFGAKRGGSTFMYFQSSRNPKHRQLAEIMQSKQWERYMPSSNAEGIKLAQTENYAFIMESSSIEYIQYRMCDLEQAGPLIDQKSYAIAYKENFEYHQQISRTISVLQEKLVIKGLYNKWWKEKGAVCFKSKSTTADPMNWDQLMGVFLVLAVGSFLTLGITLWEFFCGVRKVTKKSVATFKQTVTAEIKNVSGSKAIKPVLNRKPSNESSLTNDFNSINYTP

>TpreIR8

MCRRIVLLFILIFHCLCFVQCKRIIVGYVSEKENSVKNVTAPGSSDPEMENPLERLNWALKSYQVSDNVLDILSTICSALEGGAVTIVGNLKEVNAFYVQNICDSMEIPYVSVNSDTLRDYSNALNLYPDLGVLGQFFNELIDSMEWKTYSILYDCTESLANFKFNQLWEKDEPLVTFRLLSTTQDNRGILRKIKESGEKNFLIICKPEALKSILHQSLEVGILADHYNFIITSLDFQSEDLSEYYFSGVNMTGIRLINVDRLSGTYQVDLAPLENYTTTEYLINDAVLSFANGFRRLKYSTSGLQKSISCNMSNNWEYGISLSNFMKSVQTTGLTGLIQYDGIGIRNNFEVEILTLTRSGLAKVGNWNSSRKIEWIQLNTGNPDAEVSSLQNKTFRVLIAMSPPYGMLRETVSKMSGNDQYEGFGVDIIHEISKMLGFNYTFFVQTDNVYGSLNPATGEWNGMLRKIMDDEADLAITDLTITAERETAVDFTMPFMNLGISILYQKPKAAPPSLMSFLLPFSAKVWLYLIGVYVVMSALFFTTGRMCPDEWNNNNPCIENPESLENQFTVKNSLWFTLGAIMQQGSEIAPIGNSTRMLAGCWYFFCLIMVSSYTANLAAFLTVETVERPIKNADDLANQDVIKYGAKKGGSTLGFFKDSNSSTYAKMYEYMVNNPSVLTSSNDEGKFKVMSDNYAFLMESSSIEYISERECNLTQIGGLLDQKGYGIAMKKNASYRNELSRAVLKLQEMGVLTSLKNKWWKEKGGGGRCSEDGGSGQAEELGLANVGGVFLVLIVGTVMSFFGTIFEFLFSIFFERTDPNISAKKKLQEEVKIIVKFGVMTKATKPEEGEEHEDEEENI

>TpreIR9

MKEKSRLFLACLLVLAFHTHLSPRRSGEEAVAATGALALPPVIKIGAIFTHDQRNSSTELAFKYAVHKINKDRIVLPNTTLMYDIQYVPKDDSFHASKKACQQVKYGVQAIFGPADPVLGQHIHSICDALDIPHLEARLDLDQETREFSINLYPAQSLLNAAFRDVMNYLNWTRVAIVYEDDYGLIKLRDLVKQPKKSPEQEIYLRQADVDTYRAILSEIKAKEIRNLIVDTKPENMHHFLRMILEMQMNDYNYHYFFTTFDIETFDLEDFKYNFVNITAFRLVDADDVGVRGILRDMEKFQSAGNLLNRSHVIQAEPALMYDSVQAFAVGLRTLEHSHSLRPMNISCELEHPWDGGLSLINYINSVEMKGLSGPIEFKEGRRIQFKLDLLKLKQHSLVKVGEWRPGLGINVTDRTAFFEPGLTNVTLVVITILETPYVMMHRDKKHTGNSRFYGFCVDLLEMVAQKLGFAYELELVPDQKYGAPDPVTGEWNGMVRELMRHKADLAVGSMTINYARESVIDFTKPFMNLGISILFKVPTRHQARLFSFMNPLAIEIWLYVLAAYILVSVTMFVVARFSPYEWNNPHPCHSQGPVEIVENQFSLANSFWFTIGTLMQQGSDLNPKATSTRIVSGVWWFFTLIIIASYTANLAAFLTVERMITPIENAEDLAGQTDISYGTLDSGSTMTFFRDSMIETYKKMWRFMENRKPSVFVPTYEEGIKRVLQGNYAFLMESTMLDYIVQRDCNLTQIGGLLDSKGYGIATPMGSPWRDKISLAILELQEKGEIQILYDKWWKSPSDTCMRNDKGKESKANALGVDNIGGIFVVLLCGLTFATLIAIFEFCYNSKRNAPPESEHASPIPGTTSCSGSLQGISQVIDAQQQQQEVAELQQQEQESLCSEMAREFCRAMRCFGGGAGGGSGSSRAARRRKKKQKDSCEKCSTHVPGYPVDTSTPIPINGVRSQRSSLGMEGSPHIHMHRQTPSPDYEN

>TpreIR10

MATRTIPTYSVVTIPKAPFVMQSSVDGRFSGLLIDLLNELARRLRFRYKITVLGENEYGIMNDEGEWDGMIGMLKNGKADIGLAALSIMSERRRVVDFTEAIFPSVGISVLMRQPVVPTTLFRFLTILEVDVWLCIIGAYFLTSLLIWIFDAWSPYSYQNVVKNPKKAGVDEKDIDDECKRIFSLRESLWFCLTSLTPHGGGEAPRNLSGQLVAATWWLFGFIVVASYTANLAAFLTISKFEKVIESFDDLVEQYKFSYSVVENSTTYRYFLRMRDIEMRFYEIWKDTTLNDSLSHYERAQLAVWEYPLSDKFIRIFAALKQHGLLKSFDEAIERLEAPQSKFAFITEATDVKYRALTDCQYKEIGPEFAKKPYAIALQKNSNLTQAFNNAIYELFDNRWMQNVRTKWWEENPERKFCDTDELKGGITIKSIGGLFIFIFIGIGLSLVTLIFEYFYFTQFKRKFKNVLDFSTVAWERVKAMATKVWRKLIS

>TpreIR11

MTVTIIHSENRYELFDVYNPSYRHEGKLNVTRMGEWYFETGLNMFSTQYKYERRRDLQGIRLNFSVVAHHLPENMEFETYLTTPVNVHLDTMHRFHYSYILLLRDYYNFSISLTRSSSWGFLKNGTYDGIIGDMTKGIVDISGTPLRITTARMDVVDFTVQNVVAWPKFFLRHPKKNLLTNQFFKPFTREVWILTTIMIIINWLLLYFAIKVEHRYRKSTFYSISETNTATEIALITTAAVCQQGLGATPNLSSGRIVFTSVFLWALVLYQFYSASIVGSLLAAPSRFITTPEALLESSIEIGVEDIPYNYDFYPNSTDPVISGFYNKILKANERRKKPNFLTAEEGMKLVQRGGFAFQIDDLSGYRMIEDSYQTDEICDLQEIYLFPRVHAATGVAKHSPFKKLVTYGLRHILERGVSNRITNVWKHRRPRCPESHSSLPTPVAIYEFSPALFAFVSGVIIALLIAVFENLIRYSSRQFLFKTNRKIIEIFRVS

>TpreIR12

MSLIISSAVCSLALILTCLRVLVAAYNDFPSLITSNASMAVIVEKSFFKDVEDYRRSMSQMSDLIADVTRMHMKSSGLAYTIFGDTNVNLGRDYIVLLSVASCQSTWQLYKRAQKEKLAYLAITDPDCPRLPEGHGLSMPLLRPGDELSQIFFDLRMTKTISWPRINFIYDDTFERDMISRVVKALSIELPNRDLTLSARALFSTKYERDEAAMLKLLHETLANFRLDELGSCFMVIVSVDMIEAVLRVARQLRMVNPESQWLYVVTDASGREAKVSSFVDLLAEGDNVAFVHNTTQMSRDCNMGLTCHLSELVRALALSLEQSLHAELELYERVTEEEFEVVRLSKAERQREIISNVNRLLRKDRSWADCGECVSWSVTSAITWGTSYGRNESSASGHTASRSEGYLIDTGNWAPAFGVNMTEPLFPHVSQGFRGINLPVTSYHNPPFQVISQTSSGGLQYSGLIFDILNHLSLKLNFTYTMQLLPGFSPVAAAAVAKETAASFMTDNLDVPTSHATQVPPQLMELVRKRKVFLGAMAITVGDNLPGVNFTATVSTQTYGLMQAKPEILSRALLFVAPYTNEAWACLISALILTGPFLYLMVKWSPLSADDRGSLGLATSWQCSWYVYGALLQQGGMNLPKADSARLVVGTWWLAVMVVVATYSGNLIAFLTFPRTDAPIDSVDDLLGRSNEFVWSFANGSVVESYLSLAAANGDVKYKELFDGATRQETSKAANILESVKRDKLVYIDWKMSLEHLATSDLKSTGGCNLHVGAEDFLPENLAMMIAGDSPYLSLINDAIKRMHESGLIKKWTEDRLPPKNKCHGNMKGQEATNHKVNMGDMQGIFFVLAIGFTVAAIFIGTELVWHRKKEAAEKVFIKPFVS

>TpreIR13

MALRYALIVVMITSAAMSLPPVIRIGAIFTEDQKDSPSELAFKYAVYKINKERMILPNTTVVYDIQYVPKDDSFRTSKKACKQLSRSVQAIFGPTDSLLGPHIQSICEALDVPHIEARLDFEPSFKEFSINLYPAQDHLNRAFKDLMTFLNWTKVAIIYEEDFGLFKLQDLVKSPPSPKTEMFIRQAGPGTYRQVLKEIRHKEIYKLIIDTDPRFMQQFFRAILQLQMNDYRYHYMFTTFDLETFDLEDFKYNSVNMTAFRLVDSEAPTVIEALRHMERFQPVGHSILNKSGIIQAEPALMYDSVAVFVHGLAALDRSHVLRPANLSCEREEPWDDGLSLYNYINSADLHGLTGHIEFNEGKRTNFKLDLLKLKKEELVKVGEWKLGSGVNISDVGAFYETSATNITLVVMTREEKPYVMVKEDKNLTGNARFEGFCIDLLKWIAGQVGFQYAIRLVPDHMYGVYDPETKEWNGIVRELMEKRADLAVASMTINYARESVIDFTKPFMNLGIGILFKVTAQEPSPLFSFMNPLAVEIWLTMLAAYVCVSVTIWIVARISPNEWAEPAPCPSCKCPLQGSHVSYCDPDDEDIVLPQMVNDFTLANSFWFTIGTLMQQGSDLNPRATSTRIVGGIWWFFTLIIISSYTANLAAFLTVERMITPIENAADLAEQTDIPYGTLEGGSTMTFFRDSKIAIYQKMWRFMESKQPSVFVSDYEKGVKRVLEGNYAFLMESTMLDYAVQRDCNLTQIGALLDSKGYGIATPKGSPWRDKISLAILELQEKGVIQILYDKWWKNTGDVCNRDDKSKESKANALGIENIGGVFVVLLCGLALAILVAILEFCWNSKKNAQSDRSICAEMVSELRFAVRCGSRQRPATKLRCSSLTQGDGGVVVDDGISGCHMATTSTMIAAASTSQCRRCSSQASMMYQRRTSRHCSEQFQVGNHLQQHEQETTYVPGHEIPWLNAGSWSCGTDDGDEEIIELRSNDGGAQHQHQQRRNQLASASASAGHAAAAGAASNAAGAGTAAGPVSGAAAAAGASNYAEPITHQQQQQQLITQQQHQQLRQRQATSAIGLAQEGDQPECTMWRYDCDLAADPDVIVSPPAVMVPPAPPPPTAASIASRGTLRPILEEAPTGVPPRDHHHRYLHSVAATARSSSAALLSNKESSF

>TpreIR14

MWVFKLSQYLFLLNLCASNTLHKSLFSSEKNYQLQKLVKVLIEEVADDSQCLVTILDTYYRRRVDLAQIETFKFLPTYRIYVRENDEFTPPRQRIKRILEETTYLSCDVYLILISNGLQVANFLRYTEEERLINTRGKFIFMYDFQIFHVDMRYLWNRLINSIFIRHNFKLKRRLAGDKQLQKYEWYDLNIIMFPAQTKGFVVTRYIDTWHENRFRHGVGHFTSKIHDLKGKKLQIAVFEHVPAVTEDAKMFFDNQSDIEKNLNPLGVEFEMILIIAKVLNFKPTFYQPENVQSDRWGMSKNDTFTGLLGEAMDVAATFYLGDFYYNLRHLQILDLSWPYNTECLTFLTLESLTENSWKLLILSFRLYSWLTVILTLLLAGFISFMIAQIYKRYIGTDENYNNEYTNTQILLQSKKLRVAQKPIPAIRGWKGLYLFEDAQNSILYTYSMLLQVSLPKLPEVWSLRIFIGVWWLYTILITTFYKASMTASLANSVKRETIDTIAQLVKSEVNVGSWNEETKEFFVNSSDINLKILSNRYVVVSDEQDAVAAVVNGSLCYYENSHVLVRERVKRQILEDEKSKDSTTVSQKKISEHNLHVMEECVVNMPISLGMDKNSPLKFKVDEIVKRIIEAGLVEKWINDITQQSKTLELKQEGNAPKAFIDISKLQGAAVALGIGYFTGALALGGELFYWKKFVIKNPNFSKYRMDVFYKKT

>TpreIR15

MAARAIPTHSVVTVPKAPFVMQLSVDDRFSGLLIDLLNELARHLRFEYKIAVLMRQPVVPTTLFRFLTILEVDVWLFIIGAYFLKSLLIWIFDAWSPYSYXNVVKNPKRAGVDEKDIDDECKRIFSLRESLWFCLTSLTPHGGGEVPRNLSGQLVAATWWLFAFIVVAYLASFLTIGKFDRELRRLVYL

>TpreIR16

MRYVDDGFACVHIDHVNTILKIFNEQNSHIKFTHEVETENHLNFLDVQFIRIGSRIVTRFHANSRDKSRLCLTSPYNQRFVESTSLLLKKYRINIIPSINNKLNVIIVLGKDKTKKLDRVNSVYKFYCKSCNATYVGETKRALKKRISEHKNNKDPKFVMRLHSLDHEHEFDFKNVSILHTENNWYKRLIAEMFYIKNNVSAINKREDTRGISILYRKPVKQPPNLFSFLSPLSLDVWIYMATAYLGVSVLLFILARFSPYEWENPHDYNHQSKMTENEYTLLNSLWFTIGSLMQQGSDIAPRALSTRMVAGMWWFFTLIMISSYTANLAAFLTVERMDSPIESAEDLAKQTKIKYGALKGGSTAAFFRDSNFSTYQRMWHFMETSKAPNEVFTKSNVEGVTRVIKDKGNYAFLMESTSIEYVIERNCELTQIGGLLDSKGYGIAMPPNSPYRTAISGTILKLQEEGKLHILKTKWWKEKHGGGSCRMLCGRVMCLPTNTD

>TpreIR17

MNPADLMPQVIRQQCLEFNISNAAILFDDNFVMDHKYKSLLLNVPTRHVIVPTREAGEPLQQQISRLRDLDIVNFFVLGDESTIAAALSAASKLNFTGHKYGWFGISLVQDFSLLCQDCSSMSLMLFKPKQSQNQQQLNELMSKGLLQIPLIMSAFYYDLAKLAVLAMKSALDAGEWRRPRFVDCDDFNENVTVAMRNVDLRRRLRQVSSGSGFSPTYAGFHWGNKNGENHAKFDVDVKLWIVKDSKVVNEDQVAFWEAGIDNKLKVEKASMAASHTAVTSYRVVTVIKPPFVMRDNQTGNWSGYCIDLLNQIRDLVQFEYEIREVEDKEYGNMDEAGNWNGMVRELKDKKADIGLGALAVMAERENVVDYTVPYYDLVGISIMRRKPKAATSLFQFLTVLETDVWLCILGAYFFTSLLMWIFDKFSPYSYQNNREKYKDDEEKREFTLKECLWFCMTSLTPQGGGEAPKNLSGRLVAATWWLFGFIIIASYTANLAAFLTVSRLDAPVESLEDLSKQYKIQYAPIINSSEYRYFERMAAIENRFYEIWKDMSLNDSLSDVERAKLAVWDFPVSDKYTKMFQTMKDTGFPKNLDEALERVRAEDKTEFAFIGDATDIKYLVMTNCEFMQVGDEFSRKPYAIAVQQGSPLKDQFNNAILMMLNKRKLEALKDQWWNKNPLKQQCDKADDQSDGISIQNIGGVFIVIFVGIGLACVTLIFEYYYYRYRPQARQRHQDRSSKTKLAPGITKPMKFNLRPAPTQSFEQIDTTGTSGGGGGGGGGGGVGHRSRY

>TpreIR18

MESQNILCSNQPLLLSPDECEIIIFDVETTGLSNTDEIVQISAISLHESIFNSYIIPSVSMSKGASIVTGVSVIDGDLFLNEERLEASSAADAVNLFIQYIKTFEKQEILLAHNGNRFDTPRLLKLVKEVGKLDEFCEYVTGFADSLPIFRKVLLERTETALIYDAVHLFAMALHVLDTSQQIDVKPLSCDSTDTWDHGYSLINYMKNVEMTGLTGTIKFDNQGFRSDFTLEIMELNTKNGLEEIGSWNSSFGINFTRSFREVYTQMIDSLQNKSFIVTTILSAPYCMWKESSKRLAGNAQFEGYSIDLIHEISKILGFNYTIQLVPDGLYGSLNRETREWDGMIKELLDQKADLAIADLTITYDREQAVDFTMPFMNLVFQRKVPSNAALLLRQKVRNEPSSRLQPAKEYRRQPTTSPGKRHRRQPTTPSS

**4. OBPs**

>AmelPBP1

APDWVPPEVFDLVAEDKARCMSEHGTTQAQIDDVDKGNLVNEPSITCYMYCLLEAFSLVDDEANVDEDIMLGLLPDQLQERAQSVMGKCLPTSGSDNCNKIYNLAKCVQESAPDVWFVI

>AmelPBP2

MVSNTKQAFIYSLALLCLHAIFVNAAPDWVPPEVFDLVAEDKARCMSEHGTTQAQIDDVDKGNLVNEPSITCYMYCLLEAFSLVDDEANVDEDIMLGLLPDQLQERAQSVMGKCLPTSGSDNCNKIYNLAKCVQESAPDVWFVI

>AmelOBP1

MASNTKQAFIYSLALLCLHAIFVNAAPDWVPPEVFDLVAEDKARCMSEHGTTQAQIDDVDKGNLVNEPSITCYMYCLLEAFSLVDDEANVDEDIMLGLLPDQLQERAQSVMGKCLPTSGSDNCNKIYNLAKCVQESAPDVWFVI

>AmelOBP2

MNTLVTVTCLLAALTVVRGIDQDTVVAKYMEYLMPDIMPCADELHISEDIATNIQAAKNGADMSQLGCLKACVMKRIEMLKGTELYVEPVYKMIEVVHAGNADDIQLVKGIANECIENAKGETDECNIGNKYTDCYIEKLFS

>AmelOBP3

MKTIVILLFTLCIVSYMMVRCDDITLCLKQENLNLDDIDSLLEDESERMLRKRGCIEACLFHRLALMNDNVFDVSKFDVYLNDTDMDMDLKDSIRKIIRQCVDNAKNEDKCLTAQKFSRCVIDYVKFHITQYMISNANSNTTSEEESSDNST

>AmelOBP4

MKITIVSLLCVIYCALVHADTVAILCSQKAGFDLSDLKSMYESNSEEQMKKLGCFEACVFQKLHFMDGNTLNVEKLESGTRELTPDDFTEDVHEIIEQCVSKAADEDECMVARKYIDCALEKMKFLDDELEKIAGN

>AmelOBP5

MHVKSVLLLITIVTFVALKPVKSMSADQVEKLAKNMRKSCLQKIAITEELVDGMRRGEFPDDHDLQCYTTCIMKLLRTFKNGNFDFDMIVKQLEITMPPEEVVIGKEIVAVCRNEEYTGDDCQKTYQYVQCHYKQNPEKFFFP

>AmelOBP6

MKGLGVSLLVALLLVLLAIEDTMSKKMTIEEAKKTIKNLRKVCSKKNDTPKELLDGQFRGEFPQDERLMCYMKCIMIATKAMKNDVILWDFFVKNARMILLEEYIPRVESVVETCKKEVTSTEGCEVAWQFGKCIYENDKELYLAP

>AmelOBP7

MKKFLVIFVYILSVAVIIRANGINEILKIMAVSMKDIRYCIIHMGLTFKDFIKMQELLQEEDISEGNIKKYLTNYSCFITCALEKSHIIQNDEIQLDKLVEMANRKNISIDVKMLSECINANKSTDKCENGLNFIICFSKLLSDMYEDTFEDTLKHKSYV

>AmelOBP8

MTIEELKKTIKNLRKVCSKKNDTPKELLDGQFRGEFPQDERLMCYMKCIMIATKAMKNDVILWDFFVKNARMILLEEYIPRVESVVETCKKEVTSTEGCEVAWQFGKCIYENDKELYLAP

>AmelOBP9

MFKNYHFFFILVITLIFLYFGEADIKKDCRKESKVSWAALKKMKAGDMEQDDQNLKCYLKCFMTKHGILDKNAEVDVQKALRHLPRSMQDSTKKLFNKCKSIQNEDPCEKAYQLVKCYVEFHPEVLQTVPFL

>AmelOBP10

MKYSILLSLLITCLICSPSVHCGTRPSFVSDEMIATAASVVNACQTQTGVATVDIEAVRNGQWPETRQLKCYMYCLWEQFGLVDDKRELSLNGMLTFFQRIPAYRAEVQKAISECKGIAKGDNCEYAYRFNKCYAELSPRTYYLF

>AmelOBP11

MKAAEIWLVSLYWYLILQIALVYGEISDIDEFREMTSKYRKKCIGETKTTIEDVEATEYGEFPEDEKLKCYFNCVLEKFNVMDKKNGKIRYNLLKKVIPEAFKEIGVEMIDSCSNVDSSDKCEKSFMFMKCMYEVNPIAFIAP

>AmelOBP12

MLYNNLTIVIILIMCGVQNLRARSVNIFQDIADCVDRSNMTFHELKKLRDSSEARIKLINEEENFRNYGCFLACIWQQTGVMNGSELSTYNIAGIIEGQYHDDEDLKTFFHKIALTCEDDVHRKFLHVNDECDVALSFKLCMLKAMRNYP

>AmelOBP13

MKTIIFIFAFCLVGILAVSEESINKLRKIESVCAEENGIDLKKADDVKKGIFDKNDEKLACYVDCMLKKVGFVNADTTFNEEKFRERTTKLDSEQVNRLVNNCKDITESNSCKKSSKLLQCFIDNNLMKIFE

>AmelOBP14

MKTIVLIFGFCVCVGALTIEELKTRLHTEQSVCKTETGIDQQKANDVIEGNIDVEDKKVQLYCECILKNFNILDKNNVFKPQGIKAVMELLIDENSVKQLVSDCSTISEENPHLKASKLVQCVSKYKTMKSVDFL

>AmelOBP15

MKTILIISAICICVGALSIKDFQNAIRMGQSICMAKTGINKQIINDVNDGKINIEDENVQLYIECAMKKFSFVDKDGNFNEHVSREIAKIFLNENEINQLITECSAISDTNVHLKITKIFQCITKFKTINDILNS

>AmelOBP16

MKTFVIIFAICVCVGAMTHEELKTGIQTLQPICVGETGTSQKIIDEVYNGNVNVEDENVQSYVECMMKKFNVVDENGNFNEKNTRDIVQAVLDDNETDQLIVECSPISDANVHIKISKIFQCFMKYKTITDILNS

>AmelOBP17

MKTIVIISAICVCVSAMTLDELKSGLHTVQSVCMKEIGTAQQIIDDINEGKINMDDENVLLFIECTMKKFNVVDENANFNEKISSDIVRAVLNDNEADQLLAECSPISDPNALIKISKILECFFKYKTINQILNS

>AmelOBP18

MKTFVIISAICVCVGALTLEEFQIGLRAVVPICRIETSIDQQKEDDFRDGNIDVEDEKVQLFSECLIKKFNGYDDGGNFNEVVIREIAEIFLDENGVNKLITECSAISDADLAVKSAKLLKCIGKYKTLKEMLSG

>AmelOBP19

MKTIVVIFAFCICVNAMTIEELKIQLRDVQEICKAESGIDQQTVDDINEVNFDVEDEKPQRYNECILKQFNIVDESGNFKENIVQELTSIYLDENVIKKLVAECSVISDANIYIRFNKLVKCFGKYKTMKEVLNL

>AmelOBP20

MKTIVVIFAFCICVNAMTIEELKIQLHDVQEICKTESGIDQQTVDDINEVNFDVEDEKPQRYNECILKQFNIVDESGNFKENIVQELTSIYLDENVIKKLVAECSVISDANIYIRFNKLVKCFGKYKTMKEVLNL

>AmelOBP21

MKTIVIISAICVCVGALTLEELQIGLRAVIPVCRIDSGIDEKKEDDFRNGIIDVENEKVQLFSECLIKKFNAYDDGGNFNEVVVREIAEIYLDENEVNKLITECSAISDADIHLKSSKLIKCFAKYKTLKEIMNE

>BmorGOBP1

MWKLVVVLTVNLLQGALTDVYVMKDVTLGFGQALEQCREESQLTEEKMEEFFHFWNDDFKFEHRELGCAIQCMSRHFNLLTDSSRMHHENTDKFIKSFPNGEILSQKMIDMIHTCEKKFDSEPDHCWRILRVAECFKDACNKSGLAPSMELILAEFIMESEADK-

>BmorGOBP2

MFSFLILVFVASVADSVIGTAEVMSHVTAHFGKTLEECREESGLSVDILDEFKHFWSDDFDVVHRELGCAIICMSNKFSLMDDDVRMHHVNMDEYIKGFPNGQVLAEKMVKLIHNCEKQFDTETDDCTRVVKVAACFKKDSRKEGIAPEVAMIEAVIEKY-

>BmorPBP1

MSIQGQIALALMVYMAVGSVDASQEVMKNLSLNFGKALDECKKEMTLTDAINEDFYNFWKEGYEIKNRETGCAIMCLSTKLNMLDPEGNLHHGNAMEFAKKHGADETMAQQLIDIVHGCEKSTPANDDKCIWTLGVATCFKAEIHKLNWAPSMDVAVGEILAEV-

>BmorPBP2

MKLQVVLVVLTVEMVCGSRDVMTNLSIQFAKPLEACKKEMGLTETVLKDFYNFWIEDYEFTDRNTGCAILCMSKKLELMDGDYNLHHGKAHEFARKHGADETMAKQLVDLIHGCSQSVATMPDECERTLKVAKCFIAEIHKLKWAPDVELLMAEVLNEVSWKS-

>BmorPBP3

MARYNIVVAVLVLGVVGARGSSEAMRHIATGFIRVLDECKQELGLTDHILTDMYHFWKLDYSMMTRETGCAIICMSKKLDLIDGDGKLHHGNAQAYALKHGAATEVAAKLVEVIHGCEKLHESIDDQCSRVLEVAKCFRTGVHELHWAPKLDVIVGEVMTEI-

>BmorOBP4

MITASLHVIFALLAFVYGGKDKPVLSEEIKEIIQTVHDECVGKTGVSEEDITNCESGIFKEDVKLKCYMFCLLEEAGLVNDDGTVDYEMFTSLIPEEYFDRATKMIFSCKELDTPDKDKCERAFEVHKCSYEKDPDFYFLF-

>BmorOBP5

MKQRLRVLLLRFCILQTVLSESGVDVVKNLSLSFARFFLECDEERHFQPEVRLKVMTFWYSESSTWDRDVGCAFLCIFKKMEIDNPQDPSYRTHLELLSFANSEDNKIANQMVEIFYACGENTETDPCLWALEQVKCYKNRINQLGLTPTF-

>BmorOBP7

AVTEEELKIEFTKLVMKCTKDHPVDMSELMQLQQLIAPKKTESKCLLACAYKLNGVMTSQGLYNLEHAYKIAEMSKNGDEKRLENGKKVADICVKVNDVEVSDGEKGCERAALIFKCTLENAPKVFKFGSSEYNCQ-

>BmorOBP8

MLRVVVICVCFLVIAPYGINASSLDDLKMVYKNVIKECVGDYPITAADLKLIKARQIPNDDIKCVFACAYKKTGMMTEEGMLSVEGIKDMSQKYLSDNPEQLRKSKEFAEACSSVNDQQVSDGTKGCERAALIFKCSTEKITNFGFEL-

>BmorOBP9

MLRVVVICVCFLVVAPYGINAVSYEQKIKIRDQLDRAGFECFKDHKITEDDIKNLRANKPATGENVPCFIACVMKKTGVMNDQGVIRKGPVLELAKKVLADDKDIKKLQDYIHSCSHVNSETVHDKGKGCEFAMQAYTCMSANASKFGFNI-

>BmorOBP10

MLRVVVICVCFLVIAPYGINAVSDEQKIKIREQIDKSGFECFKDHKITEDDIKNLRARKPATGENVPCFIACVMKKTGVMNDQGVIHTEPVLQLAKKVLTDDKDIKKLQDYIHSCSHVNSKTVHDKGQGCEFAIQTYTCMSANASKFGFDV-

>BmorOBP11

MSANSFVVLAFCALAVGVNALTEEQKAEITKSSLPLIAECSKEFSVNQGDIDAAKKLGDPSGLNSCFVGCFMKKAGIINASGLFDVAATIEKSKKYLTSEEDLKAFEKLTETCAPENDKPVSDSDKGCERAKLLLDCFVANKGSFSVFSL-

>BmorOBP12

MTSFMVFFVLSVLTLKYSDALTDEQKNKIQSKFIEIGAECIVEHPISIDDINSFKNKKFPSGVNAGCFVACIFNKIGLFDDKGNLSHNSALEKAKGIFNADEEVKNLEEFLNRCAKVNGEAVGDGVKGCERAKLAYNCLIENSLEFGFNIDF-

>BmorOBP13

MLKIHVLLCFGMAILYFGSAKAVTPEESKAFEAFAKPVIEQCQKDFGMDKESFAQKNLDEIDECLIACVVEKFGITNDEKIDGDALKALVTKFVGNEEERNKINKIVEECTEDANKSGDGTCNTSTILFLCLLKNGKDLWGF-

>BmorOBP14

MSRQQLKNSGKMLKKQCMGKNDVTEEEIGDIEKGKFIEQKNVMCYIACIYQMTQIIKNNKISYEASIKQIDLMYPPELKESAKASAGRCKDVSKKYKDICEASYWTAKCMYEDNPKDFIFA-

>BmorOBP15

MFLKNIFIECVLLYFVMLNTSFVNTMTKQQIKNSGKILKKACISKNDVTEDQISDIDKGKFIEDKNVMCYIACVYSMSQVVKNNKFVHDAMVKQVDMMFPTEMRDAVKASIANCRGVAKNYKDICEASFWTAKCMYEFDPANFVFA-

>BmorOBP16

MRISFLFLISVTIITFDSVFAMTRAQVKKTMTIMKNQCMPKNGVTEDQVGKIEEGIFLENHNVMCYIACVYKTIQVVKNDRLDKDLISKQIDVLYPQEIRESTKKAVGDCINLQEKYDDWCEGIFRSTKCLYEKDPANFIFP-

>BmorOBP17

MTRQQLKNSGKIMKKTCMPKNDVTEEEIGQIEQGKFLEQRNVMCYIACIYTVTQVVKNNKLSYDAVIKQVDVMFPAEMRPAVKAAAENCKDISKTFKDICEASYWTAKCMYDFDPKNFVFP-

>BmorOBP18

MILIVIAKFLILISLCETMTMKQIKNTGKMMRKSCQPKNNVDDEKINPINDGVFIEENEVKCYIACIMKMANTMKNGKLNFEAAMKQADLLLPDEMKEPTKEAIVACRKVADSYKDVCDASFHVTKCIYNHNPSVFFFP-

>BmorOBP19

MTSAKTDVEIKAWFLGQAVECSKDHPVTTEELRMHKHELPDSKNAKCLMKCVFRKCNWLDSKGMYDINAAYASSTKDFSDDKTKQENANKLFDTCKSVNEENVGDGEEGCDRSLLLAKCLTKAAPQVSIYYS-

>BmorOBP20

MAVHIFLILASYMALAAHGQLDDEIAELAAMVRENCADESSVDLNLVEKVNAGTDLATITDGKLKCYIKCTMETAGMMSDGVVDVEAVLSLLPDSLKTKNEASLKKCDTQKGSDDCDTAYLTQICWQAANKADYFLI-

>CfloPBP1

MKSLAVCLLVVCLGALRVNAGEIPEELKGLVEGLREKCHRETGVDIEHVDKTVEGYFHPSELLGCYFSCLFNSFDLLNHDGHIDFDKMIAKIPPAYMEHAMEMINACRHLTGKNPCDSAFNIVQCFQKTNPEKFFMI

>CfloPBP2

MRIYIFLATVALTAMYYRSVKSAGQKLDIEGLRNMLKPVSGSCKRKTGASDELVSKTHDGEFPRDRNLMCFFRCISIMVKTMDKHGNPRLDDIERYAATLVDESIEPLLRKVGVACYEEVPKGHDQCEYAIAYAECCFKMDSSLYFLPY

>CfloPBP3

MKFYYFALCFLAVIVYSHAKDKDSSEGPNSYEECLAESGIKEEDIEKLANAKVPKLRCLMACVFEKEGILTDKKIDLEALKESMIEDIEGIDEKTATEIVEICTNYAKTAKDICDETNIIGICTKEQFDERNIKIKM

>CfloOBP1

MVSMKGLLVLILFASVVCVYSHHHHLEEKNLTDEQRKKIRDEVEECITESKVDKKLLDDIKDGKDFTPTRELDCFSACVLKKNGVMKEDGTIDQDKPSTNDKVKECRKLAGADACETGGKVMECFAKNNLIPKF

>CfloOBP2

MRLIAVVIVYLLGLVLLIHAKSKSSHDPHQPVDPNEERRRLYLEVIANCTKEVGITFEDYALSFINNDTKGIYEKVKCVNACMFKAHGIMRPNGTINAEKAVEHLLSSKLGVDVHVLHDIIIECSEGKGENDCDTAQKLMDCVIVR

>CfloOBP3

MKVLFAFLVICIVGAFCATLTDEQKQKLKGYKEACIAETGVDKAVIDAIGKGGPIKRDSKLDCYAACLLKKSGVMKEDGTLDIEAVRTKAATINADQEKVKKVIDKCKDLSGKDTCEKGGNILTCFFENKDIPILD

>CfloOBP4

MKHFAAVVLFVAVCFVGAFSESLSNEEAEKLMEYKESCTAETGVDEAVLMQPYDDKEELVQDEKLNCYFACILKKMDMMDSDGTINMETARSQLLRDLCPKKIDESVECLSQVGDSPCNTAGKIFGCIMKIRTTCHDN

>CfloOBP5

MLGQLQGQFVLATLFLGIVCAGTRPSFVSDKMIATAASVVNACQTQTGVSTADIESVRNGEWPDTQELKCYMYCLWEQFGLIDEKRELSLNGMLTFFQRMPAYRNEVQKAIIECKALGSYFGVNTTDDSCEYAYSFNKCYAERSPKTYYLF

>CfloOBP6

MKITIFVFVSCLFYSTLNSVLTLKCRTGNQQSDDQFQKIIQICRRRYLGYDRETGYNLRRTDSQESDSDSEEDMFDRKFLTAKGNRYNYNNKNDNSGSSGRSSFSSRDMTTHRNWNNNNPSYNNRNNTRSPYDNNNNNFNNRHQVNYNSRRSNNNNMDSRGYSDNYSISGSNNNNNYNYNNNDDDREQACVVQCFFNELNLVDQRGFPERSSVVNIMTQNIQDSELRDFVEESVIECYHMLNNSGRQEKCQFSQNLLSCLTEKASERCEDWEDE

>CfloOBP7

MRVYIFTLAVLVVAILVPSNAEKTIGDHIKECKKELEIPEGTKKFSPQDPKFKCFLTCMKKKFGVIVDGKVNADAAIKFYSKKKELEEAIKHIIKECADAANKLSDCEVGYTYLKCLHEKQKGKKSEVETKKENNEE

>CfloOBP8

MKVFICMLAVLFVAVCTPSQARDHKKMNECKKEVGITNEFKKLEKINFEDAKVKCFFTCMLKKKEMIVDGKLNEDIIIEKMSKHEEMNDTKKEIVQGCVKEANEKSDCEVGGHYKK

>CfloOBP9

MKVFLCSLAIILVAICLPCQAKGGVFKECKTESGLTDEIKKGQFPNLEDSKVKCFITCLGKKFGIIVDGKVNAEKFIEKLSKKLNLDDAKKEAVKGCAEEANKQSDCEVGSAMLKCLKEKGISMKGKKKENENSD

>CfloOBP10

MRILIFLSTFALIVIYYGSVECAEKLDVDGLRNMLKPMSNTCKRRTGVSNELIANTHNGEFPRVRPLMCYFKCLSMMLKTMDKDGITRLEDLQKATALLVREDIEPKMRKVATECFEEVPNKGDPCEYAIEFVECSYKKDKSVYFIPF

>CfloOBP11

KLYFYVGYLQPTRILQDPHQPVDPNEERRRLYLEVIANCTKEVGITFEDYALSFINNDTKGIYEKVKCVNACMFKAHGIMRPNGTINAEKAVEHLLSSKLGVDVHVLHDIIIECSEGKGENDCDTAQKLMDCVIVR

>CfloOBP12

MNYFAPIVLLSIVYFAGAYPEVSSGPQKNTKQEMQYKNSCITESKINKTLVERVEKGEMEAEMFEDEKLNCYYSCFFKKTGVMKPNGTIDEKVLFSSIPPNVPKEIIDKATKCIPQVSVETNHCEIVGNFLGYLMKIESFLDKFLN

>CfloOBP13

MKFFAVIVMCAITCLVVALPQRLSDEQETKQFNQEVENCIAETKVEKATLYQFVKEFQENGEMIQDEKLHCFIACLLEKNDVIKRNGTIDKNTLLSEMPTDMPQKKINEATECIPEAMLWN

>CfloOBP14

MYMWILMVLTIGISSISAEPIVLVRSISDTQKFCYIESGFNYGKLEKIKLRRLFPSDMSYKCYIGCFCTKKKYPMPDTRVMNEKIMRDALNRSLPQKTVNNAMASCKNVTVTEDICGTGEKLVGCLIKQGIILADFMGYIEEDKL

>CfloOBP15

MNKSILIIVAACISFVFAEPKESVLIPDFRRKDVLRARQSFCYAGNGFNDEKLREIKEFRIYPSDDSYYCYTGCFTTETKSIITGEKVFSEQYIKAALYRTFPEKKANSIIAKCKNIKVSKDICDAGKTIHSCFTIQQIVLADFIGYLDSDVI

>CfloOBP16

MNKLLMLTFALSINFVLSGKSVKEALEDIRLISRLQNYCYNETGFTREKHELIRDARTYSSDINYFCYVGCYCTHVKDLWLTKRTLNYEAVAASVTRFLSVPVAYHVINQCTNISTNSLCATGKKIHDCFAERNVIITGSIGYFDGDEI

>CfloOBP17

MKLLILTFALYIGFVLSQEYYKESQRHKRNYITSRQNFCYNETGFDKEKHTQIRLTRAYPLDISYFCFVGCATTQPKGLFLSTRSMNTTFLLEALTRTFTVEEANNIIDKCKDINTNGLCATGRSIHECFAKQGFMIADWIGYLDGDIV

>CfloOBP18

MRTAVVIFLPLASLLSFAEDPIKDINKDYIKSCLIENGFDPQQYPNGMRNVKVPEKQEQNRNCYYSCMMKKMNLMKADGSLNEDALRTKFNLNLDTLHKALNTCRTQVKNDNCKMAACLMANRGA

>CfloOBP19

MVSMKGLLVLILFASVVCVYSHHHHLEEKNLTDEQRKKIRDEVEECITESKVDKKLLDDIKDGKDFTPTRELDCFSACVLKKNGVMKEDGTIDQDKPSTNDKVKECRKLAGADACETGGKVMECFAKNNLIPKF

>CfloOBP20

MKHFAAVVLFVAVCFVGAFSESLSNEEAEKLMEYKESCTAETGVDEAVLMQPYDDKEELVQDEKLNCYFACILKKMDMMDSDGTINMETARSQLLRDLCPKKIDESVECLSQVGDSPCNTAGKIFGCIMKIRTTCHDN

>DmelOBP8a

VPMRSSPQSLALLRARDQCGRELTAAQRLQLDRMQFEDAAHVRHYLHCFWSRLQLWLDETGFQAQRIVQSFGGERRLNVEQALPAINGCNAKTSSRGSGAQTVVDWCFRAFVCVLATPVGEWYKRHMSDVINGNA-

>DmelOBP19

GVTEEQMWSAGKLMRDVCLPKYPKVSVEVADNIRNGDIPNSKDTNCYINCILEMMQAIKKGKFQLESTLKQMDIMLPDSYKDEYRKGINLCKDSTVGLKNAPNCDPAHALLSCLKNNIKVFVFP-

>DmelOBP28a

FDEKEALAKLMESAESCMPEVGATDADLQEMVKKQPASTYAGKCLRACVMKNIGILDANGKLDTEAGHEKAKQYTGNDPAKLKIALDIGETCAAITVPDDHCEAAEAYGTCFRGEAKKHGLL-

>DmelOBP44a

SDYKLRTAEDLQSARKECAASSKVTEALIAKYKTFDYPDDDITRNYIQCIFVKFDLFDEAKGFKVENLVAQLGQGKEDKAALKADIEKCADKNEQKSPANEWAFRGFKCFLGKNLPLVQAAVQKN-

>DmelOBP46a

RSTPPALDEDCELNSVDTMHDFCCDLHDESPQFSDCQMEWHEKIPYETDEEEQTYMFCTAECSFNSTNFLGRDRRSLNLNEVKEHLESDLVNDADIKLLYDTYVKCDKHALSLMPHKGVKQLSKRLSRLGCHPYPGLVLECVANEMILHCPTKRFRQTAQCEETRNHLKQCMQYLKYKS-

>DmelOBP47b

QATIDCQRPPQLVDPALCCKDGGRDQVAEQCAQRILGTANGQKAGGPPSLDTAACLAECILTSSKYIDEPQKLNLANIRSDLSAKFSNDTLYVETMTMAFSKCEPQSQRRLAMIMQQQQQVQQQKTQQQQPRCSPFSAIVLGCTYMEYFKNCPDHRWTPNAQCTLAKAYVTQCGLGA-

>DmelOBP49a

DVDCSKRPSFVNPKTCCPMPDFVTAELKQKCIKFDMTPPPPPDGEASGSFESKRRHHHPHPPPCFFSCIFNETGIYQNRKLDEAKLNAYLQEVFEDSSDLQTTATQAFTTCATKVADFEANLPPRPAPSPPPGFPMCPHDAGHLMGCVFRNMMKNCPDSIRNDSQQCTDMKEFFTKCKPPRGPPPSAEDM-

>DmelOBP50

AKCRAAPKSVQNVHVCCSAPLPNWGVFNRECHKSAIQASVSINRISKSKVNLANFLIKCRLDCDFNASSVLQGNRLIQAKVRPMLERAFSNEPTIDAYESNFAKCSTVVRSKYQELSPLSRQSDACDRHALFYSLCAYARLIFTCPDKMWQRNNRMCQEAKAYAKKCPWPALKMFMRNT-

>DmelOBP57

IQSLSLLEETNYVSDCLASNNISQAEFQELIDRNSSEEDDLENTDRRYKCFIHCLAEKGNLLDTNGYLDVDKIDQIEPVSDELREILYDCKKIYDEEEDHCEYAFKMVTCLTESFEQSDEVTEAGKNTNKLNE-

>DmelOBP58

LKCRSQEGLSEAELKRTVRNCMHRQDEDEDRGRGGQGRQGNGYEYGYGMDHDQEEQDRNPGNRGGYGNRRQRGLRQSDGRNHTSNDGGQCVAQCFFEEMNMVDGNGMPDRRKVSYLLTKDLRDRELRNFFTDTVQQCFRYLESNGRGRHHKCSAARELVKCMSEYAKAQCEDWEEHGNMLFN-

>DmelOBP69a

VEINPTIIKQVRKLRMRCLNQTGASVDVIDKSVKNRILPTDPEIKCFLYCMFDMFGLIDSQNIMHLEALLEVLPEEIYKTINGLVSSCGTQKGKDGCDTAYETVKCYIAVNGKFIWEEIIVLLG-

>DmelOBP73a

VEYLIRFETKKAKCLNPPRTARKVESVIRECQDEVRNKLVNEAYEILKEQVSQNQPPIDPNDDSIDFIWPSVPEAPSLDHSPNISQYEYIVYDEPEPQRHVARLMRNIRRLDVASSGIYHPTLVPLEDKRIAGCLLHCVYAKNNAIDQRGWPTLDGLVHFYSEGVHEHGFFMATLRSVNLCLRTMTARYGVNRKELPKKGESCDLAFDVCSHMNTNIFKDLQFWAPFISYVNESRAINYI-

>DmelOBP76a

MTMEQFLTSLDMIRSGCAPKFKLKTEDLDRLRVGDFNFPPSQDLMCYTKCVSLMAGTVNKKGEFNAPKALAQLPHLVPPEMMEMSRKSVEACRDTHKQFKESCERVYQTAKCFSENADGQFMWP-

>DmelOBP83a

QRDENYPPPGILKMAKPFHDACVEKTGVTEAAIKEFSDGEIHEDEKLKCYMNCFFHEIEVVDDNGDVHLEKLFATVPLSMRDKLMEMSKGCVHPEGDTLCHKAWWFHQCWKKADPKHYFLP-

>DmelOBP83b

QEPRRDGEWPPPAILKLGKHFHDICAPKTGVTDEAIKEFSDGQIHEDEALKCYMNCLFHEFEVVDDNGDVHMEKVLNAIPGEKLRNIMMEASKGCIHPEGDTLCHKAWWFHQCWKKADPVHYFLV-

>DmelOBP84a

LQDHAKDNGDIFIINYDSFDGDVDDISTTTSAPREADYVDFDEVNRNCNASFITSMTNVLQFNNTGDLPDDKDKVTSMCYFHCFFEKSGLMTDYKLNTDLVRKYVWPATGDSVEACEAEGKDETNACMRGYAIVKCVFTRALTDARNKPTV-

>DmelOBP85a

MSPGSVVFSMFLTRPSLDKGNSECRKSLNLPAHRKFNFAELYTINMCIEECNFIGCGYIEIDPPFRLDLANIRTNLQTIAPQPQNESIPFLVDAYRKCELFRSSHGRRFTLHLPDIEFIEEPCNPFALQITICVRIHAMQKCPSEFYVDSDECRLAREYFTQCVGDIETNLA-

>DmelOBP93a

CDVQKNDKAINSCRKSLLGNNSTNSNGEVRNLKSDKVALHACIAECSFRTNGFLLSNGTVNTQALQKSYQQRYKNDPNMSQLMLKSLNSCTDYARKRVQEFQWMPKKGDCDFYPATLLACVMEKVYINCPTSKWKNTSDCTAMWKYLVACDDVASNKKK-

>DmelOBP99a

DYVVKNRHDMLAYRDECVKELAVPVDLVEKYQKWEYPNDAKTQCYIKCVFTKWGLFDVQSGFNVENIHQQLVGNHADHNEAFHASLAACVDKNEQGSNACEWAYRGATCLLKENLAQIQKSLAPKA-

>DmelOBP99b

DDWTPKTGEEIRKIRVDCLKENPLSNDQISQLKNLIFPNEPDVRQYLTCSAIKLGIFCDQQGYHADRLAKQFKMDLSEEEALQIAQSCVDDNAQKNPTDVWAFRGHQCMMASKIGDKVRAFVKAKAEEAKKKAA-

>DmelOBP99c

ASVWKLPTAQMVYEDLEKCRQESQEEDAATLRCLVKKLGLWTDESGYNARRIAKIFAGHNQMEELMLVVEHCNRMEQDTSHLDDWAFLAYRCATSGQFGHWVKDFMSQKEVER-

>DmelOBP99d

DHHHHHHDYVVKTHEDLTNYRTQCVEKVHASEELVEKYKKWQYPDDAVTHCYLECIFQKFGFYDTEHGFDVHKIHIQLAGPGVEVHESDEVHQKIAHCAETHSKEGDSCSKAYHAGMCFMNSNLQLVQHSVKV-

>MmedPBP1

MVRVILNYIFLGPLLQTFIVSAKLPDWVPAEIIDMAQGEKGRCMSEHGTTEDMINMVNEGNIPNDPKLTCYMFCLFESFSIIDEDGVLEYGMLTEMFPDDIKAKAESVLSGCAEQPGADNCEKVYKIATCVQSKSPDMWFMI

>MmedOBP1

MKNIIIFTTAITIFTFINFSQTEARMTMTQIRNAMKPLGKTCLGKTGLSKEVQAGQHNGEFPEDEALMCYHSCLLKLAKISDKSGNINLDTVHKQIDLMMPEDLIARAKAVTTDCFGEIKSTEICRMSFEFVKCYFIKGPEIVFFP

>MmedOBP2

MKSIIFLGVLLTVLISNKAEAKSVQKRECPFKKPFEANAPKCMDKISEENMGRMMQGNMDNDEIRCFVGCVFENAGFVKDNKVQMDKVREAVDNFVDDYKYSKEVGDQVYGVVSDCAPQAEKGANNCEVSSNLLICFKTNNKFT

>MmedOBP3

MRGSVLAIVACALVVGVLGDDDMKEKHKEIFKKCAEETGVTKEDLHNHKRGEEPETKIKCFHACIAKADGAMVDGKLNKDKVIEKIPADLPDRERIIEAVTKCSEQTAADECETAHLVFKCLRENKALPHPPHHHHHHHDE

>MmedOBP4

MKCFTLTAAGILFTVLITVNNASSNSNMEELVKKSMEETFKACKDKLTPENFALLNKDPHADNQEIKCFKACGMNHAGIMADGKIQIEKMEEKLNSLLGEDKKDFSKIIIGRAKPCVEEANKGENECDVAAGFEACVQKTINTKSDN

>MmedOBP5

MKNFVVIVILALYFTATTESLQEIMNTFQKARLEVRAPCLHLLSNETLTTLKTRRHLDNPEIRCFKACLMERQGYLKDNKIFIDEYEKLIDVNLKRIKELNMKFARACVNEAEKSENKCELAHNYNRCILHQTRKHYNQTAEENDENQNQHL

>MmedOBP6

MKNTLFFTLAAAFLLGYNIPHVESRMSMAQTINTMKPLGKTCAAKTGLSKEMQDGQHEGQFPEEEALMCY

HTCLLKMAKVADKTGKLNIDAMVKQIDMLMPEDLVDKAKTACSGCADEVTATEGCRPSWEFMKCWYGRAP

ELYFFP

>MmedOBP7

MDSNIKYMCFLYIFIVVFMFFSEAIDQSDPHASTRKKCSGEFKLTDEILKLGEQDPSDFSCYLFCLFKDINIMNQKGEFDPNLAAQEVQDNLREAARKYIFMCYDLVKPNMTSDGCKNALEMVQCFKEKAPEMYEMLGLFHPPSNEPLKMTQ

>MmedOBP8

MAKFLLSSVGVLVLIAYVQSGPVPEEFKDVQPTIRAACVKESGLTNEELVNKAALGEFTDDPQLKCYLKCIFDQFRLVSKRGINFDAMLALSPPSMKENAIKMVKECRDTKGKEGDLCDLSFEVTKCLYNSNPETYFIL

>MmedOBP9

MKIFAVIFAICIVYAVAIGNLTEEERVELDRLANICVNETGFYEGHNSDDPAKNWISYGFKLQCYFSCMLKKMNIMNEDGTLNEEMIRKKIGDEVPADKIDAVITKCKDLKGANKCETATMIMKCYSDERLSLDPAEKSV

>MmedOBP10

MAIVRICNTTDPVDLRVLNDYLMNHNLNRLHIKSHHPLACFLLCVYSEFNWMDRHGGFKVHNIKAWMLRAELSENDTDILLRKCISLELTDPCTRAQYFTECFWTNHQDVTVDHRHSLHSIMHKDVHQDKIYN

>MmedOBP11

MKIIAVIFAVCFAGALAELTVEQLAKLREHSTACITETLVDDANVDAAMHHNIWRMDDLKLRCYFFCLLKKLKVMNEDGKLNEEITRQRLANLFPADRIDGVIMKCKEMKGADACETAILMAKCHADERGLLGPSPRSA

>MmedOBP12

MKGVKSIPLIAIAAVFCISINFFSTDAAFTVEQIESMMKPLGNNCVSKVGLSPELQEANRKKEFPEEKPFMCYLHCLARVTKVFDKNNQIDLEGTLKQVRLVMPDHLVEGSVKAYTVCSRAAISEDPCEKAFQYAKCYYETDAPSYFYP

>MmedOBP13

MKNILLGICIFIPSVFCGTRPSFVSDDVIGFAASGVNACQRQTGVATADIEAVRNGQWPESRQLKCYMYCLWEQFGLIDEKGELSLNGMLTFFQRIPAYRVEVQKAIRECKSIGEYLANGDNCQYAFTFNMCYAEVSPKTYYLF

>MmedOBP14

MKLFAVLFAVCFVGALAELTPEQLAKLHESRSTCITETGVEEGNVAKANDGEWLMDDLKLRCFFSCMLKKIKVLNEDGTFNEEKARKRIANDLPADKIDSVITKCKDLSGGDVCETAMLMMKCYADEKALTKIITEKSS

>MmedOBP15

MKIFAVIFAVCFVAALAELTEEQKAKLREHRTACVTETGVDEANVDAAKQGDWKMDDLKLRCFFACMMKKIKVMNEDGTLNEEITRKRMANDLPADKIDGVMMKCKDMKGADMCETAMMMMKCYADEKAFTKIITEKSS

>MmedOBP16

MRYSVFVFVGILFTFFISSDAESSGEKECPLKKAFQESIDACKDKLSEENLALLEKDENADNEDIRCFKACILNDSGVMSNGKIQIDKIEEAINAAIENVKEDEEKAKAIGESMINGAKNCAGPAEEGENECEVAHRFITCLMEHAAEEKKKHNE

>MmedOBP17

MYRLAVVFIFASVVVLSESAITAEDLVKFGMARRTCDRTNRVDPSVIDRVLQGEMINDPQFDCHVACVLKELNLLTADGSLNVEVAASKVPENLPYYNQLVGAIRSCGSRKGNDQCETAHMLFVCFHENNIPNLILG

>MmedOBP18

MQMQVNADIKRDCRKQTGVSWASLKKLKAADYNQNDPKLKCYLKCFMQKNGIFGEDDIDIEKALRHLPTGIKGPSKTTLEYCKKIPSVDSCDKAFQLAKCYFKAQPEVLKSVSFV

>NvirPBP1

MKTIVFTLCMMTVAVTCSPRPGGRGGGSMFSRESVKKCMAEMDIKREDIKTLKQNNDPKLSCLNACAMTKEEIMDEAGNIDADKLIKATLEIVQKKKPDINVEELETAMLSCIEKAKEVEDKCMKAKTLVVCSHEYWKANVKGNPSSAGGEEE

>NvirPBP2

MMKNLTLCFLVVVLGVIKVNGNEIPHEIRHMVVGVRDKCHRETGVDIEHVDRTVEGYFHPSELLGCYFSCIFNHFDLLDKDGHLDWDKLVPRIPESFKEHADEMIAACRSTTGKDPCDSALNIVQCFQKTNPSKYFVI

>NvirPBP3

MKLFFVTLCVLFAAVYGATKSDSKSEKIFHECLEENDIKESDFKNLEGKKDPKMRCLMACILEKEGALKDGEIDGDVIKKDIIAEFTEVDAQKISDAIDTCVDGANDLSDICEKTSFIGECLKVELDKLEMNMN

>NvitOBP1

MKAFLCVLGVIIAAASASCGMPEEMKQAFKECHTELGMPDEKPHGPPNPDDPKIKCFHACIMKKAGKMVDGKLDADKEIEFAKKRMPNADDSMIEKITECVKTANEQSDECEVAGAMHKCIMEKVGSPPHHHRH

>NvitOBP2

MMQGSLCALVVLSLVCLVRAGPPDWISAEILEMVQSDKGRCMAEHGTTEALIDDVNKGNLPNDKAITCYMYCLFEAFSLVDEEANIEVEMLVGFLPEHMQAVANELIDVCAKLDGADVCDKMYVMAKCVMEKRPDLWFML

>NvitOBP3

MHSVFEVHRKLVPAAFFNMKFFTVATFAMCIIGTFAAFTMTEEQAKDLQDKLDCIKETGADIATLLNIKNGIPTLYDDKVNCFAACMLEKFNIMKPDGSMDETVARLRASKSMSQEKVDRVLSSCKSEVGKDKCETGGKILECLMKNDAVPILS

>NvitOBP4

MKSFAVIFAFCFVGAIAALTEEQKAKLKEYKYACITETGVSEDVIESVKKGEQVTFDEKLNCFSACMLKKVGIMNADGTVNEEVARAKVPQDLPKDKVDQVINTCKAEVGKDSCETGGKVLACLMKTKAVSVLH

>NvitOBP5

MKTFAIVLTLCIVGAYASTLKDDQKAKLREYKESCITETSADKAVIDSIIKGGPINRDEKLDCFSACMLKKIGIMRPDGSIDVESARAKAATTNVDVAKANEVIDKCKDLKGKDTCETGGAVFGCFITNKDFPVLN

>NvitOBP6

MKVAIVACVLTICSIFAGSKADLTEDQRKILQPLKDECFQETGLDAVTLEKFKKEALQKFKTTGEVSNDEKVNCFSACMFKKIGFMSEEGKFEEDTVRALMSENFPPETLDKAIENCKNEVGKDHCETAAKLIVCFMNNKAGMENV

>NvitOBP7

MRLTLQLITLVSLVAIFKTTESKMTMDQIKNTLKPFKNSCIKKISPDVAMVEATKSGQFPEDATLMCFLKCVLSMMKVMKNGEILLPSIMQQIDIMMPDEYVETMKEICTNCYEMSLKVDDACEKAYVFVKCYYNTNSELYFFP

>NvitOBP8

MRRSILITSILIILISQYKLVKCKKMNLDELRDMLRPMSKSCKSKTGVSDEMVAATHQGIFPREKPLMCYFKCLSVMLKVMNKQGEIKPKDVERQIDLLVIPELAPTLKKIGTDCYNKVAPTNDACAYAFEIVMCGYQTDPKYYFLP

>NvitOBP9

MKSILFIFAIVCVVGVFSDDDKKDLTREQILECVAESGVDETKVEDIKLGNQGLETTREIDCFAACVFKKQGIMNEAGVITPDKPMDNEAAKQCVATTGADACDTAGKVLKCFISNNLVSLMDLDDD

>NvitOBP10

MSGQSLLLLALGIFLPHCLAGTRPSFVSDKMIATAASVVNACQMQTGVATADIESVRNGQWPDTMELKCYMYCLWEQFGLIDEKRELSLNGMLTFFQRIPAYRVEVEKAINECKALATGDTCEYAYTFNKCYAERSPRTYYLF

>NvitOBP11

MIVGRRLKYAERNDVIKADQACSNIIVLLFSSVCTATKEEEEFKSELAECKNLVGVTEDYVRDVFKSGLKGADEKFKCFIACLIQDSYKFNDGGVFDAERTIANDRGPAGLLRDYTNKALKACSNIKGYSECDAIFKVYKCMVENVEKLFNARNDRPSG

>NvitOBP12

MKKFTLIFVSCYLVFSSMHRVMSLKCRTGNQQSDDQFQKIMQICRRRFLGYDRENSRDFNARDTDSQESSDSDSDEDMFDNKFLTSAGSRYNNGKNGDKMGSGSSSRYDYSRNNSTRQSSSGRNSSNRSSNGNRSYYDNGQINSNRRSMRNSGMNSRGGYGDNNYSNTRYNSNNNNNNNNNNMNRNNMNGNNDGNYNNDGNNEREQACVTQCFFNELNLVDQRGFPERSAVIGIMTQNIQDPELRDFVEESVIECYHYINNNNSGRQEKCQFSQSLLSCLAEKGSERCEDWDDE

>NvitOBP13

MSGQSLLLLALGIFLPHCLAGTRPSFVSDKMIATAASVVNACQMQTGVATADIESVRNGQWPDTMELKCYMYCLWEQFGLIDEKRELSLNGMLTFFQRIPAYRVEVEKAINECKALGKYYATGDTCEYAYTFNKCYAERSPRTYYLF

>NvitOBP14

MKNLALLLLTLCVVSCLLINGARAGVSREQMEKMANGFRNTCVGKTGADMSLVEGIRVGNFVEDPTSMCYTKCIMGLMKTFTKQGNIDVEMLVKQINVMASPDIAGSMVTNARKCHAETSASDPCELAWLFTKCIYAADPAVYFFP

>NvitOBP15

MKIVVLCLVVLSAVACVSAGYREYQNACLDENGLTKEEFYAMKRNQDPRSGCVTACIMKKNGSMKHGIIDARGIKRRMRTLLAPFISKDKLYEKIDYCVDEAENHVGVCEKAYVLQKCLRTPRANNVQGERQKMID

>SinvPBP1

MKTFVLHIFIFALVAFASASRDSARKIGSQYDNYATCLAEHSLTEDDIFSIGEVSSGQHKTNHEDTELHKNGCVMQCLLEKDGLMSGADYDEEKMREDYIKETGAQPGDQRIEALNACMQETKDMEDKCDKSLLLVACVLAAEAVLADSNEGA

>SinvPBP2

MKHLVLCACVLIFALSNATEIQQEIQNEIKLRLDLEACLIENGLNNSGLYSMNEVSINVHTKPGNEERTRKNGCFMACVLKKQNLMEGTNIKEDEVIARLYELTRQDLKVILGKIVRKCLEEKRDITQECAKCFSIFECIIQTMDKFPREHEHEEIVTTE

>SinvOBP1

MTRLLLVVLFSLCLLLIESVNSLKCRTGIQQTDDQYRKIMQTCKKRFTTDDDDDDDYSNDSSSNENDDNDSSSVDLFGTRFFVSGGSKFSNMRSWKDPNENRNRGNDQRNGNDRRYSVSYTNGNWKNAQYPFQGSNNRDYSYSEGAGRPSYDQMYNGNSENYDKQQKQTCINQCFFNELNMVDQRGFPEQVSVIQFLTRNIHNPELQDFIEEAVIECFHYLNSDIRQNKCYFSENLVTCLIDKGKERCEDWDN

>SinvOBP2

MKTIVIVLTISFIAVLGQLTDEQKANLREYKESCINETGVDTVLIENAKKGQIAENDEKLACFATCLLKKAEIINADGHIDWKVARSKFPVPRERLDQIYNACKHITETGCEKGGKLFKCFMDNNIYLLQ

>SinvOBP3

MKETGTIFFISLVFMANLQNIESKRYTFEDIKVALEPLKKHCIDRVGKDQKVIDDANNFKIVPDWKTQCYYKCIMLNTKMMKNDKIVEKALINIAEHMLLEEYLPVVVKTIEQCHSTATKSMEGCALAYEYYKCLYDVNPTIAMYA

>SinvOBP4

MKAIIIVLAISFVAVLGQLTDEQKAKLRTYKESCINESGVDTTILENAKKGEVAESDEKLACFSTCLLKKIGIMNADEDINWEVARAKLPPGVPQEQADQIYNACKDITGTGCEKGGKVFKCFLDNKHFHLLS

>SinvOBP5

MHVSVILGVVLLQAIYVSAAPPDWIPPEMLEMVQGDKERCMAEHGTSQALIDEVNDGKLSNDRSITCYMNCLLDAFSLVDEDGNLEAEMLISVIPEEFQEIGNKILNKCAVQDGADKCEKIYNVAKCVQGTVPELWFMV

>SinvOBP6

MEKVAFFVLALIAVVVANEIVEEMAKKFETDSATVQKCLDDTGITMEELGTSLKEWAELKDEDINEMTKQSLMKYVNFLACMMEKNEMMIDSKLVVDKIVESAQNDKDLLPPVPKEVLTECLTALNENSEISREDRVFGLMFCMMDGQTDKK

>SinvOBP7

MKTLVFHIFIFALIAFASASCNSAKKIGSHYDHYQTCLTELGVTEDELFSVGEVTNGQHKTKHEDTKQHKNGCIMQCVFEKLGLMIGADFDEEKMREYYIKEKGLQPGDQRIDFLSSCMEQTKNMEDKCDKSLGFIGCVLMNEVSLPASNEEA

>SinvOBP8

MCARLLICMITIALFAVHQTNAAMTMEQIEKTALNVRNTCTSKSHADPGAVAGIQNGVFPDDNKPLKCYTLCVMKTMRTFKNGRIDDGMMIKQVDLMLPAEMAGPLKAVATKCAAEPPTGDDCSTTYQFVKCSYSTDPDHFFFP

>SinvOBP9

MKETGTIFFISLVFMANLQNIESKRYTFEDIKVALEPLKKHCIDRVGKDQKVIDDANNFKIVPDWKTQCYYKCIMLNTKMMKNDKIVEKALINIAEHMLLEEYLPVVVKTIEQCHSTATKSMEGCALAYEYYKCLYDVNPTIAMYA

>SinvOBP10

MKAIIIVLAISFVAVLGQLTDEQKAKLRTYKESCINESGVDTTILENAKKGEVAESDEKLACFSTCLLKKIGIMNADEDINWEVARAKLPPGVPQEQADQIYNACKDITGTGCEKGGKVFKCFLDNKHFHLLS

>SinvOBP11

MHHSASTHAIAIFMSVIYILFVVVIITRFKETYLSENSPFTEKETEKLRGYRTLCMNKYDVDTTIIKKAKEMENIIDYIDERLVSYVICLYKNWGIINADGHIDWKVTLSMLPGVPQKVFNEIYSACNRTTGTDYERGYELFKCFLQNEVDLL

>SinvOBP12

MKIINILLVILFLLLVGARADIKRECRQQTNVSWASLKQLKAGNIEQNDMKLKCYLKCFMVKIGILNEDSNVDVEKALRHLPRSMQESSKRILNQCKLIQAENACDRAFQIAICYVKAQPEILKNVSFI

>SinvOBP13

MRLLTVALGFLLQAWIVYCGTKRPSFVSEQMIATAASVVNACQTQTGVATADIEAVRNGQWPETRQLKCYMYCLWEQFGLVDDKRELSLNGMLTFFQRIPAYRAEVEKAIGECKGLGNYLAKGDNCEYAYAFNKCYAQLSPRTYYLF

>SinvOBP14

MARKSLILVCVSVVLTQLVVVSFSAKTDEIDWTTVHDDLRKLGATFRKKCLAETGVTIDKLEGAEMGQFPDDRKLACYFKCVMEKGGVMKKDGTINYKVLAKLLPQAYKQIGIDMMDECRDIEGSDSCEKGMKFHQCMYNANPVAFFVI

>SinvOBP15

MKHLVLCACVLLFTFQLSNSSELKQKIQEEVKIKQRIDFEACLSENGINESDLYGPTEIVSNVHTETANEEKTRKNGCFMACFLKKQNLMEGTNIKDEVIARLNEVVVTDDVEEKLRTIVRKCIKEKKDITQECDKCFPIYVCIIKAVNEERKCMQEENVRTEEEETGEPNKKK

>SinvOBP16

MKHLVLCACVLIFALSNATEIQQEIQNEIKLRLDLEACLIENGLNNSGLYSMNEVSINVHTKPGNEERTRKNGCFMACVLKKQNLMEGTNIKEDEVIARLYELTRQDLKVILGKIVRKCLEEKRDITQECAKCFSIFECIIQTMDKFPREHEHEEIVTTE

>SinvOBP17

MKGLILWVCVFIFASSSSSKLKEEKHINTNVRAIQDEIKPCLSEIGIAYEALYPPAEIIANVHTQPANKERTKNHGCFMACVLKKQNLIEGTNIKEAQVYSRLHEILDEELDGPGHQIIRKCMEEVRNMTQECEKGFSLYVCIVKEAAAHEEEAKRQKNKKN

>SinvOBP18

MKTFVLCACVFVLAVYFQSSNSSELNEQELRKIGISIRNDFNTCFSEIGITPADFVKPMEIVTNVHLQPANEERTNKHGCFIACVLKKQNLIEGTKIKEEQVYERLQLIFDENPGGPMHQIVQKCMEKVRNDAQECEKCFSVYVCTIKDMYEEEQRRKNERN

>SinvOBP19

MKSLVLCACVLLFAFQLSSSTELKEKLKNEEKNIENILETCLNEQGLSRNDMYKEEELMTKVHTESVNAERTRKVGCFVACAMEKLNLMDEATIKETQIHAKINELFEGRDQGIAHKIARKCLKKARSITQKCEKCFSLYVCIAESVHKLQGHEEHVREETEEIEETEEQI

>SinvOBP20

MACFLKKQNLMEGTNIKDEVIARLNEVVVTDDVEEKLRTIVRKCIKEKKDITQECDKCFPIYVCIIKAVNEERKCMQEENVRTEEEETGEPNKKK

>SinvOBP21

NETFVLCACVFVLAVYFQSSNSSELNEQELRKIGISIRNDFNTCFSEIGITPADFVKPMEIVTNVHLQPANEERTNKHGCFIACVLKKQNLIEGTKIKEEQVYERLQLIFDENPGGPMHQIVQKCMEKVRNDAQECEKCFSVYVCTIKDMYEEEQRRKNERN

>SinvOBP22

MYKEEELMTKVHTESVNAERTRKVGCFVACAMEKLNLMDEATIKETQIHEKINELFEGRDQGIAHKIARKCLKKARSITQKCEKCFSLYVCIAESVHKLQGHEEHVREETEEIEETEEQI

>SinvOBP23

NEHLVLCACVLIFALSNATEIQQEIQNEIKLRLDLEACLIENGLNNSGLYSMNEVSINVHTKPGNEERTRKNGCFMACVLKKQNLMEGTNIKEDEVIARLYELTRQDLKVILGKIVRKCLEEKRDITQECAKCFSIFECIIQTMDKFPREHEHEEIVTTE

>TcasOBP1

MKTVAVLLFLALAACTKQEDDDRQETIRQYRDDCIAETKVDPALIDRADNGDFTDDAKLQCFSKCFYQKAGFVSETGDLLFDVIKDKIPKEANREKALAIIDKCKELKGADSCETVYLVHKCYFLHSYGTDKKTE-

>TcasOBP2

MKAFIVLVAVAVCAQALTDEQKEKIKNYHKECSAVSGVSQDVITKARKGEFIEDPKFKEHLFCFSKKAGFQNEAGDFQEEVIRKKLNAELNDLDATNKLIAKCAVKKDSPQQTAFETIKCYYENTPTHVSLA-

>TcasOBP3

MKFLVVISTVLMANIVQGLTDEQKSKLEEYSKECLKESKVDESVLKEAEKGVYLDDPKLMNHVYCLVKKINSQKDKGELEVTQIKEKLMMQINDEKEVDKLIQLCLVQEKSARYSLGKCEVSS-

>TcasOBP4

MRASAVFLSSFIISIQAAAFNNPEDELRRSAACLEQSKVSSESIKNLQIGNFDDDERLKEYLFCVSKNAGYQDPAGHLQHEMIRLRFKGGRYSDDTINEVLQQCGHQKDTPQETAFQFMKCAYQNAFPRNYK-

>TcasOBP5

MKTIICFVFVLAGAWFCFQALTKEQIDKLEPISKECRELNGISEDTILKVRRGEAVNEPKLKNHVLCVSKKTGLASETGETNVEVLRTKLRKVSENDDEVNSIIQKCVVKKSTPEETAFEIFVCLRKVKPNFSPAN-

>TcasOBP6

MNFVCVIFILVAIIGAHGLSEQQTEKLNQLSKECRALTGVSQETITNARNGNFEEDPKLKLQVLCIGKKVGIMNESSQIDENVLKAKLRKVSDNDEEVNKIYNKCAVKKPAPEETAFETIKCVMKNKPKFSPVE-

>TcasOBP7

MKFLVCLLFVIVAANALTKEQKEKLDKISKECKNQSGVSQELIDKARTGELINDPKLKAQIYCVSKKAGLATEAGEINMDNLKTKLKKVAANDDEVNKIIQKCVVKKPTPEETAFEVYKCLHANKPNFSVVD-

>TcasOBP8

MENRLVLLIVINTLLLAQAAAKQDFHKKCLASSGANADTIAKVRNGKFSNDPQTQKYFGCMLRSVGVVNQAGQLQVAALRKQVPKDMKRDEAMKIYMSCKDKKGANNDETAYLLYKCFWEASPRHVKIDGQ-

>TcasOBP9

MKYLLFLTVITLTCGIFAFSLSNREQAIFLSTYSTCLETSKVDSERALRTASGIIDDEPKLKEFLFCINKQNGVQDDAGNFVKDAVRKRIEHPLLTDKTMEIIVNKCTRKRETGEETAYQFLKCSYFTIMNEKHQ-

>TcasOBP10

MNIYTCLVLVVIAASAQHLTEEQKNNWRKWSNECKVLIGVSQEAINKIRNNEFDSVDDKIKKHGLCFAKKASLADSSGNIIINQIKIKLKRVIEDDEEVDRIVTKCTIRKNTPEETTFETFRCLRENSSKFVPV-

>TcasOBP11

MKQIYCLITVVVLIPTLTALENEGQNPDTANCVALGGQRIKDSEIAKMAHCILTKTNLMTDKGTFNSNLLKERLRQSVHSDELVDKVVMMCTVEKETPLKSAFSGYKCLRYLVPWFPLD-

>TcasOBP12

MKIVLCLLALATVALAKKCFLAEDTDKLEVMINECKTKTGVPDDILQKARNGEKIDDPKLREHALCMMKKSEMMNDAGEMQMDKIRARIKHAVSNEAEGTRIMNECAVKKDTPLATAYEMICCLIRNKNSVDE-

>TcasOBP13

MKVFVCLAVFALVAAAQAETAKEKLRKYSDECKSVSGVSEELLNKVRNHEDVHDPKLDEHGFCILKKAGFMNEAGDILADTIKTKLKENSEHPDTVDALVEKCNEKKDTPQHTASHLFTCLVDKKVHSH-

>TcasOBP14

MKLFILLSLLSVCYARKWFDKDPQDVAKWQKECFEASGVSMESMNKLPNITLSEDPKLGENAFCLLKKLGFISEDGTLLIEKLRTSLKNQWGDEIANKLVNECARQKSTPQETAHEMFLCIPAKLK-

>TcasOBP15

MKIVLICVLIGLVVAKQQKQDTLDEEKEKMKKWTQECIQESGVTSEILQQLRNQKRVEDPKLKEYTFCTFKKNGFMNEDGKLQYDVIKSTLMKVSGSEEEANKVVKDCVVEKSTPQDTAFETVDCWYRYKKN-

>TcasOBP16

MIRYYIVLLLYFFAPPGISEEMQELVNQLHSTCVAETGVSEDLINKVNSDKVMIDDEKLKCYIKCLLTETGCISDDGVVDVEATIALLPEDMKAKTTPVIRSCGAKMGANPCESAWLTHKCYLETSPADYVLI-

>TcasOBP17

MSPLLLIFISCLFPRGISEEMQELANTLHATCVDETGVSEDAIESARKGNFAPDDKLKCYMKCIMEQMACIDDEGIIDVEATIAVLPEEYQAKAEPIVRKCGTKIGANACDNAFLTNKCWYEEDPEVSLQLN-

>TcasOBP18

MYKTRVIYVLFALCLVEIFVSRAIEMDDDMKELINNLHNTCTGETGATDDQIENARKGNFAEDDSFKCYFKCVFDQMGCMTDDGKVDSEAVIAVMPPELADKIASTVRGCTEVGANPCETAWLANKCYQKSNPDVSKVSSNVRSD-

>TcasOBP19

MSRMLPAALFVVLATLTFATAEIVVPDDLKDYINELHDHCLKEMGLTEGDHKNYNIHVKDPKMMCYMKCLMTTSKWMNMDESIQYDFILSSVHPAVKNILLPALDKCRDIPKGTMECEKAYNFNMCLFNADPENWFFI-

>TcasOBP20

MMPLKNLIILIVCPLFVDIPPDLQAEIDGYYDICYKQIGLTKDDLKAYKIGDRDPKIMCFMKCVFVEAKWMDENENLQYDYIKNTIHHSIRHITLPELENCGKKAEGDKCEKSFSFFNCMNKAEPEDWVLIQ-

>TcasOBP21

MMHLKNFVVLVVCPLFVEIPPDLEAEIDEYFEQCFEPNGVTMDDIKAYKMGDKDPKIMCFMRCLFVSGKWMDENENMQYDYIKETIHHAIRHITIPELENCGKEAQTGDKCEKSFNFFMCMNRAEPEV-

>TcasOBP22

MKMCVIFTLLLLVVLASAEEDNVGKIESVEKKCQEKTGVSEESLQKIMRLEEVDDPLVKENALCTLKAYGVMDDDGNIFPDKFEEKLKPEIGADEAKRVAEKCAVKKDSPEETAHQTLWCATEENALTDTSQEQ-

>TcasOBP23

MKISTLVAILVLAGSAVCADEDNLNTENVQSIEEDCQKETGVSDESLQELSETGDSDDPLVKKNALCILKAYGVIDDQGEISEDKLEEKLEPDRGKEEAEKVAKSCAVKKDSPEETAHEALLCMQQKSQK-

>TcasOBP24

MKSFVIFVLIIVITGQINATPSLDDFKKVQKDCQKKTGVSDESINKVNNLEPVCDDLLLQENALCILKTYEVMDEEGKICPDKLMEVLEPKFGKEKAEKLIEKCTLEKDTPQLLAHATLFCLSVQKYVV-

>TcasOBP25

MNSVLFLLVCALLDKEFLMQFLQKIKKVSEDCIAETQATKNDIKTLLEHKIPDSHEGKCMIFCFHKHFQIQNEDGSLNKVAAISLLEPIKDHSQDIYDKVVKIFNTCFDSAERDDDSCIYASNLAECAIRESKSVSVQ-

>TcasOBP26

MKLFILAGILFTGVCAVDQEFVEKFLQKMEKIGEECAEETHATSDDIADLIEQRDPKTHEGKCLIFCYHKKFNTMKEDGSLDKVGSVLALEEVRDADFELYKNILTIFVTCGDKAKIYDDPCETATALTMCGRDEAKAVSWAYFA-

>TcasOBP27

MKSTWFFLLLACSLTCAFLEKMQEFGAQCAEETDATSDDIAELIARKLPPSTHEGKCMIFCMQKKFNMMKENGGIDRAGAIAALKPLQKADPELHQKVLKIFVTCGMRVKPSPDPCDTATELALCGKKEAEAVFCRLGWKTLS-

>TcasOBP28

MKYFVVFASLFLATNALSQDFIDKFVAKVKSIGETCVPETNASKDDISSLLAHKMPDSHEGKCLIFCFHKQFQIQNDDGSINREGAIKALEPLKADDAELYEKVISIFKKCESTPVDGDSCLYAASLAECAVKEGRAVSFWKNTNFKLI-

>TcasOBP29

MKFLLVFLSVAILCTFAMDESFLQQTRDRVKAIVKECVTEEKATDSDFDDIMALKIPTSHEGKCVFFCSHKKFNMQHPDGSINKEGALDTFEVVKDVDAEFHDKVITVYNHCLSTPVDPDPCVYSVNLFQCFMKEAKAVRK-

>TcasOBP30

MKLLITLATLVVATYAIDKEFVQELRQKLRSHVEACAKEVNAGPDDVSAIFAHKLPATHEGKCIFFCMHKLYNAQNEDGSLNMAGALANLELIKDMDPDVYTKVSTSFKNCESAPFDSDPCLYAANLVTCIVKEGRAVSNNFSGFFF-

>TcasOBP31

MQLLVVVLAVCVLGANAGVSIILDPKFLEKLTQEVQAVGTSCGEKEHATADDMIEIMEEKFPPTSHEAKCVVACFYKHYKMMKEDGTFDKDAAVKAFDEIKAQDAEIHAKILKVIDACDAKKQMSDDHCVSAASMAGCVKTEAIAV-

>TcasOBP32

MLILIAVNCHKCIIVSALSLSATVFGQSLSEDEMRENARKLMTSCKDKVGASDADVEALKMHQMPESREGFCMLECVFDSAKIMQDGKFSKSGMIEGFKPLIGDDKAKLESLEKLSATCESELGDGEDKCETAKRLVECVIKNGKTHGFEVPPPRE-

>TcasOBP33

MNFFAICLCFVASTVGVSSEENDINEIRSVEENCQKQTGVSVEKVNNFELVDDPLVKENALCILKAYGIMDEDGNIYEDKLKEQITSELGEKNAEQVAKKCTIKKESPQETAHESLWCVGEQKPIPGASPDEKN-

>TcasOBP34

MSKTVFIFVIFFYLDFYATGDESVYLSNHEACVKLSGVDETLLETIYEGDVFEDMKFKTYIHCFFKKSGFQDENGVMHFDAIKSSFHKDFSQTENIDKTITECEEKKLNGESALETAFLHFKCFMGEL-

>TcasOBP35

MKAILLLLVATLSFYHMSEAQMKAALKLVRNVCQPKTKATNEQIEAMHTGNWDLDKNGKCYMWCILNMYKLIGKDNSFDWEAGIATLKAQAPESVRDPAIASVNNCKDAVKTTSDKCEAAYEIAHCMYLDNPEKYFLP-

>TcasOBP36

MKYFPHLCLCLIFFELSEAAMSEAQLKAAVKLVRNMCQPKSKATNEDIEKMHHGDWNIDRTAMCYMHCALNSNKLITKENVFNRDYAITLAEKNLPTALKTASIEAANLCKDSAKTLDDKCVAAYEISKCLYESNPEKYFLP-

>TcasOBP37

MILKASIFLILAVATFGAILEDSELMKVVENCVKKTNANESEFSSPNFLETTPSQPALCTAKCLLESLEIVNSEGNINMETLKEYAQPFESPAREAVATCGEEIKSVTTCDDMEKYRKCVEPLIKNS-

>TcasOBP38

MAKKQLVLFFLAFIFLQKFAEVREECLSENSMTMDELHEGWKMENLPESHLCFLKCLLEKREVIDENGVPQKEKIDEILTVKQLSDEKREEISTCITNVEKIENCETMSEIMRCFPKKRRD-

>TcasOBP39

MAKMCRLFVVLSLFVASQALDLMADKNFVELRNKCLDKLGLKEEDLRDLKFDGDVSEDLMCFGKCIQEEDGLLDSEGNLNEEKLEKKIETMPFLSRVSDDTKNNIMECLKEIGKIETCQDFGKQRDCIHKYV-

>TcasOBP40

MNPITSVILTFLFVFSFGEKESEEAQIFTELDGPAAELRDQCLEKNSMKVTDLKTYNTSNDIPEKELCFYKCFYEGVEFIDANGNLNVNNMKEIPAISELGDEVLNEITACVEKIGKIRCCGDLRKIEQCYQNITM-

>TcasOBP41

MWSFVTLLFSFLVLASAQGKYWTTISECLTEHSMGVEDMKKFDLPAEKMSEEMLCFNKCFYDKLLITDENGEINTDNLMSIPLVNAIDASKHDDLVTCLKKVGKIEECDGVKKIEQCFVEFI-

>TcasOBP42

MATRFCFGLLILFVGTVLVHEILEVRALCMNETGVSEETARNYKPAEDPASEEILCMVKCIFEKIGCLKDDGSFCVDTMKKKNYIMDVINEENEEKIYECLRGVGKITNCRDMAAVEECFVKNDSK-

>TcasOBP43

MSFLILLICVIPAIFCRSFSHDELDTDLSFIKTCNRTSPISMSKFGLFLTEFNLTEPGTMNECFLHCLFMKYGWMDSDGGFLLHDIKQTLEESDVEIASLEFILYKCTATESNNRCERAFVFTQCFWDKMAEQQPSEDQFFYNIEDKK-

>TcasOBP44

MKKTILLCLLSQLLLLKAAELQPEDRHQIALQCIDIVGIDQKVVEDAINIEIPKNNPKYKEFLACSYKKQGYQNENGEILMENIKKFLQKFYHPSDLQELNSCSGHNGTNHAENAYQALQCIYNRLSNMTVVGN-

>TcasOBP45

MKPIFAIITLTLCTTVHSAIRPWRTCGTWPPCPPNGKMLQNFRIKRASVRLTNTETNETTPEPKAVSSEAQATENCIIQCIFDNLQMTDSTGYPVHTKILDGLLKNTTNRELRDFLQDTTDECFQVMDKEDTMDPCSYSNKLVTCLAEKGRSNCADWPVGELPFKP-

>TcasOBP46

MLWRRGRFLYGENLDMFDPAGLQACMKKLSVGETELAKALEDKSKDPPEKIMCLFKCALEDSGFLQDGVVDKSKWPMPECVQDVVKITNCNDMVALKHCFD-

>TprePBP1

MSVSTHLPIYVFFCSFVVLSSVANVRSAEAPKEIQGLIAGVREKCHRETGVDIEHVERTTDGHFHESEVLGCYFSCVFNSFDLLDHDGHMDFDKLLKKLPAVESFADHGAAMVAACRHITGANPCESAFKIMQCWQSTYPDKYFVI

>TprePBP2

MKSERFLFLLVVLCARGSESAEPPKEIKVLLEIVREKCHRETGVDIEHVEKTMDGYFHDSEVLGCYFSCVFNSFDLLDHEGHMDFDKMLTKLSAVQSFTHAPEIVAACRHITGPNPCRSALNIMQCFHKANPDRYFLI

>TprePBP3

MTVRPRPRLGLGLLLGCYAISLVYAGTRPSFVSDKMIETASTVVNACQIQTGVTTADIESVRDGQWPESQELKCYMYCLWEQFGLVDEKNELSLNGMLTFFQRIPAYRNEVQNAINECKALGKYFATGDSCEYAYTFNKCYAERSPRTYYLF

>TpreOBP1

MKFAVFTCLMVLLVVQHYPLVQCKKMNIEELKGFTKPLTKTCKTKTGISEATLAQIAKREFPPDPVLKCYFRCIAQMGKMMDKKGNLILENMIKQVELLIVDDISPRVKSVFTECFGEMTAEESCQLAFDFIMCIERIDQELNIIV

>TpreOBP2

MQPARVFSALAAILTFQALVVYAKRPEYITDEIMDMISNDKNRCMAEYGTTEALIDQVNDGHIPNDRAITCYMYCMFESFSLVDEDGEIEIEMLVGFIPEEFQEIAAELIEACATLPGEDVCDKMYKRSSCVQAKRPDLWFMV

>TpreOBP3

MKYLAVILAFCLAGAYAGLSDEQKAKLVEHRKVCVAETGLDPVVVENIKKGQPVQFDEKLSCFAACMLKRIGIMRPDGSMDEQVARAKLPKDLPKDKVDAVINSCKTQVGRNQCETGGKVLGCLLKTKAVSILA

>TpreOBP4

MKTTLVFLAVCLAVTFASTLKDEQKAKLREFKEACIKESGVDAAVVDGIVKGGPITRGDKIDCFSACMLKKIGIMKPDGAIDVEAARGKVKTTNADPDKANKVIDACKDLVGKDACETGGNVFSCFITKKDFPVLD

>TpreOBP5

MQKIALCLAIFLVTYRVEAANEVPAEIRDLIAGVREKCHRETGVDIEHVDRTVEGYFHPSETLGCYFSCVFNQFNLLDHDGHLNFDEVLKRLEGLESFKEHGTEMIEKCRHLTGKNPCDSAFNLVQCFQQTNPEKFFVI

>TpreOBP6

MYLIVGLVLVSCILHVHANEVPMEIKNLVAGIREKCHRETGVDIEHVDRTVEGYFHESEVLGCYFSCILNSFDLLDHDGHIDFDKLVVRLKGTDSFREHGMEMVAACRGTTGKNPCDSAFKVFQCFQKTNPAKYFVI

>TpreOBP7

MKIKLATCIIILINLSAIDSKMTIEQLKNTMKPFKNTCLKKVADVDPVMVEGTKQGNFPDDPTLKCFFKCTLQMLKVLKNGELSVPAMMNQIDIMMSEELVDKTKAIVVDCDGKSKNLGDICERSFAFVKCFYEADSELYFFP

>TpreOBP8

MKLKVSGSIIFILSIYLLNVQCAKMSLDELKKMVKPISSTCQKKNNVPQDLLLASYSGVFAREKSLMCYYRCLATMLKLMNKQGQFALDKMFTQVDLLVVEELAPRIKEIAKICFDSTPKIDDPCEYTYDLVVCAYNIDSSLSVLSR

>TpreOBP9

MKLFIEIFILAVAAFCLVTAGRPDFVTDEILEMVAGDKARCMNEHGTTESMIDAVNEGNIMNDRAITCYMYCLFEAFSLVDEDGILEVEMLVGFLPENMQASAETIVNSCIDESPGDVCDKMYATAKCIYDKRPDLWFML

**5. ORs**

>AmelOR1

MRSSNIDDLPLNDRYESDIHYTFQFCHWILKPLGIYYFIYNQANKFEKILSMVLILICFFIIQFVIVPFGYYILFYEKDMNTKIKFLGPLTFCLSALFKYSYLGIKSSELGHCIKHVEKDWKMLQNEDHRVIMSRYVIMGRNLITLCAAFMYTGGLSYHTIMPLLSKRKVENFTIRPLTYPGYEAFLNIQKSPTYEIIYCMHCIYVIVVGNITMAAYSLTTIFITHACGQIKIQMLRLENLKNEKKVLETGIESHLAVVVKNHVEILRFAKNVETTLRELFLVEVIVSTLLMCLLEYYCMVEWETSDSAAILTYVILLFSFTFNILIFCYVGELLLGQGSEIATALYEIEWYNLPGRKARDIILLLVISKYPPKLTAGKIFILSMNTFSVVSLKIISSLFEHVANDH

>AmelOR2

MARIRNAREGINHTLWFAYPLSKMVGCWPLNIPSSTFSKIFNAFIIFISYLLSLIVLVPGLLYLFLKEKNGRRKIKMLMPLMSTIAQMTKYTILLRRMKEFNKLLDEIKKDWSTATQENRQIFSAKASIEHKLTTVIAITIYGGGIFYRMILPLSKGRIVLPNNVTIRLLPCPGYFGSLNVQITPNYEIIFTLQILGGFVIYTALCGVKSSCLMLCMHMCGLLRILTNKVMELTSDKDEKVVQEKIVYIVQYQTRIKEFYNYVDQFVPYVYFIEMIVGVLITCVLGYCIIVEWEDSDAMAIIAYVVLQVTCVFGTFSICYAGQLLVDESENVRQACNTLKWYRLPTKKARSLILLIIMSNYPLKVTAGRIVDVSLVTFTSIIKSAVGYMNILQQIT

>AmelOR3

MVQIRNAKEGLRHTFWFAYPFSRMLGHWPLSVSSSAFSKILNSFIIFISYLLQMIVVIPSLLYVILKEKNPKKKIKLLMPHLNSIVQMIKYTILLRQMKLIDKLLDEIKKDWSIATEENRRIFSRTASVEHKLTSIIAITIYSGGFFYRMILPFSKNKIVSNNMTIRLLPCPGYFGFLDEQVSPNYEIIFILQVFGGFVIYTAVCSTKSICLMLCMHMCGLLKILTNKVMELTNDNDERVVQEKIVHIVEYQMKIKEFLKQIDQFVPTIYLFEVFIQVLIMCIIGYCIIMEWKESNGMGLITYVIVQMTCLIGSFSVCYVGQLLIDESENIRQAFIALKWYQLPVKKSRSLILLIIISNYPIKVTAGKIIDLSLVTFITIIKTAVSYMNMLQQIT

>AmelOR4

MKFKQQGLIADLMPNINLMKATGHFMFNYYTDSSTKHIHKIYCIVHLVLILMQFGFCGINLMMESEDVDDLTANTITMLFFTHSVVKLVYFAVRSKLFYRTLGIWNNPNSHPLFAESNARYHQIAVKKMRILLLAVIGTTVLSAISWTTITFIGDSVKKVIDPVTNETTYVEIPRLMVRSWYPYDPSHGMAHILTLIFQFYWLIFCMADANLLDVLFCSWLLFACEQIQHLKNIMKPLMEFSATLDTVVPNSGELFKAGSAEQPKEQEPLPPVTPPQGENMLDMDLRGIYSNRTDFTTTFRPTAGMTFNGGVGPNGLTKKQEMLVRSAIKYWVERHKHIVRLVTAIGDAYGVALLLHMLTTTITLTLLAYQATKIHAVDTYAASVVGYLLYSLGQVFMLCIFGNRLIEESSSVMEAAYSCHWYDGSEEAKTFVQIVCQQCQKAMSISGAKFFTVSLDLFASVLGAMVTYFMVLVQLK

>AmelOR5

MVQIRNAKEGLKHTFWFAYPFSRTLGYWPLVSPSAFTKFFNSFTIFTLYFLELIVLIPGLLYVLQVKNPRTKIKLLMPHLNSIAQMAKYTIILQRAKEFSKLLDEIKKDWLLATEENRQIFSERASIEHKLTTVIVVTMYGGGFFYRTILPLSKGKILLPNNMTVRLLPCPSYFGSLNEQATPNYEIIFTLQVLGGFIIYTVLCGTKSACLMLCLHMCGLLKILTNKVMDLTNDSDEQVVQEKIVHIVEYQTRIKEFLNQLDQFVPAIYLIEVVIQVLIICIIGYCIIMEWEDSNAMAMVIYVVFQVTCVIGTFSVCYVGQLLLDESENIRQAYNTLNWYRLPVKKARSLILLILMSHYPIKVTAGRIMDLSLVTFTSIIKSAVGYMNMLRTVT

>AmelOR6

MARIRNAKDGIRHTFWFAYPFSRMLGYWPLSVSSSAFAKISNYFIIFLSYLLTLIFMVPGLLYIFLKVKNGRSRIKLLMSHINGIVQMAKYTILLRKTKEIAKLLDEIKKDWMTASEENRQIFSTRASIEHKLTMVVVVTMYGGGFFYRAILPLSKGKIVLSNNVTIRLLPCPGYFGFLDEQVSPNYEIIFTLQVLGGFVIYTAVCGTKSICLMLCLHMCGLLKILTNKVMELTNDKDEKVVQEKIAHIVDYQTRIIEFLNDLNQFVPSVYFFEIILEVLIICIIGYCLITEWEDNNTMATVIFVIFQITCFIGTFAVCYAGQLLVDESENVRQACSTLNWYRLPVKKARSLILLILMSNYPIKVTAGRIVDVSLVTFTSIIKNSVGYMNILQQVT

>AmelOR7

MNMEHFIVEKKSYNASYKNDLFFNVQLNVWTLRTIGTWPKSLDRSWLETIEHVCLCFLNYVLLAFILIPGVMYFLLEMKDFYDQMKLGSALSFFLMAVMKMCVFIIRENDIRKCIECIEDDWKNVKYQEDRKIMLENASFSRRLIVICGAFMYGGVVFYYIALPFTRAKVVEEGGNLTYRRLVYPFPKALLDARRTPANELLYTIQLLSGFVAHNITVAACGLAALLAMHACGQLQILMSWLEKLVDGRENDDENLDQRLVNIVEQHVRIINFITLTEDLLREISLVEVVGCTINICFLGYYSMMEWDTEHLIRGMTYIILLTSVTFNIFIFCYIGELLAEQTVKVGEKFYMIDWYRMPWKKSLAISLIISISRSTTKITAGNIIELSISSFGAIIKTSFAYLNILRTLTS

>AmelOR8

MMNQLNEQSVLMPVSYARDYEYSIQVNRWLLKPIGAWPNLTKATRTEKLLVKLLNFICHSLIIFTVMPCIMYIFYEDESLKTRMKAIGPTSHWLMGELNYCCLLMRAKEIVYCIEHIKYDWKTVRRARDRELMIKNAKLGRFIACIAALCMHSGIMSYTVITGFKKITFQIGNDSYSMYRLPCPFYTNLLDVRFSPMNEIVFALQLLSGFISTSVTVGACGLAAVLAMHACGQFNVVMIRSDKLVKDNNEKKQDEQTLHKKLGFIVEHHLRTLSLVWYMEKVMNMICLVELVGCTMNMCILKYYFLTEKSKTILGIYAIVYASMVFNIFIFCYIAEIVTEQGKKVGEKFYMTEWYQLPHKTALGLVLIISRSSMVIKITAGKLIQISIATFAAVFKASFAYLNMIRTIAM

>AmelOR9

MMNQTAITEEIKTNSDYSLQLNRWFLKPIGAWPLFSTTTKFEKTVSLILNIICYAIVILCATPSLMQIILAEESFYLKLKTLGPVSHWFVSTVNYTALLMKSKDIRYCFEHMEADWQTIKRMEDQQTMLKNAKFGRYVAASCAIFMQGGILCFCFVTILTTETIQVGNETRVLHVLPCAVYKKLVNVEENSINIFMLCFQFVAAAIANSSTVGIFSLAAVLAAHAYGQLSVVMVWITEFVNQSRNQKKTDDFKEIGIIVERHLRVLNFITYLENIMNRIYFLELFRCTMIICIVGYYILTEWAEKNVQNLTTYFMMLLSICFNIFIICYIGEILTEQCMKIGEVVYMTDWYYLPDKTILNLILIILRSTVVVQITAGKLFNMSIYTFGDVLKTAFAYLNLLRQMT

>AmelOR10

MEKNRSIIGHDDYERNVNLSIRWNRFLLKSLGTWPNLRESRIGKCYSVLIGIVCYGLISFMLTSSNMFLVVEVKDTYNRIKMIGPLSFFAMTLIKYYFLTFHEENIRKGIEHIEWDWKNVKHEEDKRIMIEYANYGKKLALISIFFVYSAFVFYYFVVPISVGKIRDENLTFIPLPFPSSKLIADMRQSPANEILFSVQVLSGVIIHAITATAVSIAAVFAVHACGQMQMLMNWLECLVDGRSDMNKIVDKRIAKIVVQHDRILKFLALTERALQQISFVEFLGCTMNMCLLGYYLIVEWNPKEISLSLTYISLLISFTFNIFIFCYIGDLVAEQCQKVGEMTYMIEWYRLTGKKKLCCVLIIAMSNSSIKFTAGNMVELSIYTFSDVVKTSVAFLNMLRALT

>AmelOR11

MLVLKDSSSVSYSKDWIYSVQINRWLLKAIGIWPLSLCVTTTEKIHSVILTLISIFLIGFLLVPCTLCTLLDKTGDLDTKIKMIGPFSFCIMAAIKYYVLLSRGSHIGKCIEDIRVDWFRVSSHNCLEDRKIMMENARIGRSLAIFCAGFMYSGGFFYTTVMPLCTKRTEIIDNEIVRSQAFPIYRGLLDPRTSPSFEIVQLMQCLAGFVIYSVTVGSCSLAAIFVMHACGQFQILVTKLRRLIDGLKGDKDMENIVHEQRLGNIVEHHLHILGFISQIEELLNEICFVEFIGCTLNICFLGYFLLKEWEQSETIGILTYCILLISFIFNIFILCYIGEILSEECKSIGLSAYMIDWHRLPGKKALSLILISAASNSSTKLTAGKLVELSLSSFCSVLKSSLAYLSLLRTLTT

>AmelOR12

MMADDIATVQKEFENLNEYSIQFNKWFSKTIGVWPLPSSTSKFEKIMTRILILFCWIIALFDAISGLLHFVLVKEDIIIKLKSLAPISYIFGGGLNYAVLLLRKDDILYCIEHMETDWKTITRMTDRQIMLKNAKIGRIISCCILAFMQVSAVCFCTVLGVFKRTIKIGNESMEIYVLPSPIYKIPVDTNPGHDIVLGFQYLAAYITSATVVSAFSFATVFACHASGQLTIMIIWIKEFINRPQKENKNRIDEISVIIEHHMRILSFLERAEHLLSPICFMEMFKNILSICLFSYCILAEWSEHNIRILGTYIFAVINITLNTFLICYIGEVLTERCKKIGNMVYMTNWYRLPKKDILNLIMIITRSSVEYKITAGKIIDMSVITFGNIIKTVFGYLNILRQTTML

>AmelOR13

MADDIVAIQKKFGSLNEYSIQVNRWLSKTIGVWPFTSTTSKFEKIMTKILIIVCSIIALFVTIPSMLHFILVKEDIITKLKMTGPIIYCIGGGLNYAILLFLRDDIRYCIEHIEADWKTITRTGDRQVMFKNAKIGRIISGCIGSFLQFSTISYCTVFGVFKQTIKIGNESMEIHVLPFPTYKIPVDTNLEHGIVLGFQYLTACIMTATIIIAFSLATVFACHAVGQLTIMVTWIEEFVNRPQEEKKNMRINEISVIIEHHLRILSFLERTEHLLNPIYFMEMFKNILTTCMLSYCILVEWSGHDIKVLSAYSFTITNIILSLFLICYISEVLNEKCKEIGNIVYMTNWYRLSDKDILNLIMIIIRSSVEYKMTAGKIIDMSVITFSNIIKTIFAYLNILRQVTIL

>AmelOR14

MTNDINVAKQRSDNLSEYSIKLSRWYLKPLGAWPASSSTTKMERIISQILIVICWCIILFTVIPGILYILFVKQDIYVKLKIFGPLSHWCIDGFNYAILLLRKNDILHCIEHLRADWKLITRTQDQQVMLRNAKMGRYIAAFCAIFMQVIIFFTCFILGIFKRSIHIDNKTVELYNLPCPAYKIPFDTDPTIHDIMLGTQFLSAFVVSSSASASFTLATIFTCHVLGQLNIMMIWINEFVDRLQRKENKDNHINKIGVIVEHHLRILSLIARIERITCPIYFMELFKCMMGMCMPSYYFLAEWSERNIQNLTIYVMVALSMSFNILLVCCIGEILREQCKKVGDMVYMTNWYQLPDKDILNLIMIISRSSVEVKITAGKIITMSIYTFGNIVKTVFAYLNMLRQITMM

>AmelOR15

MFDRSYNNSQLKNIHYENDIHYTLQMCQWLLKPIGVWPFVYDRTSRFEQLISIILMATCFSSLLFIILPSGHHIFFVEKDMHLKVKLLGPVGFCLSSTIKYCYLGVKGVFFEQCIKHVKNDWKMVQDPSYRIIMLKYATISRKLIIMCAVFLYTGGMSYHTVMQFLSKEKDNNNTFKPLTYLGYDPFFDTQSSPIYEIVFCMHCFAAMIMYSVTTVAYSLAAIFVTHICGQIQIQATRLQNLVENKDKKNNCDPFALIVHDHVEILRFSKNVEEALREICLAEIIESTIIMCLLEYYCMTEWQNNDAIAILTYFTLLISFTFNIFIFCYIGEILSEQCSQIGTISYEINWYKLPAKKAHDLILLISISQYPPKLTAGKIIDLSFNTFSSVVKTSVIYLNLLRTVTD

>AmelOR16

MHDRSHDNINGQLKNSHYKSDIHYTLQMCQWLLKPIGVWPLIYNQTSRFEQLISIILMGTCFSSLLFIILPSGHHILFVEKNLHMKVKAFGPAGFCLSSTIKYCYLGLKGSSFERCIEHMRKDWMMVQDPNHRTIMLKYATISRKLITMCAVFLYTGGMSYHTIMQFLSKGKNKDNYTIRPLPYIGYDPFFDTQSSPTYEIVYCIHCFTAMIMYSISTVAYSLTTIFVTHICGQIQVQIARLQDLVESKEKRKYKDCDPFALIVHDHVEVLRFSNNIEEALREICFTEIIECTIDMCMLEYYCIMEWSVGDTITLLTFFTLLISFTFNIFIFCYIGEILTEQCSQIGTVSYEIDWYKLSPKEAYDLILLISISQHPPKLTAGKIIELSLNTFSTVAKTSVVYLNLLRTVTDW

>AmelOR17

MYLSIQNPINEPRNPNYEKDIAYVTKYNKWVLTCIGIWPIILKNINKILPKIVIGINNLLCSFILIQSALHIIYEEKDVLLRLKILGLIFFSFISLMKYWALTIHKPEIKYCIEQVQSDWKQVEMENDRELMLKYGILGRNLTIYSILFMYMGSITYMSITQYAMGLQFNEHNQTIRVLIYPTYGYNIQKSPIYEIIYGVQFMCGYVVDTITSGACGLAALFVTHACGQIDIITSRLDDIVAGQFYSKNLNPDIRLMGIIKHHIRILKFSAVVETILQEVFFLEFIGSTFVICLLEYYCIADWEQKNIISLTSYVLLLISLTFNMFLLCYIGDLLIQKSSNIGVAVFMIDWFHLPTKTIQNLILIMAMSNTPAKLTVGRIVDLSLSTFGNVLKTTFVYLNFLQTAVMQ

>AmelOR18

MHLFVRDQTNQPRNLNYEKDIVYVTKHNKWILNSIGIWPTVLKGIDEYLPKIAIALSNLVLSFTVIQCVLHILLEQKDPILRLKILGLTFFSFISLMKYWVLTMRKPKIKLCIEQIQHDWKQVEFERDRKLMLKYGIIGRNLSMYSIVFMYSGGIIYHTVMHYKLGSYVDEYNRTIKLLIYPTYSRLYDVQKSPVYELVYILQCICGYMFDAVTVGACGLAALFATHICGQIDIVMAKLEDLVDGKFSKENSNPNIRLVEIIEHHIKILRFSAMVETVLQEVCFLEFIGSTFVICLLEYYCITDWQQNNTIGLTTYSLLLISLVFNIFLLCYIGNLLIEKSSNIGIVCCMIDWYQLPIKTIQGLILMIAMSNSPAKISAAGIADLSLSTFGSVLKTSFAYLNFIRTTIM

>AmelOR19

MLKKMKTTSNKDFAYAMTPLKFLAWPVGTWPLQVFNTFSIIRATFSTFLLLLMLTILQVELYLDSSNPEYNLDALILINAGILAVTKVICFHVRSLGLVSNFTSAVKDYKELNSEENRVIVRRHAYMGRAACISLIFCSYVGCTLFMIVPIVAGDKEEVINVTEESAMKYPVPFENTLILINMPENMYFLIFIVEYLMLLLTTTGNLGSDSLFFSIVFHLCGQVEILRLEYNKLSNENERTTKHITLLIKRHIYLLKLGDMLNKTISSILIVQLSSSCMLICTTGFEFILALSIGNIVMIVKTFAVICVLLIQLFAYSYVGEYLKTQTEGLGNSIYFCTWYDMPKNVSHNITFIIMRAQHPVLLTAGKFFVINMETYMSILRISMSYLSVLRVMVNS

>AmelOR20

MLISMIAIFIPTTFEIYVSIHDKNTDAVMECLPNLCASLSSVVKILNVHFNRENFNKLLEFVVKEWDELKLNELHILEEITIQGSKIAHLYRNTLLSFLILFLLVPMYFPILDMIDALNQTRSRQQLLRVNYMVFNADDYFFYVYLQLAWGAIVIVMIVITVDSLYIIIIHHVCGLFAVCSYEIQKTVKDLTVFTDIEKCSYKELKNCVIKHKKAIKFYNILNNSSQLSYLLQIGINIMGISTTAFQLAVNLDTRPQEAIRNAVFCGANQFHLFVLSLPGQILLDHCAELSNTIYCSMWYKLPVKIQKMFNIMLMRSKKSCALTVYGLYELNMENFGTTFKACISYFTMMLSLK

>BmorOR1

MLLSFKDDSRSPDIQKPQNFQYMKILRFNLKIICAWPEKQLNEIRSLGHSIHRVILPIQSVVCLACGILYIHFHFNEIPFFILASTFITVMMNLVTCSRTALVMLFERYLVLTGRFITVMHLFNFQKNSDYAYKLCTFVNRMSHFYTLYVLFSMFMGLGLFNLLPLYNNYVSGAFSDPYGPNVTFFHSVYFAFPFDYSHNFRGYIIMALFNSYVSVTCSIGLVMFDLLMCLMVMHVWGHLKILSHNLINFPRPKASHVITTPNGPTNVETYTEEESKEVFARLRECIKHYGTVDDFANDMSETFGVILLVYYGFHQVSLCMLLLECSDLSTKAMLRYGPLTLIMIQQLIQISIIFELLGSVADRIPDAVYQLPWECMDVKNRRVVYGFLRRTQNPVRFKAMGMLDVGVQTMASILKTSISYFVMLRTVAT-

>BmorOR2

MMTKVKTQGLVTDLMPCIRLLQAAGHFLFNYHADTSGMNMLLRKIYSSAHAVLIVVHYICMGINMAQYKDEVNELTANTITVLFFAHSIIKLAFFAFNSKSFYRTLAVWNQSNSHPLFTESDARYHQISLSKMRRLLYFICGMTVFSVISWVTLTFFGESVRMIASKETNETLTEPAPRLPLKAWYPFKTMSGGGYVFAFIYQIYFLLFSMALANLLDVIFCSWLIFACEQLQHLKAIMKPLMELSAALDTYRPNTAELFRVSSTDKTEKVPDAVDMDIRGIYSTQQDFGMTLRGAGGKLQNFNAENNPNGLTAKQEMLARSAIKYWVERHKHVVRLVASIGDTYGTALLFHMLVSTITLTLLAYQATKINGINVYAFSTIGYLVYTLGQVFHFCIFGNRLIEESSSVMEAAYSCQWYDGSEEAKTFVQIVCQQCQKAMTISGAKFFNVSLDLFASVLGAVVTYFMVLIQLK-

>BmorOR3

MIFVDDAVIGIKDPREYRHLRVLRTSLRLLGAWPGHYLGEETGSKYECAPMFLLMFIKIACLYLTIVYLRNNADVLGFFELGHVYLTIFMTFVTLSRGFSLTWNPNYHKVVKKFITEMHLLYFKDNSEYAMKTHRRVHKISHFYTVFLKVQMIAGLTLFNVIPMYNNYRQGNYASDRPANITYDLSIYYETFDILNTPNGYIFICVFNWFASYICCSFFCSFDLILSLMISTVSGHFRILIHNLLTFPLPEAITASKKFVDKHRCNGNRSEFVLEEAKLYSPAEMWQVTDRLRQCIDYHRKLVEFTGDISEAFGPMLFVYYLFHQVSGCLLLLECSQLNTAALVRYGVLTVVLYQQLIQLSVIVESVGTVTGRLKDAVYEVPWEYMDTSNRKTVAIFLMNVQEPLHVNALGLAKVGVQSMAAILKTSFSYFTFLRTVSE-

>BmorOR4

MFKIIKNIIVENDALKQVEKPQEFQYMKWVQYHLKYIDGWPNMDMNKKNVSKIRFHKRHLLVVEQTITFLSQMFYIVKNYGKLSFFEIGHSYITALMTIVIFSRSVVTALGRYRKIARYFVSSLHLYHYKDISEYALQTHLLVHRLSHYYTVYLISLVVTGMLLFNITPLYNNISSGVFNSPRPENMTFQHAVYLGLPFDYTTDIKGYFVVFILNWHLSHIAASYFCTFDLFLSLLILHLWGHLRIILNNLKTFPKPYTNNSMYTEEENQVVLLKLQECIRYHNFIISFTVMMSNVYDVVIIVYYLFHQVTGCLLLLQCSTLDWESLSRYGPLTLIIFQQLIQVSMIFEILGFLSDKLPNAVYSIPWEAMNVTNRKLVQVLLQKSQKPIQFKAMNMMSVGVQTMASIIKTSISYFIMLRTIARD-

>BmorOR5

MLLYYPNTQVKEKVNNVEEFTYIKFLKSFCKIMDFWPEREEKNSKTRIFRLRYILVLQFCFTLVAGVLYLTNSVGKQTFYDLGHTIITVLMNVVSLSRLILRCFKKYDVVGQQFINKIHLYHYRNDSEYAMKIHTVVHKISHNMTYIFSFCIIFGTVTFNLTPIFNNIGSDAYKNPRPDNVTLQQCVYYALPFDYTGNFKWYLLVAIFNVQKTFFCTSLFILFELSLSLMIICLWGHLRIFIHNLNHIPAPRNSFEYTKEERQEVDDTLKKCIQHHTLIIGFVRIMSETYGLAVLIYYAFQQVVGCLLLLQCSQMELKTVTRFGFLTLVLNQQLIQISVIFELLGYMSDKLQDAVYCVPWEYMDTSHRKMVYMMFRQSQIPLQLKAMNMLSIGVKTMVSILKTSVTYYLILKTVTTD-

>BmorOR6

MKEEYYLQHPRTQLFYKVLAHVSTIESTIDLTWWGYTFPKYVGWFYHLQCNVVRLFGKCVVVSQILFIILNYQTIDKSVFIIAITITPLGALVGIKAESAKAECYVNLMKNFMDKVHIHSIYRKNENNEFVKKKVIQIERVSRFTAYFLVILIAINCLSWMLKPTLHNIKHFEEIMNKSMEFQYYIYFWTPLDYKYNLRDYIIIHTLCIYLGATAVTVIVTFDIFNFIAVFHVVAHIQILKNNVKSNWSDDFNESEKKGYLVSILEYHAYIIRIFGEVQSAFGLNVASNYLQNLIEDGLFLYQIMNGEKENVLMYGLMIILYLGGLIFLSIVLEEIRRQNYDLCEYVYALPWEGMSLENQKIFVVFLQRTQPDLEFETVCGMKAGVKPAFSIVKSMFSYYVMINSRF-

>BmorOR7

MLLYHPNTQVEEKVNNVEEFTYMKFLKSFCKIMDFWPEREEKNSKTRIFRLRYILVLQFCFTLVAGVLYLKNNFGKKTFYDLGHTIITVVMNVVSVSRLILRCFKKYDVVGQQFINKIHLYHFRNDSEYSMKTYKAVHKISNNMTYIFSFSIFVCVVTFNLNPVFNNIGSGAYKNPRPDNVTLQQCVYYALPFDYTGDFKWYMLVAIFNVQKTFFCTSLFILFDLLLSMMIIHLWGHIRIFIHNLNHIPAPRNSLEYTREERQEVDNTLKKCIQHHTLIIGFVRIMSETYGLAVLIYYAFQQVVGCLLLLQCSRLDLKTITRFGFLTTMVNQQLIQISVIFELLGYMNDKLQEAVYCVPWEYMDTSHRKMVYMMFRQSQIPLQLKAMNMLSIGVKTMASILKTSVTYYLMLKTITANEA-

>BmorOR8

MSLSTRCLLKDFCKYVYYAGAGNFWYEDIYKETVPYKMYVVISFFTYTVMIFLENLAALFGKLPEVEKNSAVMFAAIHNIVLTKMFLLLYHKRSISKLNCEMAAVGENLEEASIMRRQFRKMRLGTALYFISVYLSLVAYGVESARRTIVEGAPFYTVVTYLPDYDNTTVLASFLRIFFYITWLYMMLPMMSADCMPIAHLITMTYKFVTLCRHFDQIREKFQINVKIMAKTEATEILKLGFIEGIKMHQKLMYLADEIHRVFGIIMALQVCESSAVAVLLLLRLALSPHLDLTNAFMTYTFVCSLFLLLALNLWNAGELTYQASLLSNAMFYSGWYFCDFEKDWCRDIRRLVLIGCAQAQKPLILKAFGVLDLSYETFVSVARMTYSVFAVFYKRGD-

>BmorOR9

MLALDDPLQNVDNVEDFKYVKWLRNHLKTVDAWPVYSKSKRKIQKRYVLPIFSAACFISQTVYLKNGIGTLSFVVLVHSYICFLINGSCLCRGILIATERCKRLATCYLKTVHLFHHKNRSEHAMKIHVIVHRLSHYYTIYLISLVFVGMVLFNFMPIYNNINSGAFKSPRPESVTFQHAMYLALPFDYTTNIKGYFVVFILNWYISLVTTSHFCTFDLFISLMIIHLWGHIKILMCSLEDIEGFVLGGSFKFTIEQNRKINSILQECIRHHQFTIDFTNEMSSTFGLVILFYYFFYQVSGCLLLLACSQMDIESLSRFGPMTFILFQQLIQLSIVFELISSLSENLPNAVYNVPWESMDKNNRKMIQVLLLQSQKLTRFKATSMMNVGVQAMATILKTSVSYFIMLRTMYQEH-

>BmorOR10

MRTNAKSFLFVPSKVLTLCGVWPVEKTSIFSLIYRSIMLSSQFCFLVFNGIYIGLMWGDLKAVSDALYMFFTQTTCCSKAIGFYFNFMKIKRIVASMDDVLFTAMSIEDQATIFSHSRTVNKLYKGVLGFTGFTLVQWTVLSLIGSGRTLPFNEMWVPTDISKSPNYEITFVVELWMMVISAALFMSVDTITVATMMFSCAQLDIIMKKTQQIQEIPLSPDLSSRNRSELHEKNNGILIDCIKQHQAIVRFSELCEGTFQVHSFFHLGGIVFMICVIGFRMAGESPVSAQFWAALSYLVIILGQLYLYCWCANELTTKSEQLRDKLYLTPWYDQDVKFKRNLCIAMECMAKALTFRAGSYIPLSRAMFVSILRSSYSYFAFLNQANEQ-

>BmorOR11

MDEHSHFETSLNKIKVLFKYSGMNLENTVTNTYEFLNHRWVYILNHAWTLAAVTFICIGISNGQNFIEMTCIAPCVAMTVLAVSKSFFHYINENAVKSLLENLIELERTDFERTKSVQRTEIVATEKQLLNMVINVLYVLNCSMILVFDMTPLIIIAIKYWTTNKFVRLLPYLDIFVFVPYKFEYWVMAYILQIWAECIVLLFIGAADCLFFTCCTYIRIHFRLLQYDFERLTSSRRESDGLRDDEDFRETYTNLVKRHQGLIESSSILEMIYSKSTLSNFVLSSLVICLSAFNVTVVNDVTIVMTYLIFLAMSLMQVYFLCFFDMLMSASEEVGNAVYNCSWYTEKASTGKDLLFTITRAQKPCELTAAHFAYVNLKAFMRVSFTSASITTLPTI-

>BmorOR14

MSNYIFKPFHETYRIITFTMIAAMIYPNPATEKRRLIYIGLMLLSVIPLAFMIVTEMYEFFMASDLNNTIRHSTVIGPFIGGFVKVALMYYKRRQANELVSEINRDHLAYNGLKGEDREIAASSIRNCQIYCELGWTLIVMSCGLSFPVIAILLKIHSFTFKLDSTKHMIHDINNPFTDDPEDRFESPFFEIMFVYTFFSSFIYIINYVGYDGFFGLCINHACLKMKLYCRALEDAMRSDSRRHEKIVAVIEEQRRTYEYIALIQDTFNIWLGLIYVATMIQMCTCMYHIVQSFNIDVRYIIFVISIIHIYLPCRYAANLKCMAAETPTLIYCCGWESVSDLRIKRMMPFMVARSQVIVEITAFNMFAFDMELFVWIMKTSYSMFTLMRS-

>BmorOR15

MMTLVYQTDIFKPNVFFWKMFGIWADRKSSKTYKYYSFVFLFITLIMYNSLLAINLLYTPLKIELLIREVIFCFTEITVSTKVLMILFKRNKILDAFDLLNKNEFRGNSEESSAIIQKNNSAYKTYWKLYAILSNFAYSSQVLGPLIVKLIWKTKLELPICNYYFLNEELRHDFFSGWYIYQSFGMYGHMMYNVNIDTFISGLLMMAVTQLKIIQTKLLSLKLNPRERKMDRGLMNITEVLKLNEILKHYELVLKYCSTVQSILDVAMFVQFGVASAIICVAMCGLIMVRSSTETLLFMVTYLFAMTLQIFVPAWMGTQLHFQSQELVFAAYNSEWIPRCQSFKRSIIIFVERAKIPITITGLKMFPLSLATFTSIMKTAYSFFTLIRNMQALQEE-

>BmorOR16

MSFNSEDLYLNRAKFVMKYLGVWVPPENENFARKFYKIFMMSLQHLFLFFQIIYIVEVWGDLEAVSQASYLLFTQACLCFKITVFQINMNKLKELLKQMNGYVFQPKNINQQNIIKVQATRIKRLLFAFMISSQLTCGMWALKPLFDDVGSRKFPFDMWMPVSPERSPHYHLGYSFQLVTICMSAYMYFGVDSVAFSSVIFGCAQIGVIKDKIMSIKPLGIYRNHKTYTKISRYNRKTLIECVKHHQAVISFTELVEDTYNSYLLFQLVGSVGIICMSALRILVVDWRSVQFFSILCYLSVMISQLFVCCWCGHELSATSEELHTILYNCAWYDQDVKFKRDLNFMMARARRPILLRAGYYISLSRQSFVSILRMSYSYFAVLDQTNK-

>BmorOR17

MREDKMEINNSQKFYTKMIFRYLYSVGLGDWWYQHEDRSDSHRKLYCLWAVISNAYIFLNICNELLANFRKDLTDVEKNDAIQFSFAHPLIFAKIASFFFNRKKIREVFGRLLEENRSVYSCGELEKESMKQIKRYSLAFIGVSYMTLVMSTIDGLRAHFKEGIPIRTEVTYYPSPSNSGVIVNILRFLVEFHWWYIVSVMVAIDSLAVASFVFVTFKFKLLQRYFKDMGLTVRRDQSNMTDEALADKFRRDFIVGVKLHENALWCAENVQKAFGWVYSVQVFETVALLVMCLVKLVTTNHNMIFLLANFAFMLCVIILNGSYMMPAGDVTYEASEVPTSIFLCGWELVRQTDLRFLVVVAIQRSQVPVIMKAFGIMTLSYSNFIAVSLFKFYVQFQINLF-

>BmorOR18

MGDRMVTRGHFFDFNIKYLFYVGLWPSNEAKRIEKIAYKIYEYQLHVLSLIFLVTTGIGTYKNHKDIIALLTNLDKTLVAYNFVFKVIVFVYKREELRKLIEQIVQSGDQITEDRKALMAKLVIVLTGISTVIITAFSCLALFEGEMTIDAWMPFDPMKSKMNLFAASQILAATFVVPCGYRAFAMLGIVCSLILYLRDQLVDLQNKIRDLRFATGNVEKLRDDFKLIVKKHVRLLGYSKVIEMIFKEYFFIQNMAVTAELCLNAMMVSVVGLEQKTLAASFLAFLSVALLNAYIYCYLGNELIVQSEGIAMAAYESSWILWPVDMQKDLLIVITAAQKPMKLSAGGMAVLSVQTYSQTLYNGYSIFAVLNDIVN-

>BmorOR19

MHEFVINVQNETTKLYDQLNIILYILGLQGIWVDEIKLSRRFHVFFKVVTFILHIMCGMFAGLQFFAIFTQNSLNSQQKSDVIVIGISNPMAYIFCINFIRNRNEIKDLFYHLAVVLKIYYNDVEIEKSMVNKIKSYLSTYVFASITILVSNGIIAFFQTINSDEPFLGIITAWPDKTDTSKTASYARIGFYLFWCIHFFRISTVFAVIVCILISIKYQYKILCSYFESLNKIFDDETSSHEVKEAEFENAFCNGIKIHTQIIWCVRRCQIMCRTVFSANIMLDTFVLVILMLAMVNSENDFYGLCSQMSSVLVTVVLMAFFMWTAGDINVQASQLPDAIYGSGWYNCRGKSSARIRSLVTISMNKAQQPILMWALGFVELSHKNFVAIIKSAYSVFSVFY-

>BmorOR20

MIQASKYPNSKTKELFRKIAHIAYICGLPNFWIEELNLPKSFIRVYDKIVRIFNVATYFFLGIEIAAHFTQHHLTNKQKFDLLLYSISHPILNGYGVIVSRQVGNVKKVLLDLIVNLKVKYNDPVIEEAMIKISMTYSVSFITNCVLSMLTYTFDALLMVYKKGVTFNVIITAWPDVEDTTTEASIGRIGFHIFWWLFVTRPFAVYVLVINLTTCLSHQYMNLQSYFFHLEDIFKENLSQNEKEAKYEAEYKIGVMLHANTLRCTRRCHMVWNGVMSGQIIFNISLIVIIMAQMMNSDRTLVNTFGTVLTASAILISTGFFMWNAGDVTVQASRLATAMYCSGWQNCRGKSSVSIRNMVMNTIAVAQRPLVLRGLGVIDLSYQSYLSIVKASYTVFSVIY-

>BmorOR21

MNKNMNKNHYILKTYCDKIFLVGSGNFWYQKTESRNDKTLLYKIYSCVLFFTYGFMTVLEIMAAMMGDFPEDEKRDSVTFATSHTVVMIKFISIIKNKELLKTLNRKMMMICEAHEEQTLMDEMYRTVKINVVAYCVAVYGSATFYVFEGLRKFYNGSHFVTIVTYYPSNDDDTLAATIVRIATTLVLLMMLLTMIISVDTYTMAYLIMYKYKFITLRHYFKRLRENVDELVAAGKARLAAEKLAQGLVEGIKMHNELLSLSKDIDKAFGTVMALQLCQSSGSAVSLLLQIAVTMYLLLALFLCNAGEITYQASLLSDEIFYCGWHKCNSPVLSTQRNIRDIVLIAILRAQSPLVMKAFKMVVRSTYSVFALFYAQNK-

>BmorOR22

MNKNHYILKTYCDKIFLVGSGNFWHQKTESRNDKTLLYKIYSCVLFFTYGFMTVLEIMAATMGDFPEDEKRDSVTFATSHTVVMIKFISIIKNKELLKTLNRKMMMICEAHEEQTLMDEMYRTVKINVVAYCVAVYGSATFYVFEGLRKFYNGSHFVTIVTYYPSNDDDTMLASIVRIATTLVLLMMLLSMIISVDTYTMAYLIMYKYKFITLRHYFKRLRENVDELVAAGKARLAAEKLAQGLVEGIKMHNELLSLSKDIHKAFGTVMALQLCQSSGSAVSLLLQIALSDQLTFTMGMKIFFFLAAMYLLLALFLCNAGEITYQASLLSDEIFYCGWHKCNSPVLSTQRNIRDIVLIAILRAQSPLVMKAFKMVELTYATFILVVRSTYSVFALFYAQNK-

>BmorOR23

MRAKTEFEKTIKLTKTALFLSGINIFLGEWNHWTRTFVDSIAYYLNIVGLYFVLIGEMYWLIDGTITGKSFVELSLIVPCLTISVLATAKVHYLYHNKESLLDVVDKLREIYPDEIEETANDNDQCLNDKKETVYDNDVTEVGIVNEANELLKFVNFLLSTVSFVVTMTFCTMPLFGMAGEFMETGKFVVLYPFAVKYPFDVYNTSFWVIVYVNQFWATIIVCTNIFGVDTLFYALCSYIGMNFRLLSYKFEHLEIKRNDRIINEIIVLIKRHQELIELVNKTQSLYSLSTLFNIVTSSLLICLSGFNITILSRSWSYFALLKTIYS-

>BmorOR24

MPEELFLDRSIKKIESYFRWMGINIRSGDNNNKKDVFKIRCIYFINFVLLNTDVLGAIFWFRSGLEQGKTFTEVTYNAPCLTFSFLANFKMLSLIFYEKTVHELIAALQKLEIKHFLRQNCAEELKMLKDEKNFLHAVFKGSKIVNYASILTFGCSPLVLIASNYYKTGRMDYLLPLIVLYPFDVDNITVWPIIYVRQIWSVITAVIGVCATDYLFYTFCVYISTQFRLLGHSIERVVPNNGLSVRTRLNGNLRMKFVENLKWHQELIRAASLLEQIYTKSTLYNFVTSSVIICLTGFNVAVVEDFAVILSFLFFLFMSLLQIILLCFFGDKLMKSSTNISDAVYNSKWYLTEKNVGKVLLMVQIRSQRACRLTAYGFAEVNLRAFMKILSTAWSYFALLQSLYSSHE-

>BmorOR25

MFEKALRSANFYMRVIGIPTDIRDGNRTLMERLRNRWFYCINFLWLNTDVAGEITWFVKGLLNGSSTLIENTYLIPCLTLCILGNVKTFFTIKYANHIIDLVAILKDLEIKNNAARKNETEIVKERLKFLTTSNKFLLFVIGTGIIAFGIGPLMLTASIYFSSGDMKLKLPFLIWYPFDSSDIRYWPFVYVHQVWSACIACCAVYGPDCFYFTSCTFIHIHFIHLQNDITNVIVESSRARKNGLYRGCHQAFLELTNRHKDLIRCVNLLEIIYSKSTLVNVVSSSLLICVTGFNVMVTFCWFAAPFASFLALGLVQTYLLCYYGDTIMCSSTEVSDAVYNSTWYGTNISQMRDYLFVMKRAQKPCKLTAYGFSDVNLRTFSRILSTAWSYFALLITIYRGNGQQ-

>BmorOR27

MPSSFFLPNLENPDYPSLGPTLKGLKYWGMWQSGGIKRILYNSIHAFATFFVITQYVELWIIRNNVELALRNLSVTMLSTVCVVKAGTFVCWQKYWSGIIGFVSNLEKEQLSKNDAATQAAIVKYIKYSRRVTYFYWSLVTATVFTVILAPLVGFLSSPERELIANGTLPYPEIMSSWVPFDRSRGFGYWVTALVHTLICFYGGGVVANYDSNAVVLMSFFAGQMKLLSINCSRLFDDGNEVISNNEAMKRIKECHYHHVFSTIFNSLMSPVLFLYVIICSLMLCASAVQLTTDGTSNMQRIWISEYLMALIAQLFLYCWHSNQVLYMALEDRLGGLFEACLESGRFPSKWKTGRLVLLRKDGRPADSPAGYRPIVLLDEAGKMLERIVAARIVRHLTETAPDLSAE-

>BmorOR29

MFDFLQNLEDSERPLLGPNFWLINKTGLLLPKTNFGKLAYILVHEIVTFFVVTQYVELYVIRSDLDLVLTNLKISMLSIVCIVKVNTFVFWQTSWREVLEYVNEADKFERNQTDETRGKMIETYTKYCRRLTYFYWSLVFTTFLTTTNTPLMRYWSSPIFRENLRNGTEDFPHIFSSWMPFDKNHSPGSYCTIVWHVLLCAYGAAIMAAYDTCIVVIMVFFGEKLNLLRERCKKMLANDLYNHAFVIGQLHDIHVQLIKQSRLFNSLLSPVMFLYILMCSLMLCASAYQLTSATSTAQKLLMAEYLIFGIAQLFVFCWHGNDVLFKNANVSLGPYESNWWSSSPRVRADVLLLCGQLRVRHVFTAGPFADLTLSTFIKILKGAYSYYTLLRK-

>CfloOR1

MFLVILTLATTSIMLVAVSTFTFKHITSILSLVRSMGMTIAFSTVLIKTVMFLAHREDMDYLNNNLTTQYLADLEIPENRPYMLKRIGLYAYYMYSFSAFVAITMFLYAFGPLIDYLKYGKFTRAVPSIIPFDYKSGGLMHWLFYFTEFYGLSTIWFISSAVDLAYVLYTIQMCGELKLLAKKFKELNLEENYQAQIKDCIDRHNLLIHSKKRIENTFGIIAIWMAISSAVTLCALTFQVSEIAKNKLSIQRIGRLCLSLFPKAAQIFAYGWHGNRIERESARSLDSMYGSHWPENCSKHFKNDITIVLVQKPLILVAKGCMFIQLDMFTKVVRASMSYFCLLRSLTEG

>CfloOR2

MMDASFDKYLGLNVKMLGCMGLAVSLNNRKINYKYFILEKLPTLLTTVLALFELFCRFQSITRSWNNDFSFALQMLSFLLSSFICAFKGFRIAMAINSIRSVLPEMAEMWQKYPPREHRLKARVLSSAARSLLFSKLYFFHVVLGIGIAAPPVLKFFSNCFTSEVMNEACDLTVRVYLVEYPFEINSRLLYFVILVKEEWMFFNSAFYWACCDTLYIQFVTHVILQLKILKFDINDVLKHENDEKQVTVYLIGFIKRHQVILRFCHQIEKIFSPVLFVAMITSSVNICLAIFGVQEIFKASNYNDIATYMFILAGVTYQMLLYCIYADSLTQEAMLVANAAYGSDWVDKNHKLWTYLRVLMLRSQVPVNFTALGFFTISLRRLTMISKGAVSYFAMLSARHS

>CfloOR3

MSVIIRHVEICLKVVGLWHDSTNSFGFYLVILCLISHMPFQLWHAVVVRHNFEALMANLSVLMTISAAVVKLIAFRLKGRNVRYVVKEMMDDWINENHVSNASVMKEQMNRGNYLTKWLIGAYNGVVILYMINVVTNYSWEKIEKRAYIMPAKYPVTWKRSPNYEIVVSTQFIAGLLSNNSQAMIEGLLTILVLHVGTKVHILKRELAIVSEVCQLSKDKKLTSKAMKLMINKHLNFIDFIEKIKDIYFYVSFFHVLIFTLLHFMIAYILLDAFDKGDSSMKIFMYGLFTTRSLFSTMIHCACGEYLINQGKQFVDDLNNVQWYEFSNTSDKRAISFILMKAQKPICLTPAKFGYLSYNYLSGIIRTSYSFLSLVRVSH

>CfloOR4

MQLQPFVLLSLVSIWRPKKWSSSLAVTLYSLYTVVSLIVYYTFMISEILDIILLAENVQQIMENMIQLINITNVTQKSFSFLSKRKKIIRFIDMFFDDVALPQTPEEEAIQKKFDDESRSNTHKLAGLYSISVFTFVYMPYFVSDIEDRVLPYRGWLPYSLDSVRNYRIAYLHQAWAVTIAASVNASTEVLVSGFMIQICAQFRILEERFARLTQTVKAMRDGGIDEGLVFAEEKKLLVKLIQHHLKIFEMAELLNDIYVFVILFQFAASIVVLCVCTYNLALCPSVNSDFVTIFLFQSCMLLQIFLYTWYGNEITLRSTDLGNNILLTDWRHLSQTGIKNLMIIYQRTTKPIIMSSGYVITLSNVAFTSIVKTSYSVYNVLSV

>CfloOR5

MKILPIPLAILTICGFWRPASLEPNFIAKHAYNCFSCFAVFLIYTFTLSHLVGIVISAQDFEGLTGSCFMLLSMLNVCCKITNILYFHRGIAELTNILETGYCVARDSVERDIQLRNHNKARLVTLFYLALTETTCTLITVRTFFLTPHKILPFKAWIPYATEGSITRYWLTFLHQTIAHVGAANVQIANDTLICGLLIHASAQLEVLKHRYRVITEESARSADGDDRLKRLVDCIEHHRCIKLFSAKLNGTFNVIIFFQFVTSSLVLCSSVYLLSRMTLLSVHFMSLSLYLSCMLYQIFLFCWYGNEVILQSSDLVGAMYAMDWTVFGIDDKKNLLRMMILVRKPIKFTSSFLINLSIDSYCKILKTSYSVFNFLQRTSM

>CfloOR6

MKPLYDRYYKLASLFIKYSGLWPDESFQTTYIMFFIFIVSILSILIPEVVMLYGSIGTQITVVLENTLAVIFCIASILKYIIMYNKSSQIKSLFTHMIKDWKYLRDNTELDILHKFCNQGRLMVNFYSVVSVIGLVTLMAPSYVSYVLIKYFSTDTKTYPPLVLVYCDYVFFNQTDYHFSTTTHMAIVQFENLFLETAITSTYVISVKHGCGLLRIVGYRLECIGQFEESEGNYSMDNRKEVKENLKAIMILHNRILRHTKQIDAAFCEIIAILQLLATISMAMTYFYSFCIPNTFVKSLKGAYFMLGLMIYCLYIHLIGQMLVNSSENVFYFSYCNNWYLISPRDRMLLKLLMRSSIEPIQLTAGKIMTLSMESFGKVMKVTATYVTVLTTCTTKGDKNMLELSG

>CfloOR7

MAIDTSINGYDECVSITRFTMKSMGVWPNAKSNFNLCPQILFFFSLFSMVFFIVLPQTMQLFYTEGNLDLILDILTTADIIITIACLKLIGILYNRRDLSYLLTELEKDWKTSTEAEQKAMWKNIKLCRIILISCAVLTYGTVITLTYCIEDNFNQIFLVQMILYIFTICLQGYQIVTILGMKNFKDAAATLVFIVVFTLANILSLFTYCFVAEKLRSESTSIFQAIYNIPWYEYKSTESKMILNIMQATINPFTLSAGKFVELSFQYFAKVLKCSAGYLSMLLAVQKGNNATQ

>CfloOR8

MGFQYAFGVCHTNLKIVGLWPGSINSKFNGIIPVLLLTITIFIMITFVTMAQTTKLLMIWGDLGLMINNLSTANLPITVVIIKTILFYNYKRELGMLLSSVINDWNADKTKGEVINMVRNAKTTRKLSFICLFLGMGSVNGQLAVRISQELDILPGQINKREPMLISYFPYDYKPSPIYEITWLFQYLGAILATLIYSGIYCFFVGLVLHLRGQLANLRFRLETLDIDGNKANHRKITKKIGFIVERHNILNRFAKEIENVFNGIFLVEIISCTVLICLQLFLLVTLISDDNKDINVPILQIVFTVTYVMHVGTHVLISCYVADKLQDESSAISQSAYNIQWYNMASQNSKLLLMIMQRSEKPLKLTAGKFCSFSLLLFIQILKTSGGYLSMLLAVKEKI

>CfloOR9

MVVTSTNANEMLSLSRWSLCVVGVWPDLKSSRQFATISYIIFMTSIVSICNFLIIPQTMKLFLIGNDLDMIVNILCTANIPQIVTLIKMSIFRYDSTEVKSLLHTMYKDWNSEYTSEQVKMIRYSAKVGKRIAAACIGIGYITSNIFFILRLILLQEEKSYSSGHVFLFESHFLIDVSNSPWFEVIWLLQGFCTFSMTNAYSGIDGFFAILVLHLCGQLDVLLDEINMLSIDNAKMDVKENIRSIIKRQNEINAFVKMIQGIFGQIFLIQVSACVVQFCLQGYQMIHIVTNAKVEKPILQLMFMTMYLLYLSLDFYIYCHVAEKLRTKSLEVSQAVYNCKWYDFKTKEAYDIIFIMSRAQHPLEITVGKFCSLSLNLFAIVQQQQQQQQKRVAQCRVNCTSYL

>CfloOR10

MDVLPLNFYSLRLSGLWYEENESFGFAKKVYHFIVVAAVFYFTFTLCAKVVIRDSDIKGPTESLFLALKFISLCLKIINFLYQRDNVIEIIRKMKVPHCKADNAVEAKILAKYAKTSKWLFVFLMSFSVCTVLLFFNALGSKKKSDQLTALKTYQFYNISSPTVFYTTMCFYLIASLYSVIIHVSLDTMSVGLFMVISGQLELNAYRLKKLKCGDVRCLKGCVVHNVLIRGVTKKVESLVIGVVIPFFFFGMGIICISIFEATKYNLFSFDFAWIWVFFLSSLQQVFIYCWFGNQLTLMSQEVSNRIYESDWMSVDPRERKSLLILMICNQKGRSVSCHGLCALNLDTFLWSLVIMSLILCPLPNECTLVVLKILKMSYTMIGEGVGGVGGVGGWGK

>CfloOR11

MDFHKSRYFSINKRMLKCMGLWPYQSSLTKIVIHTCTTVIFIILFIPQICCFSMHIGHNGDKTMQAAAVTIYIFVIFLKSIAVMMAENKTRKMYENIVDNWKLLKNEDEFNTMKEHSEFGKLLTIGYIAYMYSAGTFFVTVPFVPICIDYVFPTNSTRIRIFILDGEYGVDKFDNYGKIYIFESFTCISTTFIFCALDTTYSTCVQQCVGLMDVVKMRLKLATKRDRSNEDGNYHAMRECIILHKNALHFAKVLESSYSTNFLIVMATNVLIVSVGSVVIIMNLNRPLELIRFGLIYIGLFIHMFYLSWPGQRLVNIGNQLYEDTYNNDWYQCSIETQKLLRFMMMRCKEPCHLTAGGLYVMNMANFGSLLKTSMSYITVFSSYR

>CfloOR12

MLLEHQVIATMDKISSFDDYVWPNRYLLNSIGFWPKESNESCISRMLTYFRIILSIFLISTIFLPEIFTMIKYRHNIAIITGSAPRLFKKVVKHILTSLRGDGIKSLGFSIKWGKISLLDVGSVAIEMSQLIFKVYYMLARREKFYKIYKSAKELWSITSDHTERRHLEKLAKLANRASIGLFSVGMANLFTFSTVSTVLTINNYRRSNNTNYVHYFPLNIEYGIIDTQRSSNFGIVLCSQIIAMICSIIGIAGFDSTTMTLILHLSGQFRLVAARFRKLGNIMTLGLLEFQTKVEECISHHQKTLRIANETRELLAPVIFVQLMSSGLEICLSIFMLVRGGSNTEVTKYIIYAVTMFVQLVLWCLPGELIMQDSMSVGNVIYHELPWYRLPVSQQKDLFFVMARARRSCCVTALKFQVMSISKLAEVFNTAASYFALLRSVTDKSV

>CfloOR13

MDGGKKNKGIERKRVKLDDSTRAFQWNKLFMSTLGLWPAKPSDFLFSVTFGYFCYEMVLEYIDLLLFIDDLEHVILNLTENVAFTELLVRMITMRLYIREFGQLISKATRDIEENKYNDEERKVLIAYHKKSKLYMKLLVINTGITATSYYAKPLLEQFGEIVEYFGSQKNENTTFIFLLPYRFHTFYELNDASTYFWTYLSQLPFLYISWMSQSASDCLMVALVYHVSGQIAVLATRITNIDTDPSKCTKQLHDVVRSQARLLRMGRIIEKAFSAMLLSQLMGGTILICILGYQILACLANGERAILISLVSYIILVLLILYAYCSIAETLITESTRLCEAYYNCKWYDMVGKNAKIIIFCMARSQKPLQLTAGKFSVFCLTTFTNTVKASMGYLSVLRTVM

>CfloOR14

MDLLPVQFRALRYFGLWYEAPEEHRLPKMAYRVLVFLVIIQFTILQVKNIPDPRDLDIAVLSDVVFLLFLYFAHVFKLLVFVLKRASVLELLGELRSSICRPRDAYEATTLGKYKRRARNVYFYRMIVSTISCSIFFLVPIAKAGRPAFPIAATFFEVDGSAVRFALAYLLQVVAVYVTATTEVFLDSTVCAFVILANAQVDFYRHRLIVNGGFAQGPAGSVGRFVEHHLIIQSIVRRVQSTFMGIIVPVFCFGLITLCTSLFKFTQISTTLLDRAFCSFYLAYLLYPIYVYCWYGNELQEMNESIADTVYKSDWTALTASQRKSHWYVIFISQKKHEISYHGQCVLSLQTFIWMMKTSYGAFNLLQQVSGDK

>CfloOR15

MEVLPCNFRCCQIFGLWYESSSYAVLKKMYRSAVLVIIFQFIIGEIIELFLMEGDVGEFTELLYLACTFGAFGVKLLNFMARREKILDLLEDFKSQNYQAKSPQELQLYDKYRRTAKLIFLCAFAACSLTGGALCFLPLIVASENKQLHLPLRYLQFWDTSNNVCFWISYALKILSVVCSVPLNVSMDTLIYGFLILSTGQFQMNSCRFRDGQESMKSCLNHYLMIVATVKKIQNFFVPVMLPLLLSSFITLCTSIFEVSRRQVGSFDFLLLSAYLVCMLFQIFFYCYFGNELQVQSQAVPEAVYCSNWIDRTREERKNLWLVMLFAQRGCDISFHGQCKLSLNTFTSVVKTSYAALNLLKK

>CfloOR16

MIKFFPMISTHTLCGRFCRYITWVCIYSITLNGISNALGPLIDRSSEPDLMVDAYSPCDARHPTCFWPSYVNQVFGYMCTSLVQMGCDNLVYNTMERISTHFRILEHRMLLLPKLVGANKCQETRYLKQCIRDHQSINETIKDLNEAFYEQIFIQFLTSISVLCTNIYLLSILDLMSSDFLSVFAFLCCALIENFFYCWYGYGVLMSSLHVSDAIANMDWWELTKESKQMLTFVMLQTSRKVYICKNAISTLTPEAFVNILKVSYSAFNILQKTSKK

>CfloOR17

MSIGGNTEIWEKKVRKTKIMDPTEEYDQLIRPVMISCHVFGCWSQQGPGGIISSKFHRTAILLFALIASTSTTAGAVVFWGLDMNETIECILVLSVLNLALLRIIVFASKRKDMMLIVETMRNDWLDSSPFERSVLRNKCRLAFKLAKFFIISVAMTILMFALMPIIEVKLADGETGQSKLPFRGYYFFNHTTSPRYECTYFVNSVIGCFSSSTIAGGTSFCLIATIHAAAKFALVQRDFRTLDSSDWTAGGPKIEGVVRKHLECIRFAEKVESVINFLALAQFITSTGLMCFAGFQLTTMLEDKTRLLKYAAFLQAAILELFLFSYSGHRLKTESENVADVTYLSNWIGTSSPTNFKMVSMRSSKPCTITAGKFYDMSLESFLKVMGSSFSYLTVLLAIKD

>CfloOR18

MEGDGAFTFYRRWLRLVGVWPLEEKNPLQTLRYFTAALAMASLFAHTLAEMYLSEAPISGMADLLVFCSSAFVALVKHMFMHLHGDKLAVTLKSYLNDWTDTKNEGFLKIMRSRVRMYRYQFLVYYSVGYVGTTLFFLRTVVLNAMRQRQSTDEDVELALVCQASYLSKESVAKYYVLIMTIQYLQSMCVSTSGAGTDCFFLAMVFHLCGQLEILRKRWSDADAVRLLDGPGRRGRVVELIERHRRLLDVGDKLEMSFNGIVVVQLLTSIVLICMSGFSVLVALQGSDYATVLVSVNCILFMITESFIYGYASDDLMEQSENIVQAVYSFGWYDADPVTKKDIGFVIMRARLPLRITAGKFFAVTRNTIVQLLKTTASILSVMRITLTATIAEELHTV

>CfloOR19

MHERKWNRDAKFALEFSKFCVWPIGLWPLDSETLSSRIRALYSFTTQIWMIGAQSAAAYLNCGSEEDNVDYVMMAVCAVMALSKIVAFRLHSREMRFVLASVEDDLLAVTEDDTRRVVRPFVKKGRTVFYIQMIFSYMTSAIIVISALPIFMPPADPVNGNNTLDSTPLRQLPLQTGCMFAGFRDGIYASMYVYEITVILVAQHGNVGSDVFIFALAMHLCSQIELLKIKLAKIGTISEEPLDDWRPRIVGYIQRHVGILKLIRTFNESLSTMLAIQIILNAGMNLMLGLRVIMAIKSGVLEDAVRPIFAFIVLMLQLYLMCYAGDRISHQAKSILDAIYDSYWFKLPTKLRQDLYYVAMRANKPVYLMAGNLMAINLENFKRVLKASFSYFSILQMMFEAE

>CfloOR20

MESLKISFAILSICGIWKPVGINSSSSKWAYDVWRLLFMPQPYLFACAQLARIVLVDLSFQEITEILFIFISILNICCKSVNYVVRRKDIINLTSMLSTDCLLPRDKEEIEIYRRCRQFIRYVTLSSVVLVEITALMFLCLPLFRSKRASRELPFPIYVPWSENKFWSTYAGEFLAIMTVSSISASSNTLIFGLLLEARNQFDLLAYRFGAMSRHGIMLDCDGVGGFEERTLLVQNIRHHGDIFKFIDAIKNTFSIAISGQFVTSSLVISMSVYQLTMNNSFNLGFLTNLLYLMCMLVEFFLYCWFSNELTLKSESFGKSIFKTNWVALDVKNKDIIVVMLRSSKPVVITSGFFIVLSLETFMKVLKLSYSAFSVLRRGK

>DmelOR

MQDQLDHELERIDKLPKLGLLWVEYSAYALGVNIAPRKRSSKYCRLTRILVLIVNLSIIYSLVAFIMENYMISFETYVEAVLLTFQLSVGVVKMFHFQNKVESCSQLVFSTETGEVLKSLGLFQLDLPRKKELLSSVSLILLNNWMIIDRQVMFFFKIVCMPVLYYCVRPYFQYIFDCYIKDKDTCEMTLTYPAIVPYLQLGNYEFPSYVIRFFLLQSGPLWCFFAVFGFNSLFVVLTRYESGLIKVLRFLVQNSTSDILVPKDQRVKYLQCCVRLFARISSHHNQIENLFKYIILVQCSVSSILICMLLYKISTVLEVGWVWMGMIMVYFVTIALEITLYNVSAQKVESQSELLFHDWYNCSWYNESREFKFMIKMMLLFSRRTFVLSVGGFTSLSHKFLVQVFRLSANFFLLLRNMNNK-

>DmelOR1a

MSKLIEVFLGNLWTQRFTFARMGLDLQPDKKGNVLRSPLLYCIMCLTTSFELCTVCAFMVQNRNQIVLCSEALMHGLQMVSSLLKMAIFLAKSHDLVDLIQQIQSPFTEEDLVGTEWRSQNQRGQLMAAIYFMMCAGTSVSFLLMPVALTMLKYHSTGEFAPVSSFRVLLPYDVTQPHVYAMDCCLMVFVLSFFCCSTTGVDTLYGWCALGVSLQYRRLGQQLKRIPSCFNPSRSDFGLSGIFVEHARLLKIVQHFNYSFMEIAFVEVVIICGLYCSVICQYIMPHTNQNFAFLGFFSLVVTTQLCIYLFGAEQVRLEAERFSRLLYEVIPWQNLPPKHRKLFLFPIERAQRETVLGAYFFELGRPLLVWIFRTAGSFTTLMNALYAKYETH-

>DmelOR2a

MEKQEDFKLNTHSAVYYHWRVWELTGLMRPPGVSSLLYVVYSITVNLVVTVLFPLSLLARLLFTTNMAGLCENLTITITDIVANLKFANVYMVRKQLHEIRSLLRLMDARARLVGDPEEISALRKEVNIAQGTFRTFASIFVFGTTLSCVRVVVRPDRELLYPAWFGVDWMHSTRNYVLINIYQLFGLIVQAIQNCASDSYPPAFLCLLTGHMRALELRVRRIGCRTEKSNKGQTYEAWREEVYQELIECIRDLARVHRLREIIQRVLSVPCMAQFVCSAAVQCTVAMHFLYVADDHDHTAMIISIVFFSAVTLEVFVICYFGDRMRTQSEALCDAFYDCNWIEQLPKFKRELLFTLARTQRPSLIYAGNYIALSLETFEQVMRFTYSVFTLLLRAK-

>DmelOR7a

MAVSTRVATKQEVPESRRAFRNLFNCFYALGMQAPDGSRPTTSSTWQRIYACFSVVMYVWQLLLVPTFFVISYRYMGGMEITQVLTSAQVAIDAVILPAKIVALAWNLPLLRRAEHHLAALDARCREQEEFQLILDAVRFCNYLVWFYQICYAIYSSSTFVCAFLLGQPPYALYLPGLDWQRSQMQFCIQAWIEFLIMNWTCLHQASDDVYAVIYLYVVRIQVQLLARRVEKLGTDDSGQVEIYPDERRQEEHCAELQRCIVDHQTMLQLLDCISPVISRTIFVQFLITAAIMGTTMINIFIFANTNTKIASIIYLLAVTLQTAPCCYQATSLMLDNERLALAIFQCQWLGQSARFRKMLLYYLHRAQQPITLTAMKLFPINLATYFSIAKFSFSLYTLIKGMNLGERFNRTN-

>DmelOR9a

MSDKVKGKKQEEKDQSLRVQILVYRCMGIDLWSPTMANDRPWLTFVTMGPLFLFMVPMFLAAHEYITQVSLLSDTLGSTFASMLTLVKFLLFCYHRKEFVGLIYHIRAILAKEIEVWPDAREIIEVENQSDQMLSLTYTRCFGLAGIFAALKPFVGIILSSIRGDEIHLELPHNGVYPYDLQVVMFYVPTYLWNVMASYSAVTMALCVDSLLFFFTYNVCAIFKIAKHRMIHLPAVGGKEELEGLVQVLLLHQKGLQIADHIADKYRPLIFLQFFLSALQICFIGFQVADLFPNPQSLYFIAFVGSLLIALFIYSKCGENIKSASLDFGNGLYETNWTDFSPPTKRALLIAAMRAQRPCQMKGYFFEASMATFSTIVRSAVSYIMMLRSFNA-

>DmelOR10a

MSEWLRFLKRDQQLDVYFFAVPRLSLDIMGYWPGKTGDTWPWRSLIHFAILAIGVATELHAGMCFLDRQQITLALETLCPAGTSAVTLLKMFLMLRFRQDLSIMWNRLRGLLFDPNWERPEQRDIRLKHSAMAARINFWPLSAGFFTCTTYNLKPILIAMILYLQNRYEDFVWFTPFNMTMPKVLLNYPFFPLTYIFIAYTGYVTIFMFGGCDGFYFEFCAHLSALFEVLQAEIESMFRPYTDHLELSPVQLYILEQKMRSVIIRHNAIIDLTRFFRDRYTIITLAHFVSAAMVIGFSMVNLLTLGNNGLGAMLYVAYTVAALSQLLVYCYGGTLVAESSTGLCRAMFSCPWQLFKPKQRRLVQLLILRSQRPVSMAVPFFSPSLATFAAILQTSGSIIALVKSFQ-

>DmelOR13a

MFYSYPYKALSFPIQCVWLKLNGSWPLTESSRPWRSQSLLATAYIVWAWYVIASVGITISYQTAFLLNNLSDIIITTENCCTTFMGVLNFVRLIHLRLNQRKFRQLIENFSYEIWIPNSSKNNVAAECRRRMVTFSIMTSLLACLIIMYCVLPLVEIFFGPAFDAQNKPFPYKMIFPYDAQSSWIRYVMTYIFTSYAGICVVTTLFAEDTILGFFITYTCGQFHLLHQRIAGLFAGSNAELAESIQLERLKRIVEKHNNIISFAKRLEDFFNPILLANLMISSVLICMVGFQIVTGKNMFIGDYVKFIIYISSALSQLYVLCENGDALIKQSTLTAQILYECQWEGSDRIEIQSFTPTTKRIRNQIWFMILCSQQPVRITAFKFSTLSLQSFTAILSTSISYFTLLRSVYFDDEKKLD-

>DmelOR19a

MDISKVDSTRALVNHWRIFRIMGIHPPGKRTFWGRHYTAYSMVWNVTFHICIWVSFSVNLLQSNSLETFCESLCVTMPHTLYMLKLINVRRMRGQMISSHWLLRLLDKRLGCDDERQIIMAGIERAEFIFRTIFRGLACTVVLGIIYISASSEPTLMYPTWIPWNWRDSTSAYLATAMLHTTALMANATLVLNLSSYPGTYLILVSVHTKALALRVSKLGYGAPLPAVRMQAILVGYIHDHQIILRLFKSLERSLSMTCFLQFFSTACAQCTICYFLLFGNVGIMRFMNMLFLLVILTTETLLLCYTAELPCKEGESLLTAVYSCNWLSQSVNFRRLLLLMLARCQIPMILVSGVIVPISMKTFTVMIKGAYTMLTLLNEIRKTSLE-

>DmelOR19b

MDISKVDSTRALVNHWRIFRIMGIHPPGKRTFWGRHYTAYSMVWNVTFHICIWVSFSVNLLQSNSLETFCESLCVTMPHTLYMLKLINVRRMRGEMISSHWLLRLLDKRLGCADERQIIMAGIERAEFIFRTIFRGLACTVVLGIIYISASSEPTLMYPTWIPWNWKDSTSAYLATAMLHTTALMANATLVLNLSSYPGTYLILVSVHTKALALRVSKLGYGAPLPAVRMQAILVGYIHDHQIILRLFKSLERSLSMTCFLQFFSTACAQCTICYFLLFGNVGIMRFMNMLFLLVILTTETLLLCYTAELPCKEGESLLTAVYSCNWLSQSVNFRRLLLLMLARCQIPMILVSGVIVPISMKTFTVMIKGAYTMLTLLNEIRKTSLE-

>DmelOR22

MLSKFFPHIKEKPLSERVKSRDAFIYLDRLMWSFGWTEPENKRWDLHYKLWSTFVTLLIFILLPISVSVEYIQRFKTFSAGEFLSSIQIGVDMYGSSFKSYLTMMGYKKRQEAKMSLDELDKRCVCDEERTIVHRHVALGNFCYIFYHIAYTSFLISNFLSFIMKRIHAWRMYFPYVDPEKQFYISSIAEVILMGWAVFMALCTDVCPLISMLIARCHITLLKQRLRNLRSEPGRTEDEYLKELADCVRDHRLILDYVDALRSVFSGTIFVQFLLIGIVLGLSMINIMFFSTLSTGVAVVLFMSCVSMQTFPFCYLCNMIMDDCQEMADSLFQSDWTSADRRYKSTLVYFLHNIQQPIILTAGGVFPISMQTNLNMVKLAFTVVTIVKQFNLAEKFQ-

>DmelOR22a

MLSKFFPHIKEKPLSERVKSRDAFIYLDRVMWSFGWTEPENKRWILPYKLWLAFVNIVMLILLPISISIEYLHRFKTFSAGEFLSSLEIGVNMYGSSFKCAFTLIGFKKRQEAKVLLDQLDKRCLSDKERSTVHRYVAMGNFFDILYHIFYSTFVVMNFPYFLLERRHAWRMYFPYIDSDEQFYISSIAECFLMTEAIYMDLCTDVCPLISMLMARCHISLLKQRLRNLRSKPGRTEDEYLEELTECIRDHRLLLDYVDALRPVFSGTIFVQFLLIGTVLGLSMINLMFFSTFWTGVATCLFMFDVSMETFPFCYLCNMIIDDCQEMSNCLFQSDWTSADRRYKSTLVYFLHNLQQPITLTAGGVFPISMQTNLAMVKLAFSVVTVIKQFNLAERFQ-

>DmelOR22b

MLSQFFPHIKEKPLSERVKSRDAFVYLDRVMWSFGWTVPENKRWDLHYKLWSTFVTLLIFILLPISVSVEYIQRFKTFSAGEFLSSIQIGVNMYGSSFKSYLTMMGYKKRQEAKMSLDELDKRCVCDEERTIVHRHVALGNFCYIFYHIAYTSFLISNFLSFIMKRIHAWRMYFPYVDPEKQFYISSIAEVILRGWAVFMDLCTDVCPLISMVIARCHITLLKQRLRNLRSEPGRTEDEYLKELADCVRDHRLILDYVDALRSVFSGTIFVQFLLIGIVLGLSMINIMFFSTLSTGVAVVLFMSCVSMQTFPFCYLCNMIMDDCQEMADSLFQSDWTSADRRYKSTLVYFLHNLQQPIILTAGGVFPISMQTNLNMVKLAFTVVTIVKQFNLAEKFQ-

>DmelOR22c

MTDSGQPAIADHFYRIPRISGLIVGLWPQRIRGGGGRPWHAHLLFVFAFAMVVVGAVGEVSYGCVHLDNLVVALEAFCPGTTKAVCVLKLWVFFRSNRRWAELVQRLRAILWESRRQEAQRMLVGLATTANRLSLLLLSSGTATNAAFTLQPLIMGLYRWIVQLPGQTELPFNIILPSFAVQPGVFPLTYVLLTASGACTVFAFSFVDGFFICSCLYICGAFRLVQQDIRRIFADLHGDSVDVFTEEMNAEVRHRLAQVVERHNAIIDFCTDLTRQFTVIVLMHFLSAAFVLCSTILDIMLNTSSLSGLTYICYIIAALTQLFLYCFGGNHVSESSAAVADVLYDMEWYKCDARTRKVILMILRRSQRAKTIAVPFFTPSLPALRSILSTAGSYITLLKTFL-

>DmelOR24

MDSFLQVQKSTIALLGFDLFSENREMWKRPYRAMNVFSIAAIFPFILAAVLHNWKNVLLLADAMVALLITILGLFKFSMILYLRRDFKRLIDKFRLLMSNEAEQGEEYAEILNAANKQDQRMCTLFRTCFLLAWALNSVLPLVRMGLSYWLAGHAEPELPFPCLFPWNIHIIRNYVLSFIWSAFASTGVVLPAVSLDTIFCSFTSNLCAFFKIAQYKVVRFKGGSLKESQATLNKVFALYQTSLDMCNDLNQCYQPIICAQFFISSLQLCMLGYLFSITFAQTEGVYYASFIATIIIQAYIYCYCGENLKTESASFEWAIYDSPWHESLGAGGASTSICRSLLISMMRAHRGFRITGYFFEANMEAFSSIVRTAMSYITMLRSFS-

>DmelOR24a

MLPRFLTASYPMERHYFMVPKFALSLIGFYPEQKRTVLVKLWSFFNFFILTYGCYAEAYYGIHYIPINIATALDALCPVASSILSLVKMVAIWWYQDELRSLIERVRFLTEQQKSKRKLGYKKRFYTLATQLTFLLLCCGFCTSTSYSVRHLIDNILRRTHGKDWIYETPFKMMFPDLLLRLPLYPITYILVHWHGYITVVCFVGADGFFLGFCLYFTVLLLCLQDDVCDLLEVENIEKSPSEAEEARIVREMEKLVDRHNEVAELTERLSGVMVEITLAHFVTSSLIIGTSVVDILLFSGLGIIVYVVYTCAVGVEIFLYCLGGSHIMEACSNLARSTFSSHWYGHSVRVQKMTLLMVARAQRVLTIKIPFFSPSLETLTSILRFTGSLIALAKSVI-

>DmelOR30a

MELKSMDPVEMPIFGSTLKLMKFWSYLFVHNWRRYVAMTPYIIINCTQYVDIYLSTESLDFIIRNVYLAVLFTNTVVRGVLLCVQRFSYERFINILKSFYIELLQSDDPIINILVKETTRLSVLISRINLLMGCCTCIGFVTYPIFGSERVLPYGMYLPTIDEYKYASPYYEIFFVIQAIMAPMGCCMYIPYTNMVVTFTLFAILMCRVLQHKLRSLEKLKNEQVRGEIIWCIKYQLKLSGFVDSMNALNTHLHLVEFLCFGAMLCVLLFSLIIAQTIAQTVIVIAYMVMIFANSVVLYYVANELYFQSFDIAIAAYESNWMDFDVDTQKTLKFLIMRSQKPLAILVGGTYPMNLKMLQSLLNAIYSFFTLLRRVYG-

>DmelOR33a

MDSRRKVRSENLYKTYWLYWRLLGVEGDYPFRRLVDFTITSFITILFPVHLILGMYKKPQIQVFRSLHFTSECLFCSYKFFCFRWKLKEIKTIEGLLQDLDSRVESEEERNYFNQNPSRVARMLSKSYLVAAISAIITATVAGLFSTGRNLMYLGWFPYDFQATAAIYWISFSYQAIGSSLLILENLANDSYPPITFCVVSGHVRLLIMRLSRIGHDVKLSSSENTRKLIEGIQDHRKLMKIIRLLRSTLHLSQLGQFLSSGINISITLINILFFAENNFAMLYYAVFFAAMLIELFPSCYYGILMTMEFDKLPYAIFSSNWLKMDKRYNRSLIILMQLTLVPVNIKAGGIVGIDMSAFFATVRMAYSFYTLALSFRV-

>DmelOR33b

MDLKPRVIRSEDIYRTYWLYWHLLGLESNFFLNRLLDLVITIFVTIWYPIHLILGLFMERSLGDVCKGLPITAACFFASFKFICFRFKLSEIKEIEILFKELDQRALSREECEFFNQNTRREANFIWKSFIVAYGLSNISAIASVLFGGGHKLLYPAWFPYDVQATELIFWLSVTYQIAGVSLAILQNLANDSYPPMTFCVVAGHVRLLAMRLSRIGQGPEETIYLTGKQLIESIEDHRKLMKIVELLRSTMNISQLGQFISSGVNISITLVNILFFADNNFAITYYGVYFLSMVLELFPCCYYGTLISVEMNQLTYAIYSSNWMSMNRSYSRILLIFMQLTLAEVQIKAGGMIGIGMNAFFATVRLAYSFFTLAMSLR-

>DmelOR33c

MVIIDSLSFYRPFWICMRLLVPTFFKDSSRPVQLYVVLLHILVTLWFPLHLLLHLLLLPSTAEFFKNLTMSLTCVACSLKHVAHLYHLPQIVEIESLIEQLDTFIASEQEHRYYRDHVHCHARRFTRCLYISFGMIYALFLFGVFVQVISGNWELLYPAYFPFDLESNRFLGAVALGYQVFSMLVEGFQGLGNDTYTPLTLCLLAGHVHLWSIRMGQLGYFDDETVVNHQRLLDYIEQHKLLVRFHNLVSRTISEVQLVQLGGCGATLCIIVSYMLFFVGDTISLVYYLVFFGVVCVQLFPSCYFASEVAEELERLPYAIFSSRWYDQSRDHRFDLLIFTQLTLGNRGWIIKAGGLIELNLNAFFATLKMAYSLFAVVVRAKGI-

>DmelOR35a

MVRYVPRFADGQKVKLAWPLAVFRLNHIFWPLDPSTGKWGRYLDKVLAVAMSLVFMQHNDAELRYLRFEASNRNLDAFLTGMPTYLILVEAQFRSLHILLHFEKLQKFLEIFYANIYIDPRKEPEMFRKVDGKMIINRLVSAMYGAVISLYLIAPVFSIINQSKDFLYSMIFPFDSDPLYIFVPLLLTNVWVGIVIDTMMFGETNLLCELIVHLNGSYMLLKRDLQLAIEKILVARDRPHMAKQLKVLITKTLRKNVALNQFGQQLEAQYTVRVFIMFAFAAGLLCALSFKAYTNPMANYIYAIWFGAKTVELLSLGQIGSDLAFTTDSLSTMYYLTHWEQILQYSTNPSENLRLLKLINLAIEMNSKPFYVTGLKYFRVSLQAGLKILQASFSYFTFLTSMQRRQMSN-

>DmelOR42a

MDLRRWFPTLYTQSKDSPVRSRDATLYLLRCVFLMGVRKPPAKFFVAYVLWSFALNFCSTFYQPIGFLTGYISHLSEFSPGEFLTSLQVAFNAWSCSTKVLIVWALVKRFDEANNLLDEMDRRITDPGERLQIHRAVSLSNRIFFFFMAVYMVYATNTFLSAIFIGRPPYQNYYPFLDWRSSTLHLALQAGLEYFAMAGACFQDVCVDCYPVNFVLVLRAHMSIFAERLRRLGTYPYESQEQKYERLVQCIQDHKVILRFVDCLRPVISGTIFVQFLVVGLVLGFTLINIVLFANLGSAIAALSFMAAVLLETTPFCILCNYLTEDCYKLADALFQSNWIDEEKRYQKTLMYFLQKLQQPITFMAMNVFPISVGTNISVTKFSFSVFTLVKQMNISEKLAKSEMEE-

>DmelOR42b

MVFELIRPAPLTEQKRSRDGCIYLYRAMKFIGWLPPKQGVLRYVYLTWTLMTFVWCTTYLPLGFLGSYMTQIKSFSPGEFLTSLQVCINAYGSSVKVAITYSMLWRLIKAKNILDQLDLRCTAMEEREKIHLVVARSNHAFLIFTFVYCGYAGSTYLSSVLSGRPPWQLYNPFIDWHDGTLKLWVASTLEYMVMSGAVLQDQLSDSYPLIYTLILRAHLDMLRERIRRLRSDENLSEAESYEELVKCVMDHKLILRYCAIIKPVIQGTIFTQFLLIGLVLGFTLINVFFFSDIWTGIASFMFVITILLQTFPFCYTCNLIMEDCESLTHAIFQSNWVDASRRYKTTLLYFLQNVQQPIVFIAGGIFQISMSSNISVAKFAFSVITITKQMNIADKFKTD-

>DmelOR43b

MFGHFKLVYPAPISEPIQSRDSNAYMMETLRNSGLNLKNDFGIGRKIWRVFSFTYNMVILPVSFPINYVIHLAEFPPELLLQSLQLCLNTWCFALKFFTLIVYTHRLELANKHFDELDKYCVKPAEKRKVRDMVATITRLYLTFVVVYVLYATSTLLDGLLHHRVPYNTYYPFINWRVDRTQMYIQSFLEYFTVGYAIYVATATDSYPVIYVAALRTHILLLKDRIIYLGDPSNEGSSDPSYMFKSLVDCIKAHRTMLNFCDAIQPIISGTIFAQFIICGSILGIIMINMVLFADQSTRFGIVIYVMAVLLQTFPLCFYCNAIVDDCKELAHALFHSAWWVQDKRYQRTVIQFLQKLQQPMTFTAMNIFNINLATNINVAKFAFTVYAIASGMNLDQKLSIKE-

>DmelOR45a

MDASYFAVQRRALEIVGFDPSTPQLSLKHPIWAGILILSLISHNWPMVVYALQDLSDLTRLTDNFAVFMQGSQSTFKFLVMMAKRRRIGSLIHRLHKLNQAASATPNHLEKIERENQLDRYVARSFRNAAYGVICASAIAPMLLGLWGYVETGVFTPTTPMEFNFWLDERKPHFYWPIYVWGVLGVAAAAWLAIATDTLFSWLTHNVVIQFQLLELVLEEKDLNGGDSRLTGFVSRHRIALDLAKELSSIFGEIVFVKYMLSYLQLCMLAFRFSRSGWSAQVPFRATFLVAIIIQLSSYCYGGEYIKQQSLAIAQAVYGQINWPEMTPKKRRLWQMVIMRAQRPAKIFGFMFVVDLPLLLWVIRTAGSFLAMLRTFER-

>DmelOR45b

MYPRFLSRNYPLAKHLFFVTRYSFGLLGLRFGKEQSWLHLLWLVFNFVNLAHCCQAEFVFGWSHLRTSPVDAMDAFCPLACSFTTLFKLGWMWWRRQEVADLMDRIRLLIGEQEKREDSRRKVAQRSYYLMVTRCGMLVFTLGSITTGAFVLRSLWEMWVRRHQEFKFDMPFRMLFHDFAHRMPWFPVFYLYSTWSGQVTVYAFAGTDGFFFGFTLYMAFLLQALRYDIQDALKPIRDPSLRESKICCQRLADIVDRHNEIEKIVKEFSGIMAAPTFVHFVSASLVIATSVIDILLYSGYNIIRYVVYTFTVSSAIFLYCYGGTEMSTESLSLGEAAYSSAWYTWDRETRRRVFLIILRAQRPITVRVPFFAPSLPVFTSVIKFTGSIVALAKTIL-

>DmelOR46aA

MSKGVEIFYKGQKAFLNILSLWPQIERRWRIIHQVNYVHVIVFWVLLFDLLLVLHVMANLSYMSEVVKAIFILATSAGHTTKLLSIKANNVQMEELFRRLDNEEFRPRGANEELIFAAACERSRKLRDFYGALSFAALSMILIPQFALDWSHLPLKTYNPLGENTGSPAYWLLYCYQCLALSVSCITNIGFDSLCSSLFIFLKCQLDILAVRLDKIGRLITTSGGTVEQQLKENIRYHMTIVELSKTVERLLCKPISVQIFCSVLVLTANFYAIAVLSDERLELFKYVTYQACMLIQIFILCYYAGEVTQRSLDLPHELYKTSWVDWDYRSRRIALLFMQRLHSTLRIRTLNPSLGFDLMLFSSIVNCSYSYFALLKRVNS-

>DmelOR46aB

MVTEDFYKYQVWYFQILGVWQLPTWAADHQRRFQSMRFGFILVILFIMLLLFSFEMLNNISQVREILKVFFMFATEISCMAKLLHLKLKSRKLAGLVDAMLSPEFGVKSEQEMQMLELDRVAVVRMRNSYGIMSLGAASLILIVPCFDNFGELPLAMLEVCSIEGWICYWSQYLFHSICLLPTCVLNITYDSVAYSLLCFLKVQLQMLVLRLEKLGPVIEPQDNEKIAMELRECAAYYNRIVRFKDLVELFIKGPGSVQLMCSVLVLVSNLYDMSTMSIANGDAIFMLKTCIYQLVMLWQIFIICYASNEVTVQSSRLCHSIYSSQWTGWNRANRRIVLLMMQRFNSPMLLSTFNPTFAFSLEAFGSIVNCSYSYFALLKRVNS-

>DmelOR47b

MNDSGYQSNLSLLRVFLDEFRSVLRQESPGLIPRLAFYYVRAFLSLLCQYPNKKLASLPLYRWINLFIMCNVMTIFWTMFVALPESKNVIEMGDDLVWISGMALVFTKIFYMHLRCDEIDELISDFEYYNRELRPHNIDEEVLGWQRLCYVIESGLYINCFCLVNFFSAAIFLQPLLGEGKLPFHSVYPFQWHRLDLHPYTFWFLYIWQSLTSQHNLMSILMVDMVGISTFLQTALNLKLLCIEIRKLGDMEVSDKRFHEEFCRVVRFHQHIIKLVGKANRAFNGAFNAQLMASFSLISISTFETMAAAAVDPKMAAKFVLLMLVAFIQLSLWCVSGTLVYTQSVEVAQAAFDINDWHTKSPGIQRDISFVILRAQKPLMYVAEPFLPFTLGTYMLVLKNCYRLLALMQESM-

>DmelOR49a

MEKLRSYEDFIFMANMMFKTLGYDLFHTPKPWWRYLLVRGYFVLCTISNFYEASMVTTRIIEWESLAGSPSKIMRQGLHFFYMLSSQLKFITFMINRKRLLQLSHRLKELYPHKEQNQRKYEVNKYYLSCSTRNVLYVYYFVMVVMALEPLVQSCIMYLIGFGKADFTYKRIFPTRLTFDSEKPLGYVLAYVIDFTYSQFIVNVSLGTDLWMMCVSSQISMHLGYLANMLASIRPSPETEQQDCDFLASIIKRHQLMIRLQKDVNYVFGLLLASNLFTTSCLLCCMAYYTVVEGFNWEGISYMMLFASVAAQFYVVSSHGQMLIDLSTNLAKAAFESKWYEGSLRYKKEILILMAQAQRPLEISARGVIIISLDTFKILMTITYRFFAVIRQTVEK-

>DmelOR49b

MFEDIQLIYMNIKILRFWALLYDKNLRRYVCIGLASFHIFTQIVYMMSTNEGLTGIIRNSYMLVLWINTVLRAYLLLADHDRYLALIQKLTEAYYDLLNLNDSYISEILDQVNKVGKLMARGNLFFGMLTSMGFGLYPLSSSERVLPFGSKIPGLNEYESPYYEMWYIFQMLITPMGCCMYIPYTSLIVGLIMFGIVRCKALQHRLRQVALKHPYGDRDPRELREEIIACIRYQQSIIEYMDHINELTTMMFLFELMAFSALLCALLFMLIIVSGTSQLIIVCMYINMILAQILALYWYANELREQNLAVATAAYETEWFTFDVPLRKNILFMMMRAQRPAAILLGNIRPITLELFQNLLNTTYTFFTVLKRVYG-

>DmelOR56a

MFKVKDLLLSPTTFEDPIFGTHLRYFQWYGYVASKDQNRPLLSLIRCTILTASIWLSCALMLARVFRGYENLNDGATSYATAVQYFAVSIAMFNAYVQRDKVISLLRVAHSDIQNLMHEADNREMELLVATQAYTRTITLLIWIPSVIAGLMAYSDCIYRSLFLPKSVFNVPAVRRGEEHPILLFQLFPFGELCDNFVVGYLGPWYALGLGITAIPLWHTFITCLMKYVNLKLQILNKRVEEMDITRLNSKLVIGRLTASELTFWQMQLFKEFVKEQLRIRKFVQELQYLICVPVMADFIIFSVLICFLFFALTVGVPSKMDYFFMFIYLFVMAGILWIYHWHATLIVECHDELSLAYFSCGWYNFEMPLQKMLVFMMMHAQRPMKMRALLVDLNLRTFIDIGRGAYSYFNLLRSSHLY-

>DmelOR59a

MAEVRVDSLEFFKSHWTAWRYLGVAHFRVENWKNLYVFYSIVSNLLVTLCYPVHLGISLFRNRTITEDILNLTTFATCTACSVKCLLYAYNIKDVLEMERLLRLLDERVVGPEQRSIYGQVRVQLRNVLYVFIGIYMPCALFAELSFLFKEERGLMYPAWFPFDWLHSTRNYYIANAYQIVGISFQLLQNYVSDCFPAVVLCLISSHIKMLYNRFEEVGLDPARDAEKDLEACITDHKHILELFRRIEAFISLPMLIQFTVTALNVCIGLAALVFFVSEPMARMYFIFYSLAMPLQIFPSCFFGTDNEYWFGRLHYAAFSCNWHTQNRSFKRKMMLFVEQSLKKSTAVAGGMMRIHLDTFFSTLKGAYSLFTIIIRMRK-

>DmelOR59b

MAVFKLIKPAPLTEKVQSRQGNIYLYRAMWLIGWIPPKEGVLRYVYLFWTCVPFAFGVFYLPVGFIISYVQEFKNFTPGEFLTSLQVCINVYGASVKSTITYLFLWRLRKTEILLDSLDKRLANDSDRERIHNMVARCNYAFLIYSFIYCGYAGSTFLSYALSGRPPWSVYNPFIDWRDGMGSLWIQAIFEYITMSFAVLQDQLSDTYPLMFTIMFRAHMEVLKDHVRSLRMDPERSEADNYQDLVNCVLDHKTILKCCDMIRPMISRTIFVQFALIGSVLGLTLVNVFFFSNFWKGVASLLFVITILLQTFPFCYTCNMLIDDAQDLSNEIFQSNWVDAEPRYKATLVLFMHHVQQPIIFIAGGIFPISMNSNITVAKFAFSIITIVRQMNLAEQFQ-

>DmelOR59c

MTKFFFKRLQTAPLDQEVSSLDASDYYYRIAFFLGWTPPKGALLRWIYSLWTLTTMWLGIVYLPLGLSLTYVKHFDRFTPTEFLTSLQVDINCIGNVIKSCVTYSQMWRFRRMNELISSLDKRCVTTTQRRIFHKMVARVNLIVILFLSTYLGFCFLTLFTSVFAGKAPWQLYNPLVDWRKGHWQLWIASILEYCVVSIGTMQELMSDTYAIVFISLFRCHLAILRDRIANLRQDPKLSEMEHYEQMVACIQDHRTIIQCSQIIRPILSITIFAQFMLVGIDLGLAAISILFFPNTIWTIMANVSFIVAICTESFPCCMLCEHLIEDSVHVSNALFHSNWITADRSYKSAVLYFLHRAQQPIQFTAGSIFPISVQSNIAVAKFAFTIITIVNQMNLGEKFFSDRSNGDINP-

>DmelOR63a

MYSPEEAAELKRRNYRSIREMIRLSYTVGFNLLDPSRCGQVLRIWTIVLSVSSLASLYGHWQMLARYIHDIPRIGETAGTALQFLTSIAKMWYFLFAHRQIYELLRKARCHELLQKCELFERMSDLPVIKEIRQQVESTMNRYWASTRRQILIYLYSCICITTNYFINSFVINLYRYFTKPKGSYDIMLPLPSLYPAWEHKGLEFPYYHIQMYLETCSLYICGMCAVSFDGVFIVLCLHSVGLMRSLNQMVEQATSELVPPDRRVEYLRCCIYQYQRVANFATEVNNCFRHITFTQFLLSLFNWGLALFQMSVGLGNNSSITMIRMTMYLVAAGYQIVVYCYNGQRFATASEEIANAFYQVRWYGESREFRHLIRMMLMRTNRGFRLDVSWFMQMSLPTLMAMVRTSGQYFLLLQNVNQK-

>DmelOR64

MKLSETLKIDYFRVQLNAWRICGALDLSEGRYWSWSMLLCILVYLPTPMLLRGVYSFEDPVENNFSLSLTVTSLSNLMKFCMYVAQLTKMVEVQSLIGQLDARVSGESQSERHRNMTEHLLRMSKLFQITYAVVFIIAAVPFVFETELSLPMPMWFPFDWKNSMVAYIGALVFQEIGYVFQIMQCFAADSFPPLVLYLISEQCQLLILRISEIGYGYKTLEENEQDLVNCIRDQNALYRLLDVTKSLVSYPMMVQFMVIGINIAITLFVLIFYVETLYDRIYYLCFLLGITVQTYPLCYYGTMVQESFAELHYAVFCSNWVDQSASYRGHMLILAERTKRMQLLLAGNLVPIHLSTYVACWKGAYSFFTLMADRDGLGS-

>DmelOR65a

MTELRSERKNGNWDRLFGPFFESWAVFKAPQAKSRHIIAYWTRDQLKALGFYMNSEQRRLPRIVAWQYFVSIQLATALASLFYGISESIGDIVNLGRDLVFIITIIFICFRLVFFAQYAGELDVIIDALEDIYHWSIKGPATKEVQETKRLHFLLFMALIITWFSFLILFMLIKISTPFWIESQTLPFHVSWPFQLHDPSKHPIAYIIIFVSQSTTMLYFLIWLGVVENMGVSLFFELTSALRVLCIELRNLQELCLGDEDMLYRELCRMTKFHQQIILLTDRCNHIFNGAFIMQMLINFLLVSLSLFEVLAAKKNPQVAVEYMIIMLMTLGHLSFWSKFGDMFSKESEQVALAVYEAYDPNVGSKSIHRQFCFFIQRAQKPLIMKASPFPPFNLENYMFILKQCYSILTILANTLE-

>DmelOR65b

MDIQRFLKFYKVGWKTYRDPLMEASHSSIYYWREQMKAMALFTTTEERLLPYRSKWHTLVYIQMVIFFASMSFGLTESMGDHVQMGRDLAFILGAFFIIFKTYYFCWYGDELDQVISDLDALHPWAQKGPNPVEYQTGKRWYFVMAFFLATSWSFFLCILLLLLITSPMWVHQQNLPFHAAFPFQWHEKSLHPISHAIIYLFQSYFAVYCLTWLLCIEGLSICIYAEITFGIEVLCLELRQIHRHNYGLQELRMETNRLVKLHQKIVEILDRTNDVFHGTLIMQMGVNFSLVSLSVLEAVEARKDPKVVAQFAVLMLLALGHLSMWSYCGDQLSQKSLQISEAAYEAYDPTKGSKDVYRDLCVIIRRGQDPLIMRASPFPSFNLINYSAILNQCYGILTFLLKTLD-

>DmelOR65c

MDIRGNVHRFVKFYIDGWKHFRDPTMESSYSAVYYWREQMKAMFLYTTSKERQMPYRSSWHTLVIIQATVCFLTMCYGVTESLGDKVQMGRDIAFIIGFFYIAFKIYYFQWYGDELDEVVEALETFHPWAQKGPGAVDYRTAKRWYFTLAFFLASSWLVFLCIFILLLITSPLWVHQQILPLHAAFPFQWHEKSIHPISHAFIYLFQTWNVMYFLTWLVCIEGLSVSIYVEITFAIEVLCLELRHLHQRCHGYEQLRLETNRLVQFHQKIVHILDHTNKVFHGTLIMQMGVNFFLVSLSVLEAMEARKDPKVVAQFAVLMLLALGHLSMWSYFGDLLSQKSLTISEAAYEAYDPIKGSKDVYRDLCLIIRRGQEPLIMRASPFPSFNFINYSAILNQCYGILTFLLKTLD-

>DmelOR67a

MDNVAEMPEEKYVEVDDFLRLAVKFYNTLGIDPYETGRKRTIWFQIYFALNMFNMVFSFYAEVATLVDRLRDNENFLESCILLSYVSFVVMGLSKIGAVMKKKPKMTALVRQLETCFPSPSAKVQEEYAVKSWLKRCHIYTKGFGGLFMIMYFAHALIPLFIYFIQRVLLHYPDAKQIMPFYQLEPWEFRDSWLFYPSYFHQSSAGYTATCGSIAGDLMIFAVVLQVIMHYERLAKVLREFKIQAHNAPNGAKEDIRKLQSLVANHIDILRLTDLMNEVFGIPLLLNFIASALLVCLVGVQLTIALSPEYFCKQMLFLISVLLEVYLLCSFSQRLIDASENVGHAAYDMDWLGSDKRFKKILIFISMRSQKPVCLKATVVLDLSMPTMSIFLGMSYKFFCAVRTMYQ-

>DmelOR67c

METAKDNTARTFMELMRVPVQFYRTIGEDIYAHRSTNPLKSLLFKIYLYAGFINFNLLVIGELVFFYNSIQDFETIRLAIAVAPCIGFSLVADFKQAAMIRGKKTLIMLLDDLENMHPKTLAKQMEYKLPDFEKTMKRVINIFTFLCLAYTTTFSFYPAIKASVKFNFLGYDTFDRNFGFLIWFPFDATRNNLIYWIMYWDIAHGAYLAGIAFLCADLLLVVVITQICMHFNYISMRLEDHPCNSNEDKENIEFLIGIIRYHDKCLKLCEHVNDLYSFSLLLNFLMASMQICFIAFQVTESTVEVIIIYCIFLMTSMVQVFMVCYYGDTLIAASLKVGDAAYNQKWFQCSKSYCTMLKLLIMRSQKPASIRPPTFPPISLVTYMKVISMSYQFFALLRTTYSNN-

>DmelOR67d

MLKMAKVEPVERYCKVIRMIRFCVGFCGNDVADPNFRMWWLTYAVMAAIAFFFACTGYTIYVGVVINGDLTIILQALAMVGSAVQGLTKLLVTANNASHMREVQNTYEDIYREYGSKGDEYAKCLEKRIRITWTLLIGFMLVYIILLGLVITFPIFYLLILHQKVLVMQFLIPFLDHTTDGGHLILTAAHVILITFGGFGNYGGDMYLFLFVTHVPLIKDIFCVKLTEFNELVMKRNDFPKVRAMLCDLLVWHQLYTRMLQTTKKIYSIVLFVQLSTTCVGLLCTISCIFMKAWPAAPLYLLYAAITLYTFCGLGTLVENSNEDFLSVIYTNCLWYELPVKEEKLIIMMLAKAQNEVVLTAADMAPLSMNTALQLTKGIYSFSMMLMNYLG-

>DmelOR69

MQLHDHMKYIDLGCKMACIPRYQWKGRPTERQLNASEQRIVFLLGTICQIFQITGVLIYWYCNGRLATETGTFVAQLSEMCSSFCLTFVGFCNVYAISTNRNQIETLLEELHQIYPRYRKNHYRCQHYFDMAMTIMRIEFLFYMILYVYYNSAPLWVLLWEHLHEEYDLSFKTQTNTWFPWKVHGSALGFGMAVLSITVGSFVGVGFSIVTQNLICLLTFQLKLHYDGISSQLVSLDCRRPGAHKELSILIVHHSRILQLGDQVNNIMNFVFGSSLVGATIAICMSSVSIMLLDLASAFKYASGLVAFVLYNFVICYMGTEVTLAMQLQDFMRYPDLVCQAAQLPRYTWNGRRSLEVKRNLAKRIIFWLGAVNLVYHNIGCVMYGYFVDGRTKDPIEYLAELASVASMLGFTIVGTLNLWKMLSLKTHFENLLNEFEELFQLIKHRAYRIHHYQEKYTRHIRNTFIFHTSAVVYYNSLPILLMIREHLSNSQQLGYRIQSNTWYPWQVQGSIPGFFAAVACQIFSCQTNMCVNMFIQFLINFFGIQLEIHFDGLARQLETIDARNPHAKDQLKYLIVYHTKLLNLADRVNRSFNFTFLISLSVSMISNCFLAFSMTMFDFGTSLKHLLGLLLFITYNFSMCRSGTHLILTSGKVLPAAFYNNWYEGDLVYRRMLLILMMRATKPYMWKTYKLAPVSITTYMATLKFSYQMFTCVRSLK-

>MmedOR1

MEKKTINDHTSIKLMRLCMNGIGMWSIEKRRDEIISNIVICYTIATLTVGLIVETTDIYYCLGDLREMSYVAPCLLNVIVELFLMGTFVINRSEVIAFSDYTTREFWSIPYIESERKLLDDCNRKSVKIIIAFIVVIQLVVWQYITIPIIESYGKNASERTLPYNLWFTFIPFKETPYYEICFFLQSAATLTTGVCATAFATFLFTINLYATGQFKILQQRLESSCQVYNIEKIKSVEQINLIAEESYANLRKCVELHNVLLKYITRLENLYCQIMLVETLACVFLICTTGFQIVLGVDSSILRTSRSALYFCCLVTQLLLYSWSCHEIIIESLEVAEAAYRAYWYSLSWSKYGKSFRQALLIIITRSRRPCVLTVGKFVPMSLETFTAVFNSALSYFTILRQMTEEMENS

>MmedOR2

MEAFRLHLFFLSILGVWKPQGWHGIKAFLYSIYGSTVVIFNHIFILSGILNLTKFKHVSLDVFIDNFSQMLALIVVRQRIICVIENRNSISQIIESTDKYPFKLRDRQEKLIFSKFSKLAKNILIYYPIVHMCIILVHTVGHISVMDPPYALPFQGWFPYNYTRKTKIYWATATYQLYAIFSEGSIDLILDLLLPCILCYMCGHIHILRHRFGVMTEKLQIMSENNEPREKIDSAERKMTAEWVEYHIDILRLVELVKKIFERMIFVQYTVSSLLLCTLAYLLSHTKCTTMTFAANFSFFMAMFIQILLPCYCADKLTFEFLDISTGIYNSNWYQLSNNIRRSVVVILRNTYQPVTITSGFFIILSLESFTKIIKLAYTIYNLLE

>MmedOR3

MKLPAHTKGYKVTPEGAVNFIRVTVYLTCISFPLTEKTKVRINYEIILWLSIFLSISLFAPLLASIIKYSDDTFIVMKSFILMSAISNYVIKVIIVRIYHKELQQLGSALDEFIKKANESEKVILQRYVDNTWKFHGFMTCSYYLTATAVLLGPLILPQKFPTDAVYPFPVDNQIISYIVYLHQCIVGYQCSAGMALDCQAALFLWYLSARFEILISEAKNVETFDELRNYIKKHQIILLYAKELIRPTRLIAFVTVVMTKIGMIFGGIVLISDEPVVIKIQFAILVISTTINIYVCAWAADNLITVSSTAMSNAIFEISWMHAPKLRNFLQTVIHRTQKPVVIKIPGLLETLSNEYYAQFLSAAFSCFAAARVVVSS

>MmedOR4

MENIPIDIHRKFLNINITILRYSGVWPLLPTAKIGWKVFNFIYRIFNLTVFIFYLITLGADAVTNYKDLTIFGSDGCYFFGTCMCVFKACKFWASYHKIIKLIVDVYDPIDVLVRSADPGILMNIKKSYYQESIAFWGFSTLCSFFHFSVIFLIPREKGILPIRAIYPFDTKISPNYELAIIYQAYCLAYALCVTIALDITTIGFIRWSTLQIAALTSNYKNSNPNVTKRASLVTSSSDARKIIEKLNKIKITDDDVEIETFLPLDYHETKYFINDLFLSRFTTCIKNHQRLIKIIRDLNAVLSPLMLVQFATSTCIICLNGYQMILAETMTYNQWMSGWECAYGKVKSNELRNLVTIAMMPAIKPFAFNAVGLFALSMPTLLAVVKSSYFMLILLTTVTED

>MmedOR5

MYSILPGSFVVLQAIGLWKPPEYNNSPILNYYYRLRTFITFFLIYSFTITGITGLILTTKDIADVTSDCFILLSIFAICGKIANIIWSRNEIIWIIDTLNSEPCKPLNNDEIIIQQKVDRLIWHSTLFYGILTEITVFMVTFGTLLLQLPIGTLPYNTYLPWDYSHGYLYWVAYGYQIISVCLSANSDIGFDTLVPGLMLQITAKLEILKYRFINLVDTLKLTQWNGVNDKSYHNFRIENKLIADYVKCHLIILKLADTINKTFDKVILLQFFISSIVLCISVYNLAFLDVFTTEFTSIILYLCCMLMEIFILCAAGNQVTIVSSTLSDAIYHTDWINLDTSAVKSLMIIMNRGLKPIIFSSGHIIKISYDSFKTPIKLSYSSYNVLQRT

>MmedOR6

MDVFEEPFYKMIKNFSHLIGQWPYQSSRKKFTIVTLIWIAFFMQFIPQIIAIVIHFDDRDVLFEAFSSMVIDFAFIIKYLNAIYRAGLMKELWESIRRDWTLLLNDVEKRTLQHHANLGNFFSMGYAGLAYMSTTIFVTEPIFPRIVNIFVETNETIPLKLALPLEYIIIDIDKHYWLILTITNIFVFNIIIVIISCDIVLITFVQHVCGLFAVVGCRLESTPFDENYLEGQKGEDFLSNSNDIPYKHLVSCIKGHKRALEYAERLERAYTLSNGIVSGLNAPVMSITGFLMITESSTIEQLLKYATFAISQMSHLFFLCFMSQRLADMSLRIQENIGNATWYNNSLKSQKLLVLMLMRSQVPCKLTAAKLMDLFIENFAVVVKTFASYITMLLSM

>MmedOR7

MENTSIESRQSVLSMAIAGLRLCGIWSLDSSSPIFLKLLHNISNIFGIITLIIFVGTLTIDLLLNSNDLLIATDDGCYLAGISVIVFKVYQFHRHHKRIKNLTDATYQPIYVFWKSTDIGVKTVLRTNKFYEDLGFTFFVSLGGFLVIALIFFVPTEEGALPIRGAYPFNTTISPMHEVAFCLQIYAVTYGLMVIVLMDGMGLGIMRWLNVQCIILASNYRNCRTNQNNSFYLESRDDLSKIASIEDDNNNVTDIYDEPDSNITTFCPFDEQDHAGMSDCFIGRFKKCIKNHQRLLNTIDELNACFSSCMLMQLFASFSMICLTGFQAVLGATTKTSLIKFVLYLGAAFSQLLYWCWFGNELLYEVFILPHD

>MmedOR8

MDYLSSPYCRLNKILLSCLGEWPYQTSTQRRFIRSTIYFFSASIIIPKIIKLIKVWGNLDMIIECIPMLLLDAVNFVKVVNGFINFRKMRELFDRIQDDWGLNYSKREFEIMQNYAEDGKKLSQFYASYMYATMLIYFCMPIIPKVLDIVLPLNTTRPELYLFEAEYFVDQHKFYYPILIHAYITCAVAVSMLVAFDTEYAIQALHGSGIFSALRYKLENLVIKDDEADYKNDEKIKQSTYNMVVQCAVLHKRALDYADLLESSRVTCFFFVLLVNIAAISITGVQTVMKLDQPTEAIRFGVYTLAQITHIFYNSYPAQMLFDNSWKTSDAIFAGNWYRAGSKSKNLLHMMIMRSRIPCKLTAGKIYLMSLENFTGVVKTSMSYFTVLLSFR

>MmedOR9

MDNSLDSFLRINRLFLSSLGQWPQQEQISKIFTLINAVFFLITQAYFQTGGMIAAKCDQPIFMESIAPVLISFMCLVKFVNFNYNADKMRKLLEIIQADWNSINDLEELKILNSWAKDSRKNTIMYAGALYGTMAPFMLGPLVPIFCKLMPAGVLPANSSIVLEKPVLFHVEYFYDLEKYYYPLLIHSYFGTMAYMTVAVAIDSMFMVYVQHACAIFAVIGNRLEHLADDSSINFYYNPHILNDEPYKRMIECIVQHSKALQYAQMIQSANSLSFFFNWDSICSLLLSVDFNWPSQRLADESARISETTTRCAWYLTSMRSRKLLQLFIMRSSVPCQLTAGSFYTLNMQNFSAVVRTSMSYFTVLTSVQ

>MmedOR10

MFKINPEFAIAYTKLTVTLVCSWPPGRNSSRLDFLLFRIKWWISWLMGIFLVIPLIYAAYIDRRNVLEFTKSLCLAVSCGQCAVKMFFCKLQHHRIKFLLDEMEEYVKVAEPFEREIFLGYIKNCGLVHVTLNVCSLVASVGVILGPLVLPQSLPTEAKYPFSVENHPNYEIIYIHQAFAGILCSSIGSIDCQIAMLLWFSIARLELLSLEMKNITNVYQFHNCVRKHQFLLWFVDEVIKAGRNLVATTVIMTTFAVILGGVHIVGNEPMLVKLQFVIIVGGFSMLLYVTAWPSEILTRMCQNIGWTIYNSEWIRNSKELNKGIEFVIQRSNKPAVIYISGIFPAISLNYYATFLSKTFSYFTTFRIILAKLE

>MmedOR11

MELDNIKLRHLEPYYNIKVSLTLTKYIGTWPPVLEPYRSIYLLYTCVSFIFILGIYLTVQTVNLFVIWGNIELMIATGFLLMTNSIHAYKVFVILGNQKRIQVLLDKLSTTNYYHNDDKYERVFTYYAWQGLYHHIAYQSFGTVAVLCWGLTPLADAVAGNTRRLPMEAWYPYNTKKNPAFEITSGHQAVAVLIACVHNIGMDTLVTGLINAACCQLEIIKQNLKNVDLDFEYQIDKCDYEDFMNKQINKIIKHSNEIYK

>MmedOR12

MIFKATPEFAIAFTKFSTLVGTCWPNYKNAPKWKFVLFQIRWWLTFCLSVSAFLPMCYAAYNHWRNILSFTKSLFDAANTSQTFIKMILSKIHYKRLQYLLYEMENYVTNAREDERELFIVYIKRCGKLHLFVMIFGFMAILIIIVAPIGLPQPFPNIADYPFPVDESPAFELVYAHQSAATLHCLSIPVFDMQIALLLWYSGARLELLAREFKTVTDNKHFVECVKKHQYLLWYIQEIIISSRYILATTSVTCVIAVITSGVHIAGNEPVGFKIPFAGASSIIAIILYISAWPSEHLIHMCEGVGTALYESEWVQNSKALNNSMLIVMHRAQKPSTIEVIGVMPILSLPYYATFLSKTFSYFTTLRVLLSKVEMD

>MmedOR13

MDIFDAPYYRIMKNSAKLIGQWPYQSREKRIIIIIIVWAFFFMQFVPQIIAIVVHIDDPDILFEACSNMAVDFVTAIKYINTICKTNLIKKLHDRVIIDWSLMLNDEEKSTLEKHTNLGYLFSSGWAGFAYMSATIFVLEPVFPRILNVFISVNATDPFKLALPLEYIIIDREKHYWIMLFVSTVFVYNIIIVLVSCDIMYITFVQHVCGLFAVVGCRLVNTPINENYSERHKAGDYLSNSKDIPYKHLVSCIRSHRRALEFAELLEDAYCISFGLTVGLNLPVISVTGFQIITQFNTIQQLLKYASFTITEILHLFFECFMSQRLTDMSLEMQKSIAEVQWFDNSIKSRKLLIIMTMRCQVPCKLTAAKIMDLTIENFGMMVKTSGSYFTMLLSMQ

>MmedOR14

MKFFEQSYFTLPRNFARSIGRWPYQSSLQSFLIGIVIISAFILQVGPKILADIVHSDDQELILETLAPTITNVMAFAKYINTFVNARMLKILFERIKDDWESVTDKKEKIILESYAGFGKLMATGYAGFVYAATVQFITEPVLPIILNNILRTNLSAPHKFADPMEWIIIDKEKYYWILLSNSSVCIMVILTVLISYDVIFITFVYHACGLFAITGHRIENLPHDENFKIINRNTNSLKNSRDVHYKHLVSCIRIHRMALKYVDLIESTFAGCFGVVVGLNLPLMSITGVQGMKFFRLLHNRMTLQQKIKYVMFTGAQMLHLFFECFLSQQLTDMSLRVQQHIANGNWYDISTKSQKLLILMTMRSQVPCILTAGKIMELSVESFGMMMKTSGSYFTVLLSMQ

>MmedOR15

MTRSLKLQDQETFDQVAKVLKWNKWLLSTLGLWPQSPNTFIFTVNFSYFVYHMAMEYLDLFLFIDNLEHVIENLTENMAFTQILVRIAMLKKYNRQLGEVVNEAFKDYDARIYRTDEERQVFIDYMKKAKLFIKLLCAFVTMTATSYYAKPITSPPPPPEGELDVEMENATMSFILPYRFHLFYQVNDSRTWALTYLSHFPFVFVSGFGQTAADCLMVTLVFHVSGKLASLAIRISEINTEPGVCKQELRSIIIEHDRLLKMGQSIEEAFSETLLAHLIGATSLVCILGYQLLVNYARGQGADLVTFFVFIFLVFLVLYAHCVVGESLITESFKVCEAYYDCLWYKMPKESSKTIVLCMARSQKPLGLTAGKFGAFCLSTLTDVVKTAMAYLSVLRTFLVIE

>MmedOR16

MYHKLLNKLVRILRYNGIWPVESTVRSYKLLNLIFRLFNLSIIVIMMLLTIADAIANFNDISLITDNLCFFVGCSEALTKGIKYCIEYKNIVKLMNDIYGPIDIINKKNNTEVMKGINEIARFENRQFKIIFGIVSLLIVARVLGADFKNKGFPIRALFPFDATATPYYHLIYLLISYGVLLVDYTLLGVDLMVVVIMRYLTIQVDILRANCRHCDIESTRRNIVINGYDDKNNENTDIRNFVGFEIEHEDSDGKDSFDDRLKRCIIHHQKVIYMLNGLNDCFSFCVVVQILGTTVLLCLNGFQIIMGRDIHLFMRRVLASTAALLQLLLWCWYGNKLSAAADSLTINLWMCGWEDNYKHGLRNFISIPMTLSLQTLELRAIGVVPLSLQTFVSAIKTSYSVLVLLLTVAKDE

>MmedOR17

MLFKATPEFALKFTKLIALLGTSWPNYEGTPKWKLVVFQIRWWSTFFLAITACLTMCYAACNQYQNILNLTKSLFDISNTSQTFVKMFFCKVHYKRMQYLLCDMEKYVTKAKPHERDLFIKYIKRCGKLHLTVMGSGLLIIHIIILAPIALPQPFPNIAEYPFPIDGHPTYELLYLHQSCATIHCLSIPAFDCQIAMLLWYAGARLELLSEECKTITDNKQFVECIKQHQYLLWYIQEITTSSRHILATTGCTCILTAISSGVHIVSNEPVAFKVPFMISWVIVSSTLYITSWPAENVLQMCEQVGMALYESPWVQNSKELNSSILFVVQRSQKPSTIEVPGILPVLSLRYFAMFLSRTFSYFTTLRVLLDKINLDMEIPAED

>MmedOR18

MLFKATPEFAIAFTKLTSILGSSWPHYKNATKCQLIVFNIKWWFFWFMSITAFLPMCYAAYNNTKNILSFTKSLCDAANCSQAFIKMLLCKIHYRKLQFLFYEMEKYVEQARANERELFISYIKRCGRLHVSIMISAVMAAVIIIIAPIGMPQPFPNVAEYPFPVDGHPTFEIIYLQQSIATIHCMSIPVFDCQIALLLWYAGARLELLGDEFRKVTDNQQFVACVKKHQYLLWFIQEIIMSSRHILATTVVMCTIAVITSGVHIVGKEPLADKVTSVILSTGLSAVLYLCAWPAEHLAQMCENVGAALYCSTWIKNSKESNKNIFIVIQRSQKPETIQVPGILPILSLTYYATFLSKTFSYFTTLRVVLDKMED

>MmedOR19

METTATKNDNTNQNKNELQLTKTNKYENVDYKSDADYAVVVARTLLTPLGIYPLHGSDTSLSKFLIAIQIIIVFGLMLFLLVPHFIWTFFDAEDLKKLMKIIAAQIFNSLALIKFWTMIIHKKELRNCLIQLENNWKNVLCEEDRVIMIKNAKIGRFFTIAYLSLSYGGALPYHILLPLTAERIVKEDNSTQIPLPYPTDYVFFVPEDSPGYEMLFVTHIIISTMILSTNCGIYSLIATYIMHACCLFEVVCRHLDEFSKNNTNNFKTELTWIVENHNRAIQFAETLESSLNIVFLCEMVGCTVIICFLEYGVIVDWEDGKLLGLVTYVILMTSIFVNCFIISFAGERLKEQSIKIGESAYFAEWYLLPKGLVYDFMLIMIRSSKPASLSTGKVSDLSLAGFAGLVKTSAAYLNFIRAVV

>MmedOR20

MQVLTFNFFLLSIMGVWKPRGWHGIKAALYYIYQTITIILNHLCLLSLLLDLQFKNIELGDLIDNLALVFTIIIIRQKIVCIIGNRPGITHILDSLKNSPFKLEDSKEEFIFSRFEKLARNIITYYPLIYLSTLSVHSTGFISVMDPPYTLPYKGWFPYNYTRTTKTYWVTAVYQIYIVLTMGSINAISDILLPCIICYMCGHIHILRYRFQVMAEKLRIMSKNNEPKDKIISTERKLMGDWVKYHIDILNLVKFTNEIFSSVIFIQYTVSSLLLCTIAYLLSHMEPTTMRFAGNSAFLTAMFFEILLPCYCADKLTFEFLDISTGIYDTNWYHLSNNIRKSIVIILRKSYRPVTMTSGFFIVLSLESFTKVIKLAYTIYNVLE

>NvitOR1

MDDDILCVRSLQINSVSSKIRKMKEMNPAKSVDVLSTSFLYFKLIGAWRPLNLPKWLRVIYDLFTISMVILMYEMLIVTEILAIIFAEENRLKVFQDIVHITITHVSGCFKMLFVINRRQSIMLLVNGCVAKQWYPPRNELEATILTKHNNLSRRITLTYATLVGASLLAAVLNPILYSTRVLTIATWYPCNISLPICYWSSYAHQTMGILAMAIAHVATDSLIVGFTIKICTQLNVLNQRLLSINFQLENTSARCQKSQEQSLALEAILVNECIVNYKDILRFADLLSRTFIEIVFIQFCVGLTVICSTVYLLAKLSIFSYDFFGLFLYLGCMLMQMFLFCWYGNEVVLDSTKLFHTIYNINWIELQIQTQSKLLLMMLVASSPIQLFRGAIIKVNLDAFINILKFSYSAFNLLQKSS

>NvitOR2

MEIYDSRYFIINKTLMTKMGLWPYQHPLKKFLLYGLKKNFGVHMDKIIEHLALLMYIYGIKLKLVTSILSEKKLKKVYENIMENWQQIKDVHERAILVEYSERGRTLTIGYIMYMTSALLFFIILPITPMVLNVIKPLNESRPWDFIMHGEFPVNDMHAHYGEIYLFDSLACIATVLVFCTVDSMYATCIEHCIGLFAIVKSRLDLSTKFVNRQGALGIKRDDKVYDLIVKTIKLHKKIINFTHILESSYSTSFLILMGMNMLYCSLVSVLLIIKSDALMERIRYGTILLGLLIHLFYISWPGQKIIDLSTGLFEDAYSNEWYETSIRSQNLLKFMRLRCLTPCQLTAGGIYVMNFANFASIIKTSTSYITVFASFT

>NvitOR3

MERSQKNQLQDFDWALGLNRFSLRLMGIWPADQDESSKSLLTVSRIPLMILVLLCGLFLPQMWALALVIEQLPLAIDNLMTSCPAFTSCIKLFFIWRSKTILQPVIDSALQDYLRPKSKSEETAMQREALRGRLVTIADYSIMASCYVGFIFMPMLGFNVRIINNLTDCDTQRVLLVQSYFPYDYARSPAFELTHLLQLAASFFVGMAISIPDDYFCALLFHASAQFEILGLQIESLPIDGSKSGRLLSGFIERHVHLNRMVSAVERSFEFVIAAQIFCMSIMVCCLGFQVLRMLDSAAEKPTPVQILTLGGTLFTMLLHTFVDCFASENLAARSSELFFKIYSSRWYSLSWSKMRCLVPMMLVAKTPRQIRAGKILSMSLATYCSIIKSTAGYISMLIAVSGR

>NvitOR4

MTIQSVLRRKVDVLLKAIALNKMCVSPKMILLVIKFAAMYLAIWPLDSSGKHWNTAFDCLWWFYVVNNVLVIIPTLLAFYSSRRDIIAAMFSWLEILALLEALIILANFRYYRSRMQPILKEAVDYIGSANSRRQLCLEKRASIITTTFGVIVALYIAGIIIYIYRPAVTEWDGMLTTAYYPASMRSPFADVFIYITQLTALLHNGVLIVSDAFTVLLLYVCTVRLEVLQKNILRVADYDELKLWIREHERVLRLVTDTNMVVRINISKTVISFVGYSVGAGLQIISRFALVIAMNAMRLFFSATFADDLVNSSNSLINTIYSTIWYKDNRDMKIGKIIIMLRCQKLLRISVGGIMPVLGKPYLTKILYTSVSYFMTFRAITGN

>NvitOR5

MDVLPLNFRTLWLCGIWHEENEKLTVPRIAYRFLVICLMFYFTFTLSAVVFVENSNVSELTEAIFLAVTYITLCLKIVNFAFRRAEMIEILHDFRHPYCKAEHSEESEILKGYSKQARKMYIYLMAFVMSDVAYFWSTFAFKVSKNIMELPYHTYQFYNMSSKAILFSTAALQATSVLYSVSINISFDTMTAGLLILTTGQLELNAHRLSKLGEHNVDSMNGYIAHNVLINGTVDKIESFIKTVVIPFLFFSLLSICASVFQLSEYSVFSLEFLGLFSFAICILLQVLVYCWFGNELMLKSEAVTDAIYRSDWTMLSPQNRKSLQVMMICNKDGRTVSFGGQCSLTLETFVWILKTSYATISLLNRVSA

>NvitOR6

MLGKSSLNSKIPIRERDFNYSMKLSRITLSIIGLWPFRENIRCSNFKFVVILVSILMTLLSSLTFVYQTDDDDKMFHSLINSLYMLMTLVKLLMMRCKNDKLEVILSEMRIDWRKYERFSDGNKRLVDLYTGKARTSSFVCIFFMEFSITTYFISRVAYALQQPAKIREWDLPYTAVYPFEVTSSLFVPMYLWQVFSAMCLGSVTISIDCLLVTTACHATGQLAALCENIKSYGHEQRHRDETLSSEIECSCIRCIIERHVDIVRYCRLVEDAYNLILLTEFIGTTFQFCLQMYIIVEHSHDKNIVGLLSFCIYLLVFNFRLFMYCNVFDAMVEMGEKVGASAYDISWYDFHPEAVRQLMFCILRANKPLNVTAGKFFSLNRNSYKNVIMTSSSYASVLLSIK

>NvitOR7

MDFLDSRYFILNKKMLHILGIWPYQKRLERYAIRSVYFFFMGVSFVPQILCVKKYFKVDSDKFIRGVTTLLYLSGVSLKLTIAILMNGKIQIVYSKVADNWKMFTDKDEIKTLLEYSEVGRMLTLGYVVYMVLAVIVFITMPYLPVVIDIVFPINGTRPRLFVLDGEYIVDKYENYNKIYIFESVCSVVSVPIFCTIDSTYAVCVQQCVALLAIVKLRLKVATKYTKNYLRDHKYNDASQQLIIKSADLHNKVIEFAQILETSYSMVFLLLMGMNCLILSVGTLVILVNLNNPLELSRYIMIFIGLMMHMFYVSYPGQQLIDRSSAIFNDAYNNEWYECSIKSQRLLAFMMLRCTKPCELTAGGIYTMNLENFGSLVKTSISYIAVFASFT

>NvitOR8

MDKLKQSTVDIDTINNIFGNTYFKINKELQELVGLWPYQKGFSVRVVQTIMLFVLSFIMIPHLNGIRVWCGKDLGICSENIAATIYLSGCFLKYLVVLLCKRDISKVYEKIAINWLTINDPNERVILDKFSSLGKLKSIGYTVYVSAAGIGFSQFALLPFAFDYFSPLQNGSRPKIRIVRAEFFVDPIEYYWHIYATYCIVTFVSAFTIISIDTSYTAVVHQNLGIFNIVKYRLSLAKKAVGTSKDLAYEQIISAVRLHQDSLGFNNLIEVTYRVCFLLLILVCISFLTFGAITILENSDNWIDIVRLGSIEVGAVIHLFYLSWPGQLVVSESEELYYYTYNNEWYNLSAESKTLLHFMMLRCINPCCLTAAGLYVMNFENYGAIIKSTVSYITVLSSFRE

>NvitOR9

MEEDIQTYKVCLQNVVICLIFSGVWPATRPLLKRIAFFVTFFSTFSIMAHTLNFSLHNAQNVRILVRGLAAASSFLSISSKVFLFFQHQDDLVYLNDYLSKKFMSDMQNPENLPDLLSNVRTFAVFVRMYKTTAAFIASMYSVVPIIAFLKYGKYLRVYPCLYPFSYAPGGVVHWLLYGWESAGALSAWAITVGTDCIFGMYAIQICGEQRILARKLKDLRVGSNYKKQLRDCMERHHFIITVKNKFEDLYGLISIWLAISGAIVLCSLIFQVTEYLENDGGHVRAIIFFAHFSSKMMQVFMYAWYGNLINEESLAFPRAIYSSHWTDCCDTRFKNDILIVLAQRPLIVTALGCMNVQLDMFAKIVQSSISYFFLLQTLKAKGEEK

>NvitOR10

MEIFDQHYFSVNKALLKSTGLWPYESRRRKFCIRTFINLILGVFVIFPQLVRIYNYFGVNMDMVLEHAAILLFILTTYLKFLTSVYYEEKLKVVYDNIAKNWQAIKDENEVNILSQYSESGWFLTISYIMYIVIAASAYSLLPMAPVLLDMIDPLNETRPRLYILGGEYFIVDNVEDYGKVYAFELVPAAVTVWLICAVDSMYAASIEHCLGLLAIVKLRLQMCTQPSCDSRKDVSYRLIVQLIRLHKDIINFTDILESSYSSSFLILVGVNVLFLSFECIIVLTRFGQTMELIRYSMIMVGIVVHLFYLSWPGQKLTDLSIGLFQDAYLNEWYTCSTRAQKLLNLMILRCSKPCQLTAGGIYVMNFSNFAKIVKTSMSYMTVFASFR

>NvitOR11

MLTMICTADGCLCDMYMHIILCIRYFVFNKRFQMALGIWPYQSRVKNSITYAGLVLVMIIMLIPQFIRLNTYLGKDIEKTMENIFIFFYVFGIFVKLFTAHFAEDKLKILYESTAKNFETYTDAVEAEIMKRYSERGRLLTFVFLLYMISAVAVSVVLPMCPIVLDSTDPLDQPRPRMFILNGEYIVDKYEYRLRNFNKSCGLQMVEHAEAERYGGDYAYAALVRAILLHKDIIKFTEIIQTSYSLYFLLEMGATIGILTSSSVVVVMKLKQPLELLRWSLFLFGVILHIFFLTWPGQKLIDFSSDIFQEAYLNDWYKSSLKCQNLLKFMSLRCSRPCELSGGGLYIMNFINFATILKTSASYITVFSSV

>NvitOR12

MQSKERDKPKVLDIEYYFDLNIRVMSLIGLRCDGPKITGFVHRIPTYTSNTIAILILIFEICLMSDPVCSSNMELTIQTASQTVSNIQCVSKGFLFVNAIEKLQVVYNELQVLSQKYPLEDEIQVLVFDIAEKTMNFCKYYAIAICSCILFYYTPIVVNVIVYILQDPSTNHTFDFTQTLFYLKYPFTIKTFPIYSTIVSIEAVNLIAQGIFWFLGDTLFAQVTTHICIQFKILKHDIQKTFNDEGSKSKEILIGLIKRHRQLISMCMLTEDIFSPVIFSVMILSSTNLCVNIIGASTAINDGDYMNAGVYATILLITVFQIFFYCIFAEKTTEETRSLADTVYHLNWAMKDHHIRLHILLIIMRAQKPFYCTAYGFFPIGHQKLTSILSTAYSYYMMLRTTANV

>NvitOR13

MDYKMQTKEETIEVNAQYYFSLNLNLMSMIGLKCNMTENVGRFYHRIPTFITNVCALMYQSMTVYYLVEAISAKNTSLSIQIISQLVSNIQCFTKGFFLAFGINKIQFILQEKQILWKKYPPNNNNHHTILGIAQQTLTFCKFYVVAIFSCVMSYDVPLAINIFMQYLKRESTNYTYDLSRRVILVKYPFEVTEISTYVILCLQEALFVFIQCIFWVNSDTLFAQVTTHIGLQFKILKCDIEAAFNRDDAKNKEILIELVNRHRELLRICMLIEDVFSPIIFCTVFLSSINICVNVIGVRETISEKAYLDTGIYFTMLLITLFQIFFFCIFAEKLTEETRSLADAVYNLNWTIKDYKLRVYINLIIMRAQKPFYCTAYGFFPIGHQKLTGIISTSYSYYMMLQTTDK

>NvitOR14

MMKMKQQGLVADLLPNIRVMQGVGHFMFNYYSEGKKFPHRIYCIVTLLMLLMQYGMMAVNLMMESDDVDDLTANTITMLFFLHPIVKMIYFPVRSKIFYKTLAIWNNPNSHPLFAESNARFHALAITKMRRLLFCVAGATIFSVISWTGITFVDESVKRIVDPETNETTIIPIPRLMIRTFYPFNAMSGAGHVFALIYQFYYLIISMAISNSLDVLFCSWLLFACEQLQHLKAIMKPLMELSATLDTVVPNSGELFKAGSADHLRDSQGVQPSGNGDNVLDVDLRGIYSNRQDFTATFRPTAGTTFNGGVGPNGLTKKQEMLVRSAIKYWVERHKHVVRLVTSVGDAYGVALLLHMLTTTITLTLLAYQATKVNGVNVYAATVIGYLLYTLGQVFLFCIFGNRLIEESSSVMEAAYSCHWYDGSEEAKTFVQIVCQQCQKAMSISGAKFFTVSLDLFASVLGAVVTYFMVLVQLK

>NvitOR15

MMMELELLRYKAYTQNVIWFLKLAGLWPESHPVPKKILSTITLSSILVIVLTVSNFSFHNLGNIMVFTSGMCMAASSTSAFSKVALFLLHREDVVYLNKHLSGGFMRDMDEPDNRPDLLSNVKTFERFMVTHVISVAIAMFTYSVRPLLVLRKHGKYIRSFPAVYPFAYEPGGLVHWILYAVEVSGTASLWTVTIGVDCVFGVYALQVCGELRILSRKFRELRADDNYKEKLKDCIRRHHVLINAKNKLENIYGLISILLITSTTLVLCSLVFQVSELLAYGASLRLSYSKVSANFHLRVVRKPHSRRERSMSGCNVRLALDGLL

>NvitOR16

MEAKLAKYARYRNVVRRLLLLSGIWPHLEDTCRLYRVLTFSATFVIAALGAKVFAYCIDNIAHVSLFAKGMSNAFSFYTSVLKVLCYLVYRKDLVMLNDCLGRRFEDELKREDRRPLLLQSISVYTRFMCIVAGLTATALVFYTLVPLVFIFKYKKLTQIYQGRYPFAVEPGGRVYWCVCFVESISVVFVWNVVCSVDNAFGLHSFRMCGLLRSLADRFAKLQPDDPGYIVELRDCVRTHQLVLRAKEALQRVYGLVVLWTYVTSAIIMCSILYQADQAKKHMTVTRVIFFTSYITLKLLQSFTYAYYGSLVSQESEKCQNAIYTSNWPGSGDLRLMKDVLIIQSQRPIVLRANGFFIVSMEMFEKIVNTTISYFFLLQAVEEK

>NvitOR17

MKMTAINPEDYFGLNIKLMSLCGLRCSMTKTIGSFINKVPTFLANLVGIIYLVFQATFVMEAVRLRDVALTSQILSQLVSNIQCITKGFLFAVSIEKMQSILYEIRSLWERYQPDIEIQESILDDADRTLNFCKYYVIANFSCVLAYALPLVLNLFMQYQARESTNHTYDLSQMILLVKYPFEVTKVSRFIILVLLEEYLLVVSVIIWVSSDTLFAQTTTHICLQFKVLKQDIEKTFNYGGPNSKEILLKLVHRHRELLRMCMLLEDVFSPIIFFTVFLSSVNMCVNVIGTRETISDKTYLNTGIYATILTMTIFQILFFCIFAEKISEETTSLADMVYNLNWTAKDNQLGFYIYFIIVRAQRPFYCTAYRFFPIGHQRLTSIIRASFSYYMMLQTTDNK

>NvitOR18

MKMTSINPEDYFGLNIKLMSLCGLRCSMTKTIGTFINKVPTFLANLVGIIYLVFEATFVIEAVRLRDVALISQILSQLVSNIQCITKGFLFAVSIEKMQSILHEIRFLWQRYQPDEEIQESILDNADRTLNFCKYYVTANFSCVLAYALPLVLNLFMQYQARESTNHTYDLSQMILLVKYPFEVTKVSRFIILVLLEEYLLVMNVIFWVSSDTLFAQTTTHICLQFKVLKQDIEKTFNYGGPNSKEILLKLVHRHRELLRICMLLEDVFSPIIFFTVFLSSVNMCVNVIGTRETISNQTYFNTGIYATILTMTIFQIFFFCIFAEKISEETTSLADMVYNLNWTAKDNQLGFYIYFIIVRAQRPFYCTAYGFFPIGHQRLTSIIRASFSYYMMLQTTDKK

>NvitOR19

MQTNTEEKAITAVDAEYYFDLNIKLMSLIGLKCSMTETVTKFIYKIPTFLTNVLGIIYLIFQISYVREAVRSHDTSLAAQILSQTVCNIQCNSKGFLFVISIAKVQAILHEIRILWETYPPDDEIQKSILLVADKTVTFCKYYVTANLSCVLAYALQMGLNFFMQYQAREATNHTYDFSHIILLVKYPFVVTEIPTFITLFLSEEFLLIMGATLWAIIDTLFAQVTTHICLQFKILKRDIQEKFNTEGSNDKEILLKLLRRHRNLLRICMMIEDIFSPIIFFTVILSSVNMCVNVIGARETIASKAYFETCIYASIFLMTIFQIFFFCIFAEKLSDETTSIADTVYDLNWTTKDYKLRLYLRFIIVRAQKPFYCTAYGFFPIGHQRLTAIIRASYSYYMMLQTTDGK

>NvitOR20

MGLIEVLESRKIFLWICGLWPKEYQHKPKLSQMKLYFIWFNMLMMCLLVFAGLVAIATPDNIPQASIRLPRKRMMKVIMETLKLEETSKFENDNDLEIIRSWRRTRDGVLKYHMRIYGFISVAYCFLPIISQVNTYPAQTIIQASLFVSPWYEFFYGFHCAQLFLYLFIIIATDGLSMILIFKLCEELQRFECLLFERHRADDATLSEKYKSRGEFLRCIIRKHCTILDYGESICNLLTGALFTQYFLLSGTLCFSVFTILSSNSSAMANQMSIMAGTCIVQLFMISLAGELVSTRSLALADALLQSDFCCSIFGELKSSELRQTVLMQLRMQKPLKLSIGTLGVINIEFFSRIMKGVYSFTMLLRTSYV

>SinvOR1

MFANKYYERDIGNAFAMHRFFFRMIGLWPFAHANSLLPEQLETIVVVFVCFACLIVEAVPTLLYVFMVLTDIRVKLKVMGSAMFTTVEIMKYVYMLFYKSQMRNCLILVDEDWQNVVSPSDRTSMIEKVKICKRLVVLCAVILYSLNIIVRIVIPLSVGKIVTPQNITIRPMPHVAYLVILDVQQSPVYEITYIMQLLGGFFKYTIVVTTFSFVTLCAMHFCSQSNILITLINDFVNESRPENLNKKLSIVVEHQIRIKNFLQLVQSVTQYPSLVEVLGSTVMLCFVGYCIITEWEDQNILRLCVYSLILVMFVFNVFIYCYMGEQIIEHAGQVALTACTLEWYRLPDTQARALILLIIVSETPFKLKAGSFIDLSLRTFGNITKMALTYFNILRSLL

>SinvOR2

MPQNGQHQNDILYITQPTRKILRALGAWPSISKGRSIYPKAHNLLLICIAYALLSSDIIPGSLFWVMEKTTRIKLQIIPALIYDFMSAIQYGIFIVRYDQVRRCLKHVEEDWKNILSADVRNIMLRSGRTGKRLVTICGAFMYSGALTFRTILPLSMGKTVTDQNVTLRHLACPGYFFSLDVQVSPVYETVFIIQLLTSIVTVSIVTAACGLTAIFVMHACGQLKILINLMKSLVQKQWLEEREVDKKLSEIVEHQIRVRKFLRLVQHTLQEIYLMEILVNSITICILVYFMSMDWQNRNVTSVCSYMISIANVTIHMFLFCYTGEQLTSQAEKVAIASCELEWYRLPDRRARTVILLMIMSNAPTKISAGKFVDLSLKTFGDVVKTAGAYFNMLRNVIE

>SinvOR3

MIPISTISRPVEIGLRLTGIWPNSLVLFRMLWTLVMGIVLIFQYHYLLIHFSTEELPNLIDGLSTTLPYNLLFIKMIVLWVNNRIFNDVLKAMSNDWREYSGMYAMIDKAVLAHRCSKLTIGVYSTAVLLYSTASINFRKQSNDSCRELLIKMELPFNFCESPVYEIVMWVQFVHLMAVASSIGMLDGLMVTLMLHIGGQIDLMRQEVEEICPNDDKYDLPITIVRSLINKHQKIIAFTENIESLFSHIALMQFFSNTIIICCIGFLIVTSMGTDEGIRMLIKTMFFYIAITLEAFIFCFAGEYLSNKSKTIGDAVYESVWYNLKPRDCRVLLFVIMRSQKRLTITAGKFMELSLQGFTNSMKASASYVSVLYAMY

>SinvOR4

MINRKQSTLAVDVTKQNEAYISDFRYAVQISVWLLKPIGVWPLFNETSRLKIALHKALMVIGTFIVLFMVVPWTVYIVKKKLDVFLIIRTICPLLFSTTTSVRYILLLWNQDRLKFCMEHMADDWRCATIAKDRDVMLANARDGRTFGIISMVFMFSCGGLYYTLPIVMPNPINENNVTVRLHPSPSELLVFDTQASPVYEIVYFLQILSGYTIYSAFSGTCSLIANFVTHVSGQCDLLTTIFEETVDGGEHNSGSIENRIATAITRHMRLLRLVSDVSNLFTEICLVEFINASCNICLIIYYIVTDIRNNEPFMPTFMFGFGLMSIIFNLYMVCYIGDLLKERCQQVGNACYAIEWYRMPYKNAMRLLMPIAMSQYPTTLTAGKMVTMTVTTFSDILKTSMAYFNLLREFTSQDITKT

>SinvOR5

MLENFLREYNINRILLSITGLWPYQNKVVRSLLWIFCFLLEISYYPFEILLFHDHSDDAQLIFEGGYQTLILTIFVIRHLSDVLHNDKMRCLYEAIDEHWNIFTDDIEIRILREYSVLSQKIVIYYSMLHLSSLVVIIILPLTPIFLDILMPLNESRPRFFTMEVEFRVNKDEYFLPIFCYTNLVIVVGVFIALGFDSMHITCTAHACGLFAAISKQIENVLLKVNDNNNIEKTEHVNKKLELLNEEITYRKYIICLKKHQLAIEFVEILNSSYQECALAILIVLLGILSLVGIRIIYVLHELGTLIKFVFIFITAFCVLLVPCYSGQRIMDESQNIFYRAYATEWYKFSHRLKSLLLITLYRSNKPCGLKAGNMIPLSIATYATVVRMSMSYYTALLSMQD

>SinvOR6

MQILALNFLFYTIGGVWRPIKWSSKCSICLYSVLNFFSLYLLTFFVLTQLIDTIFIVDNIDDFTTNLSLLLSAIAVYCKAVTATARRSEFISLIKMLQEKPCKACNEEEINIQMKYDRLIRSYTMSYSILASFSLTGITIGEVLIALQGELPIRAWIPYDYTSTFLFWLTSLKLIVAMALSTFVNVATETVILGFCLQICAQFDILICRLRKVIESDEKQENELNSATNKTSRLSENIHYHLYIIRFAKMVNKVFSQIVFIQFFVSILVLCTSVYYLSSHKTVTDFIKLGIYTSCMFVQIFMYCWAGNEIILKSIGLSEAVYKMEWILLTISERKDLLMIMMRSTKPIKFTSSFLVTLSLESYATILKASYSAFNILQ

>SinvOR7

MQILTFNFRLYTISGLWRPIEWSSKSSKLSYSVFTITTIYLLTYFSVTHLLYIIFVIDNVEEFASTSPFFFSTISLLFKASTAVIYRNQIINLIEILQNKPCKAENKDELDIQMKYDRAIRTYSMRFVLLCSFSMTGAFVAGLLDVLKGQLPYNMWVPWECTSLFTFLSTSIQEIAGVTIATVVNVATETTVLGFSLQVCAQFEILKHRLQRMMENSNEKMSPKNSLNNGSHKINKLATHIFHHVCIIRLAEKFNNVYSQVIFIQFFVSVLVLCSIVYHLSSHLTVVDVSTWVVFTVSMFIQIFIYYWSGNQVIIKSSELGEAVYHMNWMSMTLNERKDLMMIMKRSSKPIKFTSSFLVTLSLESYTNLLRATFSAFNVLQQF

>SinvOR8

MLLHFIIVFCLSGFCLLLEMEAITTKIYFSIIMLTILLINTFLYCGAGELLTEQCNAVYRAICNLEWYKLESRNAKNLILLIIRARHPCCITAGKIIPLTVATFSTVLKTSCGYITFLLAKRA

>SinvOR9

MFLLFLLVFVGIIPGLHSLVRTWGDMMATIDNLQFTLPLLTTIIKLVVIWWKKPDLVLVLNTIANDWSNVKTDKELHVMTKRAQSARVVTIFGYILMVVAMFLLICLPFFGTSLRYRTNITDPDKVLPLQTHYFYDKDQSPYYELTYVAQALLLLMSSASYTGVDNLLGLLIFHLCGQMENLKERIINTKQFNNFNGDLAVIVNEHIRLIKFFDIVESTFTVLLLGLLLYFGTLFCLYGFLIIAVLTEGREMSMIRLIYLISGALNVCGHMCLYCVVGEILVAQCEGIYRAAYNYEWYKLNPEEARSLIIIMIRANKTLHITAGKMFPMTLSMFCNLIKTSAGYVSILLAKQG

>SinvOR10

MSTFTFIVIFFVGTIPAIHSLVRTWGDLMSMIDNLQVTLPLTMAAIKLIDIWWKKTDLLMAINMIAEDWIKDKTSKERCIMIKQAQNARIITIVCCCFMFLGSTLVVILPCFGMTVRYITNVTDPVKILPFQTHYIYDKNQSPYFEIIFAAQFLVALMCVASYTGVDNLLGLLIFHLCGQMDILKEKLINIKQFKNYNDGVALIVKEHIRLIKCFCIIESTYTLLLLGQLIYFGIIFCLYGFLILVILTEGKHMSLMRFMYLVSVAINICGHMCLFCAVGEMLISKCDDLYRAAYEHKWYKLDPKKAKILVLIMIRANKPLYITAGKMFPMTMSMFCNLIKTSAGYVSILLAMRS

>SinvOR11

MSGTASMIMGYLVYACGMFRIASYRIERAMTINNLYKNFEHETEIYKGIINAVNIHRKTMKICNYFMSSFNGTWSVLIVVGVLSLSLNIYQVFHCVLFGRNKQEFILHFVIVAACLLYMFLTNYFGQEIIDHNNHIFITVYNGDWYIAPLHVQRLILFLLQVGNKTFGLRVGGLFTGSLNTFASVKNILVTIKYTGCPIKIHD

>SinvOR12

MLKYAEDKFWYAQYDAHGSKLIKEINKRALILTWTFKLGVQATCISYVLIPLIENVGKKKTKRELPFGFYIELSINVSPNYKIKYLVWRWCEGVQTGSIRKDQCIISYQDLLWFCEKMEQNFIYLCLCQMLSSNIMLCLAGFEIFIEKGRKCDFPQTTELHYPYKRRFRLTVRDHADGDLMDGNRAIEDVAYSGNWQVLSHKGRKVRKVILFIMMRSGRVCSIFAGGFFPVSLETFVMVVSIAASYFTLLHHFAE

>SinvOR13

MIFGCWRPHSWSSLHKRLAYDVYTSTIFLLVNTFMISQLVDVILTLSNAEDISDNFFTLIALCSACCKLLVLLINRKSIIMLVDILMDKPCRPSESTEIKILYKFDKSIQINTWRFIHLGIVTVSIVILSSLFMNFRNKKLMYKAWVPFDYSSAILFYITYTHQMIGMIAAIFLSIGCDTFICGLLFHICCQIEILTYRLKKIISYSSVLRDCVYQHYHIFKFAVIVNAKFSLTTTVQFVMSTMMICFSLYHLIEKIFEIKWMTLNENSKKSLMIIMKRALVPIQIKCAYTIPMNLNSFMNILKMSYSTYNLLQHMDK

>SinvOR14

MQVMQFPFKILTVAGCRPPTSWSSSLGKRIVYHAYTIIMCLLLFTVMVPQFMDIILNVNNADDFTDCFYIMLATFIACCKILSLLLNRKNIELLTEALVEKPFRPLEPDEIEIREKCNKTVRTNSIIYTVLIELTCGSMNLTSLLTDFRRGKLAYREWIPYKWSDTVYYFTYFRQIISLTVASIVNVACDILICGLLLHIYCQIEILECRLKKSLRNRGDLGECVRLHDRIYKYARTMNEKFRLIITVQFIASMLVVCSSLYRLAKTTLSPKYIPLMLYTICMCIQILLYCWFGNEVKLKSIQFSDEIFGMDWVTADKKARQSLILIMNRSLLPIEFSSAHIITVNLDSFVKLLKMSYSVYNILKQTKEE

>SinvOR15

MVQTRIKKGKKSYFTNDTSPQSLLHHCRKDYSINIDYFLQYIENIVWLHVFLTNTTWLIPNYIYGFFRPEQFNLLAFRTVGLMRYSIVHTSTLNLPLSITISVVFCLCGFLITIIFNDRKINETALAQVYLSTTILLCLLINTFLYCGAGQLIIEQCNKVHYAVCDLEWYKLEARKARNIILLMMQTSHPFCMTAGNIIPLTMATFVNVLKSSFGYISLLLTKHS

>SinvOR16

MSFYDNHHYYHFNKTSLCIIGQWPFQSRLRNNVMFALAVFFISSLTVLEFWGLIAGITDLSIIMENTSPLLVNNFIIIKLTSCWLNQYNMKDLLEQVKETWKVIHKGPENEILQYYAEATKNYSMRYATGLYAMWLFYCLPPVIITKIYTLLPTNETYSAKFLYRLEHVLDVDKYYNLLMLHAFISVFYIVSVPIAVDSMFILCTEHVGALFGCIKYNMQRIEDSELFLLNPNIAHDEAYDIIVSCIKLHKRILKFFDRLASTYAMSFLLLLANVVICSSFNMAELVMVDNQPDEIVRILASNAAQLAHIYYLSATSQRLTDYSTEFQEVIYSCKWYQISLRSRHLLRLTLLRSTKPCQIKAGNMFIMSLETFSELLKMTMSYFTMLTSMHE

>SinvOR17

MKSNVKWNTDTTNILKFHKNFLGIIGLWVLNDKNIFSRIRWFLSTMVEMSTSITMSIEMIRHCNGHEDAMDAFLLSSSSLISMVKLLLHRVYWRQKLILVESVIHDWTYVKNSHSRDIMLKYARIGRLGSSIFFYFGCASVVSFVSSVVLANVDLPWTSGKQTFNETYERKLMLAAYCIFGKDTSLFAYCAIEALQFVQIVVNGISQCGNDGFFFDLTMHMCGQFAILRMNFTKLGCEDFSYRSKLNILLKRHYQLICLSHYLERAFTMIILAQVLMSMIVICVEGFLLLLSLEMNDALTAAKHSVFILSLCMQLFLYCFAGQTLEFQSKELACAIYESPWYTFDVSMMKTLPLIILRTAHPQQLTAGKFVAINFMTFKEILKASASYLSVLRVMIKT

>TcasOR1

MMKFKVTGLVADLMPNIRLIQASGHFMLNYHADNSGALHTLRLGYCCMHLVFVLVQTFSCNFVNLVLERGDVNDLAANTITVLFFTHCVTKFVYFAVRSKLFYRTLGIWNQPNSHPLFVESNNRYHGIALKKMRRLLYIIIIWTSFSAIAWTGITFVGDSVHNIKDPENENLTITEPIPRLLVKAWYPWDAMSGMPYYITLVFQVYYVFFSLAHANLLDSLFCSWLIFACEQLQHLKEIMKPLMELSATLDTYVPKSADLFRAPSATSQDQLIENGTNPAKKNEDLKGVYSTRQELGGHFRGGALQNFGSGGVGPNGLTKKQELMVRSAIKYWVERHKHVVRLVTAIGDAYGVALLLHMLTSTIMLTLLAYQATKITGVDKYAATVLGYLLFALAQVFHFCIFGNRLIEESSSVMEAAYSCHWYDGSEEAKTFVQIVCQQCQKAMSISGAKFFTISLDLFASVLGAVVTYFMVLVQLK-

>TcasOR3

MKLSSVTTCLFSSDFHTRMNFDWKDTIKLNFLMMKIVGLWPKEKYKINFYTLYTLISVNLFICGHVIFHTVAVFVVGRDLKHLIGALYMSLTETLLLVKICYFIKNSRLVKSLLTSLDGDIFQPKNEKQLELTNPSLIFWKKVHKSFAILVANTVFLFVSLPILSKSTKLYRLPLEAWYPYNTQKSPNYEITYLYQFISTLFRGMASVSMDTFIAALNMYIGVQCDILCDNLRNLNETNFMENLSLCIKHHKAIVSFARECNKFYNGIVLGQFFSTSIALGLAMFLLSLVTPLSTESNTLLFYLGATTSEIFLYCWFGNEVDVKSSKIPYSAFESDWTGAPIEAKKNLLIFILRTQKPIKMSAINLFSLSLETFTTILRTSWSYFAVLRQVNGQA-

>TcasOR7

MNKLQKFDWKATIRPNIAFLHYLGIWPEGEEYYKLNFYTLKTILYIIILVISTIVFQVINIFFTLDDLTSLTANIYVLLTEILYFIKLCFLVKNMPALKLLMKTLDHKLFQPKANQIVIIQPLLNFWKLIFLAFVITCSFTVLFWAIFPILDSSEEEKRLPLLAWYPYDTKISPNYELTYLHQVASYIYICYSHLNIDTFITALNTYIQCQFDILCDNLKNIKSDTKNVDTKLAKCIKHHLLILMFANTSNEFFSWIIFFQFTSSAAITGMTLFQLTVVKPFTTEFYNFMAYVTAEVVQIFMYCWFGNEVQVKSSNIPYAAFGSDWTEFSPNKQKSLLFLITRSQKSVKMSAFNVFDLTTDSFILKSAWSYFALLNQVNS-

>TcasOR17

MDDFNWISTVKTNLLLLHIGGIWPRGDGTHKLNLYTIYAIFITFTFTTYHCFSQIINFFFVDDLQALTESIFISLIQSMALVKAFYILKNMRILKNILKNLETNKMLQPRNLKQIKMVQPSLTQWRLLSQMFWISAVFAMCLFGAFPIVESTYKEFRLPYLAWYPFDTKSSPFYEIMYLHQFVSSYTIAIVDIGADTLIAALNVFVATQCEILCDNIRNINGSVEEMDSKWKECFTHHKEILKVARHCQKFFNWIVLMQFCASVICIGLTMFQLTLVVSFSSEFFSSLFYFGAITVQIFMYCWFGNEVELKSSKILYATFEANWVEAPHQVKKNILIFAIRCQNPIKMSSLNVFYLTLETFMAIFRTSWSYFAVLRQIQNRISEE-

>TcasOR20

MNSFNWQESIKTNLKALRLVGLWPKSDFYKFDLYTFCTSLTVGVIVCGHNLSQIVYILQVYSDLKALTATIFVASINFLGAVKMYFFIKHIKTVKILFKMLKTYQFKPKNIHQTQLIKPFLNLWKILYVGYSINVYLIVAMWSLLPVLNGWTWQKKLPFPARYPFDVTKSPYYELAYVYQFICIWYITVANLNLDTINIALMMYTSCQCDLLCDDLKNLTETRFFHKKLIECIKHHKAILVFAEKSNGLFNMIVLSQIATSTVVLALTMFQLSMVSPLSSEGLNHLFYIGGIIMQILLYCWFGNEVEAKSSNILYAIYESTWFEASKNSKKNLLIFSIRCQRPIKATAVKLFALSLRTFITIVRSGWSYFAVLYNVGSK-

>TcasOR24

MEEDFDFLSSLQTTLFCLRCVGTWPSNTYKLDAYTLYATASITICLFGHNFFQTVNIFFIFNDLNTLTGVIFVALTCLVAILKSLLFIFNMRRLKKLLLVDIRQKLFKPRNRQQVVMVQSRVNFWKKIYFMFTGMGVATMFFWALFPIMDGTVKEHRLPFLAWYPFSVNKSPFYEITYIYQIVSVFFIVIVNMNSDMLLVALMNILGVQCDLLCDNLKNIQFRERINEEFLRCVNHHMQILSYASDCNKFFNTIVLAQFFTTVVSLGLTMYQLTIVTPFTSEFYSFIVYGGAVLMEIFLYCWFGNEVEFKSLNIPFASFGFDWTIGSVGLQKNLIIFIAKSQRPIRMSALNLFHLSLETFVKILRTAYSYFALLNNVNSLN-

>TcasOR46

MSKSEKIHTLATYFDSNIAFLKLTAFWIYDDETTRRKKYLQHAYNIFWIFYLFVAYQPAELLYVYYSFNDLSVFLRALRDIGNHVSLAYKAFNYFIMRRDILKLMETLQHGNYHYEDCGDFQPKLIVDEEKKEALKWTKYFLNFCNAICLSMFANGVFTFIFLSDKQYVERNGQRVYHQEQPVNTVSPFGSGTKLRFFVTFIYTMIALTFYAWTIVALDSLFITIMSCISSHLKILQGAFKTVRARFIKLCASLSKLLISVSGKLESIYSTQTFVQTFISLGEMCFSLYLLSETADQNIGNEITYLIATGFELLMYCWFGNRITEASLKISYALYESDWFPTSLSFKKQIIFTMTRMQKPINVTIGKITPLAFSTFLTIARGAYSFFTFLKQRHGINH-

>TcasOR58

MPFTIKDYDLRNAFETERTLLTLSGFYPRRTKKYNFFYNTSALINLFIAYGQLFSMVVQMVIDRNELSKLSETLLFFMTHFTFLCKLTNFVYYKKKMFEIEDNLSRKIFYGFELWQIKPKIDSCKFIAKIFRILCILVVLFYTLVPYLDDKEDLSLPLPGWLPYNTKKYYYPTVIFQVMSVSVSAYNNSSIDVLTCMLITVASAEFNLLKGALKTIDFHPKGHNTKQLIEAKFENCVNHHKEIVKFAYQIETIFSKGIFLQFFASIIVICFTGFQMIVVPIPSMQFIFLIIYFSCMMCQVAMYCWYGHDIITTSDSIGQAFYMSNWYESDVKIRKNICIFLERTKKPVILTAGKFVTLSLTTFTTILRSSYSYFAVLQHLYKEDS-

>TcasOR59

MDEEFLIGTFETEKKFLRYGSFYPCGKRIKFIFLGLFMFVYSWTEFLSMITVLFVERDNLTKLSETLLFCMTQAAFLFKLVNFLYHNKTMLRIESILKNPILNCLDQFEKNIIEKYMIRVKYLARLFRILCILTVSFYGLFPFIDEDPDHMLPLPGWFPFDVKTHQIELVIAQTCGIAIGAFLNSTLDILPTILITLGSAQFDILKIRLENITSVDTSKSWLVKKAIKKCVIYHTILLNYITQIEILFHKGIFVQFTASVVVICLTGFQMLVISVRSIQFILLMIYFSTMTCQIALYCWYGNELMYRSMGLSDACYMSEWNKCDTSVCKSLAIIMERGKRPVVLKAGNIFSLKLTTLMTVLKSSYSYFAVLQRLYATSE-

>TcasOR60

MSEDYTFRNVFAREKKILTISGFYPLREYEKNYFHFFSGTIQWIISLGMLFSMIIQSVIKRNDLMVLSETLYFLTTHLTFVCKLANLEYHKKLLLDIEDMLKTTRFQKTLSLDLIEKTGMNEKIRKFNLVAKTFRIVCVWCVVLYVLVPYFDPGKSKTLPTPGWFPFNWTDKYYYGTYFFEVAGISITAHMDSSIDILSWLLVTIASFQCDILKENLKNIYYNYDKEHDIRETFKDCIRHHEEIIKFTTKVEQSFSQGILLQFLCSALVICFTGFLMLVVPVLTFQFANTIMYFCCMMIQLGMYCWYGHEIMTTSDEIGQYFYLANWYDSSLTLRKDFAIFLERAKRPITLTAGGFVVLSLNTFTRILRSSYSYFAVLKHLYNKS-

>TcasOR61

MGDYDFRAAFAFEKAIFSLSGYYQRQAGFSSLIICAIASLITIAQFLSMVMQIIVAGNDLTVLSETLLFFMTHFTYMCKLVNLLFYKSKLLHIEDLLSRPRFYGFSQNELTIIKDGIEATNTVANLFRIFCVLACIAYGLVPYLDHTKAMALPLPGWLPYDTTKYYYPTYFFQMVAVSITASVNSTIDILTWKLITIASVQFDILKRKLKDLDYKLETTSLQIQFKTCVKHHKEIVNYVKNVEKTFSKGIFIQFFASVIVICFAGFLIIITPVLSMQFLYLTLYFMCMISQVAIYCWYGHYVMTTSDEIGQDFYMSNWYESDVAFRKDIIIFMERVKKPVTFTAGNFITLSLVTLTRILRSSYSYVAVLQHLYNEV-

>TcasOR63

MGFMIQDYDLRNAFSLERKLMLVVGFYPKRDNKHEILYWLSAFFNLLISYGQLTTMIIQMVFDRSDLSKLTESLLYFFTHFTFLCKLLNFQYYSKDLIEIENFLTDPIFYGYSFEQLDIIKAKIRSCAFISNAFRICCTFTCSFYCLVPFIDESRKKILPLPGWFPYDTTNYYYSTFFVQSLSLFISAYCNTAIDILTWKLITLASAQFEILKENLTKIDYEGGFNETKGALVRCITHHAKIVNYTERVEAIFSKGIFLQLFGSVIVICTTGFQLIVVPIPSVQFAVLGTYLCGMTTQVATYCYYGHEVMTTSDAIGMSLYLSNWYASHVKIRKIVMIFLEKTKKPTIVKAGNFITLSLATLTQILRSAYSYFAVLQRLYKDS-

>TcasOR64

MMSDEYVKDVFIANRWMLRCAGLWTPSTRSKLVQIPYKIYAIVVFLFVNVYFTSTEFLSLFYTHKNLYNFIKNVNFFLTHFMGAVKVIFWFFKGHVLRDLMRTLESPEFHYEPCEGFQPGLIWRKYRRIGFKYSLGFLALAHMTLSSSYIPPLLTKLPYFSWMPFSYSTPRSYLLALGYQAGPMFSYAYSIVGMDTLFMNIMNFIAAHLVILQGAFASSKMRVLDPGQMNNEMKRNCRHLQTILRVSEDLERVHRYLTLGQLTATLFILCTSLYLISTTPASSKQFYAELVYMVAMGFQLYLYCWFGNEVTLMASEIPVNVWKADWYDCDQSFKKSMIFTMTRMQKPIYMTVGKFAPLTLQTFVYILRTSYSIFAVIKNTSI-

>TcasOR65

MTATKSLKEIPPIYLRVHLTVLQILGIDILPVESVPQNLFYTYTALIISTMCLFTIAEFLDMVLNYEDIYRLTFGLCYCVTHVLGTVKMFLMLYLRKKLWGNLTTLEEGIFKPNPTRGGPEELQIVNDAITMCNRQGYVFYTLVFLIIGARLLYASLANWPYDKHNYFDGNVTVIVNTKEMPYTTWMPFDYNDSPLYETIFAFQIFSTTVYGFYIGAADAVICGFMMLIKAQFLIVKRELETLIERAQKAAIAENPDNEDNFGREIERIELLDKRTQDYVAKYANECVYHHQELIALCDHAEEDFCYLMLLQFISSLLIVCFQLFQVSTLSPDSVEFFSMVCYLLLMLFQLLCYCWHGNEVQIVSGELSRYAFGINWIIMRESPKKTLLLLMMRAQRPCYFTAGKFSLLSLQTFMTIVRGAGSYFMFLRQMNI-

>TcasOR66

MSKNLKEIPPVYLKVHLTVLQILGIDILPNERIPQTLFYTYSVLLIATMVVFTTAECLDLVLNYEDIYKLTFGLCCCVTHVLGAAKMFLMLYLRKKLWGYFTTLENGIFKPNPCRGGAEEFEIVTSAINMCKRQGYVFYVLTVGVTGGQGLYAALANLPYDKHNYFDGNVTVVVNTKQMPYATWTPFDYNDSPLYEIMFAFQIFSTTLYGFYIGAADAVICGFLMLIKAQFLIVKRELETLVERAQRAGNPDRGDFGGGINRIEMLDDGTQVFVEKCANECVYHHQELIALCEHAEEDFCYLMLLQFISSLLIVCFQLFQLSTLSPGTFEFFSMACFLLFILFQLLCYCWHGNEVQFVSGELSRYAFSINWIIMRESPKKTLLLLMMRAQRPCYFTAGKFSLLSLQTFMTVVRGAGSYFMFLKQMNT-

>TcasOR67

MDFTIRDFDLRNSFSLERKLLLVLGFYPIRDKEKHRILHQLSAFLNLLLYYGQLLTIIIQMVIDRNDLSKLTDSTLYFLTLFTFLCKLFNFQYYGKDLIEVEKSLTDPIFYGYSFHKLQIIKAKVRSCTLVCLAFRISCTCSCFIYSVVPFIDRSGQKTLSIPGWFPYDTAKHFYITFFLQSLSLFISAHCNSATDTLPCKLISLATAQFELLKDNLRTIDYENSFEETKHALVKCITHHRKIVNYTKRVETIFSKGIFLQLFASVLVICTTGFQLVIVPFGSLKFAIHGIYLCAMTAQIAIYCYYGHDVMITSDEIGTSLYMSNWYASHIKIRKIMVIFLEKTKKPTIVLAGNFITLSLVTLTQILRSAYSYFAVLRRLYADD-

>TcasOR72

MAKLEYLTGATFTLKCAVLYPIDSNNPKIKKILYAVWAIFFILTFVTGFIQCFVFVCINPFDLVQEAMIIMSLVFYSTTFFYFIVFYKNWQNMVALVTNINKNFHRATDNVIEKISMDQASELSDKLAYVWTSSLAVGSVVPVVLAIATGNLEMPMPAWFPYDYNKSPVFEITYLWQVFCLITLAIIYGASDMFFPCITIIIGQQFKILASNFKNNFYTSLIKLGAEESIVQNFSKDIKTHEFRSFYIKYGNIFKILNNAKFQTLNRAFLKRNIKHHKLLLRFCEDLNKILNTFLLIRVSAIVFNLIFIGFNIIINADFLWTLYECPWYLCDVTYQKMLILVQMRVKRMVSTKAGNFFTMIAPSFIAFQRAVFSYITLLKEVTDLGKD-

>TcasOR73FIX

MTRKHIFLNFTVTILKLSFLWPSNDNYDQWRLVKDASLIVSLMPCALPILAHFVLQITGDVYNMVTITENLIALICIIGMIYMTICFVKNRKLVKTLVKNLPAFTKYSKTTDIILTDKKANLYTKIFVFYGVIGNVVYMIMPYLNIEKCQQRQNNDVPCGLVTRCWFPFKFDYSPVFEIVFVHQFYTCLMVSVIILDLTMLICGFLMHITNQLKHLRGFIKRFDCSSQKIAEDVIYCVKFHTAIITYSEKTNEAFGTMMMLHITLTSLVISALGFEILIVDNFNDSLRFTLHLLGWLVLLLLICYYGQLLIDESIAVAEDIYYVPWHLAPVDVQKDIYMILMRSQKPLTLNAANIGVMSFPTFLRVISSAYSYFTLLLNIKS-

>TcasOR76

MMESTVTRLKRMYLWPTASVTSRKPAFFLITFSCFLLYGSVMHLIVNDISMEEVHVIETTAGQFGVLYYLTLFTIYRKGILEIYADLSNFTKFGKPYNFDKRNKQLNQWSRWFSVVLYFFVISVFAWPGIFTQSCEDLNVALNKTEVCGVVSPVWLPFRFDYKPMKQFVYFWQSFCCLYSNGGAGTISFAMSETIEHLILRVEDLKILFPKIVAERSPEVRRKMLAKWVDYHLWLLSIGKLMNDTYRYSFSVIVLCAGTLFGCIGYTVMKNASTNFNSSFIFFGWMESVFVICVCGQRLMDAFHSVGTTVYNSEWCDTDVDFQKGVILITIRAQKPVRIYAGPFSYVSHLLILTVFQTSYSYINLLNASS-

>TcasOR77

MKYILMKKTIAFLSVTGFWPKTKESTKTRAFCILFSSSFLLFGSLGYLIVYRKFGSDDIDSIETATSHFGVLYFMFFWILKRDGLVHIVNLLSDFSKFGEPRFFNDRNRQLDYLLQYCIFVLSVATGGVFLCPIIFVKNCEMVKQEKNLTKVCGLVSNVWAPFDYSEYPMKRVVSLWESYCCFINFGCGGIMSFTMIKTMEHLHIRVEQLKDMFPDVVNEKNLAVRKQKLEKWVKYHLHLYDIGELMNNTYRYCLSVIVLCVGILFGCIGISTMQPGSSHNSLFLFMGWFQSICILCMVGQRLLDVFLSVGVMAYDSAWYEKDVDFQKAVLMIMIRARRPVLIYAGPFTNLSHLLILGVLQTSYSYINLLNAK-

>TcasOR78

MGHAIMTEILTYLTLMGFWPRSPKSSKASAFLIILSTSFLFFGILFYLIVNRQFGSSEIDSIETITSQFGVLYYLILFTWKRNDIVEIVELLSDFSKFGKPPFFDQRSTRLNYRLSCIVLILIVANIVVAALPVIYIDSCHKANEQLNLTKTCGLIAPVWLPFDYNEYPRKHLVFAWEVYCCVMNYVGSGIGALTMVGTMEHVIIRIEQLKYIFPKILDQPNPRIREQMLKNWVRYHLALFEIGRLMNDAYKWSLSVIVLCVGALFACIGISMLQSTASQINSICLFFGWFPSIAFLCMWGQRLLDSSLSVGTAVYSSRWYDMDVAFQKSVLMILIRSQKPIRISVGPFTHLSMLLLLGVFQSAYSYINLLNATS-

>TcasOR79

MGHVIMNEILTYVTLLGLWPRSRKSTKTISYLIILSSSFLFFGSLLYLVVHRKFGSNEIDSIETVTSQFAVLYYMTFFTLKREGTVRIIDQMSDFSKFGKPPLFDQHNKRLNYLLSYFVICLFVAIVGVVALPAIYTGSCHKANEQLNLTKTCGLVAPVWLPFDYNGYPLKFLVFAWEGYCCIITYACSGISSLVLVGTMEHLIIRIEQLKLMFPEILNEANRHIREQKLKNWVQYHLALFGIGKLMTATYTYCLSVIVLCVGILFGCIGVSTMQSASSNNSVFLFLGWFQSLIVLSVCGQRLIDTCLSVGIAVYNSRWYDMDVSFQKSVHMILIRSQKPILIYTGPFSYLSHLLILSVLQTAYSYINLLSARG-

>TcasOR80

MGHVIMNEILTYLTFLGLWPRSRKSTKTVAYLIISSTSFLFFGSLFYLIAHRKFGSNEIDSIETVTSQFGILYYWVLFTLKREGTVEIVERLSDFSKFGKPRFFDQRNRRLNYLLSYFVLVLMVAIGGVVALPVVYIDSCHKANERLNLTKTCGLIAPVWLPFDYNEYPRKNFVFAWEVYCCIMTYACCGIAALVLVGTMEHLIIRFEQLKLMFPEILDEPDRHTRQQKLKNWIEYHLTLFDIGKLMTSNYTYCLSVIVLCVGILFGCIGVSTMQSASSHNSVFLFFGWFQSIGVLCIWGQRLLDTCLSVGIAVYSSRWYDMDVSFQKSVLMILIRSQKPILIYAGPFSYLSHLLILSVFQTAYSYINLLGAKG-

>TcasOR84

MTEEKELRLCLWSCYYLKLSLMWPLKREEFKSSKGLYLRLLVFVIISGSTFTAMIFMHLYKSLKVGSYDVSEDLAILASNIGYVLMMTMYVSRQKDLELLLLDLSDFKTYGKPPNFDKVRKRMDLYAHLIFFYSMFGSFVYNMDKIILIDKCKEARRINEVCGSAIPFWTPFETEDLFTLTLVITYVLINIFVVVKVAMTVSVQVLEISSHINLRIEQLKIFIAGCFDRDFKASRERLDFCIRYHNVIIDFSERFSRCFSYVMFIHLAITGIIIGCLENQIVQEHQPEAMLHMGGWSTATFIACYGGQLLMDASTSIADEFYNCPWYEADVKMRKDLILIILRAQKALFVSTGPFNVLSFALFVSIMKLSYSIFTVLS-

>TcasOR86

MALNQEDAICSKSCFYLRYSFLWPEEAPTRSFYAKFILVLILSFLTAFLPLFIHFLILVERGLDPSEDLFVIISYTGFALIMIIYVIHVKKTSYLIVQLSDFEKFGKPRGFDYWDKKFRLISSGVYYYVLIASSGLNLGRWVGMAECRKERDFQVCGIVIPYWLPWKVDSWLFFILLDLYVLKMTLVVNCALFLIIIQILEITTHLKLRIDHLKEMLVKCFDSDSQTNRKQLVNCIRYHTYIINCSKLFKKCFTHAMFSLIVTMALSCGCLESQVVKFDLWALPPISAWIFILFIACMAGQILMNASLSIGDAGYHSKWYQTDANFRKYLILVLMRSHKALVLSAGPFNILCFELFVAIMKFSYSVFMLLNQN-

>TcasOR87

MKHVIMDELLIFLTFLGLWPRTPTSPKIISYLMIYSTSFLFFGSSIYLILHRKFGSDEIDTIEIITSQFGVLYYLTLLVVKRDGITKIVNLLSDFSKFGKPPLFDQRSRRLNLLLRLFVTVLLAATVAIVSVPVVFINSCNKQNLQLNATKICGLAAPVWLPFDYTQNPRKYFVSAMEIYCATMNYAGSGSGAFLVIGTMEHLVIRIEHLKNMFPEILNEPDKQIREKRLKKWIEYHLSIFEIGELMNETYKWPLSVIVLCVGILFGCIGVSTMQSVSFQNSSVFLFFGWFQSIFVLCFWGQRLLDSCLSIRKAVYNSKWHEMDVSFQKSVLMILIRSERPVLIHAGPFSYLSNLLVLGVLQTAYSYINLLNARS-

>TcasOR88

MTEEKQLRICLSSCFFLKWSFMWPTKSEEFRTSKGLYFRLLAFVIISGLTFTAMIVMHLLKSVEAGDYDISEDIAILATNTGYILMMLLYIIRQKDLESLLVDLSSFKKYQKPPKFDEVNRKLEWCTRMVFGYCVFGSVFYNLVKILAIPSCKKSRRINEVCGVAIPYWVWFDTENWSIKLPLILHTFLVIIIVDKVTLLVSLQVLEIACNIKLRLDQLNCMLVSCFDGDVEASRRRLNECIKYHKEIISYSEIFSKCFSIEMFTHLTTTGIICGCLENQVVQEHRPEAILHIGGWITAIFVSSFGGQILIDSSLSVAEAAYSSAWYEADVSLRKDLILVILRAQKALFVSTGPFNVLSFALFVSIMKMSYSILTILQ-

>TcasOR89

MKEAVLQQSKKEMHLLNLWPKGHVKHFRFRYVITLIIVSPFTLGTLTHFINVLKENLDVDLSGDISVIAVVTGLHFMLITFVWGHKKIAYLWENLGPHEYFGKPDNFEKRCKQLNFYSRLYAYYCYLGLTVYIIMKNRGGIECRRLNVERNLTEICGLVTTFWAPFDIDFFPFRQILFVDQVFATYFIVKGGAAISFTTLEVGEYIILKIKHLKRLVKEVFDDPREEVQRKKLVFCIKYHQYIISIQELYDGRYKHCNGCYILMVGIIIASLSNEIMKNHNIEALLHLVGWVFSFYICCFSGQSLLSESLTIPDAAFESKWYEAPVYMQKDLLLMMLRSQKPLMLHATPIGVMSLSLFITLVKTSYSYFTLLNQST-

>TcasOR90

MAKDTSPVLRESIEVMKYLQLWPQNERTNLRRRYFIVIFLCSPLHLGLATHLVVCLKDNLDVDLSANIAVLSAVTGLTYMLIVFVWSQDKLVHLLAKLDTHEIFGTPDNLTKRSRRLNFYAKLYSYYCYFGIVIYSLVQIIEMPQCRKMNEEKGLSEICGMIVPFWAPFDIDWFPLKQIFWLNQLLGIYIIIKGGAAVSITTFEVAQYICLKIKHLNRLLREAFDDPCDVVVEQKLLHCIRYQQHIIRTNELFNVCFKHCNGCYVVMVGIIIASLLNQILKEKSVGALVHFAGWICSFFICCHAGQAVISESLTIPEAALDSHWYEAPVKYKKVLLLLLVRSQKAFNLQATPIGIMSFDLFIALLKTSYSYFTLLHKST-

>TcasOR92

MKNQEIKICRATLTVLKYSLIWPSEADEMNPGKWYYIRVVTFILFTCPWVLSVFMHLIVSIRNNADIHLSEDVALMVAFTGVYYMTIIYVKKQPKVAFLLRDLSYFQFGKPPGFDETERILGFLSKLTFCYSVMAVVIYNYIKYRQKPECERMNKLKGLKENCGMLTPTWWPFEINYSPAFQLIFLYIFTSTQVMMKLSLMISFNVLEMAHHIILRINHLKTMILESLDEQDYEASKRKIKTCILYHLEILGFAERMDDCFSNGMFAHLTITAAICGCLEKQFVDGDNQLGSLLHIFGWILALFLACLGGQHLINASETISDAIWSSKWYDADLRLRKDLIFMMARSQVGLYLNVGGFGILSYALFLSVIKMSYSILAMLTS-

>TcasOR93

MTNLEIKICRATLKILKYSLIWPNEADEMNPGKWYYIRVATFLLITSLWVLSVFMHIVMSIIHDADVHLSEEVAFCVAFCGLYYMTMIYVKNQPKVALLLRDLSKFQFGKPPGFEEKERILGFLSQFFFYYCVMAVMVYNLVKLLQKPDCEKMNEIKGLKENCGLLTPTWLPFDINYFPAFHLTFLYVFISTQILMKLALIISFNALEMAYHVILRIDHLKIMITECLDQRNYEVSRRKLKTCILYHLEILSLSNRLNDCFSNIMFAHLTITAAICGCLEKQFVDGDNRLGALLHVCGWISALFVACIGGQHLLNASLSIPDAIWSSKWYEADVRIRKDLLFMMAKSQVGLHLNVGSFGVLSFSVFFSVLKMSYSILAMLTS-

>TcasOR94

MAIKICKFTRKNMQISLIWPREFEEINPGKWYYIRIVIFLITYGVFPFCTFLHAVVVIHNNLDIRISEDIGAVVSNIGISYMAIIYVQQQNQIAYLLKDLSDFKDFGKPPFFEEENKRLNFWSICTFIYPTCGASLYNLSKILEKSECNKINEENGLPATCGFIFPIWVPFNINYFPLFHIMLISTWFCTTMFVRLHLSISYNAFEIAHHIILRIKHLNGMIITCFDCQDYKISRQKFTTCVLYYKQILDLSNRLNQSFSSIMFVHFTMTSAVCGCLEKQFVDGEYVGGFIHLVGWIISLFIASVGGQDLVNASQSISEAIWSSKWYLADIRLKKDVLFMLMRSQKDLHMSVGSFGVLSYAFFVSVLKMSYSILAMLTS-

>TcasOR95

FIXMVVKESEIKVSRVTRKILQYSLIWPKEGDEINPGKWYYIRIFTFLSFTSLWCIAICMHFIIVLKDKIDWDVTEEIAIIIAIYGTYYMVLAYVKNQKKAARILRDLSNFERFGVPPGFEEEEKRLKVYIIGIFIYAFLTITFYNFFKLSQKGACERFNEEHHLDENCGLLSPVWIPFKVDRFPQFELVFLYLFTCCHLLMKLPLVVSYNALEMVHHIILRINHLKIMITECFDEPEYEISRRKLTQCILYHIEILEFATRVDDCFSNCMFAHLTLTGAICACLEKQIVAGISRFGAILHFIGWILALFIGCLGGQHFINASDTIPESIWASKWYNANLRLRKDLLLMMMRSQRDLHITAGPFGVVSYALFLSVLKMSYSILCVLTS-

>TcasOR97

MNNQKIQISNMTRKVLRYSLLWPKTNEELNPGIEYQFSVLGFFLVTGVLVLCITIRFFITIKAVHEVDAEVLAILIASYGSYYMICAHLKNQHKVALLMRDLSVFNNFGKPPNFDKRNNQLNFVAKLLALYSFLATIFYNGEQLINKTECKRINKEKGLSDHYCGLLAPCWLPFEIDYFPVFHLILIYAFTSGYLLIKMAIHISYNAFEIVSNIVLRIEHLKAMILETFENRNKQVCHKKFLQCILYHIEILDFAARLDDSFFNSMFGHLALTGGICACLEKQIVSGVNVVAGTLHFIGWILALFIGCVAGQYLINASEILPSAIWTAKWYDADLELKKKVLFMLARSQKSLFIRAGPFGILCYPLFVTVLKTSYSILCMLTS-

>TcasOR98FIX

MVKKESEIKISRVTRKLLQYSLLWPTEGEELNPGKWFYFRIFAFLSFTSLWCIAICMHFIFVMKDKPDWDPTEEIAIIIAIYGTYYIVLAYVKNQRKAAGILRDLSNFDKFGVPPGFEEEEQRLRVYIICVFIYGFITITFYNFYKMSQKKSCERFNIEHNLHENCGLLSPVWIPFRIDKFPRYELVFLYLLTCCHLLMKLPLIVSYNALEMVHHIILRINHLKIMITECFDDPDYEISRRKLTQCILYHTEILEFATRVDDCFSNCMFAHLTLTGTICACLEKQIVAGFSRFGAILHFFGWILALFIACLGGQQFINASDTIPEALWASKWYNADLRLRGDLLLMMMRSQRDLHITAGPFGVVSYALFVSVLKASYSILCVLTS-

>TcasOR100

MSPKDKIKICGITRKVLRYSLLWPVENDELSPGIRYKLTILAFFSITGILVFSISVYSVLEIKQGYDIDVEDVAILIAVYGTYYMVSAYLNNQHQIALLERDLSQFYKFGKPPGFEQLNSQLNFAVKVLIIYSFLGTFVYNGTKMLLREECKKNSQEKGLSDNHCGLIATFMFPFRVDYFPVFYIVLVITFLLAHTLIKLCMHISFNAYEIVNHIVLRIEHLKEMILSCFNERNQTIVQKKLRVCILYHIEILDMAARLDKNFFNTMFGHFALTGAICACLEKQIVLGVNIVAGTLHFIGWIIALFVGCVAGQCLLNASEIIPNALWAAKWYHADLRTQKTLLFMLARSQKELTIKAGPFGILCFPLFVSVLKTSYSILCMLTS-

>TcasOR102

MQNQSKPCQLDMMDETYLQFFVKSFTYLNMLPEKTTFCTTIQQYYVSVIITITTFPILADLVSQFYEESISFTSVNENFVALSALFAVIYVSVCFINRKHKIRALIADLALFETFSSKAVITETDKSVKFYTKLFIVYGIVGNLCYGLLPILGYKKCHESKSVHMTRYGIPCGLVVRFLFPFKFDYSPLAELVALYEILVCILGTSVVIVVTTLICGVLIHITVQLQCLRKIILDLSQVNDLEILEHKMKFCVKYHTAILDYGIRTDLAFNQMMLLHITWTGFIISVLGFEISTTDDYVEAFRFFMHLLGWLGMLFVVCYYGQKILDESLAIADAVYTFLWYKKSVIVQRYVLLILLRSQKPLTLRACGVKVMSLATFLGVLYSAYSYFTLLLKLKP-

>TcasOR103

MKQALKLADVLGFNPLKNDNLTKLKKYSSLICMISVVVSAILEFVSNFSALETYESAPESLVPQFQTLAKISSLLLSQKDITELIDEIKYFWKLDQFGDFHTRKLKKIYKYVTIFFYFYTLMLSGACVLFTITTVIFTPEKPLFLCYGGLHGLPSPQFEIYFVVDLAAIVIMSFGVAAYDGIFFYFAFHVYAEFKLVKVAFKGKSTFIEAVKHHDFLLKYLRKLNEIYSPIFLCQFFSNLLGICFCLFMLSRSGMPPELTSFSKYFISLVAFTVQTYIFCLIGDLVSELSLDISNVIFYVDWLDDEVYKSKTARLVIMNKAQSPVKLTIGKFTGMDLRTFLLIVRNAYSFLAFVNNALD-

>TcasOR105

MKPALKLANVLGLDPLRNDNYTQLKKMFCALCIVSLFVSAYLEFFSNFTTFETYETAPESLIPHFQTMFKMYSLIFSRTEIVELIQMAEQFYKFSQCDERKKLTKLYKRVDLFFYVYASLVAAACVLFAIVTLIFKPGKPIFLCYGGLHGLESPEFEIYLVVDLIGIVIISVTVPAFDGLFFYFALYIYTEFKLLKIAFKTMSGQELREAVKHHDFLLKYIKKLNSVYSPIFLYQFFCNLLAICFCLFMLSRSGIPPEMVSFSKYFLCLLAFLVQSYTFCSIGDLITELSEDVSNAIFYTDWLDDEAYENKTARLIIMSRAQNPVMLTIGKFANMNLRTFILIVRNAYSFLAFVNHALN-

>TcasOR106

MESALKLIDIIGLHPLKSDKYSTMRKTISFLSLVVILISAQLEFLSHLSVFEVYNSGPHSTIPPLQSLLKMATLHFYKNELIDLMEKSKSFWKLDKFGDLYKQELSKLHRLVTIIVYIYIALLTATCVQLAVLTLIFRRGKPIFLCYGGLYGLESPHYEIYSILDAIGIGVISIAVSGYDAMFFFFALDIYTEFKMIKSAFKRHSDQTVSSYNKQFIEAVKHHDFLLQYINQVNDIFSPMFLFQFFSGLLGICFSLFMISRSGLQDINTLSIYSAGLLGFTAQSYTFCLVGEVISELSEDISNEIFYTDWLDDEVYRNKTAILIVMNRAQESPKLTIGKFADMNLRTFIMIVRNAYSFLAFINNALD-

>TcasOR107

MENPLKLLHIIGLDPRQSDKYSTIKKVISFLIVLAVLLSALIEFFLHHNESQVYDTAPQSTVPNLQALLKMFALIIYKKELIDLFTKGNHFWKLDKFGDCHKQKLTKLHKYVDLFFYVYAVIITGAFLQLALLILIFEPGKPIFLCYGGLYGLESPQFEFYAVLDFLAIGVIAISVTAYDSIFFYFALYIYTEFKMIKIAFKRENCAQFIEAVKHHDFLLQYISKVNEVFSVIFLTQFFSGLLGICFNLFMISTQGTRDMKSFSTYFVGLVGYTAQSFTFCLIGELISELSEDISNEIFYTDWLDDEVYRNTTARLIVMNRAQESPKLTIGKFADMNLRTFIIILRNAYSFLAFINEVLD-

>TcasOR108

MGSILLLNSVLKKMEKALKLVNILGLDPRKNDTFSKFRSIFCFTILISASFSSHLEFFLNFKGLETCERAAESIIPQYQTMCKMATFLLYKTEMLDLIKKSERFWKLDRFGDLQAKNLHSTYPIFQIFFYVYVVILFLTCAMFALVNWIFDTGKPISLCYGESEGLETPWVEFYIVLQSVEVTIIFLGITGYDMVFLYYAGSVCIQFQMLKMAFAERKMNERQFLKAVKHHEFLLQYVEQLGDIYSMWFLLQYFSSLFGICFGLFLISKEGLPTEPERLSKYFPYIFSFTMQSFTFCMTGTMLSDWSSEISDEIFHSDWSDDQVYKNKTARLIVMNRAQRPAKISIGKFLDLNLRSFILLMRSVFSFLAFVNNILNRIN-

>TcasOR109

MGKVKFTEPLEFLNVVGLNPENCSNFSLFRRVISLGFFLVVITLGLLELLLHFEGLETCSRASEAMIVQYQLFIKIAVLLKHRKNLVVLMQKTRKFWPLDKFGQDAKIERPHKLLKAFFFAYKLIMILMALQYILRKFVSKNGKPLAIAFGESKGLSPKVDHLYFVLHSTSTFVVLHAVTGFDRLFFFLIGHVLTELKLVKKSYRLTQNRREKFLETVQHHAFALEFVRKLNRIYSQVLLNQHLSCLFGICFGLFLVSKDGIPPDLGHVTKYVPYVISFITQTFTFCFIGSLLITWSLQVPDAIFYNDWGKNQAYKYKTDKIIAMIRGQRAAKLTLGGFGDLDLESFNLVVKNAFSFFTFVNAMNQK-

>TcasOR110

MDKVEFSDPLFFLNVIGMHPFKADKFSKFRLAFSIAVYFAVIFSGVLELIVNSQGLETYARASDTLIPQCQLVCKIFVLAKYKKQIARLLNGSQRFWDLGQFGARYGNSFGKTHKYLKSFFLLYKVMLTFTCLQFLAVKIIFKIPKPIAISFGETKGLEPLYDHLYLVLHAMITLVTINLVNGFDGLFFYFIGHVLTELKMVKVAFGDSPIETNWSEEKRFKFAVRHHRFVLDFIEQFNIVYCTMLLVQHLTCLFGICFGVFLMTKDGVPPDLDRASKYLPYIVTFIFQTFTFCFAGNLLLSWSLEIPNEIFYHDWAKKTTYENKLAKIISMKRGQRAARLTLGGFANLDLDSFRMVLKNALSFFTFVNAMMNKKAVTSV-

>TcasOR111

MEKVRLTEPLFLLHIVGMSPHDSGTFARIRKIFSILVYTSTVVLSMAELFFNYKDLETVIRATESFFTQYGLAWKIAVFVVYKTELAQIIRLCDNLWPLDEFGTGHNFQFLHKFLRRFFLLYTGNLALLCTQFAVTAFFDDQFKSVMVYYGEKESRSQIYDNFVFTLQVIYLYVGCFVVAGFDCFFFYLLGHAVTELKMLTISFSCKEIGRNWGYEERFKCSVKHHIHVLELLDKINKVYSVMLLNQHLCSLFGICFGIFLMTKDGIPPNVDHFSKWSTYIFTFILQVWTYCFAGDQIMHWSLKIPDEIFYDNYWNKYSLKNGLNKIIAIQRGQKAAGVSLGGFAMLDIESFNVVIKNAVNFFMFMDKMYKRE-

>TcasOR112

MITRLMAQFAIKGRVGTGGYIMDKVKLAQPLAHLNIIGLDPLKNDRFSKIRTVITVAVFALCNVFSFSELFLHYNNPHVIVRSSEVVFPFFQNDWKIAIMLVYKKNLAQLIQNTSRFWQIDAFGKNYQYSMGIKHKYVRIFYLVYRLMLMFSCSQYILLTIGSDRPMILSFGETGGLGSGALLFYLIFHIVYLLIIFNVINGFDGLFFFLVAHVLSELQMVKVAFSSSKVITFWNHKRRFKSAIQHHRFVLDYINRLNSIYSILLLNQHISCLFGICFGLYLFISDGFPPDYEHISKYVPYVIYYITQVWVFCFAGQLIIDWSVNISDEIFYHDWTLNRTYENKTDKLIIIQRAQHAARLSLAGYGNLDLQSFNLVLKNGLSFFTFVNAVIHK-

>TcasOR159

MRGKTIESTTNPYSSLKKVFIDFAYSKLVISYTKASLTFHVLSLLLEVYYLVTNFSVELICRYGCMMCLMTYMYSKKLKLLEKPCLLDFWKVYNSSTATQRLISEKSSKTNRRLYCALTCCFFLAIILFPIWGDLNEFFIFSQVYEKYFTSWAPAFCYFYVSTLLWCCFYCFHLPGIIMYLTLHLDLQFKLIKDKITEIDKNCSQKEIYQILRLCISHHVALKKWMDKLADLLVTIMPFFFLFGALNSIATSFFVLYTLQNTTMILKIRLGTLTLCNFIIVSTFAEVGQIFSGQNNSLFEQLMDCSWYLWNIKNRKTLLMFMLNCMKPKTFSWGGITLNYSFVLFILKTSLSYASVLFKLRGETF-

>TcasOR160

MSGKTKRITTKTIHLSNPYSSFKKVFSDFAYSKIMIFYTIATLAFHMLSLFLQIYYVATNYSVELICRYGPMMCLAIYVVTAKVVGVFYYKTFTMLENQCLFVLWKTCNSSPTTQRLILNKSLKMNQKLHLALMSYFLLAIVMLPTWGDLNELFIFSQVYERYFKFWAPVLYYFYISTFLWCSYYSFHLPGCILYLTLLLDVQIKLINDKITEIDQNFSQNEISETLRLCISHHIALKRWMSTLAKMVNSVMPVFVLLGALSTVAVSFFVLNTLQNTTMILKIRLAILTVCNFVIVSTFAELGQIFSDQNNSLFEHLIDCPWYLWNVKNRKILLMFMANCMKPKTFSWGGITLDYSFAISILKTSFSYALILFKLRGETIRN-

>TcasOR164

MSGKTKRTTTTRKINLANPYSSLKKVFIDFAYSKIMMFYTKATLAFHVLSLLLELYYVATNFSVDLICRYGCMICLMTYVVTAKVVGIMFSKPFKLLEKQCLFVFWKTYNSGPTTQRLILDDSLKMNRKLYLALMFYLLLAIVLLPVWGDLNEIFIFNQVYETYFKFWAPVLYYFYISTFLWCCYYSFHLPGSIFYLTLHLDLQIRLINDKITEIDQNFCQNEISETLRMCISHHIALKSWMSKLAKLVDAVMPVFVLLGALSTVAVSFFVLNTLENTSLILKIRLTTLTVCNVFIVSTFAELGQIFSNQNNTVFEHLMNCPWYLWNITNRKTLLMFMLNCMKPKTFSWGGITLDYRFALTILKTSFSYALVLYQLRGETN-

>TcasOR165

MSDNTKKATTKSLDLTNPYSSLKKVFINFAYSKIMIVYTSATLIFHILSLMLEIYYLATNFSVELICRYGCMMCLITYMVTAKFFGMLFSNQFKFLEEQCLLDFWKAFNSGPTTQRLILKESSKMNRKIHLALTFYVILAIIMLPIWEDVNDFFMFSQVYENYFANWAPVLYYFYISTFVWCSYYSFHFAGVIMYLTLLLDLQFRLINDKITEIDQNSTQNEICGTLRLCISHHIALKRWMNKLANSVDTAMPVFILLGALSTIAVSFFVLNTLQSTSVILKIRLATITVCNLIVVATFAELGQIFSDQNNSLLEHLMDSPWYLWDVENRKTLLMFMANCMKPKTFSWGGITLDYSFALSIFKTSFSYALVLYQLRGNTF-

>TpreOR1

MRKIVARHNELNKFTETIENTFNQLFLLQITVCIFQFCLQGYLLISIITNVSLELPYLQVTFMILFIAYMTGDLYIYCYVAEKLNDRNSDLLVAVYECRWWALDKRDARSVQLIMERAQRPLQVTAGKFCVLSFRLFAIIGKTSLGYLSMLLAVKER

>TpreOR2

MIFNLNALGDFLHILEDDWKRYKTAKAEKELKLMEENAKTGRFIGLMFASFMYTAGLFFNTFIPIATMRAINRNYQTETLRQSNDNITHVLPIPAKVLIYTVHSSVMTSSSPKYELLFLAQYFFAFLRYTIMVGICSIMAAYVLHVCGQLDIVIMLLNQYIDNTNTDKTIHLDCTQRKKLSIIVTCHARALRLAARIEKTFNFMNLVDFIGCTFQICFTGFLLVMTLGGKPLIWVTWALLLISFVFNIFIICHIGEYLTQKCQEIGEIAYSIKWYDFSSKRAMNLMNIMIISSSYPTRLTAGKMVYLTMTTFSQVLSKDY

>TpreOR3

MPDQADYSFAIGPCRTGLRIFGSWPDPIIPLTQLDIFRSIIVSLTILIFGFIPQLSMAIIVANARDWNGVIEILTTATVPFVVSLTKFNVSCYQRNVLKTLTTMMKDDWNDYHLDADLKFMQENAGLGRKISQICLFLALSVVIPHCLLTTVIYFVNWGEYGDLCLISYFPFETNRRPNYEIILVGQCFSLIFGASTHAIIDGFFSILVLHICSQFKILQRQLSQLIENCRDAQHEKSFFEMLPKIIDRHDQLNRFVSLIDDSFNLMFLAQIMATATSLCFQGYQLVMVTSASENGISVLELTQLVFFIGSYSSSLFVYCYVVEKLNYESYQLVNTIFSSGWYDLPSSVTKNLMLLMCRAQKPLEVTAGKFCYFSLEFYCRILKSTGGYISMLLAVRDRLAEENQ

>TpreOR4

MANVKREKGFKVAMGIAESIMRFSGIWPGVEKPKINYTRFTFIPVMLMILVFVNIPQTIQLFYFDGNLSAILNVLTMADVPISIALVKFLVTSYNHQTLNKLLVLLNDDWKHVRDASDIEIMWQKAKTSRKVSKICMVLSAGTVFAYSGRMLYVLYISTLTNSEDNVDKQIVRPLYFSAKFFYDTQKTPNFEITWILQMIAAFLSALAFGSIDCLFISFILHLCGQLVILQRAFEKIGSDDVLTDANFDYTIAKLIKKHNRINESVHYIETSFNKSILFQALSSSILFCFQGYLFIIILSTAKDTEALIEVTFMLYFTTCFMFSVFIYCYVAEFLVDESLKLNYSIFYCKWYNLPVKKSRLLIMCLLRVRKPLQVSAGKYVFLSLNLFCHIVRTSAGYISVLLAVREKLFIQ

>TpreOR5

MSDDNLAGYEESISAIRFIARINGLWPFDKGARQYQCAVPACLIIFFIIIPQTTKAIYSRNDLDTVVEVLSTCELIEIVVLLKLFGLLYNKRDFQKLLTQVEDDWKISFEYEQKIMWSNARFSKLAAIFCVIATAGSVILHSFLFLLSVNSINKSEYNNSTPVIYQLFIKSHFPFETQNSPIYEIICFSQFSAAFLSTFVFSTFDGYFFLSILHFSGQLYNLKYNVYNLITQDSIQNKSFTRKLAVVVCRHRHIMSYIDLIENNFNLIFLLQIFSSTVVLCMQGYQFVLIISQGTRLFTSIVFIIFFMCSSIISIFVYCYIAEIIRTESDNLLYAVYEIDWINLKSRDANLLLIFMSRLILPVKITVGKLVPFSLEYFTTVMKTSAGYLSVLLAVAKD

>TpreOR6

MNLKIKAMSNNSAKTFIRHKQITNPAGSDGFEYIMAPCRFFLRLLGAWPDPLENDSWSSTLRILIVTTIMFLFAIVSQTVKLFNCWDNLNAVAEILSNCNIPTSIATVKIVNIWYHRHVLKDMLSQVIDDWKLPRTEEELTLMWQNAKVSRLLSIGCIFMTEATLLAQCMVGLWIPIFYTFQKSQLNSSVEWPLYMTGSFPYDTQKTPYYEFTIFGQLFSNVLASTSFSSSDSIFFILMLHLINQLSILKLSVTNLPQKIVTIQDRINFMNKFTSFHARHNQLWRFSLAVENTFNRMFLIQMVPCIFGLGTQGYQLISNIIQEHTPLVELVFMIYFLVLFLFTIFTYCYVAELLRRQSLEISDAIFACNWRILKSREIKLLAFVMARAQKPFEITVGKFANFSLQLYVRILKTSAGYLSMLLAVKEKIDT

>TpreOR7

MIKKLTEKFTKIDYDNFENYDPFLFLKFMYILCGIWPLDKSYYPNYARKIIHILPSLSMTGIFLIMMSAFFSPSVSTEDFIEIFSLQSAAITALTRMYFLLLRKPQVYSMIYHTIEMETKGPIQLLSKEECIIINKWRIIQDFLTKMLSVGYLLGVIFYLIPPAFFRKLPFPVFIPENLCTEWYTRLYIIECIAIFLSCPAWVSLDLYLCIFLCQLCMHLELVYAAVQDLRGKDRATLHTIIRRHSKVLMYGQKLCEVVHYIFFAICCSYGSFLIFGTITIGEISWKTHGALAIKNIVTLVVCSSTIYLICYVGELLQDLSSRIGDGILLDNYAEHGSNRKYLKELEIMNDRCKLSLKIEYTPNMIVNMKLFTNIMNYVVSTYIFVNTMFVNPV

>TpreOR8

MYFLLLRKPQVYSMIYHTIEMETKGPIQLLSKEECIIINKWRIIQDFLTKMLSVGYLLGVIFYLIPPAFFRKLPFPVFIPENLCTEWYTRLYIIECIAIFLSCPAWVSLDLYLCIFLCQLCMHLELVYAAVQDLRGKDRATLHTIIRRHSKVLMYGQKLCEVVHYIFFAICCSYGSFLIFGTITIGEISWKTHGALAIKNIVTLVVCSSTIYLICYVGELLQDLSSRIGDGILLDNYAEHGSNRKYLKELEIMNDRCKLSLKIEYTPNMIVNMKLFTNIMNYVVSTYIFVNTMFVNPV

>TpreOR9

MLNDQNSVDSFYDSTTYHLNKKCLILAGGWPFLNPRKRKTIWLAINFSLFIGFVAEIIYIGEIIDRTTEVINCLMVIFCAILAFCLSINGAVKSHSMKRLLDSVRENWNDLQSDDERNIFSKYANYGKIFTIGLSVCYYLVLLCQCVSPLIPPMLHYCITGNWTDPERNILDVEFFIDPVKYYWPIFIHGATSTVAAVWMLVAYDLFFVVVVLHCCGMFAVLRYKIKQMDIMLYRYSYDQQLVLKKIEEIVMYHLKCLEFAQKIEDFFCIQCIIQILVNTIVISVCGSQVIQLADTSPQESFKFAFVGVSGTFRLAFFNVCGQIVNDQSLRVHDQLIYTTWYEYPKRARKLFVILYNRSAEPCNLTGAKMVNLNLATYSMLMKTAMSYFMMIIETQ

>TpreOR10

MLKVHQNIDDSIFDSSAYHINKKYLIIVGAWPYLTPGKRKLMWLAINLALFTVWIPQLIHIIVIIDRKKEVIYCLMTYLCASMGFNSSVNGLLNNGALKKLFDSLKENWNDLQSDDERTIFATYANYGKMFTVGLSISYYLVILSYIVSALMPSMLHYYFTGNWTIPERNIFEAELFVDPVEYYWPLFIHGIIIAFVAIWILLAYDCFFIMIVLYCCGMFSVLLHKIEQMDTELYNYSYDQQLALEKIEEIVTYHLKCLEFAQKVEDFFCIQYVIQLLINTIVISVCGSQVINIADESPLDIFRYTYVAGSVIFRLSLTNVCGQSVHDRSLRVYEQLIHRNWYEYPIQIRKLFIVLYNRSVEPCNLTAGKMVNLNLETFSKLMKTALSYCMMIIQTQ

>TpreOR11

MLKVHQNIDDSIFDSSAYHINKKYLIIVGAWPYLTPGKRKLMWLAINLALFTVWIPQLIHIIVIIDRKKEVIYCLMTYLCASMGFNSSVNGLLNNGASDDERTIFATYANYGKMFTVGLSISYYLVILSYIVSALMPSMLHYYFTGNWTIPERNIFEAELFVDPVEYYWPLFIHGIIIAFVAIWILLAYDCFFIMIVLYCCGMFSVLLHKIEQMDTELYNYSYDQQLALEKIEEIVTYHLKCLEFAQKVEDFFCIQYVIQLLINTIVISVCGSQVINIADESPLDIFRYTYVAGSVIFRLSLTNVCGQSVHDRSLRVYEQLIHRNWYEYPIQIRKLFIVLYNRSVEPCNLTAGKMVNLNLETFSKLMKTALSYCMMIIQTQ

>TpreOR12

MNVFKSEYYKINYYYLKLLGLWPNENHSRKKFKRITVIFLIVSLLIPQYIRLFEEWGRDVDIVIELIGSIFYFTGSQMKYMSFVRVESKMKFIFEEISRHWNTLTDAKEQKLLRENGRYGRIIALGYIIPINIILVVYITVPLAPAVLDIIDPLNESRPKAFPYFAEYFIDDQKYYFELTIHGWIVCILSVQIYATFDTTYQLCMQHVCALFSIVENRIREANKLSWRGNSDSDHKIQMRDRSYERMIQAVVLHKEAMRFIGLIEECYTFVYSYVVLANTLLLSLTAVDTMLNFEKGNFKQMIRLGMLYIGFSFHLLYNMNPGQNVIDSSVHIQEAAFHTNWYDSSSKTKQLLRIIMMRSWRPCKLTANGVVTLNLETFAFVFKKSISYVAVIGSVR

>TpreOR13

MHSLVRDIIIQNVEYEILPYQFLLLTFWGIWYPQNWSLWAANIQKSYFVFISFLDIIICTEMLIFFINSFGTSNFKLINFFFVSANITGVYKAIKLMLNRKMIREFLLTYFDADWRTPKDKIEQKIHDDINAKIKRVTLIYSVSMLGIVMMKAMSPLTGSNSVSLPVEAWYPYKVEKTSWYWLTYLHQCILGSSAVCAHIGIDTLFMGLLLKTSYQLEVLKHRLRSLNISLLSLNGKGSTLSIENEKMLIIECIKYHQRIYSFGKKLNDKFQDILIILVVSSLPNICINIYALSTYTSRTKIDIIATLFCTTSAFMQFFIACWFGNEITWNSINVRDALYDLDWTVFNLDSQKLFIFIMTRSMRPMQFKIGYLLSLNLDSFIKIIKASYSSFNILQQTTH

>TpreOR14

MNVNFAPIPFRVLKCCGLWRPLSWTSWKKMTYSGFSLVSLMIVIITALLVLIAVCQMSFNDDLFAENVFLMFALINATSKAANVLLFRGRFIALLNMVASERWSKLRSDEELEIRAKCDKTIRKISVYFTTAVFVAILLRVIAPLVDLFVKGEIRLPVNAYCPCDIRNPSCYWMLYWQQAIGTGIATLIHAAKDCLICAFLLQTCSYLEILKRRIVAIPGIIASERNCGNSGNIQEMEKRLISGCVEDHDNIFLFSKILNSSLEVMLFGQIAVTLPNLCLSIYLLSTQNIASMDFVMTIQFFSAVVIELFFFCWYGNQVTLNSFDVEAAISTMDWTSLTVQSQKFLLQMMVRTARPILFRVGPIMDMNIDSFLSIMKTSYSAFSVLQSTKE

>TpreOR15

MMKMKQQGLVADLLPNIRVMQFSGHFMFNYYNEGLKFPHRVFCIVSFLLILVQYSMMGINLAMEVGDPDDMAANTITMLFFIHPIVKTIYFAARSKTFYKTLGIWNNPNTHPLFAESDAHYHSVAVQKMRKLATFVGAATIFTLFAWTTITFFEDSVKTVVDKETNETTIIPIPRLPIRAWYPFNSMKGFGHIMAFVYQFYYLAMCLTLSISVDVLFCSWLIFACEQIMHLKAIMKPLMELSATLDTVVPNSGDLFKAGSDDHLRDTNGVQPAGDGIVDSDVRGIYSNRQDFTATFRPTAGTNFNGNVGPNGLTKKQEMLVRSAIKYWVERHKHVVKLVTAIGDTYGVALLLHMLATTITLTLLAYQATKINGVNPYGATVIGYLLYTLGQVFHFCIFGNRLIEESSSVMEAAYSCHWYDGSEEAKTFVQIVCQQCQKAMSISGAKFFTVSLDLFASVLGAVVTYFMVLVQLK

>TpreOR16

MAEEEMKINEVDDFFDLHYFALNKKFQIITGLWPLETGYLKYLKQGAMASVIIWNLILFSHALGTFCGTNMDYCCENMIAFVYSTSAIFKLIGISTTGAKFTVIYRMIARNWRNTTDKVERSILEKYALISKRLSLIYIIAFSCIATIVTQLPFIPLLLNVIMPLNESREAIIVINTDYSITPYTKSGHLWVHYSLTGTTTAAVFIATDATFLLVVFQILAIFEVVKQRIRQAVLVAQESSEQKSYGILIKAVQLHKDAIQFLQLTDEANSLQFFAGLGGTIFMISFGSIALLIRMDAYADLFRVVILTMGYLFQLFILCLLGELVINASTELFNFPMLTDWHVLPIKSKKLILFFMGRTIRPSYYTAGGLYIMNVQNFASIIKTAVSYIAVVLQFR

>TpreOR17

MTFRLCVEALIGTLVNGASTQGAQFDSQYRLEEKEKRKDRVVGAAPMEIEAEYSKLTWPMRAVMSSISYWPGREQEEKDLGKRSSWSRLLHKCHRGVVNASMLVLTLGGSSEIVHLGRSADINDLIECCLIVSTAYLALLRVLVFATHATSMSRIVETMRNDWTDNYRDEADKALLRDRCLWYYKLASFYICSVIFAFASFTISPYMEIMLRKDDGPMFLPFRGYYFFNLSSVSRTEFNGIYLLNSMAGFFACGTIAGASSFSLIAAVHGSAKFAIVQKHFESVEWTSRQQVKRCVRHHQDCIKFADDVEDSINILVLGQFVMSTCLLCLAGFQFTTMLRDRGRCMKYLSFLQAATTNLFLYSIAAQALQTESLEVAEAIFRSKWIGSCSSYEIRMIIMRSRKACKITAGKFYELSLESFLKVLSSSFSYFTVLFTAKYDGV

>TpreOR18

MTTTAATKERGPSIRYKNAFSEAKKLMSWNKYLMLPLGLWPSKPNDYIFVTFFCFFYYHLFLYHVVLLVSIRSFSLMRIIGALMENVTMVQVFLRLYTMRRYNKEYGKILEEFGQDFSEDNYESNEEKRIFLSYNIQSKRFIRIVVISLGLTAMLYFTKPLIRQLSTXKKNNKRKAFTYDLPYRIYFWYKIADLNIFILTYVSQIPLLYTIGFTQTAMDCLTLTLVVHLCGQLGVLSERISKIDFINGTSKLIRAIKRHQELISTGLMLRKIYRVCLLGHFLGAAISICTLVYQLLMSISTGQKTNLVTFFVYGFLNIFRLYTHCWAGEYLIHESINVSNAFYQCEWYKLPVEDQKKIIFCIRRSQKALSLMAGNFGHFSLVMFTSIMKSAMAYLSFLRNFI

>TpreOR19

MNPDEVFDNKYFRLNRTLLKQVSLWPYESTSTKVIKRIFIMVGFYSMSLPQMIRGIEEIRSDDPDPEIIIENLSGFIYFHGVISKLITQMVTENKLKYLYEEISKDWKTITDKNEKAVLEKSAAVGHQLTIFYTGFVVLSAIFFVSITAFVPVLLNHVLPGNQTYQKQICIYAEYFVDQEKYFYYIFTHTMVIGVMTVYVATAIDSVFVNCVQHVLGLFNIIKYRLKEISRVYDSSVNNSIDLHFDVKRYLIDIILTHKKSLEFTNLIQSAYNECFFLLAGLIVAGLSAFTYVLSQNVNNPLNFMRIWFLWFGVIVYMFFVNLPGQKLLNISEELLLAIYDSSWHKFPIKTRFLIQVMMLRCLKPCRLTAGPLIEMNFASCSNILRTAFSYFTVVNSMNS

>TpreOR20

MDAVFDNPNYMISKFLLRFLGLWPTQSRLRKNLSFLAYTFIIFSLVIPIFFGMLNNKTDLVIIIEDITGILYLLSIYTKYLSFYVFEERMIRVYNQLVKDLEDITIKEEKAILRKHARQGRFLSIIYVGYGAIATMVFNSTPYIPLILDKVFPLNETRDLLFPYYADYYFIDAVEYHYTLFTFHGGMVICAATLGATAVDSMFVVNVKHNCGLFNIVCYRLENIGKSALSKDHALPTAHASDEVVYREMKSVFISHRNSIESSQIIQETLSGSFLFIFAAAMAAIAMLVFDIMLNLQKPIQIIRIGVLLVGVYLSIFYMNYIAQQIMNDSEKVNEAACNSYWYHCSPSAQKLVQLALLRSQAPIILSAGGVFDMNLATFASMIKSSASYATVLLQMQQK

**6. SNMPs**

>AmelSNMP1

MRFKKLIHDITTRFFHRGVHVILHSSFVKLQSFVTTGRKLHETLRVPVGRKCVTMKPKKLGIIGGSLLAFGILICAIAFPPFLRSQVKKQIALKDGSEMRELWSNFPVPLDFKIYLFNVTNPMEITAGEKPILEEVGPFFYDEYKQKVDLVDREEDDSLEYNLKATWFFNPSRSEGLTGEEELIVPHVLILSMIKLTLEQQPAAMGILNKAVDNIFKKPESVFVRAKAREILFDGLPVDCTGKDFASSAICSVLKEKDDALIADGPGRYLFSLFGPKNGTVLPERIRVLRGIKNYKDVGKVTEVNGKTKLDIWGEGDCNEFNGTDSTIFAPLLTEQDDIVSFAPDICRSMGARFDSYTKVKGINTYHYKADLGDMSSHPEEKCFCPSPDSCLTKNLMDLTKCVGAPLIASLPHLLGAEEKYLKMVDGLHPNEEEHGIAMDFEPMTATPLSAHKRLQFNLYLHKVAKFKLMKNFPECLFPIFWVEEGILLGDEFVKKLKTVFKTISIVGFMKWFTIVSGTCVSGAAAALFFKNKDKNKLDITKVTPQKGEEKKWPNQMTISTIQSAAVPPNLDAD

>AmelSNMP2

MWSYQVCAIICVIFGIYACITNLFSDGLFSIKNAILKNLPLIKGKDMYDEWILPVNLIFKCYFFNVTNPDEVMEGNNPNLVEYGPFTYREVFEKQIVDVDEELDEIIYDVKSTFTFDKYASLNISKRDTVTILNPAYIGTISMASIIGLTTLPPSYIEKFGNNIPKLFPNRSSIFLKANPKEILFDGVKLTCNERKFPELSTICKTLKALRSPVLKEGEKEGVYYLSIFQRVNGTIRGRFSVNRGVNNISELGNIGSYNGRRVQTIWRTEKCNTVRGSDTITWAPLINPMPSVLSFIPDLCRSIEADYDKEVSIYGLIGSRFVMRERTWFLNQSQCYCLERNKVPNCLPQGLIDVSDCLVMLRYVMLQKVPIIMSEPHFLHGDPQLLMYALGLNPSEDLHETFIVIEPYTGTPLSGQKKIQLNLKLERQPVDLLSNISEGYFPLLWCANVRIFSKIIKLQY

>BmorSNMP1

MCGIVVSIVILVVGASLVLVSTIVGFAVVPGIVENMIIDSVVLSDDSQQFERFQEVPFGLNFSVTLFNIENPAEVLEGGVPNLTERGPYIYRLRQNRIISNEEEDKLTYNRLENFEFNERASFPFTEDDIVTITNTPYHAILQVAESAFPEMASFLPMVMNGIFGQNNNAPIIDIRVGDLLFDGIPLCKDADLIGRVACNLIRNMTETSQNIETQDDGSMLFTVLAYKQNKPTAPYKVLRGLNDHTDLGRILSYNERSSLEYWVDEVDEEGESIEPSICNTIKGTDSAIFPPFVDTGNSIFPLNADICRSVELRYQYDTEYEGIPTKRFSANEWFLDNEDGCYCLNVTKGITQENGCLLRGTMELFTCSGAFMVLSYPHFLYADPIYRNGVVGMNPVEDKHRIMLDIEPNTGTPVVGAKRAQFNIFIRPISGIVATDNIKTTLMPLFWVEETSRLPLNFVEEIQGRLLRPLNLLSILIPVIVAICFVILVIGILVTIRASSKRNKIKQ

>BmorSNMP2

MLAKYTKTIFSVSVAFLVVSIVLATWGFPKIIRKQIQKNVQISNTSKMYDKWVKLPMPLDFKIYVFNVTNRDAINQGEKPNLKEIGPYVYKQYREKIILGYGDNDTIKYNLKKTFVFDPVASGDLREDDELTVINFSYMAAIISVQEMMPAAVGMINRALEQFFTNLTDPFQTVKVKDLFFDGLFLNCEGDNTALGLICGKIRAEKPPTMRISKSANGFYFSMFSHMNRTVSGPYEMVRGTENLSDLGHVISYQGKRIMSAWDDQYCGQLNGTDSTIFPPLEDGNIPEKLYTFEPDICRSLFASLVGKDTLFNISTYYYEISDMTLGSKSANPDNKCFCKRNGSVKHDGCLLMGVLNLAPCQGAPAIASLPHFYLGSDELADFFGDGIKPDKEKHNTYVHLDPITGVVIKGVKRLQFNIELRNVPSVPQLKEVPSGLFPLLWIEEGAEIPEWLRKEIMDSHTMLWYVDAARWLVLAVAVVAVLVSATLVARSAALIPWPRNSNSISFILGNSVNTSKVHS-

>CfloSNMP1

MSRVVKYGAAGAGLFVFGVMFGWMIFPKILKSQVHKQVNLKEGSQVRPLWSKFPFALEFRVYLFNVTNADDIKNGAKPILHEVGPYYFEKWQEKVDLQDHEEDDTVDFSIKNKWIFRPDMSEGLTGNEELIYPHIFILAMAMGAVREKPAMLPIVNKAINSIFKSPDNVFVKVRAMDVMFDGLPIDCTVQDIAGSAVCAMVKENADELMKDGENKYKFSLFGAKNDTASKKRLRVLRGVKHQMDVGVVVEYNGKKNISTWDDEYCDTFNGTDGTVFHPFFGEKEDIVTFAPDICRSLSFTHERKSSYKGFHTSRYTGFLGDPNTMPWQKCYCPSPDKCLGKGVMDIYKCTGVPLVLSHAHFYRGDESYLTMVDGLNPSKEKHDLFLEFEPFTGTPLLAMSRIQMNIMIHKVEKIKIMKNFPEAMLPLLWIEESVGLPDWLLKQVKSGLRMVAIAGYLKWLMIFGGLIMCGFAVFFYFHGAQKSSNRIQLPKVTSNNSSNSGSVDKKISPLSVRTLHAAQVPAALD

>CfloSNMP2

MKSSGAMKLGVGGVGLFVFGVTFGLVIFPEILKFMVHQQVNLKPGSQLRPLWSKFPFALDFRIYLFNVTNAEEVKNGAKPKLHEVGPYYFEEWQEKVDLQDREDDDTVDYTVKNKWFFKPEMSEGLTGEEELIVPHIFMLAMVMTIVREKASMIPLVSKAVDSIFKNPDNVFVRVKAMDVIVNGLPIDCTVKDLAGSAVCSEIKKNSDELMKDDEDMFRFSLIGAKNDTPTKKRLRVLRGVNNQMDVGRVVEYDGKPNISTWGDKYCDTFNGTDGTVFHPFFKEKEDIVSFAPDICRSLSIGYERPSSYKEFNTARYTAFLGDPNTIPWQKCYCPPPDTCLGKGLIDTYRCTGVPIVLSHPHFYLGDETYLRMVHGLNPSKDKHEIWLEFEPLTGVPLIAMKRIQVNVMVQKVEKFKIMKNFPEALLPLFWIEEGVALPDWFLDQIRGEHTMVAVACYLQWILMIGGIGLCGIAGLLQFEANQKSKKFEVTKAS

>CfloSNMP3

MACLIWQSLITTFGLILAATGAILGFYVFPNIVTKKIADSFQLTNGSDAFERWMNTPTPVYMKIYFFNISNPQDVKNGGAPIVKEIGPYVYDLIKSKVDIAEENDNLTYYQRQLFHFNQDSSGCHKEDDVIVVANLPLMKNKTEEKSYKINSGVKNIKLLGEIIEWNNSTKISAWNDNGTCNNVYGSDSMLFPPYVTENTNITVFQSDICRTVYLDFENRSKYKGLSSIKFVESNMLTSGATFSNNKCFCVNETKGILGEDGCLLDGAMELQTCLNTPVVLTFPHFFKAHTKYQNGVLGVNPNKTLHETFVELEPKTGVVLRGAKRVQFNIFYRKIKHAKLTDKLNETLMPVLWIDEGIELEDKYVDRLNEQLFSILMMLDKIEWALIGVGLLVSIIGFMTFFIVVKINLRKSGG

>DmelSNMP1

MQVPRVKLLMGSGAMFVFAIIYGWVIFPKILKFMISKQVTLKPGSDVRELWSNTPFPLHFYIYVFNVTNPDEVSEGAKPRLQEVGPFVFDEWKDKYDLEDDVVEDTVSFTMRNTFIFNPKESLPLTGEEEIILPHPIMLPGGISVQREKAAMMELVSKGLSIVFPDAKAFLKAKFMDLFFRGINVDCSSEEFSAKALCTVFYTGEIKQAKQVNQTHFLFSFMGQANHSDSGRFTVCRGVKNNKKLGKVVKFADEPEQDIWPDGECNTFVGTDSTVFAPGLKKEDGLWAFTPDLCRSLGAYYQHKSSYHGMPSMRYTLDLGDIRADEKLHCFCEDPEDLDTCPPKGTMNLAACVGGPLMASMPHFYLGDPKLVADVDGLNPNEKDHAVYIDFELMSGTPFQAAKRLQFNLDMEPVEGIEPMKNLPKLILPMFWVEEGVQLNKTYTNLVKYTLFLGLKINSVLRWSLITFSLVGLMFSAYLFYHKSDSLDINSILKDNNKVDDVASTKEPLPSANPKQSSTVHPVQLPNTLIPGTNPATNPATHHKMEHRERY-

>DmelSNMP2

MIHWSLIVSALGVCVAVLGGYCGWILFPNMVHKKVEQSVVIQDGSEQFKRFVNLPQPLNFKVYIFNVTNSDRIQQGAIPIVEEIGPYVYKQFRQKKVKHFSRDGSKISYVQNVHFDFDAVASAPYTQDDRIVALNMHMNAFLQVFEREITDIFQGFANRLNSRLNQTPGVRVLKRLMERIRGKRKSVLQISENDPGLALLLVHLNANLKAVFNDPRSMFVSTSVREYLFDGVRFCINPQGIAKAICNQIKESGSKTIREKSDGSLAFSFFGHKNGSGHEVYEVHTGKGDPMRVLEIQKLDDSHNLQVWLNASSEGETSVCNQINGTDASAYPPFRQRGDSMYIFSADICRSVQLFYQTDIQYQGIPGYRYSIGENFINDIGPEHDNECFCVDKLANVIKRKNGCLYAGALDLTTCLDAPVILTLPHMLGASNEYRKMIRGLKPDAKKHQTFVDVQSLTGTPLQGGKRVQFNMFLKSINRIGITENLPTVLMPAIWVEEGIQLNGEMVAFFKKKLINTLKTLNIVHWATLCGGIGVAVACLIYYIYQRGRVVEPPVK-

>MmedSNMP1

MTSCSTRFRNFMSYVPGLLLISLGIYLATEKPHTNYLINHIREAAELVEGKYGYEIWKDLDIYFKVRLYHITNPDSVMEGENPIIEERGPYVYNLNMKKRVTNVDEKLDEMAFTIFRTYQFNKNASGSCSEDDQVVLLNSAYLGTLNIIASKFPAFIGRFGNGIQYLFPKSYDIFLRGKVKDILFDGLPLVCDPVKYKDLSLLCNFLKGKKPPIIKDTDKPGVYSYSLFDKNNYTDSDPFTVNRGVENKDALGNTTSYKNLRVTKYWREKECNLVSGTDSITWAPMTEKLPFVSVYEPNICRRMTPNFKREIIVNGLLGYRYELDETTWRKENMGCYCLPNAKKVPECLETGLLDITKCQDAPVIFSEPHFLHADPNLLEYARGLKPDPIEHTTFITIEPLSGAPLSGSKKIQLNLRLNRIPGITLLANVSEGLFPILWAEEVFYLFIYFFFFLSINS

>MmedSNMP2

MLLFKKLGIAGGSVFTLGIIIGYAFFPPFLKSQIKKGIQLVDGSDMKEMWVKVPFPLDFRIYLFNITNANEIKAGAKPIVQQVGPFFYEEWKEKVDLVDREEDDTVEYKNKATWVFNQAKSAPGLTEDVVLVFPHVMILSMILATVREKPAMVGLAAKAVDSIFHKPDSVFVTATAREILWTGLPVDCSVKDFAGSAVCGILREDDSGFLKDGENYKFALFGAKNGTVIPDTIRVHRGKRNYLEVGIVTEFKGEPKLNVWPEEGDCNTFNGTDSTIFHPFLYEDEDVVSFAPDLCRSLSAIYQKPTKVKGIKTNRYIASLGDMSTDPTLKCLCPTPDTCLGKGLYDIFPCVKAPLVCSLPHFYDTDPQYLTQVDGLHPNEEDHQIFIDFEPMLGAPLSARKRLQFNIFIMPVDKFKLMKTFPNALLPLFWVEEGLILDDEYLKPIKLVFTMLKVVGIMKWLMMTAGVGLGGGAGFLFWKSTQSPQKLDITKVSPKTIQNAAGDEKKWPTSVSTIQGNNAPPSVEA

>NvitSNMP1

MKRMMKIGIAGVSMFVFGTMFGWVLFPMVLKSQVHKQIALKEGSDMRAMWSKFPFALEFRIYLFNITNADEIKSGAKPIVKQVGPYYFEEWQEKTNLVDREEDDTVEYSIKNKWIFRADLSGEGLTGEEMLVLPHVFILAMVMTTVREKPTMVPVVNKAVNSIFKNPDSVFVKVRAMDMMFDGLPIDCTVTDFAGGAVCGMLRDAADDLMKDGPDKYRFSFLGAKNDTPTTKRLRVLRGVKNLMDVGVVVEYNGKTNISTWDDDYCDTFNGTDGTIFHPFLYENEDVVSFAPDLCRSLSTTYEEKTNIAGLTTNRYTAFLGDPNTIPSQRCYCPTPDTCLKKGVMDLFKCIGAPLVASHPHFYLADEDYLNMVDGLRPSKDDHGIFLDFEPFTGSPLSARKRLQFNIMIQKVEKFKIMKNFPEALLPLFWVEEGIVLPDYLIAQVKAGHKMVAIVGWMKWLMVVGGLGMSGAAGFMYYQATQKSKKLEITKAVTNGTKPGSTEKKISPINVNTLQAAQVPPNLD

>NvitSNMP2

MALTKIQKIGVGGICMFIFSFLFSGVILPPIVKHEVKKKVALKQGWMMREVWGKFPFSFEFHFYMFNVTNHMDIKGGAKPIVAEVGPFVYEEWKEKVNQVDHDEDDTISYNAKSTFIFNAEKSKGLTGEEEVIMPHFFILGTVNSVLRDKASAMPIVSKALDSIFRKPDSIFVKAKVREILFDGIVIDCNVKDFAGSAVCNEIAQNYEEFRLQSIGDNKYSLSLFGLINGTENKARHRVKRGLKNIMEVGKVVEYDGKNNVSVWDNEICDAFNGSDGSVFHPYFDKKGKDDLVAFNADLCRSVICHYDSDTKFAGLKLLRYTTDLGTDVEKYPHHKCYCVTPDRCPKKGAMDIFKCVNAPIMITNPHFYLADPWYVSAIEGVKPDREKHMIMIDIDPFTGSPIHVHTRAQFNMFLQPVEKFKLMKTFPNALLPLIWFDEILILPDFLLKEIKGGHRQVAMAKVFKFLMMFGGLGMAGYAGFMHYKATQGENTTEVKKVPVKSSPNGVGSGEKKINISTIQPAPLPPNVD

>NvitSNMP3

MSLRICRSVCVAFGFILAGVGLLLGFYVFPLLVNQQVDDTMKLVNGTEAYERWETLPIPLQFKVYFFNVSNPDEVQNGAKPIVKEVGPYVYDEYRHKYDITEDEDGTYSYNQTQLFSFNENASKPNKEDDNIIVAHLPLMAISLIAEKRLMSELLGTVVPHLFDNPKNVFLTTTVKKFLFDGVNINCSNGGGTVRLICNQIRRNAPAQLKVPDKGVDGPFVFSLLSYKNNTHDGRYKVSSGVKDISTLGEIYAWKNSSTVDAWKPNGTCNNIYGTDTTIFPPHRTQLSRVNVFQSDICRTVNLHYNDETEYKNIKGLRFVVEKDMLMSGANYSANKCYCLKETKGINGEDGCLLDGALELYRCQNVPLVLTFPHFYLAHEKYRESVEGLNPDKSKHEIFVELEPKSGTVLRGSKRVQFNIFYRPIHGINLTNKLAHSLMPVFWIDEGVELDDPNINLLYDSLVYPMKILDGVYWTLIGLGLAIGLISMVWCMLFAHKPKHLF

>SinvSNMP1

MVTKRRACDVVVSKLRKIYNFLRRTKLLDLLPGGKALDHVLCALEKYTKSVKMVSIKKLGIGGGCSFVFGILLWCGFPGLIRSQIKKAIALKPGSEIREMWSAFPLPLDFKIYLFNITNPTEIAEGKKPKLQEVGPFFYDEYKLKFDLVDREDEDSVEYSMKSWWIFNPSKSNGLTGEEIMVYPHLMIMGMVGATLIEKPAAIGVVGKAVDSIFHKPDSIFVTAKAKDILFDGLPVDCTVTDFAGSAVCNILKTEATDLIPDGENRYKFSLFGGKNNTPVPQRMKVLRGIKNYKDVGRVLTFDGKPALDIWPEDHCNEFNGTDSTIFPPLFGPDDDIVSFGYEICRSLSAHYEAHTKIKGVNTLRYTADLGDMSTNPREKCFCPTPDTCLGKNLYDMTKCLKVPIIGSLPHFYGGDGTYLKLVDGLNPTQSEHEIDMDFEPMTATPLRAHKRLQFNMFIKPVPKFKLMKNFPEALLPLFWVEEGILLPDEFVNKVKVVFKVMAVVGFLKWLMILGGIGMGGAAGYLHYKNRDSGKLDITKVTPKTAQGEDNKEKSWPPGMNISTIQATVPPSLDRN

>SinvSNMP2

MVSIKKLGIGGGCSFVFGILLWCGFPGLIRSQIKKAIALKPGSEIREMWSAFPLPLDFKIYLFNITNPTEIAEGKKPKLQEVGPFFYDEYKLKFDLVDREDEDSVEYSMKSWWIFNPSKSNGLTGEEIMVYPHLMIMGMVGATLIEKPAAIGVVGKAVDSIFHKPDSIFVTAKAKDILFDGLPVDCTVTDFAGSAVCNILKTEATDLIPDGENRYKFSLFGGKNNTPVPQRMKVLRGIKNYKDVGRVLTFDGKPALDIWPEDHCNEFNGTDSTIFPPLFGPDDDIVSFGYEICRSLSAHYEAHTKIKGVNTLRYTADLGDMSTNPREKCFCPTPDTCLGKNLYDMTKCLKVPIIGSLPHFYGGDGTYLKLVDGLNPTQSEHEIDMDFEPMTATPLRAHKRLQFNMFIKPVPKFKLMKNFPEALLPLFWVEEGILLPDEFVNKVKVVFKVMAVVGFLKWLMILGGIGMGGAAGYLHYKNRDSGKLDITKVTPKTAQGEDNKEKSWPPGMNISTIQATVPPSLDRN

>TcasSNMP1

MTSTARRRNIMKKVYKIMDRVYNITNSVNGVVKVGYCYDAKKIDVNGDSTYTYTYNDKSGRTADDYVTVHIVGIVNTVSRDSIVDRAIKSIKDNIYITTKVRDDGMTINCKVDSATAVCTKAIGIIKNVYKSIGRNGTNRYKVRGMKKWHGRVVNHKSTVWSTKKCNRRGTDGWIIDKVGWTYSSDCRNMHVVTSHGVAKYYADGDMSSNDKCYCKTCKGMMDTRCMGVIYATHRVDKVRRTVRGKITDHIVRVIIGTAKRMNIVKKISMKTAHIWIAIVGKMIKVVVAKVDVVKYCAVCAVAGSYCYKRKKKAVTVSKTAKA-

>TcasSNMP1a

MVKWQRQLKPGNEVRDFYIKLPIPLDFRVYFFNISNPEEVKQGEKPILKQIGPYCYDAYKEKINVEDDKDNDTLTYNPYDTYFFNQMRTGDLSQDDYVTILHPLTVGIVNAVATQKPQYLSAVNKALPVIFKENSSIYLTAKVREILFDGVLINCNVKDFSANAVCSQFKGQPAMVEVEKNIYSFSLLGSRNGSIPTRITIHRGVKNAADIGRVVTIDNKTDLDVWPEPECNAFRGTDGWVFPSFLEKEDGIWTVASDLCRSFKAQYVEDLKFHGVVVRKYFADLGDMSSNPAEKCFCPAPEKCLPKGVMDLTKCMKVPLYCTLPHFLRADEKLLQQVEGLSPELERHIIKIYFEPLTGTPMLGQRRIQFNLQLMPIPKVAMMKTVPEALHPILWIEEGVELEGFLLKKVTSVFTLLKLMTFVRYIMLGLSIQGILYGGYKLYQESKSKKVSPVQNGTTESKNHNQGKTGGIELPSMNKRNKENTKNA-

>TcasSNMP1b

MSYKKITIISACCVVTIIGVAYIYAIRDISHRRNVRYKYIDRVNNVSNDVNGGVVSVGYCYDYKRIDVDNADSTYTYDIYNRSGNSDDYVTIIHVVSVNYVSVKTHYNDAGKSITAKVRDIDGMINCTSRDTAMAVCTIRTKIGISKDYKYAGNGTTRITVRGIKSNGKVAVDNVTKSDWSNCNYKGTDGWISGRKTIWMHATTCNIHADVGATSNGAVNKYYSDNICTNCSCGIDVTKCTAIYISHRSDSIRGVKGNDTSHITRIGTSMAIRNVVKKITIMNVSVIHVWVMGVVNGWRMIKTYTAVMKYISVASGTAYGGYHYKNKKYSKNIVSSK-

>TcasSNMP2

MYTLCPPTVGIFGCLALVLGVISSLYVIPTIENSYLLHAVYQNGSFLLNEFLKPEVKTVFKIYFFDVTNSEEVKKGEKPIVREIGPYVYNEFKFRTIINYTETSDTFDFFEKTQLFFNAEESGGRSENDFVTVINSALITIGNNIEDQIKHQTSKVDDVFEHFLDDYDLFIKARVRDVLFDGIVINCSNESGLVCLYLKTEQTEFLRPFGNDLKFSIFNHINVSENGPFKIQIRNNDNKRGHILAYQGRKTLNNWSLKHKCNEIKGSNLIIFPPSMSSTQVIYTFFSEFCRSIAFPFDSYSKIRDVPASVYVMSRETFDSSRKNSCFCKASGIKRGSLSCNVKGTMNLKNCKNMAIILSHPHFYLGDDVLLNYVQGLSPEKKIHESFITLGARSGIILNYAVRFQFNVPIKRNKHLGTTNMREGIFPVLWTEEIQELDEKVINFFQNMYHFINMLHLLKNVLVTFGILLMMCSIVLMVYRDWNTVFESGATKKIITGRSACNEKTNANLFPQTTNRNISDGFIKRNQPKKIFLKNVISK

>TpreSNMP1

MLAKFQQELRETLKASAAEKRRRGRTTATKGGILVVAGIVFGWVLWPMMLNFAIHRQVALRPGSKVRKIWQKFPFPLDFKIYLWNVTNPDEVTKGEKPVLNQIGPYFFEEWQEKVDIVDRKEDDTVEYSLKNKWIFVPGKSDGLDGQEELVMPHIFMLALIMAGVRERPKAMPIINKAVNSVFRNPTSLFVRVKAMDMMFNGLPIDCNVTDFAGSAVCSLLREKAEGTLQIDGPDQYRFSLLGAKNDTPSLARTRVLRGVKKLKQIGTVVEHDGKGNISKWDDEFCDTFNGTDGTVFHPKLYEDEDIVAFSPDLCRSISSKYRSKSKAKGISTNHYEASLGDPSTIPEQRCYCEAEDKCLTAGMMDLYKCVGVPLIATLPHFYLTDKQYLTMVDGLKPNPDQHKIFIDFEPFTGSPISARKRVQLNMMIFPIDKVKIIKTFPRALLPLVWIEEGIVLPNWMIRQLKLSHFAIGFMGFVKWMMILTGLVMCGFAAYLMTAADGANKATVKSANIENEKMSHSPNGKSNGIKTISAQLPVSVD

>TpreSNMP2

MATKCRIFGLVVGVIVALVGVLCGFFIFPKVVSNSVAESVKLYDGSDAYRRWEELPIPMKFKVYFFNVTNPGEVQLGHKPVVQEVGPFVYDQYRYKYNISRNTSSNELTYYQNQVLKFNSEDSHPLKETDFVTVANLPLMATINSAMKLKFKIKIISRTMDQLFDKPQDLFLRTTVRDFLFDGIPIRCQERTNPDLIQACNSVDYTSKKTPFIKLQPDNGFKFSLLSHKNNADDGQYTVKSGEDDVQLLGEIKSWKNSSTLIDVWSNETCSKIYGTDSTIFPPHVTESSKLSTFQSDICRSVDLTYQEKTTYRKVDGLRFITDENMLTASRNYSKNACYCLKKTLGITYEDGCLLDGALELQGCLHAPVVMTFPHFYLAADEYKKSVDGLQPVPLLHRTFVDLEPNTGVVLRGSKRAQFNIFYRSVEGIKLTENLTDSLMPVVWFDEGAELNDEMLEILDFSLFSPMRMLDNIVWSLIGLGAVFAVVSLMSCMFRKKT
