# Supplementary material for: Sensory gene identification in the transcriptome of the ectoparasitoid Quadrastichus mendeli
Source: Sci Rep. 2021 May 6;11:9726. doi: 10.1038/s41598-021-89253-w (PMC8102506; doi:10.1038/s41598-021-89253-w)
Supplement: Supplementary file 10 — Additional File 10. [file 41598_2021_89253_MOESM10_ESM.doc]

**Additional information**

Additional file 1: S1.xls-Sequencing summary of the *Quadrastichus mendeli* transcriptome.

Additional file 2: S2.tif-The BLASTx annotations of *Quadrastichus mendeli* transcriptome species distribution.

Additional file 3: S3.xls-GO classification.

Additional file 4: S4.xls-KOG classification.

Additional file 5: S5.xls-KEGG classification.

Additional file 6: S6.xls-Unigenes of candidate proteins.

Additional file 7: S7.doc-The sensory protein fasta sequences of *Quadrastichus mendeli*.

Additional file 8: S8.xls-The candidate proteins transcripts identified in *Quadrastichus mendeli* transcriptomes.

Additional file 9: S9.doc-Amino acid sequence of CSPs, GRs, IRs, OBPs, ORs and SNMPs from organisms other and *Quadrastichus mendeli* used in phylogenetic analyses.
